# MGME1 associates with poor prognosis and is vital for cell proliferation in lower-grade glioma

**DOI:** 10.18632/aging.204705

**Published:** 2023-05-08

**Authors:** Feng Xiao, Jie Zeng, Haiyan Wang, Hong Zhu, Yun Guo, Zhe Zhang, Yao Xiao, Guowen Hu, Kai Huang, Qing Yang, Hua Guo

**Affiliations:** 1 Departments of Neurosurgery, The Second Affiliated Hospital of Nanchang University, Nanchang 330006, Jiangxi, China; 2 Jiangxi Key Laboratory of Neurological Tumors and Cerebrovascular Diseases, Nanchang 330006, Jiangxi, China; 3 Jiangxi Health Commission Key Laboratory of Neurological Medicine, Nanchang 330006, Jiangxi, China; 4 Institute of Neuroscience, Nanchang University, Nanchang 330006, Jiangxi, China; 5 Department of Respiratory Medicine, The Second Affiliated Hospital of Nanchang University, Nanchang 330030, Jiangxi, China; 6 Department of Operation, The Second Affiliated Hospital of Nanchang University, Nanchang 330006, Jiangxi, China

**Keywords:** MGME1, lower-grade glioma, prognosis, immune cell infiltration, treatment responses, cell proliferation

## Abstract

Objective: Mitochondrial genome maintenance exonuclease 1 (MGME1) is associated with DNA depletion, deletion, duplication, and rearrangement. However, the function of MGME1 in tumors, especially lower-grade gliomas (LGGs), has not been established.

Methods: Pan-cancer analysis was used to define the expression patterns and prognostic value of MGME1 in various cancers. Subsequently, we systematically determined the associations between MGME1 expression and clinicopathological characteristics, prognosis, biological functions, immune characteristics, genomic mutations, and therapeutic responses of LGGs based on their expression patterns. The expression level and specific functions of MGME1 in LGGs was detected by conducting *in vitro* experiments.

Results: Abnormally enhanced and high MGME1 expressions were associated with poor prognoses of various tumors, including LGG. Multivariate and univariate Cox regression analyses manifested that MGME1 expression was an independent prognostic biomarker for LGG. The immune-related signatures, infiltration of immune cells, immune checkpoint genes (ICPGs), copy number alteration (CNA), tumor mutation burden (TMB), and treatment responses of LGG patients were associated with the expression of MGME1. The *in vitro* experiments affirmed that MGME1 was elevated and tightly connected with the cell proliferation and cell cycle in LGG.

Conclusions: MGME1 is an independent prognostic biomarker and closely related to the cell proliferation in LGG.

## INTRODUCTION

According to World Health Organization (WHO) statistics, glioma is the most common brain tumor in adults [1], and it is graded from I to IV [2]. Grade II and III gliomas are also designated as lower-grade gliomas (LGGs) in The Cancer Genome Atlas (TCGA) database. Chemotherapy and radiotherapy are used to treat LGG patients; however, their efficacies are suboptimal [3]. Therefore, new effective treatments for LGG need to be established.

Mitochondrial genome maintenance exonuclease 1 (MGME1) was certified as a mitochondrial DNA nuclease. MGME1 participated in the mitochondrial replication by interacting with the POLG, SSBP1, and TWNK and played an important part in maintaining 7S DNA [4, 5]. Loss-of-function mutations of MGME1 may lead to mitochondrial DNA deletions, depletion, rearrangements and duplications [6, 7]. Additionally, MGME1 played a part in the termination of replication and transcription at the end of the control region of mitochondria DNA [8]. During DNA double-strand breaks, MGME1 could cooperate with pol γ and the TWNK helicase to degrade linear mitochondria DNA [9]. This may be connected to the malignant development of some cancers. To clarify the specific roles of MGME1 in LGGs, we conducted a study to explore the specific functions of MGME1 in patients with LGGs.

In the research, we carried out pan-cancer analysis of MGME1 for 33 types of cancers and detected that its prognostic value in pan-LGG was more significant than in other cancers. Thus, it is very important to examine the specific roles of MGME1 in LGG. Afterwards, we further examined the prognostic value of MGME1 in LGGs using three independent cohorts, including the TCGA cohort (*n* = 477), the Chinese Glioma Genome Atlas (CGGA) cohort (*n* = 170), and the GSE16011 cohort (*n* = 102). We separated the samples into high- and low-MGME1 subtypes in the light of the median MGME1 expression in patients with LGG and confirmed that the prognosis of the high-MGME1 subtype was worse than that of the low-MGME1 subtype through survival analysis. We also investigated the relationships between MGME1 expression and age, isocitrate dehydrogenase (*IDH*) status, 1p/19q status gender, WHO classification, and MGMT status by analyzing clinical pathological information. We employed cox regression analysis of the aforementioned clinical indicators to inspect the independent prognostic significance of MGME1 expression for LGGs. The biological functions of MGME1 in LGGs were explored via functional enrichment analysis. We executed the single sample GSEA (ssGSEA) algorithm to ascertain the connection between MGME1 expression and 29 immune-related features, immunological features (such as ICPGs and stromal and immune scores and the expressions of tumor-infiltrating immune cells (TIICs)), genomic alterations and treatment responses. Through *in vitro* experiments, we can affirm the abnormal expression and biological functions of MGME1 in LGGs. MGME1 is an independent prognostic biological marker and might represent a new therapeutic target for LGGs.

## RESULTS

### Pan-cancer analysis of MGME1

**Figure 1:**
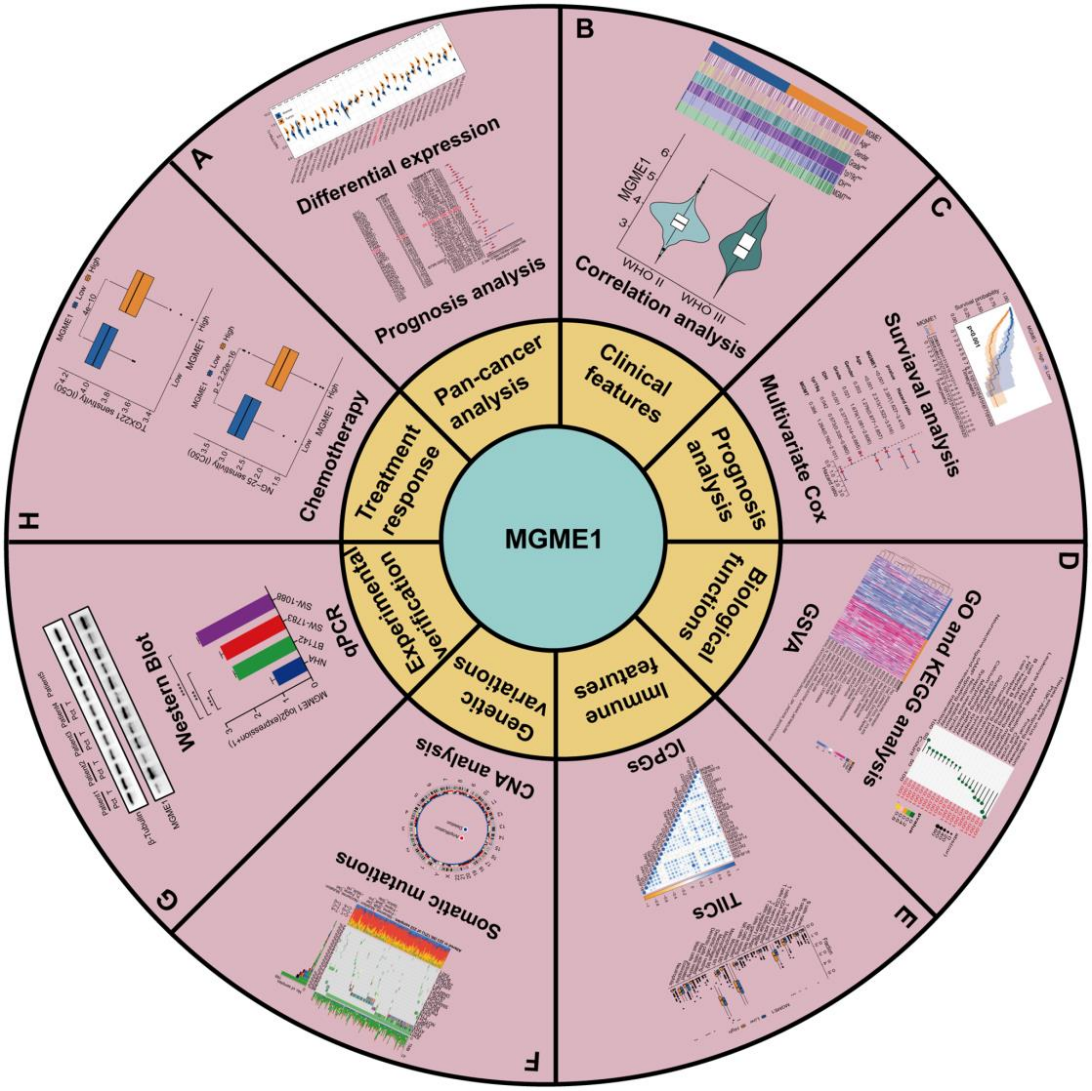
**Flowchart of research.** (**A**) Pan-cancer analysis. (**B**) Clinical features. (**C**) Prognosis analysis. (**D**) Biological functions. (**E**) Immune features. (**F**) Genetic variations. (**G**) Experimental verification. (**H**) Treatment response of MGME1 in LGG.

**Figure 2:**
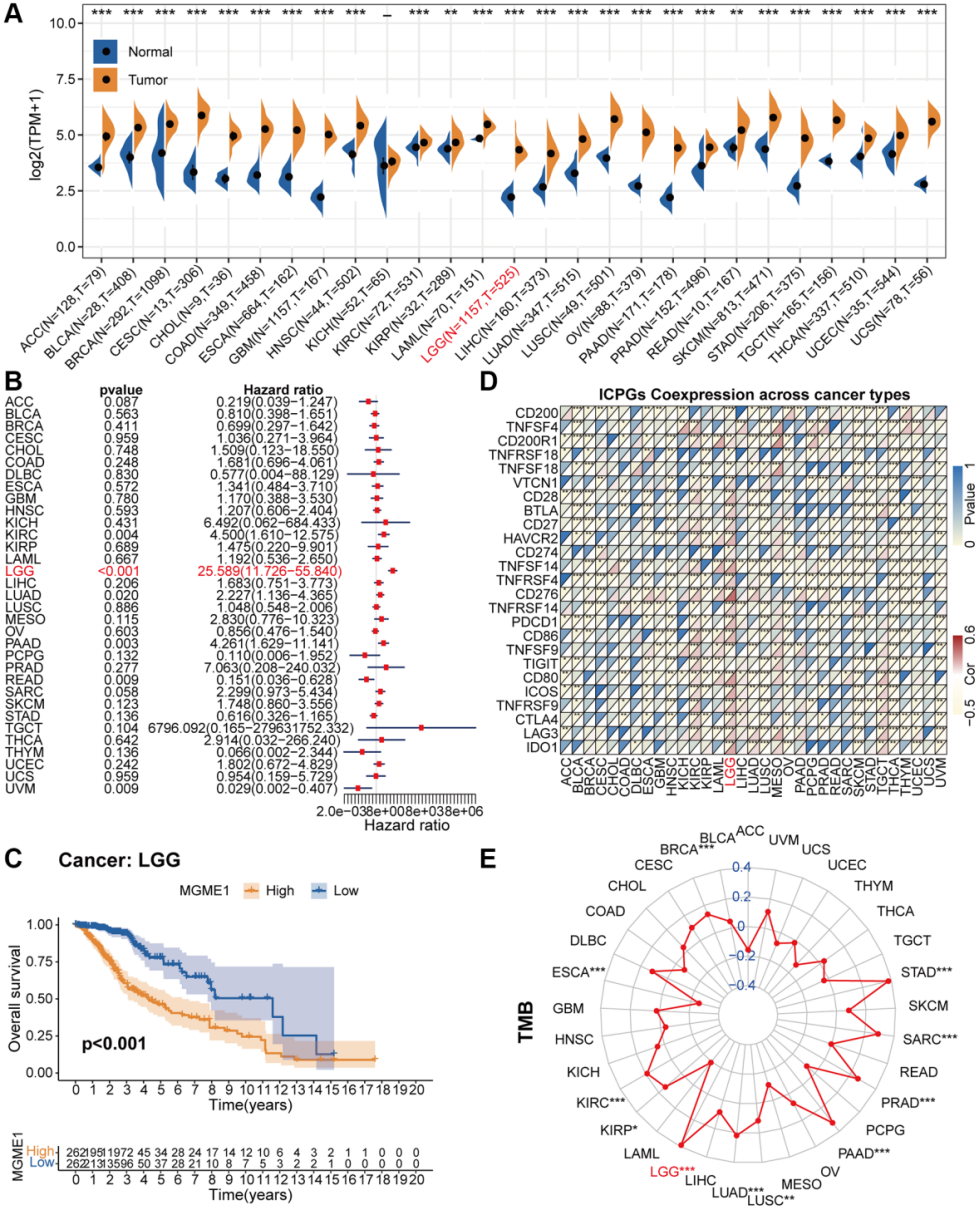
**Pan-cancer analysis of MGME1.** (**A**) Differential expressions of MGME1 in normal and cancer tissues. (**B**) Univariate Cox regression analysis of MGME1 expression in various tumors. (**C**) Kaplan-Meier analysis of MGME1 in pan-LGG. (**D**) Co-expressions of MGME1 and ICPGs in different cancers. (**E**) Differential TMB in various cancers. ^*^P < 0.05, ^**^P < 0.01, ^***^P < 0.001.

Figure 1 is a flow chart showing the entire research process. Comparison of the pan-oncogene expression data obtained from the TCGA and GTEx databases manifested that MGME1 was abnormally upregulated in various cancers. MGME1 was significantly elevated in 24 types of cancers, including ACC, BLCA, BRCA, CESC, CHOL, COAD, ESCA, GBM, HNSC, KIRC, LAML, LGG, LIHC, LUAD, LUSC, OV, PAAD, PRAD, SKCM, STAD, TGCT, THCA, UCEC, and UCS, and slightly in KIRP and READ (Figure 2A).

We exploited a univariant Cox regression analysis to inspect the association between MGME1 expression and OS and determine the prognostic significance of MGME1 in 33 cancer types. As shown in the forest chart, MGME1 expression was negatively correlated with OS for LGG, KIRC, LUAD, PAAD, UVM, and READ (Figure 2B). Survival analysis results also showed that higher expressions of MGME1 indicated worser OS of LGG (Figure 2C), PAAD (Supplementary Figure 1A), SARC (Supplementary Figure 1B), UCEC (Supplementary Figure 1C), and UVM (Supplementary Figure 1D). Additionally, we detected those higher expressions of MGME1 correlated with poorer disease special survival (DSS) of LGG (Supplementary Figure 1E), SARC (Supplementary Figure 1F), UCEC (Supplementary Figure 1G), and UVM (Supplementary Figure 1H).

Subsequently, we explored the correlation between MGME1 and ICPG expressions in 33 tumors. The results manifested that MGME1 expression was interrelated with the expressions of most ICPGs in BLCA, BRCA, KIRC, COAD, ESCA, HNSC, KIRC, KIRP, LGG, LUSC, OV, PRAD, SKCM, THCA, THYM, UCEC, and UVM (Figure 2D). We also probed the association between TMB and MGME1 expression in 33 cancers. The expression of MGME1 and TMB were positively correlated in BRCA, KIRC, LGG, LUAD, PAAD, PRAD, SARC, and STAD, and negatively correlated in ESCA (Figure 2E).

We further conducted separate correlation studies to determine the clinical value of MGME1 in patients with LGG.

### MGME1 and clinicopathological characteristics in LGG

**Figure 3:**
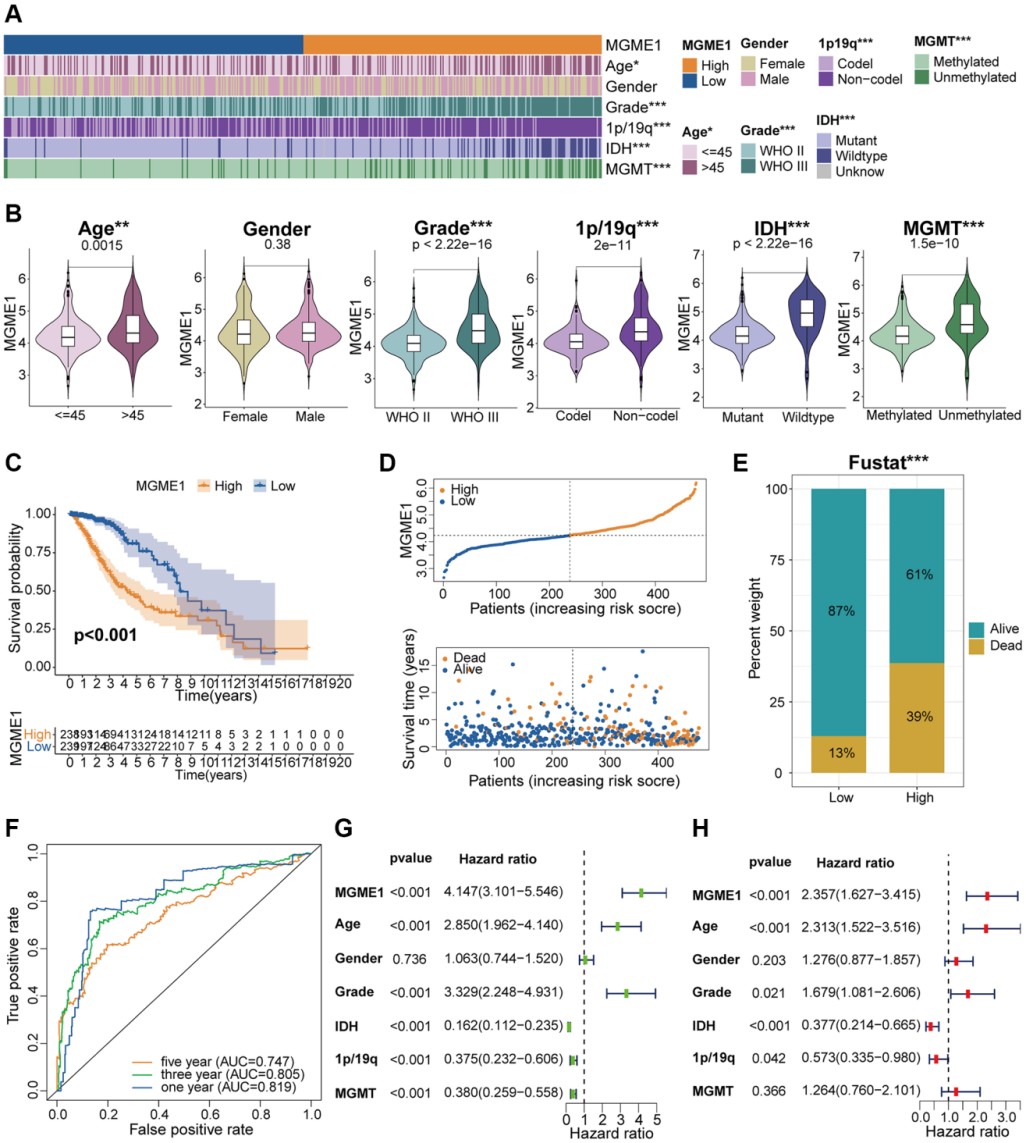
**Correlation analysis of MGME1 in TCGA.** (**A**) Association between MGME1 expression and clinical characteristics of LGG in TCGA. (**B**) Variance analysis of MGME1 expression and different clinical characteristics (including age, gender, grade, and 1p/19q, *IDH*, and MGMT statuses) in the TCGA dataset. (**C**) Prognostic analysis of high-MGME1 and low-MGME1 subtypes in the TCGA dataset. (**D**) Distribution of risk scores and OS of high-MGME1 and low-MGME1 subtypes in the TCGA dataset. (**E**) Distributions of OS of the two subtypes. (**F**) ROC curves representing the predictive value of the risk score in TCGA. (**G**, **H**) Univariate and multivariate Cox regression analyses of MGME1 expression and clinicopathological characteristics in TCGA. ^*^P < 0.05, ^**^P < 0.01, ^***^P < 0.001.

According to the median MGME1 expression, we grouped the patients with LGG into low- and high-MGME1 subgroups and examined the relationships between MGME1 expression and clinicopathological traits in the TCGA, CGGA, and GSE16011 cohorts. The results proved that the upregulation of MGME1 expression was strongly relevant with old age, 1p/19q non-codel, higher WHO grade, *IDH* wildtype, and MGMT unmethylation in the TCGA dataset (Figure 3A, 3B). We obtained the same results for the CGGA (Supplementary Figure 2A, 2B) and GSE16011 (Supplementary Figure 3A, 3B) cohorts. Hence, MGME1 expression was tightly interrelated with the clinicopathological features of LGG patients.

### Adverse prognosis of LGG is associated with increased expression of MGME1

Kaplan-Meier analysis manifested that the OS of the low-MGME1 subgroup was better than that of the high-MGME1 subgroup in the TCGA (Figure 3C), CGGA (Supplementary Figure 2C), and GSE16011 (Supplementary Figure 3C) datasets. We probed into the associations between the expression of MGME1, risk score, and OS of LGG patients and found that the upregulation of MGME1 expression was connected with worse OS and higher risk scores in the TCGA (Figure 3D), CGGA (Supplementary Figure 2D), and GSE16011 (Supplementary Figure 3D) cohorts. We also determined the proportions of patients with LGG with the selected durations of survival in the TCGA (Figure 3E), CGGA (Supplementary Figure 2E), and GSE16011 (Supplementary Figure 3E) cohorts. ROC curves were used to determine the reliability of MGME1 in forecasting the OS of patients with LGG in the three cohorts. The AUCs for 1-, 3-, and 5-year OS were 0.819, 0.805, and 0.747, severally, for the TCGA cohort (Figure 3F); 0.726, 0.795, and 0.780, severally, for CGGA cohort (Supplementary Figure 2F); and 0.733, 0.708, and 0.729, severally, for the GSE16011 cohort (Supplementary Figure 3F). These results forcefully suggest that MGME1 is a prognostic biomarker for patients with LGG.

### Independent prognostic implication of MGME1 in LGG

Multivariate and univariate Cox regression analyses were performed to define whether MGME1 was an independent prognostic indicator for the three cohorts. The results manifested that MGME1 expression, age, *IDH* status, WHO grade, and 1p/19q status were independent prognostic biomarkers of LGG in the TCGA dataset (Figure 3G, 3H). We found that MGME1 expression, WHO grade, and 1p/19q status were independent prognostic factors of LGG in the CGGA cohort (Supplementary Figure 2G, 2H). MGME1 expression and age were also considered independent prognostic factors for LGG in the GSE16011 cohort (Supplementary Figure 3G, 3H). Based on the results for the above three datasets, MGME1 expression is an independent prognostic biomarker for LGG.

### Functions of MGME1 in LGG

**Figure 4:**
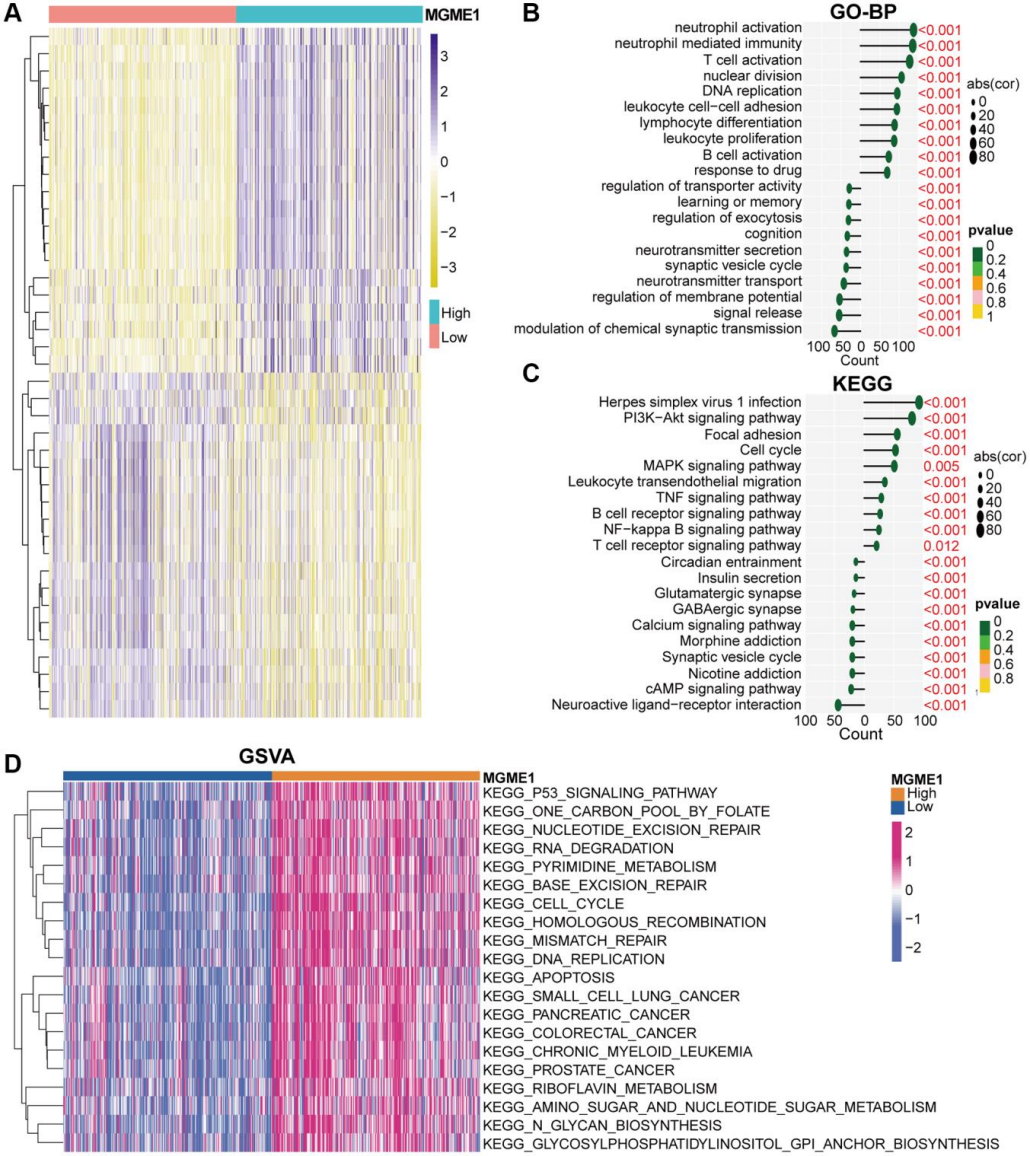
**Biological functions of MGME1 in LGG in TCGA.** (**A**) DEGs in the low-MGME1 and high-MGME1 expression subgroups. (**B**, **C**) The GO-BP (**B**) and KEGG (**C**) analyses for MGME1 in patients with LGG in the TCGA dataset. (**D**) GSVA in the TCGA dataset.

We identified the DEGs based on the average MGME1 (|log2 (fold change)| > 0.5 and *P* < 0.05) expression to assess the effect of MGME1 on the OS of patients with LGG. We selected 600 downregulated (Supplementary Table 1) and 2207 upregulated (Supplementary Table 2) DEGs from the TCGA cohort and 899 downregulated (Supplementary Table 3) and 2253 upregulated (Supplementary Table 4) DEGs from the CGGA cohort. The heatmap shows the DEGs in the TCGA (Figure 4A) and CGGA (Supplementary Figure 4A) datasets. We carried out GO-BP and KEGG analysis to deal with these downregulated and upregulated DEGs. In the TCGA cohort, the downregulation of MGME1 expression was related to the regulation of the modulation of the chemical synaptic transmission, signal release, regulation of membrane potential, and neurotransmitter transport according to the GO-BP analysis results of the downregulated DEGs. In addition, the upregulated genes were mainly enriched for neutrophil activation, T cell activation, neutrophil-mediated immunity, B cell activation leukocyte cell-cell adhesion, and response to drugs (Figure 4B). These results were also obtained for the CGGA cohort (Supplementary Figure 4B). The KEGG pathway analysis of the TCGA (Figure 4C) and CGGA (Supplementary Figure 4C) data indicated that the downregulated DEGs were enriched for neuroactive ligand-receptor interaction, cAMP signal pathway, nicotine addiction, and synaptic vesicle circulation, while the upregulated DEGs were enriched for the PI3K-Akt signal pathway, MAPK signaling pathway, B cell receptor signal pathway, NF kappaB signal pathway, leukocyte transcutaneous migration, cell cycle and T cell receptor signal pathway.

GSVA analysis was used to inspect the molecular pathways for the low- and high-MGME1 isoforms of LGG. The results indicated that the high-MGME1 isoform was mainly interrelated with the cell cycle, DNA replication, and P53 signaling pathway in TCGA (Figure 4D) and CGGA (Supplementary Figure 4D) cohorts.

### MGME1 and immune characteristics

**Figure 5:**
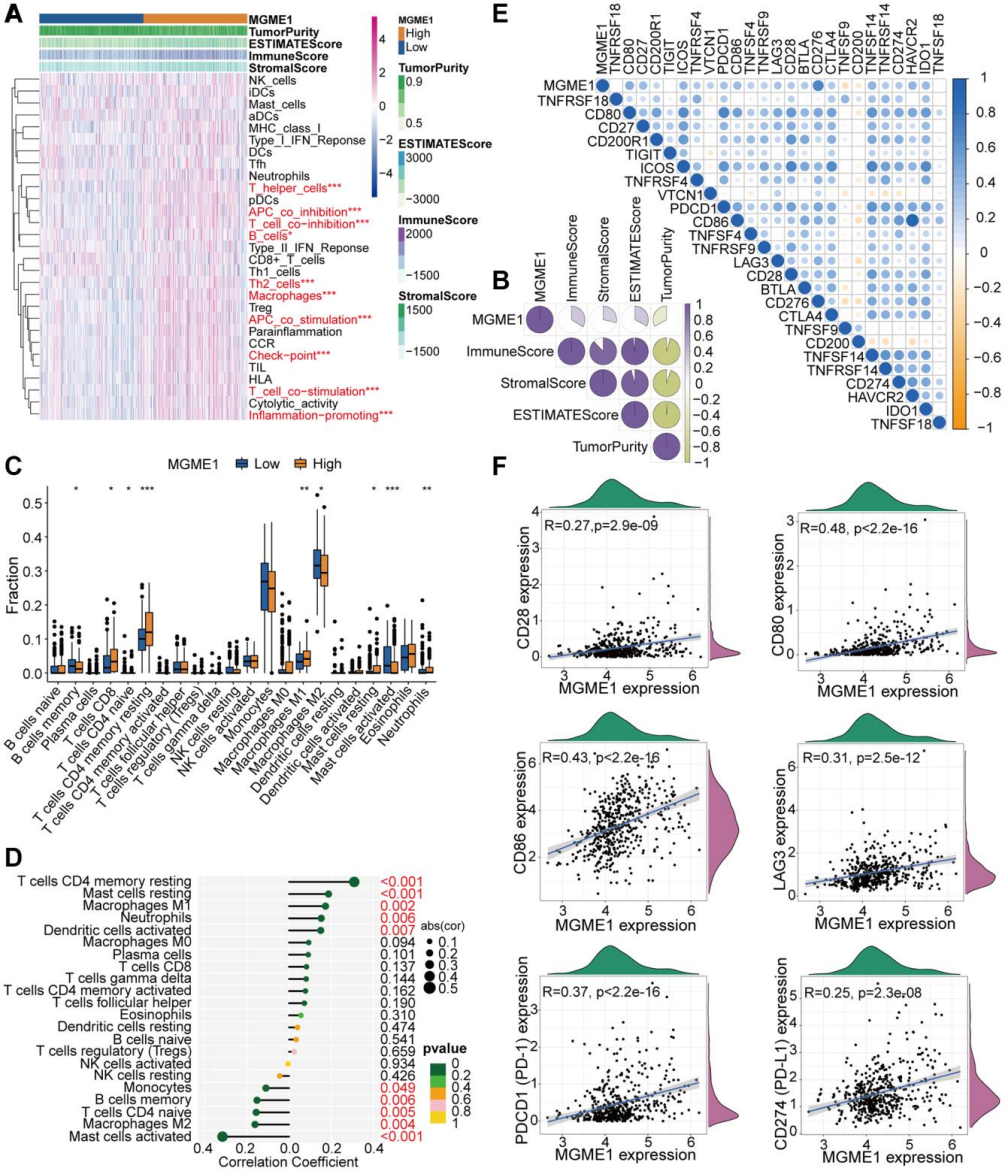
**TME and immunological features of the low-MGME1 and high-MGME1 subtypes in TCGA.** (**A**, **B**) Relationship between MGME1 expression and 29 immune-associated signatures, ESTIMATE scores, immune scores, stromal scores, and tumor purity. (**C**) Comparison of 22 types of immune cell infiltration in the two subgroups. (**D**) Lollipop plots showing the relationships between MGME1 expression and TIICs. (**E**, **F**) Co-expression analysis of MGME1 and 25 ICPGs. ^*^P < 0.05, ^**^P < 0.01, ^***^P < 0.001.

The relationship between MGME1 and immune regulation in LGG was revealed by the GO-BP and KEGG results for the upregulated DEGs. Therefore, we examined the combination of MGME1 and LGG immune characteristics. We implanted the ssGSEA algorithm to identify the abundance of 29 immune-related factors to examine the combination of MGME1 expression and immune infiltration. In the CGGA (Supplementary Figure 5A) and TCGA (Figure 5A) datasets, the low-MGME1 subgroup had significantly fewer immune-related characteristics than the high-MGME1 subgroup. The ESTIMATE algorithm showed that MGME1 expression was positively correlated with estimation, immune scores, and stromal but negatively correlated with tumor pureness in the TCGA (Figure 5B) and CGGA (Supplementary Figure 5B) cohorts. The CIBERSORT algorithm was applied to study the infiltration abundance of TIIC in the two MGME1 subgroups. In the TCGA cohort, the infiltration abundance of resting memory CD4^+^T cells, and macrophage M1 were positively correlated with MGME1 expression, while those of macrophage M2 and B cell memory cells were negatively correlated with MGME1 expression (Figure 5C, 5D). We obtained the same result for the CGCA cohort (Supplementary Figure 5C, 5D).

We executed differential correlation analysis to further investigate the differential expressions of ICPGs and MGME1 in patients with LGG. MGME1 expression and the expressions of most ICPGs in the TCGA cohort presented a positive correlation (Figure 5E). We used correlation analysis to explore the associations between MGME1 and some known ICPGs (such as PD1, PD-L1, LAG3, CD28, CD80, and CD86) in the TCGA dataset (Figure 5F). We obtained the same results for the CGGA cohort (Supplementary Figure 5E, 5F). Therefore, MGME1 may be closely related to the immune microenvironment.

### MGME1 is related to genomic variations

**Figure 6:**
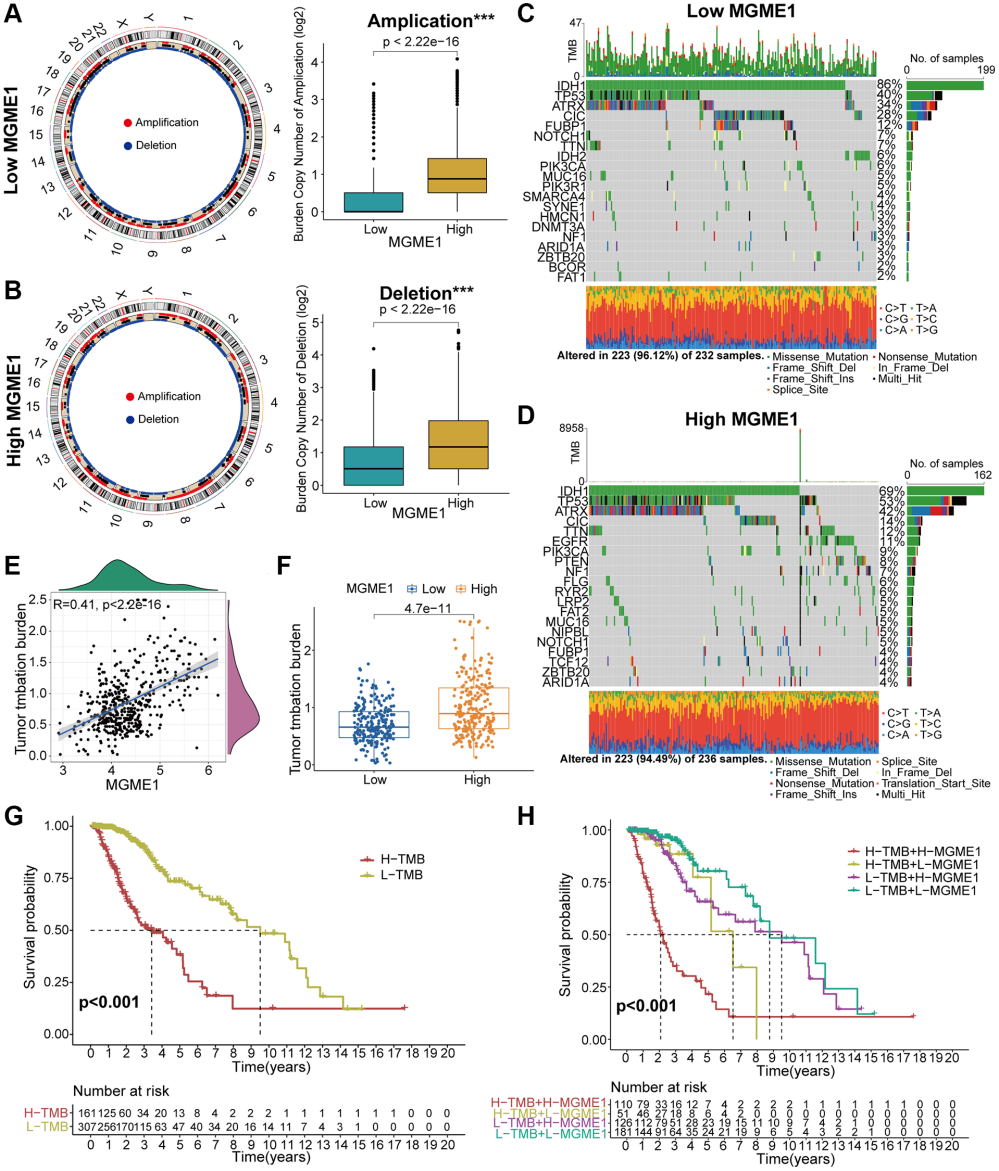
**Contradistinction of genomic mutations in the two subgroups in the TCGA dataset.** (**A**, **B**) Circos plots of low- and high-MGME1 subgroups show the amplifications and deletions of chromosomes, and the boxplots show a lower burden of copy number amplifications and deletions in the low-MGME1 subgroup. (**C**, **D**) The waterfall diagrams show the mutated genes in the low-MGME1 (**C**) and high-MGME1 (**D**) subgroups. (**E**, **F**) An association between MGME1 expression and TMB levels. (**G**, **H**) Association between TMB levels and the prognosis of patients with LGG (**G**) and the differential prognostic value in the two subtypes with different TMB level (**H**). ^*^P < 0.05, ^**^P < 0.01, ^***^P < 0.001, ^****^P < 0.0001.

Several research have indicated that genomic variations may fulfil a crucial role in regulating immune invasion and tumor immunity [10–12]. Given the value of genomic variations in tumor immune regulation and infiltration, we used CNA and somatic mutation analysis to distinguish the differential genomic mutations in the low-MGME1 and high-MGME1 expression subgroups. The frequency of CNAs, including amplification and deletion, in the low-MGME1 subgroup was sensibly lower than that in the high-MGME1 subgroup (Figure 6A, 6B). Subsequently, we established a “waterfall” map of somatic mutations to show that low-MGME1 expression subgroups had specific mutant genes. The mutation frequencies of *IDH1* and *CIC* in the high-MGME1 subgroup were lower than those in the low-MGME1 subgroup. Nevertheless, the mutation frequencies of *TP53* and *ATRX* were higher in the high-MGME1 than in the low-MGME1 subgroup (Figure 6C, 6D). The expression of MGME1 was positively correlated with TMB in patients with LGG (Figure 6E, 6F). Besides, MGME1 expression and TMB levels were negatively interrelated with the OS of LGG patients (Figure 6G, 6H). These results suggested that LGG patients with high MGME1 expression may show special immunological models.

### *In vitro* study of MGME1 expression in patients with LGG

**Figure 7:**
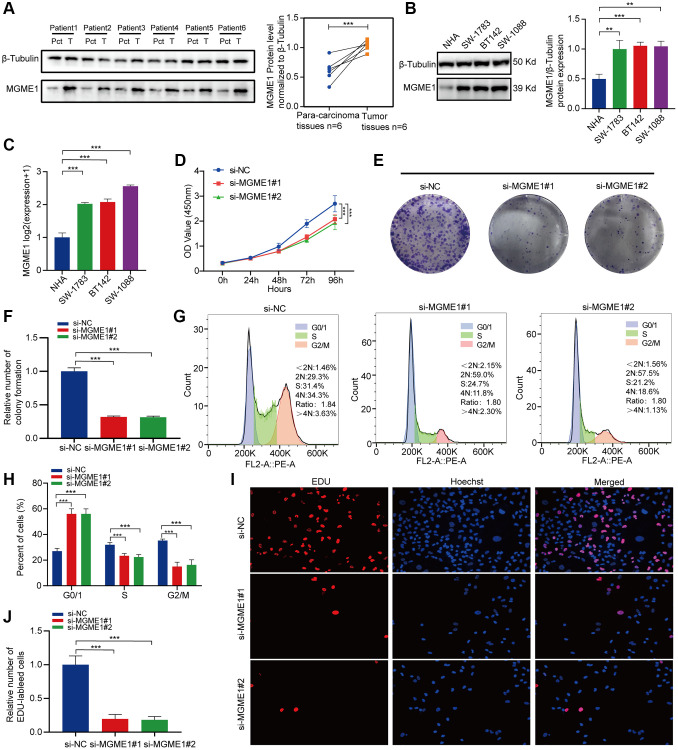
***In vitro* experimental verification of MGME1 in LGG.** (**A**) Western blot analysis of MGME1 expression in LGG tissues and corresponding para-carcinoma tissues. (**B**) Western blot and (**C**) qRT-PCR analysis of MGME1 expression in NHA and LGG cell lines. (**D**) The cell viability of si-MGME1-transfected and si-NC-transfected SW1088 cells by CCK-8 assays. (**E**, **F**) Effect of MGME1 knockdown on colony formation was counted in SW1088 cells. (**G**, **H**) Cell cycle assays were executed to assess the cell cycle distribution of the SW1088 cells transfected with si-MGME1 or si-NC lentiviruses. (**I**, **J**) Representative images (**I**) and histogram analysis (**J**) of EdU assays after silencing MGME1 in SW1088 cells.

Protein expressions levels of MGME1 in LGG tissues were sensibly higher, and the results were analyzed using ImageJ software (Figure 7A). We assessed the protein and mRNA expressions of MGME1 in these LGG cell lines, including the SW-1783, SW-1088, BT142, and NHA lines, and found that the MGME1 expression in the NHA cell lines was lower than that in the LGG cell lines (Figure 7B, 7C).

Afterwards, we performed functional experiments to rate the relationship between LGG cells and MGME1 expression in different groups. CCK-8 assays (Figure 7D), and colony formation assays (Figure 7E, 7F) manifested that the proliferation ability of SW1088 si-MGME1 group was noteworthily decreased compared with the si-NC group. What’s more, we found that downregulating MGME1 expression had a strong effect on cell cycle. Specifically, after MGME1 silencing in SW1088 cells, the number of cells in G0/G1 phase increased, while the number of cells in S and G2/M phase decreased (Figure 7G, 7H). Moreover, the downregulation of MGME1 expression could significantly restrain cell proliferation by EdU assays in SW1088 cells (Figure 7I, 7J). These results suggest that MGME1 was closely connected with the cell proliferation and cell cycle of LGG cells *in vitro*.

### Association between MGME1 expression and therapeutic response

**Figure 8:**
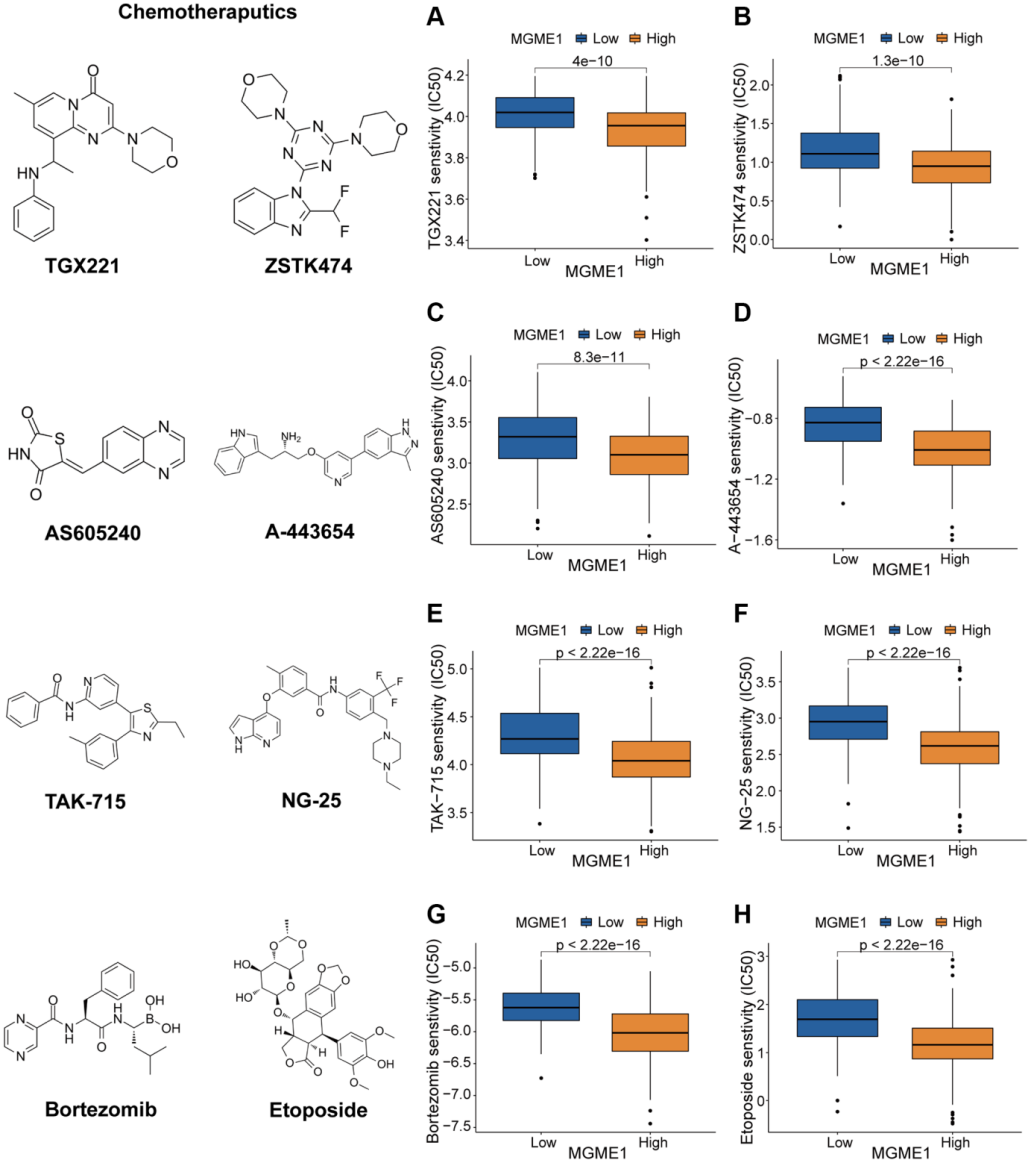
**Different responses to chemotherapy of the low-MGME1 and high-MGME1 subtypes in the TCGA dataset.** (**A**–**D**) the PI3K/AKT inhibitors: TGX221 (**A**), ZSTK474 (**B**), AS605240 (**C**), and A-443654 (**D**). (**E**, **F**) the MAPK inhibitors: TAK-715 (**E**) and NG-25 (**F**). NF kappaB inhibitor: bortezomib (**G**). DNA replication inhibitor: Etoposide (**H**).

We determined the associations between MGME1 expression and PI3K/AKT inhibitors: TGX221, ZSTK474, AS605240, and A-443654 (Figure 8A–8D); the MAPK inhibitors: TAK-715 and NG-25 (Figure 8E, 8F); NF kappaB inhibitor: bortezomib (Figure 8G); DNA replication inhibitor: Etoposide (Figure 8H) to assess the value of MGME1 expressions in guiding chemotherapy. The high MGME1 expression was relevant to the lower inhibitory centration (IC50) of these anti-cancer drugs. In effect, the high-MGME1 expression subtype was more susceptible to the anti-cancer drugs. Therefore, these anti-cancer drugs may be used for chemotherapy for patients with LGG with high MGME1 expressions in the future.

## DISCUSSION

Despite the advances of surgery and postoperative comprehensive treatment of LGG, the overall clinical prognosis of patients with LGG is still poor [13, 14]. Thus, it is necessary to define the effective prognosis and treatment goals of these patients. MGME1 is key to the regulation of cell proliferation. And yet, the role of MGME1 in LGG is unknown. Therefore, we comprehensively studied the relationships between MGME1 expression, clinical characteristics, tumor immunity, gene mutations, prognosis, biological functions, and treatment responses of LGG patients.

We used pan-cancer analysis of MGME1 for 33 cancers. The results showed that higher expressions of MGME1 were interrelated with shorter survival durations of patients with pan-LGG, higher ICPG expressions, and higher TMB. To assess the prognostic role of MGME1 in LGG, we conducted survival analysis for the TCGA cohort and found that the prognosis of the high-MGME1 subgroup was worse than that of the low-MGME1 subgroup. The upregulation of MGME1 expression was associated with worse OS. ROC curves and AUC values were exploited to confirm the accuracy of MGME1 expression for predicting the OS of LGG patients. The connection between MGME1 expression and clinicopathological characteristics of patients with LGG further confirmed that there were significant differences between the clinical factors. In addition, MGME1 was an independent prognostic biomarker of LGG, which was confirmed by Cox regression analysis. Analogous results were for the CGGA and GSE16011 datasets. Thus, MGME1 could be a forceful prognostic biomarker of LGG patients.

We analyzed the upregulation of DEGs in the TCGA and CGGA cohorts using KEGG and GO-BP enrichment analyses. The increase in MGME1 expression was observed in the PI3K-Akt signal pathway, MAPK signaling pathway, B cell receptor signal pathway, NF kappaB signal pathway, leukocyte transcutaneous migration, cell cycle and T cell receptor signal pathway. The high MGME1 expression was majorly related to DNA replication, the cell cycle, and P53 signaling pathway, which was confirmed by GSVA analysis.

**Figure 9:**
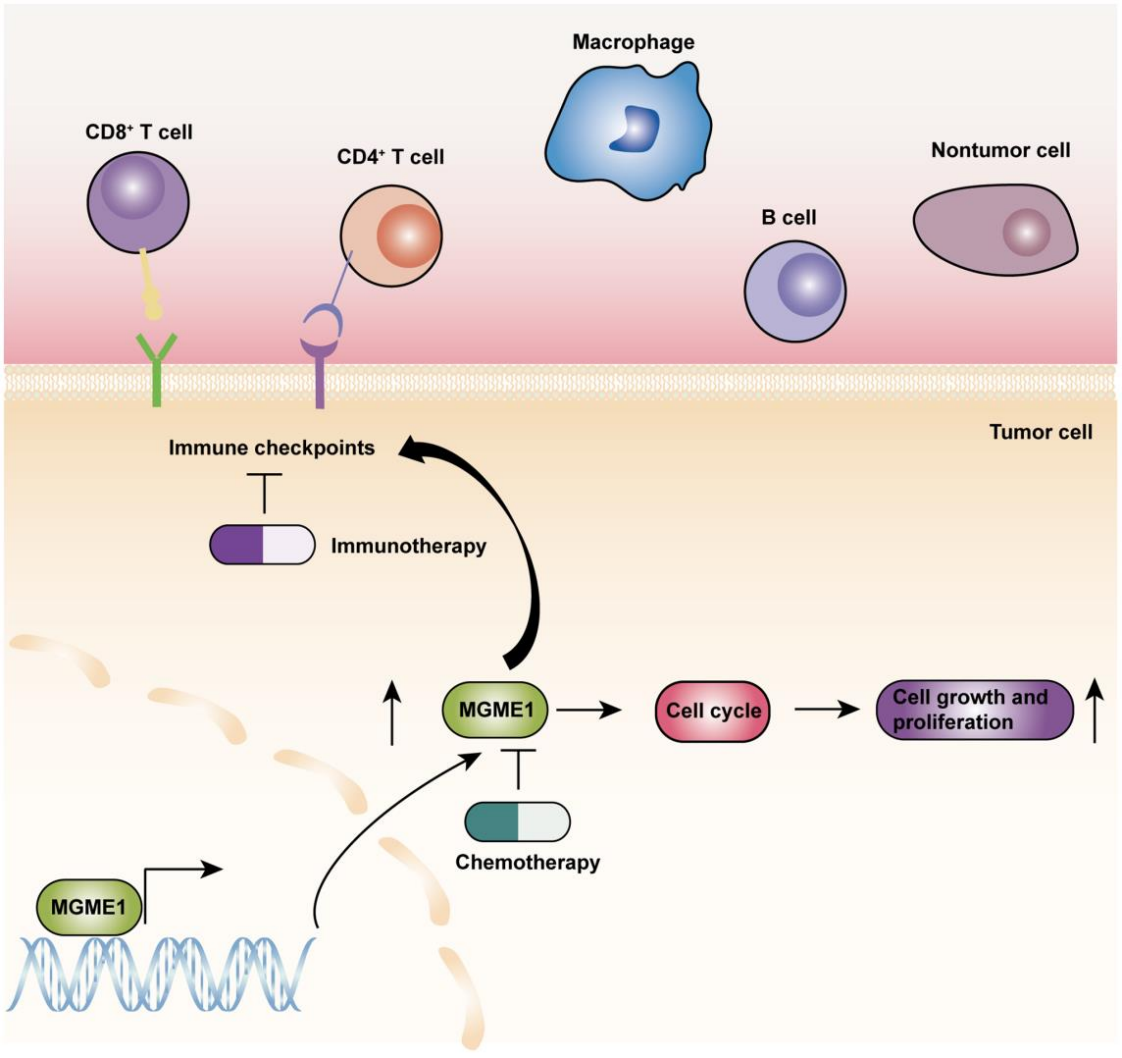
The underlying biological mechanisms of MGME1 in LGG.

On the basis of the results of GO-BP, KEGG, and GSVA analyses, we exploited the ssGSEA, ESTIMATE, and CIBERSORT algorithms to inspect the differences in the immune-related features of the two subgroups in the CGGA and TCGA cohorts and determine the compositions of tumor microenvironment and tumor-infiltrating immune cells. These results manifested that the expression of MGME1 is closely correlated to immune infiltration in LGG. The activation of specific immune cells in the tumor microenvironment (TME) has become the new strategy of immunotherapy for the treatment of tumors [15–17]. As a new immunotherapeutic drug, immune-checkpoint blockade has demonstrated good efficacy for the treatment of different types of tumors [18–20]. Hence, we investigated the relationship between MGME1 and ICPG expressions in patients with LGG. We found that the expressions of some common ICPGs, including PD1, PD-L1, LAG3, CD28, CD80, and CD86, and the expression of MGME1 based on the CGGA and TCGA datasets showed a positive correlation. In addition, somatic mutation and CNA analyses showed that the TMB and CNA loads of the high-MGME1 expression subgroup were higher than those of the low-MGME1 expression subgroup. In summary, MGME1 may play an important part in immunotherapy for patients with LGG. The underlying molecular mechanisms of MGME1 expression in LGG are shown in Figure 9.

At present, TMZ-targeted chemotherapy is the most frequently used treatment for LGG patients [21]. Nevertheless, its effectiveness is limited. Therefore, it is necessary to excavate novel chemotherapy drugs that may be exploited to treat LGG. The chemotherapy sensitivity analysis showed that the high-MGME1 subtype was more responsive than the low-MGME1 subtype to chemotherapy, such as TGX221, ZSTK474, AS605240, A-443654, TAK-715, NG-25, bortezomib, and etoposide. MGME1 may be a latent predictor of chemosensitivity in patients with LGG. Based on the above results, we conducted *in vitro* experiments to confirm that MGME1 was elevated and vital for cell proliferation and cell cycle in LGG. Importantly, we detected that the proliferation ability of the LGG cells was impaired after knockdown the MGME1. Nevertheless, there are still some limitations in the study. The underlying roles of MGME1 in LGG should be inspected by performing *in vivo* and *in vitro* experiments in the future research. Additionally, further research should be adopted to examine whether MGME1 is an effective therapeutic target for LGG patients.

## CONCLUSION

This research ascertained that MGME1 was a forceful prognostic biomarker and tightly connected with cell proliferation and cell cycle of LGG. Therefore, MGME1 may an effective therapeutic target for LGG patients.

## METHODS

### Data acquiring and processing

We carried out a pan-cancer analysis of gene expression data, survival data, and clinicopathological information collected from public databases. We obtained data on the expression of MGME1, related clinical information, and the TMB of 33 tumor types from TCGA database. MGME1 expression data of normal tissues were obtained from Genotypic-Tissue Expression (GTEx).

We used three independent LGG datasets, including TCGA, CGGA (CGGA_325), and GSE16011. The gene expression data, survival data, and clinical pathological information were gathered from CGGA (http://www.cgga.org.cn/), TCGA (https://portal.gdc.cancer.gov/), and Gene Expression Omnibus (GEO; http://www.ncbi.nlm.nih.gov/geo/) websites. RNA-seq expression data, in FPKM format, were converted to TPM values. To facilitate comparison, we converted the transformed TPM values for the RNA-seq data and the robust multichip average analysis processing values of GSE16011 by log2. We also obtained genome variation data of the LGG samples from the TCGA dataset.

### Samples inclusion criteria

Patients with LGG who conformed to the following standards were contained in the present study: (1) WHO grade II and III gliomas; (2) availability of gene expression information; and (3) overall survival (OS) of >30 days. Of the LGG samples used in this study, 477 (Supplementary Table 5), 170 (Supplementary Table 6), and 102 (Supplementary Table 7) were obtained from CGGA, TCGA, and GEO databases, respectively. We added the data of patients with LGG with OS of <30 days to the pan-cancer analysis of MGME1 to ensure the uniformity of survival data for the 33 cancer types.

### Predictive value of MGME1 and related verification

The LGG samples were split into the low- and high-MGME1 subtypes on the basis of the median MGME1 expression in the three datasets. We used Kaplan-Meier analysis to identify the prognosis of LGG in patients with the low- and high-MGME1 subtypes. We used the survival status ratio, receiver operating characteristics (ROC), and area under curve (AUC) values. Cox regression analysis was applied to determine the value of MGME1 expression as an independent biomarker for LGG in the three cohorts.

### Functional enrichment analysis

Using a false-discovery rate (FDR) of <0.05 and |log2FC| of >0.5, we employed the R package, “limma,” to examine the differentially expressed genes (DEGs) in the two subgroups [22]. Based on the DEGs, we exploited the R package, “clusterProfiler,” to perform Gene Ontology biological process (GO-BP) and Kyoto Encyclopedia of Genes and Genomes (KEGG) enrichment analyses [23]. We executed Gene set variation analysis (GSVA) to evaluate the molecular pathways with significant enrichment in the low- and high-MGME1 subgroups [24]. According to the standards of |log2 FC |>0.1, *p* < 0.05, and FDR < 0.05, the most abundant molecular pathways between the two subgroups were determined by using KEGG analysis (c2.cp.kegg.v7.2.symbols) genesets.

### Immunological features of LGGs

Immunological signatures, the abundance of immune cells and stromal cells, and the level of expression of ICPGs are immunological features. First, we obtained immune-related signatures from previous studies [25, 26] and exploited the ssGSEA algorithm to distinguish the differential enrichment of 29 immune-related features of the low- and high-MGME1 subtypes. In the light of the expression profiles of the patients with LGG, the ESTIMATE algorithm was applied to assess the enrichment of stromal and immune cells and tumor purity [27, 28]. Next, we measured four types of scores, including tumor purity, estimated score (representing non-tumor complex), stromal score (representing the richness of stromal cells), and immune score (representing the richness of immune cells).

Thereafter, the level of infiltration of TIICs was determined using the CIBERSORT algorithm in line with the gene expression data of patients with LGGs [29]. We also selected 25 ICPGs with potential therapeutic value based on previous studies [30] and studied their association with MGME1 expression.

### Genomic mutation analysis

Using the RCircos tool, we identified and visualized significant deletions and amplifications in the entire genomes of the low and high MGME1 expression subgroups [31]. The types and frequencies of gene mutations in the low- and high-MGME1 subgroups were explained and visualized using Maftools and GenVisR [32, 33]. Currently, the newly developed biomarker TMB for predicting immunotherapeutic response reflects the total number of non-synonymous mutations. First, the combination between MGME1 expression and TMB level in 33 tumor types was explored using the R package “fmsb.” At the same time, the binding between MGME1 expression and TMB was assessed using the “ggplot2” R package in an independent LGG TCGA cohort.

### Therapeutic responses of MGME1

We investigated the difference between the responses of the low- and high-MGME1 subtypes to several chemotherapeutic drugs using the “pRRophetic” R package [34]; the chemotherapy drugs included PI3K/AKT inhibitors (TGX221, ZSTK474, AS605240, and A-443654), MAPK inhibitors (TAK-715 and NG-25), and proteasome inhibitors (bortezomib and Etoposide).

### Cell culture and transfection

We obtained normal human astrocyte (NHA) cell line from the Culture Collection of the Chinese Academy of Sciences (Shanghai, China), and SW-1088, SW-1783, BT142 human LGG cell lines from the American Typical Culture Collection (ATCC). Dulbecco’s modified Eagle’s/F12 (ATCC) medium was used to culture the NHA and BT142 cell lines. Leibovitz’s L-15 medium (ATCC), which contained 10% fetal bovine serum (Gibco), was used to culture the SW-1088 and SW-1783 cell lines. The above cell lines were cultured with 37°C and 5% CO2. We obtained a lentivirus expressing MGME1 shRNA from Obio Technology (Shanghai, China), where the target sequence of MGME1 shRNA was 5′-GCTTAATTGTGGTGGCCTACA-3′. According to the protocol, lentivirus shRNA and negative control (NC) vectors were transfected into SW1088 cell line. The multiplicities of infection (MOIs) were 10 in SW1088 cells. Additionally, the transfection efficiency was improved by polybrene, and the positive cells were filtered by puromycin.

### Western blot analysis and quantitative real-time PCR

The Ethics Committee of the Second Affiliated Hospital of Nanchang University had authorized the use of human tissue in this research. We utilized a radioimmunoprecipitation assay buffer (Solarbio, China) containing a mixture of protease inhibitors to extract cell and human tissue lysates. We exploited 10% sodium dodecyl sulfate-polyacrylamide gel electrophoresis to separate the pyrolysis product, which was shifted to the polyvinylidene fluoride (PVDF) membrane and incubated with primary antibodies, including MGME1 (1:2000, 67468-1-Ig, Proteintech, China) and beta-tubulin (1:2000, 10068-1-AP, Proteintech, China). Subsequently, the PVDF membranes were further incubated with related secondary antibodies. Finally, we incubated the membranes with an enhanced chemiluminescence (ECL) substrate (Thermo Fisher Scientific, USA) and observed the protein bands on the membranes through the GV6000M imaging system (GelView6000pro). We employed the Simple P total RNA extraction kit (Biolux, China) to isolate the RNA from the cells and HiScript III-RT SuperMix (Vazyme, China) to reverse transcribe it to complementary DNA. We used the 2^-ΔΔCT^ method to process the result. The primer sequences of these genes were as below: forward MGME1 primer, 5′-TGTGGCTTAATTGTGGTGGC-3′; reverse MGME1 primer, 5′-AGTCGAAGAAGCCACTTGGT-3′; beta-tubulin forward primer, 5′-ACGCGGTTCTGTCTATCCAC-3′; and beta-tubulin reverse, 5′-GAGGTGGTTATGCCGCTCAT-3′.

### CCK-8 assay

The transfected SW-1088 cells were sowed in 96-well plates at 2 × 10^3^ per well and cultured for 4 days. Cell multiplication was checked by Cell Counting Kit 8 assay (Glpbio, USA, GK10001) on the basis of the protocol.

### Colony formation assay

The 2×10^3^ cells/well were plated in 6-well plates and cultured for 2 weeks. The cells were then stained with 0.1% crystal violet solution, and the number of colonies was recorded by ImageJ.

### EdU assay

The transfected SW-1088 cells (2 × 10^4^) were sowed in 24-well plates and incubated for 72 hours. Next, we cultured the cells with EdU reagent for 2 h. The cells were fixed by 4% paraformaldehyde and 0.5% Triton X-100, and then stained by the Hoechst staining. ImageJ was used to calculate the EdU inclusion rate.

### Cell cycle analysis

The transfected SW-1088 cells were immobilized with 70% ethanol at 4°C overnight and stained with RNase A containing propidium iodide (Suzhou, China). The cell cycle distribution was surveyed by implementing flow cytometer.

### Statistical analysis

Kaplan-Meier analysis using a two-sided logarithmic rank test was utilized to distinguish the prognosis of the high-MGME1 subset from that of the low-MGME1 subset. The AUC values and ROC curves were applied to verify the exactitude of the prognostic prediction on the basis of MGME1 expression. Cox regression analysis was applied to estimate the independent prognostic significance of MGME1. Student’s *t*-test was employed to contrast the immune-related factors associated with the two subtypes, such as 29 immune-related characteristics, TIIC, 25 ICPGs, TMB, and CNA loads. We conducted Pearson’s or Spearman’s correlation test to determine the relevance between the distributed variables. Wilcoxon’s signed-rank test was employed to inspect the discrepancy in sensitivity between the two subtypes of anticancer drugs. We used R version 4.1.0, GraphPad Prism 8 (GraphPad Software, Inc., USA), and SPSS Statistics to perform the statistical analyses. The results were supposed to be significant for *p*-values of < 0.05.

### Availability of data and materials

The data analyzed in this research can be found in the TCGA (https://portal.gdc.cancer.gov/), CGGA (http://www.cgga.org.cn/), and GEO (http://www.ncbi.nlm.nih.gov/geo/) websites.

## Supplementary Materials

Supplementary Figures

Supplementary Table 1

Supplementary Table 2

Supplementary Table 3

Supplementary Table 4

Supplementary Tables 5-7
