# Supplementary material for: MGME1 associates with poor prognosis and is vital for cell proliferation in lower-grade glioma
Source: Aging (Albany NY). 2023 May 8;15(9):3690–714. doi: 10.18632/aging.204705 (PMC10449294; doi:10.18632/aging.204705)
Supplement: Supplementary Figures [file aging-15-204705-s001.pdf]

SUPPLEMENTARY FIGURES

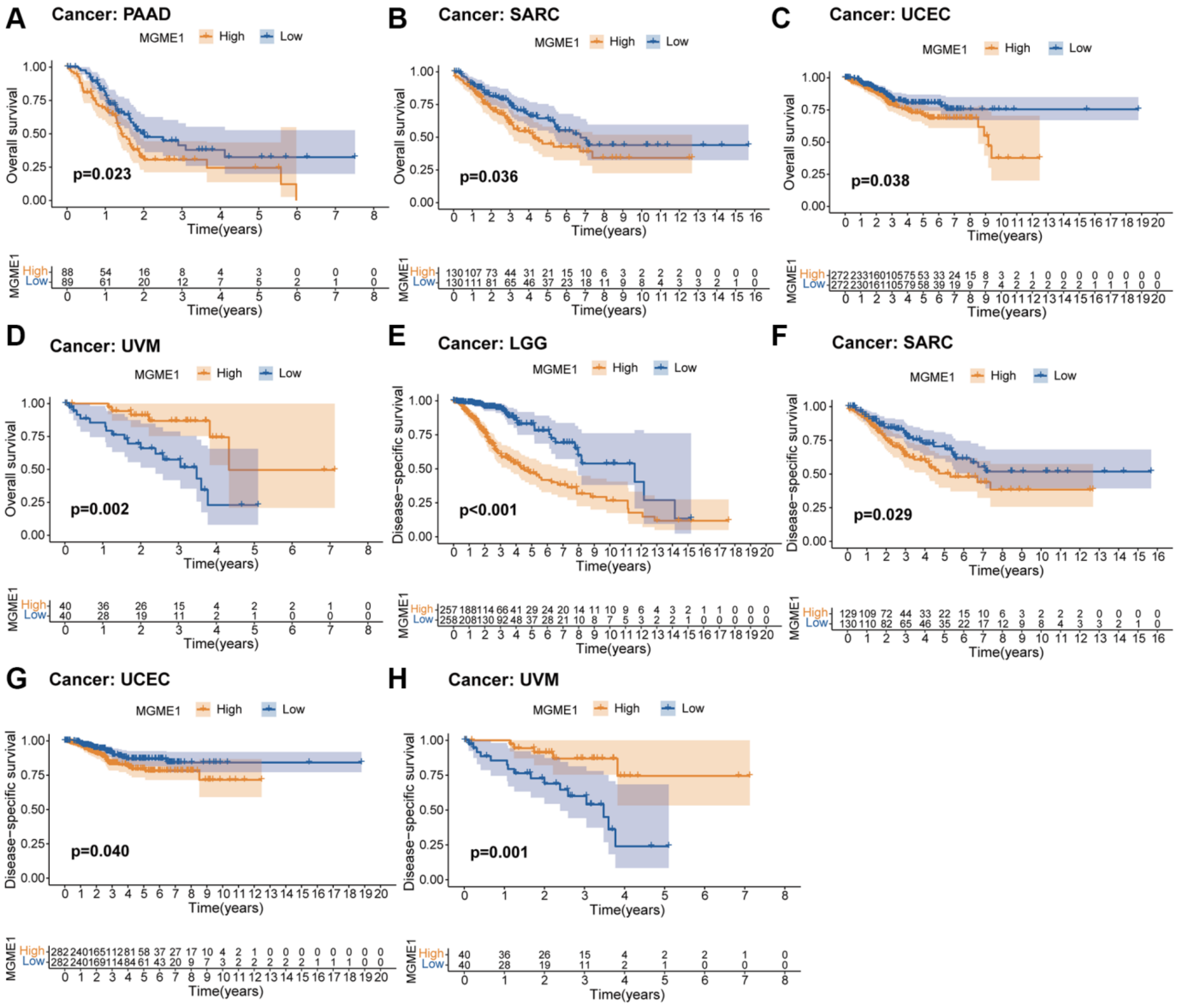

Supplementary Figure 1. Kaplan-Meier analysis of MGME1 in pan-cancer. (**A**–**D**) Correlation between MGME1 expression and OS of PAAD (**A**), SARC (**B**), UCEC (**C**), and UVM (**D**). (**E**–**H**) Correlation between MGME1 expression and disease special survival of LGG (**E**), SARC (**F**), UCEC (**G**), and UVM (**H**).

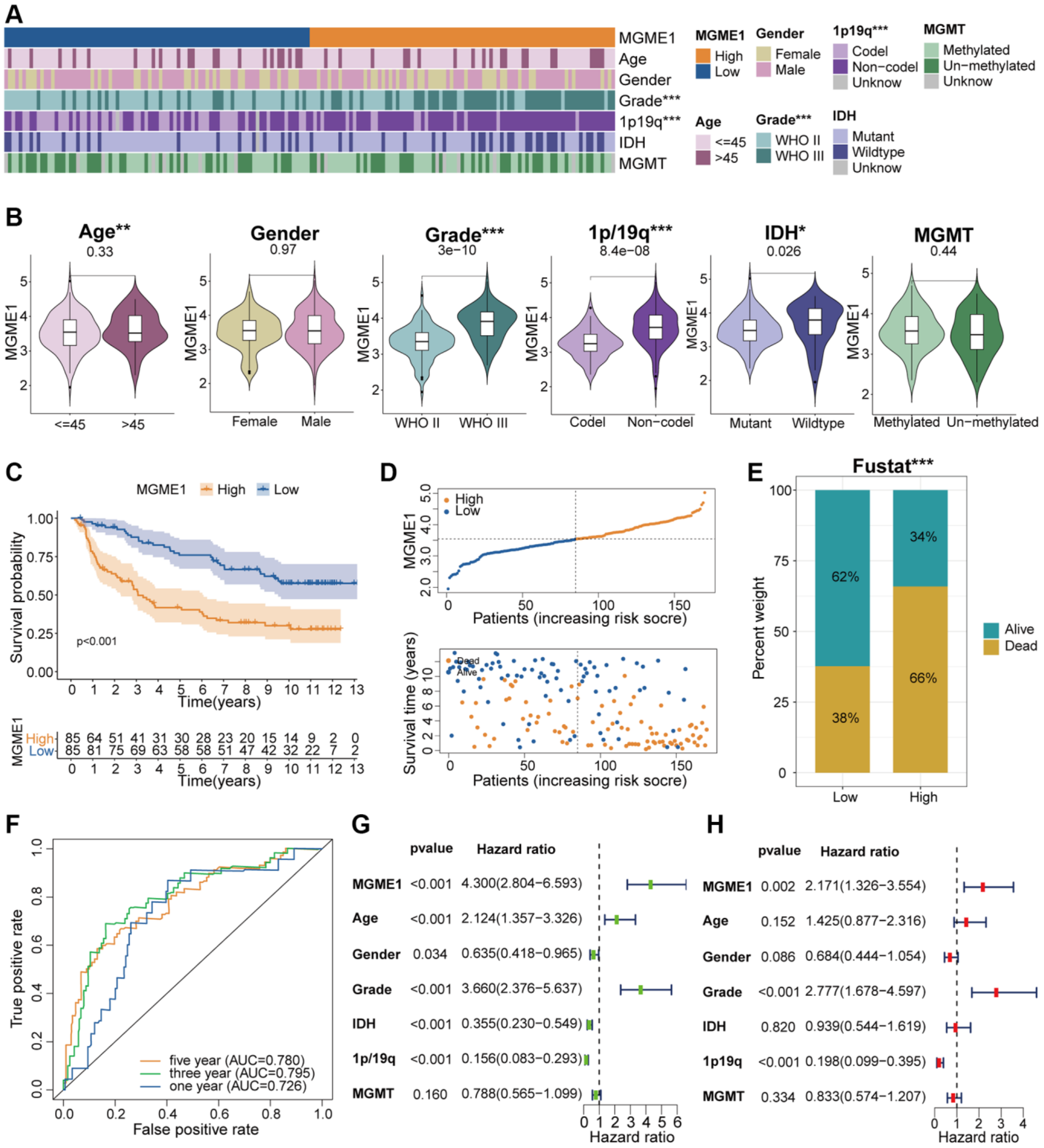

Supplementary Figure 2. Clinical correlation analysis of MGME1 in CGGA. (**A**) Association between MGME1 expression and clinical characteristics of LGG in CGGA. (**B**) Variance analysis of MGME1 expression and various clinical features (including age, gender, grade, and 1p/19q, *IDH*, and MGMT statuses) in the CGGA dataset. (**C**) Prognostic analysis of high-MGME1 and low-MGME1 subtypes in the CGGA dataset. (**D**) Distribution of the risk score and OS of the high-MGME1 and low-MGME1 subgroups in the CGGA dataset. (**E**) Survival analysis of the two subgroups. (**F**) ROC curves representing the predictive role of the risk score in CGGA. (**G**, **H**) Univariate and multivariate Cox analyses of MGME1 expression and clinicopathological features in CGGA. $^{*}P < 0.05$, $^{**}P < 0.01$, $^{***}P < 0.001$.

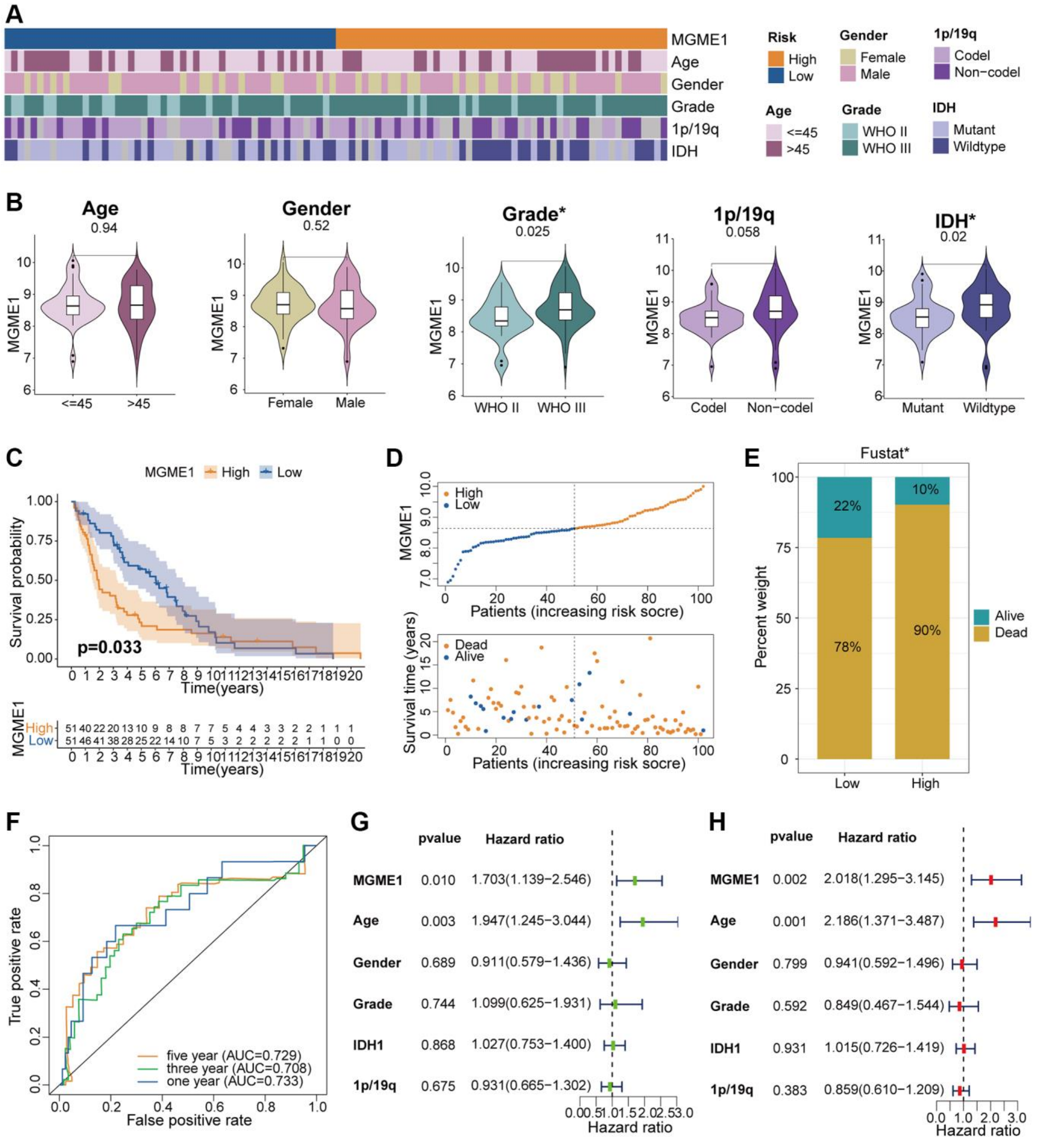

Supplementary Figure 3. Clinical correlation analysis of MGME1 in the GSE16011 dataset. (**A**) Association between MGME1 expression and clinical characteristics of LGG in the GSE16011 dataset. (**B**) Variance analysis of MGME1 expression and different clinical features (including age, gender, grade, and 1p/19q, *IDH*, and MGMT statuses) in the GSE16011 dataset. (**C**) Prognostic analysis of high-MGME1 and low-MGME1 subtypes in the GSE16011 dataset. (**D**) Distribution of risk score and OS of high-MGME1 and low-MGME1 subgroups in the GSE16011 dataset. (**E**) Survival of the two subgroups. (**F**) ROC curves representing the predictive role of the risk score in GSE16011. (**G**, **H**) Univariate and multivariate Cox regression analyses of MGME1 expression and clinicopathological characteristics in GSE16011. $^{*}P < 0.05$, $^{**}P < 0.01$, $^{***}P < 0.001$.

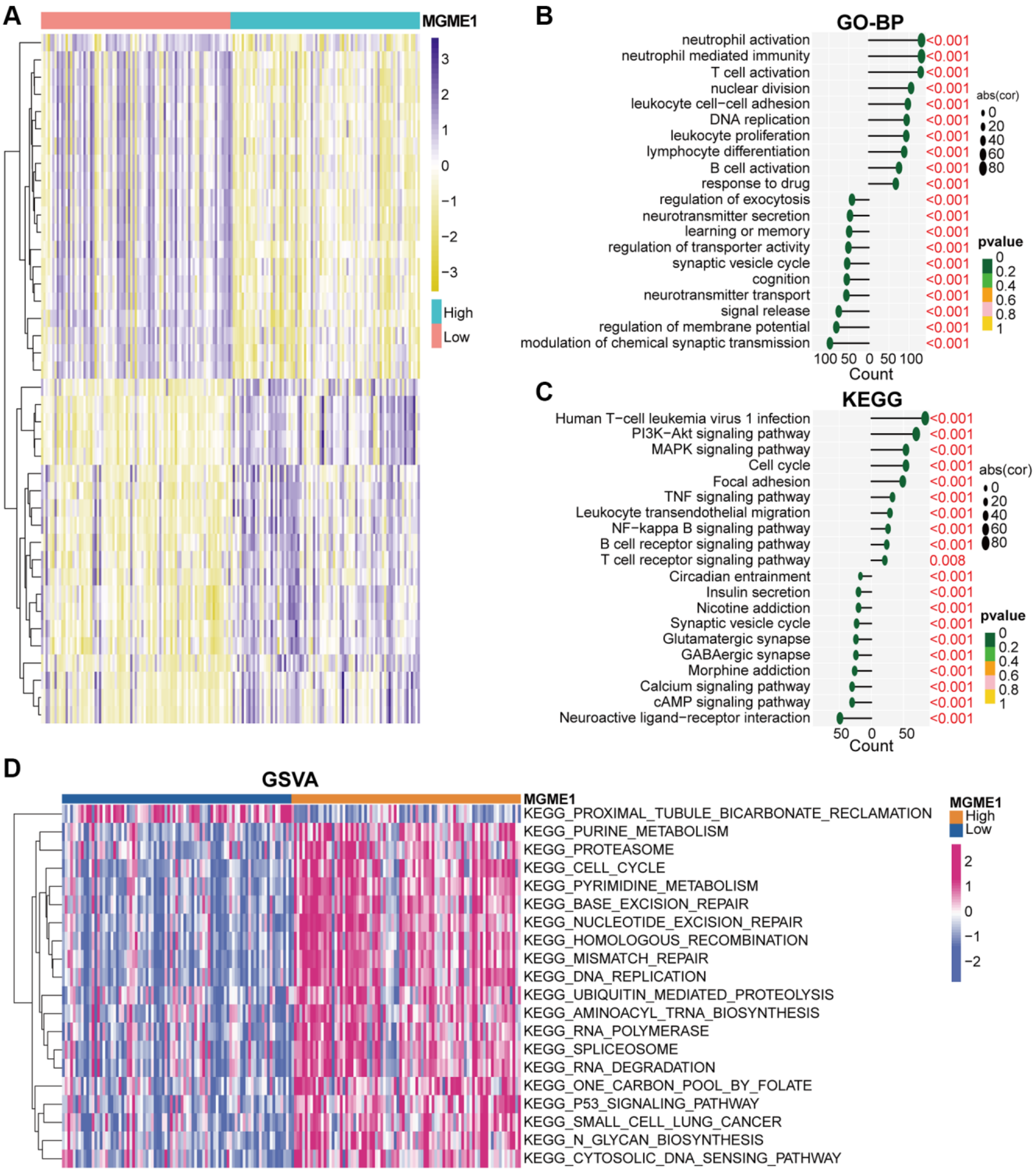

Supplementary Figure 4. Biological functions of MGME1 in LGG in CGGA. (**A**) DEGs in the low-MGME1 and high-MGME1 expression subgroups. (**B**, **C**) The GO-BP (**B**) and KEGG (**C**) analyses of MGME1 in patients with LGG patients in the CGGA dataset. (**D**) GSVA of the CGGA data.

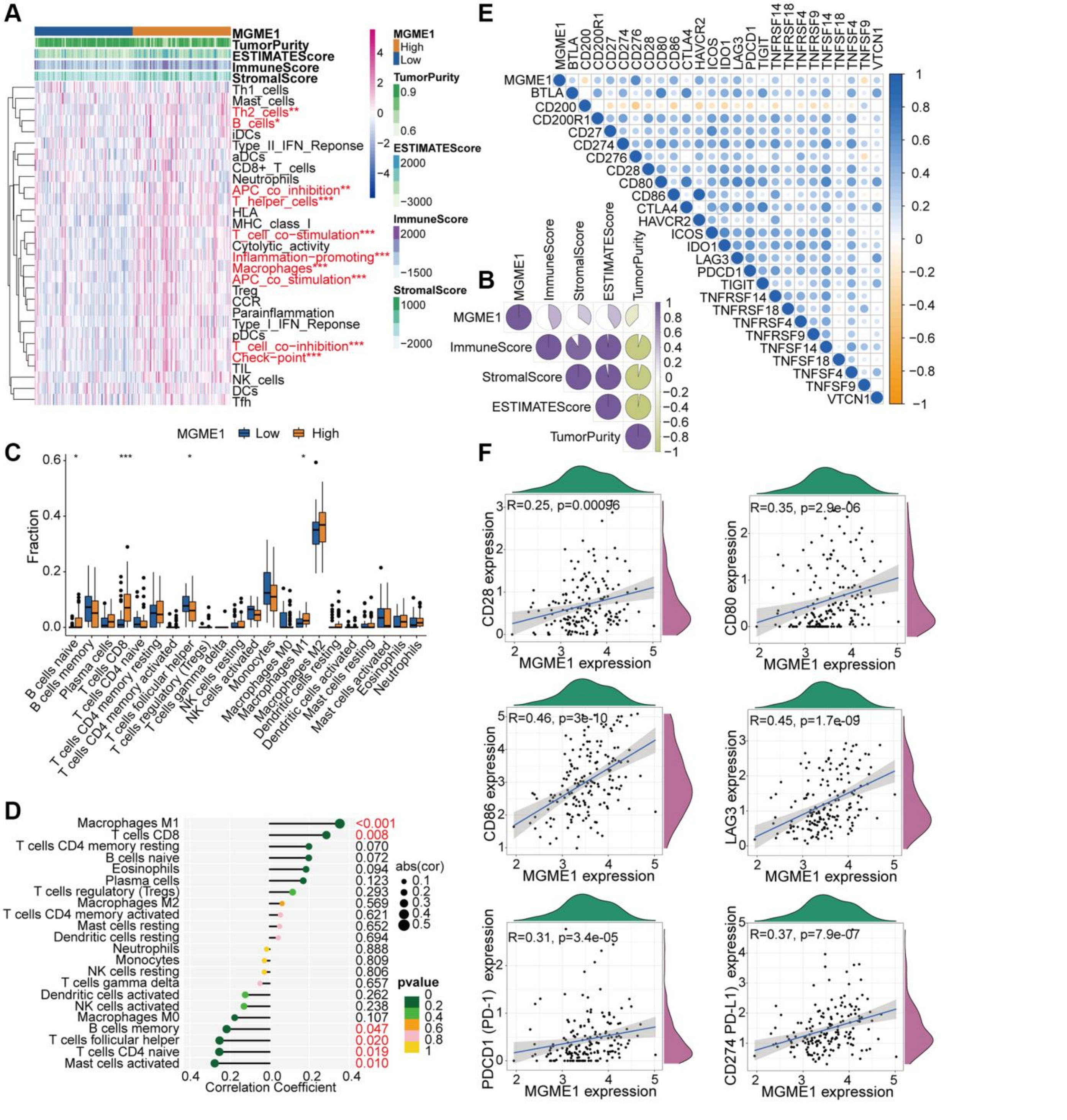

Supplementary Figure 5. Different TME and immunological characteristics of the low-MGME1 and high-MGME1 subtypes in CGGA. (**A**, **B**) Associations between MGME1 expression and 29 immune-associated signatures, ESTIMATE scores, immune scores, stromal scores, and tumor purity. (**C**) Comparisons of infiltration of 22 types of immune cells in the two subgroups. (**D**) Lollipop plots show the relationships between MGME1 expression and TIICs. (**E**, **F**) Co-expression analysis of MGME1 and 25 ICPGs. $^{*}P < 0.05$, $^{**}P < 0.01$, $^{***}P < 0.001$.
