# Supplementary material for: MGME1 associates with poor prognosis and is vital for cell proliferation in lower-grade glioma
Source: Aging (Albany NY). 2023 May 8;15(9):3690–714. doi: 10.18632/aging.204705 (PMC10449294; doi:10.18632/aging.204705)
Supplement: Supplementary Table 1 [file aging-15-204705-s002.docx]

**Supplementary Table 1. Down-regulated DEGs in TCGA dataset.**

| **id** | **logFC** | **AveExpr** | **t** | **P.Value** | **adj.P.Val** | **B** |
| --- | --- | --- | --- | --- | --- | --- |
| CPLX2 | -1.4881 | 5.510399 | -8.2658 | 1.39E-15 | 1.92E-14 | 24.5709 |
| SNCB | -1.4878 | 5.719118 | -8.6181 | 1.01E-16 | 1.59E-15 | 27.1487 |
| GRIN1 | -1.4524 | 4.335483 | -8.1491 | 3.27E-15 | 4.33E-14 | 23.7351 |
| AL022313.4 | -1.4361 | 3.945487 | -9.0509 | 3.64E-18 | 6.74E-17 | 30.4205 |
| CAMK2A | -1.4019 | 4.698479 | -6.5911 | 1.16E-10 | 9.36E-10 | 13.4749 |
| SFRP2 | -1.378 | 5.963052 | -5.99 | 4.14E-09 | 2.75E-08 | 9.99599 |
| VSNL1 | -1.3662 | 4.581628 | -6.2932 | 7.06E-10 | 5.17E-09 | 11.7158 |
| GABRD | -1.3525 | 4.798308 | -9.5381 | 7.56E-20 | 1.72E-18 | 34.2381 |
| CHGA | -1.3444 | 5.197903 | -7.8986 | 1.97E-14 | 2.41E-13 | 21.9708 |
| NSG2 | -1.3314 | 6.766044 | -8.7034 | 5.31E-17 | 8.64E-16 | 27.784 |
| PACSIN1 | -1.3255 | 4.143944 | -6.6137 | 1.01E-10 | 8.20E-10 | 13.6111 |
| SCRT1 | -1.3243 | 3.902986 | -9.1574 | 1.58E-18 | 3.04E-17 | 31.2435 |
| INA | -1.3197 | 5.606853 | -7.8133 | 3.60E-14 | 4.28E-13 | 21.3803 |
| CCK | -1.2989 | 3.519326 | -7.081 | 5.17E-12 | 4.86E-11 | 16.5137 |
| NRGN | -1.2949 | 6.868153 | -6.0067 | 3.76E-09 | 2.51E-08 | 10.0887 |
| SLC17A7 | -1.2877 | 4.603526 | -5.7442 | 1.65E-08 | 1.01E-07 | 8.6556 |
| PRLHR | -1.2862 | 2.761247 | -7.7858 | 4.36E-14 | 5.14E-13 | 21.1908 |
| AC062021.1 | -1.2761 | 3.592077 | -6.4058 | 3.60E-10 | 2.73E-09 | 12.3724 |
| NEFM | -1.2758 | 3.430654 | -6.4257 | 3.19E-10 | 2.43E-09 | 12.4898 |
| SVOP | -1.2728 | 3.244809 | -8.2611 | 1.44E-15 | 1.98E-14 | 24.537 |
| SYT4 | -1.2624 | 3.556194 | -7.3769 | 7.26E-13 | 7.47E-12 | 18.4344 |
| KCNIP2 | -1.2601 | 5.166371 | -9.406 | 2.19E-19 | 4.68E-18 | 33.1894 |
| DDN | -1.2558 | 3.825831 | -6.9747 | 1.03E-11 | 9.35E-11 | 15.8394 |
| SNAP25 | -1.2536 | 7.091278 | -6.8589 | 2.17E-11 | 1.90E-10 | 15.114 |
| GABRG2 | -1.2472 | 3.597076 | -7.0916 | 4.83E-12 | 4.55E-11 | 16.5813 |
| HRH3 | -1.2333 | 3.02902 | -9.1355 | 1.88E-18 | 3.57E-17 | 31.0737 |
| NEFL | -1.2288 | 4.277055 | -5.4665 | 7.42E-08 | 4.22E-07 | 7.20022 |
| L1CAM | -1.2117 | 4.246966 | -6.6598 | 7.58E-11 | 6.24E-10 | 13.8903 |
| TMEM130 | -1.1883 | 4.29666 | -6.886 | 1.82E-11 | 1.61E-10 | 15.2825 |
| GJB6 | -1.177 | 2.453792 | -6.6863 | 6.43E-11 | 5.33E-10 | 14.0511 |
| PHYHIP | -1.1736 | 5.263286 | -7.6085 | 1.50E-13 | 1.67E-12 | 19.981 |
| CACNG3 | -1.1642 | 2.441276 | -7.1403 | 3.51E-12 | 3.36E-11 | 16.8937 |
| SNCG | -1.16 | 5.251109 | -8.2882 | 1.18E-15 | 1.64E-14 | 24.733 |
| PVALB | -1.1587 | 1.929257 | -7.6937 | 8.31E-14 | 9.48E-13 | 20.5593 |
| CALY | -1.1515 | 3.121867 | -7.2891 | 1.31E-12 | 1.31E-11 | 17.8581 |
| SULT4A1 | -1.1508 | 4.083634 | -6.0719 | 2.59E-09 | 1.76E-08 | 10.4532 |
| FAM163B | -1.1391 | 4.506696 | -6.7808 | 3.55E-11 | 3.03E-10 | 14.63 |
| TMEM151B | -1.1363 | 4.357 | -8.7955 | 2.63E-17 | 4.43E-16 | 28.4756 |
| ACTL6B | -1.1319 | 4.580837 | -7.9619 | 1.26E-14 | 1.57E-13 | 22.4129 |
| CDK5R2 | -1.1263 | 4.292846 | -7.9655 | 1.22E-14 | 1.53E-13 | 22.4378 |
| SYN1 | -1.1246 | 6.005985 | -7.8326 | 3.14E-14 | 3.76E-13 | 21.5132 |
| ETNPPL | -1.1199 | 6.574444 | -6.633 | 8.96E-11 | 7.31E-10 | 13.7274 |
| SYT13 | -1.1193 | 3.281219 | -6.7968 | 3.21E-11 | 2.75E-10 | 14.7291 |
| CACNG2 | -1.1043 | 2.731106 | -7.7864 | 4.35E-14 | 5.12E-13 | 21.1946 |
| AL049749.1 | -1.1033 | 2.568528 | -8.2542 | 1.52E-15 | 2.08E-14 | 24.488 |
| HPCAL4 | -1.1011 | 5.135069 | -7.6515 | 1.11E-13 | 1.25E-12 | 20.2726 |
| CHGB | -1.1007 | 5.868205 | -7.5032 | 3.09E-13 | 3.33E-12 | 19.2731 |
| CABP1 | -1.0949 | 3.017664 | -9.2293 | 8.95E-19 | 1.77E-17 | 31.8027 |
| RTP5 | -1.0913 | 4.694043 | -7.4199 | 5.43E-13 | 5.68E-12 | 18.7188 |
| TESPA1 | -1.0894 | 1.629096 | -6.6803 | 6.68E-11 | 5.52E-10 | 14.0149 |
| SLC12A5 | -1.0891 | 3.235115 | -7.3923 | 6.54E-13 | 6.76E-12 | 18.5363 |
| LHX5-AS1 | -1.0887 | 2.293097 | -5.8684 | 8.25E-09 | 5.28E-08 | 9.32693 |
| AC122707.1 | -1.0834 | 2.282884 | -8.7379 | 4.08E-17 | 6.74E-16 | 28.0428 |
| GABRA1 | -1.0766 | 2.769563 | -6.0746 | 2.55E-09 | 1.74E-08 | 10.4686 |
| PRKCG | -1.0714 | 2.967166 | -6.6355 | 8.82E-11 | 7.20E-10 | 13.7428 |
| ARHGDIG | -1.0649 | 4.508013 | -9.0486 | 3.71E-18 | 6.86E-17 | 30.4028 |
| CHRM1 | -1.0567 | 3.404036 | -7.3126 | 1.12E-12 | 1.13E-11 | 18.0115 |
| BCYRN1 | -1.0542 | 2.318353 | -9.3174 | 4.45E-19 | 9.10E-18 | 32.4921 |
| CPLX1 | -1.0522 | 5.393202 | -8.4593 | 3.33E-16 | 4.93E-15 | 25.9769 |
| HPSE2 | -1.0468 | 3.5224 | -6.6195 | 9.75E-11 | 7.92E-10 | 13.6457 |
| IQSEC3 | -1.0416 | 3.084101 | -7.4722 | 3.81E-13 | 4.06E-12 | 19.0665 |
| SLC6A17 | -1.041 | 3.300621 | -6.2628 | 8.46E-10 | 6.14E-09 | 11.5398 |
| FXYD7 | -1.0398 | 3.984488 | -6.4556 | 2.66E-10 | 2.05E-09 | 12.6663 |
| PSD | -1.0343 | 5.445245 | -9.0832 | 2.83E-18 | 5.30E-17 | 30.6693 |
| VSTM2A | -1.0225 | 3.376336 | -6.7325 | 4.82E-11 | 4.05E-10 | 14.3335 |
| PPP1R1A | -1.0202 | 3.88481 | -9.452 | 1.51E-19 | 3.30E-18 | 33.5537 |
| SYT1 | -1.0092 | 4.895078 | -5.227 | 2.58E-07 | 1.37E-06 | 5.99594 |
| JPH3 | -1.0088 | 4.811673 | -8.3958 | 5.35E-16 | 7.70E-15 | 25.5127 |
| HPCA | -1.0081 | 4.4652 | -5.2414 | 2.40E-07 | 1.28E-06 | 6.06705 |
| TMEM271 | -1.0002 | 3.3249 | -8.2176 | 1.98E-15 | 2.69E-14 | 24.225 |
| F5 | -0.9956 | 2.544825 | -6.0372 | 3.16E-09 | 2.13E-08 | 10.2587 |
| CELF4 | -0.992 | 3.514282 | -7.0588 | 5.97E-12 | 5.59E-11 | 16.3723 |
| ABCC8 | -0.9916 | 3.759673 | -7.718 | 7.01E-14 | 8.08E-13 | 20.7256 |
| MPPED1 | -0.9913 | 2.108876 | -6.971 | 1.06E-11 | 9.57E-11 | 15.8159 |
| SLC8A2 | -0.9861 | 3.668358 | -7.7266 | 6.60E-14 | 7.64E-13 | 20.7843 |
| AMER3 | -0.985 | 2.358925 | -8.0979 | 4.73E-15 | 6.17E-14 | 23.3715 |
| MARCHF4 | -0.9848 | 2.683854 | -6.6054 | 1.06E-10 | 8.60E-10 | 13.5612 |
| CRTAC1 | -0.9786 | 5.545226 | -7.4662 | 3.97E-13 | 4.22E-12 | 19.026 |
| MAL2 | -0.9772 | 2.210172 | -6.4421 | 2.89E-10 | 2.22E-09 | 12.5866 |
| MYT1L | -0.977 | 2.378503 | -6.8914 | 1.76E-11 | 1.56E-10 | 15.3167 |
| GNG3 | -0.9755 | 5.157287 | -5.1685 | 3.48E-07 | 1.82E-06 | 5.70919 |
| ATP2B3 | -0.9729 | 2.256356 | -7.5495 | 2.25E-13 | 2.46E-12 | 19.5835 |
| SST | -0.9702 | 4.30203 | -5.6994 | 2.11E-08 | 1.28E-07 | 8.41647 |
| PTPRN | -0.9644 | 4.30229 | -6.0581 | 2.80E-09 | 1.90E-08 | 10.3757 |
| STMN2 | -0.9641 | 5.948608 | -4.6781 | 3.78E-06 | 1.71E-05 | 3.41917 |
| CRYM | -0.9622 | 2.825982 | -5.5667 | 4.34E-08 | 2.54E-07 | 7.71795 |
| CPNE6 | -0.9616 | 2.798883 | -6.4322 | 3.07E-10 | 2.35E-09 | 12.5279 |
| CACNA2D3 | -0.9592 | 2.592313 | -9.4918 | 1.10E-19 | 2.44E-18 | 33.8697 |
| SYNPR | -0.9573 | 2.66604 | -6.0037 | 3.83E-09 | 2.56E-08 | 10.072 |
| SV2B | -0.9547 | 2.463699 | -5.8849 | 7.52E-09 | 4.85E-08 | 9.41696 |
| TRIM67 | -0.954 | 2.869696 | -5.0703 | 5.70E-07 | 2.90E-06 | 5.23414 |
| RBFOX3 | -0.9507 | 2.132831 | -7.2583 | 1.61E-12 | 1.60E-11 | 17.6567 |
| CRLF1 | -0.945 | 4.353198 | -5.4668 | 7.41E-08 | 4.21E-07 | 7.2014 |
| BRINP1 | -0.9449 | 5.027764 | -7.6111 | 1.47E-13 | 1.64E-12 | 19.9986 |
| GABRG1 | -0.9443 | 3.624659 | -6.8887 | 1.79E-11 | 1.58E-10 | 15.2998 |
| WSCD2 | -0.9431 | 2.092049 | -7.9119 | 1.79E-14 | 2.20E-13 | 22.0638 |
| SLC6A7 | -0.9423 | 1.489402 | -6.9454 | 1.25E-11 | 1.12E-10 | 15.6545 |
| HAR1A | -0.9387 | 2.090529 | -9.9548 | 2.47E-21 | 6.80E-20 | 37.6103 |
| SYNGR3 | -0.9365 | 3.590571 | -6.5811 | 1.24E-10 | 9.93E-10 | 13.4144 |
| NEUROD6 | -0.9346 | 1.396378 | -6.8598 | 2.15E-11 | 1.89E-10 | 15.1192 |
| KCNC2 | -0.9335 | 2.010976 | -6.7599 | 4.05E-11 | 3.43E-10 | 14.5016 |
| CLEC2L | -0.9328 | 2.132832 | -7.0341 | 7.02E-12 | 6.51E-11 | 16.215 |
| RAB3A | -0.9315 | 5.917121 | -7.3748 | 7.37E-13 | 7.57E-12 | 18.4202 |
| CREG2 | -0.9274 | 2.837636 | -5.3022 | 1.76E-07 | 9.50E-07 | 6.36866 |
| SYN2 | -0.9241 | 4.598127 | -6.0277 | 3.34E-09 | 2.25E-08 | 10.206 |
| CAMKV | -0.9234 | 4.158688 | -6.3641 | 4.63E-10 | 3.47E-09 | 12.1279 |
| CAMK1G | -0.9179 | 2.540346 | -5.451 | 8.06E-08 | 4.55E-07 | 7.12053 |
| SCN3B | -0.9154 | 4.452063 | -6.8106 | 2.94E-11 | 2.53E-10 | 14.814 |
| CORO6 | -0.9136 | 2.440833 | -8.3953 | 5.37E-16 | 7.73E-15 | 25.5091 |
| LCNL1 | -0.9112 | 3.173715 | -6.9769 | 1.02E-11 | 9.24E-11 | 15.853 |
| SELL | -0.9106 | 5.527587 | -4.7453 | 2.76E-06 | 1.27E-05 | 3.72079 |
| RGS4 | -0.9081 | 3.56924 | -4.9237 | 1.17E-06 | 5.70E-06 | 4.54016 |
| NRSN1 | -0.9078 | 4.627005 | -7.3613 | 8.07E-13 | 8.25E-12 | 18.3317 |
| CCKBR | -0.9076 | 1.657781 | -7.5417 | 2.37E-13 | 2.58E-12 | 19.5313 |
| RASL10A | -0.9071 | 4.974262 | -7.2465 | 1.74E-12 | 1.72E-11 | 17.5802 |
| CHD5 | -0.9062 | 3.019613 | -6.4868 | 2.20E-10 | 1.71E-09 | 12.8509 |
| SNAP91 | -0.9049 | 5.164439 | -7.6803 | 9.11E-14 | 1.04E-12 | 20.4686 |
| NPTX1 | -0.8953 | 4.03567 | -5.2496 | 2.30E-07 | 1.23E-06 | 6.10761 |
| NEURL1 | -0.8934 | 2.831514 | -6.7458 | 4.43E-11 | 3.74E-10 | 14.4148 |
| KCNJ11 | -0.8914 | 4.173143 | -6.8261 | 2.67E-11 | 2.31E-10 | 14.9104 |
| PPP2R2C | -0.8849 | 4.16648 | -6.5246 | 1.75E-10 | 1.38E-09 | 13.0763 |
| MIR7-3HG | -0.883 | 1.896647 | -7.1907 | 2.52E-12 | 2.45E-11 | 17.2183 |
| ASIC4-AS1 | -0.8811 | 4.207012 | -5.6124 | 3.39E-08 | 2.01E-07 | 7.95657 |
| NGB | -0.8795 | 1.480308 | -6.5122 | 1.89E-10 | 1.48E-09 | 13.0022 |
| GABRB2 | -0.8791 | 2.185867 | -5.7441 | 1.65E-08 | 1.02E-07 | 8.65489 |
| GABRA5 | -0.879 | 2.421527 | -5.7216 | 1.87E-08 | 1.14E-07 | 8.53444 |
| SPHKAP | -0.8786 | 3.358695 | -6.1157 | 2.01E-09 | 1.39E-08 | 10.7002 |
| PNCK | -0.8762 | 3.199931 | -9.8971 | 3.99E-21 | 1.07E-19 | 37.1383 |
| PTPN5 | -0.8762 | 2.96642 | -5.8262 | 1.05E-08 | 6.60E-08 | 9.09725 |
| NPM2 | -0.8736 | 2.771898 | -7.3246 | 1.03E-12 | 1.05E-11 | 18.0901 |
| TUBA4A | -0.8734 | 4.600422 | -7.0108 | 8.16E-12 | 7.51E-11 | 16.0672 |
| KCNK3 | -0.8653 | 2.987469 | -6.622 | 9.59E-11 | 7.81E-10 | 13.661 |
| OGDHL | -0.863 | 2.85729 | -6.7711 | 3.78E-11 | 3.21E-10 | 14.5707 |
| CDH22 | -0.8612 | 3.088581 | -7.8176 | 3.49E-14 | 4.16E-13 | 21.4099 |
| C1QTNF4 | -0.8598 | 3.099248 | -8.5376 | 1.86E-16 | 2.83E-15 | 26.5527 |
| RAB3C | -0.8581 | 3.308332 | -6.3795 | 4.22E-10 | 3.18E-09 | 12.2179 |
| RFPL1S | -0.8565 | 2.475126 | -7.0208 | 7.65E-12 | 7.07E-11 | 16.131 |
| RBFOX1 | -0.85 | 2.498488 | -5.8299 | 1.02E-08 | 6.48E-08 | 9.11704 |
| SYT5 | -0.8449 | 3.082962 | -6.3613 | 4.70E-10 | 3.52E-09 | 12.1119 |
| AC125616.1 | -0.8421 | 1.247207 | -9.3365 | 3.82E-19 | 7.91E-18 | 32.6421 |
| AC012213.4 | -0.8408 | 2.484026 | -6.7958 | 3.23E-11 | 2.77E-10 | 14.723 |
| TMEM155 | -0.8387 | 1.942578 | -6.0819 | 2.44E-09 | 1.67E-08 | 10.5093 |
| PNMA6F | -0.8379 | 1.243802 | -7.5835 | 1.78E-13 | 1.96E-12 | 19.8121 |
| WNT7B | -0.8337 | 3.549578 | -6.2044 | 1.19E-09 | 8.48E-09 | 11.2044 |
| SCN2B | -0.833 | 3.805081 | -7.4938 | 3.29E-13 | 3.54E-12 | 19.2105 |
| TCEAL6 | -0.8327 | 3.521157 | -5.7427 | 1.66E-08 | 1.02E-07 | 8.64719 |
| PHF24 | -0.8321 | 3.228791 | -5.3516 | 1.36E-07 | 7.46E-07 | 6.61613 |
| NECAB2 | -0.8298 | 4.621091 | -8.5439 | 1.77E-16 | 2.71E-15 | 26.5994 |
| AC015540.1 | -0.8296 | 3.137744 | -8.6005 | 1.16E-16 | 1.80E-15 | 27.0176 |
| LINC00599 | -0.8282 | 3.13768 | -6.714 | 5.41E-11 | 4.53E-10 | 14.2206 |
| SMIM10L2B | -0.8247 | 3.365413 | -8.0523 | 6.57E-15 | 8.45E-14 | 23.0484 |
| SHISAL1 | -0.8244 | 3.323926 | -5.8653 | 8.40E-09 | 5.37E-08 | 9.30952 |
| SH2D5 | -0.822 | 1.915997 | -6.6892 | 6.32E-11 | 5.25E-10 | 14.0687 |
| SYP | -0.8197 | 6.459307 | -8.4423 | 3.78E-16 | 5.55E-15 | 25.8527 |
| RUNDC3A | -0.8186 | 6.23296 | -8.7116 | 4.99E-17 | 8.15E-16 | 27.8453 |
| ATP6V1G2 | -0.8184 | 7.211446 | -9.1981 | 1.15E-18 | 2.25E-17 | 31.5595 |
| USH1C | -0.8183 | 4.217872 | -4.9545 | 1.01E-06 | 4.95E-06 | 4.6844 |
| SH3GL2 | -0.8172 | 6.436623 | -6.6205 | 9.68E-11 | 7.87E-10 | 13.6521 |
| CNNM1 | -0.8172 | 2.004629 | -7.9611 | 1.26E-14 | 1.58E-13 | 22.4071 |
| NEUROD2 | -0.8163 | 1.706113 | -6.7123 | 5.47E-11 | 4.58E-10 | 14.2097 |
| AC027130.1 | -0.8155 | 2.39269 | -9.3188 | 4.40E-19 | 9.02E-18 | 32.5027 |
| RPH3A | -0.8127 | 3.5838 | -5.0803 | 5.42E-07 | 2.77E-06 | 5.28213 |
| PPP4R4 | -0.8095 | 2.464906 | -7.3294 | 9.99E-13 | 1.01E-11 | 18.1219 |
| KCNJ3 | -0.8095 | 2.588248 | -6.9665 | 1.09E-11 | 9.83E-11 | 15.7877 |
| SRRM4 | -0.8089 | 2.284469 | -6.8731 | 1.98E-11 | 1.74E-10 | 15.2024 |
| SOHLH1 | -0.807 | 1.431313 | -7.2152 | 2.14E-12 | 2.09E-11 | 17.3767 |
| CARTPT | -0.8064 | 0.854107 | -5.988 | 4.19E-09 | 2.78E-08 | 9.98466 |
| KCNJ4 | -0.8059 | 3.381792 | -5.4884 | 6.60E-08 | 3.78E-07 | 7.31265 |
| RBP4 | -0.8053 | 2.338123 | -5.9566 | 5.01E-09 | 3.29E-08 | 9.81113 |
| TMEM196 | -0.8043 | 1.995445 | -6.4144 | 3.42E-10 | 2.60E-09 | 12.4233 |
| FBXL16 | -0.802 | 6.56774 | -7.4708 | 3.85E-13 | 4.10E-12 | 19.0567 |
| HTR1A | -0.8019 | 1.407474 | -7.3387 | 9.39E-13 | 9.56E-12 | 18.1826 |
| ALDOC | -0.7986 | 9.933016 | -8.0488 | 6.74E-15 | 8.66E-14 | 23.024 |
| UNC5A | -0.7958 | 4.407894 | -7.2066 | 2.27E-12 | 2.21E-11 | 17.3209 |
| BEX5 | -0.7951 | 4.358412 | -5.7025 | 2.08E-08 | 1.26E-07 | 8.43275 |
| CBLN2 | -0.7951 | 1.938325 | -7.0596 | 5.94E-12 | 5.56E-11 | 16.3773 |
| SERPINI1 | -0.7944 | 5.383357 | -6.0771 | 2.51E-09 | 1.72E-08 | 10.4822 |
| PCSK2 | -0.7929 | 3.429944 | -5.2796 | 1.97E-07 | 1.06E-06 | 6.25614 |
| SLC30A3 | -0.7918 | 1.700292 | -5.5451 | 4.88E-08 | 2.84E-07 | 7.60559 |
| SSTR2 | -0.7912 | 3.670205 | -6.3237 | 5.89E-10 | 4.36E-09 | 11.8923 |
| DLGAP1-AS4 | -0.791 | 1.591031 | -6.688 | 6.36E-11 | 5.28E-10 | 14.0614 |
| TNNT1 | -0.7908 | 2.183191 | -7.012 | 8.10E-12 | 7.46E-11 | 16.0748 |
| MAP7D2 | -0.7908 | 2.40291 | -5.02 | 7.32E-07 | 3.67E-06 | 4.99372 |
| GDA | -0.7907 | 2.521455 | -4.8961 | 1.34E-06 | 6.46E-06 | 4.41144 |
| GABBR1 | -0.7904 | 7.487837 | -8.6794 | 6.37E-17 | 1.03E-15 | 27.6051 |
| VSTM2L | -0.7894 | 4.452507 | -5.5892 | 3.85E-08 | 2.26E-07 | 7.83519 |
| CAMSAP3 | -0.7893 | 3.374895 | -8.2348 | 1.75E-15 | 2.39E-14 | 24.3481 |
| LINC01007 | -0.7891 | 0.60364 | -7.8336 | 3.12E-14 | 3.73E-13 | 21.5203 |
| MATK | -0.7881 | 2.632876 | -7.7248 | 6.69E-14 | 7.73E-13 | 20.7718 |
| CELF3 | -0.7878 | 4.803138 | -6.3757 | 4.31E-10 | 3.25E-09 | 12.1957 |
| AC026790.2 | -0.7856 | 1.203788 | -10.07 | 9.45E-22 | 2.73E-20 | 38.5589 |
| AK5 | -0.7841 | 3.925127 | -5.236 | 2.47E-07 | 1.31E-06 | 6.0403 |
| STX1B | -0.7819 | 5.416858 | -8.203 | 2.21E-15 | 2.98E-14 | 24.12 |
| SLC32A1 | -0.7808 | 2.459061 | -4.9146 | 1.23E-06 | 5.93E-06 | 4.49785 |
| PDE2A | -0.7804 | 4.808438 | -7.1513 | 3.26E-12 | 3.14E-11 | 16.964 |
| CPNE9 | -0.7799 | 1.576323 | -7.7159 | 7.11E-14 | 8.19E-13 | 20.7111 |
| AC008780.1 | -0.7796 | 1.188654 | -7.1488 | 3.32E-12 | 3.19E-11 | 16.948 |
| CA11 | -0.7784 | 6.678273 | -8.0212 | 8.21E-15 | 1.04E-13 | 22.8291 |
| ICAM5 | -0.7774 | 2.788479 | -5.5714 | 4.23E-08 | 2.48E-07 | 7.7425 |
| SLC25A48 | -0.7769 | 3.621057 | -5.9827 | 4.32E-09 | 2.86E-08 | 9.95525 |
| CAMK2B | -0.7767 | 4.38013 | -6.1207 | 1.95E-09 | 1.35E-08 | 10.7285 |
| TAFA1 | -0.7766 | 2.003405 | -6.2117 | 1.14E-09 | 8.15E-09 | 11.2465 |
| C1QL3 | -0.7752 | 1.719492 | -5.1494 | 3.83E-07 | 1.99E-06 | 5.6158 |
| NAP1L2 | -0.7745 | 5.125218 | -7.6956 | 8.19E-14 | 9.36E-13 | 20.5725 |
| DOC2A | -0.7741 | 2.436234 | -6.326 | 5.81E-10 | 4.30E-09 | 11.9057 |
| SYT7 | -0.7738 | 3.700732 | -5.251 | 2.29E-07 | 1.22E-06 | 6.1143 |
| CALN1 | -0.7697 | 3.665769 | -5.2399 | 2.42E-07 | 1.29E-06 | 6.05961 |
| CSDC2 | -0.7696 | 5.90945 | -5.4606 | 7.66E-08 | 4.34E-07 | 7.16977 |
| RNU6-353P | -0.7696 | 1.797667 | -7.9303 | 1.57E-14 | 1.94E-13 | 22.1923 |
| ST8SIA3 | -0.7661 | 3.410751 | -5.721 | 1.87E-08 | 1.15E-07 | 8.53139 |
| GRIN3A | -0.765 | 2.210003 | -5.9313 | 5.79E-09 | 3.78E-08 | 9.67133 |
| MTND1P23 | -0.7637 | 3.224726 | -5.0538 | 6.19E-07 | 3.13E-06 | 5.1552 |
| GDF10 | -0.7623 | 2.906191 | -5.3076 | 1.71E-07 | 9.26E-07 | 6.39579 |
| KCNH3 | -0.7614 | 3.27266 | -5.8725 | 8.07E-09 | 5.17E-08 | 9.349 |
| HCN1 | -0.7609 | 1.593619 | -5.9352 | 5.66E-09 | 3.70E-08 | 9.69267 |
| C1orf115 | -0.7608 | 4.011284 | -6.0747 | 2.55E-09 | 1.74E-08 | 10.4687 |
| SLC1A2 | -0.7605 | 7.68188 | -6.5058 | 1.96E-10 | 1.54E-09 | 12.9641 |
| ATP8A2 | -0.7595 | 2.273198 | -6.2376 | 9.82E-10 | 7.06E-09 | 11.3949 |
| GABRB3 | -0.7594 | 3.949036 | -6.5317 | 1.67E-10 | 1.32E-09 | 13.1186 |
| CARMIL2 | -0.7593 | 1.692988 | -9.2332 | 8.68E-19 | 1.72E-17 | 31.8334 |
| CHST1 | -0.7573 | 5.465782 | -7.1233 | 3.92E-12 | 3.74E-11 | 16.7845 |
| HTR2A | -0.7573 | 2.301878 | -7.1357 | 3.61E-12 | 3.46E-11 | 16.8638 |
| GOLGA7B | -0.7571 | 3.954704 | -7.2938 | 1.27E-12 | 1.27E-11 | 17.8889 |
| SOWAHA | -0.7549 | 3.09552 | -5.9589 | 4.95E-09 | 3.25E-08 | 9.82373 |
| WNT10B | -0.7518 | 1.631125 | -6.5054 | 1.97E-10 | 1.54E-09 | 12.9617 |
| CKMT1A | -0.7516 | 1.77133 | -9.3186 | 4.40E-19 | 9.02E-18 | 32.5015 |
| AP001972.5 | -0.7514 | 5.443593 | -7.401 | 6.17E-13 | 6.41E-12 | 18.5934 |
| DACH2 | -0.749 | 1.99609 | -6.3945 | 3.85E-10 | 2.91E-09 | 12.306 |
| TAFA2 | -0.7479 | 2.14924 | -7.3008 | 1.21E-12 | 1.22E-11 | 17.9343 |
| ATP1A3 | -0.747 | 7.298628 | -6.9316 | 1.36E-11 | 1.22E-10 | 15.568 |
| PNMA3 | -0.7467 | 3.707093 | -6.994 | 9.10E-12 | 8.33E-11 | 15.9613 |
| RGS7 | -0.7467 | 3.731095 | -6.5241 | 1.75E-10 | 1.38E-09 | 13.0734 |
| EPHB6 | -0.7452 | 3.517703 | -5.1958 | 3.03E-07 | 1.59E-06 | 5.84242 |
| KRT222 | -0.745 | 1.166459 | -8.1311 | 3.72E-15 | 4.91E-14 | 23.6068 |
| CKMT1B | -0.7443 | 2.055631 | -8.6887 | 5.94E-17 | 9.59E-16 | 27.674 |
| SGSM1 | -0.7438 | 3.123457 | -8.5935 | 1.22E-16 | 1.90E-15 | 26.9659 |
| CEND1 | -0.7425 | 7.055789 | -8.306 | 1.04E-15 | 1.45E-14 | 24.8615 |
| DGKK | -0.7418 | 1.360365 | -6.9327 | 1.35E-11 | 1.21E-10 | 15.5747 |
| SLC26A4-AS1 | -0.7414 | 1.574125 | -5.6984 | 2.12E-08 | 1.29E-07 | 8.4113 |
| LINC00507 | -0.7413 | 0.820757 | -6.3962 | 3.81E-10 | 2.88E-09 | 12.3159 |
| CUX2 | -0.7411 | 2.724105 | -5.7397 | 1.69E-08 | 1.04E-07 | 8.63155 |
| NGEF | -0.7404 | 4.162487 | -4.9898 | 8.49E-07 | 4.22E-06 | 4.85076 |
| JAKMIP1 | -0.7404 | 3.428747 | -6.5134 | 1.87E-10 | 1.47E-09 | 13.0092 |
| ZFR2 | -0.7401 | 1.770225 | -6.1432 | 1.71E-09 | 1.19E-08 | 10.8558 |
| KCNT1 | -0.7394 | 1.565124 | -7.246 | 1.74E-12 | 1.72E-11 | 17.5768 |
| SLC4A10 | -0.7392 | 2.839273 | -5.4104 | 9.98E-08 | 5.59E-07 | 6.91387 |
| KLK7 | -0.7385 | 1.000682 | -7.1552 | 3.18E-12 | 3.06E-11 | 16.9896 |
| AC104072.1 | -0.7363 | 2.699444 | -4.7164 | 3.16E-06 | 1.44E-05 | 3.59066 |
| TUNAR | -0.7362 | 1.751784 | -6.2838 | 7.47E-10 | 5.45E-09 | 11.6613 |
| NEFH | -0.7361 | 3.291804 | -4.9514 | 1.03E-06 | 5.02E-06 | 4.66975 |
| NMNAT2 | -0.7351 | 4.955723 | -6.3503 | 5.02E-10 | 3.74E-09 | 12.0475 |
| FAIM2 | -0.735 | 7.615607 | -9.1369 | 1.86E-18 | 3.54E-17 | 31.0845 |
| KSR2 | -0.7341 | 1.811504 | -8.1555 | 3.12E-15 | 4.14E-14 | 23.7804 |
| MT-TF | -0.7316 | 4.783588 | -5.1572 | 3.68E-07 | 1.92E-06 | 5.65392 |
| SNCA | -0.7312 | 4.426722 | -5.6126 | 3.39E-08 | 2.01E-07 | 7.95778 |
| CA10 | -0.7296 | 4.567347 | -4.9955 | 8.26E-07 | 4.11E-06 | 4.8776 |
| LY6H | -0.7291 | 5.11528 | -5.3154 | 1.64E-07 | 8.91E-07 | 6.43453 |
| CLVS2 | -0.7282 | 2.480464 | -5.8823 | 7.63E-09 | 4.91E-08 | 9.4027 |
| AL354863.1 | -0.7282 | 1.208993 | -8.5162 | 2.18E-16 | 3.29E-15 | 26.3948 |
| KCNS1 | -0.7271 | 1.477588 | -5.057 | 6.09E-07 | 3.09E-06 | 5.17019 |
| GDAP1L1 | -0.727 | 5.399706 | -6.2938 | 7.04E-10 | 5.16E-09 | 11.7189 |
| PRKCB | -0.7267 | 4.281533 | -6.0006 | 3.90E-09 | 2.60E-08 | 10.0548 |
| WIF1 | -0.7259 | 1.783799 | -4.328 | 1.84E-05 | 7.51E-05 | 1.91196 |
| FBXO2 | -0.7237 | 5.965213 | -5.9856 | 4.25E-09 | 2.82E-08 | 9.97145 |
| ADARB2 | -0.7224 | 3.123375 | -6.4799 | 2.30E-10 | 1.78E-09 | 12.8098 |
| GAD2 | -0.722 | 2.27765 | -4.7555 | 2.63E-06 | 1.21E-05 | 3.76692 |
| RTN4R | -0.7216 | 3.452576 | -7.1476 | 3.34E-12 | 3.21E-11 | 16.9405 |
| HTR5A | -0.7199 | 1.175706 | -6.9542 | 1.18E-11 | 1.06E-10 | 15.7101 |
| NTSR2 | -0.7192 | 5.281338 | -4.2397 | 2.69E-05 | 0.000107 | 1.54859 |
| VIP | -0.7186 | 1.728085 | -6.0732 | 2.57E-09 | 1.75E-08 | 10.4608 |
| NELL1 | -0.7181 | 1.818934 | -5.8455 | 9.38E-09 | 5.96E-08 | 9.20222 |
| ITPKA | -0.7177 | 3.509434 | -6.6206 | 9.68E-11 | 7.87E-10 | 13.6524 |
| JPH4 | -0.7154 | 6.03188 | -6.7704 | 3.79E-11 | 3.22E-10 | 14.5661 |
| ENHO | -0.7142 | 8.558421 | -7.418 | 5.50E-13 | 5.74E-12 | 18.7061 |
| ARHGAP44 | -0.7138 | 2.518052 | -7.811 | 3.66E-14 | 4.34E-13 | 21.3638 |
| RTN1 | -0.7135 | 7.330221 | -7.3399 | 9.32E-13 | 9.48E-12 | 18.1906 |
| HIPK4 | -0.7123 | 1.174355 | -6.6797 | 6.70E-11 | 5.54E-10 | 14.0109 |
| CAMK4 | -0.7088 | 2.342546 | -5.8035 | 1.19E-08 | 7.44E-08 | 8.97436 |
| PTPRT | -0.7084 | 3.607547 | -5.6647 | 2.55E-08 | 1.53E-07 | 8.23252 |
| STX1A | -0.7084 | 4.206629 | -5.5397 | 5.02E-08 | 2.91E-07 | 7.57764 |
| SSTR1 | -0.708 | 3.119609 | -5.0525 | 6.23E-07 | 3.15E-06 | 5.14874 |
| KCNJ9 | -0.7068 | 5.195121 | -6.9702 | 1.06E-11 | 9.61E-11 | 15.8106 |
| GLT1D1 | -0.7062 | 2.190503 | -5.7047 | 2.05E-08 | 1.25E-07 | 8.44478 |
| LINC02607 | -0.7052 | 1.080101 | -6.6474 | 8.20E-11 | 6.72E-10 | 13.8147 |
| SLC24A4 | -0.7038 | 2.562702 | -6.1828 | 1.36E-09 | 9.57E-09 | 11.0811 |
| LINC00634 | -0.7034 | 5.769415 | -9.2499 | 7.60E-19 | 1.52E-17 | 31.9637 |
| TBR1 | -0.7029 | 1.408239 | -6.258 | 8.71E-10 | 6.30E-09 | 11.5122 |
| TMEM132D | -0.7025 | 1.556769 | -5.6419 | 2.89E-08 | 1.73E-07 | 8.11212 |
| RASGRF1 | -0.7021 | 3.341665 | -5.847 | 9.31E-09 | 5.92E-08 | 9.21004 |
| RIMS2 | -0.7018 | 2.458794 | -6.2984 | 6.85E-10 | 5.03E-09 | 11.7455 |
| SHANK1 | -0.7017 | 3.177583 | -5.8486 | 9.22E-09 | 5.87E-08 | 9.21905 |
| GALNT17 | -0.7012 | 3.663615 | -4.6444 | 4.42E-06 | 1.98E-05 | 3.26929 |
| OLFM3 | -0.7002 | 1.564697 | -5.775 | 1.39E-08 | 8.64E-08 | 8.82075 |
| GPR27 | -0.6997 | 4.380128 | -5.8951 | 7.10E-09 | 4.59E-08 | 9.4728 |
| DLGAP3 | -0.6995 | 3.573106 | -6.4804 | 2.29E-10 | 1.78E-09 | 12.8128 |
| XKR7 | -0.6966 | 1.92879 | -5.5806 | 4.03E-08 | 2.37E-07 | 7.79051 |
| MTCO3P12 | -0.6957 | 4.128044 | -4.8085 | 2.04E-06 | 9.59E-06 | 4.00776 |
| TPD52L1 | -0.695 | 3.220105 | -7.2608 | 1.58E-12 | 1.57E-11 | 17.6732 |
| PRSS3 | -0.6934 | 2.272941 | -6.3427 | 5.26E-10 | 3.91E-09 | 12.0029 |
| PTGDS | -0.6932 | 9.226872 | -5.6754 | 2.41E-08 | 1.45E-07 | 8.28919 |
| CBLN1 | -0.6926 | 3.10417 | -5.9803 | 4.38E-09 | 2.90E-08 | 9.94204 |
| KCNV1 | -0.6924 | 1.097309 | -5.8661 | 8.36E-09 | 5.35E-08 | 9.31419 |
| DMTN | -0.692 | 4.948916 | -5.2929 | 1.84E-07 | 9.95E-07 | 6.32243 |
| AC109439.1 | -0.6914 | 2.178321 | -5.279 | 1.98E-07 | 1.07E-06 | 6.25309 |
| ASIC2 | -0.6901 | 1.61334 | -7.0853 | 5.03E-12 | 4.74E-11 | 16.5413 |
| KCNN1 | -0.689 | 3.713385 | -8.0757 | 5.55E-15 | 7.20E-14 | 23.2136 |
| GAD1 | -0.6887 | 4.401707 | -5.8626 | 8.53E-09 | 5.44E-08 | 9.29483 |
| LINC02716 | -0.6876 | 2.848597 | -8.3398 | 8.09E-16 | 1.14E-14 | 25.1058 |
| AC021613.1 | -0.687 | 0.936789 | -7.0818 | 5.14E-12 | 4.84E-11 | 16.5189 |
| HLF | -0.6865 | 4.473885 | -9.1811 | 1.31E-18 | 2.55E-17 | 31.4271 |
| SLC7A14 | -0.686 | 3.494935 | -5.8303 | 1.02E-08 | 6.46E-08 | 9.11929 |
| AP000843.1 | -0.6853 | 1.490109 | -7.4203 | 5.42E-13 | 5.66E-12 | 18.7215 |
| MT-TE | -0.6848 | 1.447388 | -7.2827 | 1.37E-12 | 1.37E-11 | 17.8163 |
| CACNA1I | -0.6848 | 1.651502 | -7.2641 | 1.55E-12 | 1.54E-11 | 17.6945 |
| TSPYL2 | -0.6824 | 6.234965 | -8.5409 | 1.81E-16 | 2.76E-15 | 26.5769 |
| ASIC4 | -0.6816 | 5.445433 | -4.4803 | 9.34E-06 | 3.99E-05 | 2.55449 |
| AL139246.5 | -0.6808 | 1.95187 | -5.9781 | 4.43E-09 | 2.93E-08 | 9.92997 |
| VWC2L | -0.6802 | 1.591238 | -6.3262 | 5.80E-10 | 4.29E-09 | 11.9073 |
| TAGLN3 | -0.6787 | 6.557168 | -5.8543 | 8.94E-09 | 5.69E-08 | 9.24961 |
| SEZ6L2 | -0.6771 | 6.176715 | -5.2569 | 2.22E-07 | 1.19E-06 | 6.14339 |
| AL355916.2 | -0.6764 | 3.802927 | -4.1022 | 4.81E-05 | 0.000185 | 0.99723 |
| UBE2QL1 | -0.6763 | 4.031871 | -6.3494 | 5.05E-10 | 3.76E-09 | 12.0421 |
| C11orf87 | -0.6761 | 1.816271 | -4.7768 | 2.38E-06 | 1.11E-05 | 3.86344 |
| PRRT2 | -0.6738 | 4.94187 | -7.5654 | 2.01E-13 | 2.21E-12 | 19.6908 |
| LRTM2 | -0.6731 | 2.217729 | -5.1937 | 3.06E-07 | 1.61E-06 | 5.83202 |
| PNMA5 | -0.673 | 1.397594 | -7.1271 | 3.82E-12 | 3.65E-11 | 16.8085 |
| UNC13C | -0.6725 | 1.303108 | -6.4591 | 2.61E-10 | 2.01E-09 | 12.687 |
| ADAM11 | -0.6721 | 3.097357 | -6.0314 | 3.27E-09 | 2.20E-08 | 10.2265 |
| AIFM3 | -0.6719 | 2.935457 | -5.1712 | 3.43E-07 | 1.79E-06 | 5.72209 |
| PANX2 | -0.6714 | 3.257257 | -7.4186 | 5.48E-13 | 5.72E-12 | 18.7101 |
| CASKIN1 | -0.6712 | 4.322227 | -7.8891 | 2.11E-14 | 2.57E-13 | 21.9047 |
| MT-RNR1 | -0.6709 | 12.94464 | -9.9935 | 1.79E-21 | 5.01E-20 | 37.9286 |
| CALB2 | -0.6705 | 2.996003 | -4.4737 | 9.63E-06 | 4.10E-05 | 2.52601 |
| TTC9B | -0.6704 | 4.095373 | -6.5169 | 1.83E-10 | 1.44E-09 | 13.0305 |
| CHRM4 | -0.6699 | 2.46358 | -6.7955 | 3.24E-11 | 2.77E-10 | 14.7209 |
| MAPK8IP2 | -0.6699 | 5.795825 | -8.2716 | 1.34E-15 | 1.84E-14 | 24.6127 |
| KNDC1 | -0.6681 | 4.217204 | -7.0229 | 7.55E-12 | 6.98E-11 | 16.1437 |
| PDE1A | -0.6681 | 2.760063 | -6.3429 | 5.25E-10 | 3.90E-09 | 12.0042 |
| CPNE7 | -0.6674 | 1.758589 | -6.2315 | 1.02E-09 | 7.31E-09 | 11.36 |
| LINC00836 | -0.667 | 3.282366 | -4.7737 | 2.41E-06 | 1.12E-05 | 3.84926 |
| CTXN3 | -0.6664 | 0.790632 | -6.3046 | 6.60E-10 | 4.85E-09 | 11.7816 |
| LHX5 | -0.6663 | 1.574881 | -4.7018 | 3.38E-06 | 1.54E-05 | 3.52486 |
| FRMPD4 | -0.6661 | 1.506096 | -6.1089 | 2.09E-09 | 1.44E-08 | 10.6618 |
| SLC22A6 | -0.6651 | 2.509707 | -6.0291 | 3.31E-09 | 2.23E-08 | 10.2138 |
| ACBD7 | -0.6644 | 4.429142 | -4.9422 | 1.07E-06 | 5.23E-06 | 4.62667 |
| DUSP26 | -0.6641 | 6.175891 | -7.479 | 3.64E-13 | 3.89E-12 | 19.1115 |
| RTN4RL1 | -0.6627 | 2.009409 | -5.3834 | 1.15E-07 | 6.38E-07 | 6.77699 |
| GRIN2C | -0.6615 | 3.088455 | -7.4569 | 4.23E-13 | 4.48E-12 | 18.9646 |
| PNMA8B | -0.6615 | 4.066198 | -8.1635 | 2.94E-15 | 3.92E-14 | 23.8381 |
| GALNT9 | -0.6613 | 3.145149 | -5.3657 | 1.26E-07 | 6.96E-07 | 6.68726 |
| AL365205.4 | -0.6602 | 1.584139 | -8.4012 | 5.14E-16 | 7.41E-15 | 25.5525 |
| HAR1B | -0.6594 | 1.397161 | -8.4128 | 4.71E-16 | 6.84E-15 | 25.637 |
| NAPB | -0.6587 | 5.398428 | -5.5785 | 4.08E-08 | 2.39E-07 | 7.77963 |
| AC005696.4 | -0.6577 | 4.122001 | -6.114 | 2.03E-09 | 1.40E-08 | 10.6903 |
| RHBDL1 | -0.6576 | 3.438584 | -7.8342 | 3.10E-14 | 3.72E-13 | 21.5246 |
| SPX | -0.6567 | 3.860313 | -4.6384 | 4.54E-06 | 2.03E-05 | 3.243 |
| AP003355.2 | -0.6561 | 1.118897 | -6.001 | 3.89E-09 | 2.59E-08 | 10.0572 |
| DGCR6 | -0.6557 | 3.606755 | -6.6668 | 7.26E-11 | 5.98E-10 | 13.9327 |
| OLFM1 | -0.6539 | 6.244857 | -5.7978 | 1.23E-08 | 7.67E-08 | 8.94374 |
| C1QL2 | -0.6539 | 2.468013 | -5.6588 | 2.64E-08 | 1.58E-07 | 8.20101 |
| ASPHD1 | -0.6529 | 5.376816 | -6.3953 | 3.83E-10 | 2.90E-09 | 12.3106 |
| CNTNAP2 | -0.6518 | 3.640379 | -5.3521 | 1.35E-07 | 7.44E-07 | 6.61866 |
| GPR22 | -0.6513 | 1.251421 | -6.1173 | 1.99E-09 | 1.37E-08 | 10.7088 |
| FRRS1L | -0.6503 | 3.790901 | -5.5225 | 5.51E-08 | 3.18E-07 | 7.48826 |
| AC104024.2 | -0.65 | 0.899515 | -6.6162 | 9.94E-11 | 8.08E-10 | 13.6261 |
| STYK1 | -0.6497 | 1.039238 | -6.7099 | 5.55E-11 | 4.64E-10 | 14.1952 |
| VWA5B2 | -0.6496 | 2.48866 | -7.419 | 5.47E-13 | 5.71E-12 | 18.7125 |
| RASAL1 | -0.648 | 2.043987 | -4.9126 | 1.24E-06 | 5.99E-06 | 4.4885 |
| GABRA4 | -0.6478 | 1.369676 | -5.8472 | 9.30E-09 | 5.91E-08 | 9.21141 |
| SRRM3 | -0.6468 | 3.499838 | -5.5482 | 4.80E-08 | 2.79E-07 | 7.62154 |
| FAM155A | -0.6466 | 3.780941 | -6.1305 | 1.84E-09 | 1.28E-08 | 10.784 |
| CYGB | -0.6439 | 2.308449 | -6.0558 | 2.84E-09 | 1.93E-08 | 10.3629 |
| NPY | -0.6429 | 4.576563 | -4.1013 | 4.83E-05 | 0.000186 | 0.9935 |
| DYNC1I1 | -0.6417 | 4.018909 | -5.8195 | 1.09E-08 | 6.84E-08 | 9.06093 |
| AC079946.1 | -0.6402 | 1.462182 | -7.849 | 2.80E-14 | 3.37E-13 | 21.6266 |
| EEF1A2 | -0.6377 | 7.202496 | -4.7211 | 3.09E-06 | 1.41E-05 | 3.61149 |
| AC092720.2 | -0.6367 | 1.684915 | -7.8064 | 3.78E-14 | 4.48E-13 | 21.3324 |
| GJD2 | -0.6344 | 1.231886 | -6.0636 | 2.71E-09 | 1.85E-08 | 10.4064 |
| PNMA6A | -0.634 | 3.422086 | -6.9218 | 1.45E-11 | 1.29E-10 | 15.5068 |
| MIR124-2HG | -0.6334 | 2.367523 | -6.3259 | 5.81E-10 | 4.30E-09 | 11.9051 |
| DNM1 | -0.6326 | 4.93595 | -4.2948 | 2.12E-05 | 8.59E-05 | 1.77458 |
| GRIN2B | -0.6322 | 1.537101 | -5.9499 | 5.21E-09 | 3.42E-08 | 9.77381 |
| HMGCLL1 | -0.6321 | 2.056915 | -6.744 | 4.48E-11 | 3.78E-10 | 14.404 |
| MFSD4A | -0.6306 | 3.283627 | -5.8165 | 1.10E-08 | 6.95E-08 | 9.04486 |
| ATCAY | -0.6305 | 6.67019 | -4.5935 | 5.59E-06 | 2.46E-05 | 3.045 |
| MT-ND6 | -0.6304 | 13.43817 | -9.3277 | 4.10E-19 | 8.43E-18 | 32.573 |
| AC124798.1 | -0.6303 | 2.49876 | -7.7751 | 4.70E-14 | 5.52E-13 | 21.1169 |
| SLIT1 | -0.6302 | 6.135433 | -4.991 | 8.44E-07 | 4.19E-06 | 4.85657 |
| ARPP21 | -0.6299 | 4.071019 | -6.8306 | 2.59E-11 | 2.25E-10 | 14.9382 |
| ISLR2 | -0.6288 | 1.834303 | -5.5433 | 4.93E-08 | 2.86E-07 | 7.59604 |
| BEX2 | -0.6285 | 6.802021 | -6.6045 | 1.07E-10 | 8.65E-10 | 13.5553 |
| CHRNA4 | -0.6272 | 2.726411 | -6.5025 | 2.00E-10 | 1.56E-09 | 12.9444 |
| LINC02440 | -0.6265 | 2.84991 | -5.1615 | 3.61E-07 | 1.88E-06 | 5.6747 |
| FSTL5 | -0.6262 | 2.63452 | -4.7898 | 2.23E-06 | 1.04E-05 | 3.92273 |
| HTR5A-AS1 | -0.6257 | 0.901894 | -6.5197 | 1.80E-10 | 1.42E-09 | 13.0468 |
| SCG3 | -0.6252 | 8.851355 | -5.5007 | 6.19E-08 | 3.55E-07 | 7.37559 |
| LINC02283 | -0.6237 | 5.255938 | -3.5741 | 0.00039 | 0.001281 | -0.9625 |
| PRRT1 | -0.6235 | 4.420934 | -8.7462 | 3.83E-17 | 6.34E-16 | 28.105 |
| CHN1 | -0.6227 | 6.041129 | -5.1705 | 3.45E-07 | 1.80E-06 | 5.71867 |
| PDZD4 | -0.6227 | 7.473279 | -7.6337 | 1.26E-13 | 1.41E-12 | 20.1518 |
| SHISA6 | -0.6224 | 3.094166 | -3.9983 | 7.39E-05 | 0.000275 | 0.59162 |
| CACNA1B | -0.6221 | 2.039884 | -5.6809 | 2.34E-08 | 1.41E-07 | 8.31833 |
| TEF | -0.6211 | 5.697619 | -9.5509 | 6.81E-20 | 1.55E-18 | 34.3408 |
| CPNE5 | -0.6207 | 5.572509 | -5.5308 | 5.27E-08 | 3.05E-07 | 7.53146 |
| ACSL6 | -0.6203 | 4.118572 | -6.7093 | 5.57E-11 | 4.65E-10 | 14.1918 |
| FEZF2 | -0.6203 | 2.154702 | -6.2656 | 8.32E-10 | 6.04E-09 | 11.5559 |
| ASPDH | -0.6201 | 3.275585 | -6.8547 | 2.22E-11 | 1.94E-10 | 15.0877 |
| STXBP1 | -0.6199 | 6.316218 | -6.4954 | 2.09E-10 | 1.63E-09 | 12.902 |
| SERTM1 | -0.6197 | 1.357141 | -5.2736 | 2.03E-07 | 1.09E-06 | 6.22657 |
| ZDHHC22 | -0.6195 | 6.542652 | -5.1669 | 3.51E-07 | 1.83E-06 | 5.70143 |
| PRMT8 | -0.6192 | 1.4954 | -6.1184 | 1.98E-09 | 1.37E-08 | 10.7155 |
| HS3ST2 | -0.6184 | 2.92017 | -4.614 | 5.09E-06 | 2.26E-05 | 3.13508 |
| RXFP1 | -0.6179 | 1.090638 | -6.9005 | 1.66E-11 | 1.47E-10 | 15.3734 |
| AL021395.1 | -0.6166 | 1.324359 | -6.7323 | 4.82E-11 | 4.06E-10 | 14.3321 |
| ATOH8 | -0.616 | 4.923573 | -5.4417 | 8.46E-08 | 4.77E-07 | 7.07324 |
| LYVE1 | -0.6156 | 2.937928 | -4.7278 | 2.99E-06 | 1.37E-05 | 3.64203 |
| GRIA1 | -0.6148 | 5.738317 | -4.7242 | 3.05E-06 | 1.39E-05 | 3.62557 |
| GABRA3 | -0.6112 | 4.352965 | -4.9345 | 1.11E-06 | 5.42E-06 | 4.59059 |
| LINC02217 | -0.6105 | 0.808667 | -7.5815 | 1.80E-13 | 1.99E-12 | 19.7991 |
| GNAL | -0.6098 | 3.526939 | -6.8244 | 2.70E-11 | 2.33E-10 | 14.8999 |
| EMX1 | -0.6092 | 1.09265 | -6.1254 | 1.90E-09 | 1.32E-08 | 10.755 |
| FAM133A | -0.609 | 3.02335 | -6.4839 | 2.24E-10 | 1.74E-09 | 12.8336 |
| FAM81A | -0.6081 | 3.09481 | -6.0666 | 2.67E-09 | 1.82E-08 | 10.4235 |
| AC018358.1 | -0.6079 | 0.727519 | -7.2782 | 1.41E-12 | 1.41E-11 | 17.7867 |
| MRO | -0.6076 | 5.440223 | -6.3418 | 5.29E-10 | 3.93E-09 | 11.9979 |
| CELF5 | -0.6075 | 4.256641 | -5.5349 | 5.15E-08 | 2.99E-07 | 7.55248 |
| PCP4L1 | -0.6071 | 2.995433 | -3.9627 | 8.55E-05 | 0.000315 | 0.45491 |
| CYS1 | -0.607 | 2.886283 | -7.7379 | 6.10E-14 | 7.08E-13 | 20.8619 |
| SLC35F3 | -0.606 | 1.605654 | -6.3089 | 6.43E-10 | 4.74E-09 | 11.8064 |
| UNC13A | -0.605 | 4.680069 | -5.698 | 2.13E-08 | 1.29E-07 | 8.40866 |
| MT-ND1 | -0.605 | 13.70092 | -10.652 | 6.61E-24 | 2.54E-22 | 43.4569 |
| PTPRR | -0.6042 | 1.374562 | -6.1086 | 2.09E-09 | 1.44E-08 | 10.66 |
| MIR770 | -0.6041 | 3.694422 | -3.8606 | 0.00013 | 0.00046 | 0.06925 |
| MCHR2 | -0.6036 | 0.727064 | -6.3859 | 4.06E-10 | 3.06E-09 | 12.2557 |
| CYP46A1 | -0.6009 | 3.410572 | -8.0379 | 7.29E-15 | 9.31E-14 | 22.947 |
| CLEC4GP1 | -0.6002 | 1.021719 | -6.4549 | 2.67E-10 | 2.06E-09 | 12.6617 |
| SDS | -0.6 | 3.323076 | -5.6471 | 2.81E-08 | 1.68E-07 | 8.13951 |
| TLCD3B | -0.5995 | 4.420338 | -6.358 | 4.80E-10 | 3.59E-09 | 12.0924 |
| RNU6-529P | -0.5981 | 5.861358 | -6.0902 | 2.33E-09 | 1.60E-08 | 10.5563 |
| RYR2 | -0.5975 | 1.313942 | -5.5751 | 4.15E-08 | 2.43E-07 | 7.76168 |
| MT-RNR2 | -0.5969 | 14.79945 | -10.179 | 3.77E-22 | 1.15E-20 | 39.4651 |
| SSTR3 | -0.5964 | 0.914529 | -6.8328 | 2.56E-11 | 2.22E-10 | 14.952 |
| NDRG2 | -0.5959 | 9.358185 | -7.4524 | 4.36E-13 | 4.61E-12 | 18.9346 |
| CRH | -0.5936 | 0.965105 | -6.6314 | 9.05E-11 | 7.38E-10 | 13.7181 |
| COL26A1 | -0.5934 | 2.688606 | -5.371 | 1.23E-07 | 6.78E-07 | 6.71405 |
| CBX7 | -0.5927 | 4.777827 | -9.7586 | 1.25E-20 | 3.13E-19 | 36.0113 |
| PTER | -0.5919 | 2.533077 | -4.3227 | 1.88E-05 | 7.67E-05 | 1.88982 |
| C3orf80 | -0.5898 | 1.417966 | -5.536 | 5.12E-08 | 2.97E-07 | 7.55811 |
| FBLL1 | -0.5885 | 4.968899 | -5.7271 | 1.81E-08 | 1.11E-07 | 8.56423 |
| AL031710.1 | -0.5866 | 1.037351 | -7.5871 | 1.74E-13 | 1.92E-12 | 19.8365 |
| CYP4X1 | -0.5844 | 1.769285 | -6.3504 | 5.02E-10 | 3.74E-09 | 12.0482 |
| EPHA10 | -0.5835 | 1.877784 | -6.8311 | 2.58E-11 | 2.24E-10 | 14.9413 |
| ELAVL2 | -0.5818 | 3.274969 | -5.2348 | 2.48E-07 | 1.32E-06 | 6.03423 |
| AC107398.3 | -0.5813 | 3.879386 | -5.213 | 2.78E-07 | 1.47E-06 | 5.92707 |
| TMEM179 | -0.5796 | 4.190993 | -4.4165 | 1.24E-05 | 5.21E-05 | 2.28283 |
| PLK5 | -0.5796 | 1.812492 | -8.0525 | 6.56E-15 | 8.44E-14 | 23.0497 |
| AL513217.1 | -0.5795 | 2.236025 | -5.835 | 9.96E-09 | 6.31E-08 | 9.1447 |
| NRXN3 | -0.5794 | 2.351136 | -5.4474 | 8.21E-08 | 4.64E-07 | 7.10226 |
| CRY2 | -0.5793 | 5.671349 | -9.5995 | 4.59E-20 | 1.07E-18 | 34.729 |
| CACNG8 | -0.5788 | 3.344513 | -5.4696 | 7.30E-08 | 4.15E-07 | 7.21581 |
| CDH18 | -0.5779 | 2.41539 | -5.0449 | 6.47E-07 | 3.27E-06 | 5.11243 |
| AC110491.1 | -0.5776 | 1.367489 | -5.8078 | 1.16E-08 | 7.28E-08 | 8.99755 |
| SOWAHB | -0.5771 | 0.857258 | -6.3431 | 5.25E-10 | 3.90E-09 | 12.0051 |
| GNG13 | -0.577 | 1.353518 | -5.9867 | 4.22E-09 | 2.80E-08 | 9.97769 |
| STXBP6 | -0.5768 | 2.985808 | -6.4135 | 3.44E-10 | 2.61E-09 | 12.4175 |
| KCNAB2 | -0.5761 | 4.344933 | -5.3056 | 1.72E-07 | 9.35E-07 | 6.38572 |
| MT-TT | -0.5748 | 3.673123 | -5.3782 | 1.18E-07 | 6.55E-07 | 6.75054 |
| SHANK2 | -0.5748 | 2.548646 | -6.1211 | 1.94E-09 | 1.35E-08 | 10.7307 |
| CA7 | -0.5746 | 1.222884 | -5.668 | 2.51E-08 | 1.51E-07 | 8.2497 |
| PPP1R1B | -0.5743 | 5.125547 | -4.2461 | 2.62E-05 | 0.000105 | 1.5749 |
| NOG | -0.5731 | 4.387801 | -4.645 | 4.41E-06 | 1.97E-05 | 3.27226 |
| BSN | -0.5717 | 3.736826 | -5.5386 | 5.05E-08 | 2.93E-07 | 7.57187 |
| CHRNB2 | -0.5702 | 3.786657 | -5.0943 | 5.05E-07 | 2.59E-06 | 5.34963 |
| MTRNR2L12 | -0.5702 | 3.179569 | -6.8704 | 2.01E-11 | 1.77E-10 | 15.1852 |
| CAMKK1 | -0.5698 | 3.343795 | -4.9624 | 9.71E-07 | 4.77E-06 | 4.72176 |
| RALYL | -0.5689 | 2.531014 | -5.2721 | 2.05E-07 | 1.10E-06 | 6.2191 |
| NEGR1 | -0.5689 | 3.180138 | -5.8733 | 8.03E-09 | 5.15E-08 | 9.3534 |
| ZMAT4 | -0.5686 | 1.600573 | -5.6016 | 3.60E-08 | 2.13E-07 | 7.90012 |
| AL033519.1 | -0.568 | 2.027954 | -4.3753 | 1.49E-05 | 6.19E-05 | 2.10938 |
| RIMBP2 | -0.5666 | 2.060753 | -4.8807 | 1.45E-06 | 6.92E-06 | 4.34009 |
| YPEL4 | -0.5664 | 3.707392 | -6.4846 | 2.23E-10 | 1.74E-09 | 12.8382 |
| ELFN2 | -0.5654 | 4.798884 | -4.6271 | 4.79E-06 | 2.13E-05 | 3.19298 |
| MAL | -0.5652 | 5.333544 | -3.2216 | 0.00136 | 0.004074 | -2.1283 |
| SNORC | -0.5632 | 3.79365 | -4.1405 | 4.10E-05 | 0.000159 | 1.14906 |
| BICDL1 | -0.5631 | 1.867081 | -6.1103 | 2.07E-09 | 1.43E-08 | 10.6695 |
| CELF2-DT | -0.562 | 0.857165 | -6.1214 | 1.94E-09 | 1.35E-08 | 10.7323 |
| RIMS3 | -0.5611 | 4.360604 | -4.8747 | 1.49E-06 | 7.11E-06 | 4.31233 |
| KIF5A | -0.5606 | 7.144164 | -4.9272 | 1.15E-06 | 5.60E-06 | 4.55681 |
| DUSP9 | -0.5604 | 2.49303 | -5.1258 | 4.32E-07 | 2.23E-06 | 5.50157 |
| SEZ6L | -0.5601 | 6.864837 | -4.1269 | 4.34E-05 | 0.000168 | 1.09511 |
| CDH9 | -0.5597 | 1.044783 | -6.5494 | 1.50E-10 | 1.20E-09 | 13.2246 |
| MT-ND2 | -0.5596 | 13.62681 | -10.312 | 1.22E-22 | 3.95E-21 | 40.5768 |
| TCEAL2 | -0.5595 | 7.519206 | -7.9166 | 1.73E-14 | 2.13E-13 | 22.0962 |
| MYL3 | -0.5594 | 2.710543 | -6.8464 | 2.34E-11 | 2.05E-10 | 15.0361 |
| LYNX1 | -0.5592 | 5.589141 | -6.8205 | 2.76E-11 | 2.39E-10 | 14.8755 |
| SLC17A6 | -0.5577 | 1.266044 | -5.399 | 1.06E-07 | 5.92E-07 | 6.85578 |
| PCDH8 | -0.5575 | 2.565472 | -4.7324 | 2.93E-06 | 1.34E-05 | 3.66256 |
| MTND6P4 | -0.5574 | 2.567678 | -5.4007 | 1.05E-07 | 5.87E-07 | 6.86432 |
| ALDH2 | -0.5566 | 6.598337 | -9.7864 | 9.96E-21 | 2.53E-19 | 36.2361 |
| PDIA2 | -0.556 | 3.402137 | -4.8853 | 1.41E-06 | 6.78E-06 | 4.36131 |
| MARCHF11 | -0.5554 | 1.057168 | -7.1462 | 3.37E-12 | 3.24E-11 | 16.9317 |
| TAC3 | -0.5553 | 1.791464 | -4.3209 | 1.89E-05 | 7.73E-05 | 1.88228 |
| AC002428.2 | -0.5538 | 1.518014 | -5.1333 | 4.16E-07 | 2.15E-06 | 5.53775 |
| PLPPR3 | -0.5531 | 3.098329 | -4.3884 | 1.41E-05 | 5.86E-05 | 2.16435 |
| HS6ST3 | -0.5527 | 1.957179 | -4.0284 | 6.53E-05 | 0.000245 | 0.70825 |
| RPRML | -0.5514 | 1.348216 | -5.5788 | 4.07E-08 | 2.39E-07 | 7.7808 |
| HPR | -0.5508 | 3.834047 | -3.838 | 0.00014 | 0.000501 | -0.0149 |
| KCNQ5 | -0.5485 | 3.122246 | -5.3457 | 1.40E-07 | 7.68E-07 | 6.58681 |
| RASD2 | -0.5485 | 3.120202 | -4.8452 | 1.71E-06 | 8.13E-06 | 4.17608 |
| AL731533.2 | -0.5477 | 2.931687 | -7.8096 | 3.69E-14 | 4.38E-13 | 21.3545 |
| MICAL2 | -0.5472 | 2.257655 | -4.6839 | 3.68E-06 | 1.66E-05 | 3.44524 |
| FBXO41 | -0.5464 | 4.075617 | -5.7036 | 2.06E-08 | 1.25E-07 | 8.43889 |
| SCD | -0.5446 | 9.499706 | -6.1429 | 1.71E-09 | 1.19E-08 | 10.8543 |
| CDHR1 | -0.5445 | 4.294378 | -4.7979 | 2.15E-06 | 1.01E-05 | 3.95956 |
| MIR6071 | -0.5444 | 2.995396 | -4.0363 | 6.33E-05 | 0.000238 | 0.73893 |
| RIPPLY2 | -0.5423 | 4.120777 | -6.6465 | 8.24E-11 | 6.75E-10 | 13.8096 |
| RTN4RL2 | -0.5414 | 3.705517 | -5.3143 | 1.65E-07 | 8.96E-07 | 6.42905 |
| KCNK1 | -0.5406 | 3.538694 | -4.4492 | 1.07E-05 | 4.55E-05 | 2.42147 |
| EIF4E1B | -0.5405 | 0.658767 | -8.7345 | 4.19E-17 | 6.91E-16 | 28.0171 |
| CA4 | -0.5403 | 3.568238 | -5.1891 | 3.14E-07 | 1.65E-06 | 5.80951 |
| MT-ATP6 | -0.5399 | 14.34842 | -11.285 | 2.47E-26 | 1.33E-24 | 48.9781 |
| KCNK12 | -0.5397 | 1.884525 | -5.5493 | 4.77E-08 | 2.78E-07 | 7.62733 |
| TRHDE | -0.539 | 0.844491 | -6.8404 | 2.44E-11 | 2.12E-10 | 14.9987 |
| TPPP | -0.5382 | 6.287595 | -4.873 | 1.50E-06 | 7.16E-06 | 4.30471 |
| HS3ST4 | -0.538 | 2.433887 | -4.7088 | 3.27E-06 | 1.49E-05 | 3.55649 |
| MT-TC | -0.5374 | 4.54217 | -5.5103 | 5.88E-08 | 3.38E-07 | 7.42521 |
| MTCO1P12 | -0.5366 | 7.908426 | -6.6546 | 7.83E-11 | 6.44E-10 | 13.8587 |
| RAB26 | -0.536 | 3.110829 | -6.4369 | 2.98E-10 | 2.28E-09 | 12.5555 |
| CLVS1 | -0.5359 | 1.440005 | -5.7579 | 1.53E-08 | 9.45E-08 | 8.72877 |
| LINC01476 | -0.5355 | 0.538108 | -6.8934 | 1.74E-11 | 1.54E-10 | 15.329 |
| PYDC1 | -0.5338 | 0.67356 | -7.7263 | 6.62E-14 | 7.65E-13 | 20.7819 |
| AF106564.1 | -0.5338 | 1.764708 | -6.4626 | 2.55E-10 | 1.97E-09 | 12.7077 |
| LHX6 | -0.5334 | 1.34688 | -5.8372 | 9.83E-09 | 6.24E-08 | 9.15691 |
| RIMS1 | -0.5332 | 2.073007 | -5.2071 | 2.86E-07 | 1.51E-06 | 5.89798 |
| SEMA4A | -0.5324 | 3.143839 | -6.7351 | 4.74E-11 | 3.99E-10 | 14.3492 |
| AC012213.1 | -0.5319 | 1.783386 | -4.9859 | 8.65E-07 | 4.29E-06 | 4.83256 |
| SHD | -0.5315 | 6.584928 | -3.4262 | 0.00067 | 0.002108 | -1.4657 |
| LINC01785 | -0.5309 | 0.813596 | -7.3217 | 1.05E-12 | 1.07E-11 | 18.0713 |
| DGCR10 | -0.5307 | 2.239441 | -6.2331 | 1.01E-09 | 7.24E-09 | 11.3689 |
| AL354798.1 | -0.5306 | 2.499836 | -5.9315 | 5.78E-09 | 3.78E-08 | 9.67251 |
| TSPOAP1 | -0.529 | 4.708201 | -6.5531 | 1.47E-10 | 1.17E-09 | 13.2468 |
| KCNS2 | -0.529 | 0.981863 | -5.4038 | 1.03E-07 | 5.78E-07 | 6.88026 |
| ANKRD24 | -0.5285 | 3.331312 | -7.2556 | 1.64E-12 | 1.62E-11 | 17.6394 |
| RCAN2 | -0.5284 | 4.733683 | -4.4789 | 9.41E-06 | 4.02E-05 | 2.54815 |
| MT-CYB | -0.5281 | 13.84517 | -9.6698 | 2.59E-20 | 6.22E-19 | 35.2937 |
| SEPTIN5 | -0.5277 | 5.778234 | -5.5954 | 3.72E-08 | 2.19E-07 | 7.86774 |
| HSPA12A | -0.5275 | 4.370546 | -5.8256 | 1.05E-08 | 6.62E-08 | 9.09401 |
| RPSAP69 | -0.5274 | 0.679038 | -7.3851 | 6.87E-13 | 7.08E-12 | 18.4884 |
| RIT2 | -0.5272 | 2.644774 | -3.43 | 0.00066 | 0.002081 | -1.4532 |
| TAC1 | -0.5263 | 2.450003 | -3.2499 | 0.00124 | 0.003724 | -2.039 |
| FADS6 | -0.526 | 0.690238 | -6.7928 | 3.29E-11 | 2.82E-10 | 14.7045 |
| AC104051.2 | -0.5255 | 2.335273 | -2.3108 | 0.02127 | 0.048425 | -4.5996 |
| FAM131C | -0.5245 | 2.839323 | -5.1296 | 4.23E-07 | 2.19E-06 | 5.52004 |
| TUBB4A | -0.5212 | 8.092545 | -4.7969 | 2.16E-06 | 1.01E-05 | 3.95495 |
| DGCR9 | -0.5207 | 3.003062 | -5.7502 | 1.60E-08 | 9.84E-08 | 8.68775 |
| AC015967.1 | -0.5204 | 1.773471 | -8.4439 | 3.74E-16 | 5.49E-15 | 25.8645 |
| KCTD16 | -0.5203 | 1.602783 | -5.9388 | 5.55E-09 | 3.63E-08 | 9.71245 |
| CRHR1 | -0.5202 | 2.210063 | -6.651 | 8.01E-11 | 6.57E-10 | 13.8364 |
| LINC01202 | -0.5202 | 0.585176 | -6.2825 | 7.53E-10 | 5.49E-09 | 11.6535 |
| SYNGR1 | -0.5201 | 5.790853 | -6.5528 | 1.47E-10 | 1.17E-09 | 13.2445 |
| SCGN | -0.5196 | 1.005885 | -4.6543 | 4.22E-06 | 1.89E-05 | 3.3132 |
| GPR61 | -0.5192 | 1.347807 | -8.0047 | 9.24E-15 | 1.17E-13 | 22.7135 |
| KBTBD11-OT1 | -0.5187 | 3.39052 | -4.5297 | 7.48E-06 | 3.24E-05 | 2.76718 |
| INSM2 | -0.5173 | 1.204925 | -5.2959 | 1.81E-07 | 9.81E-07 | 6.33714 |
| DLGAP2 | -0.5165 | 1.245175 | -5.9275 | 5.91E-09 | 3.86E-08 | 9.6506 |
| HHATL | -0.5162 | 4.520328 | -4.1021 | 4.82E-05 | 0.000185 | 0.99681 |
| TENM2 | -0.5157 | 1.664878 | -4.819 | 1.94E-06 | 9.16E-06 | 4.05595 |
| AC134312.3 | -0.5152 | 0.88842 | -5.4568 | 7.81E-08 | 4.43E-07 | 7.1501 |
| AL359091.1 | -0.5138 | 4.306844 | -4.2444 | 2.64E-05 | 0.000105 | 1.5679 |
| SLC7A4 | -0.5138 | 1.295721 | -5.8626 | 8.53E-09 | 5.44E-08 | 9.29528 |
| BASP1 | -0.5135 | 7.542851 | -5.6919 | 2.20E-08 | 1.33E-07 | 8.37648 |
| PCBP3 | -0.5128 | 2.957455 | -6.1095 | 2.08E-09 | 1.43E-08 | 10.6649 |
| PCSK1N | -0.5124 | 7.17378 | -5.4794 | 6.93E-08 | 3.95E-07 | 7.26594 |
| CISTR | -0.5114 | 1.737113 | -5.4904 | 6.54E-08 | 3.74E-07 | 7.32284 |
| GPR6 | -0.5112 | 0.606523 | -7.5153 | 2.84E-13 | 3.07E-12 | 19.354 |
| GRM3 | -0.51 | 3.894917 | -4.0435 | 6.14E-05 | 0.000232 | 0.76695 |
| KCNC1 | -0.5098 | 3.036209 | -5.43 | 9.00E-08 | 5.06E-07 | 7.01337 |
| DNMBP-AS1 | -0.5095 | 1.45502 | -5.6916 | 2.20E-08 | 1.33E-07 | 8.37488 |
| SMOC1 | -0.5094 | 8.411645 | -3.2256 | 0.00134 | 0.004024 | -2.1158 |
| RAP1GAP2 | -0.5093 | 3.437057 | -4.6864 | 3.64E-06 | 1.65E-05 | 3.45617 |
| SLC1A6 | -0.5092 | 2.039142 | -5.9789 | 4.41E-09 | 2.92E-08 | 9.93428 |
| AGAP2 | -0.5075 | 5.186674 | -4.4608 | 1.02E-05 | 4.33E-05 | 2.47086 |
| GPR83 | -0.5074 | 1.155845 | -5.135 | 4.12E-07 | 2.13E-06 | 5.54629 |
| STEAP2 | -0.5074 | 2.514979 | -5.913 | 6.42E-09 | 4.17E-08 | 9.57071 |
| GALNT13 | -0.5071 | 5.870046 | -3.8617 | 0.00013 | 0.000458 | 0.07309 |
| TNNT2 | -0.5064 | 0.646976 | -6.6715 | 7.05E-11 | 5.82E-10 | 13.961 |
| AL391834.1 | -0.506 | 3.501262 | -6.0454 | 3.01E-09 | 2.04E-08 | 10.3045 |
| CBLN4 | -0.5059 | 1.603554 | -3.4798 | 0.00055 | 0.001766 | -1.2858 |
| GRAMD1B | -0.5049 | 4.607246 | -6.8384 | 2.47E-11 | 2.15E-10 | 14.9864 |
| KCNB1 | -0.5036 | 4.455521 | -5.8565 | 8.82E-09 | 5.62E-08 | 9.26197 |
| AC138649.1 | -0.5033 | 1.893054 | -5.0423 | 6.55E-07 | 3.31E-06 | 5.10001 |
| MT-TP | -0.5023 | 10.96847 | -6.429 | 3.13E-10 | 2.39E-09 | 12.5088 |
| LGI3 | -0.502 | 4.921313 | -3.9188 | 0.0001 | 0.000371 | 0.28772 |
| LRRC73 | -0.5019 | 2.613676 | -6.2156 | 1.12E-09 | 7.98E-09 | 11.2686 |
| LDHD | -0.5019 | 4.612273 | -7.792 | 4.18E-14 | 4.93E-13 | 21.2331 |
| TMEM151A | -0.5016 | 4.830281 | -4.2296 | 2.81E-05 | 0.000112 | 1.50763 |
| SLC6A15 | -0.5013 | 1.576628 | -4.6505 | 4.30E-06 | 1.92E-05 | 3.29636 |
| GPR26 | -0.5011 | 0.74485 | -5.5119 | 5.83E-08 | 3.36E-07 | 7.43334 |
| UNC79 | -0.5004 | 3.375841 | -4.6051 | 5.30E-06 | 2.35E-05 | 3.0958 |
| KRT17 | -0.5003 | 1.18187 | -4.5189 | 7.85E-06 | 3.39E-05 | 2.72021 |
