# Supplementary material for: MGME1 associates with poor prognosis and is vital for cell proliferation in lower-grade glioma
Source: Aging (Albany NY). 2023 May 8;15(9):3690–714. doi: 10.18632/aging.204705 (PMC10449294; doi:10.18632/aging.204705)
Supplement: Supplementary Table 2 [file aging-15-204705-s003.docx]

**Supplementary Table 2. Up-regulated DEGs in TCGA dataset.**

| **id** | **logFC** | **AveExpr** | **t** | **P.Value** | **adj.P.Val** | **B** |
| --- | --- | --- | --- | --- | --- | --- |
| ZNF137P | 0.5 | 1.677281 | 12.234 | 4.09E-30 | 3.79E-28 | 57.5847 |
| HOXB-AS1 | 0.50005 | 0.548285 | 6.91392 | 1.52E-11 | 1.36E-10 | 15.4572 |
| PAG1 | 0.50009 | 4.131502 | 8.58761 | 1.27E-16 | 1.98E-15 | 26.9222 |
| VPS54 | 0.50014 | 4.138487 | 13.9406 | 2.78E-37 | 9.45E-35 | 73.9198 |
| RAB8A | 0.50018 | 3.954036 | 13.9139 | 3.62E-37 | 1.22E-34 | 73.657 |
| VEZF1 | 0.50023 | 5.847059 | 10.8975 | 7.72E-25 | 3.42E-23 | 45.5773 |
| VWF | 0.50035 | 4.952191 | 5.61897 | 3.27E-08 | 1.95E-07 | 7.99125 |
| SLC35F5 | 0.50039 | 3.595491 | 10.7484 | 2.85E-24 | 1.15E-22 | 44.2873 |
| CDH1 | 0.50042 | 1.836741 | 4.87915 | 1.46E-06 | 6.97E-06 | 4.33304 |
| PPCS | 0.50043 | 4.055943 | 6.59189 | 1.16E-10 | 9.31E-10 | 13.4795 |
| GBGT1 | 0.50049 | 2.35815 | 8.14452 | 3.38E-15 | 4.47E-14 | 23.7025 |
| AGA | 0.50063 | 4.317116 | 8.90938 | 1.09E-17 | 1.94E-16 | 29.3381 |
| MS4A4E | 0.50086 | 0.651782 | 8.19618 | 2.32E-15 | 3.12E-14 | 24.0713 |
| DIAPH1 | 0.50086 | 4.725126 | 10.1521 | 4.74E-22 | 1.42E-20 | 39.2406 |
| PSMC3IP | 0.50109 | 3.041823 | 10.8297 | 1.40E-24 | 5.93E-23 | 44.9892 |
| PSPH | 0.50113 | 5.176073 | 8.71515 | 4.85E-17 | 7.94E-16 | 27.8722 |
| HCG15 | 0.50175 | 1.486459 | 9.18599 | 1.26E-18 | 2.46E-17 | 31.4654 |
| IPP | 0.50178 | 3.682161 | 11.3081 | 2.01E-26 | 1.10E-24 | 49.1831 |
| SLC31A1 | 0.50183 | 4.64844 | 10.7104 | 3.97E-24 | 1.57E-22 | 43.961 |
| CCDC40 | 0.50184 | 2.069856 | 6.79219 | 3.31E-11 | 2.83E-10 | 14.7005 |
| DDX5 | 0.50184 | 7.90353 | 11.5824 | 1.68E-27 | 1.08E-25 | 51.6337 |
| C1GALT1C1L | 0.50187 | 2.522485 | 5.73743 | 1.71E-08 | 1.05E-07 | 8.61921 |
| ZNF618 | 0.50188 | 1.987267 | 8.62636 | 9.52E-17 | 1.50E-15 | 27.2098 |
| FTL | 0.50203 | 12.01203 | 6.98476 | 9.66E-12 | 8.80E-11 | 15.9027 |
| HNRNPH1 | 0.50205 | 6.350307 | 10.8641 | 1.04E-24 | 4.50E-23 | 45.287 |
| MDK | 0.50221 | 5.356403 | 4.235 | 2.74E-05 | 0.000109 | 1.52962 |
| ZNF567 | 0.50239 | 2.359125 | 11.6133 | 1.27E-27 | 8.35E-26 | 51.9125 |
| DYRK3 | 0.50251 | 1.957163 | 9.35179 | 3.38E-19 | 7.05E-18 | 32.7623 |
| ZNF211 | 0.50252 | 3.951417 | 10.0793 | 8.74E-22 | 2.53E-20 | 38.6363 |
| SP110 | 0.50256 | 1.730886 | 10.2801 | 1.60E-22 | 5.09E-21 | 40.3084 |
| APOBEC3F | 0.50273 | 1.139716 | 9.2037 | 1.10E-18 | 2.15E-17 | 31.6032 |
| PHF13 | 0.50283 | 4.467407 | 10.3991 | 5.82E-23 | 1.97E-21 | 41.3088 |
| UGGT1 | 0.50284 | 4.272858 | 11.7586 | 3.36E-28 | 2.42E-26 | 53.226 |
| KIAA1958 | 0.50288 | 3.125859 | 10.0409 | 1.20E-21 | 3.44E-20 | 38.3194 |
| MIR186 | 0.50288 | 3.00585 | 4.64889 | 4.33E-06 | 1.94E-05 | 3.28928 |
| TAF12 | 0.5029 | 4.495789 | 12.4227 | 6.94E-31 | 7.34E-29 | 59.3403 |
| SPIN4 | 0.50295 | 2.309268 | 9.55063 | 6.83E-20 | 1.56E-18 | 34.3384 |
| ATP11B | 0.50303 | 3.256737 | 10.1339 | 5.52E-22 | 1.64E-20 | 39.0889 |
| EGR2 | 0.5032 | 3.588675 | 3.4768 | 0.00055 | 0.001784 | -1.2959 |
| PICALM | 0.50321 | 6.550892 | 13.4367 | 4.02E-35 | 9.22E-33 | 68.9948 |
| ZNF528-AS1 | 0.50338 | 3.881494 | 7.56095 | 2.08E-13 | 2.27E-12 | 19.6605 |
| SLC29A1 | 0.50342 | 4.763972 | 9.07339 | 3.05E-18 | 5.70E-17 | 30.594 |
| HOXB4 | 0.50343 | 0.371959 | 6.4494 | 2.76E-10 | 2.13E-09 | 12.6295 |
| LINC01224 | 0.50409 | 0.564211 | 8.42911 | 4.17E-16 | 6.10E-15 | 25.756 |
| GART | 0.50415 | 4.867486 | 11.6103 | 1.31E-27 | 8.57E-26 | 51.8849 |
| SOCS4 | 0.50418 | 3.97344 | 12.3457 | 1.43E-30 | 1.42E-28 | 58.6222 |
| SLC6A9 | 0.50424 | 5.290717 | 4.96509 | 9.59E-07 | 4.72E-06 | 4.73429 |
| MYH9 | 0.50424 | 6.55998 | 9.75869 | 1.25E-20 | 3.13E-19 | 36.0117 |
| ITGA1 | 0.50434 | 1.892389 | 8.20134 | 2.23E-15 | 3.01E-14 | 24.1083 |
| ZGRF1 | 0.50443 | 1.638516 | 10.5336 | 1.84E-23 | 6.59E-22 | 42.4486 |
| FGFRL1 | 0.50446 | 4.051413 | 5.07198 | 5.65E-07 | 2.88E-06 | 5.24215 |
| SNORA5C | 0.50447 | 2.659796 | 6.91327 | 1.53E-11 | 1.36E-10 | 15.4531 |
| TM7SF3 | 0.50454 | 5.521588 | 10.4603 | 3.45E-23 | 1.20E-21 | 41.8267 |
| LINC01094 | 0.50464 | 4.807047 | 4.67384 | 3.85E-06 | 1.74E-05 | 3.40017 |
| PRIMPOL | 0.50465 | 3.833093 | 12.4964 | 3.46E-31 | 3.84E-29 | 60.0298 |
| PAXBP1 | 0.505 | 4.561463 | 8.7831 | 2.89E-17 | 4.84E-16 | 28.3823 |
| LYST | 0.505 | 3.020605 | 9.52232 | 8.59E-20 | 1.93E-18 | 34.1126 |
| TOP1 | 0.50505 | 6.39565 | 11.3197 | 1.81E-26 | 1.00E-24 | 49.2855 |
| P4HB | 0.50514 | 7.290705 | 11.6183 | 1.21E-27 | 8.02E-26 | 51.9575 |
| HNF4G | 0.50548 | 1.425357 | 9.03396 | 4.16E-18 | 7.66E-17 | 30.2906 |
| MMP7 | 0.50548 | 0.655898 | 5.74541 | 1.64E-08 | 1.01E-07 | 8.66193 |
| IKBKB | 0.50561 | 4.112206 | 13.3017 | 1.50E-34 | 3.17E-32 | 67.6893 |
| SNRPB | 0.50564 | 7.415729 | 10.7652 | 2.46E-24 | 1.00E-22 | 44.4322 |
| RAB27A | 0.50571 | 1.929379 | 9.84074 | 6.36E-21 | 1.66E-19 | 36.6781 |
| SCYL2 | 0.50578 | 4.177736 | 11.6504 | 9.05E-28 | 6.09E-26 | 52.2471 |
| POLA1 | 0.50602 | 3.811647 | 10.1339 | 5.52E-22 | 1.64E-20 | 39.0886 |
| TOPBP1 | 0.50611 | 4.488709 | 10.4183 | 4.94E-23 | 1.68E-21 | 41.4715 |
| ZNF724 | 0.50617 | 1.186245 | 9.28314 | 5.84E-19 | 1.18E-17 | 32.2234 |
| PLEK2 | 0.50623 | 0.756341 | 8.82037 | 2.17E-17 | 3.69E-16 | 28.6634 |
| EIF4EBP1 | 0.5063 | 5.754211 | 6.57933 | 1.25E-10 | 1.00E-09 | 13.404 |
| HIF1A | 0.50636 | 7.123464 | 7.64294 | 1.18E-13 | 1.33E-12 | 20.2143 |
| BAZ1B | 0.50653 | 5.970253 | 11.8466 | 1.50E-28 | 1.13E-26 | 54.0257 |
| C16orf54 | 0.50657 | 1.000683 | 10.2799 | 1.61E-22 | 5.09E-21 | 40.3066 |
| AC069499.2 | 0.50661 | 1.224626 | 8.52644 | 2.02E-16 | 3.06E-15 | 26.4702 |
| DCAF13 | 0.50663 | 3.74068 | 12.3684 | 1.16E-30 | 1.17E-28 | 58.8335 |
| NRP1 | 0.50696 | 3.221988 | 7.34206 | 9.18E-13 | 9.36E-12 | 18.205 |
| RAVER1 | 0.50697 | 4.41308 | 7.97563 | 1.14E-14 | 1.43E-13 | 22.5091 |
| HAGLR | 0.50712 | 2.396077 | 4.82143 | 1.92E-06 | 9.06E-06 | 4.06707 |
| AC138207.3 | 0.50723 | 3.220312 | 4.50438 | 8.39E-06 | 3.61E-05 | 2.65779 |
| DPYSL5 | 0.50746 | 5.236454 | 5.83868 | 9.75E-09 | 6.19E-08 | 9.16494 |
| C21orf58 | 0.50759 | 2.110729 | 8.41748 | 4.55E-16 | 6.62E-15 | 25.671 |
| DENND1C | 0.50766 | 2.004565 | 8.65991 | 7.38E-17 | 1.18E-15 | 27.4595 |
| LAMC3 | 0.50775 | 2.35467 | 6.64213 | 8.47E-11 | 6.93E-10 | 13.7829 |
| HBP1 | 0.5079 | 4.264564 | 10.8641 | 1.04E-24 | 4.50E-23 | 45.2878 |
| CABP4 | 0.50798 | 0.905756 | 10.5179 | 2.10E-23 | 7.48E-22 | 42.3154 |
| LHFPL6 | 0.508 | 6.494352 | 7.01633 | 7.88E-12 | 7.26E-11 | 16.1023 |
| SFMBT1 | 0.50808 | 2.757391 | 13.3151 | 1.32E-34 | 2.81E-32 | 67.8183 |
| STC1 | 0.50809 | 0.827924 | 5.81449 | 1.12E-08 | 7.03E-08 | 9.03377 |
| CEP112 | 0.50823 | 1.684372 | 8.50486 | 2.37E-16 | 3.56E-15 | 26.3113 |
| SPRY2 | 0.50826 | 5.754301 | 6.49097 | 2.15E-10 | 1.67E-09 | 12.8759 |
| STT3A | 0.50828 | 5.186997 | 12.1038 | 1.38E-29 | 1.19E-27 | 56.3817 |
| GBP4 | 0.50831 | 2.877681 | 6.04934 | 2.95E-09 | 2.00E-08 | 10.3267 |
| PRDX4 | 0.50857 | 6.078823 | 10.1388 | 5.30E-22 | 1.58E-20 | 39.1294 |
| EFNA1 | 0.50866 | 4.84141 | 7.57399 | 1.90E-13 | 2.09E-12 | 19.7483 |
| SEPTIN9 | 0.50872 | 6.113585 | 10.7779 | 2.20E-24 | 9.05E-23 | 44.5421 |
| UTP20 | 0.50872 | 3.122243 | 12.0263 | 2.84E-29 | 2.33E-27 | 55.6689 |
| FBN3 | 0.50873 | 1.150821 | 5.7733 | 1.40E-08 | 8.72E-08 | 8.81158 |
| ZNF606 | 0.50901 | 3.184846 | 11.0728 | 1.64E-25 | 7.93E-24 | 47.1066 |
| AC069282.1 | 0.50915 | 3.658554 | 8.63249 | 9.09E-17 | 1.44E-15 | 27.2553 |
| PANX1 | 0.50927 | 4.522861 | 8.4796 | 2.87E-16 | 4.26E-15 | 26.1258 |
| FABP7 | 0.50942 | 5.983055 | 3.64945 | 0.00029 | 0.000987 | -0.6986 |
| MTBP | 0.50957 | 1.306391 | 11.6682 | 7.69E-28 | 5.23E-26 | 52.4079 |
| CCDC14 | 0.5096 | 3.746247 | 8.19211 | 2.39E-15 | 3.21E-14 | 24.0422 |
| EFHC1 | 0.5097 | 3.72919 | 9.54826 | 6.96E-20 | 1.58E-18 | 34.3195 |
| RIPK3 | 0.50972 | 1.30268 | 10.3421 | 9.47E-23 | 3.10E-21 | 40.8292 |
| ZDHHC1 | 0.50987 | 3.059709 | 5.61295 | 3.38E-08 | 2.01E-07 | 7.95967 |
| RRP1B | 0.50992 | 4.63203 | 12.7851 | 2.21E-32 | 3.06E-30 | 62.7492 |
| PDS5A | 0.51004 | 4.536646 | 14.5484 | 6.23E-40 | 4.38E-37 | 79.959 |
| COQ2 | 0.51009 | 3.218008 | 12.3236 | 1.76E-30 | 1.72E-28 | 58.4171 |
| TUT7 | 0.51015 | 3.436895 | 11.8355 | 1.66E-28 | 1.24E-26 | 53.9248 |
| CSF1 | 0.51026 | 5.626948 | 6.17337 | 1.43E-09 | 1.01E-08 | 11.0272 |
| CEPT1 | 0.51052 | 3.095561 | 12.2124 | 5.01E-30 | 4.54E-28 | 57.3848 |
| ZNF345 | 0.51061 | 1.939558 | 13.0912 | 1.16E-33 | 2.02E-31 | 65.6648 |
| AL035461.3 | 0.51071 | 2.655876 | 7.69777 | 8.07E-14 | 9.23E-13 | 20.5873 |
| FLII | 0.51107 | 5.546645 | 14.1026 | 5.52E-38 | 2.31E-35 | 75.5192 |
| ABRACL | 0.51124 | 3.723644 | 7.40678 | 5.94E-13 | 6.17E-12 | 18.6318 |
| GSTK1 | 0.51165 | 5.765985 | 12.4121 | 7.67E-31 | 8.01E-29 | 59.2415 |
| SLC8B1 | 0.51165 | 3.518273 | 8.47614 | 2.94E-16 | 4.37E-15 | 26.1003 |
| WDR1 | 0.51173 | 6.475056 | 12.0473 | 2.34E-29 | 1.94E-27 | 55.8626 |
| TBCCD1 | 0.51176 | 3.80534 | 12.9766 | 3.52E-33 | 5.35E-31 | 64.5695 |
| URB1 | 0.51177 | 4.252916 | 9.75808 | 1.26E-20 | 3.14E-19 | 36.0067 |
| PSMB8 | 0.51193 | 5.753926 | 6.83133 | 2.58E-11 | 2.24E-10 | 14.9426 |
| PPM1D | 0.51214 | 3.786507 | 10.8627 | 1.05E-24 | 4.55E-23 | 45.2756 |
| GPX1 | 0.51226 | 8.650611 | 8.63989 | 8.59E-17 | 1.36E-15 | 27.3103 |
| MTX1P1 | 0.51231 | 3.863411 | 8.82453 | 2.10E-17 | 3.59E-16 | 28.6949 |
| TMEM69 | 0.51239 | 4.70026 | 13.1843 | 4.71E-34 | 9.03E-32 | 66.5588 |
| AL049871.1 | 0.5124 | 0.629386 | 7.34254 | 9.15E-13 | 9.33E-12 | 18.2081 |
| ARRDC4 | 0.51247 | 5.187715 | 6.68385 | 6.53E-11 | 5.41E-10 | 14.0364 |
| TRIT1 | 0.51253 | 4.249864 | 9.2428 | 8.04E-19 | 1.60E-17 | 31.908 |
| EVC2 | 0.51289 | 0.674739 | 8.02506 | 7.99E-15 | 1.02E-13 | 22.8564 |
| AL671883.2 | 0.51305 | 1.672612 | 8.81092 | 2.33E-17 | 3.96E-16 | 28.5921 |
| AL133415.1 | 0.51323 | 0.793378 | 8.15779 | 3.07E-15 | 4.08E-14 | 23.7971 |
| DCBLD2 | 0.51328 | 3.004766 | 9.9482 | 2.61E-21 | 7.16E-20 | 37.5565 |
| PSTPIP2 | 0.51334 | 1.401495 | 8.94311 | 8.43E-18 | 1.51E-16 | 29.5951 |
| MTTP | 0.51349 | 1.59388 | 6.15819 | 1.57E-09 | 1.10E-08 | 10.9408 |
| EYA3 | 0.51351 | 3.459639 | 11.8451 | 1.52E-28 | 1.14E-26 | 54.0124 |
| TMSB4XP1 | 0.51356 | 1.614382 | 7.26583 | 1.53E-12 | 1.52E-11 | 17.7061 |
| TMEM230 | 0.5136 | 6.356478 | 14.2138 | 1.81E-38 | 8.80E-36 | 76.6213 |
| AC010186.2 | 0.51365 | 2.209438 | 11.4044 | 8.44E-27 | 4.89E-25 | 50.0395 |
| FZD6 | 0.51371 | 2.283594 | 6.70029 | 5.89E-11 | 4.91E-10 | 14.1366 |
| DDX39A | 0.51377 | 5.494411 | 9.58991 | 4.96E-20 | 1.15E-18 | 34.6525 |
| ATP11A | 0.51389 | 3.899761 | 8.8293 | 2.03E-17 | 3.47E-16 | 28.7309 |
| AL512329.2 | 0.51399 | 1.594955 | 4.74085 | 2.82E-06 | 1.29E-05 | 3.70064 |
| C1QTNF6 | 0.51406 | 2.315082 | 9.46425 | 1.37E-19 | 3.01E-18 | 33.6509 |
| IL4R | 0.51421 | 3.283207 | 8.02956 | 7.74E-15 | 9.86E-14 | 22.8881 |
| TBC1D2B | 0.51426 | 3.578139 | 10.4006 | 5.75E-23 | 1.95E-21 | 41.3216 |
| OSM | 0.51455 | 1.62607 | 4.87038 | 1.52E-06 | 7.25E-06 | 4.29246 |
| LTBP2 | 0.51473 | 2.20801 | 8.00485 | 9.24E-15 | 1.17E-13 | 22.7142 |
| LINC02488 | 0.51475 | 1.21056 | 4.39092 | 1.39E-05 | 5.80E-05 | 2.17477 |
| TIFA | 0.51492 | 1.767725 | 8.93951 | 8.66E-18 | 1.55E-16 | 29.5676 |
| SOX5 | 0.5151 | 3.141464 | 5.43755 | 8.65E-08 | 4.88E-07 | 7.05191 |
| GDPD2 | 0.51522 | 3.568236 | 4.88444 | 1.42E-06 | 6.81E-06 | 4.35755 |
| AC007344.1 | 0.5153 | 0.62686 | 7.99181 | 1.01E-14 | 1.28E-13 | 22.6226 |
| DIAPH3 | 0.51546 | 1.226519 | 9.7263 | 1.63E-20 | 4.01E-19 | 35.7496 |
| AC025171.1 | 0.51547 | 2.340245 | 11.5028 | 3.47E-27 | 2.13E-25 | 50.919 |
| RPS2 | 0.51565 | 9.127467 | 8.67525 | 6.57E-17 | 1.06E-15 | 27.5739 |
| TRRAP | 0.5161 | 4.643015 | 9.90927 | 3.61E-21 | 9.72E-20 | 37.2375 |
| VCAN | 0.51622 | 6.43595 | 5.51729 | 5.66E-08 | 3.27E-07 | 7.46146 |
| ACTR3 | 0.51632 | 5.087721 | 14.2306 | 1.53E-38 | 7.65E-36 | 76.7884 |
| GPSM2 | 0.51652 | 4.505973 | 8.73246 | 4.25E-17 | 7.01E-16 | 28.0019 |
| MTFR1 | 0.51673 | 3.001437 | 14.4932 | 1.09E-39 | 6.95E-37 | 79.4056 |
| PCMTD2 | 0.51675 | 5.89987 | 10.2664 | 1.80E-22 | 5.68E-21 | 40.1938 |
| MMEL1 | 0.51679 | 0.893848 | 6.73042 | 4.88E-11 | 4.10E-10 | 14.3208 |
| NUP85 | 0.51689 | 4.272263 | 12.6844 | 5.79E-32 | 7.35E-30 | 61.7972 |
| RAB13 | 0.51711 | 6.344701 | 8.06454 | 6.02E-15 | 7.78E-14 | 23.135 |
| TRAF4 | 0.51739 | 5.539872 | 6.71253 | 5.46E-11 | 4.57E-10 | 14.2113 |
| SLC30A6 | 0.51753 | 3.751715 | 14.6014 | 3.65E-40 | 2.81E-37 | 80.4902 |
| ZNF790 | 0.51768 | 2.760778 | 12.6385 | 8.97E-32 | 1.09E-29 | 61.3644 |
| MAP3K20 | 0.51772 | 2.681082 | 7.4165 | 5.56E-13 | 5.80E-12 | 18.6962 |
| ANTXR1 | 0.51782 | 6.498368 | 8.51917 | 2.13E-16 | 3.22E-15 | 26.4167 |
| ALOX15B | 0.51783 | 1.790429 | 4.00129 | 7.30E-05 | 0.000272 | 0.60317 |
| CARD9 | 0.51787 | 1.953083 | 8.47789 | 2.90E-16 | 4.32E-15 | 26.1132 |
| SOX2 | 0.51805 | 9.102636 | 7.32336 | 1.04E-12 | 1.05E-11 | 18.0822 |
| RIOX2 | 0.51811 | 3.067066 | 6.92841 | 1.39E-11 | 1.24E-10 | 15.5481 |
| AL513477.1 | 0.51819 | 1.934912 | 9.13296 | 1.91E-18 | 3.64E-17 | 31.0541 |
| PLB1 | 0.51831 | 1.510126 | 9.66104 | 2.78E-20 | 6.65E-19 | 35.2234 |
| ZNF649 | 0.51833 | 3.762488 | 8.41518 | 4.63E-16 | 6.72E-15 | 25.6543 |
| SAT1 | 0.51843 | 7.538879 | 8.86581 | 1.53E-17 | 2.66E-16 | 29.0073 |
| ZNF90 | 0.51847 | 1.203203 | 11.8206 | 1.90E-28 | 1.41E-26 | 53.7888 |
| AVIL | 0.51901 | 2.615679 | 7.81219 | 3.63E-14 | 4.31E-13 | 21.3723 |
| INPPL1 | 0.51916 | 6.135938 | 10.3212 | 1.13E-22 | 3.67E-21 | 40.6528 |
| MASTL | 0.51932 | 2.897278 | 9.99503 | 1.77E-21 | 4.96E-20 | 37.9412 |
| HLA-J | 0.5194 | 1.791682 | 6.68966 | 6.30E-11 | 5.23E-10 | 14.0717 |
| ZNF700 | 0.51951 | 3.064272 | 12.9192 | 6.11E-33 | 9.05E-31 | 64.0222 |
| HMGN4 | 0.51957 | 6.593395 | 12.3036 | 2.13E-30 | 2.03E-28 | 58.2308 |
| C1orf109 | 0.51959 | 3.875475 | 11.1726 | 6.75E-26 | 3.42E-24 | 47.9846 |
| CYREN | 0.51961 | 3.844569 | 13.0449 | 1.82E-33 | 2.93E-31 | 65.2219 |
| JAK3 | 0.51966 | 1.68168 | 6.30571 | 6.56E-10 | 4.83E-09 | 11.788 |
| AC138207.8 | 0.51979 | 2.181764 | 6.43677 | 2.98E-10 | 2.29E-09 | 12.5548 |
| POT1 | 0.51993 | 4.159722 | 11.6291 | 1.10E-27 | 7.32E-26 | 52.0544 |
| AKAP13 | 0.52001 | 4.120073 | 10.7591 | 2.60E-24 | 1.06E-22 | 44.3794 |
| ALDH16A1 | 0.52005 | 3.647529 | 9.58762 | 5.06E-20 | 1.17E-18 | 34.6341 |
| CA13 | 0.52005 | 1.565111 | 10.089 | 8.05E-22 | 2.34E-20 | 38.717 |
| PLEKHA2 | 0.52023 | 4.79462 | 10.8607 | 1.07E-24 | 4.62E-23 | 45.2583 |
| RNU6-510P | 0.52026 | 2.648982 | 7.60168 | 1.57E-13 | 1.74E-12 | 19.935 |
| HLCS | 0.52048 | 3.214996 | 11.796 | 2.39E-28 | 1.74E-26 | 53.565 |
| RELB | 0.52057 | 3.069168 | 8.09102 | 4.97E-15 | 6.48E-14 | 23.3224 |
| ABCC4 | 0.52064 | 3.248333 | 9.05758 | 3.46E-18 | 6.42E-17 | 30.4722 |
| PIGN | 0.52079 | 3.393497 | 13.1899 | 4.46E-34 | 8.64E-32 | 66.6127 |
| AC004943.2 | 0.52081 | 1.750893 | 13.6118 | 7.20E-36 | 1.92E-33 | 70.6971 |
| CBFB | 0.5209 | 5.194993 | 10.4406 | 4.08E-23 | 1.41E-21 | 41.6595 |
| FES | 0.5209 | 2.887857 | 8.57111 | 1.44E-16 | 2.23E-15 | 26.8001 |
| MMP16 | 0.52128 | 4.554395 | 5.41689 | 9.65E-08 | 5.41E-07 | 6.94666 |
| RO60 | 0.52134 | 4.470852 | 11.4027 | 8.57E-27 | 4.96E-25 | 50.0247 |
| CTNNA1 | 0.52136 | 6.723984 | 12.141 | 9.76E-30 | 8.60E-28 | 56.7252 |
| ZNF227 | 0.52137 | 3.60918 | 12.2728 | 2.85E-30 | 2.68E-28 | 57.9446 |
| TMEM248 | 0.52147 | 6.344694 | 13.0199 | 2.32E-33 | 3.65E-31 | 64.9828 |
| RNF19A | 0.52149 | 5.823264 | 6.54317 | 1.56E-10 | 1.24E-09 | 13.1871 |
| YTHDF2 | 0.5217 | 5.517824 | 12.8556 | 1.13E-32 | 1.63E-30 | 63.4181 |
| CEBPG | 0.52176 | 5.21131 | 10.3881 | 6.40E-23 | 2.15E-21 | 41.2166 |
| TTC7A | 0.5219 | 4.120068 | 10.5544 | 1.53E-23 | 5.57E-22 | 42.6262 |
| ZNF43 | 0.52196 | 3.390362 | 10.13 | 5.71E-22 | 1.69E-20 | 39.0568 |
| AL109918.1 | 0.52196 | 3.472043 | 7.70077 | 7.90E-14 | 9.05E-13 | 20.6078 |
| HEATR5A | 0.52197 | 3.995767 | 8.78357 | 2.88E-17 | 4.83E-16 | 28.3859 |
| FGD3 | 0.52203 | 2.730263 | 7.84311 | 2.92E-14 | 3.50E-13 | 21.586 |
| CCND1 | 0.52217 | 5.947241 | 4.98087 | 8.87E-07 | 4.39E-06 | 4.80866 |
| KDELR3 | 0.52228 | 1.531569 | 7.26613 | 1.53E-12 | 1.52E-11 | 17.708 |
| PMP2 | 0.5223 | 9.656857 | 5.81787 | 1.10E-08 | 6.90E-08 | 9.05206 |
| ITGAV | 0.5223 | 6.539267 | 8.18834 | 2.45E-15 | 3.29E-14 | 24.0152 |
| AC018638.8 | 0.52246 | 3.002592 | 5.92217 | 6.09E-09 | 3.97E-08 | 9.62115 |
| RPS2P32 | 0.52265 | 1.300874 | 6.61848 | 9.80E-11 | 7.96E-10 | 13.6399 |
| PLAGL2 | 0.52282 | 3.560103 | 12.2311 | 4.21E-30 | 3.88E-28 | 57.558 |
| CYB5RL | 0.523 | 1.587803 | 12.4389 | 5.95E-31 | 6.37E-29 | 59.4918 |
| ZNF551 | 0.52301 | 2.611353 | 10.6904 | 4.72E-24 | 1.85E-22 | 43.7888 |
| H2BC9 | 0.52313 | 0.428025 | 8.51203 | 2.25E-16 | 3.39E-15 | 26.3641 |
| NFATC3 | 0.52321 | 2.595037 | 12.4584 | 4.95E-31 | 5.35E-29 | 59.6738 |
| UBTD2 | 0.52327 | 4.949714 | 11.8084 | 2.13E-28 | 1.56E-26 | 53.6779 |
| PSME4 | 0.52332 | 4.086704 | 13.5786 | 9.98E-36 | 2.63E-33 | 70.3734 |
| AL365357.1 | 0.5234 | 2.532833 | 2.40637 | 0.01649 | 0.038698 | -4.3775 |
| RHPN1-AS1 | 0.52361 | 1.316743 | 11.1842 | 6.09E-26 | 3.10E-24 | 48.0862 |
| ABCB7 | 0.52362 | 4.526517 | 13.4622 | 3.13E-35 | 7.38E-33 | 69.2424 |
| FGD2 | 0.52366 | 2.253617 | 7.0834 | 5.09E-12 | 4.79E-11 | 16.529 |
| ZDHHC15 | 0.52377 | 3.018763 | 11.2265 | 4.18E-26 | 2.18E-24 | 48.4598 |
| DENND3 | 0.52378 | 3.034448 | 7.96362 | 1.24E-14 | 1.55E-13 | 22.4249 |
| LINC01831 | 0.52392 | 0.761077 | 7.43529 | 4.90E-13 | 5.15E-12 | 18.8208 |
| HM13 | 0.52404 | 5.183552 | 13.2975 | 1.57E-34 | 3.28E-32 | 67.6483 |
| NFKB1 | 0.52409 | 3.894062 | 11.5997 | 1.44E-27 | 9.35E-26 | 51.7892 |
| SYNGR2 | 0.52422 | 5.031309 | 7.15171 | 3.25E-12 | 3.13E-11 | 16.9669 |
| LZIC | 0.5243 | 5.039261 | 10.2732 | 1.70E-22 | 5.38E-21 | 40.2509 |
| RASGRP4 | 0.52432 | 1.814509 | 8.19736 | 2.30E-15 | 3.10E-14 | 24.0798 |
| UBE2S | 0.52454 | 4.034526 | 7.78747 | 4.31E-14 | 5.08E-13 | 21.202 |
| EVI5 | 0.5248 | 3.725211 | 11.3792 | 1.06E-26 | 6.00E-25 | 49.8144 |
| RNF149 | 0.52506 | 3.483892 | 8.23186 | 1.79E-15 | 2.44E-14 | 24.3271 |
| B3GLCT | 0.52519 | 4.2931 | 7.80578 | 3.79E-14 | 4.50E-13 | 21.3281 |
| ZNF850 | 0.52526 | 1.04676 | 13.6159 | 6.91E-36 | 1.85E-33 | 70.7374 |
| ERAP2 | 0.52527 | 2.869232 | 4.51542 | 7.98E-06 | 3.44E-05 | 2.7054 |
| ACTN4 | 0.52541 | 6.747555 | 10.7236 | 3.54E-24 | 1.41E-22 | 44.0743 |
| TEX261 | 0.52546 | 6.173253 | 13.8001 | 1.12E-36 | 3.47E-34 | 72.5386 |
| M6PR | 0.52552 | 5.444174 | 10.8174 | 1.56E-24 | 6.56E-23 | 44.8831 |
| ABCD3 | 0.52553 | 5.002553 | 11.3694 | 1.16E-26 | 6.52E-25 | 49.728 |
| CMTM1 | 0.52561 | 2.058808 | 9.49898 | 1.04E-19 | 2.31E-18 | 33.9269 |
| TCF3 | 0.52561 | 5.671663 | 9.20952 | 1.05E-18 | 2.06E-17 | 31.6485 |
| MEX3D | 0.52566 | 4.190403 | 9.70993 | 1.87E-20 | 4.55E-19 | 35.6174 |
| TMBIM1 | 0.5257 | 5.221882 | 6.36486 | 4.60E-10 | 3.45E-09 | 12.1325 |
| RHNO1 | 0.52572 | 5.098933 | 11.5833 | 1.67E-27 | 1.07E-25 | 51.6417 |
| CTNNAL1 | 0.52576 | 4.292895 | 9.84711 | 6.04E-21 | 1.58E-19 | 36.73 |
| ZNF229 | 0.5258 | 2.856465 | 8.5932 | 1.22E-16 | 1.90E-15 | 26.9637 |
| TMTC2 | 0.52588 | 3.338662 | 5.74738 | 1.62E-08 | 9.99E-08 | 8.67248 |
| C19orf54 | 0.52591 | 4.155574 | 12.0913 | 1.55E-29 | 1.32E-27 | 56.2673 |
| SOCS1 | 0.52628 | 1.659984 | 6.54945 | 1.50E-10 | 1.20E-09 | 13.2247 |
| ZNF112 | 0.52633 | 2.999544 | 11.3834 | 1.02E-26 | 5.80E-25 | 49.8519 |
| CDH2 | 0.52642 | 6.459143 | 9.98021 | 2.00E-21 | 5.56E-20 | 37.8194 |
| HAT1 | 0.52644 | 4.158946 | 14.2994 | 7.68E-39 | 4.21E-36 | 77.4726 |
| ELOA | 0.5268 | 4.904501 | 13.1253 | 8.35E-34 | 1.49E-31 | 65.9925 |
| PLIN2 | 0.52685 | 3.339826 | 8.50068 | 2.45E-16 | 3.67E-15 | 26.2806 |
| FCMR | 0.527 | 1.60295 | 7.72362 | 6.74E-14 | 7.78E-13 | 20.7639 |
| NUP37 | 0.52713 | 3.159001 | 13.8797 | 5.09E-37 | 1.67E-34 | 73.3205 |
| CCT6A | 0.52735 | 7.085704 | 10.718 | 3.72E-24 | 1.48E-22 | 44.0261 |
| CCNL2 | 0.52755 | 5.500461 | 6.52127 | 1.78E-10 | 1.41E-09 | 13.0563 |
| TC2N | 0.52757 | 1.265399 | 6.31739 | 6.12E-10 | 4.51E-09 | 11.8558 |
| SPHK1 | 0.5277 | 2.411684 | 6.20094 | 1.22E-09 | 8.64E-09 | 11.1846 |
| PGAP6 | 0.52783 | 4.909689 | 12.1586 | 8.29E-30 | 7.38E-28 | 56.8876 |
| TRAC | 0.52784 | 1.391659 | 6.00034 | 3.90E-09 | 2.60E-08 | 10.0533 |
| ALDH3B1 | 0.528 | 2.645878 | 8.79593 | 2.62E-17 | 4.41E-16 | 28.479 |
| AC009318.1 | 0.52815 | 1.270163 | 9.71413 | 1.80E-20 | 4.40E-19 | 35.6513 |
| XKR8 | 0.52835 | 2.523166 | 5.70695 | 2.03E-08 | 1.23E-07 | 8.45657 |
| PLEKHG1 | 0.52837 | 3.317098 | 7.06001 | 5.93E-12 | 5.55E-11 | 16.3798 |
| MORC4 | 0.52847 | 3.179011 | 11.4112 | 7.94E-27 | 4.61E-25 | 50.1002 |
| NXT1 | 0.52851 | 4.984184 | 11.0861 | 1.46E-25 | 7.12E-24 | 47.2232 |
| WDR34 | 0.52879 | 5.590765 | 9.25878 | 7.09E-19 | 1.42E-17 | 32.0328 |
| SCP2 | 0.52914 | 5.387181 | 11.6891 | 6.36E-28 | 4.39E-26 | 52.5959 |
| CCRL2 | 0.52916 | 1.637857 | 9.78112 | 1.04E-20 | 2.63E-19 | 36.1935 |
| SOX4 | 0.52922 | 6.7989 | 4.57981 | 5.95E-06 | 2.61E-05 | 2.98512 |
| TRIB2 | 0.5293 | 6.158377 | 5.87973 | 7.74E-09 | 4.98E-08 | 9.38853 |
| ITPRIP | 0.5293 | 3.188766 | 8.38885 | 5.63E-16 | 8.09E-15 | 25.4623 |
| OSBPL9 | 0.52942 | 4.055325 | 8.61906 | 1.01E-16 | 1.58E-15 | 27.1555 |
| DSE | 0.52954 | 2.190409 | 10.3414 | 9.53E-23 | 3.12E-21 | 40.8229 |
| PLEKHA8P1 | 0.52962 | 1.673887 | 11.3068 | 2.03E-26 | 1.11E-24 | 49.171 |
| DISP1 | 0.52969 | 3.202434 | 9.57341 | 5.68E-20 | 1.31E-18 | 34.5205 |
| H4C8 | 0.5297 | 2.457295 | 6.98504 | 9.64E-12 | 8.79E-11 | 15.9044 |
| TAGAP | 0.52984 | 2.945206 | 6.38821 | 4.00E-10 | 3.02E-09 | 12.2692 |
| ELOCP19 | 0.52996 | 1.835065 | 8.1611 | 2.99E-15 | 3.99E-14 | 23.8207 |
| DNAAF3 | 0.53008 | 0.86839 | 7.52353 | 2.68E-13 | 2.91E-12 | 19.4093 |
| FAM181A-AS1 | 0.53009 | 1.392772 | 5.89099 | 7.27E-09 | 4.69E-08 | 9.45008 |
| AC010186.3 | 0.53012 | 1.630894 | 12.392 | 9.27E-31 | 9.48E-29 | 59.0537 |
| DUSP16 | 0.53015 | 4.705922 | 11.0704 | 1.68E-25 | 8.08E-24 | 47.0857 |
| TRH | 0.53035 | 1.839834 | 3.32946 | 0.00094 | 0.002895 | -1.784 |
| GAB1 | 0.53049 | 4.873443 | 9.41054 | 2.11E-19 | 4.52E-18 | 33.2256 |
| PECAM1 | 0.5307 | 4.448362 | 8.39737 | 5.28E-16 | 7.62E-15 | 25.5244 |
| STAG1 | 0.53084 | 3.867831 | 8.43408 | 4.02E-16 | 5.89E-15 | 25.7923 |
| IGHM | 0.53093 | 1.023224 | 4.7087 | 3.28E-06 | 1.49E-05 | 3.55599 |
| FEM1C | 0.53094 | 4.625948 | 10.3673 | 7.64E-23 | 2.53E-21 | 41.0413 |
| ARHGEF6 | 0.53095 | 6.458866 | 7.82071 | 3.41E-14 | 4.07E-13 | 21.4311 |
| C5orf15 | 0.531 | 5.789885 | 13.066 | 1.48E-33 | 2.46E-31 | 65.4234 |
| ODF3B | 0.53126 | 2.261628 | 5.57326 | 4.19E-08 | 2.46E-07 | 7.75204 |
| OLFML2A | 0.53146 | 2.350513 | 7.08015 | 5.20E-12 | 4.89E-11 | 16.5082 |
| MSH6 | 0.53154 | 3.926624 | 13.2041 | 3.89E-34 | 7.61E-32 | 66.7492 |
| RHOJ | 0.53171 | 3.654066 | 6.68606 | 6.44E-11 | 5.34E-10 | 14.0498 |
| AC110597.1 | 0.53175 | 1.714896 | 8.41784 | 4.54E-16 | 6.60E-15 | 25.6737 |
| AL671277.1 | 0.53183 | 2.811736 | 3.71081 | 0.00023 | 0.000793 | -0.4797 |
| GEN1 | 0.53209 | 1.590542 | 10.5493 | 1.60E-23 | 5.80E-22 | 42.5821 |
| SNAP23 | 0.53211 | 3.977852 | 13.0287 | 2.13E-33 | 3.37E-31 | 65.0668 |
| ATAD5 | 0.53216 | 1.671542 | 10.7869 | 2.04E-24 | 8.42E-23 | 44.6199 |
| MTF2 | 0.53229 | 3.403192 | 11.4151 | 7.67E-27 | 4.48E-25 | 50.1348 |
| GTF2I | 0.53232 | 4.343457 | 7.21581 | 2.13E-12 | 2.08E-11 | 17.3809 |
| AL357500.2 | 0.53241 | 1.775256 | 7.2797 | 1.39E-12 | 1.39E-11 | 17.7965 |
| COL9A3 | 0.5326 | 3.529496 | 3.79913 | 0.00016 | 0.000577 | -0.1585 |
| ABCA1 | 0.53265 | 4.991638 | 6.36521 | 4.59E-10 | 3.44E-09 | 12.1345 |
| SMO | 0.53269 | 4.720463 | 7.56319 | 2.05E-13 | 2.24E-12 | 19.6756 |
| NOP2 | 0.53275 | 4.568889 | 11.4172 | 7.52E-27 | 4.40E-25 | 50.1539 |
| SOWAHD | 0.53293 | 1.390302 | 8.63156 | 9.15E-17 | 1.44E-15 | 27.2484 |
| EPHA2 | 0.53307 | 2.474595 | 6.1271 | 1.88E-09 | 1.30E-08 | 10.7645 |
| CPED1 | 0.53317 | 1.465673 | 8.40259 | 5.08E-16 | 7.34E-15 | 25.5624 |
| ARID1A | 0.53327 | 5.497794 | 10.4331 | 4.35E-23 | 1.49E-21 | 41.596 |
| VAT1 | 0.53335 | 7.190179 | 10.6758 | 5.36E-24 | 2.08E-22 | 43.6635 |
| FRMD4B | 0.53358 | 2.614528 | 7.57442 | 1.89E-13 | 2.08E-12 | 19.7512 |
| CD151 | 0.53358 | 6.000616 | 7.89551 | 2.01E-14 | 2.46E-13 | 21.9495 |
| DUSP6 | 0.53363 | 4.314707 | 5.42812 | 9.09E-08 | 5.11E-07 | 7.00381 |
| RNA5SP82 | 0.53365 | 1.556727 | 6.7409 | 4.57E-11 | 3.85E-10 | 14.385 |
| CENPO | 0.53397 | 3.332151 | 10.5866 | 1.16E-23 | 4.31E-22 | 42.9003 |
| AF001548.2 | 0.534 | 3.22114 | 7.46883 | 3.90E-13 | 4.15E-12 | 19.0438 |
| HEATR3 | 0.53433 | 3.881673 | 13.2526 | 2.42E-34 | 4.87E-32 | 67.216 |
| ZNF347 | 0.53437 | 2.406626 | 12.6741 | 6.39E-32 | 8.00E-30 | 61.6997 |
| IGLC2 | 0.53437 | 1.219419 | 3.64252 | 0.0003 | 0.001011 | -0.7231 |
| ZNF616 | 0.53442 | 2.880118 | 13.0695 | 1.43E-33 | 2.40E-31 | 65.4574 |
| LRGUK | 0.53442 | 0.738928 | 10.06 | 1.03E-21 | 2.95E-20 | 38.4766 |
| FUT4 | 0.53445 | 1.716476 | 9.11992 | 2.12E-18 | 4.02E-17 | 30.9531 |
| GPX1P1 | 0.53446 | 2.29359 | 4.6266 | 4.80E-06 | 2.14E-05 | 3.19069 |
| MBOAT1 | 0.53448 | 1.300449 | 8.67928 | 6.37E-17 | 1.03E-15 | 27.604 |
| AL445307.1 | 0.53455 | 2.169182 | 7.79725 | 4.03E-14 | 4.77E-13 | 21.2693 |
| FYCO1 | 0.53513 | 3.126472 | 10.726 | 3.47E-24 | 1.38E-22 | 44.0945 |
| TRIM45 | 0.53516 | 2.553367 | 9.99229 | 1.81E-21 | 5.05E-20 | 37.9187 |
| FGFBP2 | 0.5352 | 1.574198 | 4.92139 | 1.19E-06 | 5.76E-06 | 4.52948 |
| MAP3K14 | 0.53521 | 3.456007 | 10.1769 | 3.85E-22 | 1.17E-20 | 39.4462 |
| VMP1 | 0.53528 | 4.912109 | 10.8363 | 1.32E-24 | 5.61E-23 | 45.0469 |
| DHX15 | 0.53544 | 5.160576 | 13.3366 | 1.07E-34 | 2.32E-32 | 68.0266 |
| COL18A1 | 0.53548 | 4.224764 | 6.9508 | 1.20E-11 | 1.08E-10 | 15.6887 |
| SELENOP | 0.53553 | 5.139565 | 5.16115 | 3.61E-07 | 1.88E-06 | 5.67323 |
| MEIS1 | 0.53554 | 2.277742 | 8.67515 | 6.58E-17 | 1.06E-15 | 27.5731 |
| PTTG1IP | 0.53559 | 8.698963 | 9.62626 | 3.69E-20 | 8.70E-19 | 34.9438 |
| TULP3 | 0.53561 | 4.646133 | 10.3118 | 1.23E-22 | 3.95E-21 | 40.5747 |
| TREM1 | 0.53563 | 0.662218 | 7.397 | 6.34E-13 | 6.56E-12 | 18.5671 |
| PVT1 | 0.53569 | 0.95535 | 9.30197 | 5.03E-19 | 1.02E-17 | 32.3709 |
| IL16 | 0.53584 | 1.586148 | 9.28315 | 5.84E-19 | 1.18E-17 | 32.2235 |
| GLB1 | 0.53585 | 4.693388 | 13.1824 | 4.80E-34 | 9.16E-32 | 66.5404 |
| ZNRF2 | 0.53616 | 2.983896 | 11.0895 | 1.42E-25 | 6.92E-24 | 47.2537 |
| TP73 | 0.53642 | 1.052887 | 6.97301 | 1.04E-11 | 9.45E-11 | 15.8285 |
| CDC42 | 0.53643 | 6.984261 | 12.5679 | 1.76E-31 | 2.04E-29 | 60.7004 |
| CD3E | 0.53652 | 0.883391 | 7.39323 | 6.51E-13 | 6.73E-12 | 18.5422 |
| LINC01116 | 0.53657 | 1.634585 | 4.84329 | 1.73E-06 | 8.20E-06 | 4.16747 |
| MGA | 0.53692 | 3.050817 | 11.2762 | 2.68E-26 | 1.43E-24 | 48.8995 |
| SMCO4 | 0.53713 | 3.901359 | 8.91426 | 1.05E-17 | 1.87E-16 | 29.3753 |
| MIR5581 | 0.53733 | 2.511876 | 5.37544 | 1.20E-07 | 6.64E-07 | 6.73653 |
| PLXNA3 | 0.53734 | 4.126922 | 9.84429 | 6.18E-21 | 1.61E-19 | 36.707 |
| TRIO | 0.53735 | 4.858342 | 7.59821 | 1.61E-13 | 1.78E-12 | 19.9116 |
| NOL11 | 0.53744 | 4.55168 | 12.9217 | 5.97E-33 | 8.86E-31 | 64.0459 |
| ZNF776 | 0.53764 | 3.51066 | 10.748 | 2.86E-24 | 1.16E-22 | 44.284 |
| F2RL2 | 0.53776 | 0.770735 | 5.80884 | 1.15E-08 | 7.24E-08 | 9.00324 |
| AC004067.1 | 0.53778 | 1.391622 | 7.57097 | 1.94E-13 | 2.13E-12 | 19.728 |
| CANT1 | 0.53792 | 4.876805 | 12.7027 | 4.86E-32 | 6.26E-30 | 61.9702 |
| AL049839.2 | 0.538 | 1.162036 | 6.22143 | 1.08E-09 | 7.72E-09 | 11.3019 |
| LINC01778 | 0.5381 | 1.999462 | 8.16598 | 2.89E-15 | 3.85E-14 | 23.8556 |
| BTBD19 | 0.53823 | 1.972964 | 8.21618 | 2.00E-15 | 2.72E-14 | 24.2146 |
| YAP1 | 0.5383 | 4.301924 | 5.64104 | 2.90E-08 | 1.73E-07 | 8.10742 |
| LINC01956 | 0.53836 | 0.782824 | 6.37029 | 4.46E-10 | 3.35E-09 | 12.1642 |
| SASS6 | 0.53836 | 2.223504 | 13.1564 | 6.18E-34 | 1.13E-31 | 66.2905 |
| TMED10 | 0.53848 | 7.398809 | 12.677 | 6.22E-32 | 7.83E-30 | 61.7272 |
| PDLIM4 | 0.53855 | 2.40714 | 3.9348 | 9.57E-05 | 0.000349 | 0.34855 |
| STX11 | 0.53865 | 1.657998 | 7.54797 | 2.27E-13 | 2.48E-12 | 19.5733 |
| TFDP2 | 0.5387 | 4.1791 | 10.5137 | 2.18E-23 | 7.73E-22 | 42.2794 |
| PUS7 | 0.53886 | 3.169531 | 10.5526 | 1.56E-23 | 5.65E-22 | 42.6109 |
| HP1BP3 | 0.53908 | 6.498644 | 11.3755 | 1.10E-26 | 6.19E-25 | 49.7816 |
| SIPA1L3 | 0.53911 | 3.461086 | 10.2141 | 2.81E-22 | 8.69E-21 | 39.7562 |
| BBS9 | 0.53917 | 3.601872 | 13.66 | 4.47E-36 | 1.27E-33 | 71.1681 |
| MAP2K3 | 0.53943 | 3.706803 | 9.77476 | 1.10E-20 | 2.76E-19 | 36.1419 |
| HEXB | 0.53962 | 4.625924 | 9.91046 | 3.57E-21 | 9.63E-20 | 37.2473 |
| ZBED6 | 0.53965 | 2.132492 | 5.8341 | 1.00E-08 | 6.34E-08 | 9.14008 |
| PLCG2 | 0.53983 | 1.799749 | 9.99154 | 1.82E-21 | 5.08E-20 | 37.9125 |
| NCF1C | 0.53991 | 1.439643 | 8.34357 | 7.87E-16 | 1.11E-14 | 25.1333 |
| INAVA | 0.54011 | 2.31993 | 6.43463 | 3.02E-10 | 2.31E-09 | 12.5422 |
| CHPF2 | 0.54026 | 4.951145 | 10.7157 | 3.79E-24 | 1.51E-22 | 44.0058 |
| MYZAP | 0.54096 | 0.878083 | 7.26577 | 1.53E-12 | 1.52E-11 | 17.7057 |
| ASIP | 0.54097 | 1.719041 | 7.02193 | 7.59E-12 | 7.02E-11 | 16.1378 |
| AGRN | 0.541 | 5.940864 | 8.58833 | 1.27E-16 | 1.97E-15 | 26.9275 |
| SMARCE1 | 0.54115 | 4.186196 | 15.9922 | 2.21E-46 | 1.21E-42 | 94.6706 |
| CHRNA9 | 0.5412 | 0.546862 | 6.4679 | 2.47E-10 | 1.91E-09 | 12.7389 |
| DNAJC22 | 0.54138 | 1.394118 | 8.23485 | 1.75E-15 | 2.39E-14 | 24.3486 |
| GLI2 | 0.54141 | 2.0734 | 7.58762 | 1.73E-13 | 1.91E-12 | 19.8401 |
| ARRDC3 | 0.54149 | 5.078979 | 9.57857 | 5.44E-20 | 1.26E-18 | 34.5617 |
| SRBD1 | 0.54154 | 3.651118 | 13.1055 | 1.01E-33 | 1.78E-31 | 65.802 |
| DENND2A | 0.5416 | 5.636184 | 7.13976 | 3.52E-12 | 3.37E-11 | 16.89 |
| GNA13 | 0.54162 | 6.077595 | 10.834 | 1.35E-24 | 5.72E-23 | 45.0267 |
| SYNC | 0.54173 | 1.799665 | 6.70626 | 5.68E-11 | 4.74E-10 | 14.173 |
| COLEC12 | 0.54176 | 3.11141 | 5.69686 | 2.14E-08 | 1.30E-07 | 8.40287 |
| ZIC1 | 0.54202 | 3.719695 | 5.33794 | 1.46E-07 | 7.98E-07 | 6.54768 |
| COL4A6 | 0.54218 | 1.231671 | 7.02705 | 7.35E-12 | 6.80E-11 | 16.1703 |
| COTL1 | 0.54246 | 6.125786 | 8.23522 | 1.74E-15 | 2.38E-14 | 24.3512 |
| NEAT1 | 0.54259 | 3.80694 | 5.47649 | 7.04E-08 | 4.01E-07 | 7.25123 |
| HOXC9 | 0.54276 | 0.322253 | 7.76173 | 5.17E-14 | 6.04E-13 | 21.025 |
| TREML1 | 0.5428 | 1.982493 | 6.939 | 1.30E-11 | 1.16E-10 | 15.6145 |
| HOXA1 | 0.54283 | 0.416521 | 9.47232 | 1.29E-19 | 2.83E-18 | 33.715 |
| POLA2 | 0.54284 | 3.155265 | 12.4274 | 6.64E-31 | 7.05E-29 | 59.3843 |
| ESM1 | 0.54321 | 0.787114 | 5.17091 | 3.44E-07 | 1.80E-06 | 5.72083 |
| GSX2 | 0.54323 | 0.727149 | 6.02262 | 3.44E-09 | 2.31E-08 | 10.1774 |
| CCDC138 | 0.54334 | 1.904698 | 13.2875 | 1.73E-34 | 3.57E-32 | 67.5523 |
| SF3A3 | 0.54339 | 5.579614 | 13.8668 | 5.78E-37 | 1.89E-34 | 73.1938 |
| KLF10 | 0.544 | 4.277097 | 6.3981 | 3.77E-10 | 2.85E-09 | 12.3272 |
| CMTR2 | 0.54412 | 3.242649 | 13.8154 | 9.63E-37 | 3.03E-34 | 72.689 |
| ALPK3 | 0.54427 | 1.90884 | 8.1138 | 4.22E-15 | 5.53E-14 | 23.4841 |
| VGLL4 | 0.54429 | 4.520241 | 9.37238 | 2.87E-19 | 6.04E-18 | 32.9244 |
| TMEM131L | 0.54434 | 2.731958 | 9.40176 | 2.27E-19 | 4.83E-18 | 33.1563 |
| OTP | 0.54438 | 0.316043 | 7.42005 | 5.43E-13 | 5.67E-12 | 18.7197 |
| H2BC21 | 0.54447 | 5.009784 | 6.86838 | 2.04E-11 | 1.79E-10 | 15.1728 |
| SELPLG | 0.54452 | 5.624435 | 5.39128 | 1.10E-07 | 6.15E-07 | 6.81664 |
| APOC1 | 0.54457 | 7.57097 | 5.46312 | 7.56E-08 | 4.29E-07 | 7.18264 |
| SIX6 | 0.54463 | 0.386386 | 6.81752 | 2.82E-11 | 2.43E-10 | 14.8571 |
| URB2 | 0.54468 | 3.292589 | 12.2625 | 3.13E-30 | 2.93E-28 | 57.8493 |
| SERPINF1 | 0.54472 | 4.196058 | 4.93812 | 1.09E-06 | 5.33E-06 | 4.60767 |
| MIR3176 | 0.54503 | 2.089692 | 5.86936 | 8.21E-09 | 5.26E-08 | 9.33193 |
| ZNF474 | 0.54529 | 0.641237 | 8.37733 | 6.13E-16 | 8.79E-15 | 25.3785 |
| ZFP36L1 | 0.5453 | 6.935903 | 7.24648 | 1.74E-12 | 1.72E-11 | 17.58 |
| TES | 0.54539 | 1.777677 | 9.23845 | 8.33E-19 | 1.66E-17 | 31.8741 |
| RBBP4 | 0.54554 | 5.858106 | 12.69 | 5.49E-32 | 7.00E-30 | 61.8502 |
| PGD | 0.54564 | 6.982394 | 12.7632 | 2.73E-32 | 3.72E-30 | 62.5421 |
| HMGN2P5 | 0.54598 | 2.462366 | 7.8159 | 3.53E-14 | 4.21E-13 | 21.3979 |
| ROCK1 | 0.54604 | 4.002175 | 9.73312 | 1.54E-20 | 3.81E-19 | 35.8047 |
| HNRNPCP1 | 0.54625 | 1.647066 | 10.6104 | 9.45E-24 | 3.55E-22 | 43.1038 |
| PILRA | 0.54641 | 3.588142 | 8.29145 | 1.15E-15 | 1.60E-14 | 24.7562 |
| GYPC | 0.54647 | 4.019237 | 6.16219 | 1.53E-09 | 1.07E-08 | 10.9636 |
| HOXD4 | 0.54669 | 0.707283 | 7.2542 | 1.65E-12 | 1.64E-11 | 17.6303 |
| SPSB1 | 0.54681 | 4.776886 | 7.99713 | 9.76E-15 | 1.23E-13 | 22.66 |
| TMEM176A | 0.54682 | 3.99336 | 4.8784 | 1.46E-06 | 6.99E-06 | 4.32955 |
| SRSF10 | 0.54684 | 4.472654 | 14.033 | 1.11E-37 | 4.27E-35 | 74.8312 |
| MDFIC | 0.54687 | 2.998908 | 5.87179 | 8.10E-09 | 5.19E-08 | 9.34515 |
| BTG3-AS1 | 0.54692 | 1.125034 | 11.0216 | 2.58E-25 | 1.21E-23 | 46.6587 |
| EYA4 | 0.54693 | 0.687486 | 6.36425 | 4.62E-10 | 3.46E-09 | 12.1289 |
| ZNF69 | 0.54695 | 2.229581 | 11.8988 | 9.25E-29 | 7.19E-27 | 54.5021 |
| HOXA2 | 0.54699 | 0.372707 | 6.17665 | 1.41E-09 | 9.91E-09 | 11.0459 |
| PNRC2 | 0.54699 | 5.635649 | 12.8499 | 1.19E-32 | 1.71E-30 | 63.3637 |
| NOTCH3 | 0.54702 | 4.65792 | 7.89191 | 2.06E-14 | 2.52E-13 | 21.9245 |
| TRIM56 | 0.54715 | 4.02643 | 8.16517 | 2.91E-15 | 3.88E-14 | 23.8498 |
| ERF | 0.54723 | 5.932481 | 9.93696 | 2.87E-21 | 7.81E-20 | 37.4643 |
| SCLT1 | 0.54724 | 2.026347 | 14.3911 | 3.05E-39 | 1.76E-36 | 78.386 |
| ZNF761 | 0.54732 | 2.715029 | 12.6147 | 1.12E-31 | 1.35E-29 | 61.1407 |
| ATG4C | 0.54733 | 4.286298 | 10.9365 | 5.48E-25 | 2.49E-23 | 45.916 |
| PAXIP1-AS2 | 0.54767 | 2.401116 | 7.28676 | 1.33E-12 | 1.33E-11 | 17.8426 |
| GPC3 | 0.54771 | 1.546523 | 5.50261 | 6.12E-08 | 3.52E-07 | 7.38567 |
| NCF1 | 0.54785 | 1.282392 | 9.23594 | 8.49E-19 | 1.69E-17 | 31.8544 |
| RNF213 | 0.54817 | 3.956571 | 10.1919 | 3.39E-22 | 1.04E-20 | 39.5715 |
| FUCA1 | 0.54843 | 4.606944 | 9.169 | 1.44E-18 | 2.79E-17 | 31.3334 |
| SLC25A24 | 0.54845 | 1.732695 | 9.12013 | 2.12E-18 | 4.01E-17 | 30.9548 |
| PTBP3 | 0.54848 | 3.547906 | 11.4095 | 8.06E-27 | 4.68E-25 | 50.0848 |
| CAST | 0.54855 | 4.122199 | 11.0739 | 1.63E-25 | 7.87E-24 | 47.1162 |
| ADPGK | 0.5486 | 4.155954 | 14.9532 | 1.02E-41 | 1.33E-38 | 84.0351 |
| THBS1 | 0.54861 | 1.945703 | 4.63625 | 4.59E-06 | 2.05E-05 | 3.23334 |
| TNFAIP2 | 0.54879 | 3.468932 | 6.23141 | 1.02E-09 | 7.31E-09 | 11.3592 |
| VEGFA | 0.54881 | 3.217954 | 5.73265 | 1.76E-08 | 1.08E-07 | 8.59362 |
| GALM | 0.54923 | 3.22416 | 8.05216 | 6.58E-15 | 8.46E-14 | 23.0475 |
| LRRC8D | 0.54923 | 5.112083 | 11.4141 | 7.73E-27 | 4.51E-25 | 50.1262 |
| GALNT7 | 0.54925 | 3.26922 | 10.4288 | 4.51E-23 | 1.54E-21 | 41.5603 |
| RPS6KA3 | 0.5493 | 3.676374 | 10.3967 | 5.94E-23 | 2.00E-21 | 41.2889 |
| GSAP | 0.54936 | 1.968418 | 7.58378 | 1.78E-13 | 1.96E-12 | 19.8142 |
| ZSCAN16 | 0.5494 | 3.441138 | 12.2634 | 3.11E-30 | 2.91E-28 | 57.8577 |
| PABPC4 | 0.54957 | 5.43174 | 13.8797 | 5.09E-37 | 1.67E-34 | 73.3202 |
| CBX3 | 0.54978 | 6.764164 | 13.8845 | 4.85E-37 | 1.61E-34 | 73.3675 |
| TRMT13 | 0.54992 | 3.337658 | 11.3242 | 1.74E-26 | 9.64E-25 | 49.3256 |
| EPS8 | 0.55001 | 4.294306 | 10.2735 | 1.70E-22 | 5.37E-21 | 40.2533 |
| ITGA4 | 0.55003 | 1.068847 | 8.68765 | 5.98E-17 | 9.66E-16 | 27.6665 |
| SRSF11 | 0.55038 | 5.65653 | 10.5372 | 1.78E-23 | 6.41E-22 | 42.4791 |
| SUSD6 | 0.5506 | 5.199901 | 10.334 | 1.01E-22 | 3.31E-21 | 40.7608 |
| ZNF845 | 0.55103 | 2.21024 | 13.7367 | 2.10E-36 | 6.35E-34 | 71.918 |
| DAP | 0.55156 | 6.190629 | 12.3876 | 9.67E-31 | 9.81E-29 | 59.0127 |
| TMEM43 | 0.55179 | 6.054438 | 13.4135 | 5.04E-35 | 1.14E-32 | 68.7702 |
| SEPSECS | 0.55179 | 2.977814 | 13.6929 | 3.23E-36 | 9.53E-34 | 71.4891 |
| HOXD11 | 0.55195 | 0.316366 | 7.44878 | 4.47E-13 | 4.72E-12 | 18.9104 |
| ECE1 | 0.55244 | 5.271176 | 8.35278 | 7.35E-16 | 1.04E-14 | 25.2001 |
| TMEM52B | 0.55258 | 1.624526 | 7.91267 | 1.78E-14 | 2.19E-13 | 22.069 |
| EDEM2 | 0.55267 | 4.944173 | 13.824 | 8.84E-37 | 2.82E-34 | 72.7735 |
| MX1 | 0.55276 | 3.704997 | 5.27102 | 2.06E-07 | 1.11E-06 | 6.21359 |
| EBI3 | 0.55296 | 3.782449 | 6.3418 | 5.29E-10 | 3.93E-09 | 11.9978 |
| STK38L | 0.55297 | 4.64579 | 9.08868 | 2.71E-18 | 5.09E-17 | 30.7119 |
| FIGNL1 | 0.55305 | 3.188704 | 11.3794 | 1.06E-26 | 6.00E-25 | 49.8163 |
| H1-2 | 0.55309 | 5.297692 | 5.88054 | 7.71E-09 | 4.96E-08 | 9.39297 |
| PRXL2C | 0.5532 | 3.50779 | 14.291 | 8.36E-39 | 4.54E-36 | 77.3886 |
| RNPEP | 0.55326 | 5.069206 | 13.3272 | 1.17E-34 | 2.54E-32 | 67.935 |
| INTS13 | 0.55363 | 4.489809 | 13.5456 | 1.38E-35 | 3.50E-33 | 70.0524 |
| NUP107 | 0.55377 | 3.494366 | 11.7968 | 2.37E-28 | 1.72E-26 | 53.5729 |
| CEP89 | 0.5538 | 3.263055 | 12.2548 | 3.37E-30 | 3.13E-28 | 57.7777 |
| CARHSP1 | 0.5538 | 4.766089 | 9.43731 | 1.70E-19 | 3.68E-18 | 33.4374 |
| PTPN12 | 0.55392 | 5.235816 | 14.1609 | 3.08E-38 | 1.40E-35 | 76.0961 |
| LFNG | 0.554 | 6.181942 | 5.04257 | 6.54E-07 | 3.30E-06 | 5.10144 |
| SLC25A13 | 0.55415 | 4.386927 | 11.5164 | 3.07E-27 | 1.90E-25 | 51.0408 |
| IGFBP4 | 0.55424 | 5.684422 | 5.76914 | 1.44E-08 | 8.91E-08 | 8.78922 |
| BTN3A3 | 0.55448 | 3.951255 | 7.8994 | 1.96E-14 | 2.39E-13 | 21.9767 |
| BCL10 | 0.5546 | 3.542468 | 14.4665 | 1.43E-39 | 8.60E-37 | 79.1386 |
| PRIMA1 | 0.55463 | 3.464288 | 6.56954 | 1.33E-10 | 1.06E-09 | 13.3452 |
| AL355974.2 | 0.55466 | 2.590888 | 4.17059 | 3.61E-05 | 0.000141 | 1.26939 |
| HLA-E | 0.55469 | 8.6926 | 8.0616 | 6.14E-15 | 7.94E-14 | 23.1142 |
| MYO7A | 0.55478 | 2.166076 | 8.68288 | 6.20E-17 | 1.00E-15 | 27.6308 |
| CNOT1 | 0.55491 | 4.967956 | 10.3313 | 1.04E-22 | 3.37E-21 | 40.7383 |
| ALPK1 | 0.55518 | 1.407501 | 10.3866 | 6.48E-23 | 2.17E-21 | 41.204 |
| ARL13B | 0.55541 | 3.39821 | 12.128 | 1.10E-29 | 9.65E-28 | 56.605 |
| PDIA6 | 0.55545 | 6.240495 | 10.8585 | 1.09E-24 | 4.69E-23 | 45.2385 |
| RFC4 | 0.55552 | 3.905579 | 10.8378 | 1.31E-24 | 5.56E-23 | 45.0591 |
| CD99 | 0.55555 | 7.928933 | 4.58796 | 5.73E-06 | 2.52E-05 | 3.0208 |
| TRIM47 | 0.5556 | 6.453793 | 6.93806 | 1.31E-11 | 1.17E-10 | 15.6086 |
| ZNF311 | 0.55584 | 2.274552 | 7.44258 | 4.66E-13 | 4.92E-12 | 18.8692 |
| TFAP2A | 0.55589 | 1.804267 | 5.54433 | 4.90E-08 | 2.85E-07 | 7.60153 |
| CD33 | 0.55592 | 1.684084 | 8.82855 | 2.04E-17 | 3.49E-16 | 28.7252 |
| HFE | 0.55604 | 1.481331 | 9.07215 | 3.08E-18 | 5.75E-17 | 30.5844 |
| OR52K3P | 0.55652 | 0.663388 | 10.3856 | 6.53E-23 | 2.19E-21 | 41.1956 |
| XAF1 | 0.55653 | 1.623593 | 6.8664 | 2.06E-11 | 1.81E-10 | 15.1605 |
| PHETA2 | 0.5566 | 2.052878 | 9.55583 | 6.55E-20 | 1.50E-18 | 34.38 |
| LRP6 | 0.55663 | 4.219568 | 9.68004 | 2.38E-20 | 5.74E-19 | 35.3763 |
| ATF7IP | 0.55664 | 5.010553 | 9.22905 | 8.97E-19 | 1.78E-17 | 31.8007 |
| DONSON | 0.55667 | 3.621304 | 11.2862 | 2.45E-26 | 1.32E-24 | 48.9881 |
| MX2 | 0.5567 | 1.076251 | 7.56104 | 2.08E-13 | 2.27E-12 | 19.6611 |
| RNU6-850P | 0.55684 | 2.538931 | 5.98465 | 4.27E-09 | 2.83E-08 | 9.96617 |
| TMEM168 | 0.55708 | 3.52578 | 9.30385 | 4.95E-19 | 1.01E-17 | 32.3856 |
| CCDC8 | 0.55731 | 1.842252 | 5.10398 | 4.82E-07 | 2.47E-06 | 5.39605 |
| PBX3 | 0.55741 | 3.956936 | 7.54414 | 2.33E-13 | 2.54E-12 | 19.5475 |
| DAPP1 | 0.55753 | 0.954073 | 10.3658 | 7.74E-23 | 2.56E-21 | 41.0282 |
| PRKD2 | 0.55774 | 3.822406 | 10.5317 | 1.87E-23 | 6.69E-22 | 42.4323 |
| TNFRSF10B | 0.55794 | 4.029485 | 7.43166 | 5.02E-13 | 5.27E-12 | 18.7967 |
| AC073415.1 | 0.55801 | 1.649164 | 7.92567 | 1.62E-14 | 2.00E-13 | 22.1597 |
| CD164 | 0.55836 | 5.561174 | 11.2944 | 2.27E-26 | 1.23E-24 | 49.0608 |
| VASN | 0.55841 | 2.468353 | 4.4277 | 1.18E-05 | 4.98E-05 | 2.33012 |
| ATP7A | 0.55844 | 2.268391 | 13.1352 | 7.58E-34 | 1.36E-31 | 66.0873 |
| AC018647.2 | 0.55848 | 4.002909 | 12.2872 | 2.49E-30 | 2.35E-28 | 58.0782 |
| POLH | 0.55892 | 3.485082 | 10.0187 | 1.45E-21 | 4.10E-20 | 38.1361 |
| MCL1 | 0.55905 | 7.519793 | 9.89269 | 4.14E-21 | 1.11E-19 | 37.102 |
| LIG1 | 0.55907 | 3.648605 | 9.65472 | 2.93E-20 | 6.98E-19 | 35.1726 |
| SPRY4 | 0.55908 | 3.298591 | 3.81892 | 0.00015 | 0.000537 | -0.0856 |
| HOXA3 | 0.55929 | 0.376104 | 6.77789 | 3.62E-11 | 3.08E-10 | 14.6123 |
| BCL3 | 0.55929 | 2.768499 | 8.37214 | 6.37E-16 | 9.11E-15 | 25.3407 |
| KDM1A | 0.5593 | 5.816963 | 10.0832 | 8.46E-22 | 2.46E-20 | 38.6685 |
| AC112777.1 | 0.55935 | 1.774918 | 7.57999 | 1.82E-13 | 2.01E-12 | 19.7887 |
| WRN | 0.55937 | 3.10999 | 10.6313 | 7.89E-24 | 2.99E-22 | 43.2821 |
| TMEM35B | 0.55941 | 2.926623 | 11.6643 | 7.97E-28 | 5.39E-26 | 52.372 |
| ZNF92 | 0.55942 | 4.245555 | 9.91215 | 3.52E-21 | 9.51E-20 | 37.2611 |
| ARSD | 0.55946 | 2.665749 | 6.45428 | 2.68E-10 | 2.07E-09 | 12.6583 |
| LCTL | 0.55949 | 1.007903 | 8.82596 | 2.08E-17 | 3.55E-16 | 28.7057 |
| POLE | 0.55958 | 2.445523 | 9.82554 | 7.21E-21 | 1.87E-19 | 36.5544 |
| PAFAH2 | 0.55965 | 3.524716 | 15.898 | 5.91E-46 | 2.70E-42 | 93.6965 |
| CARD6 | 0.55975 | 2.168136 | 9.72938 | 1.59E-20 | 3.91E-19 | 35.7745 |
| TONSL | 0.55995 | 2.378196 | 8.8784 | 1.39E-17 | 2.43E-16 | 29.1027 |
| LGR6 | 0.56012 | 1.218821 | 3.91848 | 0.0001 | 0.000371 | 0.28666 |
| AC002091.2 | 0.56012 | 1.087779 | 9.62529 | 3.72E-20 | 8.76E-19 | 34.9361 |
| PREX1 | 0.56024 | 6.759724 | 7.98326 | 1.08E-14 | 1.36E-13 | 22.5626 |
| BTN2A3P | 0.5603 | 1.090226 | 12.7506 | 3.08E-32 | 4.16E-30 | 62.4226 |
| TENT5B | 0.56039 | 1.205716 | 6.23229 | 1.01E-09 | 7.27E-09 | 11.3643 |
| SLC44A3-AS1 | 0.56044 | 1.163521 | 8.92318 | 9.83E-18 | 1.75E-16 | 29.4432 |
| AC241585.1 | 0.56048 | 4.244911 | 7.08935 | 4.90E-12 | 4.62E-11 | 16.567 |
| LPAR1 | 0.56049 | 4.827108 | 4.35355 | 1.64E-05 | 6.77E-05 | 2.01822 |
| AC118549.1 | 0.5607 | 3.767656 | 12.0549 | 2.18E-29 | 1.82E-27 | 55.9324 |
| DHRSX | 0.56087 | 4.137737 | 11.4185 | 7.43E-27 | 4.36E-25 | 50.1651 |
| AL021807.1 | 0.56103 | 1.048309 | 9.18443 | 1.28E-18 | 2.48E-17 | 31.4533 |
| DPY19L3 | 0.56118 | 4.767899 | 11.2153 | 4.61E-26 | 2.40E-24 | 48.3611 |
| TMEM220 | 0.56125 | 2.282399 | 8.63248 | 9.09E-17 | 1.44E-15 | 27.2553 |
| SLC66A3 | 0.56134 | 3.468591 | 10.8265 | 1.44E-24 | 6.09E-23 | 44.9617 |
| RIPK1 | 0.56167 | 4.346258 | 13.287 | 1.73E-34 | 3.57E-32 | 67.5476 |
| MTFR2 | 0.56179 | 1.028936 | 11.1934 | 5.61E-26 | 2.87E-24 | 48.1674 |
| HPD | 0.5619 | 0.899908 | 6.41682 | 3.37E-10 | 2.56E-09 | 12.4373 |
| ZMYM4 | 0.56193 | 4.960973 | 12.2982 | 2.24E-30 | 2.13E-28 | 58.1806 |
| SNX7 | 0.56197 | 4.997394 | 9.62509 | 3.73E-20 | 8.77E-19 | 34.9345 |
| ARMCX2 | 0.5623 | 5.140003 | 8.42436 | 4.32E-16 | 6.30E-15 | 25.7213 |
| AC106865.1 | 0.56236 | 0.69989 | 8.68052 | 6.31E-17 | 1.02E-15 | 27.6132 |
| AC015813.6 | 0.56253 | 3.423206 | 10.6028 | 1.01E-23 | 3.78E-22 | 43.0386 |
| PINLYP | 0.56255 | 1.49026 | 5.98656 | 4.22E-09 | 2.80E-08 | 9.97678 |
| USP18 | 0.56258 | 2.973806 | 7.67968 | 9.15E-14 | 1.04E-12 | 20.464 |
| PARP11 | 0.56281 | 3.333144 | 11.3769 | 1.08E-26 | 6.12E-25 | 49.7947 |
| FKBP7 | 0.5629 | 2.916738 | 13.0814 | 1.28E-33 | 2.20E-31 | 65.5715 |
| TMOD3 | 0.56296 | 3.47517 | 11.5126 | 3.17E-27 | 1.95E-25 | 51.0074 |
| CRYBG1 | 0.56299 | 1.031655 | 8.43963 | 3.86E-16 | 5.66E-15 | 25.8329 |
| WSCD1 | 0.56315 | 5.606061 | 5.9316 | 5.78E-09 | 3.78E-08 | 9.67303 |
| EIF2AK3 | 0.5633 | 3.794353 | 11.1066 | 1.22E-25 | 6.00E-24 | 47.4039 |
| PFN1 | 0.56334 | 8.559887 | 12.321 | 1.81E-30 | 1.75E-28 | 58.3927 |
| ZNF548 | 0.56366 | 3.313493 | 12.1627 | 7.97E-30 | 7.14E-28 | 56.9259 |
| CLEC2B | 0.56371 | 2.069532 | 8.16443 | 2.92E-15 | 3.90E-14 | 23.8445 |
| MBNL3 | 0.56374 | 1.252422 | 9.69111 | 2.18E-20 | 5.27E-19 | 35.4655 |
| H3P6 | 0.56404 | 3.958916 | 8.27767 | 1.28E-15 | 1.76E-14 | 24.6567 |
| NUP160 | 0.56408 | 4.128836 | 10.7444 | 2.95E-24 | 1.19E-22 | 44.2527 |
| PXYLP1 | 0.56424 | 4.478828 | 8.96443 | 7.14E-18 | 1.29E-16 | 29.7579 |
| LPCAT3 | 0.56447 | 2.939709 | 10.3657 | 7.75E-23 | 2.56E-21 | 41.0271 |
| PIK3R3 | 0.56454 | 3.999373 | 9.86222 | 5.33E-21 | 1.41E-19 | 36.8532 |
| MIR590 | 0.56464 | 2.254143 | 6.63822 | 8.67E-11 | 7.09E-10 | 13.7593 |
| PLOD2 | 0.56481 | 3.967081 | 7.39155 | 6.58E-13 | 6.80E-12 | 18.5311 |
| TCTEX1D1 | 0.56485 | 1.126792 | 4.42436 | 1.20E-05 | 5.05E-05 | 2.31598 |
| RPF1 | 0.56492 | 5.370032 | 13.0744 | 1.37E-33 | 2.32E-31 | 65.5044 |
| AC131097.3 | 0.56542 | 0.693547 | 7.50489 | 3.05E-13 | 3.29E-12 | 19.2845 |
| POLD3 | 0.56549 | 3.323983 | 14.5952 | 3.88E-40 | 2.96E-37 | 80.4276 |
| ZMPSTE24 | 0.56558 | 5.568445 | 12.5598 | 1.90E-31 | 2.19E-29 | 60.6242 |
| B4GALT1 | 0.5657 | 3.886514 | 8.64888 | 8.03E-17 | 1.27E-15 | 27.3773 |
| AC015922.3 | 0.56589 | 3.033697 | 6.28827 | 7.27E-10 | 5.32E-09 | 11.687 |
| NKX2-5 | 0.56597 | 0.485536 | 6.89723 | 1.70E-11 | 1.50E-10 | 15.3528 |
| IER5L | 0.5661 | 3.107404 | 6.76017 | 4.05E-11 | 3.43E-10 | 14.5033 |
| GTF2IP23 | 0.56616 | 2.063845 | 8.37695 | 6.15E-16 | 8.81E-15 | 25.3757 |
| CD3D | 0.56622 | 0.820327 | 7.82773 | 3.25E-14 | 3.88E-13 | 21.4796 |
| SPPL2A | 0.56625 | 4.207436 | 12.6613 | 7.22E-32 | 8.93E-30 | 61.5796 |
| EXOSC10 | 0.56634 | 4.995748 | 11.7405 | 3.97E-28 | 2.82E-26 | 53.0612 |
| ADGRL4 | 0.56642 | 3.483648 | 7.65795 | 1.06E-13 | 1.20E-12 | 20.3162 |
| RBM38 | 0.56667 | 5.304992 | 11.049 | 2.03E-25 | 9.65E-24 | 46.8986 |
| ZNF560 | 0.56676 | 1.487963 | 4.50038 | 8.54E-06 | 3.67E-05 | 2.64056 |
| ZNF486 | 0.56678 | 2.588048 | 11.8141 | 2.02E-28 | 1.49E-26 | 53.7301 |
| TP73-AS1 | 0.56692 | 2.546811 | 6.03195 | 3.26E-09 | 2.19E-08 | 10.2295 |
| CIAO2A | 0.56698 | 5.081289 | 15.5735 | 1.72E-44 | 4.97E-41 | 90.356 |
| ARHGEF1 | 0.56698 | 4.577698 | 9.36074 | 3.15E-19 | 6.59E-18 | 32.8328 |
| H1-9P | 0.5672 | 0.754163 | 5.9783 | 4.43E-09 | 2.93E-08 | 9.93098 |
| TEAD3 | 0.56728 | 2.055833 | 6.79011 | 3.35E-11 | 2.86E-10 | 14.6877 |
| PRPF38B | 0.56789 | 4.893645 | 12.6269 | 1.00E-31 | 1.21E-29 | 61.2549 |
| LTBR | 0.5679 | 2.807548 | 8.69987 | 5.45E-17 | 8.86E-16 | 27.7578 |
| PTN | 0.56792 | 9.333912 | 6.46181 | 2.56E-10 | 1.98E-09 | 12.7029 |
| LYPLA1 | 0.56794 | 4.187569 | 12.7576 | 2.88E-32 | 3.91E-30 | 62.4886 |
| AC091057.1 | 0.56801 | 0.840037 | 10.6493 | 6.75E-24 | 2.59E-22 | 43.4362 |
| APLNR | 0.56811 | 6.247924 | 3.50344 | 0.0005 | 0.00163 | -1.2055 |
| HROB | 0.56839 | 1.726168 | 10.6853 | 4.94E-24 | 1.93E-22 | 43.7452 |
| RHBDF1 | 0.5686 | 3.384549 | 6.78816 | 3.39E-11 | 2.90E-10 | 14.6757 |
| EFNA4 | 0.56865 | 2.509425 | 8.65612 | 7.60E-17 | 1.21E-15 | 27.4312 |
| PHTF1 | 0.56887 | 2.668078 | 10.3395 | 9.68E-23 | 3.17E-21 | 40.8071 |
| TUBB | 0.56906 | 8.781661 | 10.841 | 1.27E-24 | 5.41E-23 | 45.0873 |
| HMGB1P1 | 0.56922 | 2.01352 | 7.33011 | 9.95E-13 | 1.01E-11 | 18.1265 |
| RAI14 | 0.56944 | 3.135038 | 11.4696 | 4.68E-27 | 2.80E-25 | 50.6218 |
| NBPF8 | 0.56954 | 1.785853 | 10.6018 | 1.02E-23 | 3.81E-22 | 43.0302 |
| ZNF473 | 0.5697 | 2.586806 | 13.5203 | 1.77E-35 | 4.43E-33 | 69.8068 |
| CHSY1 | 0.56974 | 4.382366 | 11.2084 | 4.91E-26 | 2.54E-24 | 48.3003 |
| CTNND1 | 0.57006 | 6.215612 | 12.7188 | 4.17E-32 | 5.44E-30 | 62.1223 |
| C1orf194 | 0.57027 | 2.871867 | 4.86913 | 1.53E-06 | 7.29E-06 | 4.28666 |
| CARD8-AS1 | 0.57037 | 1.997043 | 12.8535 | 1.15E-32 | 1.66E-30 | 63.3974 |
| SMAD1 | 0.57047 | 4.441365 | 9.23345 | 8.66E-19 | 1.72E-17 | 31.835 |
| ARL2BP | 0.57068 | 3.954856 | 10.3798 | 6.87E-23 | 2.29E-21 | 41.1465 |
| ZNF432 | 0.57103 | 2.954452 | 11.6644 | 7.96E-28 | 5.39E-26 | 52.3732 |
| TMEM209 | 0.57107 | 4.131339 | 11.8094 | 2.11E-28 | 1.55E-26 | 53.687 |
| OSCAR | 0.57113 | 2.526098 | 7.53717 | 2.45E-13 | 2.66E-12 | 19.5007 |
| SEC61A1 | 0.57122 | 7.54515 | 13.0538 | 1.67E-33 | 2.73E-31 | 65.3067 |
| UGCG | 0.57126 | 4.238037 | 10.6556 | 6.39E-24 | 2.47E-22 | 43.4905 |
| ST6GAL1 | 0.57132 | 5.082822 | 8.79084 | 2.72E-17 | 4.58E-16 | 28.4407 |
| TLN1 | 0.57137 | 6.577475 | 11.9125 | 8.15E-29 | 6.36E-27 | 54.627 |
| DARS2 | 0.57163 | 3.904434 | 11.9173 | 7.80E-29 | 6.11E-27 | 54.6706 |
| COL6A1 | 0.57168 | 6.556066 | 7.68602 | 8.76E-14 | 9.97E-13 | 20.5072 |
| HLA-G | 0.57169 | 1.77815 | 7.14641 | 3.37E-12 | 3.23E-11 | 16.9328 |
| FCGR1CP | 0.57175 | 2.258591 | 6.23619 | 9.91E-10 | 7.12E-09 | 11.3867 |
| DDX58 | 0.57176 | 3.668629 | 9.0286 | 4.33E-18 | 7.96E-17 | 30.2494 |
| MMP19 | 0.57188 | 0.904022 | 6.69885 | 5.95E-11 | 4.95E-10 | 14.1278 |
| STK36 | 0.57219 | 4.245241 | 10.0402 | 1.21E-21 | 3.45E-20 | 38.3136 |
| TMEM123 | 0.57227 | 6.617364 | 12.1008 | 1.42E-29 | 1.22E-27 | 56.354 |
| RAD54B | 0.57232 | 1.025727 | 14.3369 | 5.27E-39 | 2.95E-36 | 77.8455 |
| QTRT2 | 0.57236 | 2.61464 | 14.3832 | 3.30E-39 | 1.89E-36 | 78.3074 |
| LRRCC1 | 0.57239 | 4.228371 | 10.8383 | 1.30E-24 | 5.54E-23 | 45.0634 |
| MEX3A | 0.57287 | 4.558144 | 5.87485 | 7.96E-09 | 5.11E-08 | 9.36185 |
| IGKC | 0.57294 | 1.6119 | 3.78217 | 0.00018 | 0.000614 | -0.2208 |
| KIF24 | 0.57313 | 1.584216 | 10.7798 | 2.17E-24 | 8.92E-23 | 44.5584 |
| DCHS1 | 0.57331 | 3.968651 | 6.77231 | 3.75E-11 | 3.19E-10 | 14.5779 |
| MIR3125 | 0.57349 | 1.55203 | 5.94863 | 5.24E-09 | 3.44E-08 | 9.76687 |
| CERS2 | 0.57351 | 5.899357 | 11.6702 | 7.55E-28 | 5.14E-26 | 52.4257 |
| PPIAP48 | 0.57364 | 0.84981 | 7.35086 | 8.65E-13 | 8.84E-12 | 18.2628 |
| NOTCH2 | 0.57371 | 5.086637 | 7.25574 | 1.63E-12 | 1.62E-11 | 17.6403 |
| IGHA1 | 0.57387 | 1.652801 | 3.49473 | 0.00052 | 0.001679 | -1.2352 |
| IFNAR2 | 0.57392 | 3.607489 | 13.5273 | 1.65E-35 | 4.15E-33 | 69.8746 |
| ZNF558 | 0.57414 | 3.537539 | 12.2151 | 4.88E-30 | 4.46E-28 | 57.4104 |
| MIS18A | 0.57417 | 4.029717 | 13.3003 | 1.52E-34 | 3.20E-32 | 67.6762 |
| METTL1 | 0.57425 | 3.787699 | 7.32541 | 1.03E-12 | 1.04E-11 | 18.0956 |
| ZNF234 | 0.57429 | 2.739183 | 10.5509 | 1.58E-23 | 5.73E-22 | 42.5963 |
| CGAS | 0.57467 | 1.262483 | 11.8171 | 1.96E-28 | 1.46E-26 | 53.7569 |
| C4orf46 | 0.57477 | 2.968622 | 13.5545 | 1.26E-35 | 3.24E-33 | 70.1393 |
| POGLUT2 | 0.57477 | 2.877465 | 10.7137 | 3.86E-24 | 1.53E-22 | 43.9888 |
| RIT1 | 0.57493 | 4.594972 | 14.9421 | 1.14E-41 | 1.42E-38 | 83.9223 |
| NUP188 | 0.57503 | 4.913892 | 11.8994 | 9.20E-29 | 7.16E-27 | 54.5075 |
| FKBP14 | 0.57526 | 3.85616 | 10.9822 | 3.66E-25 | 1.69E-23 | 46.3143 |
| TMEM159 | 0.57541 | 2.668639 | 7.83182 | 3.16E-14 | 3.78E-13 | 21.5079 |
| ACSL1 | 0.57547 | 4.43193 | 7.77248 | 4.79E-14 | 5.61E-13 | 21.0989 |
| B3GNT7 | 0.5755 | 1.57878 | 6.0895 | 2.34E-09 | 1.60E-08 | 10.5522 |
| CCER2 | 0.57551 | 2.183336 | 5.52756 | 5.36E-08 | 3.10E-07 | 7.51457 |
| CD109 | 0.57552 | 1.929563 | 7.33459 | 9.65E-13 | 9.81E-12 | 18.1559 |
| ILDR2 | 0.57559 | 3.802053 | 7.48299 | 3.54E-13 | 3.79E-12 | 19.1382 |
| SERBP1 | 0.57572 | 6.325803 | 14.0202 | 1.26E-37 | 4.78E-35 | 74.7048 |
| MS4A14 | 0.57582 | 1.314242 | 9.42505 | 1.88E-19 | 4.04E-18 | 33.3404 |
| MAGT1 | 0.57585 | 5.462715 | 11.1692 | 6.97E-26 | 3.51E-24 | 47.9541 |
| IL17RA | 0.57594 | 3.612399 | 10.2814 | 1.59E-22 | 5.04E-21 | 40.3192 |
| FSCN1 | 0.57598 | 7.424786 | 7.69008 | 8.51E-14 | 9.71E-13 | 20.5349 |
| STING1 | 0.57599 | 3.662541 | 8.68038 | 6.32E-17 | 1.02E-15 | 27.6122 |
| AC141557.1 | 0.5761 | 2.281883 | 4.32621 | 1.85E-05 | 7.57E-05 | 1.90441 |
| LPAR4 | 0.57624 | 1.923991 | 7.03477 | 6.99E-12 | 6.49E-11 | 16.2193 |
| ISL2 | 0.57649 | 0.49554 | 9.84756 | 6.01E-21 | 1.57E-19 | 36.7337 |
| PHEX | 0.5766 | 1.573053 | 7.92813 | 1.60E-14 | 1.97E-13 | 22.1769 |
| AC025171.5 | 0.57667 | 0.913419 | 8.23962 | 1.69E-15 | 2.31E-14 | 24.3828 |
| NCAPD3 | 0.57673 | 3.562738 | 11.0763 | 1.59E-25 | 7.70E-24 | 47.1378 |
| STMP1 | 0.57673 | 5.43947 | 13.4475 | 3.62E-35 | 8.40E-33 | 69.0995 |
| PRKDC | 0.57682 | 5.390801 | 10.746 | 2.91E-24 | 1.17E-22 | 44.2669 |
| ENG | 0.57701 | 5.457214 | 8.86369 | 1.56E-17 | 2.70E-16 | 28.9912 |
| LIMS1 | 0.57702 | 4.211556 | 12.232 | 4.17E-30 | 3.85E-28 | 57.5667 |
| ARID5A | 0.57721 | 4.427627 | 7.84784 | 2.82E-14 | 3.39E-13 | 21.6187 |
| GALNT10 | 0.57733 | 4.645466 | 8.86998 | 1.48E-17 | 2.58E-16 | 29.0389 |
| S100PBP | 0.57733 | 3.386545 | 13.4879 | 2.43E-35 | 5.82E-33 | 69.4922 |
| VASH1 | 0.57741 | 5.477911 | 10.5943 | 1.09E-23 | 4.05E-22 | 42.9665 |
| DTYMK | 0.57743 | 4.903739 | 10.8967 | 7.78E-25 | 3.45E-23 | 45.5699 |
| CAP1 | 0.57754 | 7.457514 | 12.9407 | 4.97E-33 | 7.44E-31 | 64.227 |
| ZNF146 | 0.57775 | 5.87565 | 11.8895 | 1.01E-28 | 7.75E-27 | 54.4173 |
| DEK | 0.57788 | 5.961526 | 12.5767 | 1.62E-31 | 1.89E-29 | 60.7827 |
| SLC35D2 | 0.57789 | 4.570526 | 11.7481 | 3.70E-28 | 2.64E-26 | 53.1309 |
| AC093627.7 | 0.5779 | 1.768185 | 7.59586 | 1.63E-13 | 1.81E-12 | 19.8958 |
| LDHA | 0.57819 | 5.926185 | 7.07398 | 5.41E-12 | 5.08E-11 | 16.4689 |
| BATF3 | 0.57819 | 2.095409 | 7.06691 | 5.67E-12 | 5.31E-11 | 16.4238 |
| GNGT2 | 0.57846 | 1.767458 | 9.81395 | 7.94E-21 | 2.05E-19 | 36.4601 |
| AC008750.1 | 0.57869 | 1.197622 | 9.44738 | 1.57E-19 | 3.41E-18 | 33.5172 |
| BST1 | 0.5792 | 1.5309 | 10.5006 | 2.44E-23 | 8.62E-22 | 42.1681 |
| ADAMTSL4 | 0.57921 | 1.435514 | 8.75077 | 3.70E-17 | 6.13E-16 | 28.1392 |
| CYTOR | 0.57922 | 1.235258 | 7.10334 | 4.47E-12 | 4.23E-11 | 16.6565 |
| F11R | 0.57928 | 2.595659 | 8.49741 | 2.51E-16 | 3.76E-15 | 26.2566 |
| PROM1 | 0.57958 | 2.398151 | 7.24235 | 1.79E-12 | 1.76E-11 | 17.5532 |
| RN7SKP16 | 0.57958 | 1.297178 | 9.31299 | 4.61E-19 | 9.42E-18 | 32.4574 |
| ERBIN | 0.57965 | 5.59472 | 9.20512 | 1.08E-18 | 2.13E-17 | 31.6142 |
| CHD1 | 0.57966 | 3.318528 | 12.4391 | 5.95E-31 | 6.37E-29 | 59.4931 |
| SNRNP40 | 0.57989 | 4.452692 | 13.5123 | 1.92E-35 | 4.69E-33 | 69.7286 |
| MIR6503 | 0.57997 | 0.593378 | 7.37185 | 7.51E-13 | 7.72E-12 | 18.4011 |
| BICD1 | 0.58002 | 3.076548 | 10.0947 | 7.68E-22 | 2.24E-20 | 38.764 |
| AL355922.1 | 0.58011 | 1.067739 | 6.94976 | 1.21E-11 | 1.09E-10 | 15.6821 |
| WDR38 | 0.58026 | 1.079156 | 4.9203 | 1.19E-06 | 5.78E-06 | 4.52435 |
| THBS3 | 0.58029 | 3.798345 | 9.03975 | 3.97E-18 | 7.33E-17 | 30.3351 |
| FZD1 | 0.5803 | 3.028581 | 9.42816 | 1.83E-19 | 3.95E-18 | 33.365 |
| AC010332.1 | 0.58031 | 4.634958 | 7.79639 | 4.05E-14 | 4.79E-13 | 21.2634 |
| LINC02308 | 0.58034 | 1.322708 | 6.66287 | 7.44E-11 | 6.12E-10 | 13.9087 |
| IFT81 | 0.58035 | 3.574188 | 12.2226 | 4.55E-30 | 4.18E-28 | 57.4797 |
| WIPF1 | 0.58051 | 4.471394 | 11.5786 | 1.74E-27 | 1.11E-25 | 51.5997 |
| AC098851.1 | 0.58054 | 0.986434 | 10.4356 | 4.26E-23 | 1.46E-21 | 41.6174 |
| NLRC4 | 0.58057 | 1.583785 | 11.5719 | 1.85E-27 | 1.18E-25 | 51.5398 |
| NANP | 0.58059 | 2.353693 | 13.2875 | 1.73E-34 | 3.57E-32 | 67.5526 |
| HMG20B | 0.58092 | 5.341955 | 11.0963 | 1.33E-25 | 6.54E-24 | 47.3133 |
| AC011899.2 | 0.58111 | 0.884189 | 11.6432 | 9.67E-28 | 6.49E-26 | 52.1813 |
| CYP27B1 | 0.58112 | 0.842117 | 7.39465 | 6.44E-13 | 6.66E-12 | 18.5516 |
| LSM14A | 0.58115 | 6.14513 | 12.9621 | 4.04E-33 | 6.10E-31 | 64.4317 |
| G0S2 | 0.58142 | 1.738457 | 4.27109 | 2.35E-05 | 9.46E-05 | 1.67703 |
| PHC2 | 0.58155 | 6.837571 | 12.4618 | 4.80E-31 | 5.21E-29 | 59.7057 |
| DHX40 | 0.58162 | 5.17801 | 13.1615 | 5.88E-34 | 1.09E-31 | 66.3395 |
| KANSL1L | 0.58186 | 2.826482 | 8.30901 | 1.01E-15 | 1.42E-14 | 24.883 |
| SERTAD3 | 0.58188 | 3.755448 | 8.49483 | 2.56E-16 | 3.83E-15 | 26.2376 |
| EVA1C | 0.5819 | 2.853263 | 6.83787 | 2.48E-11 | 2.15E-10 | 14.9832 |
| ZNF677 | 0.58192 | 3.171554 | 10.8681 | 1.00E-24 | 4.36E-23 | 45.3219 |
| GSDMD | 0.58202 | 3.165606 | 7.51984 | 2.75E-13 | 2.98E-12 | 19.3846 |
| BACE2 | 0.58225 | 2.11079 | 9.53235 | 7.92E-20 | 1.79E-18 | 34.1926 |
| EFNB1 | 0.58247 | 4.327619 | 11.5144 | 3.12E-27 | 1.93E-25 | 51.0231 |
| SZRD1 | 0.58253 | 5.88083 | 12.9972 | 2.88E-33 | 4.45E-31 | 64.7664 |
| P3H1 | 0.58264 | 3.008683 | 11.5272 | 2.78E-27 | 1.72E-25 | 51.1379 |
| STXBP2 | 0.58287 | 2.320218 | 8.97094 | 6.79E-18 | 1.23E-16 | 29.8076 |
| HPS3 | 0.58301 | 3.515813 | 12.7176 | 4.22E-32 | 5.50E-30 | 62.1101 |
| MFAP4 | 0.58342 | 4.512766 | 5.17461 | 3.37E-07 | 1.76E-06 | 5.73887 |
| LDLRAD3 | 0.58374 | 5.799715 | 8.57939 | 1.36E-16 | 2.10E-15 | 26.8614 |
| SPAG1 | 0.58383 | 2.618436 | 7.8111 | 3.65E-14 | 4.34E-13 | 21.3648 |
| ERAP1 | 0.58399 | 4.287226 | 9.71095 | 1.85E-20 | 4.52E-19 | 35.6256 |
| SHC1 | 0.58405 | 4.699138 | 10.1805 | 3.73E-22 | 1.13E-20 | 39.4769 |
| SAMHD1 | 0.58447 | 5.912528 | 7.88007 | 2.25E-14 | 2.73E-13 | 21.8423 |
| CCDC15 | 0.58473 | 1.570535 | 12.6467 | 8.29E-32 | 1.01E-29 | 61.4418 |
| BAX | 0.5849 | 5.555291 | 11.6309 | 1.08E-27 | 7.21E-26 | 52.0709 |
| ZNF518B | 0.58517 | 2.304818 | 6.84143 | 2.42E-11 | 2.11E-10 | 15.0053 |
| RESF1 | 0.58575 | 3.490885 | 13.1325 | 7.78E-34 | 1.39E-31 | 66.0616 |
| ABCD1 | 0.58593 | 3.535013 | 11.774 | 2.92E-28 | 2.10E-26 | 53.3658 |
| AC138207.5 | 0.5861 | 3.063543 | 7.55475 | 2.17E-13 | 2.37E-12 | 19.6188 |
| AFAP1L1 | 0.5864 | 2.667329 | 7.40497 | 6.01E-13 | 6.24E-12 | 18.6199 |
| SNAI2 | 0.58673 | 2.020504 | 6.72159 | 5.16E-11 | 4.33E-10 | 14.2667 |
| POFUT1 | 0.58692 | 5.20104 | 13.1481 | 6.70E-34 | 1.22E-31 | 66.2104 |
| DRAM2 | 0.58706 | 4.988456 | 13.4397 | 3.90E-35 | 8.99E-33 | 69.0239 |
| ARHGAP4 | 0.58759 | 3.769759 | 7.83401 | 3.11E-14 | 3.72E-13 | 21.523 |
| MFAP3 | 0.58769 | 3.224157 | 8.01176 | 8.79E-15 | 1.11E-13 | 22.7628 |
| RASAL3 | 0.58787 | 2.443216 | 7.79666 | 4.04E-14 | 4.78E-13 | 21.2653 |
| ARL11 | 0.58798 | 1.315983 | 10.9725 | 3.99E-25 | 1.82E-23 | 46.2295 |
| SPA17 | 0.58807 | 2.50747 | 7.90741 | 1.85E-14 | 2.27E-13 | 22.0324 |
| ADCY7 | 0.58809 | 2.388632 | 8.54175 | 1.80E-16 | 2.75E-15 | 26.5831 |
| IGF2 | 0.58826 | 3.455319 | 4.81705 | 1.96E-06 | 9.23E-06 | 4.04701 |
| TUT4 | 0.58846 | 3.059553 | 11.332 | 1.62E-26 | 9.02E-25 | 49.3951 |
| ARL4C | 0.58873 | 4.902875 | 5.16051 | 3.62E-07 | 1.89E-06 | 5.67009 |
| PLA2G2A | 0.58888 | 0.584454 | 4.8487 | 1.69E-06 | 8.00E-06 | 4.19238 |
| RBP1 | 0.58899 | 3.253077 | 4.0448 | 6.11E-05 | 0.000231 | 0.77193 |
| IKBKE | 0.58934 | 2.489568 | 10.2364 | 2.33E-22 | 7.23E-21 | 39.9425 |
| TRIM21 | 0.58938 | 4.016167 | 8.40479 | 5.00E-16 | 7.23E-15 | 25.5785 |
| AQP5 | 0.58939 | 0.912133 | 5.02246 | 7.23E-07 | 3.63E-06 | 5.00563 |
| FKBP5 | 0.58951 | 4.022105 | 4.59913 | 5.45E-06 | 2.41E-05 | 3.0698 |
| PLOD3 | 0.58964 | 5.132095 | 10.8144 | 1.60E-24 | 6.71E-23 | 44.8574 |
| CHODL | 0.58974 | 1.170156 | 6.93529 | 1.33E-11 | 1.19E-10 | 15.5912 |
| PIK3R6 | 0.58983 | 1.418186 | 10.9163 | 6.55E-25 | 2.93E-23 | 45.7406 |
| NFATC1 | 0.5902 | 2.194225 | 8.12316 | 3.94E-15 | 5.18E-14 | 23.5506 |
| SH3TC1 | 0.59044 | 2.696713 | 7.45359 | 4.32E-13 | 4.58E-12 | 18.9424 |
| SLC34A2 | 0.59046 | 0.529507 | 8.55045 | 1.69E-16 | 2.58E-15 | 26.6473 |
| TRAIP | 0.59074 | 2.248239 | 10.439 | 4.14E-23 | 1.43E-21 | 41.6464 |
| AL450124.1 | 0.59085 | 2.937751 | 8.01817 | 8.39E-15 | 1.07E-13 | 22.8079 |
| MFSD1 | 0.59085 | 5.021333 | 13.565 | 1.14E-35 | 2.95E-33 | 70.241 |
| EXTL2 | 0.59101 | 4.233421 | 11.7767 | 2.85E-28 | 2.06E-26 | 53.3901 |
| TEP1 | 0.59118 | 3.017578 | 12.3062 | 2.08E-30 | 1.99E-28 | 58.2549 |
| FBXO4 | 0.59133 | 3.189713 | 11.8249 | 1.83E-28 | 1.36E-26 | 53.8279 |
| UGDH | 0.59144 | 4.315771 | 10.1623 | 4.35E-22 | 1.31E-20 | 39.3246 |
| LST1 | 0.59145 | 4.075152 | 7.13062 | 3.74E-12 | 3.57E-11 | 16.8313 |
| LBR | 0.59148 | 4.834055 | 11.4872 | 3.99E-27 | 2.42E-25 | 50.7794 |
| HAUS8 | 0.59195 | 1.996368 | 12.5941 | 1.37E-31 | 1.62E-29 | 60.9461 |
| NFIA | 0.592 | 5.350923 | 8.06786 | 5.87E-15 | 7.60E-14 | 23.1585 |
| PLK3 | 0.59225 | 3.334489 | 9.47377 | 1.27E-19 | 2.80E-18 | 33.7265 |
| C19orf48 | 0.59247 | 4.537642 | 10.2451 | 2.16E-22 | 6.75E-21 | 40.0157 |
| PKD2 | 0.59267 | 4.038804 | 10.6632 | 5.98E-24 | 2.31E-22 | 43.5558 |
| TXNDC12 | 0.59293 | 5.665827 | 13.4942 | 2.29E-35 | 5.50E-33 | 69.5533 |
| NUP62 | 0.59316 | 5.012762 | 13.559 | 1.21E-35 | 3.11E-33 | 70.1828 |
| FHL3 | 0.59322 | 4.793985 | 8.68719 | 6.00E-17 | 9.69E-16 | 27.663 |
| TMEM106A | 0.59323 | 2.224408 | 8.53958 | 1.83E-16 | 2.79E-15 | 26.5671 |
| PRIM2 | 0.59326 | 2.516918 | 14.8189 | 4.00E-41 | 4.30E-38 | 82.678 |
| EMB | 0.59345 | 2.061025 | 8.01773 | 8.42E-15 | 1.07E-13 | 22.8048 |
| NPC2 | 0.59346 | 6.573277 | 8.57543 | 1.40E-16 | 2.16E-15 | 26.832 |
| MEX3C | 0.59352 | 4.656312 | 11.0842 | 1.48E-25 | 7.24E-24 | 47.2069 |
| FOSL2 | 0.5937 | 4.281983 | 6.78418 | 3.48E-11 | 2.97E-10 | 14.6511 |
| AC060766.1 | 0.59405 | 1.448015 | 9.73983 | 1.46E-20 | 3.62E-19 | 35.859 |
| ERMAP | 0.59423 | 3.583991 | 11.1933 | 5.62E-26 | 2.87E-24 | 48.167 |
| MREG | 0.59441 | 3.707467 | 8.56338 | 1.53E-16 | 2.35E-15 | 26.7429 |
| GPR84 | 0.59446 | 1.207741 | 8.03882 | 7.24E-15 | 9.26E-14 | 22.9534 |
| CTBS | 0.59544 | 2.536575 | 10.1878 | 3.51E-22 | 1.07E-20 | 39.5371 |
| RSAD2 | 0.59548 | 2.712985 | 5.47685 | 7.02E-08 | 4.00E-07 | 7.25311 |
| DDX12P | 0.59564 | 1.484568 | 8.20899 | 2.11E-15 | 2.86E-14 | 24.1631 |
| ARAP3 | 0.59583 | 2.911708 | 7.2897 | 1.30E-12 | 1.31E-11 | 17.8618 |
| WDR77 | 0.596 | 4.678644 | 14.6062 | 3.48E-40 | 2.72E-37 | 80.5379 |
| SELENOF | 0.5961 | 7.208788 | 13.4973 | 2.22E-35 | 5.38E-33 | 69.5834 |
| HEATR1 | 0.59627 | 3.421941 | 10.9155 | 6.59E-25 | 2.95E-23 | 45.7335 |
| CNTF | 0.59663 | 1.834264 | 8.57589 | 1.39E-16 | 2.15E-15 | 26.8354 |
| RTP4 | 0.59683 | 2.935047 | 7.77996 | 4.55E-14 | 5.34E-13 | 21.1503 |
| DDX20 | 0.59711 | 3.090655 | 13.952 | 2.48E-37 | 8.65E-35 | 74.0323 |
| IGHG2 | 0.59725 | 1.1717 | 3.99054 | 7.63E-05 | 0.000283 | 0.56173 |
| STAT3 | 0.59746 | 6.025306 | 13.0694 | 1.43E-33 | 2.40E-31 | 65.4567 |
| TRIP6 | 0.59757 | 5.49545 | 6.71887 | 5.25E-11 | 4.40E-10 | 14.2501 |
| PRKD3 | 0.5976 | 4.107544 | 11.8002 | 2.30E-28 | 1.68E-26 | 53.6031 |
| GLIPR2 | 0.59765 | 6.033359 | 8.75106 | 3.69E-17 | 6.12E-16 | 28.1414 |
| ZNF816 | 0.59765 | 1.839223 | 12.9697 | 3.76E-33 | 5.69E-31 | 64.5036 |
| RECQL4 | 0.59768 | 3.387928 | 8.15624 | 3.10E-15 | 4.12E-14 | 23.786 |
| TRIM24 | 0.5977 | 4.651188 | 10.534 | 1.83E-23 | 6.57E-22 | 42.4522 |
| H2AC8 | 0.59794 | 2.303618 | 7.09991 | 4.57E-12 | 4.32E-11 | 16.6345 |
| GNL3L | 0.59815 | 3.363973 | 9.78344 | 1.02E-20 | 2.59E-19 | 36.2123 |
| MANEA | 0.59832 | 3.252973 | 11.5233 | 2.88E-27 | 1.78E-25 | 51.1031 |
| DDX60 | 0.59891 | 3.071721 | 8.36225 | 6.85E-16 | 9.75E-15 | 25.2688 |
| TRIM38 | 0.59902 | 1.842589 | 11.0708 | 1.67E-25 | 8.05E-24 | 47.0897 |
| ABCC1 | 0.59914 | 3.371612 | 12.1098 | 1.31E-29 | 1.13E-27 | 56.4374 |
| ARHGAP15 | 0.5992 | 1.29458 | 10.981 | 3.70E-25 | 1.70E-23 | 46.304 |
| UBA7 | 0.59951 | 4.481959 | 8.12752 | 3.82E-15 | 5.03E-14 | 23.5815 |
| GPR37 | 0.59971 | 5.261388 | 4.15721 | 3.82E-05 | 0.000149 | 1.21581 |
| MOXD1 | 0.60026 | 1.856609 | 3.54545 | 0.00043 | 0.001413 | -1.0617 |
| ARHGAP9 | 0.60044 | 2.366081 | 9.15687 | 1.59E-18 | 3.05E-17 | 31.2394 |
| SH2B3 | 0.60056 | 4.037911 | 11.0655 | 1.75E-25 | 8.42E-24 | 47.0431 |
| EGR1 | 0.60082 | 6.847936 | 3.81345 | 0.00016 | 0.000548 | -0.1058 |
| CENPN | 0.60094 | 2.553642 | 11.2914 | 2.33E-26 | 1.26E-24 | 49.0343 |
| SOX9 | 0.60107 | 7.312714 | 7.97691 | 1.13E-14 | 1.42E-13 | 22.518 |
| CDCP1 | 0.60124 | 1.263138 | 9.83936 | 6.44E-21 | 1.68E-19 | 36.6669 |
| TNPO1 | 0.60128 | 5.196201 | 12.2626 | 3.13E-30 | 2.93E-28 | 57.8498 |
| AC093673.1 | 0.60132 | 4.837239 | 8.30908 | 1.01E-15 | 1.42E-14 | 24.8835 |
| MIR4435-2HG | 0.60158 | 1.195232 | 7.90351 | 1.90E-14 | 2.33E-13 | 22.0052 |
| AC027307.2 | 0.60163 | 3.273881 | 9.66562 | 2.68E-20 | 6.43E-19 | 35.2602 |
| TMEM165 | 0.60177 | 4.337806 | 11.7374 | 4.08E-28 | 2.89E-26 | 53.0335 |
| HIP1 | 0.60179 | 5.832806 | 8.39531 | 5.37E-16 | 7.73E-15 | 25.5094 |
| KNSTRN | 0.60189 | 3.436277 | 11.3816 | 1.04E-26 | 5.89E-25 | 49.8361 |
| LXN | 0.60216 | 2.397856 | 8.16429 | 2.92E-15 | 3.90E-14 | 23.8435 |
| SIKE1 | 0.60235 | 4.208703 | 14.2304 | 1.54E-38 | 7.65E-36 | 76.7865 |
| POLQ | 0.6024 | 0.648036 | 12.3951 | 9.01E-31 | 9.26E-29 | 59.0824 |
| TMEM67 | 0.60257 | 2.192814 | 10.4096 | 5.32E-23 | 1.80E-21 | 41.3975 |
| N4BP2 | 0.60263 | 2.557105 | 11.1646 | 7.25E-26 | 3.65E-24 | 47.914 |
| IRAK4 | 0.60282 | 3.219607 | 14.7688 | 6.67E-41 | 6.41E-38 | 82.1727 |
| CEBPD | 0.60286 | 4.930612 | 5.46245 | 7.58E-08 | 4.30E-07 | 7.17922 |
| TXLNB | 0.60342 | 1.254665 | 6.76428 | 3.94E-11 | 3.34E-10 | 14.5285 |
| DNMT1 | 0.60358 | 4.79685 | 11.0204 | 2.61E-25 | 1.22E-23 | 46.6481 |
| ITPRID2 | 0.60368 | 5.551467 | 10.9551 | 4.65E-25 | 2.12E-23 | 46.0779 |
| B3GALT5-AS1 | 0.60403 | 1.704369 | 8.27873 | 1.27E-15 | 1.75E-14 | 24.6644 |
| MCM7 | 0.60424 | 6.427643 | 9.09598 | 2.56E-18 | 4.82E-17 | 30.7682 |
| SPC25 | 0.60438 | 2.757687 | 5.48335 | 6.79E-08 | 3.88E-07 | 7.28649 |
| WDR45P1 | 0.60445 | 1.037246 | 9.00592 | 5.17E-18 | 9.45E-17 | 30.0754 |
| FRMD8 | 0.60449 | 4.794587 | 13.6316 | 5.92E-36 | 1.63E-33 | 70.8906 |
| CTPS1 | 0.60479 | 3.763824 | 10.8156 | 1.59E-24 | 6.65E-23 | 44.8672 |
| FAM91A1 | 0.60487 | 4.309839 | 13.9008 | 4.12E-37 | 1.38E-34 | 73.5282 |
| HSD3B7 | 0.60514 | 2.615525 | 9.46712 | 1.34E-19 | 2.94E-18 | 33.6737 |
| PPIC | 0.60548 | 3.179155 | 7.14073 | 3.50E-12 | 3.35E-11 | 16.8963 |
| CTDSP2 | 0.60555 | 6.725789 | 11.4817 | 4.20E-27 | 2.53E-25 | 50.73 |
| TMEM37 | 0.60566 | 2.231503 | 8.98545 | 6.07E-18 | 1.10E-16 | 29.9186 |
| ZNF644 | 0.60567 | 4.356714 | 12.7417 | 3.35E-32 | 4.49E-30 | 62.3384 |
| SLCO2B1 | 0.60592 | 5.515398 | 9.19023 | 1.22E-18 | 2.38E-17 | 31.4984 |
| SHMT1 | 0.60597 | 3.071937 | 11.5469 | 2.32E-27 | 1.46E-25 | 51.3151 |
| DAB2 | 0.60615 | 4.043559 | 8.50759 | 2.32E-16 | 3.49E-15 | 26.3315 |
| SOWAHC | 0.60648 | 3.467087 | 10.5496 | 1.60E-23 | 5.79E-22 | 42.5845 |
| EPB41 | 0.60666 | 3.589749 | 8.19998 | 2.26E-15 | 3.04E-14 | 24.0986 |
| HLA-F | 0.60682 | 3.937702 | 7.50458 | 3.06E-13 | 3.30E-12 | 19.2824 |
| XPR1 | 0.60746 | 4.72983 | 11.9646 | 5.03E-29 | 4.03E-27 | 55.1033 |
| ARPC5 | 0.60753 | 4.918664 | 15.0112 | 5.62E-42 | 8.11E-39 | 84.6221 |
| RBM43 | 0.60755 | 3.129487 | 11.0166 | 2.70E-25 | 1.26E-23 | 46.6148 |
| COL22A1 | 0.60772 | 1.802242 | 4.58306 | 5.86E-06 | 2.58E-05 | 2.99936 |
| PRPS2 | 0.60794 | 3.300092 | 6.15184 | 1.63E-09 | 1.14E-08 | 10.9048 |
| ADAMTS7 | 0.608 | 1.157024 | 8.61934 | 1.00E-16 | 1.58E-15 | 27.1576 |
| POLE2 | 0.60816 | 1.508822 | 11.4126 | 7.84E-27 | 4.57E-25 | 50.1122 |
| TMC8 | 0.60826 | 2.505551 | 8.51045 | 2.28E-16 | 3.42E-15 | 26.3525 |
| AC145098.1 | 0.6083 | 1.374783 | 9.82668 | 7.15E-21 | 1.85E-19 | 36.5637 |
| AC010615.1 | 0.60846 | 2.211315 | 9.48474 | 1.16E-19 | 2.58E-18 | 33.8137 |
| MAD2L2 | 0.60935 | 5.287246 | 10.2566 | 1.96E-22 | 6.15E-21 | 40.1118 |
| TMEM156 | 0.60954 | 2.185486 | 7.83337 | 3.12E-14 | 3.74E-13 | 21.5186 |
| KIAA0355 | 0.60958 | 4.097956 | 12.8355 | 1.37E-32 | 1.93E-30 | 63.2267 |
| RGS18 | 0.60979 | 1.928697 | 8.65571 | 7.62E-17 | 1.21E-15 | 27.4281 |
| TRIP10 | 0.61037 | 3.736438 | 8.67105 | 6.79E-17 | 1.09E-15 | 27.5425 |
| ZNF260 | 0.61053 | 4.22403 | 12.3567 | 1.29E-30 | 1.29E-28 | 58.7248 |
| GALNT1 | 0.61068 | 5.633038 | 13.5961 | 8.40E-36 | 2.23E-33 | 70.5439 |
| GMFG | 0.61082 | 4.392773 | 8.94778 | 8.13E-18 | 1.46E-16 | 29.6307 |
| DEPP1 | 0.61115 | 5.031157 | 6.1212 | 1.94E-09 | 1.35E-08 | 10.7311 |
| IGDCC4 | 0.61116 | 3.749735 | 6.01268 | 3.64E-09 | 2.43E-08 | 10.122 |
| SCPEP1 | 0.61129 | 4.680401 | 11.0504 | 2.00E-25 | 9.54E-24 | 46.9106 |
| SLC40A1 | 0.61149 | 4.789931 | 7.95531 | 1.32E-14 | 1.64E-13 | 22.3667 |
| PLIN3 | 0.61163 | 6.107386 | 8.4935 | 2.58E-16 | 3.86E-15 | 26.2278 |
| RPN2 | 0.61167 | 7.785744 | 14.0196 | 1.26E-37 | 4.78E-35 | 74.699 |
| SPN | 0.61177 | 1.429818 | 9.96456 | 2.28E-21 | 6.29E-20 | 37.6908 |
| SLFN12 | 0.6119 | 1.320677 | 11.0587 | 1.86E-25 | 8.91E-24 | 46.9833 |
| MIR10B | 0.61195 | 0.77298 | 6.32873 | 5.72E-10 | 4.24E-09 | 11.9217 |
| ODF2 | 0.61233 | 4.298456 | 13.9847 | 1.79E-37 | 6.50E-35 | 74.354 |
| GALNT2 | 0.61256 | 5.264163 | 12.3645 | 1.20E-30 | 1.20E-28 | 58.7979 |
| GNL2 | 0.61265 | 4.262762 | 14.2475 | 1.29E-38 | 6.56E-36 | 76.9566 |
| BACH1 | 0.61276 | 3.829978 | 12.8374 | 1.34E-32 | 1.90E-30 | 63.2449 |
| MCM6 | 0.61284 | 4.629359 | 10.3096 | 1.25E-22 | 4.02E-21 | 40.5562 |
| H2AX | 0.61316 | 6.661801 | 9.97275 | 2.13E-21 | 5.90E-20 | 37.758 |
| TLR8 | 0.61356 | 0.892369 | 9.90331 | 3.79E-21 | 1.02E-19 | 37.1888 |
| NKIRAS2 | 0.61362 | 4.671914 | 13.0662 | 1.48E-33 | 2.46E-31 | 65.426 |
| KDM4A | 0.61385 | 4.989873 | 12.5238 | 2.67E-31 | 3.01E-29 | 60.2866 |
| ZNF300 | 0.61449 | 3.758576 | 7.76022 | 5.22E-14 | 6.10E-13 | 21.0147 |
| HELLS | 0.61462 | 1.360265 | 10.8192 | 1.54E-24 | 6.46E-23 | 44.899 |
| CKLF | 0.61472 | 3.912982 | 14.1015 | 5.58E-38 | 2.32E-35 | 75.5081 |
| PROSER3 | 0.61483 | 2.259628 | 11.0073 | 2.93E-25 | 1.36E-23 | 46.5336 |
| ZDHHC12 | 0.61497 | 3.217017 | 8.70182 | 5.37E-17 | 8.74E-16 | 27.7724 |
| SPATA13 | 0.61506 | 4.088756 | 10.625 | 8.33E-24 | 3.14E-22 | 43.2287 |
| TTYH3 | 0.61506 | 6.328224 | 8.67387 | 6.64E-17 | 1.07E-15 | 27.5636 |
| NKG7 | 0.61513 | 1.769232 | 7.40779 | 5.90E-13 | 6.13E-12 | 18.6385 |
| SIPA1 | 0.61543 | 4.634404 | 9.45403 | 1.49E-19 | 3.25E-18 | 33.5699 |
| MSTN | 0.61554 | 3.616937 | 3.62922 | 0.00032 | 0.00106 | -0.77 |
| IGFBP7-AS1 | 0.61571 | 1.880959 | 6.11539 | 2.01E-09 | 1.39E-08 | 10.6983 |
| ERCC6L | 0.6162 | 0.681118 | 12.8204 | 1.58E-32 | 2.22E-30 | 63.0836 |
| TFRC | 0.61639 | 4.53302 | 7.7411 | 5.97E-14 | 6.93E-13 | 20.8836 |
| FCGRT | 0.61669 | 6.200137 | 9.84394 | 6.20E-21 | 1.62E-19 | 36.7042 |
| TAP1 | 0.61704 | 5.311425 | 8.12118 | 4.00E-15 | 5.26E-14 | 23.5364 |
| HAUS5 | 0.61704 | 3.584366 | 11.328 | 1.68E-26 | 9.33E-25 | 49.3592 |
| NUP210 | 0.61712 | 3.617707 | 6.51362 | 1.87E-10 | 1.47E-09 | 13.0106 |
| TOR4A | 0.61794 | 2.324142 | 10.8122 | 1.63E-24 | 6.82E-23 | 44.8383 |
| ADAM28 | 0.6182 | 2.603315 | 7.37178 | 7.52E-13 | 7.72E-12 | 18.4006 |
| GBP5 | 0.61826 | 0.684971 | 9.11982 | 2.12E-18 | 4.02E-17 | 30.9524 |
| STAC3 | 0.61827 | 2.769661 | 10.2806 | 1.60E-22 | 5.07E-21 | 40.3128 |
| MPZL1 | 0.6184 | 5.660903 | 11.1165 | 1.11E-25 | 5.50E-24 | 47.4907 |
| RFC3 | 0.61842 | 3.483987 | 11.0281 | 2.44E-25 | 1.15E-23 | 46.7158 |
| TGFB1I1 | 0.61876 | 3.110305 | 8.70198 | 5.36E-17 | 8.73E-16 | 27.7736 |
| RP2 | 0.61904 | 4.133571 | 10.7543 | 2.71E-24 | 1.10E-22 | 44.3383 |
| AC092675.1 | 0.6192 | 1.665947 | 8.32999 | 8.70E-16 | 1.23E-14 | 25.0348 |
| RNFT1 | 0.61922 | 2.969169 | 13.1706 | 5.38E-34 | 1.01E-31 | 66.4264 |
| GATM | 0.61926 | 6.287011 | 5.87902 | 7.78E-09 | 5.00E-08 | 9.38465 |
| IGFBP7 | 0.61946 | 8.528315 | 8.02103 | 8.22E-15 | 1.05E-13 | 22.8281 |
| CCN4 | 0.61968 | 0.877769 | 6.79653 | 3.22E-11 | 2.76E-10 | 14.7273 |
| F3 | 0.61979 | 5.806918 | 4.91715 | 1.21E-06 | 5.87E-06 | 4.50966 |
| MAN1C1 | 0.62002 | 3.700411 | 5.99343 | 4.06E-09 | 2.70E-08 | 10.0149 |
| GAB3 | 0.62024 | 2.123369 | 11.1533 | 8.02E-26 | 4.03E-24 | 47.8145 |
| GRN | 0.6204 | 7.011392 | 11.2314 | 3.99E-26 | 2.09E-24 | 48.5036 |
| PLOD1 | 0.62041 | 5.656816 | 11.4229 | 7.14E-27 | 4.21E-25 | 50.2048 |
| CXCL11 | 0.62081 | 0.882529 | 6.81288 | 2.90E-11 | 2.50E-10 | 14.8284 |
| PKIB | 0.62081 | 1.923562 | 5.80987 | 1.15E-08 | 7.20E-08 | 9.00879 |
| LAMA4 | 0.62088 | 3.485331 | 7.58685 | 1.74E-13 | 1.92E-12 | 19.8349 |
| LAMA2 | 0.6212 | 2.624251 | 7.09318 | 4.78E-12 | 4.51E-11 | 16.5915 |
| STK17A | 0.62158 | 4.574098 | 10.5183 | 2.09E-23 | 7.46E-22 | 42.3188 |
| ZNF736 | 0.62178 | 2.862204 | 12.757 | 2.90E-32 | 3.92E-30 | 62.4828 |
| CHRNA1 | 0.62187 | 0.941423 | 5.6662 | 2.53E-08 | 1.52E-07 | 8.24024 |
| LAMB2 | 0.62224 | 6.100903 | 9.687 | 2.25E-20 | 5.44E-19 | 35.4324 |
| KDELR1 | 0.6224 | 6.831885 | 13.0798 | 1.30E-33 | 2.22E-31 | 65.5558 |
| WAS | 0.62244 | 4.218359 | 8.20499 | 2.17E-15 | 2.94E-14 | 24.1344 |
| PIM1 | 0.62252 | 4.360932 | 8.7094 | 5.07E-17 | 8.27E-16 | 27.8291 |
| PARP4 | 0.62267 | 4.584641 | 11.5888 | 1.59E-27 | 1.02E-25 | 51.6917 |
| RNASE3 | 0.62276 | 1.357713 | 7.33648 | 9.53E-13 | 9.69E-12 | 18.1683 |
| CCDC80 | 0.62278 | 5.262141 | 6.02921 | 3.31E-09 | 2.23E-08 | 10.2142 |
| CITED1 | 0.62332 | 3.693564 | 5.61845 | 3.28E-08 | 1.95E-07 | 7.98853 |
| GMIP | 0.62351 | 3.989402 | 11.1012 | 1.28E-25 | 6.29E-24 | 47.3557 |
| ITGB8 | 0.6238 | 5.203904 | 7.4007 | 6.19E-13 | 6.41E-12 | 18.5916 |
| ANXA2R | 0.62394 | 2.070714 | 9.51681 | 8.98E-20 | 2.01E-18 | 34.0688 |
| GALNT3 | 0.62399 | 0.84062 | 8.93409 | 9.04E-18 | 1.61E-16 | 29.5263 |
| TSPAN6 | 0.62414 | 5.365466 | 10.919 | 6.39E-25 | 2.87E-23 | 45.7641 |
| WDR3 | 0.62416 | 3.665532 | 12.8183 | 1.61E-32 | 2.25E-30 | 63.0636 |
| SMIM3 | 0.62422 | 4.324812 | 7.88973 | 2.10E-14 | 2.55E-13 | 21.9094 |
| AC002456.1 | 0.62431 | 2.512223 | 7.84441 | 2.89E-14 | 3.47E-13 | 21.595 |
| CXCL16 | 0.62444 | 6.064917 | 9.02827 | 4.35E-18 | 7.97E-17 | 30.2468 |
| LINC01235 | 0.62459 | 2.261159 | 4.84396 | 1.72E-06 | 8.18E-06 | 4.17057 |
| AK2 | 0.62501 | 5.012318 | 13.1032 | 1.03E-33 | 1.81E-31 | 65.7801 |
| KCNQ1 | 0.62538 | 2.975961 | 8.89047 | 1.27E-17 | 2.22E-16 | 29.1944 |
| TICRR | 0.62542 | 1.488986 | 11.0209 | 2.60E-25 | 1.22E-23 | 46.6526 |
| ZNF45 | 0.6259 | 3.431457 | 13.017 | 2.38E-33 | 3.74E-31 | 64.9549 |
| CKS1B | 0.6261 | 3.25976 | 12.3962 | 8.91E-31 | 9.18E-29 | 59.0927 |
| CSRP2 | 0.6264 | 5.108624 | 5.49661 | 6.32E-08 | 3.63E-07 | 7.35471 |
| MUC1 | 0.62688 | 2.318378 | 8.8501 | 1.73E-17 | 2.98E-16 | 28.8883 |
| TGFB1 | 0.62694 | 5.350195 | 8.21751 | 1.98E-15 | 2.70E-14 | 24.2242 |
| NUAK2 | 0.62716 | 1.76262 | 8.79215 | 2.70E-17 | 4.53E-16 | 28.4506 |
| ANKRD10-IT1 | 0.62732 | 4.411486 | 7.42309 | 5.32E-13 | 5.57E-12 | 18.7399 |
| HHEX | 0.62734 | 2.567443 | 9.64774 | 3.10E-20 | 7.37E-19 | 35.1164 |
| KCNJ5 | 0.62751 | 2.208658 | 7.5852 | 1.76E-13 | 1.94E-12 | 19.8238 |
| PSMB8-AS1 | 0.62768 | 3.351854 | 8.53945 | 1.83E-16 | 2.79E-15 | 26.5661 |
| TIGAR | 0.62789 | 3.709077 | 11.4212 | 7.25E-27 | 4.27E-25 | 50.1895 |
| APOL1 | 0.62791 | 3.286158 | 6.78342 | 3.50E-11 | 2.98E-10 | 14.6464 |
| MARVELD1 | 0.62801 | 3.046783 | 7.18138 | 2.68E-12 | 2.59E-11 | 17.1582 |
| AC125807.2 | 0.6283 | 1.103347 | 8.50488 | 2.37E-16 | 3.56E-15 | 26.3115 |
| SLC16A1 | 0.62835 | 5.996538 | 11.0776 | 1.57E-25 | 7.63E-24 | 47.1493 |
| AC012073.1 | 0.62837 | 1.717554 | 11.9599 | 5.26E-29 | 4.18E-27 | 55.0608 |
| SLC25A43 | 0.62851 | 2.802187 | 6.78602 | 3.44E-11 | 2.94E-10 | 14.6625 |
| PRSS23 | 0.62865 | 2.598888 | 7.6233 | 1.35E-13 | 1.51E-12 | 20.0812 |
| CNPY4 | 0.62899 | 4.842122 | 12.4136 | 7.56E-31 | 7.93E-29 | 59.2557 |
| ZNF480 | 0.62905 | 3.265175 | 14.5622 | 5.43E-40 | 3.96E-37 | 80.0966 |
| CCNE2 | 0.62911 | 2.177713 | 10.1245 | 5.98E-22 | 1.77E-20 | 39.0112 |
| YBX1 | 0.62912 | 8.970929 | 10.3236 | 1.11E-22 | 3.59E-21 | 40.6738 |
| SLC35D1 | 0.62914 | 3.31869 | 12.2989 | 2.23E-30 | 2.12E-28 | 58.1874 |
| GIMAP4 | 0.62919 | 4.797087 | 8.62519 | 9.60E-17 | 1.51E-15 | 27.2011 |
| NECAP2 | 0.62944 | 5.185998 | 11.185 | 6.05E-26 | 3.08E-24 | 48.0939 |
| ITPKC | 0.62954 | 4.337201 | 9.68919 | 2.21E-20 | 5.34E-19 | 35.4501 |
| KCTD12 | 0.62957 | 6.218193 | 7.69942 | 7.98E-14 | 9.13E-13 | 20.5986 |
| CHAF1B | 0.62965 | 1.800795 | 7.94148 | 1.45E-14 | 1.80E-13 | 22.2701 |
| FANCA | 0.62973 | 1.487022 | 10.7291 | 3.37E-24 | 1.35E-22 | 44.1212 |
| RTKN2 | 0.6298 | 1.201509 | 10.1236 | 6.02E-22 | 1.78E-20 | 39.0035 |
| HPGDS | 0.63013 | 2.98466 | 7.01223 | 8.09E-12 | 7.45E-11 | 16.0764 |
| TMPO | 0.63021 | 4.503526 | 11.6735 | 7.33E-28 | 5.00E-26 | 52.4549 |
| PLAC8 | 0.63032 | 0.835576 | 8.84017 | 1.87E-17 | 3.20E-16 | 28.8131 |
| RAD18 | 0.63045 | 2.585541 | 13.518 | 1.81E-35 | 4.49E-33 | 69.7841 |
| POU3F2 | 0.63049 | 5.369563 | 9.74865 | 1.36E-20 | 3.38E-19 | 35.9304 |
| B4GALT5 | 0.63104 | 6.126581 | 11.6032 | 1.39E-27 | 9.08E-26 | 51.8213 |
| FAM20A | 0.63109 | 1.143957 | 9.03186 | 4.23E-18 | 7.77E-17 | 30.2744 |
| PDGFA | 0.63116 | 4.277021 | 6.47993 | 2.30E-10 | 1.78E-09 | 12.8103 |
| CARD8 | 0.63135 | 3.246908 | 13.6816 | 3.62E-36 | 1.04E-33 | 71.3786 |
| NSUN7 | 0.63141 | 0.70158 | 9.46167 | 1.40E-19 | 3.07E-18 | 33.6305 |
| TMX1 | 0.63186 | 5.247803 | 13.1194 | 8.84E-34 | 1.57E-31 | 65.9359 |
| KCNK13 | 0.63189 | 1.923852 | 8.82232 | 2.14E-17 | 3.64E-16 | 28.6781 |
| TCIRG1 | 0.63193 | 3.487241 | 8.24343 | 1.64E-15 | 2.25E-14 | 24.4102 |
| MYO1E | 0.63194 | 3.257319 | 9.21241 | 1.02E-18 | 2.02E-17 | 31.671 |
| DNAAF5 | 0.63197 | 3.84784 | 14.5634 | 5.36E-40 | 3.96E-37 | 80.1085 |
| LILRA1 | 0.63199 | 1.699908 | 9.00373 | 5.26E-18 | 9.61E-17 | 30.0586 |
| OSTC | 0.63236 | 5.598016 | 15.0681 | 3.14E-42 | 4.92E-39 | 85.1995 |
| C6orf118 | 0.63245 | 1.275695 | 7.48793 | 3.42E-13 | 3.67E-12 | 19.1712 |
| AC090559.1 | 0.63297 | 2.765572 | 7.48927 | 3.39E-13 | 3.64E-12 | 19.1802 |
| ABHD15 | 0.63299 | 3.165905 | 12.7206 | 4.10E-32 | 5.36E-30 | 62.1387 |
| ZIK1 | 0.63307 | 2.996971 | 12.3426 | 1.48E-30 | 1.46E-28 | 58.5938 |
| ATL3 | 0.63353 | 4.602933 | 11.0972 | 1.32E-25 | 6.50E-24 | 47.3206 |
| SFT2D2 | 0.63364 | 4.249114 | 12.8649 | 1.03E-32 | 1.50E-30 | 63.506 |
| LILRA2 | 0.63376 | 1.938511 | 8.32964 | 8.72E-16 | 1.23E-14 | 25.0323 |
| DSCC1 | 0.63394 | 2.930166 | 12.6488 | 8.13E-32 | 9.95E-30 | 61.4617 |
| KLHL6 | 0.63403 | 1.467901 | 9.93835 | 2.83E-21 | 7.73E-20 | 37.4757 |
| BMP1 | 0.63411 | 3.803299 | 11.9655 | 4.99E-29 | 4.00E-27 | 55.1113 |
| C8orf88 | 0.63412 | 2.046367 | 8.53121 | 1.95E-16 | 2.96E-15 | 26.5054 |
| PRKAR1B-AS1 | 0.63418 | 1.581204 | 9.22443 | 9.30E-19 | 1.84E-17 | 31.7647 |
| CTHRC1 | 0.63434 | 1.786824 | 6.45834 | 2.62E-10 | 2.02E-09 | 12.6823 |
| LITAF | 0.6345 | 5.470438 | 7.6459 | 1.16E-13 | 1.30E-12 | 20.2344 |
| GUSB | 0.63491 | 4.783109 | 12.622 | 1.05E-31 | 1.26E-29 | 61.2086 |
| ELF1 | 0.63494 | 4.569109 | 11.5987 | 1.45E-27 | 9.42E-26 | 51.7806 |
| PTK7 | 0.63498 | 3.791052 | 10.4527 | 3.68E-23 | 1.28E-21 | 41.762 |
| C18orf54 | 0.63511 | 2.227421 | 11.8704 | 1.20E-28 | 9.19E-27 | 54.2425 |
| HCST | 0.63524 | 3.661806 | 6.55198 | 1.48E-10 | 1.18E-09 | 13.2399 |
| TLR6 | 0.63524 | 1.507373 | 10.3621 | 7.99E-23 | 2.64E-21 | 40.9972 |
| SEC24D | 0.63568 | 2.35137 | 10.6071 | 9.73E-24 | 3.65E-22 | 43.0754 |
| AL662791.1 | 0.63575 | 1.597344 | 10.2709 | 1.74E-22 | 5.48E-21 | 40.2315 |
| LRRC42 | 0.63576 | 4.550684 | 12.5202 | 2.76E-31 | 3.10E-29 | 60.2529 |
| NASP | 0.63635 | 5.475785 | 11.9154 | 7.93E-29 | 6.20E-27 | 54.6537 |
| PARPBP | 0.6367 | 1.123983 | 13.7685 | 1.53E-36 | 4.69E-34 | 72.229 |
| H3C10 | 0.63717 | 1.04166 | 9.92046 | 3.29E-21 | 8.91E-20 | 37.3292 |
| RTTN | 0.6374 | 2.553619 | 13.1382 | 7.37E-34 | 1.34E-31 | 66.1159 |
| WASF2 | 0.63757 | 6.289113 | 11.3847 | 1.01E-26 | 5.74E-25 | 49.8641 |
| GBE1 | 0.6378 | 4.040874 | 11.5285 | 2.75E-27 | 1.71E-25 | 51.1494 |
| CCDC89 | 0.6379 | 1.659654 | 8.03793 | 7.28E-15 | 9.31E-14 | 22.9471 |
| USP1 | 0.63793 | 5.021592 | 13.0492 | 1.74E-33 | 2.83E-31 | 65.2634 |
| HOXA6 | 0.63821 | 0.370708 | 8.03842 | 7.26E-15 | 9.28E-14 | 22.9505 |
| HS3ST3B1 | 0.6383 | 0.922992 | 6.66699 | 7.25E-11 | 5.98E-10 | 13.9338 |
| AC025176.1 | 0.63833 | 0.61378 | 10.3162 | 1.18E-22 | 3.82E-21 | 40.6115 |
| KIF20B | 0.63862 | 1.848175 | 13.4767 | 2.71E-35 | 6.47E-33 | 69.3834 |
| LIMD1 | 0.63871 | 2.840524 | 8.17444 | 2.72E-15 | 3.63E-14 | 23.9159 |
| SBNO2 | 0.63885 | 3.755338 | 11.4697 | 4.68E-27 | 2.80E-25 | 50.6224 |
| MIR3151 | 0.63886 | 2.708849 | 5.46092 | 7.64E-08 | 4.33E-07 | 7.17138 |
| DDOST | 0.63888 | 6.981539 | 14.4786 | 1.26E-39 | 7.86E-37 | 79.2602 |
| ZNF528 | 0.63894 | 3.094803 | 11.7828 | 2.69E-28 | 1.95E-26 | 53.4451 |
| CCDC77 | 0.63903 | 3.129268 | 13.7317 | 2.21E-36 | 6.60E-34 | 71.8685 |
| KDM5A | 0.6393 | 3.803689 | 11.5531 | 2.20E-27 | 1.38E-25 | 51.3706 |
| PRMT6 | 0.63937 | 4.445092 | 13.9885 | 1.72E-37 | 6.30E-35 | 74.3917 |
| GNLY | 0.63982 | 1.273646 | 7.39262 | 6.53E-13 | 6.75E-12 | 18.5382 |
| ITGAM | 0.63994 | 3.19224 | 7.20336 | 2.31E-12 | 2.26E-11 | 17.3003 |
| FZD7 | 0.64033 | 3.887612 | 5.4908 | 6.52E-08 | 3.74E-07 | 7.32482 |
| IGHG1 | 0.64065 | 1.266872 | 4.00092 | 7.32E-05 | 0.000272 | 0.60172 |
| BLM | 0.64076 | 2.755648 | 6.07836 | 2.49E-09 | 1.70E-08 | 10.4895 |
| AC108463.1 | 0.64091 | 0.908222 | 8.60694 | 1.10E-16 | 1.72E-15 | 27.0655 |
| RCAN1 | 0.64097 | 5.342708 | 10.3472 | 9.07E-23 | 2.98E-21 | 40.8717 |
| ZNF468 | 0.64143 | 2.268021 | 12.8167 | 1.64E-32 | 2.28E-30 | 63.0481 |
| AGTRAP | 0.64147 | 4.900523 | 8.97427 | 6.62E-18 | 1.20E-16 | 29.8331 |
| CPM | 0.64156 | 3.520065 | 7.12508 | 3.87E-12 | 3.70E-11 | 16.7958 |
| FXYD5 | 0.64164 | 4.216135 | 7.78696 | 4.33E-14 | 5.10E-13 | 21.1985 |
| BORA | 0.6417 | 1.65134 | 15.9164 | 4.87E-46 | 2.43E-42 | 93.8872 |
| HAS2-AS1 | 0.64181 | 2.079634 | 9.86382 | 5.26E-21 | 1.39E-19 | 36.8662 |
| OR2I1P | 0.64192 | 0.93174 | 7.97409 | 1.15E-14 | 1.44E-13 | 22.4983 |
| OSR2 | 0.64194 | 0.516464 | 9.05492 | 3.53E-18 | 6.55E-17 | 30.4517 |
| CFH | 0.64211 | 2.297503 | 7.70256 | 7.81E-14 | 8.94E-13 | 20.62 |
| STAC | 0.6423 | 0.740868 | 6.31504 | 6.20E-10 | 4.58E-09 | 11.8422 |
| AL512785.1 | 0.64238 | 0.845699 | 7.05776 | 6.02E-12 | 5.63E-11 | 16.3655 |
| PPP1R18 | 0.64247 | 5.543133 | 11.2452 | 3.53E-26 | 1.87E-24 | 48.6255 |
| HMGN2 | 0.64271 | 7.279494 | 12.0804 | 1.72E-29 | 1.46E-27 | 56.1664 |
| TNFAIP3 | 0.64285 | 2.765818 | 9.04398 | 3.84E-18 | 7.10E-17 | 30.3675 |
| MYBPH | 0.64322 | 0.882216 | 8.37455 | 6.26E-16 | 8.95E-15 | 25.3582 |
| DHFR | 0.64368 | 3.846521 | 9.68159 | 2.35E-20 | 5.67E-19 | 35.3888 |
| ARHGAP18 | 0.64406 | 2.915372 | 8.35464 | 7.25E-16 | 1.03E-14 | 25.2136 |
| YBX1P10 | 0.64434 | 3.072662 | 10.4623 | 3.39E-23 | 1.19E-21 | 41.8434 |
| ZC3HAV1L | 0.64442 | 1.315215 | 8.86927 | 1.49E-17 | 2.59E-16 | 29.0335 |
| ALG6 | 0.64481 | 2.872595 | 14.0307 | 1.13E-37 | 4.34E-35 | 74.8084 |
| RFC2 | 0.64496 | 5.197461 | 13.5698 | 1.09E-35 | 2.83E-33 | 70.2886 |
| HOXB3 | 0.64506 | 0.480432 | 6.95519 | 1.17E-11 | 1.05E-10 | 15.7163 |
| PCDHB7 | 0.64561 | 2.357128 | 5.55036 | 4.74E-08 | 2.76E-07 | 7.63286 |
| LRRK2 | 0.64575 | 2.574042 | 7.63408 | 1.26E-13 | 1.41E-12 | 20.1542 |
| PLCB2 | 0.64586 | 3.552286 | 7.80653 | 3.77E-14 | 4.47E-13 | 21.3333 |
| GSC | 0.64608 | 1.772439 | 6.97009 | 1.06E-11 | 9.62E-11 | 15.8101 |
| KLF6 | 0.64639 | 5.119543 | 9.65944 | 2.82E-20 | 6.73E-19 | 35.2105 |
| H19 | 0.64678 | 0.744637 | 4.98975 | 8.49E-07 | 4.22E-06 | 4.85058 |
| EN2 | 0.64695 | 1.25664 | 7.59388 | 1.66E-13 | 1.83E-12 | 19.8824 |
| SINHCAF | 0.64708 | 3.407478 | 7.9768 | 1.13E-14 | 1.42E-13 | 22.5173 |
| IMPACT | 0.64723 | 4.580564 | 8.05889 | 6.27E-15 | 8.08E-14 | 23.0951 |
| SUSD3 | 0.64739 | 3.433136 | 6.41885 | 3.33E-10 | 2.53E-09 | 12.4492 |
| PSMB9 | 0.6475 | 4.233944 | 7.81638 | 3.52E-14 | 4.20E-13 | 21.4013 |
| ORC6 | 0.64762 | 2.041639 | 9.28935 | 5.56E-19 | 1.13E-17 | 32.272 |
| NADK | 0.64786 | 4.799937 | 13.3707 | 7.66E-35 | 1.68E-32 | 68.356 |
| PLD4 | 0.64803 | 3.472455 | 6.35105 | 5.00E-10 | 3.73E-09 | 12.0518 |
| SOCS6 | 0.64886 | 4.583408 | 10.7942 | 1.91E-24 | 7.94E-23 | 44.6822 |
| IRF5 | 0.64886 | 3.210434 | 8.56933 | 1.46E-16 | 2.25E-15 | 26.7869 |
| C5orf34 | 0.6493 | 1.629714 | 13.0095 | 2.56E-33 | 3.98E-31 | 64.884 |
| PXDN | 0.64933 | 3.809472 | 6.56482 | 1.37E-10 | 1.09E-09 | 13.3168 |
| SRGN | 0.64996 | 6.947666 | 7.80183 | 3.90E-14 | 4.62E-13 | 21.3009 |
| ADGRE2 | 0.65033 | 0.986747 | 10.6299 | 7.99E-24 | 3.02E-22 | 43.2703 |
| RHOC | 0.65035 | 6.786233 | 9.58449 | 5.19E-20 | 1.20E-18 | 34.6091 |
| CTSZ | 0.65047 | 6.472376 | 8.30082 | 1.08E-15 | 1.50E-14 | 24.8238 |
| TLR10 | 0.65062 | 1.504135 | 9.17886 | 1.33E-18 | 2.59E-17 | 31.41 |
| IL10RB | 0.651 | 4.459892 | 14.7968 | 5.01E-41 | 5.09E-38 | 82.455 |
| C1orf112 | 0.65103 | 1.856325 | 16.1929 | 2.71E-47 | 1.65E-43 | 96.75 |
| FILIP1L | 0.65139 | 2.870133 | 7.25963 | 1.59E-12 | 1.58E-11 | 17.6657 |
| RB1 | 0.65158 | 5.170111 | 12.5533 | 2.02E-31 | 2.33E-29 | 60.5632 |
| ZYX | 0.65193 | 6.832735 | 10.0675 | 9.64E-22 | 2.78E-20 | 38.5393 |
| AL139393.3 | 0.652 | 4.0552 | 6.81972 | 2.78E-11 | 2.40E-10 | 14.8707 |
| DDIT4L | 0.65219 | 1.495223 | 4.81858 | 1.95E-06 | 9.17E-06 | 4.05406 |
| SLC39A1 | 0.65262 | 6.239103 | 14.8834 | 2.07E-41 | 2.37E-38 | 83.329 |
| PTPRZ1 | 0.65266 | 9.027271 | 6.87595 | 1.94E-11 | 1.71E-10 | 15.22 |
| JUN | 0.65267 | 7.275427 | 7.40304 | 6.09E-13 | 6.32E-12 | 18.6071 |
| CHST6 | 0.65402 | 2.750614 | 6.22946 | 1.03E-09 | 7.39E-09 | 11.348 |
| ITGB3BP | 0.6543 | 2.357006 | 12.6993 | 5.03E-32 | 6.44E-30 | 61.9373 |
| BTN3A1 | 0.65431 | 4.13499 | 10.3719 | 7.35E-23 | 2.44E-21 | 41.0798 |
| RGS10 | 0.65432 | 5.808714 | 6.98934 | 9.38E-12 | 8.57E-11 | 15.9316 |
| CREB3L2 | 0.65456 | 3.928822 | 13.6853 | 3.49E-36 | 1.01E-33 | 71.4152 |
| IL4I1 | 0.65463 | 1.360847 | 8.69238 | 5.77E-17 | 9.35E-16 | 27.7018 |
| GNAI3 | 0.65474 | 3.337705 | 14.378 | 3.48E-39 | 1.97E-36 | 78.2551 |
| SLC2A5 | 0.65485 | 4.284671 | 6.21339 | 1.13E-09 | 8.07E-09 | 11.2558 |
| GZMA | 0.65506 | 1.121184 | 7.35963 | 8.16E-13 | 8.34E-12 | 18.3206 |
| MCM3 | 0.65533 | 5.674403 | 13.462 | 3.14E-35 | 7.38E-33 | 69.2407 |
| SIPA1L2 | 0.65545 | 4.941777 | 11.082 | 1.51E-25 | 7.37E-24 | 47.1873 |
| PTGES3P1 | 0.65592 | 4.233253 | 9.04894 | 3.70E-18 | 6.84E-17 | 30.4057 |
| IKZF1 | 0.65595 | 2.087596 | 8.89345 | 1.24E-17 | 2.17E-16 | 29.217 |
| TEX26 | 0.65597 | 1.349858 | 5.82731 | 1.04E-08 | 6.57E-08 | 9.1032 |
| MCM4 | 0.6563 | 4.814891 | 9.90739 | 3.66E-21 | 9.86E-20 | 37.2222 |
| H2AC11 | 0.65645 | 0.974113 | 10.5722 | 1.32E-23 | 4.82E-22 | 42.7778 |
| RBMS1 | 0.65662 | 2.529808 | 10.5848 | 1.18E-23 | 4.37E-22 | 42.8846 |
| MYL12A | 0.65689 | 5.106896 | 9.30246 | 5.01E-19 | 1.02E-17 | 32.3748 |
| BATF | 0.65691 | 1.28247 | 8.52951 | 1.97E-16 | 2.99E-15 | 26.4929 |
| CCDC102A | 0.65737 | 2.814271 | 10.8786 | 9.12E-25 | 4.00E-23 | 45.413 |
| PTGER4 | 0.65746 | 1.383646 | 9.20209 | 1.11E-18 | 2.18E-17 | 31.5906 |
| EHD4 | 0.65755 | 3.238804 | 9.56435 | 6.11E-20 | 1.41E-18 | 34.4481 |
| RDX | 0.65764 | 6.599137 | 12.2954 | 2.30E-30 | 2.18E-28 | 58.1545 |
| BRCA2 | 0.65765 | 0.820383 | 13.9835 | 1.81E-37 | 6.53E-35 | 74.3425 |
| PARP12 | 0.65767 | 2.847119 | 8.76168 | 3.40E-17 | 5.67E-16 | 28.2212 |
| PIK3CG | 0.65776 | 1.224474 | 10.1738 | 3.95E-22 | 1.20E-20 | 39.4208 |
| MEIS3P1 | 0.65778 | 2.408434 | 7.27345 | 1.45E-12 | 1.45E-11 | 17.7558 |
| COL28A1 | 0.65783 | 2.443182 | 4.79089 | 2.22E-06 | 1.04E-05 | 3.92757 |
| DCTD | 0.65821 | 4.836404 | 7.70009 | 7.94E-14 | 9.09E-13 | 20.6032 |
| FOSL1 | 0.65839 | 1.883727 | 6.4704 | 2.43E-10 | 1.88E-09 | 12.7537 |
| AL161785.1 | 0.65841 | 2.561024 | 7.94448 | 1.42E-14 | 1.76E-13 | 22.291 |
| CSF1R | 0.65874 | 6.517739 | 5.99402 | 4.05E-09 | 2.69E-08 | 10.0182 |
| GLA | 0.65882 | 4.207822 | 15.2381 | 5.48E-43 | 1.07E-39 | 86.9278 |
| ADGRE1 | 0.65885 | 0.71276 | 8.93985 | 8.64E-18 | 1.54E-16 | 29.5702 |
| BAZ1A | 0.65943 | 3.44833 | 11.2193 | 4.45E-26 | 2.32E-24 | 48.3959 |
| GRIK3 | 0.66057 | 5.381561 | 7.06553 | 5.72E-12 | 5.36E-11 | 16.415 |
| MMRN1 | 0.66117 | 1.349281 | 8.85505 | 1.66E-17 | 2.87E-16 | 28.9257 |
| E2F3 | 0.66123 | 4.012424 | 11.5322 | 2.65E-27 | 1.65E-25 | 51.183 |
| GNS | 0.6614 | 5.673419 | 11.3036 | 2.09E-26 | 1.14E-24 | 49.1425 |
| HOXA11 | 0.66163 | 0.589747 | 7.18556 | 2.60E-12 | 2.53E-11 | 17.1852 |
| FOS | 0.66174 | 7.074231 | 4.60643 | 5.27E-06 | 2.33E-05 | 3.10186 |
| TP53 | 0.66187 | 5.448271 | 8.85761 | 1.63E-17 | 2.82E-16 | 28.9451 |
| PTPN13 | 0.66201 | 4.482647 | 9.13998 | 1.81E-18 | 3.46E-17 | 31.1085 |
| E2F8 | 0.66209 | 0.758065 | 11.3855 | 1.00E-26 | 5.71E-25 | 49.8714 |
| HAUS1 | 0.6624 | 4.178325 | 14.7884 | 5.46E-41 | 5.44E-38 | 82.3703 |
| IL2RG | 0.6625 | 1.564897 | 9.46889 | 1.32E-19 | 2.90E-18 | 33.6878 |
| ZNF107 | 0.66256 | 3.082654 | 11.7519 | 3.58E-28 | 2.56E-26 | 53.1646 |
| CD300C | 0.66301 | 2.095778 | 8.91902 | 1.02E-17 | 1.80E-16 | 29.4115 |
| PYCARD | 0.66332 | 3.483998 | 7.77945 | 4.56E-14 | 5.36E-13 | 21.1468 |
| SIGLEC8 | 0.66393 | 4.322771 | 6.02854 | 3.32E-09 | 2.24E-08 | 10.2104 |
| BLNK | 0.66408 | 2.544005 | 7.52105 | 2.73E-13 | 2.96E-12 | 19.3927 |
| SNRPGP10 | 0.66412 | 2.268941 | 7.47616 | 3.71E-13 | 3.96E-12 | 19.0927 |
| HS3ST1 | 0.66488 | 2.571412 | 5.91405 | 6.38E-09 | 4.15E-08 | 9.57654 |
| H2BC5 | 0.66497 | 3.770878 | 7.60768 | 1.51E-13 | 1.67E-12 | 19.9756 |
| FKBP9 | 0.66503 | 4.455072 | 7.36529 | 7.85E-13 | 8.04E-12 | 18.3579 |
| WDFY4 | 0.66526 | 1.876494 | 9.62382 | 3.77E-20 | 8.86E-19 | 34.9243 |
| MAP7D3 | 0.66601 | 2.198215 | 11.6829 | 6.73E-28 | 4.63E-26 | 52.5401 |
| IL18BP | 0.66636 | 3.636484 | 11.1515 | 8.16E-26 | 4.09E-24 | 47.798 |
| NCF2 | 0.66661 | 3.259312 | 8.83868 | 1.89E-17 | 3.24E-16 | 28.8019 |
| PHACTR4 | 0.66671 | 4.273942 | 14.0386 | 1.05E-37 | 4.09E-35 | 74.8866 |
| ATF3 | 0.66752 | 3.483662 | 6.04795 | 2.97E-09 | 2.01E-08 | 10.3189 |
| AC022432.1 | 0.66766 | 1.27446 | 9.34373 | 3.60E-19 | 7.50E-18 | 32.6989 |
| TWSG1 | 0.66766 | 4.779488 | 10.591 | 1.12E-23 | 4.16E-22 | 42.9381 |
| KANK2 | 0.66783 | 4.299038 | 8.81022 | 2.35E-17 | 3.98E-16 | 28.5868 |
| LATS2 | 0.66823 | 2.18281 | 9.44488 | 1.60E-19 | 3.48E-18 | 33.4974 |
| DESI2 | 0.6686 | 4.270905 | 14.0337 | 1.10E-37 | 4.27E-35 | 74.8383 |
| SWAP70 | 0.66861 | 3.586007 | 9.39565 | 2.38E-19 | 5.06E-18 | 33.108 |
| LTBP1 | 0.66908 | 3.836655 | 6.71128 | 5.50E-11 | 4.60E-10 | 14.2037 |
| WLS | 0.66927 | 7.390286 | 9.07296 | 3.07E-18 | 5.71E-17 | 30.5906 |
| ADAM17 | 0.66963 | 4.443439 | 11.3048 | 2.07E-26 | 1.13E-24 | 49.1532 |
| RNASEH2A | 0.66979 | 4.832033 | 10.9937 | 3.31E-25 | 1.53E-23 | 46.4146 |
| NCF4 | 0.66986 | 3.516126 | 7.9465 | 1.40E-14 | 1.74E-13 | 22.3051 |
| H6PD | 0.67043 | 4.408004 | 12.7114 | 4.48E-32 | 5.80E-30 | 62.0517 |
| FAAP24 | 0.6706 | 1.698821 | 15.0541 | 3.62E-42 | 5.52E-39 | 85.0575 |
| OR4N2 | 0.67099 | 1.355673 | 4.96099 | 9.78E-07 | 4.81E-06 | 4.715 |
| HOXB2 | 0.67111 | 0.827656 | 6.90346 | 1.63E-11 | 1.45E-10 | 15.3918 |
| GINS4 | 0.67123 | 1.352755 | 14.1043 | 5.43E-38 | 2.29E-35 | 75.5358 |
| TNFSF8 | 0.67175 | 1.436495 | 9.98809 | 1.87E-21 | 5.23E-20 | 37.8842 |
| IRF7 | 0.67201 | 3.731115 | 7.75487 | 5.42E-14 | 6.32E-13 | 20.9779 |
| AC103923.1 | 0.67202 | 1.592327 | 9.46062 | 1.41E-19 | 3.09E-18 | 33.6221 |
| SH2D4A | 0.67212 | 0.941009 | 8.08023 | 5.37E-15 | 6.98E-14 | 23.246 |
| FNBP1L | 0.67259 | 4.42346 | 7.31628 | 1.09E-12 | 1.10E-11 | 18.0358 |
| PLXDC2 | 0.67268 | 4.135984 | 7.50352 | 3.08E-13 | 3.32E-12 | 19.2754 |
| IRF8 | 0.67285 | 3.375869 | 7.48025 | 3.61E-13 | 3.85E-12 | 19.12 |
| TFEC | 0.673 | 1.408266 | 11.0476 | 2.05E-25 | 9.75E-24 | 46.8866 |
| NAMPTP1 | 0.67322 | 1.617167 | 7.60904 | 1.49E-13 | 1.66E-12 | 19.9848 |
| BIN2 | 0.6735 | 2.939446 | 8.72378 | 4.54E-17 | 7.46E-16 | 27.9368 |
| ERI1 | 0.67363 | 3.024104 | 15.2908 | 3.19E-43 | 6.47E-40 | 87.4646 |
| PCED1B | 0.67363 | 2.226435 | 9.59388 | 4.81E-20 | 1.12E-18 | 34.6843 |
| RAB34 | 0.67378 | 3.441762 | 6.2197 | 1.09E-09 | 7.80E-09 | 11.292 |
| TMEM45A | 0.67389 | 2.319668 | 9.94669 | 2.64E-21 | 7.22E-20 | 37.5441 |
| NUP205 | 0.6742 | 4.598062 | 13.0777 | 1.32E-33 | 2.26E-31 | 65.5363 |
| TMED5 | 0.67433 | 4.474135 | 12.5814 | 1.54E-31 | 1.82E-29 | 60.8266 |
| AJUBA | 0.67454 | 2.011164 | 10.1935 | 3.34E-22 | 1.02E-20 | 39.5848 |
| APOL6 | 0.67459 | 3.262207 | 8.62965 | 9.28E-17 | 1.46E-15 | 27.2342 |
| HLA-C | 0.6754 | 8.689781 | 8.20143 | 2.23E-15 | 3.01E-14 | 24.109 |
| MNDA | 0.67541 | 3.751968 | 7.14254 | 3.46E-12 | 3.31E-11 | 16.9079 |
| MOB1A | 0.67592 | 5.202636 | 14.761 | 7.22E-41 | 6.82E-38 | 82.0946 |
| ZMYM1 | 0.67601 | 2.637341 | 14.6106 | 3.32E-40 | 2.64E-37 | 80.5821 |
| CCND2 | 0.67612 | 6.677452 | 7.75419 | 5.44E-14 | 6.35E-13 | 20.9733 |
| ADPRH | 0.67615 | 2.24536 | 11.4125 | 7.85E-27 | 4.57E-25 | 50.1118 |
| CSF2RB | 0.67626 | 1.560596 | 10.1584 | 4.49E-22 | 1.35E-20 | 39.2925 |
| NRAS | 0.67629 | 5.327374 | 14.5283 | 7.65E-40 | 5.30E-37 | 79.7571 |
| CHST14 | 0.67644 | 4.3409 | 13.4949 | 2.27E-35 | 5.48E-33 | 69.5596 |
| ENPEP | 0.67665 | 1.328918 | 9.29657 | 5.25E-19 | 1.07E-17 | 32.3286 |
| NEDD4 | 0.67683 | 2.666655 | 9.47043 | 1.30E-19 | 2.87E-18 | 33.7 |
| RNASET2 | 0.67684 | 4.43629 | 8.80349 | 2.47E-17 | 4.19E-16 | 28.536 |
| ANG | 0.67687 | 1.805968 | 8.69372 | 5.71E-17 | 9.26E-16 | 27.7118 |
| ACTL6A | 0.67753 | 4.604242 | 14.2759 | 9.72E-39 | 5.17E-36 | 77.2388 |
| GNB4 | 0.6782 | 4.942769 | 10.8369 | 1.31E-24 | 5.59E-23 | 45.0518 |
| CAVIN1 | 0.6788 | 5.539558 | 7.10535 | 4.41E-12 | 4.18E-11 | 16.6693 |
| TXLNA | 0.67914 | 5.307038 | 13.1607 | 5.92E-34 | 1.09E-31 | 66.3319 |
| MR1 | 0.67924 | 2.813797 | 10.0388 | 1.23E-21 | 3.49E-20 | 38.3018 |
| CDH11 | 0.67932 | 4.088063 | 11.7392 | 4.02E-28 | 2.85E-26 | 53.0502 |
| TRAM1 | 0.67934 | 6.118641 | 14.648 | 2.27E-40 | 1.95E-37 | 80.9578 |
| LOX | 0.67985 | 1.522183 | 8.45781 | 3.37E-16 | 4.98E-15 | 25.966 |
| CMTM7 | 0.67999 | 2.414416 | 9.52513 | 8.39E-20 | 1.89E-18 | 34.1351 |
| ADAM19 | 0.6803 | 2.262554 | 8.69663 | 5.59E-17 | 9.07E-16 | 27.7336 |
| CD48 | 0.68032 | 1.136844 | 8.13714 | 3.56E-15 | 4.71E-14 | 23.65 |
| TGIF2 | 0.68033 | 3.634607 | 10.1579 | 4.51E-22 | 1.36E-20 | 39.2885 |
| SNX20 | 0.68073 | 1.179909 | 10.7401 | 3.06E-24 | 1.23E-22 | 44.2162 |
| SIGLEC7 | 0.68106 | 1.472863 | 9.50261 | 1.01E-19 | 2.24E-18 | 33.9557 |
| LOXL1 | 0.68143 | 1.391924 | 7.24625 | 1.74E-12 | 1.72E-11 | 17.5786 |
| KCNE3 | 0.68145 | 1.823523 | 10.7923 | 1.94E-24 | 8.06E-23 | 44.666 |
| AHR | 0.68155 | 2.941518 | 7.61452 | 1.44E-13 | 1.60E-12 | 20.0218 |
| CIP2A | 0.68159 | 2.020937 | 12.2128 | 4.99E-30 | 4.53E-28 | 57.3887 |
| CLEC17A | 0.68169 | 0.97513 | 8.51795 | 2.15E-16 | 3.25E-15 | 26.4077 |
| EVC | 0.68192 | 1.687766 | 7.46749 | 3.93E-13 | 4.18E-12 | 19.0349 |
| TBX15 | 0.68192 | 2.216734 | 7.46185 | 4.09E-13 | 4.34E-12 | 18.9973 |
| IRX5 | 0.68194 | 0.731293 | 8.84581 | 1.79E-17 | 3.08E-16 | 28.8558 |
| TWIST1 | 0.68196 | 1.597133 | 7.8586 | 2.61E-14 | 3.16E-13 | 21.6933 |
| PRRX1 | 0.6823 | 5.320159 | 9.04591 | 3.79E-18 | 7.00E-17 | 30.3824 |
| C9orf64 | 0.68232 | 2.930818 | 6.94053 | 1.28E-11 | 1.15E-10 | 15.6241 |
| CYTH4 | 0.6825 | 3.025445 | 8.22106 | 1.93E-15 | 2.63E-14 | 24.2496 |
| IFIH1 | 0.68273 | 3.195328 | 8.82423 | 2.11E-17 | 3.59E-16 | 28.6926 |
| PTPN7 | 0.6828 | 1.026359 | 10.6275 | 8.15E-24 | 3.08E-22 | 43.2498 |
| STK32B | 0.683 | 2.232163 | 6.60174 | 1.09E-10 | 8.79E-10 | 13.5388 |
| MXD3 | 0.68335 | 2.218622 | 8.40875 | 4.86E-16 | 7.03E-15 | 25.6074 |
| VANGL1 | 0.68354 | 2.03005 | 11.2873 | 2.42E-26 | 1.30E-24 | 48.9985 |
| AGAP2-AS1 | 0.68354 | 2.083904 | 6.14766 | 1.67E-09 | 1.16E-08 | 10.881 |
| SIRPB2 | 0.68358 | 1.65268 | 9.79009 | 9.66E-21 | 2.46E-19 | 36.2663 |
| SALL1 | 0.68377 | 5.654758 | 8.76478 | 3.32E-17 | 5.54E-16 | 28.2446 |
| PTAR1 | 0.68381 | 3.91003 | 13.5081 | 2.00E-35 | 4.86E-33 | 69.6878 |
| BTN3A2 | 0.68387 | 4.008293 | 7.96448 | 1.23E-14 | 1.54E-13 | 22.4309 |
| RCSD1 | 0.68389 | 3.482098 | 10.5859 | 1.17E-23 | 4.33E-22 | 42.8944 |
| DENND2D | 0.68391 | 1.294844 | 10.6492 | 6.76E-24 | 2.59E-22 | 43.4356 |
| PI4K2B | 0.68416 | 2.574795 | 13.9936 | 1.64E-37 | 6.03E-35 | 74.4424 |
| AC010273.3 | 0.68419 | 1.24784 | 9.10889 | 2.31E-18 | 4.38E-17 | 30.8679 |
| TNFAIP8L2 | 0.68426 | 3.872753 | 7.84898 | 2.80E-14 | 3.37E-13 | 21.6266 |
| MOV10 | 0.68457 | 3.891075 | 11.1219 | 1.06E-25 | 5.26E-24 | 47.538 |
| WARS2-IT1 | 0.68462 | 1.400741 | 9.61866 | 3.93E-20 | 9.23E-19 | 34.8829 |
| AOAH | 0.68462 | 3.291339 | 8.61146 | 1.07E-16 | 1.67E-15 | 27.0991 |
| TGFBR2 | 0.68492 | 4.852704 | 9.89181 | 4.17E-21 | 1.11E-19 | 37.0948 |
| CD2 | 0.68492 | 0.947151 | 8.35816 | 7.06E-16 | 1.00E-14 | 25.2391 |
| PKN2 | 0.68505 | 4.319412 | 13.1005 | 1.06E-33 | 1.85E-31 | 65.7543 |
| FAM183A | 0.68516 | 0.730819 | 5.65392 | 2.71E-08 | 1.62E-07 | 8.17533 |
| TM4SF1 | 0.68597 | 3.691036 | 6.52891 | 1.70E-10 | 1.34E-09 | 13.1019 |
| CRB2 | 0.68619 | 3.50087 | 5.52267 | 5.50E-08 | 3.18E-07 | 7.48928 |
| TLR5 | 0.68619 | 2.056814 | 9.27857 | 6.06E-19 | 1.22E-17 | 32.1876 |
| IDH1 | 0.68692 | 5.712688 | 12.2135 | 4.96E-30 | 4.52E-28 | 57.3955 |
| SIGLEC1 | 0.68787 | 1.877044 | 6.57451 | 1.29E-10 | 1.03E-09 | 13.375 |
| PTCRA | 0.68791 | 0.836596 | 9.66417 | 2.71E-20 | 6.50E-19 | 35.2486 |
| CASP8 | 0.68814 | 1.707176 | 13.3092 | 1.40E-34 | 2.96E-32 | 67.7618 |
| XRN2 | 0.68826 | 5.944902 | 15.8733 | 7.64E-46 | 2.99E-42 | 93.442 |
| CRYZ | 0.68842 | 3.917461 | 9.63916 | 3.33E-20 | 7.87E-19 | 35.0475 |
| IRF1 | 0.68894 | 2.779637 | 8.98379 | 6.14E-18 | 1.12E-16 | 29.9059 |
| ETV6 | 0.68932 | 3.548154 | 14.5542 | 5.88E-40 | 4.19E-37 | 80.0165 |
| TMEM106C | 0.68953 | 4.850956 | 11.7333 | 4.24E-28 | 2.99E-26 | 52.9962 |
| FOXD3 | 0.68975 | 0.896292 | 7.60906 | 1.49E-13 | 1.66E-12 | 19.9849 |
| CENPL | 0.68979 | 2.03032 | 15.4421 | 6.70E-44 | 1.53E-40 | 89.0103 |
| HOXD13 | 0.69003 | 0.4016 | 7.31253 | 1.12E-12 | 1.13E-11 | 18.0112 |
| OASL | 0.69005 | 1.405668 | 7.24274 | 1.78E-12 | 1.76E-11 | 17.5557 |
| ZNF662 | 0.69009 | 2.294109 | 7.71841 | 6.99E-14 | 8.06E-13 | 20.7283 |
| PTPN6 | 0.69013 | 3.530484 | 8.46896 | 3.10E-16 | 4.60E-15 | 26.0477 |
| CYTIP | 0.69025 | 1.620066 | 10.5173 | 2.11E-23 | 7.51E-22 | 42.3099 |
| APOBEC3G | 0.69044 | 1.648485 | 10.4622 | 3.39E-23 | 1.19E-21 | 41.8428 |
| FBLIM1 | 0.69072 | 2.228987 | 8.38879 | 5.63E-16 | 8.09E-15 | 25.4619 |
| NETO2 | 0.69111 | 3.364656 | 7.09983 | 4.57E-12 | 4.32E-11 | 16.634 |
| H2AC6 | 0.6913 | 5.201933 | 8.14305 | 3.41E-15 | 4.52E-14 | 23.692 |
| STAT5A | 0.69142 | 3.276429 | 9.33033 | 4.01E-19 | 8.27E-18 | 32.5935 |
| LIPG | 0.69149 | 1.706854 | 8.5195 | 2.13E-16 | 3.22E-15 | 26.4191 |
| SERPINB1 | 0.69169 | 4.0282 | 9.45119 | 1.52E-19 | 3.32E-18 | 33.5474 |
| CASP3 | 0.69185 | 4.967352 | 13.0228 | 2.25E-33 | 3.56E-31 | 65.0107 |
| MACORIS | 0.69192 | 3.503467 | 7.95832 | 1.29E-14 | 1.60E-13 | 22.3878 |
| AC092718.4 | 0.69258 | 3.185686 | 9.81464 | 7.89E-21 | 2.04E-19 | 36.4657 |
| WDHD1 | 0.69293 | 1.956704 | 13.2354 | 2.87E-34 | 5.72E-32 | 67.0499 |
| AC133065.3 | 0.69315 | 1.853081 | 9.81069 | 8.15E-21 | 2.10E-19 | 36.4336 |
| CAPZA1 | 0.6932 | 5.501547 | 15.8737 | 7.61E-46 | 2.99E-42 | 93.4458 |
| AL390728.4 | 0.6933 | 3.550037 | 10.3832 | 6.67E-23 | 2.23E-21 | 41.1752 |
| HDAC1 | 0.69381 | 5.117638 | 11.384 | 1.02E-26 | 5.78E-25 | 49.8573 |
| CPXM1 | 0.69467 | 5.807333 | 5.21599 | 2.73E-07 | 1.45E-06 | 5.9417 |
| SHKBP1 | 0.69517 | 4.353262 | 12.0568 | 2.14E-29 | 1.80E-27 | 55.9493 |
| COLGALT1 | 0.69532 | 5.090985 | 13.6307 | 5.97E-36 | 1.63E-33 | 70.8817 |
| HOXD-AS2 | 0.6954 | 1.004341 | 7.65315 | 1.10E-13 | 1.24E-12 | 20.2836 |
| UBD | 0.69575 | 1.103031 | 8.31782 | 9.51E-16 | 1.33E-14 | 24.9467 |
| IGF2BP3 | 0.69583 | 0.474906 | 9.22429 | 9.31E-19 | 1.84E-17 | 31.7636 |
| CXorf21 | 0.69617 | 2.027192 | 8.48435 | 2.77E-16 | 4.12E-15 | 26.1606 |
| BCL2L12 | 0.69619 | 2.355691 | 10.4146 | 5.10E-23 | 1.73E-21 | 41.4396 |
| TTF2 | 0.69653 | 1.910391 | 14.9706 | 8.52E-42 | 1.17E-38 | 84.2107 |
| NFATC2 | 0.69655 | 2.362081 | 9.78533 | 1.00E-20 | 2.55E-19 | 36.2276 |
| DDX60L | 0.69667 | 2.005822 | 11.4996 | 3.57E-27 | 2.18E-25 | 50.8902 |
| SOCS2 | 0.6969 | 2.488652 | 7.05014 | 6.32E-12 | 5.90E-11 | 16.317 |
| IFNGR2 | 0.69702 | 5.586358 | 14.8758 | 2.24E-41 | 2.48E-38 | 83.2517 |
| PIRT | 0.69708 | 2.52231 | 4.97847 | 8.98E-07 | 4.44E-06 | 4.79733 |
| APOBEC3B | 0.69725 | 1.292577 | 10.8617 | 1.06E-24 | 4.58E-23 | 45.2667 |
| TMEM140 | 0.69742 | 4.424773 | 12.0504 | 2.27E-29 | 1.89E-27 | 55.8902 |
| SLC4A7 | 0.69768 | 2.751705 | 12.1527 | 8.75E-30 | 7.78E-28 | 56.8332 |
| APOC2 | 0.69797 | 1.629746 | 9.33542 | 3.85E-19 | 7.97E-18 | 32.6335 |
| ZFP36 | 0.69798 | 6.537177 | 5.61113 | 3.42E-08 | 2.03E-07 | 7.95012 |
| ARHGAP25 | 0.69813 | 2.7453 | 10.7848 | 2.07E-24 | 8.58E-23 | 44.6013 |
| STK40 | 0.69863 | 4.700651 | 11.5714 | 1.86E-27 | 1.18E-25 | 51.5353 |
| TMEM51 | 0.69864 | 3.96951 | 11.0512 | 1.99E-25 | 9.47E-24 | 46.918 |
| SKP2 | 0.69934 | 3.717075 | 14.6597 | 2.02E-40 | 1.76E-37 | 81.0752 |
| HGF | 0.69941 | 1.227988 | 8.86834 | 1.50E-17 | 2.61E-16 | 29.0265 |
| ZC3HAV1 | 0.69948 | 4.272567 | 13.0714 | 1.41E-33 | 2.37E-31 | 65.4755 |
| ADAP2 | 0.69962 | 3.637933 | 9.65047 | 3.03E-20 | 7.21E-19 | 35.1383 |
| MYCBP | 0.69972 | 2.861202 | 13.3227 | 1.22E-34 | 2.62E-32 | 67.892 |
| VAV3 | 0.69977 | 1.197098 | 6.67069 | 7.09E-11 | 5.85E-10 | 13.9563 |
| IGFBP3 | 0.6998 | 4.637248 | 4.50063 | 8.53E-06 | 3.67E-05 | 2.64162 |
| TSPO | 0.69986 | 5.969866 | 7.6157 | 1.43E-13 | 1.59E-12 | 20.0298 |
| NFE2L3 | 0.70026 | 2.191293 | 11.8125 | 2.05E-28 | 1.51E-26 | 53.7155 |
| SEL1L3 | 0.70027 | 2.4282 | 5.64122 | 2.90E-08 | 1.73E-07 | 8.10832 |
| CDC25A | 0.70034 | 2.178473 | 10.0102 | 1.56E-21 | 4.39E-20 | 38.0664 |
| SYDE1 | 0.70036 | 3.905125 | 11.0868 | 1.45E-25 | 7.08E-24 | 47.2298 |
| RUNX3 | 0.7005 | 1.505358 | 10.4933 | 2.60E-23 | 9.15E-22 | 42.1061 |
| ZNF436 | 0.70062 | 4.569071 | 11.4233 | 7.12E-27 | 4.20E-25 | 50.2085 |
| KDELR2 | 0.70066 | 6.45647 | 15.5028 | 3.58E-44 | 8.91E-41 | 89.632 |
| KIRREL1 | 0.7013 | 3.193358 | 10.1959 | 3.27E-22 | 1.01E-20 | 39.6051 |
| VASP | 0.70163 | 4.592397 | 13.1653 | 5.67E-34 | 1.06E-31 | 66.3758 |
| AC064875.1 | 0.70169 | 0.839569 | 7.22731 | 1.97E-12 | 1.94E-11 | 17.4555 |
| LMNB2 | 0.70171 | 5.082778 | 12.4427 | 5.74E-31 | 6.17E-29 | 59.5276 |
| HELZ2 | 0.70172 | 2.831392 | 9.26632 | 6.68E-19 | 1.34E-17 | 32.0918 |
| AIF1 | 0.70183 | 6.08579 | 7.14585 | 3.38E-12 | 3.24E-11 | 16.9292 |
| TMEM176B | 0.70213 | 5.500535 | 6.2388 | 9.75E-10 | 7.02E-09 | 11.4017 |
| F13A1 | 0.70215 | 2.34988 | 5.2644 | 2.13E-07 | 1.14E-06 | 6.18075 |
| HOXA4 | 0.70225 | 0.453057 | 7.32148 | 1.05E-12 | 1.07E-11 | 18.0699 |
| PCDH18 | 0.70339 | 2.890944 | 10.2908 | 1.47E-22 | 4.68E-21 | 40.3985 |
| VCL | 0.70369 | 4.279817 | 9.91328 | 3.49E-21 | 9.43E-20 | 37.2704 |
| IBSP | 0.70373 | 0.610226 | 6.19186 | 1.29E-09 | 9.10E-09 | 11.1327 |
| ZNF28 | 0.70381 | 2.723099 | 14.1359 | 3.96E-38 | 1.76E-35 | 75.8491 |
| DBF4 | 0.70385 | 2.72411 | 12.6079 | 1.20E-31 | 1.43E-29 | 61.0767 |
| STK38 | 0.704 | 4.644124 | 15.471 | 4.97E-44 | 1.18E-40 | 89.3064 |
| DOCK7 | 0.70431 | 4.294684 | 8.9456 | 8.27E-18 | 1.48E-16 | 29.6141 |
| SP140L | 0.70461 | 1.759089 | 11.6095 | 1.31E-27 | 8.62E-26 | 51.8779 |
| CASP2 | 0.70473 | 3.590564 | 14.1188 | 4.70E-38 | 2.04E-35 | 75.6792 |
| ZBTB42 | 0.70516 | 1.973222 | 9.65685 | 2.88E-20 | 6.86E-19 | 35.1897 |
| CCR5AS | 0.7053 | 1.678321 | 8.77887 | 2.98E-17 | 5.00E-16 | 28.3505 |
| CENPI | 0.70552 | 1.241948 | 12.6313 | 9.60E-32 | 1.16E-29 | 61.2968 |
| NAGA | 0.70557 | 4.731217 | 12.8312 | 1.42E-32 | 2.01E-30 | 63.1861 |
| RACGAP1 | 0.70623 | 4.050205 | 9.91892 | 3.33E-21 | 9.01E-20 | 37.3165 |
| CSF2RA | 0.70629 | 3.746366 | 7.60469 | 1.54E-13 | 1.71E-12 | 19.9554 |
| CHIC2 | 0.70631 | 4.523164 | 12.3113 | 1.98E-30 | 1.90E-28 | 58.3021 |
| SELENON | 0.7064 | 7.069672 | 12.5282 | 2.56E-31 | 2.90E-29 | 60.3274 |
| SQOR | 0.70668 | 2.672414 | 10.0989 | 7.41E-22 | 2.17E-20 | 38.7985 |
| SERPINA5 | 0.70753 | 0.868752 | 6.79487 | 3.25E-11 | 2.78E-10 | 14.717 |
| HS2ST1 | 0.70764 | 4.519309 | 12.3387 | 1.53E-30 | 1.50E-28 | 58.5571 |
| RHBDF2 | 0.70772 | 3.76791 | 7.9414 | 1.45E-14 | 1.80E-13 | 22.2695 |
| HLA-A | 0.70822 | 8.894674 | 9.32375 | 4.23E-19 | 8.68E-18 | 32.5418 |
| CROT | 0.70837 | 3.954054 | 11.736 | 4.13E-28 | 2.92E-26 | 53.0212 |
| RUBCNL | 0.70845 | 2.034894 | 8.53112 | 1.95E-16 | 2.96E-15 | 26.5048 |
| SMC2 | 0.70867 | 4.028361 | 11.9708 | 4.75E-29 | 3.83E-27 | 55.1597 |
| ASAP3 | 0.70887 | 5.119209 | 9.32669 | 4.13E-19 | 8.49E-18 | 32.5649 |
| SOX11 | 0.70957 | 4.173683 | 6.45313 | 2.70E-10 | 2.08E-09 | 12.6515 |
| DOCK11 | 0.70981 | 3.161925 | 11.8929 | 9.77E-29 | 7.56E-27 | 54.4474 |
| NLRC5 | 0.7103 | 2.486734 | 10.1398 | 5.26E-22 | 1.57E-20 | 39.138 |
| H2BC11 | 0.7104 | 1.262854 | 11.3195 | 1.81E-26 | 1.00E-24 | 49.2839 |
| AASS | 0.71076 | 4.259575 | 9.42913 | 1.82E-19 | 3.92E-18 | 33.3727 |
| BVES | 0.7118 | 2.451187 | 8.53553 | 1.89E-16 | 2.87E-15 | 26.5372 |
| PALD1 | 0.7123 | 5.617102 | 12.066 | 1.96E-29 | 1.65E-27 | 56.0341 |
| TNFSF13B | 0.71233 | 3.11249 | 5.2807 | 1.96E-07 | 1.06E-06 | 6.2617 |
| TMEM71 | 0.71236 | 1.015045 | 9.74389 | 1.41E-20 | 3.51E-19 | 35.8919 |
| IFNLR1 | 0.71265 | 1.607526 | 9.60897 | 4.25E-20 | 9.96E-19 | 34.8052 |
| RSPH4A | 0.71308 | 1.514227 | 8.24037 | 1.68E-15 | 2.30E-14 | 24.3882 |
| NFIL3 | 0.71318 | 4.959314 | 11.5925 | 1.53E-27 | 9.94E-26 | 51.725 |
| H4C9 | 0.71326 | 2.547291 | 10.705 | 4.16E-24 | 1.64E-22 | 43.9142 |
| FKBP10 | 0.71365 | 5.804477 | 7.72527 | 6.66E-14 | 7.70E-13 | 20.7751 |
| WDR78 | 0.71374 | 1.564262 | 9.63938 | 3.32E-20 | 7.86E-19 | 35.0492 |
| RGS19 | 0.71386 | 4.559355 | 12.1484 | 9.12E-30 | 8.08E-28 | 56.7931 |
| FRMD3 | 0.7141 | 3.373917 | 9.07962 | 2.91E-18 | 5.43E-17 | 30.6419 |
| NDC1 | 0.7143 | 3.672418 | 15.1882 | 9.16E-43 | 1.62E-39 | 86.419 |
| GLIPR1 | 0.71435 | 2.999234 | 9.47362 | 1.27E-19 | 2.80E-18 | 33.7253 |
| FANCC | 0.7145 | 2.205319 | 13.6866 | 3.44E-36 | 1.01E-33 | 71.4277 |
| A2M | 0.71478 | 8.117309 | 10.1932 | 3.35E-22 | 1.03E-20 | 39.5819 |
| HCP5 | 0.71485 | 2.769105 | 8.16062 | 3.00E-15 | 4.00E-14 | 23.8173 |
| SIX1 | 0.71514 | 2.228279 | 6.55647 | 1.44E-10 | 1.15E-09 | 13.2667 |
| EML4 | 0.71527 | 3.651178 | 16.5395 | 7.09E-49 | 6.47E-45 | 100.359 |
| AC015922.2 | 0.71561 | 3.114716 | 7.27324 | 1.45E-12 | 1.45E-11 | 17.7544 |
| DEF6 | 0.71594 | 3.519606 | 8.51118 | 2.26E-16 | 3.41E-15 | 26.3579 |
| YBX3 | 0.71666 | 3.900431 | 9.9937 | 1.79E-21 | 5.01E-20 | 37.9303 |
| CCDC18 | 0.71666 | 1.288294 | 14.4855 | 1.18E-39 | 7.42E-37 | 79.3291 |
| PIK3R5 | 0.71686 | 2.349888 | 9.65175 | 3.00E-20 | 7.14E-19 | 35.1486 |
| VAV1 | 0.71704 | 2.883669 | 8.90665 | 1.12E-17 | 1.98E-16 | 29.3174 |
| HTRA3 | 0.71732 | 1.179284 | 8.62441 | 9.66E-17 | 1.52E-15 | 27.1953 |
| GPR82 | 0.71737 | 0.811445 | 9.65888 | 2.83E-20 | 6.76E-19 | 35.206 |
| FPGT | 0.7175 | 2.89772 | 13.4522 | 3.45E-35 | 8.05E-33 | 69.1457 |
| LGALS3BP | 0.71755 | 7.4831 | 10.1997 | 3.17E-22 | 9.76E-21 | 39.6362 |
| BTG3 | 0.71771 | 5.084021 | 13.073 | 1.39E-33 | 2.34E-31 | 65.491 |
| SOAT1 | 0.71819 | 4.134765 | 15.5118 | 3.26E-44 | 8.91E-41 | 89.7236 |
| LIMA1 | 0.71871 | 5.786333 | 8.88596 | 1.31E-17 | 2.30E-16 | 29.1602 |
| GBP1P1 | 0.71882 | 1.329102 | 8.18882 | 2.45E-15 | 3.28E-14 | 24.0187 |
| NTN1 | 0.71974 | 5.685424 | 8.69077 | 5.84E-17 | 9.45E-16 | 27.6898 |
| GLMP | 0.72004 | 3.826225 | 11.1913 | 5.72E-26 | 2.92E-24 | 48.1494 |
| SMC5 | 0.72045 | 4.018117 | 14.1852 | 2.42E-38 | 1.14E-35 | 76.3371 |
| UHRF1 | 0.72063 | 4.097236 | 7.7232 | 6.76E-14 | 7.80E-13 | 20.761 |
| GGH | 0.72113 | 3.813639 | 11.4794 | 4.28E-27 | 2.58E-25 | 50.7098 |
| CD300LF | 0.72117 | 1.60937 | 9.95106 | 2.55E-21 | 6.99E-20 | 37.58 |
| EDNRA | 0.72194 | 3.770944 | 7.30383 | 1.19E-12 | 1.20E-11 | 17.9542 |
| HAND2 | 0.72235 | 0.571387 | 8.96577 | 7.07E-18 | 1.28E-16 | 29.7681 |
| CLEC5A | 0.72255 | 0.9295 | 7.46283 | 4.06E-13 | 4.31E-12 | 19.0039 |
| SLC4A2 | 0.72256 | 4.936274 | 12.3664 | 1.18E-30 | 1.19E-28 | 58.8152 |
| MYO9B | 0.72281 | 5.342057 | 14.059 | 8.53E-38 | 3.44E-35 | 75.0883 |
| SYTL3 | 0.72282 | 2.302507 | 10.847 | 1.20E-24 | 5.16E-23 | 45.1389 |
| S100A6 | 0.72283 | 8.797642 | 7.28621 | 1.33E-12 | 1.34E-11 | 17.839 |
| DDR2 | 0.72339 | 3.757018 | 6.36829 | 4.51E-10 | 3.38E-09 | 12.1525 |
| PRAM1 | 0.72346 | 2.587698 | 8.44729 | 3.65E-16 | 5.37E-15 | 25.8889 |
| SH3PXD2B | 0.72398 | 4.818952 | 12.3134 | 1.94E-30 | 1.88E-28 | 58.3217 |
| POLD1 | 0.72416 | 3.427404 | 12.5188 | 2.80E-31 | 3.14E-29 | 60.2392 |
| FUCA2 | 0.72476 | 4.584257 | 8.56254 | 1.54E-16 | 2.37E-15 | 26.7367 |
| CCDC146 | 0.72489 | 2.860457 | 8.784 | 2.87E-17 | 4.81E-16 | 28.3891 |
| NEXN | 0.72489 | 2.00468 | 10.3219 | 1.13E-22 | 3.64E-21 | 40.659 |
| FCGR2C | 0.72513 | 0.748974 | 9.63145 | 3.54E-20 | 8.35E-19 | 34.9856 |
| PTBP1 | 0.72568 | 5.928092 | 14.6215 | 2.98E-40 | 2.40E-37 | 80.6917 |
| CD101 | 0.72576 | 1.317013 | 10.1578 | 4.52E-22 | 1.36E-20 | 39.2874 |
| BMP8B | 0.72589 | 2.56628 | 7.01701 | 7.84E-12 | 7.23E-11 | 16.1067 |
| SLC44A5 | 0.7266 | 2.868785 | 7.31639 | 1.09E-12 | 1.10E-11 | 18.0365 |
| PABPC1L | 0.7269 | 2.85166 | 7.51288 | 2.89E-13 | 3.12E-12 | 19.338 |
| TP53INP1 | 0.72699 | 4.266985 | 12.4593 | 4.91E-31 | 5.32E-29 | 59.6828 |
| PCED1B-AS1 | 0.72714 | 2.595617 | 8.86635 | 1.52E-17 | 2.65E-16 | 29.0113 |
| LCP2 | 0.72793 | 2.996934 | 10.0045 | 1.63E-21 | 4.59E-20 | 38.0195 |
| CNGA3 | 0.72796 | 2.667483 | 4.80807 | 2.05E-06 | 9.61E-06 | 4.00597 |
| RNA5SP118 | 0.7288 | 3.137364 | 6.24602 | 9.35E-10 | 6.74E-09 | 11.4432 |
| IGF2BP2 | 0.7291 | 1.000981 | 6.9107 | 1.56E-11 | 1.38E-10 | 15.4371 |
| DSN1 | 0.7291 | 3.631657 | 12.9701 | 3.74E-33 | 5.68E-31 | 64.5073 |
| FAM181A | 0.72923 | 2.804585 | 5.06661 | 5.81E-07 | 2.95E-06 | 5.2164 |
| CTTNBP2NL | 0.73015 | 3.886659 | 14.0847 | 6.61E-38 | 2.68E-35 | 75.3417 |
| CDCA4 | 0.7303 | 2.764281 | 12.491 | 3.64E-31 | 4.02E-29 | 59.979 |
| FABP5 | 0.73039 | 2.748321 | 5.59906 | 3.65E-08 | 2.15E-07 | 7.88688 |
| MICB | 0.7307 | 1.593246 | 11.3941 | 9.27E-27 | 5.34E-25 | 49.9472 |
| CELSR1 | 0.73075 | 0.966652 | 9.38892 | 2.51E-19 | 5.32E-18 | 33.0549 |
| TPST1 | 0.73087 | 5.752938 | 11.8763 | 1.14E-28 | 8.73E-27 | 54.2965 |
| LINC01150 | 0.73091 | 1.968412 | 9.52649 | 8.30E-20 | 1.87E-18 | 34.1459 |
| LAP3 | 0.73112 | 6.258886 | 12.3205 | 1.82E-30 | 1.76E-28 | 58.3879 |
| SLC26A2 | 0.73123 | 2.616757 | 11.8124 | 2.05E-28 | 1.51E-26 | 53.7145 |
| SKAP2 | 0.73161 | 3.553385 | 7.53918 | 2.41E-13 | 2.63E-12 | 19.5142 |
| MCAM | 0.73174 | 4.555218 | 9.11652 | 2.18E-18 | 4.13E-17 | 30.9269 |
| CHAF1A | 0.73179 | 3.59669 | 11.1929 | 5.64E-26 | 2.88E-24 | 48.1635 |
| ICAM1 | 0.7319 | 3.081381 | 6.97574 | 1.02E-11 | 9.30E-11 | 15.8457 |
| BMF | 0.73213 | 2.773019 | 9.20128 | 1.12E-18 | 2.19E-17 | 31.5844 |
| PALLD | 0.73306 | 4.842532 | 12.9383 | 5.08E-33 | 7.59E-31 | 64.2047 |
| CNTRL | 0.7332 | 2.870196 | 14.9547 | 1.00E-41 | 1.33E-38 | 84.05 |
| HCK | 0.73332 | 3.978742 | 8.42037 | 4.45E-16 | 6.48E-15 | 25.6922 |
| C7orf31 | 0.73382 | 1.981097 | 11.5708 | 1.87E-27 | 1.19E-25 | 51.5293 |
| MTMR11 | 0.73445 | 3.07335 | 10.2938 | 1.43E-22 | 4.58E-21 | 40.4229 |
| TLR3 | 0.73587 | 2.155515 | 11.0543 | 1.94E-25 | 9.23E-24 | 46.9446 |
| TRIM5 | 0.73644 | 2.999682 | 9.90039 | 3.88E-21 | 1.04E-19 | 37.165 |
| SH3GLB1 | 0.73663 | 5.712561 | 15.2098 | 7.33E-43 | 1.38E-39 | 86.6395 |
| SLC47A2 | 0.73674 | 1.243954 | 6.79282 | 3.29E-11 | 2.82E-10 | 14.7044 |
| TPM4 | 0.7369 | 5.830605 | 13.9772 | 1.93E-37 | 6.87E-35 | 74.2801 |
| PIFO | 0.73725 | 3.033908 | 6.79284 | 3.29E-11 | 2.82E-10 | 14.7045 |
| SPAG5 | 0.73835 | 2.899135 | 9.25535 | 7.28E-19 | 1.46E-17 | 32.006 |
| RBL1 | 0.73864 | 2.776908 | 14.5089 | 9.30E-40 | 6.14E-37 | 79.5631 |
| RELL1 | 0.73871 | 3.029899 | 10.636 | 7.58E-24 | 2.87E-22 | 43.3226 |
| LINC01736 | 0.73872 | 4.483873 | 5.37698 | 1.19E-07 | 6.58E-07 | 6.74435 |
| NMI | 0.73894 | 2.833636 | 10.3767 | 7.05E-23 | 2.35E-21 | 41.12 |
| NID2 | 0.73903 | 1.861898 | 9.19401 | 1.18E-18 | 2.32E-17 | 31.5278 |
| RFWD3 | 0.73907 | 3.465605 | 15.03 | 4.64E-42 | 6.87E-39 | 84.813 |
| DCLRE1B | 0.7399 | 3.479883 | 14.9486 | 1.07E-41 | 1.36E-38 | 83.9878 |
| BTN2A2 | 0.74 | 2.599529 | 12.213 | 4.98E-30 | 4.53E-28 | 57.3908 |
| TMSB15A | 0.74003 | 3.682333 | 5.1749 | 3.37E-07 | 1.76E-06 | 5.74028 |
| FAM20C | 0.7406 | 5.258398 | 10.3769 | 7.04E-23 | 2.35E-21 | 41.1216 |
| BST2 | 0.74104 | 6.491625 | 7.43917 | 4.77E-13 | 5.03E-12 | 18.8466 |
| CSTA | 0.74127 | 1.192458 | 8.41036 | 4.80E-16 | 6.95E-15 | 25.6191 |
| TSPAN12 | 0.74172 | 4.794125 | 8.06159 | 6.14E-15 | 7.94E-14 | 23.1141 |
| DOCK8 | 0.74197 | 2.577863 | 9.09475 | 2.58E-18 | 4.87E-17 | 30.7587 |
| LRR1 | 0.74262 | 2.641016 | 15.6352 | 9.08E-45 | 2.93E-41 | 90.9894 |
| SCIMP | 0.74325 | 1.696929 | 10.8997 | 7.58E-25 | 3.36E-23 | 45.5964 |
| CALD1 | 0.74388 | 5.136988 | 11.3927 | 9.38E-27 | 5.39E-25 | 49.9354 |
| MCM5 | 0.74394 | 4.03472 | 13.631 | 5.96E-36 | 1.63E-33 | 70.8842 |
| CD4 | 0.7441 | 4.99528 | 9.29886 | 5.15E-19 | 1.05E-17 | 32.3465 |
| CSF3R | 0.74411 | 2.906061 | 8.99771 | 5.51E-18 | 1.01E-16 | 30.0125 |
| SERPINA3 | 0.74436 | 1.13002 | 7.96298 | 1.25E-14 | 1.56E-13 | 22.4205 |
| CEP135 | 0.74482 | 1.726993 | 15.158 | 1.25E-42 | 2.01E-39 | 86.1127 |
| ITGAX | 0.74583 | 3.523546 | 7.92688 | 1.61E-14 | 1.99E-13 | 22.1682 |
| LPCAT2 | 0.74633 | 3.115667 | 8.29115 | 1.16E-15 | 1.61E-14 | 24.754 |
| PGM2 | 0.74638 | 4.052959 | 14.1939 | 2.21E-38 | 1.06E-35 | 76.4236 |
| CD302 | 0.74651 | 2.270468 | 9.86555 | 5.18E-21 | 1.37E-19 | 36.8804 |
| ARHGAP30 | 0.74686 | 3.399702 | 10.3334 | 1.02E-22 | 3.32E-21 | 40.7561 |
| ITPRIPL2 | 0.74727 | 3.060187 | 12.4327 | 6.32E-31 | 6.75E-29 | 59.4337 |
| WDR76 | 0.74762 | 2.811579 | 10.8688 | 9.94E-25 | 4.34E-23 | 45.3279 |
| CEBPA | 0.74773 | 3.960432 | 8.08971 | 5.02E-15 | 6.54E-14 | 23.3132 |
| ZWILCH | 0.74773 | 2.541989 | 13.1904 | 4.44E-34 | 8.63E-32 | 66.6167 |
| STAB1 | 0.74885 | 4.292045 | 7.78037 | 4.53E-14 | 5.33E-13 | 21.1531 |
| TMSB4X | 0.74899 | 10.30134 | 11.8608 | 1.31E-28 | 9.96E-27 | 54.155 |
| JPT2 | 0.74901 | 4.360031 | 12.7208 | 4.09E-32 | 5.36E-30 | 62.1405 |
| FBLN5 | 0.74903 | 3.248421 | 7.08934 | 4.90E-12 | 4.62E-11 | 16.567 |
| RHPN2 | 0.74963 | 3.97939 | 8.44825 | 3.62E-16 | 5.34E-15 | 25.896 |
| TEAD2 | 0.74991 | 3.015714 | 9.43554 | 1.73E-19 | 3.73E-18 | 33.4234 |
| C4A | 0.74997 | 3.150642 | 6.64745 | 8.19E-11 | 6.71E-10 | 13.8152 |
| CD180 | 0.75033 | 1.590402 | 10.4423 | 4.02E-23 | 1.39E-21 | 41.6738 |
| SAA1 | 0.75041 | 0.604498 | 6.4097 | 3.51E-10 | 2.67E-09 | 12.3954 |
| FBXO32 | 0.75052 | 4.974626 | 7.32273 | 1.05E-12 | 1.06E-11 | 18.0781 |
| DOCK2 | 0.75078 | 2.451971 | 9.48448 | 1.17E-19 | 2.58E-18 | 33.8116 |
| TNFRSF1B | 0.75092 | 4.794772 | 9.40613 | 2.19E-19 | 4.67E-18 | 33.1908 |
| CLEC7A | 0.75114 | 2.283224 | 8.46365 | 3.23E-16 | 4.77E-15 | 26.0088 |
| SLC15A3 | 0.75185 | 3.35851 | 9.52759 | 8.23E-20 | 1.86E-18 | 34.1547 |
| PCOLCE | 0.7522 | 2.937605 | 8.3641 | 6.76E-16 | 9.62E-15 | 25.2823 |
| PPP1R3B | 0.75239 | 2.617406 | 11.651 | 9.00E-28 | 6.07E-26 | 52.2521 |
| SEPTIN10 | 0.75273 | 4.469935 | 13.0972 | 1.10E-33 | 1.91E-31 | 65.7223 |
| CALHM6 | 0.75278 | 2.250708 | 7.85136 | 2.75E-14 | 3.31E-13 | 21.6431 |
| FCGR1A | 0.75348 | 3.407526 | 7.43594 | 4.87E-13 | 5.13E-12 | 18.8251 |
| COL5A1 | 0.75391 | 1.499718 | 7.29061 | 1.30E-12 | 1.30E-11 | 17.8678 |
| AJ011932.1 | 0.75403 | 0.921569 | 8.32836 | 8.80E-16 | 1.24E-14 | 25.023 |
| GADD45A | 0.75415 | 5.414513 | 7.44965 | 4.44E-13 | 4.70E-12 | 18.9162 |
| EPHB2 | 0.75431 | 2.766203 | 10.1789 | 3.78E-22 | 1.15E-20 | 39.4633 |
| ARPC1B | 0.75472 | 5.08166 | 9.85726 | 5.55E-21 | 1.46E-19 | 36.8127 |
| MAD2L1 | 0.75504 | 2.724292 | 11.462 | 5.02E-27 | 2.99E-25 | 50.5541 |
| GDF15 | 0.75548 | 1.280611 | 6.93083 | 1.37E-11 | 1.22E-10 | 15.5632 |
| CASP6 | 0.75549 | 2.930207 | 12.9426 | 4.88E-33 | 7.32E-31 | 64.2457 |
| ATAD2 | 0.75608 | 3.56733 | 12.0282 | 2.79E-29 | 2.30E-27 | 55.6869 |
| GAPT | 0.75616 | 1.209824 | 9.95653 | 2.44E-21 | 6.70E-20 | 37.6249 |
| MATN2 | 0.75692 | 5.374021 | 7.71429 | 7.19E-14 | 8.28E-13 | 20.7001 |
| HERC5 | 0.75723 | 2.871033 | 8.12459 | 3.90E-15 | 5.13E-14 | 23.5607 |
| CBX2 | 0.75737 | 2.441722 | 9.62261 | 3.81E-20 | 8.94E-19 | 34.9146 |
| AR | 0.7576 | 2.319746 | 7.45041 | 4.42E-13 | 4.67E-12 | 18.9212 |
| MFNG | 0.75794 | 3.995888 | 10.2699 | 1.75E-22 | 5.52E-21 | 40.2232 |
| TANC1 | 0.75806 | 3.89715 | 13.1854 | 4.66E-34 | 8.96E-32 | 66.5695 |
| TBXAS1 | 0.7581 | 3.49184 | 9.23805 | 8.35E-19 | 1.66E-17 | 31.8709 |
| MAN2B1 | 0.75894 | 4.778668 | 13.4129 | 5.07E-35 | 1.14E-32 | 68.7647 |
| CDT1 | 0.75926 | 2.906918 | 8.50284 | 2.41E-16 | 3.61E-15 | 26.2965 |
| THEMIS2 | 0.76126 | 3.635303 | 8.79624 | 2.61E-17 | 4.40E-16 | 28.4813 |
| PDLIM1 | 0.7615 | 2.975107 | 6.8245 | 2.69E-11 | 2.33E-10 | 14.9003 |
| TRIM14 | 0.76175 | 4.039255 | 9.55728 | 6.47E-20 | 1.48E-18 | 34.3916 |
| SERINC2 | 0.76341 | 1.985175 | 8.14471 | 3.37E-15 | 4.47E-14 | 23.7039 |
| C7orf57 | 0.76342 | 0.917365 | 6.54869 | 1.51E-10 | 1.20E-09 | 13.2201 |
| FBXO5 | 0.7636 | 2.900005 | 11.4332 | 6.51E-27 | 3.85E-25 | 50.2964 |
| EPSTI1 | 0.76369 | 1.991575 | 9.17186 | 1.41E-18 | 2.73E-17 | 31.3557 |
| AC018755.4 | 0.76424 | 2.252111 | 8.13064 | 3.73E-15 | 4.92E-14 | 23.6037 |
| SERPINB8 | 0.76434 | 2.526972 | 12.982 | 3.34E-33 | 5.11E-31 | 64.6212 |
| FAM126A | 0.7644 | 2.571375 | 12.1587 | 8.28E-30 | 7.38E-28 | 56.8885 |
| AL357992.1 | 0.76462 | 1.247874 | 8.76058 | 3.43E-17 | 5.71E-16 | 28.213 |
| CCN1 | 0.76509 | 5.234555 | 5.04068 | 6.60E-07 | 3.33E-06 | 5.09243 |
| CCL5 | 0.76513 | 2.173303 | 7.13774 | 3.57E-12 | 3.41E-11 | 16.8771 |
| PARP14 | 0.76553 | 3.865775 | 10.0859 | 8.27E-22 | 2.40E-20 | 38.6908 |
| MAP1LC3C | 0.7657 | 0.843333 | 7.73839 | 6.08E-14 | 7.06E-13 | 20.865 |
| HASPIN | 0.7669 | 0.862939 | 13.0091 | 2.57E-33 | 3.99E-31 | 64.8795 |
| P3H2 | 0.76708 | 2.181249 | 8.66261 | 7.23E-17 | 1.16E-15 | 27.4796 |
| ADAM12 | 0.76721 | 1.057144 | 8.79753 | 2.59E-17 | 4.37E-16 | 28.4911 |
| HPSE | 0.76771 | 2.431372 | 9.37784 | 2.74E-19 | 5.79E-18 | 32.9675 |
| UNC93B1 | 0.76832 | 3.817675 | 9.18901 | 1.23E-18 | 2.40E-17 | 31.4889 |
| SLC30A7 | 0.76855 | 2.756453 | 16.7549 | 7.29E-50 | 1.33E-45 | 102.612 |
| HLA-H | 0.76904 | 5.14868 | 8.76888 | 3.22E-17 | 5.37E-16 | 28.2753 |
| AC092131.1 | 0.76916 | 1.583833 | 7.01284 | 8.06E-12 | 7.42E-11 | 16.0802 |
| ANXA5 | 0.7697 | 7.942014 | 10.4565 | 3.56E-23 | 1.24E-21 | 41.794 |
| VAMP8 | 0.77004 | 5.321035 | 9.25324 | 7.41E-19 | 1.48E-17 | 31.9895 |
| SLC1A5 | 0.77032 | 3.728038 | 8.563 | 1.53E-16 | 2.36E-15 | 26.7401 |
| TTC26 | 0.77035 | 2.291466 | 12.9077 | 6.82E-33 | 1.00E-30 | 63.9132 |
| E2F1 | 0.77047 | 3.850835 | 8.54354 | 1.78E-16 | 2.71E-15 | 26.5964 |
| CKAP2 | 0.7706 | 4.267233 | 10.9023 | 7.41E-25 | 3.29E-23 | 45.6186 |
| IGFBP5 | 0.7711 | 7.017678 | 6.84184 | 2.41E-11 | 2.10E-10 | 15.0078 |
| GPSM3 | 0.77111 | 4.812094 | 10.0072 | 1.60E-21 | 4.50E-20 | 38.0418 |
| NEDD1 | 0.77122 | 3.599146 | 14.2482 | 1.28E-38 | 6.56E-36 | 76.9632 |
| CDK4 | 0.77218 | 6.24616 | 9.36516 | 3.04E-19 | 6.38E-18 | 32.8675 |
| TP53I3 | 0.77256 | 4.097424 | 10.1369 | 5.39E-22 | 1.60E-20 | 39.1139 |
| HOXD10 | 0.77297 | 0.54187 | 7.88007 | 2.25E-14 | 2.73E-13 | 21.8422 |
| HOXB7 | 0.77334 | 1.113957 | 8.50097 | 2.44E-16 | 3.66E-15 | 26.2828 |
| CDCA3 | 0.77339 | 2.127872 | 11.5921 | 1.54E-27 | 9.96E-26 | 51.7216 |
| SECTM1 | 0.77357 | 1.840872 | 8.07015 | 5.78E-15 | 7.48E-14 | 23.1746 |
| APOBR | 0.77376 | 2.566141 | 10.1984 | 3.21E-22 | 9.86E-21 | 39.6253 |
| B2M | 0.77544 | 9.68731 | 11.0369 | 2.26E-25 | 1.07E-23 | 46.7924 |
| CX3CR1 | 0.77558 | 5.467129 | 5.41055 | 9.97E-08 | 5.58E-07 | 6.91443 |
| AL035446.1 | 0.77574 | 1.663006 | 6.4186 | 3.33E-10 | 2.53E-09 | 12.4478 |
| PLCE1 | 0.77629 | 2.878906 | 12.3775 | 1.06E-30 | 1.08E-28 | 58.9184 |
| REEP4 | 0.77658 | 3.975426 | 13.2387 | 2.78E-34 | 5.55E-32 | 67.0818 |
| MNS1 | 0.77668 | 3.089478 | 12.9056 | 6.97E-33 | 1.02E-30 | 63.893 |
| TNFRSF1A | 0.77691 | 5.471564 | 9.37983 | 2.70E-19 | 5.71E-18 | 32.9832 |
| TRAM2 | 0.77742 | 3.112836 | 11.9418 | 6.22E-29 | 4.90E-27 | 54.8943 |
| HMGB2 | 0.7778 | 5.978076 | 11.8665 | 1.25E-28 | 9.47E-27 | 54.2074 |
| LPAR5 | 0.77794 | 3.849198 | 7.91692 | 1.73E-14 | 2.13E-13 | 22.0987 |
| ITGB1 | 0.77834 | 5.299119 | 11.8272 | 1.79E-28 | 1.34E-26 | 53.849 |
| PARVG | 0.77859 | 2.451013 | 10.1658 | 4.22E-22 | 1.28E-20 | 39.3543 |
| ISG15 | 0.77866 | 6.22974 | 6.10263 | 2.17E-09 | 1.49E-08 | 10.6262 |
| HSPA6 | 0.77889 | 2.065184 | 6.21004 | 1.16E-09 | 8.22E-09 | 11.2367 |
| LOXL3 | 0.77918 | 3.651295 | 11.1825 | 6.19E-26 | 3.14E-24 | 48.0712 |
| CLEC18B | 0.77955 | 1.206936 | 10.8126 | 1.63E-24 | 6.80E-23 | 44.8418 |
| RBM47 | 0.78004 | 1.699445 | 10.781 | 2.14E-24 | 8.85E-23 | 44.5688 |
| RMI2 | 0.78029 | 2.05318 | 9.68019 | 2.38E-20 | 5.74E-19 | 35.3775 |
| FNDC3B | 0.78057 | 2.506928 | 14.2769 | 9.63E-39 | 5.17E-36 | 77.248 |
| RPS6KA1 | 0.78123 | 3.616935 | 9.84117 | 6.34E-21 | 1.65E-19 | 36.6816 |
| TRIM22 | 0.78138 | 4.278533 | 8.83867 | 1.89E-17 | 3.24E-16 | 28.8017 |
| PIK3AP1 | 0.78256 | 3.181948 | 10.0049 | 1.63E-21 | 4.58E-20 | 38.0221 |
| MAOB | 0.78277 | 5.838347 | 4.13251 | 4.24E-05 | 0.000164 | 1.11732 |
| CD69 | 0.78283 | 1.337561 | 9.54619 | 7.08E-20 | 1.61E-18 | 34.303 |
| DOK3 | 0.78309 | 2.452607 | 12.0072 | 3.39E-29 | 2.76E-27 | 55.4938 |
| NEK6 | 0.78326 | 5.11183 | 10.3399 | 9.65E-23 | 3.16E-21 | 40.8105 |
| SP100 | 0.78376 | 2.148397 | 11.5029 | 3.46E-27 | 2.13E-25 | 50.9197 |
| S100A8 | 0.78431 | 2.625894 | 5.57949 | 4.05E-08 | 2.38E-07 | 7.78457 |
| BGN | 0.78448 | 6.503714 | 7.23549 | 1.87E-12 | 1.84E-11 | 17.5086 |
| LY86 | 0.78509 | 4.944714 | 7.93773 | 1.49E-14 | 1.84E-13 | 22.2438 |
| IRX1 | 0.78523 | 3.122307 | 4.65065 | 4.29E-06 | 1.92E-05 | 3.29709 |
| LYN | 0.78654 | 3.651442 | 10.8193 | 1.53E-24 | 6.46E-23 | 44.8991 |
| FAM114A1 | 0.78813 | 2.930968 | 10.2817 | 1.58E-22 | 5.03E-21 | 40.3216 |
| NCAPG2 | 0.78836 | 3.301794 | 13.4713 | 2.86E-35 | 6.80E-33 | 69.3302 |
| CYBA | 0.78838 | 4.831948 | 8.96233 | 7.26E-18 | 1.31E-16 | 29.7418 |
| CCL2 | 0.78857 | 4.200507 | 5.09895 | 4.94E-07 | 2.53E-06 | 5.37182 |
| DPY19L1 | 0.78934 | 5.007357 | 10.8929 | 8.05E-25 | 3.56E-23 | 45.5369 |
| CYFIP1 | 0.78965 | 5.519419 | 12.5961 | 1.34E-31 | 1.60E-29 | 60.965 |
| XRCC2 | 0.78978 | 1.464416 | 12.3236 | 1.76E-30 | 1.72E-28 | 58.4171 |
| ELN | 0.78994 | 5.094504 | 8.44058 | 3.83E-16 | 5.62E-15 | 25.8398 |
| ELF4 | 0.79061 | 2.001545 | 11.3162 | 1.87E-26 | 1.03E-24 | 49.2543 |
| RCC2 | 0.79112 | 6.260328 | 14.705 | 1.28E-40 | 1.13E-37 | 81.5305 |
| LGALS9 | 0.79115 | 4.250576 | 9.07893 | 2.93E-18 | 5.46E-17 | 30.6367 |
| C2 | 0.79129 | 2.14639 | 9.26333 | 6.84E-19 | 1.37E-17 | 32.0684 |
| IFI6 | 0.7916 | 8.044144 | 6.6382 | 8.68E-11 | 7.09E-10 | 13.7591 |
| ITGA2 | 0.79189 | 2.263836 | 9.01007 | 5.01E-18 | 9.16E-17 | 30.1072 |
| KNTC1 | 0.79276 | 2.097282 | 13.8015 | 1.11E-36 | 3.44E-34 | 72.5524 |
| SLC16A3 | 0.79284 | 2.557707 | 10.3442 | 9.31E-23 | 3.06E-21 | 40.8463 |
| CXCR4 | 0.79295 | 4.984135 | 8.13579 | 3.60E-15 | 4.75E-14 | 23.6404 |
| BRCA1 | 0.79316 | 2.310533 | 15.1759 | 1.04E-42 | 1.73E-39 | 86.2948 |
| B3GNT5 | 0.79348 | 1.799159 | 10.3873 | 6.44E-23 | 2.16E-21 | 41.2099 |
| MCM8 | 0.79375 | 2.131316 | 14.7394 | 8.99E-41 | 8.35E-38 | 81.8764 |
| RPE65 | 0.79391 | 3.469416 | 5.51506 | 5.73E-08 | 3.30E-07 | 7.44994 |
| RNF135 | 0.79397 | 3.012784 | 11.2923 | 2.32E-26 | 1.25E-24 | 49.0426 |
| EMILIN1 | 0.79403 | 4.410591 | 9.79527 | 9.26E-21 | 2.36E-19 | 36.3083 |
| CHEK1 | 0.7945 | 2.364265 | 13.2576 | 2.31E-34 | 4.65E-32 | 67.264 |
| LUM | 0.79592 | 1.896734 | 7.65306 | 1.10E-13 | 1.24E-12 | 20.283 |
| CASP4 | 0.79636 | 1.777904 | 12.3606 | 1.25E-30 | 1.24E-28 | 58.7609 |
| PLAUR | 0.79664 | 2.171583 | 9.53501 | 7.75E-20 | 1.75E-18 | 34.2138 |
| NDE1 | 0.79695 | 3.308669 | 13.5171 | 1.83E-35 | 4.51E-33 | 69.7751 |
| NFIA-AS2 | 0.79743 | 3.454964 | 6.97343 | 1.04E-11 | 9.43E-11 | 15.8312 |
| HOXA7 | 0.79844 | 0.491356 | 8.79921 | 2.55E-17 | 4.32E-16 | 28.5038 |
| ANXA2P2 | 0.79849 | 1.262584 | 9.89163 | 4.18E-21 | 1.11E-19 | 37.0933 |
| SEC61G | 0.79868 | 5.845392 | 7.85078 | 2.76E-14 | 3.32E-13 | 21.6391 |
| IL13RA1 | 0.79892 | 5.235681 | 9.89443 | 4.08E-21 | 1.09E-19 | 37.1162 |
| DSEL | 0.79895 | 3.978402 | 11.5161 | 3.07E-27 | 1.90E-25 | 51.0385 |
| WNT5A-AS1 | 0.80073 | 1.638604 | 11.026 | 2.49E-25 | 1.17E-23 | 46.6975 |
| BCAT1 | 0.80104 | 2.595445 | 7.28782 | 1.32E-12 | 1.32E-11 | 17.8496 |
| ADGRE5 | 0.80114 | 3.356041 | 9.51265 | 9.28E-20 | 2.07E-18 | 34.0356 |
| ELK3 | 0.80141 | 4.051212 | 11.6997 | 5.77E-28 | 4.01E-26 | 52.6924 |
| HMOX1 | 0.80163 | 5.428248 | 8.6927 | 5.76E-17 | 9.33E-16 | 27.7042 |
| LRRC55 | 0.80185 | 3.913103 | 6.51013 | 1.91E-10 | 1.50E-09 | 12.9898 |
| ST8SIA4 | 0.80242 | 2.297743 | 12.573 | 1.67E-31 | 1.95E-29 | 60.748 |
| INPP5D | 0.80245 | 3.888131 | 10.5546 | 1.53E-23 | 5.57E-22 | 42.6275 |
| UBE2T | 0.80246 | 3.948774 | 8.75847 | 3.49E-17 | 5.80E-16 | 28.1971 |
| BTK | 0.8029 | 2.644606 | 10.7955 | 1.89E-24 | 7.86E-23 | 44.694 |
| ADA2 | 0.80309 | 3.862322 | 9.95564 | 2.45E-21 | 6.75E-20 | 37.6175 |
| RBBP8 | 0.80326 | 3.636841 | 13.2883 | 1.71E-34 | 3.57E-32 | 67.5603 |
| GPR34 | 0.80405 | 5.500206 | 7.45923 | 4.16E-13 | 4.42E-12 | 18.9799 |
| LRRN4CL | 0.80463 | 1.900527 | 6.82157 | 2.75E-11 | 2.37E-10 | 14.8821 |
| FZD2 | 0.80465 | 2.537244 | 10.4436 | 3.98E-23 | 1.37E-21 | 41.6855 |
| TUBA1C | 0.80482 | 3.187109 | 9.40562 | 2.20E-19 | 4.69E-18 | 33.1867 |
| FERMT3 | 0.80502 | 4.043129 | 9.80408 | 8.61E-21 | 2.20E-19 | 36.3799 |
| KLHL4 | 0.80518 | 2.832094 | 7.61692 | 1.41E-13 | 1.58E-12 | 20.0381 |
| ADAM9 | 0.80551 | 5.503002 | 13.0462 | 1.80E-33 | 2.90E-31 | 65.2347 |
| PSRC1 | 0.8065 | 5.201746 | 8.39437 | 5.40E-16 | 7.77E-15 | 25.5025 |
| LAMB1 | 0.80677 | 3.246525 | 7.70421 | 7.72E-14 | 8.85E-13 | 20.6313 |
| SPRY1 | 0.80689 | 3.086609 | 8.47709 | 2.92E-16 | 4.34E-15 | 26.1073 |
| BRIP1 | 0.80727 | 0.913412 | 12.653 | 7.81E-32 | 9.62E-30 | 61.5012 |
| CENPW | 0.80756 | 2.848567 | 10.9158 | 6.58E-25 | 2.94E-23 | 45.7359 |
| CLCF1 | 0.80778 | 1.56726 | 8.58148 | 1.34E-16 | 2.07E-15 | 26.8768 |
| C1orf226 | 0.80796 | 4.174158 | 10.1385 | 5.31E-22 | 1.58E-20 | 39.1271 |
| CDC7 | 0.80816 | 3.490338 | 12.3973 | 8.82E-31 | 9.11E-29 | 59.1028 |
| IL10RA | 0.80832 | 3.035933 | 9.28663 | 5.68E-19 | 1.15E-17 | 32.2507 |
| PCNA | 0.80888 | 6.781405 | 14.7728 | 6.40E-41 | 6.27E-38 | 82.213 |
| COL8A1 | 0.8089 | 0.976426 | 7.56763 | 1.98E-13 | 2.18E-12 | 19.7055 |
| COL8A2 | 0.80943 | 2.534734 | 8.08661 | 5.13E-15 | 6.68E-14 | 23.2912 |
| EEF1AKMT3 | 0.80974 | 3.064262 | 10.6938 | 4.59E-24 | 1.80E-22 | 43.8177 |
| NFAM1 | 0.81167 | 2.416641 | 11.2152 | 4.62E-26 | 2.40E-24 | 48.3605 |
| ESCO2 | 0.81212 | 0.886959 | 12.5388 | 2.31E-31 | 2.65E-29 | 60.427 |
| NAMPT | 0.81315 | 4.456867 | 9.66175 | 2.77E-20 | 6.62E-19 | 35.2291 |
| PLA2G4A | 0.81407 | 3.018167 | 10.203 | 3.08E-22 | 9.50E-21 | 39.6643 |
| OAS3 | 0.81463 | 3.531148 | 7.78896 | 4.27E-14 | 5.03E-13 | 21.2122 |
| TENT5A | 0.81513 | 2.684452 | 10.5672 | 1.37E-23 | 5.03E-22 | 42.7347 |
| PROS1 | 0.81577 | 4.238369 | 9.82141 | 7.46E-21 | 1.93E-19 | 36.5208 |
| RDH10 | 0.81589 | 3.280766 | 9.10442 | 2.40E-18 | 4.52E-17 | 30.8334 |
| PLBD1 | 0.81608 | 1.901187 | 10.5935 | 1.09E-23 | 4.08E-22 | 42.9594 |
| DUSP10 | 0.81636 | 3.005613 | 10.5114 | 2.22E-23 | 7.88E-22 | 42.2599 |
| LIF | 0.81645 | 1.160317 | 7.93732 | 1.50E-14 | 1.85E-13 | 22.241 |
| FREM2 | 0.8171 | 1.510933 | 8.02878 | 7.78E-15 | 9.91E-14 | 22.8826 |
| ARHGDIB | 0.81755 | 6.451739 | 11.0555 | 1.91E-25 | 9.15E-24 | 46.9551 |
| PDIA5 | 0.81777 | 2.148924 | 12.7249 | 3.94E-32 | 5.17E-30 | 62.1793 |
| CD276 | 0.818 | 4.812796 | 13.0761 | 1.34E-33 | 2.29E-31 | 65.5208 |
| TAGLN2 | 0.81804 | 6.341035 | 8.21741 | 1.99E-15 | 2.70E-14 | 24.2234 |
| CD37 | 0.81823 | 3.774175 | 8.36752 | 6.59E-16 | 9.39E-15 | 25.3071 |
| STIL | 0.81839 | 1.407518 | 14.5162 | 8.63E-40 | 5.77E-37 | 79.6365 |
| SYK | 0.81974 | 3.69157 | 9.08417 | 2.81E-18 | 5.26E-17 | 30.6771 |
| CDK6 | 0.82034 | 3.493505 | 7.96036 | 1.27E-14 | 1.58E-13 | 22.4021 |
| MILR1 | 0.82042 | 2.838492 | 9.81749 | 7.71E-21 | 1.99E-19 | 36.4889 |
| SLA | 0.8215 | 3.312112 | 9.29189 | 5.45E-19 | 1.10E-17 | 32.2919 |
| AL390755.1 | 0.82181 | 3.95202 | 6.01763 | 3.54E-09 | 2.37E-08 | 10.1496 |
| MFAP2 | 0.82361 | 1.034503 | 9.34016 | 3.71E-19 | 7.70E-18 | 32.6708 |
| SPINK8 | 0.82382 | 2.335905 | 8.79833 | 2.57E-17 | 4.34E-16 | 28.4971 |
| GIMAP2 | 0.82454 | 3.229297 | 12.0861 | 1.63E-29 | 1.38E-27 | 56.2188 |
| TMSB4XP8 | 0.82463 | 4.978639 | 8.60825 | 1.09E-16 | 1.71E-15 | 27.0752 |
| MMP9 | 0.82516 | 1.099773 | 6.70714 | 5.65E-11 | 4.72E-10 | 14.1783 |
| ANTXR2 | 0.8262 | 2.382492 | 12.6941 | 5.28E-32 | 6.74E-30 | 61.889 |
| MAP3K1 | 0.82696 | 3.338883 | 12.6815 | 5.95E-32 | 7.54E-30 | 61.7699 |
| H3C6 | 0.82713 | 1.595 | 9.30055 | 5.09E-19 | 1.03E-17 | 32.3598 |
| PLEKHG2 | 0.82745 | 3.523084 | 12.4866 | 3.79E-31 | 4.16E-29 | 59.9375 |
| HLA-B | 0.82799 | 8.835342 | 9.24969 | 7.62E-19 | 1.52E-17 | 31.9618 |
| IGSF6 | 0.82808 | 3.344141 | 10.0023 | 1.66E-21 | 4.68E-20 | 38.0008 |
| SLC37A2 | 0.82824 | 2.508915 | 10.3707 | 7.42E-23 | 2.47E-21 | 41.0696 |
| ITPRIPL1 | 0.82847 | 1.718131 | 10.215 | 2.79E-22 | 8.62E-21 | 39.7644 |
| KPNA2 | 0.82878 | 5.592686 | 13.2657 | 2.13E-34 | 4.33E-32 | 67.3422 |
| SLC16A4 | 0.82921 | 3.460618 | 9.81264 | 8.02E-21 | 2.07E-19 | 36.4494 |
| LPAR6 | 0.82938 | 3.616398 | 11.0701 | 1.68E-25 | 8.10E-24 | 47.0829 |
| DDIAS | 0.82943 | 1.295355 | 13.5167 | 1.83E-35 | 4.51E-33 | 69.7718 |
| SLC2A10 | 0.82978 | 2.621868 | 8.34137 | 8.00E-16 | 1.13E-14 | 25.1173 |
| MYOF | 0.83 | 3.081192 | 9.63016 | 3.58E-20 | 8.44E-19 | 34.9752 |
| GJC1 | 0.8301 | 2.055578 | 11.1391 | 9.11E-26 | 4.54E-24 | 47.6893 |
| DNALI1 | 0.83089 | 4.493911 | 10.1379 | 5.34E-22 | 1.59E-20 | 39.1221 |
| GPX8 | 0.83153 | 1.115361 | 8.96508 | 7.11E-18 | 1.28E-16 | 29.7628 |
| LINC01579 | 0.83185 | 0.97884 | 7.08011 | 5.20E-12 | 4.89E-11 | 16.508 |
| LAT2 | 0.83208 | 3.707573 | 9.64164 | 3.26E-20 | 7.72E-19 | 35.0674 |
| ZFP36L2 | 0.83227 | 6.850456 | 12.7423 | 3.33E-32 | 4.48E-30 | 62.3441 |
| SLN | 0.83248 | 2.507671 | 4.65278 | 4.25E-06 | 1.91E-05 | 3.30656 |
| LILRB1 | 0.83257 | 2.31332 | 10.1927 | 3.36E-22 | 1.03E-20 | 39.5779 |
| F2RL1 | 0.83288 | 2.280382 | 7.32674 | 1.02E-12 | 1.03E-11 | 18.1044 |
| TRIP13 | 0.83305 | 2.075088 | 11.9657 | 4.98E-29 | 4.00E-27 | 55.1139 |
| SPI1 | 0.83333 | 5.072036 | 8.91585 | 1.04E-17 | 1.85E-16 | 29.3874 |
| CD248 | 0.83387 | 2.705331 | 7.78189 | 4.48E-14 | 5.28E-13 | 21.1636 |
| REST | 0.83416 | 3.007477 | 12.5433 | 2.22E-31 | 2.55E-29 | 60.4693 |
| PLK4 | 0.83422 | 1.830197 | 12.6992 | 5.03E-32 | 6.44E-30 | 61.9369 |
| ACTN1 | 0.83453 | 4.416941 | 7.91683 | 1.73E-14 | 2.13E-13 | 22.098 |
| MAP3K7CL | 0.83511 | 1.580716 | 9.0931 | 2.62E-18 | 4.92E-17 | 30.746 |
| PKMYT1 | 0.83526 | 2.24747 | 9.73644 | 1.50E-20 | 3.71E-19 | 35.8316 |
| TMIGD3 | 0.8353 | 4.505795 | 8.52212 | 2.09E-16 | 3.16E-15 | 26.4384 |
| CMTM3 | 0.83555 | 5.542587 | 13.4178 | 4.84E-35 | 1.10E-32 | 68.8117 |
| HLA-DQB2 | 0.83556 | 1.721505 | 6.62092 | 9.66E-11 | 7.85E-10 | 13.6546 |
| ARC | 0.83734 | 5.013251 | 5.96664 | 4.73E-09 | 3.12E-08 | 9.86642 |
| RECQL | 0.8378 | 3.863868 | 12.8129 | 1.70E-32 | 2.36E-30 | 63.0124 |
| IFI16 | 0.83783 | 5.159343 | 11.5548 | 2.16E-27 | 1.36E-25 | 51.386 |
| SALL3 | 0.83901 | 4.082434 | 7.96785 | 1.20E-14 | 1.51E-13 | 22.4546 |
| OSMR | 0.84071 | 2.944291 | 8.79683 | 2.60E-17 | 4.39E-16 | 28.4858 |
| POC1A | 0.84116 | 2.674527 | 11.7066 | 5.42E-28 | 3.78E-26 | 52.7542 |
| CHEK2 | 0.84157 | 1.830043 | 16.6457 | 2.31E-49 | 2.53E-45 | 101.469 |
| ZNF217 | 0.84273 | 2.865004 | 12.9789 | 3.44E-33 | 5.25E-31 | 64.5918 |
| HOXC10 | 0.84281 | 0.49417 | 7.96644 | 1.22E-14 | 1.52E-13 | 22.4447 |
| C5AR1 | 0.84286 | 3.075976 | 7.86933 | 2.42E-14 | 2.93E-13 | 21.7677 |
| C1RL | 0.84349 | 2.788454 | 9.13497 | 1.88E-18 | 3.59E-17 | 31.0696 |
| C1S | 0.84469 | 4.569503 | 7.54724 | 2.28E-13 | 2.49E-12 | 19.5684 |
| POGLUT3 | 0.84475 | 3.42497 | 10.444 | 3.97E-23 | 1.37E-21 | 41.6884 |
| CMTM6 | 0.84507 | 4.906582 | 17.503 | 2.57E-53 | 7.03E-49 | 110.49 |
| CAV1 | 0.84529 | 4.267502 | 7.19527 | 2.44E-12 | 2.38E-11 | 17.248 |
| CREB5 | 0.84605 | 3.569243 | 11.2965 | 2.23E-26 | 1.21E-24 | 49.0797 |
| C1orf162 | 0.84623 | 3.688251 | 9.63501 | 3.44E-20 | 8.13E-19 | 35.0142 |
| COL14A1 | 0.84654 | 1.806846 | 8.42366 | 4.35E-16 | 6.34E-15 | 25.7161 |
| NEMP1 | 0.84661 | 3.665335 | 15.6914 | 5.07E-45 | 1.74E-41 | 91.5675 |
| PLVAP | 0.84668 | 3.975636 | 9.16712 | 1.46E-18 | 2.83E-17 | 31.3188 |
| S100A10 | 0.84679 | 6.527917 | 6.83873 | 2.46E-11 | 2.14E-10 | 14.9885 |
| CXCL9 | 0.84696 | 0.962416 | 8.97386 | 6.64E-18 | 1.20E-16 | 29.8299 |
| GLIS3 | 0.84715 | 3.087758 | 8.71503 | 4.86E-17 | 7.94E-16 | 27.8713 |
| CENPH | 0.8477 | 3.175401 | 13.003 | 2.72E-33 | 4.22E-31 | 64.8219 |
| C3AR1 | 0.84784 | 5.009086 | 7.83305 | 3.13E-14 | 3.75E-13 | 21.5164 |
| EMILIN2 | 0.84799 | 1.742383 | 8.95325 | 7.79E-18 | 1.40E-16 | 29.6724 |
| MND1 | 0.84864 | 1.590292 | 11.8649 | 1.26E-28 | 9.60E-27 | 54.1927 |
| NRM | 0.84951 | 4.086687 | 12.6555 | 7.63E-32 | 9.42E-30 | 61.5243 |
| HK2 | 0.85053 | 3.805019 | 9.33207 | 3.96E-19 | 8.17E-18 | 32.6072 |
| WWTR1 | 0.85105 | 4.407913 | 8.39436 | 5.40E-16 | 7.77E-15 | 25.5024 |
| LEFTY2 | 0.85144 | 1.639638 | 5.93306 | 5.73E-09 | 3.75E-08 | 9.68107 |
| NES | 0.85153 | 8.163706 | 8.39377 | 5.43E-16 | 7.81E-15 | 25.4981 |
| TLR7 | 0.85172 | 2.604524 | 8.93613 | 8.89E-18 | 1.59E-16 | 29.5418 |
| WDR62 | 0.85239 | 1.346531 | 11.5551 | 2.16E-27 | 1.36E-25 | 51.3888 |
| KIF18A | 0.85303 | 0.976464 | 13.5733 | 1.05E-35 | 2.74E-33 | 70.3223 |
| EGFR | 0.85316 | 6.499868 | 6.09848 | 2.22E-09 | 1.53E-08 | 10.6028 |
| EME1 | 0.85481 | 1.870804 | 12.8114 | 1.72E-32 | 2.39E-30 | 62.9984 |
| ANO6 | 0.85494 | 4.589969 | 12.1191 | 1.20E-29 | 1.04E-27 | 56.5234 |
| MYO1F | 0.85532 | 3.152198 | 10.9413 | 5.25E-25 | 2.39E-23 | 45.9583 |
| FBP1 | 0.85535 | 2.668886 | 10.5148 | 2.16E-23 | 7.67E-22 | 42.2889 |
| ITGB4 | 0.85657 | 5.060992 | 6.25981 | 8.61E-10 | 6.24E-09 | 11.5226 |
| LRRC25 | 0.85705 | 2.681143 | 9.93663 | 2.87E-21 | 7.83E-20 | 37.4616 |
| CD84 | 0.85785 | 2.69966 | 9.44789 | 1.56E-19 | 3.40E-18 | 33.5212 |
| CCR5 | 0.85791 | 1.403822 | 10.5494 | 1.60E-23 | 5.80E-22 | 42.5836 |
| RAC2 | 0.85897 | 2.768979 | 10.1401 | 5.24E-22 | 1.56E-20 | 39.1406 |
| CD300A | 0.85943 | 3.567446 | 10.2054 | 3.02E-22 | 9.33E-21 | 39.6843 |
| LRRC17 | 0.86046 | 3.546479 | 8.01958 | 8.31E-15 | 1.06E-13 | 22.8178 |
| TMEM255A | 0.86084 | 4.90809 | 9.34345 | 3.61E-19 | 7.52E-18 | 32.6967 |
| FANCD2 | 0.86173 | 1.863117 | 13.5469 | 1.36E-35 | 3.47E-33 | 70.0656 |
| STAT1 | 0.86181 | 5.881738 | 10.931 | 5.75E-25 | 2.60E-23 | 45.8685 |
| POSTN | 0.86234 | 0.977807 | 5.40851 | 1.01E-07 | 5.64E-07 | 6.90404 |
| SDC1 | 0.86302 | 2.163034 | 9.42615 | 1.86E-19 | 4.01E-18 | 33.349 |
| SGO2 | 0.86376 | 1.897193 | 14.6229 | 2.93E-40 | 2.40E-37 | 80.7054 |
| CENPM | 0.86489 | 2.51935 | 10.2431 | 2.20E-22 | 6.86E-21 | 39.9988 |
| DEPDC1 | 0.8652 | 0.770522 | 12.6867 | 5.67E-32 | 7.21E-30 | 61.8188 |
| GAL3ST4 | 0.86558 | 4.816872 | 12.7691 | 2.58E-32 | 3.54E-30 | 62.5979 |
| MS4A4A | 0.86594 | 3.443363 | 8.2615 | 1.44E-15 | 1.98E-14 | 24.5402 |
| CALU | 0.86608 | 6.04556 | 14.0075 | 1.43E-37 | 5.32E-35 | 74.5795 |
| GPR183 | 0.86622 | 3.14655 | 8.2225 | 1.91E-15 | 2.60E-14 | 24.26 |
| TFPI | 0.86672 | 1.817988 | 8.12016 | 4.03E-15 | 5.29E-14 | 23.5292 |
| MXRA5 | 0.86719 | 1.475305 | 8.79409 | 2.66E-17 | 4.47E-16 | 28.4652 |
| RAB32 | 0.86768 | 3.924004 | 10.694 | 4.58E-24 | 1.80E-22 | 43.8196 |
| SIGLEC10 | 0.86809 | 4.070262 | 9.44393 | 1.62E-19 | 3.50E-18 | 33.4898 |
| TYROBP | 0.86814 | 6.99539 | 8.90829 | 1.10E-17 | 1.95E-16 | 29.3298 |
| CD86 | 0.86817 | 3.361083 | 9.41749 | 2.00E-19 | 4.29E-18 | 33.2806 |
| BARD1 | 0.86854 | 2.806078 | 14.4111 | 2.49E-39 | 1.45E-36 | 78.586 |
| CARD16 | 0.86913 | 1.865567 | 10.2408 | 2.24E-22 | 6.98E-21 | 39.9793 |
| SIGLEC9 | 0.8699 | 2.324193 | 10.4027 | 5.65E-23 | 1.91E-21 | 41.3396 |
| CAPN5 | 0.87033 | 4.835605 | 10.9233 | 6.16E-25 | 2.77E-23 | 45.8011 |
| GEM | 0.87072 | 4.202539 | 7.41833 | 5.49E-13 | 5.73E-12 | 18.7083 |
| CD14 | 0.87141 | 6.385504 | 7.30615 | 1.17E-12 | 1.18E-11 | 17.9694 |
| E2F7 | 0.87317 | 0.877468 | 11.9425 | 6.18E-29 | 4.88E-27 | 54.9009 |
| PDIA4 | 0.87397 | 6.465939 | 14.1122 | 5.02E-38 | 2.15E-35 | 75.614 |
| MYD88 | 0.87423 | 3.949349 | 10.8879 | 8.41E-25 | 3.70E-23 | 45.4937 |
| ITGA5 | 0.87537 | 3.060782 | 10.5644 | 1.41E-23 | 5.15E-22 | 42.7108 |
| TMEM119 | 0.87711 | 4.639308 | 7.39058 | 6.62E-13 | 6.84E-12 | 18.5247 |
| PLP2 | 0.87727 | 4.643193 | 7.95964 | 1.28E-14 | 1.59E-13 | 22.3971 |
| CNN3 | 0.87748 | 8.597016 | 12.6676 | 6.80E-32 | 8.45E-30 | 61.6384 |
| HAMP | 0.88059 | 1.889638 | 7.55556 | 2.16E-13 | 2.36E-12 | 19.6243 |
| ANGPT2 | 0.88183 | 2.281965 | 8.29357 | 1.14E-15 | 1.58E-14 | 24.7714 |
| RNASE6 | 0.88237 | 4.821997 | 9.36867 | 2.95E-19 | 6.21E-18 | 32.8952 |
| NCKAP1L | 0.88283 | 3.199473 | 10.4113 | 5.25E-23 | 1.78E-21 | 41.4121 |
| DTX3L | 0.88338 | 4.112368 | 12.6461 | 8.34E-32 | 1.02E-29 | 61.4364 |
| PLEK | 0.88382 | 3.662585 | 9.4537 | 1.49E-19 | 3.26E-18 | 33.5673 |
| CLIC4 | 0.88439 | 7.524552 | 13.634 | 5.79E-36 | 1.60E-33 | 70.9135 |
| MIS18BP1 | 0.88562 | 2.437928 | 14.8749 | 2.26E-41 | 2.48E-38 | 83.2436 |
| IFI44L | 0.88582 | 3.874662 | 7.04496 | 6.54E-12 | 6.09E-11 | 16.2841 |
| TNFRSF19 | 0.88611 | 3.729485 | 8.64936 | 8.00E-17 | 1.27E-15 | 27.3808 |
| CLSPN | 0.88694 | 1.15752 | 11.919 | 7.67E-29 | 6.02E-27 | 54.6867 |
| SPATA6 | 0.88747 | 3.373629 | 8.28558 | 1.21E-15 | 1.67E-14 | 24.7138 |
| APBB1IP | 0.88853 | 4.307275 | 8.73983 | 4.02E-17 | 6.64E-16 | 28.0571 |
| PDGFD | 0.88943 | 1.652294 | 8.33996 | 8.08E-16 | 1.14E-14 | 25.1071 |
| SOD2 | 0.88975 | 5.461826 | 8.72463 | 4.52E-17 | 7.41E-16 | 27.9431 |
| OAS1 | 0.89046 | 3.075679 | 8.46096 | 3.29E-16 | 4.87E-15 | 25.9891 |
| TGFB2-AS1 | 0.89085 | 2.650242 | 7.61698 | 1.41E-13 | 1.58E-12 | 20.0385 |
| TCF19 | 0.89106 | 3.274626 | 12.366 | 1.18E-30 | 1.19E-28 | 58.8111 |
| LAMC1 | 0.89139 | 4.215442 | 10.9491 | 4.90E-25 | 2.23E-23 | 46.0259 |
| LHFPL2 | 0.89209 | 4.119875 | 12.3925 | 9.23E-31 | 9.45E-29 | 59.0587 |
| LINC02587 | 0.89228 | 0.882262 | 6.39175 | 3.92E-10 | 2.96E-09 | 12.2899 |
| KLHDC8A | 0.89263 | 4.764474 | 7.62692 | 1.32E-13 | 1.48E-12 | 20.1058 |
| NEIL3 | 0.89484 | 0.917318 | 13.0814 | 1.28E-33 | 2.20E-31 | 65.5709 |
| PDCD1LG2 | 0.89524 | 1.551543 | 11.231 | 4.01E-26 | 2.10E-24 | 48.4997 |
| TEAD4 | 0.89623 | 2.203098 | 9.21986 | 9.65E-19 | 1.91E-17 | 31.7291 |
| SLAMF8 | 0.89643 | 1.763949 | 9.80715 | 8.39E-21 | 2.15E-19 | 36.4048 |
| ALOX5 | 0.89654 | 3.244256 | 9.55934 | 6.36E-20 | 1.46E-18 | 34.408 |
| IKBIP | 0.89657 | 2.676219 | 16.3291 | 6.49E-48 | 4.44E-44 | 98.166 |
| LCP1 | 0.89715 | 4.269988 | 10.2083 | 2.95E-22 | 9.11E-21 | 39.708 |
| AC090692.1 | 0.89805 | 2.427308 | 8.62008 | 9.98E-17 | 1.57E-15 | 27.1631 |
| MGME1 | 0.89819 | 4.327572 | 25.3508 | 2.36E-90 | 1.29E-85 | 194.926 |
| OAS2 | 0.89949 | 2.845675 | 8.92877 | 9.42E-18 | 1.68E-16 | 29.4858 |
| TGFBR1 | 0.90045 | 4.704927 | 15.7756 | 2.11E-45 | 7.72E-42 | 92.4338 |
| PTAFR | 0.90091 | 3.574585 | 9.13672 | 1.86E-18 | 3.54E-17 | 31.0832 |
| HOXA10 | 0.90253 | 0.70459 | 8.32353 | 9.12E-16 | 1.28E-14 | 24.988 |
| RAD51 | 0.90488 | 1.586038 | 12.4959 | 3.47E-31 | 3.86E-29 | 60.0247 |
| LY96 | 0.9056 | 3.893165 | 9.92313 | 3.22E-21 | 8.73E-20 | 37.351 |
| DMRTA2 | 0.90654 | 0.99097 | 7.43817 | 4.80E-13 | 5.06E-12 | 18.8399 |
| TNFAIP6 | 0.9071 | 1.729264 | 8.21718 | 1.99E-15 | 2.70E-14 | 24.2218 |
| C4B | 0.90823 | 3.275377 | 7.66246 | 1.03E-13 | 1.17E-12 | 20.3469 |
| RCC1 | 0.90851 | 3.96526 | 13.1581 | 6.08E-34 | 1.11E-31 | 66.3068 |
| NID1 | 0.90901 | 4.729207 | 9.75612 | 1.28E-20 | 3.19E-19 | 35.9909 |
| IL1RAP | 0.90912 | 2.425706 | 9.44624 | 1.59E-19 | 3.44E-18 | 33.5081 |
| PTGFRN | 0.90925 | 4.429092 | 10.0618 | 1.01E-21 | 2.91E-20 | 38.4922 |
| SULF1 | 0.90966 | 3.206292 | 6.39897 | 3.75E-10 | 2.84E-09 | 12.3323 |
| RNF122 | 0.90981 | 3.934552 | 12.124 | 1.14E-29 | 1.00E-27 | 56.5685 |
| SLC43A3 | 0.90991 | 2.486289 | 9.58146 | 5.32E-20 | 1.23E-18 | 34.5849 |
| BCL2A1 | 0.90998 | 2.081397 | 8.2944 | 1.13E-15 | 1.57E-14 | 24.7774 |
| LILRB4 | 0.91065 | 3.819589 | 9.33261 | 3.94E-19 | 8.14E-18 | 32.6114 |
| PARP9 | 0.91098 | 3.725618 | 12.5253 | 2.63E-31 | 2.98E-29 | 60.3005 |
| NCAPD2 | 0.91155 | 4.845041 | 16.6548 | 2.10E-49 | 2.53E-45 | 101.564 |
| RUNX1 | 0.91172 | 1.738724 | 10.8895 | 8.28E-25 | 3.66E-23 | 45.5081 |
| LAIR1 | 0.9135 | 3.112436 | 9.96737 | 2.23E-21 | 6.16E-20 | 37.7138 |
| CDCA7 | 0.91396 | 2.943438 | 10.079 | 8.76E-22 | 2.54E-20 | 38.634 |
| CCR1 | 0.91411 | 3.478667 | 9.19587 | 1.17E-18 | 2.29E-17 | 31.5422 |
| CDC6 | 0.91416 | 1.655195 | 12.4793 | 4.06E-31 | 4.44E-29 | 59.8697 |
| CD58 | 0.91498 | 2.953826 | 11.0303 | 2.39E-25 | 1.13E-23 | 46.7351 |
| NGFR | 0.91554 | 3.224327 | 7.25431 | 1.65E-12 | 1.64E-11 | 17.631 |
| AC008760.2 | 0.91575 | 1.174504 | 10.5341 | 1.83E-23 | 6.57E-22 | 42.4529 |
| COL11A1 | 0.91622 | 2.706451 | 6.943 | 1.26E-11 | 1.14E-10 | 15.6396 |
| IQGAP1 | 0.91682 | 4.211903 | 11.7153 | 5.00E-28 | 3.51E-26 | 52.8333 |
| S100A9 | 0.91831 | 4.202804 | 5.83678 | 9.86E-09 | 6.25E-08 | 9.15461 |
| GPX7 | 0.92078 | 3.866759 | 14.4127 | 2.46E-39 | 1.45E-36 | 78.6013 |
| TLR1 | 0.92171 | 2.166833 | 11.7419 | 3.92E-28 | 2.79E-26 | 53.0741 |
| PRR11 | 0.9224 | 2.816904 | 13.0678 | 1.46E-33 | 2.43E-31 | 65.4415 |
| GNG5 | 0.92289 | 6.620684 | 11.7828 | 2.69E-28 | 1.95E-26 | 53.4455 |
| HLA-DRB6 | 0.9262 | 3.655266 | 6.20595 | 1.18E-09 | 8.41E-09 | 11.2133 |
| C21orf62 | 0.92727 | 2.900641 | 7.04087 | 6.72E-12 | 6.25E-11 | 16.2581 |
| NNMT | 0.92807 | 2.000651 | 7.0103 | 8.19E-12 | 7.53E-11 | 16.0642 |
| AQP1 | 0.9291 | 7.46253 | 4.95612 | 1.00E-06 | 4.91E-06 | 4.69209 |
| SLC7A7 | 0.92915 | 3.046834 | 12.4135 | 7.57E-31 | 7.93E-29 | 59.2545 |
| RAB42 | 0.9295 | 1.54573 | 13.0112 | 2.52E-33 | 3.94E-31 | 64.9 |
| CDC25C | 0.92974 | 1.135558 | 13.037 | 1.96E-33 | 3.13E-31 | 65.1467 |
| ITGAL | 0.93049 | 2.14138 | 11.1971 | 5.43E-26 | 2.78E-24 | 48.2002 |
| GINS2 | 0.9305 | 2.548089 | 12.1396 | 9.89E-30 | 8.69E-28 | 56.712 |
| IFI44 | 0.93133 | 4.648992 | 10.6441 | 7.06E-24 | 2.69E-22 | 43.3922 |
| ABI3 | 0.93413 | 4.015679 | 10.5101 | 2.25E-23 | 7.95E-22 | 42.2493 |
| MRC2 | 0.93479 | 4.655127 | 9.32135 | 4.31E-19 | 8.84E-18 | 32.5229 |
| SERPING1 | 0.93506 | 5.251935 | 7.55192 | 2.21E-13 | 2.42E-12 | 19.5998 |
| DPYD | 0.9356 | 2.458752 | 10.9083 | 7.03E-25 | 3.12E-23 | 45.6709 |
| HCLS1 | 0.93812 | 4.721459 | 9.88751 | 4.32E-21 | 1.15E-19 | 37.0596 |
| CDKN3 | 0.94047 | 2.218512 | 11.7565 | 3.43E-28 | 2.46E-26 | 53.2069 |
| NPNT | 0.94272 | 2.616432 | 6.9547 | 1.17E-11 | 1.06E-10 | 15.7132 |
| FGL2 | 0.9433 | 3.271167 | 9.53628 | 7.67E-20 | 1.74E-18 | 34.224 |
| CENPK | 0.94347 | 1.314827 | 12.584 | 1.51E-31 | 1.78E-29 | 60.8516 |
| COL6A2 | 0.94388 | 3.579202 | 7.54748 | 2.28E-13 | 2.49E-12 | 19.57 |
| KIAA0040 | 0.94427 | 2.917751 | 9.05943 | 3.41E-18 | 6.33E-17 | 30.4864 |
| GINS1 | 0.94597 | 2.847124 | 13.264 | 2.17E-34 | 4.39E-32 | 67.3255 |
| ZNF367 | 0.94664 | 2.660244 | 13.2008 | 4.01E-34 | 7.83E-32 | 66.7172 |
| CIITA | 0.94677 | 1.73813 | 10.7898 | 1.99E-24 | 8.23E-23 | 44.6444 |
| JAG1 | 0.94707 | 4.225007 | 12.0864 | 1.62E-29 | 1.38E-27 | 56.2221 |
| DIRAS3 | 0.94909 | 3.064684 | 8.03459 | 7.46E-15 | 9.52E-14 | 22.9236 |
| CD53 | 0.95064 | 5.557701 | 9.97662 | 2.06E-21 | 5.73E-20 | 37.7899 |
| PTGS1 | 0.95116 | 3.275174 | 10.0292 | 1.33E-21 | 3.77E-20 | 38.2229 |
| HSPA7 | 0.9515 | 1.531783 | 8.67934 | 6.37E-17 | 1.03E-15 | 27.6044 |
| ID3 | 0.95239 | 8.168403 | 8.08209 | 5.30E-15 | 6.89E-14 | 23.2591 |
| DPEP1 | 0.95727 | 1.047969 | 8.98012 | 6.32E-18 | 1.15E-16 | 29.8778 |
| H2BC12 | 0.95809 | 4.44261 | 12.0252 | 2.87E-29 | 2.35E-27 | 55.6594 |
| KIF18B | 0.95843 | 2.641614 | 9.21954 | 9.67E-19 | 1.91E-17 | 31.7265 |
| OIP5 | 0.95951 | 1.851748 | 13.3742 | 7.41E-35 | 1.63E-32 | 68.3893 |
| EVI2B | 0.95986 | 4.072346 | 9.97557 | 2.08E-21 | 5.77E-20 | 37.7812 |
| FOXJ1 | 0.96015 | 3.476257 | 7.03795 | 6.84E-12 | 6.36E-11 | 16.2395 |
| CTSC | 0.96018 | 3.079417 | 12.0982 | 1.46E-29 | 1.25E-27 | 56.3302 |
| VSIG4 | 0.96126 | 5.965604 | 7.7523 | 5.52E-14 | 6.43E-13 | 20.9603 |
| LSP1 | 0.96162 | 2.157995 | 9.73863 | 1.47E-20 | 3.65E-19 | 35.8493 |
| FOXD1 | 0.96168 | 2.71331 | 8.41052 | 4.79E-16 | 6.94E-15 | 25.6203 |
| SGO1 | 0.96222 | 1.061803 | 13.9438 | 2.69E-37 | 9.21E-35 | 73.9516 |
| HSPG2 | 0.96232 | 2.370424 | 9.88357 | 4.46E-21 | 1.19E-19 | 37.0274 |
| DEPDC1B | 0.96289 | 1.314785 | 13.5762 | 1.02E-35 | 2.68E-33 | 70.3505 |
| DRAXIN | 0.96334 | 2.467875 | 10.0237 | 1.39E-21 | 3.94E-20 | 38.1778 |
| TNFRSF11B | 0.96503 | 1.25776 | 9.85261 | 5.77E-21 | 1.51E-19 | 36.7748 |
| PLK1 | 0.96512 | 2.637768 | 12.577 | 1.61E-31 | 1.89E-29 | 60.7853 |
| F2R | 0.96553 | 5.422002 | 10.7087 | 4.03E-24 | 1.59E-22 | 43.946 |
| HAVCR2 | 0.9661 | 4.238815 | 10.1539 | 4.67E-22 | 1.40E-20 | 39.2549 |
| FAM111A | 0.96639 | 3.287099 | 15.5792 | 1.62E-44 | 4.94E-41 | 90.4154 |
| CASP1 | 0.96668 | 2.838972 | 11.5379 | 2.52E-27 | 1.57E-25 | 51.2344 |
| MEST | 0.96687 | 5.083993 | 10.639 | 7.38E-24 | 2.80E-22 | 43.3481 |
| TIMELESS | 0.96771 | 3.590061 | 13.3788 | 7.08E-35 | 1.57E-32 | 68.4339 |
| GAS1 | 0.96799 | 4.279858 | 10.2695 | 1.76E-22 | 5.54E-21 | 40.2195 |
| SHOX2 | 0.96886 | 0.678925 | 9.0383 | 4.02E-18 | 7.41E-17 | 30.3239 |
| COL5A2 | 0.97119 | 3.028377 | 8.80263 | 2.49E-17 | 4.21E-16 | 28.5296 |
| CENPE | 0.97217 | 1.090901 | 13.8522 | 6.68E-37 | 2.15E-34 | 73.0504 |
| IL18 | 0.97847 | 3.368528 | 10.6414 | 7.23E-24 | 2.75E-22 | 43.3685 |
| LAPTM5 | 0.97918 | 7.309949 | 10.1336 | 5.54E-22 | 1.64E-20 | 39.0867 |
| CKS2 | 0.97948 | 5.186447 | 11.3676 | 1.18E-26 | 6.61E-25 | 49.712 |
| SASH3 | 0.98048 | 3.91628 | 10.6646 | 5.91E-24 | 2.29E-22 | 43.5675 |
| PTX3 | 0.98195 | 1.996733 | 9.18285 | 1.29E-18 | 2.51E-17 | 31.441 |
| MCM2 | 0.98308 | 4.057274 | 12.8684 | 9.96E-33 | 1.46E-30 | 63.5394 |
| CYTL1 | 0.98329 | 3.571733 | 9.87276 | 4.88E-21 | 1.29E-19 | 36.9392 |
| SAMSN1 | 0.98843 | 3.303151 | 10.7393 | 3.09E-24 | 1.24E-22 | 44.209 |
| PTPRC | 0.98952 | 2.817029 | 10.5798 | 1.23E-23 | 4.54E-22 | 42.8427 |
| KNL1 | 0.99078 | 1.189545 | 13.1779 | 5.01E-34 | 9.54E-32 | 66.4968 |
| WNT5A | 0.99284 | 3.260585 | 11.6268 | 1.12E-27 | 7.46E-26 | 52.0334 |
| TGIF1 | 0.99348 | 2.741515 | 12.8486 | 1.20E-32 | 1.72E-30 | 63.3515 |
| ECT2 | 0.99368 | 2.808463 | 13.7158 | 2.58E-36 | 7.68E-34 | 71.7135 |
| MCM10 | 0.99459 | 1.323006 | 11.8205 | 1.90E-28 | 1.41E-26 | 53.7883 |
| CDCA5 | 0.99505 | 3.003299 | 10.2838 | 1.56E-22 | 4.95E-21 | 40.339 |
| MEOX2 | 0.99665 | 1.091397 | 6.58641 | 1.20E-10 | 9.62E-10 | 13.4465 |
| ORC1 | 0.99752 | 1.391068 | 13.9492 | 2.55E-37 | 8.85E-35 | 74.0042 |
| AEBP1 | 1.00208 | 5.530841 | 5.60367 | 3.56E-08 | 2.10E-07 | 7.911 |
| MS4A7 | 1.00304 | 4.043645 | 10.2578 | 1.94E-22 | 6.09E-21 | 40.122 |
| CLIC1 | 1.0054 | 5.867621 | 10.7504 | 2.80E-24 | 1.13E-22 | 44.3047 |
| SIGLEC14 | 1.00648 | 2.59452 | 10.0072 | 1.60E-21 | 4.50E-20 | 38.0418 |
| UCP2 | 1.00842 | 4.93057 | 10.683 | 5.04E-24 | 1.97E-22 | 43.7253 |
| IL13RA2 | 1.00855 | 1.815502 | 6.33305 | 5.57E-10 | 4.13E-09 | 11.9469 |
| TREM2 | 1.01059 | 6.640954 | 8.61958 | 1.00E-16 | 1.57E-15 | 27.1594 |
| ABCC3 | 1.01072 | 1.130626 | 8.09688 | 4.76E-15 | 6.22E-14 | 23.364 |
| CDCA7L | 1.01112 | 3.3403 | 10.5907 | 1.12E-23 | 4.16E-22 | 42.9351 |
| CDCA2 | 1.01311 | 1.069682 | 14.2179 | 1.74E-38 | 8.60E-36 | 76.6618 |
| CAPG | 1.01363 | 5.322924 | 9.19421 | 1.18E-18 | 2.31E-17 | 31.5294 |
| NIBAN1 | 1.01544 | 2.36081 | 10.9629 | 4.34E-25 | 1.98E-23 | 46.1459 |
| GAS2L3 | 1.01664 | 1.151583 | 14.0918 | 6.15E-38 | 2.52E-35 | 75.4121 |
| PRC1 | 1.01684 | 3.356271 | 11.8415 | 1.57E-28 | 1.18E-26 | 53.9789 |
| C1QA | 1.01767 | 7.805767 | 9.58894 | 5.00E-20 | 1.16E-18 | 34.6447 |
| KIF14 | 1.0182 | 1.092046 | 14.1892 | 2.32E-38 | 1.11E-35 | 76.3775 |
| TUBB6 | 1.01842 | 3.666378 | 10.1856 | 3.57E-22 | 1.09E-20 | 39.5193 |
| RAD54L | 1.01902 | 1.606793 | 13.7406 | 2.02E-36 | 6.15E-34 | 71.9554 |
| ITGB2 | 1.02147 | 4.912263 | 9.85885 | 5.48E-21 | 1.44E-19 | 36.8257 |
| RGS16 | 1.02169 | 3.364377 | 8.69714 | 5.57E-17 | 9.04E-16 | 27.7374 |
| EXO1 | 1.02254 | 1.301788 | 12.5875 | 1.46E-31 | 1.72E-29 | 60.8843 |
| RGS1 | 1.02421 | 4.749352 | 6.83347 | 2.55E-11 | 2.21E-10 | 14.9559 |
| AC026401.3 | 1.02484 | 3.224583 | 12.5417 | 2.25E-31 | 2.58E-29 | 60.4545 |
| MCUB | 1.02497 | 2.484559 | 11.1765 | 6.52E-26 | 3.30E-24 | 48.0189 |
| FLNA | 1.02619 | 6.569307 | 12.5213 | 2.73E-31 | 3.07E-29 | 60.2631 |
| SAMD9 | 1.02704 | 2.562879 | 12.7146 | 4.34E-32 | 5.64E-30 | 62.0825 |
| GPR65 | 1.02921 | 1.439722 | 12.5683 | 1.75E-31 | 2.03E-29 | 60.7044 |
| ESPL1 | 1.03064 | 1.199167 | 13.4158 | 4.93E-35 | 1.12E-32 | 68.7926 |
| SERPINH1 | 1.0328 | 4.26308 | 11.2679 | 2.88E-26 | 1.54E-24 | 48.8265 |
| FCER1G | 1.03521 | 6.051402 | 10.3634 | 7.90E-23 | 2.61E-21 | 41.0079 |
| SKA1 | 1.03677 | 1.273558 | 13.2128 | 3.57E-34 | 7.01E-32 | 66.833 |
| HOTAIRM1 | 1.03916 | 1.468628 | 8.73034 | 4.32E-17 | 7.12E-16 | 27.9859 |
| FAM83D | 1.04059 | 1.78354 | 11.8808 | 1.09E-28 | 8.40E-27 | 54.3371 |
| CPVL | 1.04116 | 4.229588 | 10.5147 | 2.16E-23 | 7.67E-22 | 42.288 |
| FMOD | 1.0419 | 2.349088 | 6.85565 | 2.21E-11 | 1.93E-10 | 15.0936 |
| LINC02732 | 1.04281 | 1.703397 | 9.26155 | 6.93E-19 | 1.39E-17 | 32.0544 |
| AC099850.4 | 1.04375 | 3.04298 | 12.4905 | 3.66E-31 | 4.03E-29 | 59.9746 |
| AURKA | 1.04376 | 2.340212 | 14.5224 | 8.11E-40 | 5.51E-37 | 79.6985 |
| FANCI | 1.04507 | 2.663886 | 12.9352 | 5.24E-33 | 7.80E-31 | 64.1753 |
| HMMR | 1.04616 | 1.567149 | 13.6491 | 4.98E-36 | 1.39E-33 | 71.0616 |
| ZWINT | 1.04629 | 3.591578 | 12.0541 | 2.19E-29 | 1.83E-27 | 55.9242 |
| OLFML2B | 1.0474 | 3.547267 | 9.78058 | 1.04E-20 | 2.64E-19 | 36.1891 |
| SFRP4 | 1.0474 | 2.894937 | 8.20721 | 2.14E-15 | 2.90E-14 | 24.1504 |
| PLSCR1 | 1.0477 | 3.731421 | 12.3413 | 1.49E-30 | 1.47E-28 | 58.5818 |
| KIF15 | 1.04917 | 2.34256 | 13.6404 | 5.43E-36 | 1.51E-33 | 70.9766 |
| MMP2 | 1.05202 | 4.856895 | 11.7508 | 3.61E-28 | 2.58E-26 | 53.155 |
| APOBEC3C | 1.05223 | 3.632842 | 12.3513 | 1.36E-30 | 1.35E-28 | 58.6746 |
| C1QC | 1.05224 | 8.251769 | 10.1269 | 5.86E-22 | 1.73E-20 | 39.0306 |
| CD44 | 1.05447 | 5.676043 | 8.23968 | 1.69E-15 | 2.31E-14 | 24.3833 |
| CTSS | 1.05562 | 4.451737 | 11.3515 | 1.36E-26 | 7.61E-25 | 49.5685 |
| MSN | 1.05965 | 6.114863 | 10.7501 | 2.81E-24 | 1.14E-22 | 44.3024 |
| CYBB | 1.06189 | 4.897517 | 9.49832 | 1.04E-19 | 2.32E-18 | 33.9216 |
| ADAMTS15 | 1.06281 | 2.438625 | 9.27217 | 6.37E-19 | 1.28E-17 | 32.1375 |
| AL354919.2 | 1.06345 | 1.21668 | 9.15564 | 1.60E-18 | 3.08E-17 | 31.2298 |
| SOCS3 | 1.06385 | 3.544597 | 6.75381 | 4.21E-11 | 3.56E-10 | 14.4642 |
| E2F2 | 1.06534 | 1.372179 | 12.5921 | 1.40E-31 | 1.65E-29 | 60.9275 |
| FN1 | 1.06567 | 5.719356 | 11.2019 | 5.20E-26 | 2.67E-24 | 48.2424 |
| FOXD3-AS1 | 1.06617 | 1.730808 | 8.54368 | 1.77E-16 | 2.71E-15 | 26.5974 |
| HOXD8 | 1.0667 | 1.664381 | 7.58927 | 1.71E-13 | 1.89E-12 | 19.8513 |
| HLA-DMA | 1.06841 | 5.169884 | 10.5375 | 1.78E-23 | 6.40E-22 | 42.4818 |
| HLA-DQA2 | 1.06877 | 2.445144 | 6.2497 | 9.15E-10 | 6.61E-09 | 11.4643 |
| SAMD9L | 1.0691 | 3.106961 | 11.5405 | 2.46E-27 | 1.54E-25 | 51.2576 |
| EZH2 | 1.06927 | 3.368986 | 12.4865 | 3.80E-31 | 4.16E-29 | 59.9365 |
| LOXL2 | 1.06997 | 2.852381 | 11.3698 | 1.15E-26 | 6.50E-25 | 49.7314 |
| FPR1 | 1.07071 | 3.922619 | 8.55005 | 1.69E-16 | 2.59E-15 | 26.6444 |
| COL1A2 | 1.0708 | 3.450604 | 9.41053 | 2.11E-19 | 4.52E-18 | 33.2256 |
| OLFML3 | 1.07093 | 5.125341 | 12.2273 | 4.36E-30 | 4.01E-28 | 57.5225 |
| EN1 | 1.07365 | 0.787804 | 10.1039 | 7.11E-22 | 2.08E-20 | 38.8399 |
| SHCBP1 | 1.07539 | 1.78694 | 14.7121 | 1.19E-40 | 1.07E-37 | 81.6018 |
| FPR3 | 1.07551 | 2.163526 | 9.78609 | 9.98E-21 | 2.54E-19 | 36.2338 |
| ANKRD22 | 1.07686 | 2.425002 | 8.51329 | 2.23E-16 | 3.36E-15 | 26.3734 |
| FCGR2A | 1.07699 | 3.419206 | 11.1681 | 7.03E-26 | 3.54E-24 | 47.9444 |
| TLR2 | 1.07746 | 3.261778 | 10.1241 | 6.00E-22 | 1.77E-20 | 39.008 |
| CD93 | 1.07768 | 3.190174 | 10.0378 | 1.24E-21 | 3.52E-20 | 38.294 |
| PYGL | 1.0812 | 3.859163 | 12.7322 | 3.67E-32 | 4.87E-30 | 62.2481 |
| FYB1 | 1.08445 | 3.448408 | 11.8686 | 1.22E-28 | 9.33E-27 | 54.2263 |
| S100A4 | 1.08772 | 3.284167 | 8.8297 | 2.02E-17 | 3.46E-16 | 28.7339 |
| ARHGAP11A | 1.08882 | 2.04483 | 14.268 | 1.05E-38 | 5.51E-36 | 77.1603 |
| TGFBI | 1.0895 | 3.423529 | 8.55917 | 1.58E-16 | 2.43E-15 | 26.7118 |
| LINC02381 | 1.09089 | 3.792466 | 10.2592 | 1.92E-22 | 6.03E-21 | 40.1334 |
| HOXA5 | 1.09165 | 0.731893 | 8.79949 | 2.55E-17 | 4.31E-16 | 28.5059 |
| SRPX2 | 1.09302 | 2.214176 | 9.51204 | 9.33E-20 | 2.08E-18 | 34.0308 |
| FSTL1 | 1.09325 | 4.585006 | 12.2737 | 2.82E-30 | 2.66E-28 | 57.953 |
| PLAT | 1.09338 | 3.124572 | 9.5808 | 5.35E-20 | 1.24E-18 | 34.5796 |
| ANXA2 | 1.09532 | 4.052747 | 10.0397 | 1.22E-21 | 3.47E-20 | 38.3093 |
| SLC11A1 | 1.09556 | 2.618899 | 9.46957 | 1.31E-19 | 2.89E-18 | 33.6932 |
| KIF11 | 1.09557 | 2.799759 | 13.2161 | 3.46E-34 | 6.82E-32 | 66.8643 |
| STEAP3 | 1.09758 | 3.119714 | 9.03151 | 4.24E-18 | 7.79E-17 | 30.2718 |
| OLR1 | 1.10465 | 4.521389 | 8.6554 | 7.64E-17 | 1.22E-15 | 27.4258 |
| CP | 1.10978 | 2.598955 | 8.01943 | 8.32E-15 | 1.06E-13 | 22.8168 |
| HLA-DMB | 1.11076 | 3.977446 | 10.923 | 6.17E-25 | 2.78E-23 | 45.7987 |
| ST14 | 1.11271 | 2.179745 | 11.6148 | 1.25E-27 | 8.25E-26 | 51.9256 |
| SKA3 | 1.11531 | 1.790446 | 12.6689 | 6.72E-32 | 8.37E-30 | 61.6505 |
| KIF23 | 1.11583 | 1.357645 | 13.6178 | 6.78E-36 | 1.82E-33 | 70.7561 |
| CFI | 1.11705 | 3.349632 | 9.405 | 2.21E-19 | 4.71E-18 | 33.1819 |
| HAS2 | 1.11722 | 2.453512 | 11.4662 | 4.83E-27 | 2.88E-25 | 50.5915 |
| CCNB1 | 1.11872 | 3.591426 | 13.405 | 5.48E-35 | 1.23E-32 | 68.688 |
| ECM2 | 1.11876 | 3.240608 | 9.60576 | 4.36E-20 | 1.02E-18 | 34.7794 |
| GBP3 | 1.12153 | 3.427299 | 9.27045 | 6.46E-19 | 1.30E-17 | 32.1241 |
| SMC4 | 1.12635 | 2.469226 | 14.5034 | 9.83E-40 | 6.41E-37 | 79.508 |
| HOXC4 | 1.12856 | 1.381108 | 12.1471 | 9.23E-30 | 8.16E-28 | 56.7812 |
| WEE1 | 1.12875 | 2.484842 | 13.6864 | 3.45E-36 | 1.01E-33 | 71.4255 |
| GPNMB | 1.12959 | 3.645654 | 7.72437 | 6.71E-14 | 7.75E-13 | 20.769 |
| TCIM | 1.13988 | 4.065146 | 9.38056 | 2.69E-19 | 5.68E-18 | 32.9889 |
| C1QB | 1.14201 | 8.076688 | 10.4414 | 4.05E-23 | 1.40E-21 | 41.6668 |
| CDC45 | 1.14327 | 1.87498 | 11.9159 | 7.90E-29 | 6.18E-27 | 54.6581 |
| C1R | 1.14541 | 4.910229 | 9.39783 | 2.34E-19 | 4.97E-18 | 33.1252 |
| CDK2 | 1.14692 | 3.447407 | 16.4453 | 1.91E-48 | 1.50E-44 | 99.376 |
| CRISPLD1 | 1.14752 | 5.013715 | 13.1768 | 5.07E-34 | 9.61E-32 | 66.4867 |
| PCLAF | 1.14797 | 1.758283 | 12.4283 | 6.58E-31 | 7.02E-29 | 59.3931 |
| EMILIN3 | 1.14828 | 2.07 | 6.97539 | 1.03E-11 | 9.32E-11 | 15.8435 |
| TACC3 | 1.15224 | 2.96001 | 13.0364 | 1.97E-33 | 3.14E-31 | 65.1408 |
| CA3 | 1.15706 | 1.57169 | 9.45348 | 1.50E-19 | 3.26E-18 | 33.5655 |
| NEK2 | 1.15864 | 1.569797 | 12.7645 | 2.69E-32 | 3.68E-30 | 62.5544 |
| GBP2 | 1.15979 | 3.738614 | 8.91436 | 1.05E-17 | 1.87E-16 | 29.3761 |
| FAM111B | 1.16067 | 1.510245 | 13.407 | 5.37E-35 | 1.21E-32 | 68.7074 |
| CDKN2C | 1.16366 | 4.363295 | 11.007 | 2.94E-25 | 1.36E-23 | 46.5314 |
| CD74 | 1.16654 | 9.048877 | 9.25428 | 7.35E-19 | 1.47E-17 | 31.9976 |
| ALOX5AP | 1.16707 | 4.593455 | 9.68 | 2.38E-20 | 5.74E-19 | 35.376 |
| HLA-DQB1 | 1.16963 | 3.714582 | 8.14107 | 3.46E-15 | 4.58E-14 | 23.6779 |
| TGFB2 | 1.16965 | 3.319504 | 8.47324 | 3.01E-16 | 4.46E-15 | 26.0791 |
| DTL | 1.1734 | 1.994989 | 12.1544 | 8.62E-30 | 7.67E-28 | 56.849 |
| S100A11 | 1.17364 | 6.872417 | 11.2361 | 3.83E-26 | 2.01E-24 | 48.545 |
| TTK | 1.17453 | 1.383982 | 14.1765 | 2.64E-38 | 1.23E-35 | 76.2516 |
| CD163 | 1.1751 | 3.178005 | 7.11882 | 4.04E-12 | 3.84E-11 | 16.7557 |
| GNG12 | 1.17515 | 4.659585 | 10.0263 | 1.36E-21 | 3.86E-20 | 38.1989 |
| MMP14 | 1.17539 | 5.13582 | 11.1367 | 9.31E-26 | 4.62E-24 | 47.668 |
| LMNB1 | 1.1765 | 4.247986 | 13.1585 | 6.05E-34 | 1.11E-31 | 66.3105 |
| S1PR3 | 1.17756 | 3.477774 | 11.586 | 1.63E-27 | 1.05E-25 | 51.6662 |
| IQGAP3 | 1.18166 | 1.61023 | 13.4546 | 3.37E-35 | 7.90E-33 | 69.1687 |
| SCIN | 1.18267 | 2.56451 | 10.3201 | 1.14E-22 | 3.70E-21 | 40.644 |
| RAD51AP1 | 1.18411 | 2.390513 | 15.1789 | 1.01E-42 | 1.73E-39 | 86.3251 |
| LYZ | 1.18427 | 3.059093 | 8.37882 | 6.06E-16 | 8.69E-15 | 25.3893 |
| EMP3 | 1.18703 | 3.60591 | 7.5708 | 1.94E-13 | 2.13E-12 | 19.7268 |
| SPC24 | 1.18832 | 2.235054 | 11.5003 | 3.55E-27 | 2.17E-25 | 50.8969 |
| CENPA | 1.19289 | 1.378783 | 13.9791 | 1.89E-37 | 6.78E-35 | 74.2988 |
| S100A3 | 1.19507 | 2.037544 | 9.45081 | 1.53E-19 | 3.33E-18 | 33.5443 |
| MGP | 1.1961 | 4.239912 | 7.58719 | 1.73E-13 | 1.92E-12 | 19.8372 |
| VCAM1 | 1.19772 | 3.965962 | 8.10068 | 4.64E-15 | 6.06E-14 | 23.391 |
| TNC | 1.20153 | 5.808766 | 9.75649 | 1.27E-20 | 3.18E-19 | 35.9938 |
| RNASE2 | 1.20375 | 2.515737 | 10.0615 | 1.01E-21 | 2.92E-20 | 38.4891 |
| EMP1 | 1.20461 | 4.955281 | 9.56052 | 6.30E-20 | 1.45E-18 | 34.4175 |
| TYMP | 1.20713 | 3.084205 | 10.2936 | 1.43E-22 | 4.58E-21 | 40.4219 |
| CRNDE | 1.20716 | 1.865574 | 10.2485 | 2.10E-22 | 6.56E-21 | 40.044 |
| NUF2 | 1.20736 | 2.072836 | 14.1149 | 4.88E-38 | 2.11E-35 | 75.6406 |
| HLA-DPB1 | 1.21322 | 6.011524 | 9.08365 | 2.82E-18 | 5.28E-17 | 30.6731 |
| ASPM | 1.21622 | 1.135268 | 14.5589 | 5.61E-40 | 4.04E-37 | 80.0641 |
| FLNC | 1.22307 | 3.533048 | 8.82083 | 2.16E-17 | 3.68E-16 | 28.6669 |
| NCAPH | 1.22307 | 2.207485 | 13.2719 | 2.01E-34 | 4.10E-32 | 67.4017 |
| SERPINA1 | 1.22422 | 3.190844 | 10.7826 | 2.11E-24 | 8.73E-23 | 44.5827 |
| CKAP2L | 1.23315 | 1.463633 | 14.0436 | 9.96E-38 | 3.93E-35 | 74.9354 |
| C3 | 1.23589 | 7.019144 | 9.28328 | 5.83E-19 | 1.18E-17 | 32.2244 |
| TYMS | 1.23595 | 3.872871 | 11.816 | 1.98E-28 | 1.47E-26 | 53.7468 |
| GBP1 | 1.23911 | 3.628924 | 10.172 | 4.01E-22 | 1.21E-20 | 39.4057 |
| CEP55 | 1.24019 | 1.43643 | 15.319 | 2.38E-43 | 5.03E-40 | 87.7524 |
| PLAU | 1.24519 | 2.708551 | 10.7761 | 2.24E-24 | 9.18E-23 | 44.5263 |
| SERPINE1 | 1.24688 | 3.683263 | 7.66063 | 1.04E-13 | 1.18E-12 | 20.3345 |
| HOXD9 | 1.2537 | 0.98933 | 10.3715 | 7.37E-23 | 2.45E-21 | 41.0761 |
| MS4A6A | 1.25735 | 3.395942 | 10.6741 | 5.44E-24 | 2.11E-22 | 43.6488 |
| MSR1 | 1.25924 | 2.524604 | 12.6096 | 1.18E-31 | 1.41E-29 | 61.0923 |
| TK1 | 1.2595 | 2.949029 | 12.547 | 2.14E-31 | 2.46E-29 | 60.5042 |
| LTF | 1.26028 | 1.663225 | 6.44396 | 2.86E-10 | 2.19E-09 | 12.5973 |
| TROAP | 1.26365 | 1.622025 | 13.137 | 7.46E-34 | 1.35E-31 | 66.104 |
| BUB1 | 1.26599 | 1.783074 | 14.4727 | 1.34E-39 | 8.26E-37 | 79.2008 |
| HLA-DRB1 | 1.26631 | 7.561012 | 9.14776 | 1.70E-18 | 3.27E-17 | 31.1687 |
| GTSE1 | 1.27942 | 1.879185 | 13.7322 | 2.19E-36 | 6.60E-34 | 71.8733 |
| TIMP1 | 1.28411 | 5.13141 | 7.80796 | 3.73E-14 | 4.43E-13 | 21.3431 |
| PTTG1 | 1.28974 | 3.2565 | 12.671 | 6.58E-32 | 8.22E-30 | 61.6708 |
| HJURP | 1.29026 | 1.719816 | 13.1166 | 9.09E-34 | 1.61E-31 | 65.9082 |
| COL1A1 | 1.29391 | 2.61381 | 8.86959 | 1.49E-17 | 2.59E-16 | 29.036 |
| CCNA2 | 1.31142 | 2.538669 | 14.929 | 1.30E-41 | 1.59E-38 | 83.7898 |
| HLA-DPA1 | 1.31188 | 5.392753 | 9.80759 | 8.36E-21 | 2.15E-19 | 36.4084 |
| BUB1B | 1.31485 | 1.884441 | 13.0099 | 2.55E-33 | 3.98E-31 | 64.8874 |
| IQGAP2 | 1.32662 | 2.226323 | 12.9849 | 3.25E-33 | 4.98E-31 | 64.6487 |
| HLA-DOA | 1.32763 | 3.467159 | 10.6065 | 9.78E-24 | 3.66E-22 | 43.0706 |
| NCAPG | 1.33197 | 1.662871 | 14.1498 | 3.44E-38 | 1.55E-35 | 75.9862 |
| HLA-DRB5 | 1.33407 | 5.979075 | 8.95925 | 7.44E-18 | 1.34E-16 | 29.7182 |
| CXCL10 | 1.34807 | 1.8355 | 9.16195 | 1.52E-18 | 2.94E-17 | 31.2787 |
| HLA-DQA1 | 1.34936 | 2.838389 | 9.5538 | 6.65E-20 | 1.52E-18 | 34.3638 |
| DLGAP5 | 1.35018 | 1.366562 | 14.8125 | 4.27E-41 | 4.42E-38 | 82.6137 |
| APOL4 | 1.35627 | 2.255877 | 10.6424 | 7.17E-24 | 2.73E-22 | 43.3774 |
| CDK1 | 1.3747 | 2.588331 | 13.133 | 7.75E-34 | 1.39E-31 | 66.0663 |
| CENPU | 1.37823 | 2.408627 | 13.9454 | 2.65E-37 | 9.13E-35 | 73.9669 |
| SPP1 | 1.37937 | 8.954714 | 9.52352 | 8.50E-20 | 1.91E-18 | 34.1222 |
| KIF4A | 1.38191 | 2.167521 | 14.4687 | 1.40E-39 | 8.50E-37 | 79.1606 |
| PLEKHA4 | 1.38425 | 4.240709 | 11.7347 | 4.18E-28 | 2.95E-26 | 53.0094 |
| VIM | 1.38527 | 8.379418 | 10.3659 | 7.73E-23 | 2.56E-21 | 41.0293 |
| NDC80 | 1.40426 | 1.848322 | 14.9068 | 1.63E-41 | 1.90E-38 | 83.5659 |
| SPOCD1 | 1.41214 | 2.027913 | 9.69983 | 2.03E-20 | 4.92E-19 | 35.5359 |
| HLA-DRA | 1.4151 | 8.54253 | 10.0144 | 1.50E-21 | 4.25E-20 | 38.1004 |
| MELK | 1.41725 | 1.839841 | 14.216 | 1.77E-38 | 8.68E-36 | 76.6436 |
| COL4A2 | 1.42367 | 4.475923 | 11.2904 | 2.36E-26 | 1.27E-24 | 49.0252 |
| KIF20A | 1.43314 | 1.676325 | 14.9227 | 1.39E-41 | 1.65E-38 | 83.7265 |
| MKI67 | 1.43317 | 2.284385 | 12.6787 | 6.12E-32 | 7.73E-30 | 61.7431 |
| CDCA8 | 1.43503 | 2.287186 | 15.3416 | 1.89E-43 | 4.14E-40 | 87.9833 |
| COL3A1 | 1.43512 | 2.895843 | 9.64473 | 3.18E-20 | 7.54E-19 | 35.0922 |
| IGFBP2 | 1.43607 | 3.750105 | 9.1655 | 1.48E-18 | 2.86E-17 | 31.3063 |
| KIFC1 | 1.44217 | 2.842828 | 12.6312 | 9.61E-32 | 1.16E-29 | 61.296 |
| TNFRSF12A | 1.4436 | 3.715604 | 10.6522 | 6.59E-24 | 2.54E-22 | 43.4609 |
| ASF1B | 1.44372 | 2.498543 | 14.1246 | 4.43E-38 | 1.96E-35 | 75.737 |
| AURKB | 1.47868 | 2.134077 | 13.1863 | 4.62E-34 | 8.92E-32 | 66.5773 |
| KIF2C | 1.47882 | 2.335134 | 14.9803 | 7.71E-42 | 1.08E-38 | 84.3093 |
| FCGR3A | 1.48806 | 5.167167 | 11.5827 | 1.68E-27 | 1.08E-25 | 51.6368 |
| METTL7B | 1.48898 | 4.005341 | 9.09667 | 2.55E-18 | 4.80E-17 | 30.7735 |
| CCNB2 | 1.51162 | 2.277399 | 14.6248 | 2.88E-40 | 2.39E-37 | 80.725 |
| FOXM1 | 1.54474 | 3.068525 | 13.3252 | 1.20E-34 | 2.58E-32 | 67.9163 |
| NAPSB | 1.55897 | 3.712827 | 10.6495 | 6.74E-24 | 2.59E-22 | 43.4384 |
| CENPF | 1.56487 | 2.407997 | 14.522 | 8.15E-40 | 5.51E-37 | 79.6941 |
| PBK | 1.59506 | 2.85034 | 12.4716 | 4.37E-31 | 4.75E-29 | 59.7979 |
| TPX2 | 1.59679 | 3.632731 | 14.252 | 1.24E-38 | 6.39E-36 | 77.0005 |
| ANXA1 | 1.6023 | 4.41343 | 10.1964 | 3.26E-22 | 1.00E-20 | 39.609 |
| PIMREG | 1.60383 | 2.583662 | 13.2868 | 1.74E-34 | 3.57E-32 | 67.5453 |
| RRM2 | 1.61676 | 2.367335 | 13.6549 | 4.71E-36 | 1.32E-33 | 71.1178 |
| BIRC5 | 1.64319 | 2.716624 | 13.1653 | 5.67E-34 | 1.06E-31 | 66.3758 |
| CDC20 | 1.6565 | 2.845754 | 14.8156 | 4.14E-41 | 4.36E-38 | 82.6445 |
| FCGBP | 1.6583 | 3.633422 | 10.8677 | 1.00E-24 | 4.37E-23 | 45.3187 |
| COL4A1 | 1.66466 | 4.092979 | 12.1153 | 1.24E-29 | 1.07E-27 | 56.4877 |
| MYBL2 | 1.6804 | 2.573085 | 12.5694 | 1.73E-31 | 2.02E-29 | 60.714 |
| NUSAP1 | 1.68221 | 3.640789 | 14.1196 | 4.66E-38 | 2.04E-35 | 75.6874 |
| PDPN | 1.70833 | 3.571739 | 10.5009 | 2.43E-23 | 8.60E-22 | 42.1705 |
| CHI3L2 | 1.80548 | 3.834745 | 10.7566 | 2.65E-24 | 1.08E-22 | 44.3584 |
| CHI3L1 | 1.80866 | 4.577935 | 7.03167 | 7.13E-12 | 6.61E-11 | 16.1996 |
| UBE2C | 1.84225 | 3.433359 | 13.4429 | 3.78E-35 | 8.74E-33 | 69.0556 |
| TOP2A | 1.98065 | 3.433001 | 14.0451 | 9.80E-38 | 3.92E-35 | 74.9508 |
