# Supplementary material for: MGME1 associates with poor prognosis and is vital for cell proliferation in lower-grade glioma
Source: Aging (Albany NY). 2023 May 8;15(9):3690–714. doi: 10.18632/aging.204705 (PMC10449294; doi:10.18632/aging.204705)
Supplement: Supplementary Table 3 [file aging-15-204705-s004.docx]

**Supplementary Table 3. Down-regulated DEGs in CGGA dataset.**

| **id** | **logFC** | **AveExpr** | **t** | **P.Value** | **adj.P.Val** | **B** |
| --- | --- | --- | --- | --- | --- | --- |
| SLC17A7 | -2.1655 | 4.455761 | -5.9956 | 1.17E-08 | 1.42E-07 | 9.40562 |
| CAMK2A | -1.9348 | 4.250693 | -5.9898 | 1.21E-08 | 1.45E-07 | 9.37711 |
| VSNL1 | -1.9025 | 4.646095 | -5.2861 | 3.78E-07 | 2.80E-06 | 6.06568 |
| PACSIN1 | -1.8964 | 3.462276 | -6.2311 | 3.49E-09 | 5.01E-08 | 10.5751 |
| NRGN | -1.8768 | 6.065147 | -5.4656 | 1.61E-07 | 1.35E-06 | 6.88314 |
| GRIN1 | -1.8575 | 4.041773 | -6.3623 | 1.76E-09 | 2.75E-08 | 11.2388 |
| CPLX2 | -1.8397 | 5.079459 | -6.3546 | 1.83E-09 | 2.84E-08 | 11.1995 |
| SNCB | -1.8348 | 5.084756 | -6.632 | 4.18E-10 | 8.10E-09 | 12.6294 |
| SNAP25 | -1.7732 | 6.832573 | -5.8635 | 2.29E-08 | 2.51E-07 | 8.76244 |
| CABP1 | -1.7429 | 3.820338 | -7.2528 | 1.36E-11 | 4.43E-10 | 15.9537 |
| NEFM | -1.7022 | 3.66429 | -5.4129 | 2.07E-07 | 1.68E-06 | 6.641 |
| SYN2 | -1.6954 | 3.699184 | -5.9459 | 1.51E-08 | 1.76E-07 | 9.16243 |
| NEFL | -1.6867 | 3.536503 | -4.9494 | 1.77E-06 | 1.08E-05 | 4.58513 |
| SVOP | -1.6822 | 3.450028 | -6.3213 | 2.18E-09 | 3.33E-08 | 11.0305 |
| DDN | -1.6718 | 3.288028 | -5.9917 | 1.20E-08 | 1.44E-07 | 9.38637 |
| CHGA | -1.6624 | 4.531999 | -6.0769 | 7.75E-09 | 9.91E-08 | 9.80594 |
| SYT1 | -1.659 | 4.405864 | -5.1884 | 5.96E-07 | 4.14E-06 | 5.62898 |
| GABRD | -1.6563 | 3.658146 | -8.2259 | 4.72E-14 | 3.49E-12 | 21.4616 |
| CCK | -1.6503 | 4.051708 | -5.0041 | 1.39E-06 | 8.67E-06 | 4.82099 |
| ETNPPL | -1.6241 | 5.699047 | -5.2816 | 3.86E-07 | 2.85E-06 | 6.04529 |
| SULT4A1 | -1.6143 | 3.362996 | -5.6419 | 6.86E-08 | 6.42E-07 | 7.70457 |
| TMEM130 | -1.613 | 3.923067 | -5.8769 | 2.14E-08 | 2.37E-07 | 8.82737 |
| RBFOX3 | -1.607 | 2.813533 | -6.9579 | 7.06E-11 | 1.79E-09 | 14.3542 |
| GJB6 | -1.6048 | 2.146628 | -6.9332 | 8.09E-11 | 2.01E-09 | 14.2217 |
| SYN1 | -1.5985 | 4.749872 | -7.0283 | 4.78E-11 | 1.30E-09 | 14.7327 |
| PHYHIP | -1.5613 | 4.38193 | -6.7202 | 2.59E-10 | 5.36E-09 | 13.0916 |
| RP5-1119A7.17 | -1.5537 | 2.901983 | -7.1768 | 2.08E-11 | 6.41E-10 | 15.5381 |
| HPCAL4 | -1.55 | 4.209952 | -6.5001 | 8.47E-10 | 1.48E-08 | 11.9453 |
| GABRG2 | -1.5399 | 3.167448 | -5.8659 | 2.26E-08 | 2.48E-07 | 8.77407 |
| KCNIP2 | -1.5282 | 4.920182 | -6.3309 | 2.07E-09 | 3.19E-08 | 11.0793 |
| GNG3 | -1.5265 | 4.452132 | -4.8608 | 2.63E-06 | 1.53E-05 | 4.20772 |
| SFRP2 | -1.5069 | 4.982293 | -4.1193 | 5.91E-05 | 0.000235 | 1.25755 |
| NTSR2 | -1.4992 | 3.910072 | -5.6435 | 6.80E-08 | 6.38E-07 | 7.71214 |
| PRLHR | -1.4736 | 2.169208 | -6.1471 | 5.40E-09 | 7.26E-08 | 10.1547 |
| CPLX1 | -1.4571 | 4.756808 | -6.9828 | 6.15E-11 | 1.61E-09 | 14.488 |
| GABRA1 | -1.4557 | 2.636628 | -5.2733 | 4.01E-07 | 2.94E-06 | 6.00824 |
| CELF4 | -1.4441 | 3.848054 | -6.0474 | 9.01E-09 | 1.13E-07 | 9.66043 |
| STMN2 | -1.4436 | 5.333898 | -4.3008 | 2.86E-05 | 0.000124 | 1.94397 |
| CALY | -1.4383 | 3.789204 | -5.0869 | 9.51E-07 | 6.24E-06 | 5.18137 |
| NPTX1 | -1.4306 | 3.34858 | -5.5592 | 1.03E-07 | 9.07E-07 | 7.31707 |
| MYT1L | -1.4173 | 3.047237 | -6.1101 | 6.53E-09 | 8.54E-08 | 9.97083 |
| IQSEC3 | -1.4111 | 2.862402 | -7.0635 | 3.93E-11 | 1.10E-09 | 14.9226 |
| TMEM151B | -1.3859 | 3.874583 | -7.1468 | 2.47E-11 | 7.36E-10 | 15.3747 |
| SCRT1 | -1.3689 | 3.044112 | -6.3606 | 1.77E-09 | 2.77E-08 | 11.23 |
| RPH3A | -1.3571 | 3.871721 | -5.4448 | 1.78E-07 | 1.47E-06 | 6.7875 |
| SLC6A17 | -1.3556 | 2.530222 | -5.7793 | 3.48E-08 | 3.58E-07 | 8.35733 |
| SYT4 | -1.3527 | 3.214615 | -5.7257 | 4.54E-08 | 4.48E-07 | 8.10171 |
| HRH3 | -1.3525 | 2.150226 | -7.2863 | 1.12E-11 | 3.76E-10 | 16.1375 |
| INA | -1.3484 | 4.516722 | -4.925 | 1.98E-06 | 1.18E-05 | 4.48072 |
| SV2B | -1.3423 | 2.512257 | -5.6216 | 7.57E-08 | 6.99E-07 | 7.60944 |
| FAM163B | -1.3357 | 3.421795 | -5.1403 | 7.44E-07 | 5.04E-06 | 5.41588 |
| SYT13 | -1.3335 | 2.657985 | -5.4106 | 2.10E-07 | 1.69E-06 | 6.63078 |
| PVALB | -1.3333 | 2.086931 | -5.3931 | 2.28E-07 | 1.82E-06 | 6.55085 |
| NSG2 | -1.3297 | 6.079631 | -4.8277 | 3.05E-06 | 1.74E-05 | 4.06817 |
| KIAA1045 | -1.3233 | 2.602281 | -5.6737 | 5.86E-08 | 5.60E-07 | 7.85494 |
| PPP2R2C | -1.3224 | 4.663759 | -5.776 | 3.54E-08 | 3.63E-07 | 8.34149 |
| SYT5 | -1.3159 | 3.611046 | -5.8216 | 2.82E-08 | 2.99E-07 | 8.56058 |
| GABRA5 | -1.3139 | 2.450676 | -5.3605 | 2.66E-07 | 2.06E-06 | 6.40235 |
| SCN3B | -1.3132 | 4.301502 | -6.1759 | 4.65E-09 | 6.39E-08 | 10.2984 |
| HPCA | -1.3098 | 4.159223 | -4.471 | 1.41E-05 | 6.67E-05 | 2.60892 |
| L1CAM | -1.3023 | 4.163544 | -5.1775 | 6.26E-07 | 4.34E-06 | 5.58048 |
| VSTM2A | -1.2968 | 3.859622 | -5.0491 | 1.13E-06 | 7.26E-06 | 5.01626 |
| CKMT1A | -1.2965 | 2.987888 | -5.9564 | 1.43E-08 | 1.68E-07 | 9.21386 |
| RBFOX1 | -1.2964 | 2.917698 | -5.2444 | 4.59E-07 | 3.31E-06 | 5.87863 |
| CRYM | -1.2958 | 3.161502 | -4.2655 | 3.30E-05 | 0.000141 | 1.80846 |
| CREG2 | -1.2892 | 2.524261 | -5.1394 | 7.47E-07 | 5.06E-06 | 5.41179 |
| SYT7 | -1.2884 | 3.221298 | -5.5821 | 9.18E-08 | 8.23E-07 | 7.42393 |
| SHISA6 | -1.2865 | 2.63262 | -5.3984 | 2.22E-07 | 1.78E-06 | 6.57505 |
| CDK5R2 | -1.2821 | 3.201755 | -6.4312 | 1.22E-09 | 2.02E-08 | 11.5908 |
| SYNPR | -1.2721 | 2.692825 | -4.7621 | 4.07E-06 | 2.24E-05 | 3.7932 |
| RGS4 | -1.2715 | 3.895463 | -4.4573 | 1.50E-05 | 7.02E-05 | 2.55481 |
| CHGB | -1.2642 | 5.306336 | -5.0179 | 1.30E-06 | 8.22E-06 | 4.88066 |
| SHANK1 | -1.2606 | 3.420274 | -6.7075 | 2.78E-10 | 5.69E-09 | 13.0249 |
| PRKCG | -1.2571 | 2.145461 | -5.5116 | 1.29E-07 | 1.11E-06 | 7.09569 |
| CACNG2 | -1.2539 | 2.306814 | -6.2772 | 2.75E-09 | 4.07E-08 | 10.8075 |
| TMEM155 | -1.2417 | 1.890307 | -5.7038 | 5.06E-08 | 4.92E-07 | 7.99756 |
| MAL2 | -1.2372 | 1.937734 | -5.4261 | 1.95E-07 | 1.59E-06 | 6.70168 |
| SNCG | -1.2368 | 5.056028 | -5.0008 | 1.41E-06 | 8.78E-06 | 4.80673 |
| CLEC2L | -1.2323 | 2.053615 | -6.0373 | 9.48E-09 | 1.18E-07 | 9.61073 |
| MPPED1 | -1.221 | 2.298881 | -5.4759 | 1.53E-07 | 1.29E-06 | 6.93087 |
| AC112229.7 | -1.2203 | 1.899446 | -5.4158 | 2.04E-07 | 1.66E-06 | 6.65464 |
| ARHGDIG | -1.2203 | 4.591288 | -6.0542 | 8.70E-09 | 1.09E-07 | 9.69381 |
| SST | -1.2178 | 3.730963 | -4.3564 | 2.27E-05 | 0.000102 | 2.15898 |
| OPALIN | -1.2133 | 3.072768 | -3.8107 | 0.00019 | 0.00067 | 0.14647 |
| RAB3A | -1.2113 | 4.957154 | -5.6743 | 5.85E-08 | 5.59E-07 | 7.85771 |
| NRSN1 | -1.2102 | 4.668718 | -6.1738 | 4.70E-09 | 6.45E-08 | 10.2882 |
| SYNGR3 | -1.2062 | 3.306706 | -5.4819 | 1.49E-07 | 1.26E-06 | 6.95857 |
| SLC8A2 | -1.2054 | 3.042215 | -6.2219 | 3.66E-09 | 5.22E-08 | 10.529 |
| JPH3 | -1.2045 | 4.292849 | -5.826 | 2.76E-08 | 2.94E-07 | 8.58155 |
| CHD5 | -1.2044 | 2.473199 | -5.7244 | 4.57E-08 | 4.50E-07 | 8.09521 |
| CKMT1B | -1.1996 | 3.479556 | -5.5105 | 1.30E-07 | 1.11E-06 | 7.09069 |
| FXYD7 | -1.1955 | 4.555761 | -4.5504 | 1.01E-05 | 4.96E-05 | 2.92647 |
| GABRB2 | -1.1941 | 2.230171 | -5.0705 | 1.02E-06 | 6.67E-06 | 5.10948 |
| VSTM2L | -1.192 | 3.551544 | -5.3951 | 2.26E-07 | 1.80E-06 | 6.55989 |
| SLC1A2 | -1.1892 | 6.52622 | -6.5199 | 7.63E-10 | 1.35E-08 | 12.0474 |
| MTND1P23 | -1.1882 | 2.637899 | -4.7573 | 4.16E-06 | 2.28E-05 | 3.77313 |
| UNC13C | -1.188 | 1.697964 | -6.6474 | 3.85E-10 | 7.55E-09 | 12.7097 |
| BRINP1 | -1.1842 | 4.209195 | -6.1654 | 4.91E-09 | 6.70E-08 | 10.246 |
| C1QTNF4 | -1.1808 | 3.503335 | -6.4165 | 1.32E-09 | 2.16E-08 | 11.5153 |
| PTPRN | -1.1799 | 4.719074 | -5.0332 | 1.21E-06 | 7.73E-06 | 4.94724 |
| CACNG3 | -1.1763 | 1.825826 | -5.2182 | 5.19E-07 | 3.68E-06 | 5.7615 |
| KCNC2 | -1.1738 | 2.198105 | -5.1579 | 6.86E-07 | 4.69E-06 | 5.49367 |
| RFPL1S | -1.1713 | 2.473881 | -6.2492 | 3.18E-09 | 4.63E-08 | 10.6663 |
| GDA | -1.1628 | 2.461325 | -4.6933 | 5.49E-06 | 2.91E-05 | 3.5082 |
| SLC30A3 | -1.1625 | 1.899984 | -5.165 | 6.64E-07 | 4.56E-06 | 5.52516 |
| CBLN2 | -1.1614 | 2.272038 | -5.6153 | 7.81E-08 | 7.16E-07 | 7.57961 |
| ACBD7 | -1.1556 | 3.610405 | -5.2035 | 5.55E-07 | 3.90E-06 | 5.69598 |
| TBR1 | -1.1551 | 1.788126 | -5.7279 | 4.49E-08 | 4.44E-07 | 8.11194 |
| CHRM1 | -1.1461 | 2.448056 | -5.5913 | 8.78E-08 | 7.93E-07 | 7.46695 |
| EPHB6 | -1.1439 | 3.278163 | -5.3038 | 3.48E-07 | 2.61E-06 | 6.14526 |
| CAMK2B | -1.1412 | 4.504531 | -5.2687 | 4.10E-07 | 3.00E-06 | 5.98757 |
| FBXL16 | -1.1405 | 5.597293 | -5.9677 | 1.35E-08 | 1.60E-07 | 9.26892 |
| CPNE5 | -1.1403 | 4.320395 | -6.8178 | 1.52E-10 | 3.46E-09 | 13.6071 |
| EEF1A2 | -1.137 | 5.849873 | -4.8202 | 3.15E-06 | 1.79E-05 | 4.03654 |
| LY6H | -1.1332 | 5.141616 | -4.9083 | 2.13E-06 | 1.27E-05 | 4.40963 |
| KIAA1644 | -1.1323 | 2.560369 | -5.6547 | 6.44E-08 | 6.07E-07 | 7.76528 |
| NEFH | -1.1288 | 2.532883 | -5.2785 | 3.91E-07 | 2.88E-06 | 6.03135 |
| SLC12A5 | -1.1284 | 4.193866 | -6.2056 | 3.99E-09 | 5.61E-08 | 10.4472 |
| SLC26A4-AS1 | -1.1284 | 1.82397 | -5.0823 | 9.71E-07 | 6.36E-06 | 5.16105 |
| CAMK1G | -1.1248 | 1.993119 | -5.2864 | 3.77E-07 | 2.80E-06 | 6.06706 |
| KCNJ4 | -1.1247 | 2.570707 | -4.9311 | 1.92E-06 | 1.16E-05 | 4.50706 |
| RASL10A | -1.1239 | 4.887235 | -5.0154 | 1.32E-06 | 8.30E-06 | 4.86994 |
| DMTN | -1.1202 | 4.944699 | -5.2282 | 4.95E-07 | 3.53E-06 | 5.80598 |
| EMX2OS | -1.1183 | 3.008292 | -6.0201 | 1.04E-08 | 1.27E-07 | 9.52613 |
| NEUROD2 | -1.1157 | 1.888881 | -5.3196 | 3.23E-07 | 2.45E-06 | 6.21686 |
| ACTL6B | -1.1122 | 3.938454 | -4.804 | 3.38E-06 | 1.90E-05 | 3.96868 |
| KCNH3 | -1.1104 | 2.782304 | -5.5042 | 1.34E-07 | 1.15E-06 | 7.06149 |
| ST8SIA3 | -1.1089 | 3.011894 | -5.3824 | 2.40E-07 | 1.89E-06 | 6.50181 |
| NELL1 | -1.1084 | 1.978389 | -6.1863 | 4.41E-09 | 6.10E-08 | 10.3507 |
| MAP7D2 | -1.103 | 2.162864 | -4.702 | 5.28E-06 | 2.82E-05 | 3.54417 |
| OLFM1 | -1.1023 | 6.868118 | -5.3512 | 2.78E-07 | 2.15E-06 | 6.36008 |
| NEURL | -1.1008 | 2.603706 | -5.6193 | 7.65E-08 | 7.05E-07 | 7.59856 |
| TUBA4A | -1.0985 | 4.848712 | -5.2444 | 4.59E-07 | 3.31E-06 | 5.87846 |
| ABCC8 | -1.0935 | 4.420348 | -4.8049 | 3.37E-06 | 1.90E-05 | 3.97225 |
| KCNJ11 | -1.0919 | 3.323206 | -5.4349 | 1.87E-07 | 1.54E-06 | 6.74217 |
| RASGRF1 | -1.0902 | 3.428987 | -5.5049 | 1.33E-07 | 1.14E-06 | 7.06474 |
| RP11-47I22.1 | -1.0857 | 2.178892 | -4.4535 | 1.52E-05 | 7.12E-05 | 2.53984 |
| DOC2A | -1.0855 | 3.286954 | -5.0429 | 1.16E-06 | 7.45E-06 | 4.98939 |
| RBP4 | -1.0797 | 2.133748 | -5.1498 | 7.12E-07 | 4.85E-06 | 5.45782 |
| TNNT1 | -1.0791 | 2.530154 | -5.3011 | 3.52E-07 | 2.64E-06 | 6.13324 |
| SNAP91 | -1.0742 | 4.812966 | -5.3803 | 2.42E-07 | 1.91E-06 | 6.4923 |
| CPNE6 | -1.0738 | 3.302634 | -4.3526 | 2.31E-05 | 0.000103 | 2.14412 |
| TESPA1 | -1.0717 | 1.770063 | -4.6402 | 6.90E-06 | 3.56E-05 | 3.2903 |
| CALN1 | -1.0712 | 3.050463 | -5.0348 | 1.21E-06 | 7.68E-06 | 4.95408 |
| PTPN5 | -1.0686 | 2.848651 | -4.5028 | 1.24E-05 | 5.92E-05 | 2.7354 |
| RP11-286B14.1 | -1.0669 | 1.248453 | -5.3949 | 2.26E-07 | 1.80E-06 | 6.55883 |
| RTN1 | -1.0646 | 7.730623 | -5.5777 | 9.37E-08 | 8.39E-07 | 7.40369 |
| NPY | -1.0618 | 4.584362 | -3.7034 | 0.00029 | 0.000952 | -0.223 |
| CRLF1 | -1.055 | 4.433426 | -3.8709 | 0.00015 | 0.000548 | 0.35769 |
| HS3ST2 | -1.0541 | 2.30223 | -5.6225 | 7.54E-08 | 6.96E-07 | 7.61356 |
| C1QL3 | -1.0539 | 1.463367 | -5.078 | 9.90E-07 | 6.47E-06 | 5.14243 |
| PSD | -1.0519 | 5.562866 | -5.5731 | 9.58E-08 | 8.56E-07 | 7.3822 |
| RTN4R | -1.0512 | 3.052352 | -6.1447 | 5.47E-09 | 7.33E-08 | 10.1427 |
| RGS7 | -1.051 | 2.898169 | -5.9127 | 1.78E-08 | 2.03E-07 | 9.00077 |
| ATP1A3 | -1.0509 | 6.133991 | -4.9371 | 1.87E-06 | 1.13E-05 | 4.53279 |
| AIFM3 | -1.0464 | 3.288755 | -4.7941 | 3.54E-06 | 1.98E-05 | 3.92678 |
| ITPKA | -1.0402 | 2.802973 | -5.7398 | 4.23E-08 | 4.23E-07 | 8.16861 |
| ATP2B3 | -1.0341 | 1.868457 | -6.0699 | 8.03E-09 | 1.02E-07 | 9.77149 |
| SH3GL2 | -1.0329 | 5.284383 | -4.704 | 5.24E-06 | 2.81E-05 | 3.55222 |
| SH2D5 | -1.0328 | 1.816285 | -5.3412 | 2.91E-07 | 2.24E-06 | 6.31452 |
| GABRG1 | -1.0316 | 2.706246 | -4.9548 | 1.73E-06 | 1.05E-05 | 4.60863 |
| NAPB | -1.0311 | 4.756962 | -5.2855 | 3.79E-07 | 2.81E-06 | 6.063 |
| DLGAP3 | -1.028 | 2.925447 | -5.6736 | 5.87E-08 | 5.60E-07 | 7.85462 |
| MAL | -1.0277 | 4.652252 | -3.3065 | 0.00115 | 0.003212 | -1.5098 |
| WSCD2 | -1.0259 | 1.958589 | -6.0358 | 9.56E-09 | 1.18E-07 | 9.6034 |
| CAMKV | -1.0244 | 3.880596 | -4.2074 | 4.16E-05 | 0.000172 | 1.58761 |
| F5 | -1.0226 | 2.335026 | -3.5637 | 0.00047 | 0.001485 | -0.6902 |
| ICAM5 | -1.0221 | 2.545606 | -4.8419 | 2.86E-06 | 1.64E-05 | 4.12801 |
| NGEF | -1.0212 | 4.099454 | -4.3415 | 2.42E-05 | 0.000107 | 2.1011 |
| UNC5A | -1.0199 | 3.321224 | -5.7301 | 4.44E-08 | 4.40E-07 | 8.1223 |
| CA11 | -1.0187 | 5.829693 | -6.2006 | 4.09E-09 | 5.73E-08 | 10.4223 |
| SLC25A48 | -1.0168 | 4.524534 | -4.1402 | 5.44E-05 | 0.000219 | 1.33535 |
| RP11-862L9.3 | -1.0162 | 3.842493 | -2.8238 | 0.00531 | 0.012248 | -2.9009 |
| AK5 | -1.0146 | 4.183829 | -3.9912 | 9.74E-05 | 0.000365 | 0.78753 |
| SLC6A7 | -1.0136 | 1.233328 | -5.5042 | 1.34E-07 | 1.15E-06 | 7.06162 |
| MAPK8IP2 | -1.0123 | 5.314444 | -7.053 | 4.16E-11 | 1.15E-09 | 14.8661 |
| PTPRT | -1.0114 | 2.747056 | -5.2762 | 3.96E-07 | 2.91E-06 | 6.02109 |
| PTGDS | -1.0055 | 9.478504 | -4.3011 | 2.85E-05 | 0.000124 | 1.94512 |
| SNCA | -1.0034 | 5.334613 | -4.598 | 8.27E-06 | 4.17E-05 | 3.11849 |
| ALDOC | -1.0019 | 9.163057 | -5.2311 | 4.88E-07 | 3.49E-06 | 5.81893 |
| SEZ6L2 | -1.0012 | 4.958679 | -5.1312 | 7.76E-07 | 5.22E-06 | 5.37599 |
| ACSL6 | -1.0004 | 4.806652 | -5.6889 | 5.44E-08 | 5.26E-07 | 7.92669 |
| KCNN1 | -0.9996 | 3.157784 | -6.3525 | 1.85E-09 | 2.87E-08 | 11.1889 |
| AC013268.5 | -0.9994 | 1.354051 | -5.257 | 4.33E-07 | 3.15E-06 | 5.93496 |
| MATK | -0.9987 | 2.605586 | -6.1907 | 4.31E-09 | 5.99E-08 | 10.3725 |
| C1orf115 | -0.9957 | 3.077725 | -5.0482 | 1.13E-06 | 7.29E-06 | 5.01219 |
| SCN2B | -0.9945 | 2.852433 | -5.9077 | 1.83E-08 | 2.08E-07 | 8.97655 |
| RP11-192H23.5 | -0.9945 | 3.063614 | -3.9416 | 0.00012 | 0.000432 | 0.60915 |
| SLC25A18 | -0.9943 | 5.673509 | -6.4417 | 1.16E-09 | 1.93E-08 | 11.6445 |
| SYP | -0.9934 | 6.355822 | -5.7089 | 4.93E-08 | 4.81E-07 | 8.02147 |
| UBE2QL1 | -0.9933 | 2.915069 | -5.8476 | 2.48E-08 | 2.68E-07 | 8.68547 |
| WIF1 | -0.992 | 1.520013 | -4.0821 | 6.84E-05 | 0.000267 | 1.11973 |
| MBP | -0.99 | 9.78837 | -2.4054 | 0.01722 | 0.033847 | -3.9443 |
| DNM1 | -0.9887 | 5.298269 | -4.4403 | 1.61E-05 | 7.46E-05 | 2.48765 |
| EMX1 | -0.988 | 1.359463 | -5.6412 | 6.88E-08 | 6.44E-07 | 7.70158 |
| NPTXR | -0.9861 | 5.026371 | -5.944 | 1.52E-08 | 1.78E-07 | 9.15336 |
| KIF5A | -0.9842 | 6.354168 | -4.9094 | 2.12E-06 | 1.26E-05 | 4.41437 |
| LINC00087 | -0.9834 | 2.521558 | -6.3055 | 2.37E-09 | 3.57E-08 | 10.9502 |
| PRKCB | -0.9822 | 3.975006 | -5.2437 | 4.60E-07 | 3.32E-06 | 5.87553 |
| RUNDC3A | -0.9816 | 6.440221 | -5.5416 | 1.12E-07 | 9.78E-07 | 7.23531 |
| TCEAL6 | -0.9811 | 3.042301 | -4.1185 | 5.93E-05 | 0.000236 | 1.25439 |
| CSDC2 | -0.9774 | 4.37838 | -4.5761 | 9.08E-06 | 4.52E-05 | 3.0299 |
| DGCR6 | -0.9772 | 5.839821 | -5.055 | 1.10E-06 | 7.09E-06 | 5.04201 |
| RAP1GAP2 | -0.9732 | 2.914664 | -6.6553 | 3.69E-10 | 7.28E-09 | 12.751 |
| TTC9B | -0.9699 | 4.029301 | -4.5851 | 8.73E-06 | 4.36E-05 | 3.06644 |
| RAB3C | -0.9689 | 3.040631 | -4.6751 | 5.94E-06 | 3.12E-05 | 3.4332 |
| DLGAP1-AS4 | -0.9674 | 1.022159 | -6.3801 | 1.60E-09 | 2.54E-08 | 11.3295 |
| GRIN2C | -0.9642 | 2.947278 | -6.7753 | 1.92E-10 | 4.19E-09 | 13.3822 |
| SELL | -0.9614 | 4.733082 | -3.08 | 0.00241 | 0.006167 | -2.187 |
| KCNT1 | -0.9601 | 1.896248 | -5.3327 | 3.03E-07 | 2.32E-06 | 6.27604 |
| RP11-143K11.1 | -0.959 | 2.847811 | -5.1818 | 6.14E-07 | 4.27E-06 | 5.59929 |
| FXYD1 | -0.9572 | 4.895684 | -3.5121 | 0.00057 | 0.001735 | -0.859 |
| NGB | -0.9542 | 1.104482 | -5.2622 | 4.22E-07 | 3.08E-06 | 5.95817 |
| SERPINI1 | -0.9515 | 5.111087 | -3.9386 | 0.00012 | 0.000436 | 0.59827 |
| SRRM3 | -0.9492 | 3.743207 | -5.0082 | 1.36E-06 | 8.54E-06 | 4.8385 |
| C1orf95 | -0.9487 | 3.567842 | -6.1037 | 6.75E-09 | 8.79E-08 | 9.9391 |
| JAKMIP1 | -0.9487 | 2.970803 | -5.0635 | 1.06E-06 | 6.86E-06 | 5.07899 |
| GNAL | -0.9482 | 3.39352 | -5.9161 | 1.75E-08 | 2.00E-07 | 9.01732 |
| CTD-2396E7.9 | -0.9471 | 3.430318 | -4.6615 | 6.30E-06 | 3.28E-05 | 3.37755 |
| SLIT1 | -0.9459 | 4.57104 | -4.0743 | 7.05E-05 | 0.000274 | 1.09109 |
| SLC24A4 | -0.9446 | 2.411354 | -5.6868 | 5.50E-08 | 5.30E-07 | 7.91665 |
| RP11-227B21.2 | -0.9435 | 1.633883 | -5.5305 | 1.18E-07 | 1.02E-06 | 7.1837 |
| CDH22 | -0.9434 | 2.491492 | -5.6117 | 7.94E-08 | 7.27E-07 | 7.5629 |
| CTD-2562J17.7 | -0.943 | 3.03736 | -6.1922 | 4.28E-09 | 5.95E-08 | 10.3801 |
| PPP4R4 | -0.941 | 2.116835 | -5.6195 | 7.65E-08 | 7.04E-07 | 7.59935 |
| HAR1A | -0.9406 | 1.332086 | -7.9958 | 1.85E-13 | 1.11E-11 | 20.1306 |
| CRTAC1 | -0.9376 | 4.633322 | -4.2964 | 2.91E-05 | 0.000126 | 1.92714 |
| PCP4L1 | -0.9367 | 2.445827 | -3.9578 | 0.00011 | 0.000409 | 0.66703 |
| KNDC1 | -0.9348 | 3.308179 | -6.2024 | 4.06E-09 | 5.68E-08 | 10.4312 |
| RP11-1263C18.1 | -0.933 | 1.736532 | -5.5892 | 8.86E-08 | 7.99E-07 | 7.45753 |
| WNT7B | -0.9321 | 2.68421 | -4.4048 | 1.86E-05 | 8.49E-05 | 2.34781 |
| MT-ND1 | -0.9319 | 13.24071 | -10.103 | 4.13E-19 | 6.24E-16 | 32.8193 |
| MIR7-3HG | -0.9316 | 2.109871 | -4.8972 | 2.24E-06 | 1.32E-05 | 4.36239 |
| ARPP21 | -0.931 | 4.56856 | -5.3259 | 3.13E-07 | 2.39E-06 | 6.24538 |
| GAD2 | -0.9301 | 1.86024 | -4.3206 | 2.63E-05 | 0.000116 | 2.02014 |
| STX1B | -0.93 | 4.596512 | -6.3557 | 1.82E-09 | 2.83E-08 | 11.2052 |
| SOHLH1 | -0.9287 | 1.14455 | -6.1156 | 6.35E-09 | 8.32E-08 | 9.99781 |
| PANX2 | -0.9285 | 2.52856 | -6.6948 | 2.98E-10 | 6.05E-09 | 12.9581 |
| EMX2 | -0.9276 | 2.798995 | -5.9036 | 1.87E-08 | 2.11E-07 | 8.95666 |
| PPP1R1A | -0.9262 | 3.992779 | -4.9371 | 1.87E-06 | 1.13E-05 | 4.53242 |
| TUBB4A | -0.9255 | 7.357855 | -4.348 | 2.35E-05 | 0.000105 | 2.12618 |
| WBSCR17 | -0.9254 | 2.950639 | -4.0253 | 8.54E-05 | 0.000325 | 0.91141 |
| TGFBR3L | -0.9235 | 1.778563 | -4.5074 | 1.21E-05 | 5.83E-05 | 2.75404 |
| STAC2 | -0.923 | 2.384143 | -3.9676 | 0.00011 | 0.000395 | 0.70243 |
| CHN1 | -0.9228 | 6.539984 | -5.134 | 7.66E-07 | 5.16E-06 | 5.38823 |
| UNC13A | -0.9226 | 3.96059 | -5.5834 | 9.12E-08 | 8.19E-07 | 7.43018 |
| NDRG2 | -0.9215 | 9.91658 | -5.6993 | 5.17E-08 | 5.02E-07 | 7.9759 |
| KCNC1 | -0.9215 | 2.954283 | -6.1005 | 6.86E-09 | 8.91E-08 | 9.92295 |
| RIMS1 | -0.9206 | 2.590464 | -5.1528 | 7.02E-07 | 4.79E-06 | 5.47122 |
| RP11-245J24.1 | -0.9203 | 1.169882 | -6.1603 | 5.04E-09 | 6.84E-08 | 10.2204 |
| HTR2A | -0.9192 | 1.877723 | -6.1293 | 5.92E-09 | 7.85E-08 | 10.0663 |
| CPLX3 | -0.9189 | 1.509001 | -5.4444 | 1.78E-07 | 1.48E-06 | 6.78589 |
| CAMK4 | -0.918 | 2.450631 | -5.3014 | 3.51E-07 | 2.63E-06 | 6.13456 |
| CYP46A1 | -0.9179 | 4.104516 | -6.1195 | 6.23E-09 | 8.19E-08 | 10.0172 |
| SLC4A10 | -0.9164 | 2.793361 | -4.4842 | 1.34E-05 | 6.35E-05 | 2.66148 |
| CACNA2D3 | -0.9158 | 2.087042 | -6.1216 | 6.16E-09 | 8.11E-08 | 10.0277 |
| RLTPR | -0.9154 | 2.388813 | -5.6373 | 7.01E-08 | 6.55E-07 | 7.68322 |
| CNTNAP2 | -0.9148 | 3.369158 | -5.3396 | 2.94E-07 | 2.26E-06 | 6.30716 |
| HLF | -0.9139 | 3.941616 | -5.929 | 1.64E-08 | 1.90E-07 | 9.07996 |
| C11orf87 | -0.9137 | 1.449251 | -4.9708 | 1.61E-06 | 9.89E-06 | 4.67717 |
| LGI3 | -0.9131 | 4.160886 | -3.9476 | 0.00012 | 0.000423 | 0.63039 |
| RP1-293L6.1 | -0.9089 | 1.576856 | -5.3385 | 2.95E-07 | 2.27E-06 | 6.30215 |
| FAM19A1 | -0.9078 | 1.728417 | -5.4404 | 1.82E-07 | 1.50E-06 | 6.76718 |
| GABBR1 | -0.9077 | 7.423438 | -5.8729 | 2.18E-08 | 2.41E-07 | 8.80798 |
| RASAL1 | -0.9057 | 2.07649 | -4.4038 | 1.87E-05 | 8.52E-05 | 2.34401 |
| TNNT2 | -0.9048 | 1.459748 | -4.6216 | 7.48E-06 | 3.82E-05 | 3.21435 |
| USH1C | -0.9035 | 4.197406 | -3.0758 | 0.00245 | 0.006237 | -2.1992 |
| SGSM1 | -0.9017 | 2.321614 | -6.6222 | 4.41E-10 | 8.46E-09 | 12.5781 |
| RIMS2 | -0.8992 | 2.908057 | -5.0081 | 1.36E-06 | 8.54E-06 | 4.8382 |
| CELF3 | -0.8962 | 4.588002 | -4.1304 | 5.66E-05 | 0.000226 | 1.29867 |
| OLFM3 | -0.8954 | 1.49825 | -5.0531 | 1.11E-06 | 7.14E-06 | 5.03382 |
| GABRB3 | -0.8946 | 4.105321 | -4.2445 | 3.59E-05 | 0.000152 | 1.7283 |
| ARHGAP44 | -0.8927 | 2.78785 | -5.9836 | 1.25E-08 | 1.49E-07 | 9.34663 |
| ATP6V1G2 | -0.8914 | 6.575126 | -5.8537 | 2.40E-08 | 2.62E-07 | 8.71491 |
| RP11-2E11.9 | -0.8898 | 2.965637 | -5.1274 | 7.90E-07 | 5.30E-06 | 5.35886 |
| CAMSAP3 | -0.8888 | 2.66385 | -5.5384 | 1.13E-07 | 9.91E-07 | 7.22029 |
| SSTR2 | -0.8853 | 3.486178 | -4.717 | 4.95E-06 | 2.67E-05 | 3.60592 |
| CACNG8 | -0.8845 | 2.387366 | -5.926 | 1.67E-08 | 1.92E-07 | 9.06566 |
| RIMBP2 | -0.884 | 2.12022 | -5.0539 | 1.11E-06 | 7.12E-06 | 5.03727 |
| STX1A | -0.8837 | 4.496377 | -4.6087 | 7.90E-06 | 4.00E-05 | 3.16185 |
| CYP17A1-AS1 | -0.8834 | 1.847767 | -6.5776 | 5.60E-10 | 1.04E-08 | 12.346 |
| AC062021.1 | -0.8826 | 2.155264 | -3.4396 | 0.00073 | 0.002164 | -1.0924 |
| BEX2 | -0.8825 | 6.107167 | -5.4862 | 1.46E-07 | 1.24E-06 | 6.97825 |
| PDE2A | -0.8822 | 4.866872 | -4.8591 | 2.65E-06 | 1.54E-05 | 4.2007 |
| LMO3 | -0.8819 | 5.430724 | -4.7667 | 3.99E-06 | 2.20E-05 | 3.81268 |
| LYNX1 | -0.8817 | 4.86767 | -6.8574 | 1.23E-10 | 2.87E-09 | 13.8174 |
| CTD-2396E7.10 | -0.8812 | 3.685089 | -4.5708 | 9.28E-06 | 4.60E-05 | 3.00846 |
| GRIN3A | -0.8802 | 1.764228 | -5.1064 | 8.69E-07 | 5.76E-06 | 5.26673 |
| MOBP | -0.8788 | 5.647565 | -2.3379 | 0.02055 | 0.039409 | -4.0982 |
| NNAT | -0.8786 | 3.863959 | -2.9153 | 0.00403 | 0.009635 | -2.6524 |
| OGDHL | -0.878 | 2.672018 | -4.6289 | 7.24E-06 | 3.71E-05 | 3.24427 |
| SRRM4 | -0.8763 | 1.678401 | -5.8863 | 2.04E-08 | 2.27E-07 | 8.87262 |
| RIMS3 | -0.8748 | 3.540133 | -4.6369 | 7.00E-06 | 3.61E-05 | 3.27701 |
| ADAM11 | -0.8744 | 2.639481 | -5.8835 | 2.07E-08 | 2.30E-07 | 8.85932 |
| PRKAR1B | -0.8742 | 5.719335 | -5.2513 | 4.44E-07 | 3.22E-06 | 5.90924 |
| PRMT8 | -0.8717 | 1.791631 | -5.0713 | 1.02E-06 | 6.65E-06 | 5.11306 |
| CAMKK1 | -0.8714 | 3.200883 | -4.7424 | 4.43E-06 | 2.42E-05 | 3.71122 |
| HSPA12A | -0.8701 | 3.75311 | -6.3077 | 2.34E-09 | 3.53E-08 | 10.9617 |
| TPPP | -0.8688 | 5.199933 | -4.201 | 4.27E-05 | 0.000176 | 1.56351 |
| CHST1 | -0.868 | 4.811537 | -5.7411 | 4.21E-08 | 4.21E-07 | 8.17469 |
| KCNA1 | -0.8675 | 1.455639 | -5.4892 | 1.44E-07 | 1.22E-06 | 6.99241 |
| VWA5B2 | -0.8646 | 2.702173 | -5.989 | 1.21E-08 | 1.46E-07 | 9.37335 |
| SLC7A14 | -0.8631 | 2.816379 | -4.9132 | 2.09E-06 | 1.24E-05 | 4.43054 |
| NMNAT2 | -0.863 | 4.267414 | -4.8185 | 3.18E-06 | 1.80E-05 | 4.02949 |
| BSN | -0.8629 | 2.769321 | -6.0706 | 8.00E-09 | 1.02E-07 | 9.7749 |
| SCG3 | -0.8609 | 7.472059 | -3.9741 | 0.0001 | 0.000386 | 0.7258 |
| SYCE1 | -0.8571 | 1.337356 | -4.9736 | 1.59E-06 | 9.79E-06 | 4.68951 |
| RPRML | -0.8541 | 1.527393 | -4.5767 | 9.05E-06 | 4.51E-05 | 3.0324 |
| MTND2P28 | -0.8535 | 7.926592 | -7.0847 | 3.49E-11 | 9.94E-10 | 15.0377 |
| RP11-513I15.6 | -0.8527 | 3.686921 | -5.4026 | 2.18E-07 | 1.75E-06 | 6.5939 |
| ADARB2 | -0.8514 | 3.23184 | -4.2263 | 3.86E-05 | 0.000162 | 1.65911 |
| ASIC2 | -0.8498 | 1.390683 | -5.9981 | 1.16E-08 | 1.40E-07 | 9.41773 |
| ENHO | -0.8495 | 7.000636 | -4.4362 | 1.64E-05 | 7.57E-05 | 2.47136 |
| SOWAHA | -0.8491 | 2.51242 | -4.425 | 1.71E-05 | 7.88E-05 | 2.42725 |
| FAM19A2 | -0.8484 | 2.64432 | -4.9886 | 1.49E-06 | 9.21E-06 | 4.7542 |
| MT-ND2 | -0.8479 | 13.46061 | -9.8948 | 1.56E-18 | 1.12E-15 | 31.5239 |
| FGF13 | -0.8471 | 3.620016 | -4.4846 | 1.34E-05 | 6.34E-05 | 2.66292 |
| MICAL2 | -0.8464 | 3.59429 | -4.5044 | 1.23E-05 | 5.88E-05 | 2.74212 |
| GALNT9 | -0.8461 | 3.227719 | -3.9965 | 9.54E-05 | 0.000358 | 0.80687 |
| TAC3 | -0.8455 | 1.876491 | -3.6397 | 0.00036 | 0.001167 | -0.4377 |
| CA4 | -0.8423 | 3.364087 | -4.9156 | 2.06E-06 | 1.23E-05 | 4.44077 |
| CORO6 | -0.8417 | 2.981519 | -5.5669 | 9.88E-08 | 8.80E-07 | 7.35335 |
| MGAT5B | -0.8395 | 3.345444 | -5.178 | 6.25E-07 | 4.33E-06 | 5.58256 |
| GRM3 | -0.839 | 3.936438 | -4.1784 | 4.67E-05 | 0.000191 | 1.47845 |
| CASKIN1 | -0.8388 | 4.194484 | -5.1634 | 6.69E-07 | 4.59E-06 | 5.51804 |
| NPM2 | -0.8375 | 2.911759 | -4.4839 | 1.34E-05 | 6.35E-05 | 2.66017 |
| PNMAL2 | -0.8362 | 3.301666 | -5.6744 | 5.84E-08 | 5.59E-07 | 7.85834 |
| PDZD4 | -0.8361 | 6.870894 | -5.8748 | 2.16E-08 | 2.39E-07 | 8.81703 |
| ATP2B2 | -0.8357 | 4.060551 | -4.9517 | 1.76E-06 | 1.07E-05 | 4.59499 |
| WNT10B | -0.835 | 1.482234 | -5.18 | 6.19E-07 | 4.30E-06 | 5.59151 |
| GLT1D1 | -0.8342 | 1.723761 | -4.6811 | 5.79E-06 | 3.05E-05 | 3.45805 |
| BEX5 | -0.8313 | 3.406192 | -3.8342 | 0.00018 | 0.00062 | 0.22846 |
| SHANK2 | -0.8302 | 3.111016 | -5.0427 | 1.16E-06 | 7.45E-06 | 4.98832 |
| STXBP1 | -0.8297 | 5.951553 | -5.3862 | 2.35E-07 | 1.86E-06 | 6.5192 |
| FAIM2 | -0.8284 | 6.918898 | -5.8704 | 2.21E-08 | 2.44E-07 | 8.79592 |
| FAM153B | -0.8283 | 1.22382 | -5.1637 | 6.68E-07 | 4.59E-06 | 5.51909 |
| SCG5 | -0.8282 | 6.016034 | -4.0487 | 7.80E-05 | 0.0003 | 0.99687 |
| CTD-2380F24.1 | -0.8261 | 1.810608 | -5.3646 | 2.61E-07 | 2.03E-06 | 6.4209 |
| TUBA8 | -0.8253 | 2.57001 | -4.608 | 7.92E-06 | 4.01E-05 | 3.15909 |
| ATP8A2 | -0.8246 | 1.734843 | -5.1146 | 8.37E-07 | 5.58E-06 | 5.30254 |
| KIF1A | -0.8231 | 6.872781 | -6.2082 | 3.93E-09 | 5.54E-08 | 10.4604 |
| RAPGEF4 | -0.8202 | 5.185885 | -4.6736 | 5.98E-06 | 3.13E-05 | 3.42712 |
| PPP1R16B | -0.8185 | 3.674138 | -4.3168 | 2.68E-05 | 0.000117 | 2.00557 |
| FBXO41 | -0.8171 | 3.536938 | -6.0533 | 8.74E-09 | 1.10E-07 | 9.68948 |
| ZFR2 | -0.8169 | 2.277839 | -4.4326 | 1.66E-05 | 7.67E-05 | 2.45704 |
| JPH4 | -0.8145 | 5.172301 | -4.6644 | 6.22E-06 | 3.25E-05 | 3.3895 |
| TPD52L1 | -0.8139 | 4.602244 | -4.1828 | 4.59E-05 | 0.000188 | 1.4948 |
| RP11-423G4.7 | -0.8126 | 1.139269 | -6.4606 | 1.05E-09 | 1.78E-08 | 11.7416 |
| MMD2 | -0.8121 | 4.002273 | -3.749 | 0.00024 | 0.000821 | -0.067 |
| AC007192.6 | -0.8101 | 2.393029 | -3.4179 | 0.00079 | 0.002309 | -1.1613 |
| FBXO2 | -0.8078 | 5.225041 | -3.461 | 0.00068 | 0.002026 | -1.0237 |
| FSTL5 | -0.8074 | 2.287161 | -4.1237 | 5.81E-05 | 0.000231 | 1.27385 |
| KSR2 | -0.8072 | 1.336191 | -7.2959 | 1.06E-11 | 3.58E-10 | 16.1905 |
| CUX2 | -0.807 | 2.016993 | -4.5753 | 9.11E-06 | 4.53E-05 | 3.02689 |
| ATP1A2 | -0.8067 | 7.658459 | -3.9486 | 0.00011 | 0.000422 | 0.634 |
| CHRNA4 | -0.8055 | 2.337958 | -4.8009 | 3.43E-06 | 1.93E-05 | 3.95564 |
| FRRS1L | -0.8045 | 3.100655 | -4.5623 | 9.62E-06 | 4.75E-05 | 2.97436 |
| SLC32A1 | -0.8025 | 1.830186 | -3.794 | 0.00021 | 0.000708 | 0.08828 |
| RXFP1 | -0.8017 | 1.149105 | -5.8066 | 3.04E-08 | 3.19E-07 | 8.48815 |
| TAGLN3 | -0.8016 | 5.906667 | -3.9775 | 0.0001 | 0.000382 | 0.73794 |
| RP11-320H14.1 | -0.7992 | 1.871512 | -3.8548 | 0.00016 | 0.000578 | 0.30089 |
| RP11-731J8.2 | -0.799 | 2.859043 | -4.9143 | 2.08E-06 | 1.24E-05 | 4.4351 |
| PRSS3 | -0.7963 | 2.320567 | -4.3381 | 2.45E-05 | 0.000108 | 2.08778 |
| RCAN2 | -0.7956 | 4.396135 | -4.266 | 3.29E-05 | 0.00014 | 1.81057 |
| KLK7 | -0.7936 | 1.114667 | -4.5948 | 8.38E-06 | 4.22E-05 | 3.10547 |
| C15orf59 | -0.793 | 6.047147 | -5.1591 | 6.82E-07 | 4.67E-06 | 5.49896 |
| DGCR5 | -0.7909 | 3.617828 | -5.4429 | 1.80E-07 | 1.49E-06 | 6.77873 |
| TNR | -0.7878 | 5.516575 | -2.8775 | 0.00452 | 0.010646 | -2.7558 |
| CLVS2 | -0.7868 | 2.078719 | -4.6356 | 7.04E-06 | 3.62E-05 | 3.27168 |
| CELF5 | -0.7864 | 3.92066 | -4.0128 | 8.96E-05 | 0.000338 | 0.86593 |
| HS3ST4 | -0.7859 | 1.998065 | -4.6741 | 5.96E-06 | 3.13E-05 | 3.42923 |
| PNMA3 | -0.784 | 2.738867 | -4.7885 | 3.62E-06 | 2.02E-05 | 3.90347 |
| HMGCLL1 | -0.7822 | 2.224476 | -4.9665 | 1.64E-06 | 1.01E-05 | 4.65858 |
| CNNM1 | -0.782 | 1.599395 | -5.4198 | 2.01E-07 | 1.63E-06 | 6.67267 |
| CARNS1 | -0.7819 | 4.306103 | -2.6415 | 0.00902 | 0.019402 | -3.3743 |
| 3-Sep | -0.7817 | 6.761369 | -5.8545 | 2.39E-08 | 2.61E-07 | 8.71908 |
| DIRAS2 | -0.781 | 3.730292 | -4.0966 | 6.46E-05 | 0.000254 | 1.17345 |
| RP11-81K13.1 | -0.7805 | 1.125033 | -4.8317 | 3.00E-06 | 1.71E-05 | 4.08501 |
| NECAB2 | -0.7795 | 3.865005 | -4.5229 | 1.14E-05 | 5.50E-05 | 2.81587 |
| KBTBD11 | -0.7793 | 4.482275 | -5.7876 | 3.34E-08 | 3.47E-07 | 8.39724 |
| KCNS1 | -0.7788 | 1.194549 | -4.1854 | 4.55E-05 | 0.000187 | 1.50476 |
| HCN1 | -0.7788 | 1.310641 | -4.4263 | 1.70E-05 | 7.85E-05 | 2.43229 |
| CDH18 | -0.778 | 2.574524 | -4.3957 | 1.93E-05 | 8.78E-05 | 2.31221 |
| CPNE9 | -0.7773 | 1.335734 | -5.3883 | 2.33E-07 | 1.85E-06 | 6.52865 |
| SPHKAP | -0.7772 | 2.366223 | -3.9589 | 0.00011 | 0.000407 | 0.67093 |
| VIP | -0.7771 | 1.238898 | -4.6533 | 6.52E-06 | 3.39E-05 | 3.34376 |
| LPPR3 | -0.777 | 3.183171 | -3.671 | 0.00032 | 0.001056 | -0.3327 |
| MT-RNR2 | -0.7765 | 13.90149 | -8.21 | 5.19E-14 | 3.81E-12 | 21.3689 |
| CACNA1B | -0.7762 | 1.585352 | -4.7774 | 3.80E-06 | 2.11E-05 | 3.85711 |
| ADAP1 | -0.7751 | 4.649067 | -4.2817 | 3.09E-05 | 0.000133 | 1.87064 |
| CHRNB2 | -0.7737 | 2.72164 | -4.7364 | 4.55E-06 | 2.47E-05 | 3.68654 |
| RGR | -0.7706 | 3.270156 | -3.3282 | 0.00107 | 0.003018 | -1.4427 |
| C12orf39 | -0.769 | 3.384881 | -3.2624 | 0.00133 | 0.003656 | -1.645 |
| CA10 | -0.7685 | 4.012581 | -2.9719 | 0.00339 | 0.008285 | -2.4951 |
| NEUROD6 | -0.7685 | 1.239937 | -4.0396 | 8.08E-05 | 0.00031 | 0.96362 |
| NRIP3 | -0.7683 | 2.554457 | -4.1258 | 5.76E-05 | 0.00023 | 1.28172 |
| DYNC1I1 | -0.766 | 3.787969 | -4.1697 | 4.84E-05 | 0.000197 | 1.44554 |
| RTN4RL1 | -0.7659 | 1.407737 | -4.6794 | 5.83E-06 | 3.07E-05 | 3.45104 |
| CXXC11 | -0.7649 | 3.067799 | -3.7021 | 0.00029 | 0.000956 | -0.2272 |
| ZDHHC22 | -0.7634 | 5.220229 | -3.3947 | 0.00085 | 0.002473 | -1.2347 |
| NAP1L2 | -0.7621 | 4.133293 | -4.5611 | 9.67E-06 | 4.77E-05 | 2.9694 |
| SYNGR1 | -0.7616 | 5.303262 | -6.1122 | 6.46E-09 | 8.45E-08 | 9.98129 |
| TEF | -0.7614 | 4.454099 | -6.6133 | 4.62E-10 | 8.77E-09 | 12.5319 |
| AC123886.2 | -0.7606 | 1.028519 | -6.0442 | 9.16E-09 | 1.14E-07 | 9.64469 |
| FAM155A | -0.7605 | 3.771329 | -4.1831 | 4.59E-05 | 0.000188 | 1.49623 |
| DACH2 | -0.7605 | 1.811883 | -4.2675 | 3.27E-05 | 0.00014 | 1.81633 |
| ERMN | -0.7596 | 5.183846 | -2.3005 | 0.02263 | 0.042871 | -4.1817 |
| CPE | -0.7585 | 9.667947 | -5.0107 | 1.34E-06 | 8.45E-06 | 4.84968 |
| TMEM179 | -0.758 | 3.842403 | -3.6395 | 0.00036 | 0.001168 | -0.4384 |
| TAC1 | -0.7578 | 2.255255 | -2.9068 | 0.00414 | 0.009857 | -2.6758 |
| PCDH8 | -0.7575 | 2.357931 | -4.4596 | 1.48E-05 | 6.96E-05 | 2.56366 |
| GPR22 | -0.7566 | 1.36882 | -4.65 | 6.62E-06 | 3.43E-05 | 3.3303 |
| LINC00320 | -0.7559 | 3.586173 | -3.7524 | 0.00024 | 0.000812 | -0.0554 |
| PNCK | -0.7527 | 4.110812 | -5.0643 | 1.05E-06 | 6.84E-06 | 5.08229 |
| FAM57B | -0.7525 | 4.28594 | -4.226 | 3.86E-05 | 0.000162 | 1.65793 |
| AMER3 | -0.7519 | 1.745932 | -4.8465 | 2.81E-06 | 1.61E-05 | 4.14749 |
| NECAB1 | -0.7513 | 3.244425 | -3.661 | 0.00033 | 0.00109 | -0.3662 |
| MRO | -0.7508 | 4.087582 | -4.6446 | 6.77E-06 | 3.50E-05 | 3.30847 |
| RYR2 | -0.7501 | 1.645039 | -4.4717 | 1.41E-05 | 6.66E-05 | 2.61172 |
| NRXN3 | -0.7497 | 3.209051 | -4.5704 | 9.30E-06 | 4.60E-05 | 3.00693 |
| ASPHD1 | -0.7494 | 4.723937 | -4.6715 | 6.03E-06 | 3.16E-05 | 3.41838 |
| LINC00599 | -0.7484 | 3.387069 | -3.7831 | 0.00021 | 0.000734 | 0.05065 |
| PITPNM3 | -0.7484 | 2.351675 | -4.3882 | 2.00E-05 | 9.02E-05 | 2.28273 |
| CAMK2N2 | -0.7474 | 4.359765 | -4.753 | 4.23E-06 | 2.32E-05 | 3.7555 |
| PCSK2 | -0.7466 | 3.250326 | -3.3798 | 0.0009 | 0.002585 | -1.2817 |
| WASF3 | -0.7465 | 5.663227 | -6.2546 | 3.09E-09 | 4.53E-08 | 10.6933 |
| MUSTN1 | -0.7462 | 2.53193 | -4.6967 | 5.41E-06 | 2.88E-05 | 3.52218 |
| LINC00617 | -0.746 | 1.500236 | -4.5495 | 1.02E-05 | 4.98E-05 | 2.92269 |
| RP1-257A7.5 | -0.7456 | 4.485872 | -5.4167 | 2.03E-07 | 1.66E-06 | 6.65879 |
| CACNA1I | -0.7453 | 1.196906 | -6.63 | 4.23E-10 | 8.18E-09 | 12.6189 |
| RGS20 | -0.7452 | 2.994252 | -4.4435 | 1.59E-05 | 7.38E-05 | 2.50038 |
| LY86-AS1 | -0.7451 | 0.844604 | -5.1319 | 7.73E-07 | 5.21E-06 | 5.37872 |
| RP11-416I2.1 | -0.7435 | 0.706214 | -5.7494 | 4.04E-08 | 4.07E-07 | 8.21426 |
| PPP1R1B | -0.7433 | 5.162504 | -3.4426 | 0.00072 | 0.002144 | -1.0828 |
| VIPR1 | -0.742 | 1.960654 | -5.6249 | 7.45E-08 | 6.90E-07 | 7.6248 |
| TDRD9 | -0.7416 | 1.724039 | -5.1557 | 6.93E-07 | 4.73E-06 | 5.48412 |
| AGAP2 | -0.7392 | 5.080837 | -3.4207 | 0.00078 | 0.002291 | -1.1525 |
| STXBP6 | -0.7387 | 3.446302 | -4.7189 | 4.91E-06 | 2.65E-05 | 3.61381 |
| HAPLN1 | -0.7385 | 3.631439 | -2.9233 | 0.00393 | 0.009429 | -2.6301 |
| TCEAL5 | -0.7377 | 5.138677 | -4.8799 | 2.42E-06 | 1.42E-05 | 4.28858 |
| COX7A1 | -0.7368 | 3.611619 | -4.2415 | 3.63E-05 | 0.000153 | 1.71714 |
| RTBDN | -0.7367 | 1.434057 | -4.6503 | 6.61E-06 | 3.43E-05 | 3.33167 |
| 4-Mar | -0.7358 | 1.94788 | -4.296 | 2.91E-05 | 0.000126 | 1.92548 |
| CBX7 | -0.7352 | 4.83926 | -6.6057 | 4.82E-10 | 9.08E-09 | 12.4923 |
| GDAP1L1 | -0.7335 | 5.466294 | -3.3281 | 0.00107 | 0.003018 | -1.4432 |
| RP11-434B12.1 | -0.7315 | 1.809511 | -5.7336 | 4.37E-08 | 4.34E-07 | 8.13892 |
| KCNV1 | -0.731 | 1.034828 | -4.5131 | 1.18E-05 | 5.70E-05 | 2.77688 |
| TMEM151A | -0.7308 | 3.986855 | -3.4872 | 0.00062 | 0.00187 | -0.9394 |
| ABLIM2 | -0.7305 | 3.311607 | -5.3953 | 2.25E-07 | 1.80E-06 | 6.56091 |
| PTPRR | -0.7302 | 1.507891 | -4.7842 | 3.69E-06 | 2.05E-05 | 3.88561 |
| CTD-3193O13.11 | -0.7301 | 3.582992 | -4.1238 | 5.81E-05 | 0.000231 | 1.27412 |
| PART1 | -0.7285 | 1.387069 | -4.8164 | 3.21E-06 | 1.81E-05 | 4.02037 |
| AC090425.1 | -0.7283 | 0.772751 | -5.7503 | 4.02E-08 | 4.06E-07 | 8.21881 |
| MT-ND5 | -0.7283 | 12.49035 | -8.6068 | 4.75E-15 | 5.42E-13 | 23.6991 |
| TF | -0.7283 | 7.780961 | -2.7802 | 0.00604 | 0.01372 | -3.0169 |
| PRRT1 | -0.7253 | 4.597154 | -6.473 | 9.79E-10 | 1.68E-08 | 11.8054 |
| HSD17B6 | -0.725 | 3.491572 | -4.6944 | 5.46E-06 | 2.90E-05 | 3.51283 |
| FRMPD2P1 | -0.7241 | 1.42296 | -4.8725 | 2.50E-06 | 1.46E-05 | 4.25725 |
| RAB11FIP4 | -0.7236 | 4.303108 | -4.5716 | 9.25E-06 | 4.59E-05 | 3.01165 |
| ENTPD3 | -0.723 | 1.250949 | -5.1064 | 8.69E-07 | 5.76E-06 | 5.26673 |
| RP13-514E23.1 | -0.7228 | 2.830565 | -5.6571 | 6.36E-08 | 6.01E-07 | 7.77645 |
| GDF10 | -0.7225 | 2.247942 | -3.82 | 0.00019 | 0.000651 | 0.17883 |
| ELAVL2 | -0.7206 | 3.071027 | -3.646 | 0.00035 | 0.001143 | -0.4168 |
| MCF2L2 | -0.72 | 3.316525 | -5.265 | 4.17E-07 | 3.05E-06 | 5.97071 |
| FRMPD4 | -0.7196 | 1.127732 | -4.9256 | 1.97E-06 | 1.18E-05 | 4.48327 |
| SCD | -0.7194 | 8.258599 | -4.5681 | 9.39E-06 | 4.64E-05 | 2.99749 |
| MT-ND4 | -0.7173 | 14.12822 | -9.7511 | 3.87E-18 | 2.41E-15 | 30.6342 |
| SH3GL3 | -0.716 | 3.970187 | -3.4724 | 0.00065 | 0.001958 | -0.9874 |
| SLC24A2 | -0.7159 | 3.074331 | -3.6893 | 0.0003 | 0.000996 | -0.2705 |
| MRVI1 | -0.7156 | 4.533006 | -4.8819 | 2.40E-06 | 1.41E-05 | 4.29716 |
| NELL2 | -0.7154 | 4.510379 | -3.0211 | 0.00291 | 0.00725 | -2.3561 |
| MFSD4 | -0.7151 | 3.580916 | -4.5748 | 9.13E-06 | 4.54E-05 | 3.0245 |
| MT-CYB | -0.713 | 13.07718 | -8.428 | 1.40E-14 | 1.29E-12 | 22.6435 |
| SLC1A6 | -0.7117 | 2.448253 | -3.9361 | 0.00012 | 0.00044 | 0.58929 |
| PDXP | -0.7117 | 5.30073 | -4.3189 | 2.65E-05 | 0.000116 | 2.0138 |
| PAPLN | -0.7113 | 3.200376 | -4.2788 | 3.12E-05 | 0.000134 | 1.85961 |
| CPNE7 | -0.711 | 1.684377 | -4.3021 | 2.84E-05 | 0.000124 | 1.94888 |
| OPCML | -0.711 | 3.439473 | -3.821 | 0.00019 | 0.000649 | 0.1823 |
| ALDH1A1 | -0.7088 | 4.767388 | -3.3949 | 0.00085 | 0.002472 | -1.2342 |
| PRDM8 | -0.7065 | 2.775237 | -4.0456 | 7.89E-05 | 0.000303 | 0.98552 |
| GRIN2B | -0.7056 | 1.177191 | -5.0706 | 1.02E-06 | 6.66E-06 | 5.10999 |
| RAP1GAP | -0.7038 | 4.874767 | -4.6897 | 5.58E-06 | 2.95E-05 | 3.49319 |
| FAM131C | -0.7027 | 2.09796 | -5.1698 | 6.49E-07 | 4.47E-06 | 5.54638 |
| TRHDE | -0.7023 | 1.084107 | -5.0166 | 1.31E-06 | 8.26E-06 | 4.87511 |
| HIF3A | -0.7001 | 3.150443 | -3.5433 | 0.00051 | 0.001578 | -0.7572 |
| MT-ND4L | -0.6992 | 13.81607 | -8.2085 | 5.24E-14 | 3.83E-12 | 21.3601 |
| MT-ATP6 | -0.6991 | 14.44867 | -9.7473 | 3.97E-18 | 2.41E-15 | 30.6109 |
| TMEM235 | -0.699 | 2.548392 | -2.803 | 0.00565 | 0.012936 | -2.9563 |
| MT-ATP8 | -0.6983 | 14.89972 | -6.7417 | 2.31E-10 | 4.88E-09 | 13.2048 |
| CTD-2023N9.3 | -0.6978 | 0.866623 | -4.7121 | 5.06E-06 | 2.72E-05 | 3.58567 |
| DUSP26 | -0.6974 | 4.764065 | -4.2329 | 3.76E-05 | 0.000158 | 1.68421 |
| RP11-361F15.2 | -0.6969 | 2.288154 | -4.8931 | 2.28E-06 | 1.35E-05 | 4.34472 |
| FAM153C | -0.6957 | 1.140904 | -4.5853 | 8.73E-06 | 4.36E-05 | 3.06704 |
| CABLES1 | -0.6953 | 3.631411 | -4.6744 | 5.96E-06 | 3.13E-05 | 3.43034 |
| CCKBR | -0.695 | 1.585179 | -3.6732 | 0.00032 | 0.001049 | -0.325 |
| SEMA4A | -0.6947 | 3.191717 | -5.8493 | 2.45E-08 | 2.66E-07 | 8.6938 |
| C7orf41 | -0.6945 | 7.300984 | -4.7522 | 4.25E-06 | 2.33E-05 | 3.75218 |
| RTN4RL2 | -0.6929 | 3.529909 | -4.2518 | 3.48E-05 | 0.000148 | 1.75617 |
| LINC00634 | -0.6926 | 4.463891 | -4.7623 | 4.06E-06 | 2.24E-05 | 3.7943 |
| SDS | -0.6921 | 3.320933 | -3.9002 | 0.00014 | 0.000498 | 0.46122 |
| KCNJ3 | -0.6918 | 2.022336 | -4.0697 | 7.18E-05 | 0.000279 | 1.07398 |
| DLGAP1 | -0.6914 | 4.871225 | -4.2313 | 3.78E-05 | 0.000159 | 1.67828 |
| PTER | -0.6898 | 2.104201 | -3.8759 | 0.00015 | 0.000539 | 0.37517 |
| HBQ1 | -0.6894 | 1.267693 | -5.4096 | 2.10E-07 | 1.70E-06 | 6.62634 |
| MAGEE1 | -0.6891 | 3.095549 | -5.2185 | 5.18E-07 | 3.67E-06 | 5.76292 |
| CEND1 | -0.6872 | 5.556285 | -4.4259 | 1.71E-05 | 7.86E-05 | 2.43074 |
| SEPT7P3 | -0.6871 | 1.032536 | -2.6183 | 0.00963 | 0.020532 | -3.4326 |
| CES4A | -0.6857 | 2.745109 | -3.8894 | 0.00014 | 0.000515 | 0.42299 |
| ATCAY | -0.6854 | 5.3946 | -2.943 | 0.0037 | 0.008949 | -2.5757 |
| ATOH8 | -0.6848 | 4.709246 | -3.1751 | 0.00178 | 0.004699 | -1.9077 |
| RP11-481A20.10 | -0.6829 | 1.149042 | -4.2902 | 2.98E-05 | 0.000129 | 1.90328 |
| SLC29A2 | -0.6829 | 2.676239 | -4.9489 | 1.78E-06 | 1.08E-05 | 4.58318 |
| TMEM88B | -0.6813 | 3.264412 | -2.4076 | 0.01712 | 0.033675 | -3.9393 |
| PABPC1L2B | -0.6808 | 0.968016 | -5.3775 | 2.45E-07 | 1.93E-06 | 6.47946 |
| DCTN1-AS1 | -0.6803 | 1.238256 | -5.1292 | 7.83E-07 | 5.26E-06 | 5.36677 |
| ISLR2 | -0.6786 | 1.799606 | -3.8971 | 0.00014 | 0.000503 | 0.45019 |
| MT-RNR1 | -0.6775 | 12.55027 | -6.0068 | 1.11E-08 | 1.35E-07 | 9.46081 |
| FAM81A | -0.6764 | 3.354225 | -4.4856 | 1.33E-05 | 6.32E-05 | 2.66694 |
| NSG1 | -0.6763 | 4.596296 | -3.2762 | 0.00127 | 0.003512 | -1.603 |
| RP11-355I22.7 | -0.6758 | 2.506254 | -3.7199 | 0.00027 | 0.000902 | -0.1667 |
| GABRA2 | -0.6757 | 2.767803 | -3.1218 | 0.00211 | 0.005476 | -2.0653 |
| TMEM59L | -0.6756 | 7.059496 | -5.6316 | 7.21E-08 | 6.70E-07 | 7.65634 |
| RP11-285F16.1 | -0.6756 | 1.931954 | -4.5892 | 8.58E-06 | 4.31E-05 | 3.08283 |
| MT-ND6 | -0.6755 | 13.10905 | -7.9643 | 2.23E-13 | 1.31E-11 | 19.9494 |
| FAM107A | -0.6753 | 8.359784 | -3.6376 | 0.00036 | 0.001175 | -0.4449 |
| DMRTC1B | -0.6732 | 2.947569 | -5.6038 | 8.26E-08 | 7.52E-07 | 7.52554 |
| HAPLN4 | -0.6727 | 2.100537 | -3.4487 | 0.00071 | 0.002104 | -1.0633 |
| ENC1 | -0.6722 | 4.968119 | -2.7445 | 0.00671 | 0.015021 | -3.1104 |
| ACHE | -0.6719 | 3.477176 | -4.6809 | 5.79E-06 | 3.05E-05 | 3.45697 |
| PPP1R13B | -0.6702 | 3.425748 | -7.5996 | 1.87E-12 | 7.88E-11 | 17.8788 |
| KCNK4 | -0.6701 | 1.607996 | -5.0234 | 1.27E-06 | 8.04E-06 | 4.90451 |
| LRTM2 | -0.6701 | 1.806828 | -3.576 | 0.00045 | 0.001427 | -0.6496 |
| CBLN4 | -0.6699 | 1.201682 | -3.5572 | 0.00049 | 0.001513 | -0.7115 |
| TRIM67 | -0.6682 | 2.127049 | -2.5903 | 0.01042 | 0.021963 | -3.5022 |
| RASGRF2 | -0.668 | 2.55376 | -4.3653 | 2.19E-05 | 9.82E-05 | 2.19354 |
| CALB1 | -0.6672 | 2.144781 | -3.5245 | 0.00054 | 0.001668 | -0.8185 |
| 5-Sep | -0.6671 | 6.62427 | -4.1616 | 5.00E-05 | 0.000203 | 1.41519 |
| LINC00086 | -0.6646 | 2.830823 | -5.1542 | 6.98E-07 | 4.76E-06 | 5.47731 |
| SERTM1 | -0.6643 | 0.961254 | -4.6787 | 5.85E-06 | 3.08E-05 | 3.44824 |
| ZMAT4 | -0.6629 | 1.457562 | -4.5358 | 1.08E-05 | 5.24E-05 | 2.86747 |
| HAPLN2 | -0.6625 | 5.269041 | -2.318 | 0.02163 | 0.041209 | -4.1427 |
| SEC14L5 | -0.6618 | 2.210196 | -3.1834 | 0.00173 | 0.004591 | -1.8832 |
| MAST3 | -0.6617 | 3.840299 | -5.126 | 7.95E-07 | 5.34E-06 | 5.35281 |
| CXCL14 | -0.6617 | 4.253255 | -2.3691 | 0.01895 | 0.03676 | -4.0277 |
| GRM5 | -0.6597 | 1.930492 | -3.6951 | 0.0003 | 0.000978 | -0.2509 |
| SLC22A6 | -0.6594 | 1.56043 | -4.6298 | 7.22E-06 | 3.70E-05 | 3.24802 |
| GPR27 | -0.6594 | 3.535549 | -3.6655 | 0.00033 | 0.001074 | -0.3512 |
| NCDN | -0.659 | 5.688231 | -3.8995 | 0.00014 | 0.000499 | 0.45884 |
| BCYRN1 | -0.659 | 1.650131 | -3.504 | 0.00059 | 0.001779 | -0.885 |
| RP11-1C8.7 | -0.6585 | 1.337934 | -4.5187 | 1.16E-05 | 5.58E-05 | 2.79921 |
| RGS7BP | -0.6584 | 2.513262 | -4.0239 | 8.58E-05 | 0.000326 | 0.90651 |
| BEGAIN | -0.658 | 2.728778 | -4.0072 | 9.16E-05 | 0.000345 | 0.84563 |
| KCNK1 | -0.6578 | 3.530182 | -3.1596 | 0.00187 | 0.004909 | -1.9538 |
| LRRC7 | -0.6574 | 2.119855 | -4.2303 | 3.80E-05 | 0.00016 | 1.67428 |
| NCS1 | -0.6569 | 5.372977 | -4.2781 | 3.13E-05 | 0.000135 | 1.8568 |
| GPR98 | -0.6562 | 4.752873 | -3.3164 | 0.00111 | 0.00312 | -1.4792 |
| GRAMD1B | -0.6557 | 4.002135 | -6.0831 | 7.50E-09 | 9.64E-08 | 9.83698 |
| RP11-297M9.2 | -0.6555 | 1.824531 | -3.7051 | 0.00029 | 0.000947 | -0.2171 |
| CLVS1 | -0.6544 | 2.034831 | -4.0497 | 7.77E-05 | 0.000299 | 1.00065 |
| PRR18 | -0.6536 | 3.519294 | -3.1197 | 0.00213 | 0.005508 | -2.0714 |
| KCNK3 | -0.6535 | 2.020726 | -4.0541 | 7.63E-05 | 0.000294 | 1.01681 |
| FEZF2 | -0.6532 | 1.78216 | -4.8827 | 2.39E-06 | 1.40E-05 | 4.30053 |
| AC013268.2 | -0.6531 | 0.909996 | -4.4674 | 1.44E-05 | 6.76E-05 | 2.59481 |
| RIIAD1 | -0.6527 | 2.632184 | -3.617 | 0.00039 | 0.001254 | -0.5137 |
| S100A1 | -0.6525 | 7.067408 | -2.8462 | 0.00497 | 0.011556 | -2.8407 |
| KIAA0513 | -0.6521 | 4.543876 | -5.2967 | 3.59E-07 | 2.68E-06 | 6.1136 |
| SYBU | -0.6521 | 5.702032 | -5.1481 | 7.18E-07 | 4.88E-06 | 5.45014 |
| MTATP6P1 | -0.6519 | 11.4808 | -7.8651 | 4.00E-13 | 2.10E-11 | 19.3818 |
| SHANK3 | -0.6519 | 3.573047 | -5.6735 | 5.87E-08 | 5.60E-07 | 7.8537 |
| SPTBN4 | -0.6518 | 4.016487 | -5.3623 | 2.64E-07 | 2.05E-06 | 6.41015 |
| ALDH2 | -0.6498 | 7.579409 | -5.1452 | 7.27E-07 | 4.93E-06 | 5.43768 |
| HPSE2 | -0.6492 | 2.015973 | -3.2231 | 0.00152 | 0.0041 | -1.7641 |
| AMZ1 | -0.6477 | 2.459102 | -4.0169 | 8.82E-05 | 0.000334 | 0.88073 |
| KCNAB2 | -0.6456 | 4.669665 | -3.8792 | 0.00015 | 0.000533 | 0.38681 |
| HECW1 | -0.6456 | 1.904373 | -4.3755 | 2.10E-05 | 9.46E-05 | 2.23338 |
| COL26A1 | -0.6441 | 1.788582 | -4.4384 | 1.62E-05 | 7.51E-05 | 2.48 |
| SLC4A4 | -0.644 | 5.59825 | -3.7711 | 0.00022 | 0.000763 | 0.00923 |
| BZRAP1 | -0.6437 | 5.278567 | -4.5147 | 1.18E-05 | 5.67E-05 | 2.78319 |
| SEZ6L | -0.6435 | 5.675631 | -2.5539 | 0.01152 | 0.023941 | -3.5916 |
| IQCA1 | -0.643 | 2.060511 | -3.9261 | 0.00013 | 0.000455 | 0.55352 |
| MAP1A | -0.643 | 5.963572 | -5.7645 | 3.75E-08 | 3.81E-07 | 8.2864 |
| RP11-482M8.3 | -0.6428 | 1.022184 | -6.49 | 8.95E-10 | 1.55E-08 | 11.8929 |
| TMEM132D | -0.6425 | 1.320808 | -4.062 | 7.40E-05 | 0.000287 | 1.04595 |
| AC131056.3 | -0.6422 | 1.171665 | -4.7399 | 4.48E-06 | 2.44E-05 | 3.70104 |
| GOT1 | -0.6408 | 5.337108 | -4.9362 | 1.88E-06 | 1.13E-05 | 4.52869 |
| TSPYL2 | -0.6402 | 6.170222 | -5.4222 | 1.98E-07 | 1.62E-06 | 6.68376 |
| CCDC64 | -0.6399 | 2.582201 | -3.6712 | 0.00032 | 0.001055 | -0.3318 |
| CA7 | -0.6398 | 0.938188 | -4.8707 | 2.52E-06 | 1.47E-05 | 4.24957 |
| KCNB1 | -0.6389 | 3.331087 | -4.4691 | 1.43E-05 | 6.72E-05 | 2.60141 |
| STYK1 | -0.6382 | 0.898536 | -4.6435 | 6.80E-06 | 3.52E-05 | 3.304 |
| FAM153A | -0.6369 | 1.022582 | -4.7873 | 3.64E-06 | 2.03E-05 | 3.89845 |
| FLJ30594 | -0.6362 | 3.366121 | -3.704 | 0.00029 | 0.00095 | -0.2207 |
| FAM19A5 | -0.6361 | 5.716293 | -5.0165 | 1.31E-06 | 8.26E-06 | 4.87459 |
| ZDHHC8P1 | -0.636 | 1.587403 | -3.8935 | 0.00014 | 0.000509 | 0.43759 |
| UNC79 | -0.636 | 2.770898 | -4.0766 | 6.99E-05 | 0.000272 | 1.09945 |
| HHATL | -0.6354 | 4.6705 | -2.736 | 0.00688 | 0.015367 | -3.1326 |
| KCNJ16 | -0.6347 | 3.906989 | -2.8271 | 0.00526 | 0.012147 | -2.8919 |
| SLC6A1 | -0.6337 | 6.11378 | -4.3964 | 1.93E-05 | 8.76E-05 | 2.31482 |
| PAK3 | -0.6331 | 2.818937 | -4.0845 | 6.78E-05 | 0.000265 | 1.12872 |
| PEX5L | -0.6316 | 3.332556 | -3.089 | 0.00234 | 0.006013 | -2.1609 |
| SPOCK3 | -0.6315 | 4.709346 | -2.5488 | 0.01169 | 0.024234 | -3.6042 |
| APLP1 | -0.6315 | 8.236796 | -3.8441 | 0.00017 | 0.0006 | 0.26313 |
| MT-CO1 | -0.6311 | 14.65839 | -8.779 | 1.66E-15 | 2.30E-13 | 24.7239 |
| MT-CO3 | -0.6304 | 14.23535 | -8.8502 | 1.07E-15 | 1.60E-13 | 25.1497 |
| NEGR1 | -0.6303 | 3.736781 | -3.5691 | 0.00047 | 0.001459 | -0.6725 |
| CHRM4 | -0.6303 | 1.90971 | -4.6275 | 7.29E-06 | 3.73E-05 | 3.23852 |
| CASQ1 | -0.6302 | 3.765485 | -4.1341 | 5.57E-05 | 0.000223 | 1.31269 |
| RASD2 | -0.6295 | 2.413298 | -3.8182 | 0.00019 | 0.000654 | 0.17265 |
| SLCO1A2 | -0.6288 | 3.840788 | -3.078 | 0.00243 | 0.0062 | -2.1926 |
| PDE1A | -0.6274 | 3.037905 | -3.6959 | 0.00029 | 0.000975 | -0.2482 |
| RNF175 | -0.626 | 2.492928 | -3.8178 | 0.00019 | 0.000655 | 0.1712 |
| AC068987.1 | -0.6257 | 1.749948 | -4.6976 | 5.39E-06 | 2.87E-05 | 3.52604 |
| LHPP | -0.6242 | 6.235385 | -3.5242 | 0.00055 | 0.001669 | -0.8194 |
| MYRIP | -0.6235 | 2.84998 | -4.5613 | 9.66E-06 | 4.76E-05 | 2.97037 |
| LHX6 | -0.6235 | 1.479858 | -4.181 | 4.63E-05 | 0.00019 | 1.488 |
| LYVE1 | -0.6227 | 2.90087 | -3.24 | 0.00144 | 0.003904 | -1.713 |
| HCN2 | -0.6225 | 4.599066 | -4.1263 | 5.75E-05 | 0.000229 | 1.28346 |
| KRTAP5-AS1 | -0.6224 | 1.252079 | -4.6704 | 6.06E-06 | 3.17E-05 | 3.41386 |
| SHISA9 | -0.6224 | 2.903245 | -3.1608 | 0.00186 | 0.004893 | -1.9504 |
| CRY2 | -0.6209 | 4.988714 | -5.7873 | 3.34E-08 | 3.48E-07 | 8.39565 |
| MAP7 | -0.6209 | 4.111161 | -3.4907 | 0.00061 | 0.00185 | -0.9281 |
| DOC2B | -0.6203 | 1.636341 | -4.4598 | 1.48E-05 | 6.95E-05 | 2.56478 |
| CNKSR2 | -0.6186 | 3.293044 | -4.1789 | 4.67E-05 | 0.000191 | 1.48033 |
| RP11-14N7.2 | -0.6167 | 3.947753 | -3.3069 | 0.00115 | 0.003208 | -1.5087 |
| RP11-275H4.1 | -0.6167 | 1.471135 | -3.4935 | 0.00061 | 0.001836 | -0.9192 |
| MT-CO2 | -0.6156 | 14.21691 | -8.7795 | 1.65E-15 | 2.30E-13 | 24.7272 |
| SLC1A4 | -0.6155 | 5.606783 | -4.2536 | 3.46E-05 | 0.000147 | 1.76311 |
| PWAR6 | -0.6139 | 4.060323 | -4.3387 | 2.45E-05 | 0.000108 | 2.09035 |
| SSTR1 | -0.6138 | 1.965759 | -3.3721 | 0.00092 | 0.002646 | -1.306 |
| GABRA3 | -0.613 | 3.509388 | -3.1042 | 0.00223 | 0.00575 | -2.1165 |
| HTR5A-AS1 | -0.6127 | 0.767656 | -4.8743 | 2.48E-06 | 1.45E-05 | 4.26499 |
| MT3 | -0.6119 | 9.377603 | -3.2232 | 0.00152 | 0.0041 | -1.7637 |
| LINC00844 | -0.6116 | 6.708219 | -2.8681 | 0.00465 | 0.01092 | -2.7813 |
| KB-1517D11.4 | -0.611 | 0.641864 | -5.3413 | 2.91E-07 | 2.24E-06 | 6.31511 |
| RALYL | -0.6108 | 2.771694 | -3.1511 | 0.00192 | 0.00503 | -1.979 |
| 4-Sep | -0.6101 | 7.214252 | -3.1112 | 0.00218 | 0.005643 | -2.0963 |
| LINC00957 | -0.6098 | 2.276424 | -3.6449 | 0.00035 | 0.001147 | -0.4203 |
| ZNF385B | -0.6096 | 1.466757 | -4.354 | 2.30E-05 | 0.000102 | 2.14941 |
| SCN2A | -0.6096 | 3.10106 | -3.7569 | 0.00024 | 0.0008 | -0.0398 |
| PDE1B | -0.6093 | 2.922425 | -3.8742 | 0.00015 | 0.000542 | 0.3691 |
| SNPH | -0.6086 | 3.710465 | -5.1814 | 6.15E-07 | 4.27E-06 | 5.59764 |
| CRHR1 | -0.6085 | 1.82546 | -5.1764 | 6.30E-07 | 4.36E-06 | 5.57549 |
| TMEM191A | -0.6076 | 1.57914 | -4.3616 | 2.23E-05 | 9.96E-05 | 2.17926 |
| ANKRD24 | -0.6076 | 2.653549 | -5.7293 | 4.46E-08 | 4.42E-07 | 8.1187 |
| FA2H | -0.6076 | 3.919437 | -2.5802 | 0.01071 | 0.022497 | -3.5271 |
| NRG3 | -0.6071 | 3.558415 | -3.9542 | 0.00011 | 0.000414 | 0.65405 |
| ATP9A | -0.6066 | 6.133852 | -5.1677 | 6.56E-07 | 4.52E-06 | 5.53679 |
| PRRT2 | -0.6064 | 4.761767 | -4.151 | 5.21E-05 | 0.000211 | 1.37566 |
| CACNB1 | -0.6063 | 4.082925 | -5.6236 | 7.50E-08 | 6.93E-07 | 7.61856 |
| RP3-395M20.12 | -0.6056 | 1.62462 | -3.6844 | 0.00031 | 0.001012 | -0.2872 |
| FBLL1 | -0.6056 | 3.797072 | -3.7827 | 0.00021 | 0.000735 | 0.04931 |
| MARVELD3 | -0.6056 | 2.193256 | -3.086 | 0.00237 | 0.006064 | -2.1697 |
| CEP170B | -0.6054 | 3.441523 | -5.3749 | 2.48E-07 | 1.95E-06 | 6.46774 |
| PABPC1L2A | -0.6054 | 0.945026 | -4.6887 | 5.60E-06 | 2.96E-05 | 3.48944 |
| ARRB1 | -0.6039 | 4.461381 | -4.6824 | 5.75E-06 | 3.03E-05 | 3.46338 |
| PEG3 | -0.6039 | 4.26949 | -2.9906 | 0.0032 | 0.007868 | -2.4424 |
| NKAIN2 | -0.6028 | 3.334599 | -2.6955 | 0.00773 | 0.016986 | -3.237 |
| MYBPC1 | -0.6022 | 3.689233 | -2.8735 | 0.00458 | 0.010763 | -2.7669 |
| REEP6 | -0.6018 | 3.738286 | -5.446 | 1.77E-07 | 1.47E-06 | 6.7931 |
| SHISA7 | -0.6017 | 3.103435 | -3.6711 | 0.00032 | 0.001056 | -0.3323 |
| EPHA10 | -0.6016 | 1.70694 | -4.5678 | 9.40E-06 | 4.65E-05 | 2.99646 |
| ASPDH | -0.6007 | 3.309116 | -3.7738 | 0.00022 | 0.000756 | 0.0184 |
| CTD-2081K17.2 | -0.6003 | 2.24979 | -2.7491 | 0.00662 | 0.01485 | -3.0983 |
| MRAP2 | -0.5997 | 1.894444 | -3.2837 | 0.00124 | 0.003436 | -1.5799 |
| C2orf82 | -0.5993 | 3.59522 | -2.5163 | 0.01278 | 0.026176 | -3.6829 |
| AATK | -0.5981 | 5.166714 | -3.15 | 0.00193 | 0.005047 | -1.9824 |
| PYGM | -0.5976 | 2.649758 | -3.4869 | 0.00062 | 0.001872 | -0.9406 |
| CSMD3 | -0.5967 | 2.726046 | -3.3607 | 0.00096 | 0.002737 | -1.3416 |
| KCNJ9 | -0.5962 | 4.38409 | -3.5608 | 0.00048 | 0.001497 | -0.6996 |
| LINC00507 | -0.596 | 0.585549 | -4.1376 | 5.50E-05 | 0.000221 | 1.32552 |
| GLS2 | -0.5958 | 2.149428 | -4.267 | 3.28E-05 | 0.00014 | 1.81425 |
| RP11-307B6.3 | -0.5949 | 1.76796 | -3.2944 | 0.0012 | 0.003331 | -1.5471 |
| ELFN2 | -0.5948 | 3.649352 | -2.9568 | 0.00355 | 0.008631 | -2.5371 |
| CHL1 | -0.5944 | 4.99424 | -2.9002 | 0.00422 | 0.010021 | -2.6939 |
| RP11-82C23.2 | -0.5937 | 1.501812 | -2.5236 | 0.01253 | 0.025754 | -3.6652 |
| NT5C1A | -0.5936 | 1.478437 | -4.8922 | 2.29E-06 | 1.35E-05 | 4.34117 |
| DNMBP-AS1 | -0.5935 | 1.020653 | -4.8545 | 2.71E-06 | 1.57E-05 | 4.18106 |
| LINC00966 | -0.5932 | 2.239189 | -3.5568 | 0.00049 | 0.001514 | -0.7128 |
| RP11-826N14.2 | -0.5924 | 0.619207 | -5.7849 | 3.38E-08 | 3.51E-07 | 8.38439 |
| ZNF204P | -0.5914 | 2.281381 | -3.6973 | 0.00029 | 0.000971 | -0.2436 |
| GABBR2 | -0.5911 | 4.597393 | -2.8038 | 0.00564 | 0.012912 | -2.9543 |
| CTD-2210P24.4 | -0.5901 | 2.058799 | -4.5878 | 8.63E-06 | 4.33E-05 | 3.0774 |
| MYL3 | -0.5899 | 2.396983 | -4.4776 | 1.38E-05 | 6.51E-05 | 2.63534 |
| SLC35F3 | -0.5898 | 1.215385 | -4.5554 | 9.91E-06 | 4.87E-05 | 2.94644 |
| SPTBN2 | -0.5898 | 5.049521 | -3.7831 | 0.00021 | 0.000734 | 0.05078 |
| EIF4E1B | -0.5888 | 0.840958 | -5.1837 | 6.09E-07 | 4.23E-06 | 5.60776 |
| SPOCK1 | -0.5885 | 5.832055 | -3.4816 | 0.00063 | 0.001902 | -0.9576 |
| BAI3 | -0.588 | 4.665126 | -4.6039 | 8.06E-06 | 4.08E-05 | 3.14244 |
| GREM2 | -0.588 | 0.867679 | -4.878 | 2.44E-06 | 1.43E-05 | 4.28065 |
| MT-ND3 | -0.5878 | 13.72719 | -6.6601 | 3.59E-10 | 7.13E-09 | 12.7765 |
| C3orf80 | -0.5877 | 1.187369 | -3.8876 | 0.00014 | 0.000518 | 0.41653 |
| IPCEF1 | -0.5875 | 2.363002 | -3.8292 | 0.00018 | 0.000631 | 0.21093 |
| RP1-269M15.3 | -0.5867 | 0.893758 | -4.6007 | 8.17E-06 | 4.13E-05 | 3.12962 |
| NRXN1 | -0.5859 | 5.670885 | -3.4022 | 0.00083 | 0.002419 | -1.211 |
| RP1-63M2.6 | -0.5854 | 0.943917 | -3.4759 | 0.00065 | 0.001937 | -0.976 |
| HAR1B | -0.5852 | 0.85224 | -6.4105 | 1.36E-09 | 2.22E-08 | 11.485 |
| AP3B2 | -0.5843 | 4.693726 | -4.2725 | 3.20E-05 | 0.000137 | 1.83525 |
| THRB | -0.5826 | 3.232334 | -4.4622 | 1.47E-05 | 6.90E-05 | 2.57396 |
| CHRNA7 | -0.5825 | 1.622427 | -5.0626 | 1.06E-06 | 6.88E-06 | 5.07498 |
| IQSEC2 | -0.5819 | 3.851083 | -5.6885 | 5.45E-08 | 5.27E-07 | 7.92482 |
| DOK6 | -0.5818 | 2.606365 | -4.0361 | 8.19E-05 | 0.000313 | 0.95078 |
| TSPAN7 | -0.5817 | 8.41225 | -4.9668 | 1.64E-06 | 1.01E-05 | 4.65999 |
| ATP1B1 | -0.58 | 6.861746 | -3.2075 | 0.0016 | 0.004283 | -1.811 |
| KCNAB1 | -0.5799 | 2.877624 | -4.0051 | 9.23E-05 | 0.000348 | 0.83801 |
| CORT | -0.5792 | 1.824876 | -3.7369 | 0.00025 | 0.000854 | -0.1086 |
| STEAP2 | -0.5791 | 2.135924 | -4.5257 | 1.12E-05 | 5.44E-05 | 2.82723 |
| MYH7B | -0.5783 | 1.938313 | -5.9046 | 1.86E-08 | 2.11E-07 | 8.96143 |
| WNK2 | -0.5772 | 3.623329 | -3.0284 | 0.00284 | 0.007103 | -2.3353 |
| IL34 | -0.5762 | 2.72054 | -4.4067 | 1.85E-05 | 8.43E-05 | 2.35535 |
| RIMS4 | -0.5754 | 3.478332 | -3.3434 | 0.00102 | 0.002883 | -1.3957 |
| RHBDL3 | -0.5746 | 4.51061 | -2.9608 | 0.0035 | 0.008537 | -2.526 |
| GABRA4 | -0.574 | 1.383748 | -3.685 | 0.00031 | 0.00101 | -0.2851 |
| GUCY1B3 | -0.5732 | 3.883803 | -4.0046 | 9.25E-05 | 0.000348 | 0.83616 |
| RP11-588K22.2 | -0.572 | 2.724907 | -4.066 | 7.29E-05 | 0.000283 | 1.06047 |
| CHL1-AS2 | -0.5719 | 1.944889 | -3.6303 | 0.00037 | 0.001202 | -0.4692 |
| CNRIP1 | -0.5717 | 5.995508 | -4.7755 | 3.84E-06 | 2.13E-05 | 3.84911 |
| RP11-439C15.4 | -0.5716 | 3.385468 | -3.1065 | 0.00222 | 0.005714 | -2.1099 |
| VAMP2 | -0.5714 | 7.496536 | -5.9147 | 1.77E-08 | 2.01E-07 | 9.01067 |
| BOK | -0.5697 | 3.831678 | -2.4796 | 0.01412 | 0.028506 | -3.7705 |
| PAK6 | -0.5693 | 2.165539 | -3.4072 | 0.00082 | 0.002385 | -1.1952 |
| TMEM63C | -0.5689 | 2.921626 | -4.4011 | 1.89E-05 | 8.60E-05 | 2.33338 |
| LINC00672 | -0.5688 | 3.545562 | -3.3866 | 0.00088 | 0.002533 | -1.2603 |
| DOCK3 | -0.5687 | 3.329608 | -4.9809 | 1.54E-06 | 9.49E-06 | 4.72088 |
| PCBP3 | -0.5686 | 3.345148 | -4.0321 | 8.32E-05 | 0.000318 | 0.93629 |
| HSD11B1 | -0.566 | 1.820118 | -3.6673 | 0.00033 | 0.001069 | -0.3452 |
| SPRN | -0.5654 | 2.861058 | -4.8176 | 3.19E-06 | 1.81E-05 | 4.0254 |
| CACNA2D2 | -0.5643 | 1.994392 | -5.002 | 1.40E-06 | 8.74E-06 | 4.81169 |
| CTD-3193O13.9 | -0.564 | 3.552162 | -3.6572 | 0.00034 | 0.001103 | -0.3789 |
| CNTFR | -0.5637 | 5.309162 | -3.1998 | 0.00164 | 0.004379 | -1.834 |
| ACSBG1 | -0.5637 | 5.204777 | -3.3888 | 0.00087 | 0.002517 | -1.2533 |
| PCDHGA3 | -0.5636 | 2.221976 | -3.171 | 0.0018 | 0.004755 | -1.9199 |
| STXBP5-AS1 | -0.5636 | 1.370077 | -5.2853 | 3.79E-07 | 2.81E-06 | 6.06183 |
| CD300LG | -0.5635 | 0.874762 | -4.184 | 4.57E-05 | 0.000188 | 1.49933 |
| LRRC73 | -0.5633 | 1.673569 | -4.7922 | 3.57E-06 | 2.00E-05 | 3.91901 |
| RNF144A-AS1 | -0.5628 | 1.05225 | -4.4377 | 1.63E-05 | 7.53E-05 | 2.47739 |
| TCERG1L | -0.5624 | 0.87003 | -4.5722 | 9.23E-06 | 4.58E-05 | 3.01398 |
| LDHD | -0.5611 | 3.453682 | -5.3662 | 2.59E-07 | 2.02E-06 | 6.42826 |
| RP11-571M6.8 | -0.5609 | 2.815049 | -3.1973 | 0.00165 | 0.004411 | -1.8416 |
| PELI3 | -0.5604 | 3.63011 | -6.1904 | 4.32E-09 | 6.00E-08 | 10.3709 |
| MTMR7 | -0.56 | 2.929371 | -4.1545 | 5.14E-05 | 0.000208 | 1.38885 |
| BEX1 | -0.5595 | 7.980304 | -3.3999 | 0.00084 | 0.002435 | -1.2182 |
| C1QL2 | -0.5595 | 1.585065 | -3.5036 | 0.00059 | 0.001781 | -0.8865 |
| DNAJC6 | -0.5589 | 5.01788 | -3.8929 | 0.00014 | 0.00051 | 0.43537 |
| GPC5 | -0.5587 | 2.335594 | -3.5538 | 0.00049 | 0.001528 | -0.7227 |
| PRKCZ | -0.5582 | 5.06598 | -3.5959 | 0.00042 | 0.001341 | -0.5838 |
| RP11-809C18.3 | -0.5582 | 1.349303 | -3.2758 | 0.00128 | 0.003515 | -1.6041 |
| SLITRK1 | -0.5581 | 2.828264 | -3.292 | 0.00121 | 0.003353 | -1.5546 |
| SCN8A | -0.5578 | 2.627158 | -4.2883 | 3.00E-05 | 0.00013 | 1.89572 |
| GRM5-AS1 | -0.5575 | 1.169584 | -3.2485 | 0.0014 | 0.00381 | -1.6873 |
| ALDH5A1 | -0.5572 | 5.188278 | -5.0124 | 1.33E-06 | 8.39E-06 | 4.85689 |
| RP11-100M12.3 | -0.557 | 0.606637 | -5.4765 | 1.53E-07 | 1.29E-06 | 6.9335 |
| GRM2 | -0.5568 | 1.657244 | -3.5287 | 0.00054 | 0.001648 | -0.8049 |
| CARTPT | -0.5565 | 0.862803 | -3.2462 | 0.00141 | 0.003835 | -1.6943 |
| CLDN10 | -0.5552 | 3.544387 | -2.3611 | 0.01935 | 0.037403 | -4.0458 |
| LINC00641 | -0.5544 | 4.347481 | -5.5596 | 1.02E-07 | 9.06E-07 | 7.31908 |
| BSCL2 | -0.5542 | 5.805225 | -3.1996 | 0.00164 | 0.004382 | -1.8347 |
| GFOD1 | -0.5539 | 2.670379 | -4.5072 | 1.21E-05 | 5.83E-05 | 2.75327 |
| VAMP1 | -0.5532 | 4.178383 | -3.9334 | 0.00012 | 0.000444 | 0.57971 |
| PDZD2 | -0.5525 | 3.737645 | -4.2096 | 4.13E-05 | 0.000171 | 1.59609 |
| PCDH7 | -0.5524 | 3.832745 | -3.3617 | 0.00096 | 0.002729 | -1.3384 |
| CDR1 | -0.5523 | 5.317889 | -2.5741 | 0.0109 | 0.022833 | -3.5421 |
| PNOC | -0.5522 | 1.660286 | -2.3562 | 0.0196 | 0.037816 | -4.0569 |
| SCN1B | -0.5521 | 4.726587 | -3.206 | 0.00161 | 0.004301 | -1.8156 |
| LZTS3 | -0.5516 | 4.929495 | -5.4126 | 2.08E-07 | 1.68E-06 | 6.63978 |
| KIAA1161 | -0.5504 | 4.18518 | -4.1497 | 5.24E-05 | 0.000211 | 1.37091 |
| IQSEC1 | -0.5503 | 4.707442 | -4.6451 | 6.76E-06 | 3.50E-05 | 3.31055 |
| LCNL1 | -0.5501 | 3.043849 | -3.018 | 0.00293 | 0.007311 | -2.3647 |
| RP11-143K11.5 | -0.5496 | 0.955498 | -5.3426 | 2.89E-07 | 2.23E-06 | 6.32102 |
| XKR7 | -0.5489 | 1.346918 | -3.723 | 0.00027 | 0.000893 | -0.1561 |
| ANK3 | -0.5484 | 3.911087 | -3.4359 | 0.00074 | 0.002187 | -1.1043 |
| GPR26 | -0.5484 | 0.576372 | -5.0514 | 1.12E-06 | 7.19E-06 | 5.02642 |
| ANXA3 | -0.5474 | 1.674801 | -4.4397 | 1.61E-05 | 7.48E-05 | 2.48536 |
| GALNTL5 | -0.5467 | 0.761091 | -4.8542 | 2.71E-06 | 1.57E-05 | 4.17992 |
| CDH9 | -0.5462 | 1.232408 | -3.6114 | 0.0004 | 0.001276 | -0.5322 |
| NSF | -0.5461 | 5.614489 | -4.0234 | 8.60E-05 | 0.000327 | 0.90473 |
| PPP3CA | -0.5451 | 5.541773 | -5.2804 | 3.88E-07 | 2.86E-06 | 6.04003 |
| SLC13A5 | -0.5445 | 1.23184 | -3.8578 | 0.00016 | 0.000572 | 0.3115 |
| ATP8A1 | -0.5441 | 4.556942 | -3.4543 | 0.0007 | 0.002069 | -1.0454 |
| RP11-127B20.2 | -0.5435 | 2.077335 | -4.086 | 6.74E-05 | 0.000264 | 1.13433 |
| CCNI2 | -0.5435 | 3.46305 | -2.7181 | 0.00724 | 0.016068 | -3.179 |
| CTD-3199J23.4 | -0.5432 | 3.750824 | -2.9536 | 0.00358 | 0.008704 | -2.546 |
| TRHDE-AS1 | -0.5432 | 0.997224 | -4.5797 | 8.94E-06 | 4.45E-05 | 3.0446 |
| CTD-2184D3.6 | -0.5424 | 0.924442 | -4.9432 | 1.82E-06 | 1.10E-05 | 4.5588 |
| STMN3 | -0.5421 | 7.098741 | -5.3029 | 3.49E-07 | 2.62E-06 | 6.14149 |
| REPS2 | -0.542 | 3.444009 | -3.6877 | 0.0003 | 0.001002 | -0.2761 |
| SEZ6 | -0.5418 | 5.37594 | -2.9583 | 0.00353 | 0.008598 | -2.5331 |
| PCDHGA9 | -0.5418 | 2.730794 | -2.279 | 0.0239 | 0.044946 | -4.229 |
| DIO2 | -0.5417 | 3.121176 | -2.94 | 0.00374 | 0.009017 | -2.5839 |
| C2CD4C | -0.5414 | 2.369286 | -4.2193 | 3.97E-05 | 0.000165 | 1.63273 |
| NAP1L3 | -0.5413 | 5.625472 | -3.8919 | 0.00014 | 0.000511 | 0.43178 |
| KIF6 | -0.541 | 2.655743 | -2.8004 | 0.00569 | 0.013022 | -2.9631 |
| SLITRK5 | -0.5407 | 3.005297 | -4.017 | 8.82E-05 | 0.000334 | 0.88139 |
| DGKZ | -0.5406 | 5.815142 | -4.6777 | 5.87E-06 | 3.09E-05 | 3.44413 |
| LRP4 | -0.5395 | 5.893148 | -4.0606 | 7.44E-05 | 0.000288 | 1.04085 |
| AC140481.7 | -0.5389 | 1.253328 | -3.1582 | 0.00188 | 0.00493 | -1.9581 |
| SRCIN1 | -0.5389 | 4.199002 | -3.0752 | 0.00245 | 0.006247 | -2.2009 |
| GNAO1 | -0.5383 | 6.555464 | -4.6324 | 7.14E-06 | 3.67E-05 | 3.25854 |
| LINC00982 | -0.5375 | 2.461458 | -3.5425 | 0.00051 | 0.001581 | -0.7599 |
| SPRYD3 | -0.5367 | 5.663308 | -5.447 | 1.76E-07 | 1.46E-06 | 6.79743 |
| AP003039.3 | -0.5359 | 1.721598 | -3.0086 | 0.00302 | 0.007502 | -2.3916 |
| STAT4 | -0.5357 | 1.22075 | -4.0344 | 8.24E-05 | 0.000315 | 0.94469 |
| RP11-74E22.4 | -0.5343 | 1.495428 | -4.1312 | 5.64E-05 | 0.000226 | 1.30181 |
| EDIL3 | -0.5338 | 4.895461 | -2.4296 | 0.01615 | 0.031994 | -3.8882 |
| PHACTR1 | -0.5328 | 4.569719 | -4.1018 | 6.33E-05 | 0.00025 | 1.1925 |
| INPP5F | -0.5326 | 4.543614 | -4.0758 | 7.01E-05 | 0.000273 | 1.09661 |
| PDE4A | -0.5326 | 3.473262 | -4.7953 | 3.52E-06 | 1.97E-05 | 3.93202 |
| PDIA2 | -0.5325 | 3.44451 | -3.1828 | 0.00173 | 0.004598 | -1.8851 |
| SNRPN | -0.5322 | 5.508824 | -4.9078 | 2.14E-06 | 1.27E-05 | 4.40764 |
| LGI1 | -0.532 | 3.18231 | -2.7168 | 0.00727 | 0.016112 | -3.1822 |
| TTPA | -0.5317 | 1.596612 | -4.0045 | 9.25E-05 | 0.000348 | 0.83594 |
| UNC80 | -0.5307 | 3.023161 | -4.0302 | 8.38E-05 | 0.00032 | 0.92922 |
| GALNT16 | -0.5304 | 4.18047 | -4.5079 | 1.21E-05 | 5.82E-05 | 2.75596 |
| SEMA6B | -0.5301 | 4.724226 | -3.6854 | 0.00031 | 0.001009 | -0.284 |
| PLEKHB1 | -0.5295 | 8.721017 | -3.6752 | 0.00032 | 0.001043 | -0.3185 |
| LINGO1 | -0.5294 | 6.000043 | -3.0755 | 0.00245 | 0.006242 | -2.2001 |
| GNG13 | -0.5291 | 0.73523 | -4.3506 | 2.33E-05 | 0.000104 | 2.13654 |
| RAB26 | -0.5283 | 3.04084 | -3.684 | 0.00031 | 0.001013 | -0.2885 |
| RP11-863P13.5 | -0.5279 | 0.65489 | -4.8864 | 2.35E-06 | 1.38E-05 | 4.31646 |
| DLG2 | -0.5276 | 4.145516 | -3.7497 | 0.00024 | 0.000819 | -0.0646 |
| ADRA1B | -0.5273 | 1.845572 | -3.5679 | 0.00047 | 0.001464 | -0.6764 |
| PARM1 | -0.5272 | 2.906378 | -2.9679 | 0.00343 | 0.008371 | -2.5061 |
| TUB | -0.5268 | 4.898297 | -3.7719 | 0.00022 | 0.000761 | 0.012 |
| KIAA1244 | -0.5262 | 3.194323 | -4.1582 | 5.07E-05 | 0.000205 | 1.40254 |
| MAP3K9 | -0.526 | 1.488877 | -4.3462 | 2.37E-05 | 0.000105 | 2.11937 |
| RP11-686F15.3 | -0.5259 | 0.916287 | -4.6673 | 6.14E-06 | 3.21E-05 | 3.40146 |
| DLG4 | -0.5257 | 5.967887 | -5.2264 | 4.99E-07 | 3.56E-06 | 5.79802 |
| RELL2 | -0.5256 | 2.878962 | -4.3368 | 2.47E-05 | 0.000109 | 2.08284 |
| PAQR8 | -0.5251 | 5.305572 | -3.0918 | 0.00232 | 0.005966 | -2.1527 |
| ANKRD29 | -0.525 | 2.016803 | -3.9357 | 0.00012 | 0.00044 | 0.58793 |
| LINC01018 | -0.5249 | 0.844226 | -4.8999 | 2.21E-06 | 1.31E-05 | 4.37392 |
| PCLO | -0.5241 | 1.95418 | -3.311 | 0.00113 | 0.003169 | -1.496 |
| SYT2 | -0.5238 | 0.763616 | -3.8621 | 0.00016 | 0.000564 | 0.32661 |
| DNAJA4 | -0.5235 | 3.819049 | -3.0186 | 0.00293 | 0.007299 | -2.363 |
| CADPS2 | -0.5235 | 3.009563 | -3.2607 | 0.00134 | 0.003676 | -1.6502 |
| KIAA1549L | -0.5233 | 3.234193 | -4.3511 | 2.33E-05 | 0.000103 | 2.13815 |
| PAQR6 | -0.5231 | 6.949248 | -3.1465 | 0.00195 | 0.005102 | -1.9927 |
| TRIM17 | -0.5223 | 1.966131 | -4.5871 | 8.66E-06 | 4.33E-05 | 3.07453 |
| FUT9 | -0.5223 | 3.288401 | -3.5725 | 0.00046 | 0.001443 | -0.6611 |
| NEBL | -0.5213 | 5.063613 | -3.501 | 0.00059 | 0.001795 | -0.8949 |
| FGF14 | -0.5208 | 2.912297 | -3.3316 | 0.00106 | 0.002987 | -1.4324 |
| SYNPO | -0.5207 | 4.48081 | -2.2406 | 0.02634 | 0.048888 | -4.3128 |
| KCNK12 | -0.5206 | 1.529929 | -3.8713 | 0.00015 | 0.000547 | 0.35903 |
| RP3-406A7.7 | -0.5201 | 2.04877 | -3.0747 | 0.00245 | 0.006252 | -2.2023 |
| RP11-389G6.3 | -0.52 | 0.773316 | -4.2343 | 3.74E-05 | 0.000157 | 1.68971 |
| YJEFN3 | -0.52 | 3.906189 | -3.647 | 0.00035 | 0.00114 | -0.4134 |
| PDYN | -0.5199 | 1.672349 | -2.2846 | 0.02356 | 0.044378 | -4.2167 |
| RND1 | -0.5193 | 4.207512 | -3.6131 | 0.0004 | 0.001269 | -0.5265 |
| SERP2 | -0.5185 | 5.119348 | -4.3819 | 2.05E-05 | 9.23E-05 | 2.25843 |
| GPR61 | -0.5184 | 1.569514 | -4.4479 | 1.56E-05 | 7.27E-05 | 2.51768 |
| SLC7A4 | -0.5177 | 0.922857 | -4.8336 | 2.97E-06 | 1.70E-05 | 4.09278 |
| LINC00943 | -0.5167 | 1.219722 | -4.0201 | 8.71E-05 | 0.000331 | 0.89259 |
| CPEB3 | -0.5166 | 2.440205 | -5.2196 | 5.15E-07 | 3.65E-06 | 5.76779 |
| MAP1LC3A | -0.5162 | 5.690803 | -2.9011 | 0.00421 | 0.01 | -2.6914 |
| CAMK2G | -0.5157 | 5.917931 | -4.2257 | 3.87E-05 | 0.000162 | 1.65689 |
| AJAP1 | -0.5145 | 1.918794 | -4.0993 | 6.39E-05 | 0.000252 | 1.18347 |
| STXBP5L | -0.5139 | 1.823781 | -3.4617 | 0.00068 | 0.002022 | -1.0216 |
| SNTA1 | -0.5139 | 4.990143 | -3.4182 | 0.00079 | 0.002308 | -1.1606 |
| RGS6 | -0.513 | 2.502235 | -3.0106 | 0.003 | 0.007459 | -2.3859 |
| CRH | -0.5124 | 0.761579 | -4.3077 | 2.78E-05 | 0.000121 | 1.97047 |
| PHYHD1 | -0.5123 | 4.521462 | -3.2716 | 0.00129 | 0.00356 | -1.617 |
| YPEL4 | -0.5123 | 3.815935 | -3.7171 | 0.00027 | 0.00091 | -0.1761 |
| BASP1 | -0.5122 | 6.619029 | -3.2667 | 0.00132 | 0.003612 | -1.632 |
| TUBG2 | -0.5121 | 5.805939 | -8.0097 | 1.71E-13 | 1.04E-11 | 20.2101 |
| RP11-122K13.14 | -0.512 | 0.673255 | -4.3656 | 2.19E-05 | 9.81E-05 | 2.19489 |
| APOD | -0.512 | 7.726228 | -2.9005 | 0.00422 | 0.010012 | -2.693 |
| CPNE4 | -0.5118 | 2.447575 | -2.5205 | 0.01264 | 0.025933 | -3.6728 |
| U82695.10 | -0.5115 | 1.558615 | -5.2906 | 3.70E-07 | 2.75E-06 | 6.08571 |
| P2RX5 | -0.5115 | 1.755728 | -3.4885 | 0.00062 | 0.001863 | -0.9355 |
| TRNP1 | -0.5112 | 3.275119 | -3.5216 | 0.00055 | 0.001682 | -0.8278 |
| AC004019.13 | -0.5112 | 1.15438 | -4.5237 | 1.13E-05 | 5.49E-05 | 2.81913 |
| ABHD8 | -0.5111 | 4.406735 | -4.5457 | 1.03E-05 | 5.05E-05 | 2.90757 |
| RP11-379F4.4 | -0.511 | 1.223067 | -4.2669 | 3.28E-05 | 0.00014 | 1.81382 |
| GATSL3 | -0.5109 | 4.30708 | -3.4915 | 0.00061 | 0.001846 | -0.9256 |
| SYT12 | -0.5098 | 2.525347 | -3.832 | 0.00018 | 0.000625 | 0.22082 |
| KCNIP3 | -0.5094 | 5.112217 | -2.754 | 0.00653 | 0.014674 | -3.0857 |
| RP11-82L18.4 | -0.5092 | 3.869108 | -2.3875 | 0.01806 | 0.035267 | -3.9857 |
| NR1D1 | -0.509 | 3.93288 | -5.224 | 5.05E-07 | 3.59E-06 | 5.78747 |
| CTD-2126E3.4 | -0.5074 | 1.732804 | -4.3813 | 2.05E-05 | 9.26E-05 | 2.25586 |
| ZNF365 | -0.5073 | 3.512491 | -3.8339 | 0.00018 | 0.000621 | 0.2275 |
| MCF2 | -0.5073 | 2.037385 | -3.5244 | 0.00054 | 0.001669 | -0.819 |
| DGCR10 | -0.5066 | 0.996556 | -5.0659 | 1.05E-06 | 6.80E-06 | 5.08966 |
| DIRAS1 | -0.5061 | 4.475275 | -3.7471 | 0.00024 | 0.000826 | -0.0734 |
| PRKAG2-AS1 | -0.5061 | 2.484143 | -3.7632 | 0.00023 | 0.000784 | -0.018 |
| PPFIA3 | -0.5055 | 4.022997 | -4.8107 | 3.29E-06 | 1.85E-05 | 3.99669 |
| LCN12 | -0.5048 | 2.449777 | -3.5306 | 0.00053 | 0.001639 | -0.7986 |
| RP11-690D19.3 | -0.5047 | 2.363741 | -6.0186 | 1.04E-08 | 1.28E-07 | 9.51837 |
| FSTL4 | -0.5045 | 1.293375 | -3.9738 | 0.0001 | 0.000387 | 0.72472 |
| CALM3 | -0.5042 | 8.492119 | -4.2912 | 2.97E-05 | 0.000129 | 1.90702 |
| LDOC1 | -0.5038 | 5.412872 | -4.4039 | 1.87E-05 | 8.52E-05 | 2.3445 |
| ITPR1 | -0.5031 | 3.24694 | -3.0003 | 0.0031 | 0.00767 | -2.4151 |
| FN3K | -0.5028 | 5.372357 | -5.6473 | 6.67E-08 | 6.27E-07 | 7.73032 |
| ANO5 | -0.5027 | 2.067686 | -4.029 | 8.42E-05 | 0.000321 | 0.92504 |
| RAB6B | -0.502 | 6.015824 | -4.2095 | 4.13E-05 | 0.000171 | 1.59545 |
| BRSK2 | -0.5017 | 4.898284 | -3.5 | 0.00059 | 0.0018 | -0.8983 |
| CBFA2T3 | -0.5014 | 2.011926 | -4.935 | 1.89E-06 | 1.14E-05 | 4.52377 |
| VSTM2B | -0.5009 | 3.822238 | -2.7866 | 0.00593 | 0.013494 | -2.9999 |
| RP1-239B22.5 | -0.5007 | 1.517476 | -4.5458 | 1.03E-05 | 5.05E-05 | 2.9079 |
| MGAT4C | -0.5006 | 2.815441 | -2.6538 | 0.00871 | 0.018826 | -3.3433 |
| CDK5R1 | -0.5004 | 4.799835 | -3.0129 | 0.00298 | 0.007411 | -2.3793 |
