# Supplementary material for: MGME1 associates with poor prognosis and is vital for cell proliferation in lower-grade glioma
Source: Aging (Albany NY). 2023 May 8;15(9):3690–714. doi: 10.18632/aging.204705 (PMC10449294; doi:10.18632/aging.204705)
Supplement: Supplementary Table 4 [file aging-15-204705-s005.docx]

**Supplementary Table 4. Up-regulated DEGs in CGGA dataset.**

| **id** | **logFC** | **AveExpr** | **t** | **P.Value** | **adj.P.Vl** | **B** |
| --- | --- | --- | --- | --- | --- | --- |
| SEC22B | 0.50008 | 3.797124 | 7.84894 | 4.39E-13 | 2.27E-11 | 19.2895 |
| HIST2H2BB | 0.50008 | 1.849738 | 3.60984 | 0.0004 | 0.001283 | -0.5375 |
| NANP | 0.50011 | 1.701103 | 7.77238 | 6.87E-13 | 3.39E-11 | 18.854 |
| CCDC146 | 0.50014 | 2.821101 | 3.48989 | 0.00061 | 0.001855 | -0.9309 |
| COMMD8 | 0.50015 | 3.601103 | 5.72939 | 4.46E-08 | 4.42E-07 | 8.11909 |
| FZD1 | 0.50026 | 2.799616 | 5.42692 | 1.94E-07 | 1.59E-06 | 6.70546 |
| AAED1 | 0.50082 | 2.875427 | 6.56108 | 6.12E-10 | 1.12E-08 | 12.2604 |
| CTDSPL2 | 0.50088 | 3.869287 | 5.58983 | 8.84E-08 | 7.97E-07 | 7.4603 |
| GPR1 | 0.50106 | 0.527396 | 4.50285 | 1.24E-05 | 5.92E-05 | 2.73578 |
| HIST1H2BN | 0.50122 | 1.269235 | 5.78992 | 3.30E-08 | 3.44E-07 | 8.40819 |
| MORC4 | 0.50146 | 2.852348 | 7.37549 | 6.77E-12 | 2.42E-10 | 16.6296 |
| GRIK3 | 0.50158 | 4.298988 | 3.23102 | 0.00148 | 0.004009 | -1.7402 |
| CLDN7 | 0.5016 | 1.348432 | 5.15707 | 6.89E-07 | 4.71E-06 | 5.48995 |
| RRP15 | 0.50166 | 2.889671 | 6.35493 | 1.83E-09 | 2.84E-08 | 11.2014 |
| RP11-344B5.2 | 0.50191 | 1.873438 | 3.69713 | 0.00029 | 0.000971 | -0.2441 |
| LSM10 | 0.50199 | 5.246871 | 6.69343 | 3.00E-10 | 6.08E-09 | 12.9509 |
| IL1R1 | 0.50205 | 2.181516 | 3.31293 | 0.00113 | 0.003152 | -1.49 |
| IRAK1 | 0.50221 | 5.617159 | 6.63805 | 4.05E-10 | 7.87E-09 | 12.661 |
| SLC37A3 | 0.50236 | 4.608005 | 7.48698 | 3.58E-12 | 1.38E-10 | 17.2487 |
| RAB34 | 0.50276 | 3.958371 | 2.95395 | 0.00358 | 0.008699 | -2.5452 |
| LIMD2 | 0.50286 | 4.298852 | 5.1349 | 7.63E-07 | 5.14E-06 | 5.39208 |
| PPM1D | 0.50287 | 3.342445 | 6.57514 | 5.68E-10 | 1.05E-08 | 12.3334 |
| IER2 | 0.50307 | 5.254292 | 3.52328 | 0.00055 | 0.001673 | -0.8225 |
| LIN9 | 0.5031 | 1.880519 | 7.13192 | 2.68E-11 | 7.89E-10 | 15.2938 |
| IRF9 | 0.50314 | 6.555652 | 5.21759 | 5.20E-07 | 3.69E-06 | 5.75869 |
| PHYKPL | 0.50323 | 4.723339 | 6.79138 | 1.76E-10 | 3.87E-09 | 13.467 |
| ADAM19 | 0.50327 | 1.901728 | 4.41186 | 1.81E-05 | 8.26E-05 | 2.37559 |
| ST7L | 0.50339 | 3.730254 | 8.07664 | 1.15E-13 | 7.29E-12 | 20.5962 |
| EYA3 | 0.50384 | 2.928268 | 7.01321 | 5.19E-11 | 1.40E-09 | 14.6514 |
| ARHGAP6 | 0.50384 | 2.41771 | 3.8206 | 0.00019 | 0.000649 | 0.181 |
| TAF1A | 0.50388 | 2.608632 | 6.85167 | 1.27E-10 | 2.95E-09 | 13.7868 |
| IER3 | 0.50394 | 3.582878 | 2.46063 | 0.01486 | 0.0298 | -3.8155 |
| CYP4F12 | 0.50394 | 1.426082 | 3.05246 | 0.00263 | 0.006641 | -2.2664 |
| ALPK2 | 0.5042 | 0.822565 | 3.16864 | 0.00181 | 0.004786 | -1.927 |
| ZNF789 | 0.50434 | 3.719215 | 6.613 | 4.63E-10 | 8.78E-09 | 12.5303 |
| PRKD1 | 0.50452 | 3.805929 | 4.89451 | 2.27E-06 | 1.34E-05 | 4.35082 |
| CLEC10A | 0.5047 | 0.880191 | 4.29948 | 2.87E-05 | 0.000125 | 1.93884 |
| KDELR3 | 0.50473 | 1.316439 | 4.12759 | 5.72E-05 | 0.000228 | 1.28832 |
| RCSD1 | 0.50478 | 3.486687 | 4.64027 | 6.90E-06 | 3.56E-05 | 3.29063 |
| COL4A6 | 0.50491 | 1.289094 | 4.42651 | 1.70E-05 | 7.84E-05 | 2.4332 |
| ARRDC4 | 0.5052 | 4.233493 | 3.80226 | 0.0002 | 0.000688 | 0.11711 |
| PLCB2 | 0.50521 | 4.162165 | 4.09808 | 6.43E-05 | 0.000253 | 1.17882 |
| LYPLAL1 | 0.50523 | 4.747415 | 7.46259 | 4.12E-12 | 1.57E-10 | 17.1128 |
| GCOM1 | 0.50526 | 0.997681 | 3.48111 | 0.00063 | 0.001905 | -0.9592 |
| RIPK1 | 0.50528 | 3.42281 | 6.9197 | 8.71E-11 | 2.15E-09 | 14.1495 |
| LZIC | 0.50529 | 4.791954 | 6.88541 | 1.05E-10 | 2.51E-09 | 13.9665 |
| UBTD2 | 0.50535 | 3.929249 | 6.6637 | 3.52E-10 | 7.01E-09 | 12.7951 |
| MRGBP | 0.50537 | 3.719205 | 9.23537 | 9.89E-17 | 2.83E-14 | 27.474 |
| ADAMTSL4 | 0.50542 | 1.479476 | 4.19486 | 4.38E-05 | 0.00018 | 1.54035 |
| EFCAB13 | 0.50552 | 1.032757 | 6.46933 | 9.98E-10 | 1.71E-08 | 11.7866 |
| RP11-750H9.5 | 0.50562 | 2.549888 | 3.71238 | 0.00028 | 0.000925 | -0.1923 |
| INTS9 | 0.50565 | 3.629358 | 8.46149 | 1.15E-14 | 1.11E-12 | 22.8409 |
| YAP1 | 0.50597 | 4.059967 | 3.36799 | 0.00094 | 0.002679 | -1.3188 |
| TLN1 | 0.50611 | 6.215908 | 5.73897 | 4.25E-08 | 4.24E-07 | 8.16469 |
| LILRB2 | 0.50616 | 1.643431 | 4.44287 | 1.59E-05 | 7.40E-05 | 2.49771 |
| LRRC8D | 0.5062 | 4.650449 | 6.77149 | 1.96E-10 | 4.26E-09 | 13.3619 |
| RBMS2 | 0.50632 | 2.985378 | 6.86765 | 1.16E-10 | 2.75E-09 | 13.8718 |
| CTB-25B13.12 | 0.50633 | 2.548174 | 4.91124 | 2.10E-06 | 1.25E-05 | 4.4221 |
| ALYREF | 0.50641 | 5.717307 | 6.63749 | 4.06E-10 | 7.89E-09 | 12.6581 |
| HAS3 | 0.50651 | 1.274392 | 5.89496 | 1.95E-08 | 2.19E-07 | 8.91472 |
| POLE3 | 0.50656 | 5.44607 | 9.00537 | 4.12E-16 | 8.08E-14 | 26.0821 |
| AC117395.1 | 0.50682 | 2.648109 | 4.43121 | 1.67E-05 | 7.71E-05 | 2.45172 |
| SCN7A | 0.50684 | 0.929958 | 3.6545 | 0.00034 | 0.001113 | -0.3882 |
| SNRPE | 0.50688 | 6.609773 | 4.79991 | 3.45E-06 | 1.94E-05 | 3.95134 |
| SEMA3F | 0.50689 | 1.746265 | 3.56322 | 0.00047 | 0.001487 | -0.6917 |
| NBPF15 | 0.5069 | 3.699947 | 5.65007 | 6.58E-08 | 6.20E-07 | 7.74331 |
| CBFB | 0.507 | 4.823752 | 5.93455 | 1.60E-08 | 1.85E-07 | 9.10722 |
| NR2F2 | 0.50705 | 3.166479 | 3.25433 | 0.00137 | 0.003748 | -1.6696 |
| LCK | 0.50732 | 1.1612 | 3.55597 | 0.00049 | 0.001518 | -0.7156 |
| VEZF1 | 0.50735 | 5.548559 | 5.72645 | 4.52E-08 | 4.47E-07 | 8.1051 |
| GABPB1 | 0.50748 | 3.235739 | 7.09009 | 3.39E-11 | 9.69E-10 | 15.0668 |
| FLT3LG | 0.50766 | 3.26314 | 5.47323 | 1.55E-07 | 1.31E-06 | 6.91845 |
| PPP1R8 | 0.5079 | 5.283108 | 8.0368 | 1.45E-13 | 8.93E-12 | 20.3663 |
| MYC | 0.50804 | 4.944932 | 3.15102 | 0.00192 | 0.005031 | -1.9792 |
| ZNF625-ZNF20 | 0.50807 | 1.81657 | 7.52506 | 2.88E-12 | 1.14E-10 | 17.4611 |
| ARHGAP9 | 0.50809 | 3.151086 | 4.37367 | 2.12E-05 | 9.52E-05 | 2.22618 |
| MRE11A | 0.5083 | 2.895168 | 7.91794 | 2.93E-13 | 1.65E-11 | 19.6837 |
| IGFBPL1 | 0.50833 | 1.64093 | 2.55563 | 0.01147 | 0.023852 | -3.5874 |
| TMCO4 | 0.5084 | 2.442076 | 4.80848 | 3.32E-06 | 1.87E-05 | 3.98729 |
| SH3BP5-AS1 | 0.50848 | 2.476973 | 4.85537 | 2.70E-06 | 1.56E-05 | 4.18482 |
| SOCS4 | 0.50851 | 3.249797 | 7.98843 | 1.93E-13 | 1.16E-11 | 20.088 |
| NUP85 | 0.50856 | 4.684479 | 8.38887 | 1.77E-14 | 1.59E-12 | 22.414 |
| MYSM1 | 0.50864 | 2.888069 | 6.85954 | 1.21E-10 | 2.85E-09 | 13.8287 |
| FABP5P7 | 0.50867 | 1.091377 | 2.52908 | 0.01234 | 0.025408 | -3.652 |
| HHEX | 0.50884 | 2.044162 | 4.88346 | 2.38E-06 | 1.40E-05 | 4.30388 |
| TMEM43 | 0.50888 | 5.02585 | 6.8341 | 1.39E-10 | 3.20E-09 | 13.6935 |
| AC004797.1 | 0.50888 | 3.347074 | 5.10698 | 8.67E-07 | 5.75E-06 | 5.26925 |
| RASAL3 | 0.50889 | 2.406638 | 4.2599 | 3.37E-05 | 0.000144 | 1.78715 |
| TWIST1 | 0.50901 | 1.449041 | 3.68945 | 0.0003 | 0.000996 | -0.2702 |
| TCEB1P19 | 0.50924 | 1.630038 | 4.58764 | 8.64E-06 | 4.33E-05 | 3.07659 |
| SH3PXD2B | 0.50935 | 3.860217 | 5.09107 | 9.33E-07 | 6.13E-06 | 5.1995 |
| FBXO4 | 0.50939 | 3.191981 | 6.39523 | 1.48E-09 | 2.38E-08 | 11.4068 |
| SLC35D1 | 0.50943 | 2.463213 | 6.61126 | 4.67E-10 | 8.86E-09 | 12.5213 |
| BZW1 | 0.50948 | 6.678261 | 7.96177 | 2.26E-13 | 1.32E-11 | 19.9349 |
| GSX2 | 0.50953 | 0.925639 | 3.11768 | 0.00214 | 0.00554 | -2.0773 |
| MYO1C | 0.50961 | 3.590313 | 5.69569 | 5.26E-08 | 5.10E-07 | 7.95897 |
| GEMIN6 | 0.50962 | 3.25936 | 8.30233 | 2.99E-14 | 2.43E-12 | 21.9073 |
| MTFR2 | 0.50984 | 1.035277 | 7.14711 | 2.46E-11 | 7.35E-10 | 15.3764 |
| GLB1 | 0.5099 | 4.371636 | 7.13876 | 2.58E-11 | 7.65E-10 | 15.331 |
| FGD2 | 0.50993 | 3.289729 | 3.96936 | 0.00011 | 0.000393 | 0.70872 |
| HSPBAP1 | 0.51034 | 2.918375 | 7.49172 | 3.49E-12 | 1.35E-10 | 17.275 |
| SPATS2L | 0.51035 | 5.474013 | 5.26266 | 4.21E-07 | 3.08E-06 | 5.9603 |
| APOBR | 0.51046 | 1.75788 | 4.83261 | 2.98E-06 | 1.71E-05 | 4.08875 |
| PRKCDBP | 0.51083 | 2.206519 | 3.63433 | 0.00037 | 0.001187 | -0.4558 |
| SNAP23 | 0.51084 | 5.083928 | 6.56016 | 6.15E-10 | 1.13E-08 | 12.2557 |
| RPL24P2 | 0.51088 | 1.878844 | 3.52467 | 0.00054 | 0.001667 | -0.818 |
| ZBTB33 | 0.51089 | 3.899765 | 7.66995 | 1.25E-12 | 5.58E-11 | 18.2745 |
| C1orf174 | 0.51095 | 3.682701 | 8.27513 | 3.52E-14 | 2.77E-12 | 21.7484 |
| RNF149 | 0.511 | 3.826444 | 4.99964 | 1.41E-06 | 8.81E-06 | 4.80166 |
| CDC42 | 0.51103 | 6.974199 | 7.08127 | 3.56E-11 | 1.01E-09 | 15.019 |
| WRAP73 | 0.51121 | 4.107456 | 7.40903 | 5.59E-12 | 2.06E-10 | 16.8153 |
| ENO3 | 0.51124 | 3.452476 | 5.12847 | 7.86E-07 | 5.28E-06 | 5.36378 |
| DUSP5 | 0.51131 | 2.082784 | 3.12786 | 0.00207 | 0.005382 | -2.0474 |
| ZNF644 | 0.51147 | 4.379427 | 7.57469 | 2.16E-12 | 8.95E-11 | 17.7389 |
| C8orf76 | 0.51176 | 4.024013 | 7.69793 | 1.06E-12 | 4.88E-11 | 18.4324 |
| TCF7L1 | 0.51185 | 3.879738 | 4.34143 | 2.42E-05 | 0.000107 | 2.10082 |
| SIPA1 | 0.51201 | 4.433893 | 4.65031 | 6.61E-06 | 3.43E-05 | 3.33168 |
| POLG2 | 0.51206 | 3.156492 | 6.73327 | 2.42E-10 | 5.07E-09 | 13.1603 |
| KLRC4 | 0.51211 | 1.999166 | 2.76033 | 0.0064 | 0.014437 | -3.069 |
| RNF19B | 0.51211 | 3.745094 | 5.57875 | 9.33E-08 | 8.36E-07 | 7.40848 |
| CHRNA1 | 0.5122 | 0.934301 | 3.32383 | 0.00109 | 0.003053 | -1.4563 |
| ATP6V0E1 | 0.51253 | 7.358804 | 6.31203 | 2.29E-09 | 3.47E-08 | 10.9836 |
| ACN9 | 0.51275 | 4.119057 | 4.42464 | 1.72E-05 | 7.88E-05 | 2.42583 |
| ELF1 | 0.51282 | 4.285014 | 6.25056 | 3.16E-09 | 4.60E-08 | 10.673 |
| DLGAP1-AS2 | 0.51301 | 1.819039 | 4.82329 | 3.11E-06 | 1.77E-05 | 4.0495 |
| EXTL2 | 0.51306 | 3.954814 | 6.21368 | 3.83E-09 | 5.41E-08 | 10.4877 |
| EXOSC3 | 0.51308 | 3.988493 | 8.65572 | 3.52E-15 | 4.18E-13 | 23.9897 |
| ZNF124 | 0.51314 | 2.28961 | 7.72791 | 8.91E-13 | 4.21E-11 | 18.602 |
| KNSTRN | 0.51319 | 3.648957 | 6.3331 | 2.05E-09 | 3.16E-08 | 11.0904 |
| HOXC9 | 0.51326 | 0.503542 | 4.05883 | 7.49E-05 | 0.00029 | 1.03419 |
| PROCR | 0.51328 | 2.598164 | 5.05568 | 1.10E-06 | 7.07E-06 | 5.04489 |
| RBM8A | 0.51338 | 6.518171 | 8.20921 | 5.21E-14 | 3.82E-12 | 21.3644 |
| TBC1D10C | 0.51347 | 1.393607 | 5.52001 | 1.24E-07 | 1.07E-06 | 7.13491 |
| COL18A1 | 0.51366 | 4.517108 | 3.54473 | 0.00051 | 0.001571 | -0.7524 |
| ASIP | 0.51386 | 1.22405 | 4.0861 | 6.73E-05 | 0.000264 | 1.13455 |
| SLPI | 0.51391 | 2.757856 | 2.35846 | 0.01948 | 0.037627 | -4.0518 |
| GALNT3 | 0.51406 | 1.178351 | 4.31121 | 2.74E-05 | 0.00012 | 1.98399 |
| FOXD3 | 0.51413 | 0.582204 | 4.86685 | 2.56E-06 | 1.49E-05 | 4.23342 |
| NCF1C | 0.51415 | 1.563306 | 4.33365 | 2.50E-05 | 0.00011 | 2.07068 |
| FIGNL1 | 0.51424 | 3.017629 | 7.03511 | 4.60E-11 | 1.26E-09 | 14.7695 |
| B4GALT1 | 0.51428 | 3.164777 | 4.60261 | 8.11E-06 | 4.10E-05 | 3.13729 |
| IFIT2 | 0.51449 | 4.576403 | 3.85756 | 0.00016 | 0.000572 | 0.31054 |
| PRR16 | 0.51455 | 1.45426 | 3.95491 | 0.00011 | 0.000413 | 0.65672 |
| NUP188 | 0.51462 | 4.536937 | 6.22938 | 3.53E-09 | 5.04E-08 | 10.5665 |
| RBM15 | 0.51464 | 2.205873 | 7.47698 | 3.79E-12 | 1.45E-10 | 17.1929 |
| PABPC4 | 0.51469 | 5.938814 | 7.64103 | 1.47E-12 | 6.46E-11 | 18.1116 |
| ZAP70 | 0.51473 | 1.39719 | 4.85587 | 2.69E-06 | 1.56E-05 | 4.18694 |
| ARSJ | 0.51478 | 1.564963 | 3.61296 | 0.0004 | 0.00127 | -0.5271 |
| RPL39 | 0.51497 | 10.40909 | 4.69879 | 5.36E-06 | 2.86E-05 | 3.53083 |
| SFPQ | 0.51507 | 7.587514 | 7.04805 | 4.28E-11 | 1.18E-09 | 14.8394 |
| TAF12 | 0.51556 | 4.034161 | 7.62291 | 1.64E-12 | 7.03E-11 | 18.0096 |
| KLRK1 | 0.51562 | 2.470801 | 3.00056 | 0.0031 | 0.007665 | -2.4143 |
| RBM7 | 0.51573 | 4.236568 | 6.80277 | 1.66E-10 | 3.68E-09 | 13.5273 |
| BTG1 | 0.51574 | 5.639436 | 6.068 | 8.11E-09 | 1.03E-07 | 9.76212 |
| HPS3 | 0.51576 | 3.706774 | 7.13968 | 2.57E-11 | 7.62E-10 | 15.336 |
| TBC1D1 | 0.51582 | 5.005198 | 4.43474 | 1.65E-05 | 7.61E-05 | 2.46564 |
| TONSL | 0.51588 | 1.915821 | 5.66668 | 6.07E-08 | 5.77E-07 | 7.8217 |
| MARS | 0.51594 | 6.48951 | 4.5048 | 1.23E-05 | 5.88E-05 | 2.74358 |
| ZNF3 | 0.51601 | 4.740262 | 6.02722 | 9.99E-09 | 1.23E-07 | 9.56101 |
| TRIP10 | 0.51603 | 3.923199 | 4.77034 | 3.92E-06 | 2.17E-05 | 3.82766 |
| GIMAP4 | 0.51607 | 4.226551 | 4.82098 | 3.14E-06 | 1.78E-05 | 4.03982 |
| OLFML2A | 0.51608 | 1.72751 | 3.83133 | 0.00018 | 0.000627 | 0.21849 |
| C19orf40 | 0.51617 | 1.375548 | 7.18506 | 1.99E-11 | 6.16E-10 | 15.5832 |
| RND3 | 0.51627 | 3.983359 | 3.36725 | 0.00094 | 0.002685 | -1.3211 |
| RP4-717I23.3 | 0.51628 | 4.096192 | 4.94825 | 1.78E-06 | 1.08E-05 | 4.58042 |
| LINC00998 | 0.51645 | 5.936259 | 5.77405 | 3.57E-08 | 3.67E-07 | 8.33221 |
| CCDC40 | 0.51648 | 2.781418 | 4.09505 | 6.50E-05 | 0.000255 | 1.16761 |
| AC007362.1 | 0.51654 | 3.003646 | 3.6708 | 0.00032 | 0.001057 | -0.3333 |
| ZFAND6 | 0.51656 | 6.044204 | 7.90848 | 3.10E-13 | 1.73E-11 | 19.6295 |
| MED30 | 0.51665 | 4.002164 | 7.26105 | 1.30E-11 | 4.27E-10 | 15.9991 |
| SLAMF7 | 0.51667 | 0.844522 | 5.2916 | 3.68E-07 | 2.74E-06 | 6.0904 |
| FUCA1 | 0.51676 | 3.707962 | 5.45787 | 1.67E-07 | 1.40E-06 | 6.84766 |
| ACTR3 | 0.51681 | 6.349234 | 6.45499 | 1.08E-09 | 1.82E-08 | 11.7128 |
| HOXB3 | 0.517 | 0.770633 | 2.97993 | 0.0033 | 0.0081 | -2.4724 |
| CCNL1 | 0.51706 | 5.47766 | 6.46783 | 1.01E-09 | 1.72E-08 | 11.7789 |
| ZNF22 | 0.51736 | 4.980094 | 5.51282 | 1.28E-07 | 1.10E-06 | 7.10153 |
| NAGA | 0.51739 | 3.955574 | 5.5647 | 9.98E-08 | 8.89E-07 | 7.34287 |
| DDX5 | 0.51746 | 8.572018 | 8.00283 | 1.78E-13 | 1.07E-11 | 20.1708 |
| PIK3CG | 0.51748 | 1.393448 | 5.48951 | 1.44E-07 | 1.22E-06 | 6.99364 |
| ZNF45 | 0.51755 | 3.101717 | 6.90122 | 9.65E-11 | 2.33E-09 | 14.0509 |
| GSC | 0.51762 | 1.174435 | 3.92017 | 0.00013 | 0.000465 | 0.53237 |
| LYAR | 0.51784 | 3.480504 | 7.7371 | 8.44E-13 | 4.03E-11 | 18.654 |
| DERL2 | 0.51787 | 5.020986 | 7.47186 | 3.91E-12 | 1.49E-10 | 17.1644 |
| RER1 | 0.51798 | 5.928948 | 7.87332 | 3.81E-13 | 2.02E-11 | 19.4286 |
| SNHG7 | 0.51805 | 5.586267 | 3.8013 | 0.0002 | 0.00069 | 0.11377 |
| FEM1C | 0.51828 | 3.716289 | 6.48873 | 9.01E-10 | 1.56E-08 | 11.8864 |
| PTPN12 | 0.51831 | 5.372725 | 6.62119 | 4.43E-10 | 8.49E-09 | 12.573 |
| COL9A1 | 0.51843 | 2.185505 | 2.87325 | 0.00458 | 0.010768 | -2.7674 |
| P2RY11 | 0.51843 | 3.805545 | 6.25834 | 3.03E-09 | 4.45E-08 | 10.7122 |
| NOP10 | 0.51844 | 6.705044 | 7.47776 | 3.78E-12 | 1.45E-10 | 17.1973 |
| RP11-75L1.2 | 0.51847 | 2.523319 | 5.05663 | 1.09E-06 | 7.05E-06 | 5.04902 |
| ERAP1 | 0.51856 | 4.350321 | 5.31459 | 3.30E-07 | 2.50E-06 | 6.19411 |
| COQ2 | 0.5186 | 3.058711 | 7.75703 | 7.52E-13 | 3.64E-11 | 18.7669 |
| SSR1 | 0.51863 | 5.688718 | 6.85772 | 1.23E-10 | 2.87E-09 | 13.819 |
| SLC25A19 | 0.51873 | 3.16526 | 5.50171 | 1.35E-07 | 1.16E-06 | 7.05009 |
| TOR4A | 0.51886 | 1.643515 | 5.78047 | 3.46E-08 | 3.58E-07 | 8.36294 |
| EIF4A1 | 0.51889 | 8.366128 | 7.00409 | 5.46E-11 | 1.46E-09 | 14.6023 |
| SLC25A13 | 0.51906 | 3.908972 | 6.41373 | 1.34E-09 | 2.19E-08 | 11.5013 |
| NDNF | 0.51927 | 1.612358 | 3.04519 | 0.00269 | 0.006777 | -2.2872 |
| IL2RB | 0.51933 | 0.850115 | 5.61793 | 7.71E-08 | 7.08E-07 | 7.59203 |
| NAIP | 0.51937 | 3.414216 | 6.18205 | 4.51E-09 | 6.22E-08 | 10.3292 |
| EDEM2 | 0.51941 | 3.835189 | 8.09663 | 1.02E-13 | 6.59E-12 | 20.7117 |
| REM1 | 0.51969 | 1.418903 | 2.95888 | 0.00353 | 0.008584 | -2.5314 |
| MASTL | 0.51973 | 2.312392 | 7.39963 | 5.90E-12 | 2.15E-10 | 16.7632 |
| CD164 | 0.52009 | 5.931838 | 5.78128 | 3.45E-08 | 3.56E-07 | 8.36681 |
| PLXDC2 | 0.5204 | 3.890719 | 3.64949 | 0.00035 | 0.00113 | -0.405 |
| FUBP1 | 0.52042 | 5.819448 | 5.24842 | 4.50E-07 | 3.26E-06 | 5.89647 |
| RAP1A | 0.52057 | 5.868448 | 6.36998 | 1.69E-09 | 2.66E-08 | 11.278 |
| ZBTB42 | 0.52067 | 1.401064 | 5.13707 | 7.55E-07 | 5.11E-06 | 5.40168 |
| E2F5 | 0.52072 | 3.961008 | 4.90407 | 2.17E-06 | 1.29E-05 | 4.39156 |
| SDF4 | 0.52075 | 6.323754 | 7.2283 | 1.56E-11 | 4.97E-10 | 15.8195 |
| SKA2 | 0.52089 | 6.239272 | 6.59809 | 5.02E-10 | 9.41E-09 | 12.4527 |
| HIST2H2AA3 | 0.52108 | 1.847848 | 4.34895 | 2.35E-05 | 0.000104 | 2.13 |
| HDAC2 | 0.52118 | 5.973241 | 6.55026 | 6.48E-10 | 1.18E-08 | 12.2043 |
| DPAGT1 | 0.5212 | 4.426746 | 8.25244 | 4.03E-14 | 3.09E-12 | 21.6161 |
| GLB1L | 0.52125 | 3.099589 | 6.56823 | 5.89E-10 | 1.08E-08 | 12.2975 |
| SAMD13 | 0.52129 | 2.17206 | 4.8604 | 2.64E-06 | 1.53E-05 | 4.20613 |
| SPICE1 | 0.52153 | 2.676629 | 6.7578 | 2.12E-10 | 4.54E-09 | 13.2896 |
| ENTPD1 | 0.52195 | 3.974389 | 5.91659 | 1.75E-08 | 2.00E-07 | 9.01978 |
| ABCB7 | 0.52199 | 3.795288 | 8.18928 | 5.87E-14 | 4.18E-12 | 21.2486 |
| LAMC3 | 0.52206 | 2.527264 | 3.55685 | 0.00049 | 0.001514 | -0.7127 |
| PMM2 | 0.52216 | 3.146344 | 6.5679 | 5.90E-10 | 1.08E-08 | 12.2958 |
| APH1A | 0.52234 | 6.757248 | 8.4798 | 1.03E-14 | 1.02E-12 | 22.9487 |
| ASPN | 0.52235 | 1.172324 | 4.58897 | 8.59E-06 | 4.31E-05 | 3.08198 |
| ERF | 0.5224 | 5.301931 | 6.07484 | 7.83E-09 | 1.00E-07 | 9.79595 |
| MR1 | 0.52243 | 3.047668 | 4.77936 | 3.77E-06 | 2.09E-05 | 3.8653 |
| SCARNA2 | 0.52256 | 2.780681 | 2.63447 | 0.0092 | 0.019739 | -3.3921 |
| CLP1 | 0.52266 | 3.006395 | 8.65373 | 3.57E-15 | 4.21E-13 | 23.9778 |
| RP11-686D22.7 | 0.52286 | 1.077017 | 5.38806 | 2.33E-07 | 1.85E-06 | 6.52772 |
| RGS3 | 0.52288 | 4.054819 | 4.19995 | 4.29E-05 | 0.000177 | 1.55953 |
| SS18 | 0.52288 | 5.29842 | 6.53327 | 7.10E-10 | 1.27E-08 | 12.1164 |
| TMEM69 | 0.5231 | 4.338746 | 7.94995 | 2.43E-13 | 1.40E-11 | 19.8671 |
| MBOAT1 | 0.52335 | 1.332332 | 5.73419 | 4.35E-08 | 4.33E-07 | 8.14192 |
| LMO1 | 0.52335 | 2.274574 | 3.07733 | 0.00243 | 0.00621 | -2.1947 |
| CMPK2 | 0.52336 | 2.775652 | 4.20648 | 4.18E-05 | 0.000173 | 1.5842 |
| ING3 | 0.52337 | 4.208706 | 6.38501 | 1.56E-09 | 2.49E-08 | 11.3546 |
| IFI27 | 0.52373 | 6.492591 | 3.97694 | 0.0001 | 0.000383 | 0.73608 |
| PTPN13 | 0.52381 | 4.050527 | 5.1987 | 5.68E-07 | 3.98E-06 | 5.67458 |
| WAS | 0.52415 | 3.415394 | 4.37512 | 2.11E-05 | 9.47E-05 | 2.23181 |
| ZNF69 | 0.5243 | 1.996349 | 8.05145 | 1.33E-13 | 8.30E-12 | 20.4508 |
| RPLP0 | 0.52431 | 10.24359 | 4.57573 | 9.09E-06 | 4.52E-05 | 3.02843 |
| POFUT1 | 0.52435 | 4.339897 | 6.86304 | 1.19E-10 | 2.81E-09 | 13.8473 |
| GZMK | 0.52447 | 0.7148 | 5.06401 | 1.06E-06 | 6.84E-06 | 5.0812 |
| LMNA | 0.52462 | 5.758042 | 5.42652 | 1.94E-07 | 1.59E-06 | 6.70361 |
| SUSD1 | 0.52463 | 3.046086 | 5.56017 | 1.02E-07 | 9.04E-07 | 7.32173 |
| PTPN2 | 0.52497 | 3.802748 | 7.03382 | 4.63E-11 | 1.26E-09 | 14.7626 |
| ZNF790 | 0.525 | 2.689429 | 6.73838 | 2.35E-10 | 4.95E-09 | 13.1872 |
| CCDC19 | 0.52529 | 0.798841 | 3.44835 | 0.00071 | 0.002106 | -1.0644 |
| L3HYPDH | 0.5253 | 4.259917 | 5.82244 | 2.81E-08 | 2.98E-07 | 8.56441 |
| PRPF38A | 0.52534 | 4.778348 | 7.2335 | 1.51E-11 | 4.86E-10 | 15.848 |
| BCL10 | 0.52553 | 2.514112 | 8.76279 | 1.83E-15 | 2.49E-13 | 24.6272 |
| HEXB | 0.52558 | 5.429811 | 5.37149 | 2.52E-07 | 1.98E-06 | 6.4522 |
| SLX1A-SULT1A3 | 0.52559 | 3.067303 | 3.40191 | 0.00083 | 0.00242 | -1.212 |
| DKC1 | 0.5256 | 4.84486 | 7.89692 | 3.32E-13 | 1.81E-11 | 19.5635 |
| FAM76B | 0.52588 | 4.079379 | 5.95365 | 1.45E-08 | 1.70E-07 | 9.20038 |
| TMEM176B | 0.5259 | 5.343322 | 2.73183 | 0.00696 | 0.015525 | -3.1434 |
| CANT1 | 0.52594 | 4.307974 | 7.1004 | 3.20E-11 | 9.18E-10 | 15.1227 |
| SERTAD1 | 0.52609 | 2.718616 | 4.5356 | 1.08E-05 | 5.24E-05 | 2.86685 |
| GBX2 | 0.52623 | 2.357131 | 2.81034 | 0.00553 | 0.012695 | -2.9368 |
| C7orf73 | 0.52631 | 5.738603 | 6.68649 | 3.12E-10 | 6.27E-09 | 12.9145 |
| POU3F2 | 0.52655 | 4.513012 | 4.83066 | 3.01E-06 | 1.72E-05 | 4.08052 |
| SAMHD1 | 0.52676 | 4.789317 | 5.11818 | 8.24E-07 | 5.51E-06 | 5.3185 |
| LINC00339 | 0.52687 | 3.182506 | 6.76497 | 2.03E-10 | 4.39E-09 | 13.3274 |
| WASF2 | 0.52704 | 5.160919 | 5.37691 | 2.46E-07 | 1.93E-06 | 6.4769 |
| HIST1H2BC | 0.52711 | 1.932334 | 4.71195 | 5.06E-06 | 2.72E-05 | 3.58519 |
| HSPA5 | 0.52713 | 6.906496 | 5.71326 | 4.83E-08 | 4.72E-07 | 8.04237 |
| SHFM1 | 0.52719 | 7.324583 | 6.21899 | 3.72E-09 | 5.29E-08 | 10.5143 |
| PODXL | 0.52732 | 4.893512 | 5.76148 | 3.80E-08 | 3.86E-07 | 8.2721 |
| LYPLA2 | 0.52735 | 4.782177 | 7.02221 | 4.94E-11 | 1.34E-09 | 14.6999 |
| CTSL | 0.52741 | 6.432594 | 5.23305 | 4.84E-07 | 3.47E-06 | 5.82771 |
| VAT1 | 0.52754 | 6.287151 | 6.05135 | 8.83E-09 | 1.11E-07 | 9.67991 |
| SNX6 | 0.52792 | 5.591603 | 8.91219 | 7.32E-16 | 1.23E-13 | 25.5216 |
| MYO7A | 0.52798 | 2.341345 | 5.10555 | 8.73E-07 | 5.78E-06 | 5.26301 |
| CYFIP1 | 0.52816 | 5.741062 | 6.50591 | 8.22E-10 | 1.44E-08 | 11.975 |
| TMEM106A | 0.52833 | 1.894065 | 5.01424 | 1.32E-06 | 8.33E-06 | 4.86484 |
| DDIT4L | 0.52844 | 1.365827 | 2.72954 | 0.00701 | 0.015609 | -3.1493 |
| NBPF1 | 0.52845 | 5.267502 | 5.89655 | 1.94E-08 | 2.18E-07 | 8.92244 |
| RP11-153M7.1 | 0.52847 | 0.85678 | 5.10795 | 8.63E-07 | 5.73E-06 | 5.27355 |
| CLDN15 | 0.52855 | 3.201272 | 5.60694 | 8.13E-08 | 7.42E-07 | 7.54047 |
| CD72 | 0.52886 | 2.001884 | 4.75766 | 4.15E-06 | 2.28E-05 | 3.77481 |
| FBL | 0.52891 | 6.671911 | 6.17203 | 4.75E-09 | 6.50E-08 | 10.2791 |
| GBP4 | 0.52893 | 2.486046 | 4.81571 | 3.21E-06 | 1.82E-05 | 4.01765 |
| BATF3 | 0.52911 | 2.53879 | 3.86919 | 0.00016 | 0.000551 | 0.35151 |
| RBM12 | 0.52914 | 4.734197 | 5.78158 | 3.44E-08 | 3.56E-07 | 8.36826 |
| CPSF4 | 0.52917 | 5.452945 | 6.40228 | 1.42E-09 | 2.30E-08 | 11.4428 |
| PHF13 | 0.52929 | 3.260473 | 6.91204 | 9.09E-11 | 2.22E-09 | 14.1086 |
| FHL3 | 0.5293 | 3.902859 | 4.95764 | 1.71E-06 | 1.04E-05 | 4.62069 |
| NUP93 | 0.52943 | 4.786076 | 8.19289 | 5.75E-14 | 4.10E-12 | 21.2696 |
| HM13 | 0.52946 | 6.468462 | 7.80293 | 5.75E-13 | 2.90E-11 | 19.0276 |
| C16orf87 | 0.52952 | 3.259332 | 6.75836 | 2.11E-10 | 4.53E-09 | 13.2926 |
| N4BP2 | 0.53003 | 2.095463 | 6.94354 | 7.64E-11 | 1.92E-09 | 14.2772 |
| NOL8 | 0.5301 | 4.316009 | 8.68629 | 2.92E-15 | 3.59E-13 | 24.1714 |
| BNIP2 | 0.5302 | 4.841635 | 7.36946 | 7.01E-12 | 2.49E-10 | 16.5962 |
| GRAP | 0.53048 | 2.079717 | 5.58126 | 9.21E-08 | 8.26E-07 | 7.42019 |
| ARF6 | 0.53057 | 4.787354 | 7.278 | 1.18E-11 | 3.90E-10 | 16.0921 |
| FEN1 | 0.53062 | 4.073967 | 6.83777 | 1.37E-10 | 3.14E-09 | 13.7129 |
| ZNF345 | 0.53067 | 2.165918 | 7.58808 | 2.00E-12 | 8.36E-11 | 17.814 |
| MANF | 0.53079 | 5.806306 | 5.64996 | 6.59E-08 | 6.20E-07 | 7.74277 |
| SIRPB2 | 0.53083 | 1.522718 | 5.0976 | 9.05E-07 | 5.97E-06 | 5.22812 |
| RP11-253E3.3 | 0.53121 | 0.674208 | 5.54392 | 1.10E-07 | 9.69E-07 | 7.24602 |
| CD37 | 0.5314 | 4.44463 | 3.24107 | 0.00143 | 0.003893 | -1.7098 |
| ZNF561 | 0.53144 | 4.134557 | 6.5358 | 7.01E-10 | 1.26E-08 | 12.1295 |
| HOXC6 | 0.53149 | 0.639486 | 4.17935 | 4.66E-05 | 0.000191 | 1.48195 |
| FAM72D | 0.5315 | 0.995469 | 7.67238 | 1.23E-12 | 5.52E-11 | 18.2882 |
| ZNRD1 | 0.53157 | 4.361802 | 7.6046 | 1.82E-12 | 7.74E-11 | 17.9067 |
| NAT2 | 0.53194 | 0.731148 | 5.25372 | 4.39E-07 | 3.19E-06 | 5.92022 |
| RP11-303E16.2 | 0.53197 | 1.919017 | 5.57354 | 9.57E-08 | 8.55E-07 | 7.38413 |
| ADORA3 | 0.53225 | 5.142556 | 3.28379 | 0.00124 | 0.003436 | -1.5797 |
| TARBP2 | 0.53228 | 4.63584 | 7.67111 | 1.24E-12 | 5.55E-11 | 18.2811 |
| ITGAX | 0.5323 | 3.835508 | 3.56293 | 0.00048 | 0.001488 | -0.6927 |
| FAM129B | 0.53232 | 4.924218 | 4.79 | 3.60E-06 | 2.01E-05 | 3.90981 |
| DDB2 | 0.53248 | 3.051155 | 5.33031 | 3.07E-07 | 2.34E-06 | 6.26522 |
| PCED1B | 0.53259 | 1.826367 | 4.93489 | 1.89E-06 | 1.14E-05 | 4.52317 |
| TRAFD1 | 0.53259 | 4.903511 | 8.46876 | 1.10E-14 | 1.07E-12 | 22.8837 |
| ZNF738 | 0.5326 | 3.68208 | 5.36478 | 2.61E-07 | 2.03E-06 | 6.42164 |
| GOLIM4 | 0.53274 | 6.36078 | 4.56158 | 9.65E-06 | 4.76E-05 | 2.97133 |
| CPSF6 | 0.53301 | 4.978455 | 5.8963 | 1.94E-08 | 2.18E-07 | 8.92123 |
| HOXA2 | 0.53314 | 0.566146 | 3.9196 | 0.00013 | 0.000466 | 0.53033 |
| RIPK2 | 0.53328 | 4.062605 | 4.97943 | 1.55E-06 | 9.55E-06 | 4.71443 |
| NFATC3 | 0.53329 | 3.372076 | 8.45707 | 1.18E-14 | 1.13E-12 | 22.8148 |
| IRF5 | 0.53332 | 3.076 | 4.30598 | 2.80E-05 | 0.000122 | 1.96386 |
| ITPRIP | 0.53342 | 2.844865 | 5.35909 | 2.68E-07 | 2.08E-06 | 6.39577 |
| RRM1 | 0.53353 | 4.667401 | 5.74436 | 4.14E-08 | 4.15E-07 | 8.19042 |
| ZNF662 | 0.53366 | 2.039651 | 4.01118 | 9.02E-05 | 0.00034 | 0.86012 |
| PPIB | 0.53369 | 8.137055 | 7.16833 | 2.18E-11 | 6.69E-10 | 15.492 |
| KLHL4 | 0.53372 | 2.679504 | 3.32169 | 0.00109 | 0.003072 | -1.4629 |
| CCDC138 | 0.53374 | 2.105412 | 7.91588 | 2.97E-13 | 1.66E-11 | 19.6719 |
| RP11-71N10.1 | 0.53386 | 1.956024 | 3.83754 | 0.00017 | 0.000613 | 0.24025 |
| SUV39H2 | 0.53391 | 2.920797 | 6.398 | 1.46E-09 | 2.35E-08 | 11.4209 |
| VGLL4 | 0.53395 | 5.663734 | 6.4329 | 1.21E-09 | 2.01E-08 | 11.5995 |
| STAT5A | 0.53406 | 2.983689 | 4.63486 | 7.06E-06 | 3.63E-05 | 3.26854 |
| LAPTM4B | 0.53435 | 6.210505 | 5.45274 | 1.71E-07 | 1.43E-06 | 6.82407 |
| ACRBP | 0.53448 | 1.751117 | 4.99518 | 1.44E-06 | 8.96E-06 | 4.78238 |
| RP1-74M1.3 | 0.5347 | 1.013827 | 4.59223 | 8.47E-06 | 4.26E-05 | 3.09521 |
| TMED9 | 0.53471 | 6.569572 | 7.30338 | 1.02E-11 | 3.48E-10 | 16.2317 |
| CYB5RL | 0.53487 | 2.080218 | 7.72126 | 9.26E-13 | 4.35E-11 | 18.5644 |
| FAM229A | 0.5351 | 2.459453 | 4.98115 | 1.54E-06 | 9.49E-06 | 4.72188 |
| DNAJC22 | 0.53511 | 1.344714 | 4.99884 | 1.42E-06 | 8.84E-06 | 4.79822 |
| GMIP | 0.53518 | 3.5771 | 6.00166 | 1.14E-08 | 1.38E-07 | 9.43538 |
| IFRD1 | 0.53528 | 5.359375 | 6.53257 | 7.13E-10 | 1.27E-08 | 12.1128 |
| CASP7 | 0.53531 | 2.834987 | 5.25303 | 4.41E-07 | 3.20E-06 | 5.91712 |
| AEN | 0.53533 | 3.416456 | 4.44432 | 1.58E-05 | 7.37E-05 | 2.50341 |
| EPHA1 | 0.53533 | 1.292887 | 4.87301 | 2.49E-06 | 1.46E-05 | 4.25951 |
| DUSP16 | 0.53556 | 3.822237 | 5.96505 | 1.37E-08 | 1.62E-07 | 9.25606 |
| ZFP36 | 0.53556 | 5.923158 | 2.58513 | 0.01057 | 0.022238 | -3.515 |
| ASUN | 0.5356 | 4.091114 | 7.10875 | 3.05E-11 | 8.85E-10 | 15.1679 |
| ZNF260 | 0.53566 | 3.394607 | 6.47771 | 9.55E-10 | 1.64E-08 | 11.8297 |
| PELI1 | 0.53569 | 4.464433 | 6.06483 | 8.24E-09 | 1.04E-07 | 9.74647 |
| TIA1 | 0.53587 | 5.828092 | 5.30817 | 3.40E-07 | 2.56E-06 | 6.16513 |
| ILF2 | 0.53602 | 7.092489 | 7.71944 | 9.36E-13 | 4.39E-11 | 18.5541 |
| RNF138 | 0.53613 | 4.264518 | 7.08085 | 3.56E-11 | 1.01E-09 | 15.0167 |
| AGBL2 | 0.53614 | 1.35716 | 4.25663 | 3.42E-05 | 0.000145 | 1.77464 |
| RP4-545K15.3 | 0.53617 | 1.872327 | 2.46847 | 0.01455 | 0.029267 | -3.797 |
| MTF2 | 0.53618 | 3.179199 | 7.69571 | 1.07E-12 | 4.91E-11 | 18.4199 |
| STAT3 | 0.53619 | 5.896513 | 6.45694 | 1.07E-09 | 1.80E-08 | 11.7229 |
| NFATC1 | 0.5366 | 2.451776 | 4.61044 | 7.84E-06 | 3.98E-05 | 3.16908 |
| AC011242.6 | 0.53663 | 1.567832 | 4.31095 | 2.74E-05 | 0.00012 | 1.98299 |
| MMS22L | 0.5367 | 1.892771 | 7.73127 | 8.73E-13 | 4.16E-11 | 18.621 |
| HOXD11 | 0.53682 | 0.585237 | 3.49854 | 0.0006 | 0.001808 | -0.9028 |
| CARD6 | 0.53687 | 1.740636 | 6.05008 | 8.89E-09 | 1.11E-07 | 9.67364 |
| C19orf10 | 0.53703 | 6.030098 | 7.93072 | 2.72E-13 | 1.54E-11 | 19.7569 |
| TGFBR2 | 0.53712 | 4.152916 | 4.67771 | 5.87E-06 | 3.09E-05 | 3.44407 |
| YTHDF2 | 0.53724 | 4.970005 | 8.32946 | 2.54E-14 | 2.11E-12 | 22.0659 |
| BACH1 | 0.53726 | 3.591599 | 7.15629 | 2.34E-11 | 7.06E-10 | 15.4264 |
| FKBP9 | 0.5375 | 4.634 | 3.44014 | 0.00073 | 0.00216 | -1.0906 |
| FKBP15 | 0.53773 | 4.292536 | 8.76517 | 1.81E-15 | 2.47E-13 | 24.6414 |
| BCL6B | 0.53789 | 2.193501 | 4.44603 | 1.57E-05 | 7.32E-05 | 2.51018 |
| KDELR1 | 0.53794 | 5.918448 | 6.11772 | 6.28E-09 | 8.25E-08 | 10.0085 |
| TNFSF10 | 0.53797 | 3.026702 | 3.78178 | 0.00022 | 0.000737 | 0.04607 |
| TMEM255B | 0.53799 | 1.990236 | 4.83963 | 2.89E-06 | 1.66E-05 | 4.11836 |
| DNA2 | 0.5382 | 1.7083 | 6.81542 | 1.54E-10 | 3.49E-09 | 13.5943 |
| CCDC15 | 0.53821 | 1.361933 | 7.87638 | 3.74E-13 | 1.99E-11 | 19.4461 |
| CD302 | 0.53822 | 3.086078 | 4.24089 | 3.64E-05 | 0.000154 | 1.71468 |
| CTC-241F20.3 | 0.53825 | 1.485774 | 8.34165 | 2.36E-14 | 1.97E-12 | 22.1372 |
| TRIM45 | 0.53835 | 2.521169 | 6.60606 | 4.81E-10 | 9.07E-09 | 12.4942 |
| PABPC1L | 0.53849 | 4.190901 | 4.18483 | 4.56E-05 | 0.000187 | 1.50254 |
| BLNK | 0.53851 | 2.575084 | 3.79583 | 0.0002 | 0.000703 | 0.09477 |
| OST4 | 0.53863 | 7.846156 | 8.61192 | 4.60E-15 | 5.30E-13 | 23.7298 |
| SH2B3 | 0.53883 | 3.318688 | 5.58584 | 9.01E-08 | 8.10E-07 | 7.44162 |
| FRRS1 | 0.53889 | 0.730783 | 6.2367 | 3.39E-09 | 4.88E-08 | 10.6033 |
| PIK3C3 | 0.53898 | 4.907228 | 7.41386 | 5.44E-12 | 2.00E-10 | 16.8421 |
| VWF | 0.53917 | 3.79948 | 3.86963 | 0.00015 | 0.00055 | 0.35307 |
| SH2D4A | 0.53933 | 0.948019 | 4.11133 | 6.10E-05 | 0.000241 | 1.22792 |
| ARHGEF1 | 0.53951 | 5.317949 | 5.68147 | 5.64E-08 | 5.43E-07 | 7.89163 |
| CETN3 | 0.53957 | 4.7706 | 6.57973 | 5.54E-10 | 1.03E-08 | 12.3572 |
| MS4A14 | 0.53957 | 1.339833 | 4.75171 | 4.26E-06 | 2.33E-05 | 3.75004 |
| RNPEP | 0.53958 | 4.549323 | 8.53971 | 7.13E-15 | 7.58E-13 | 23.3023 |
| TNFRSF10C | 0.53969 | 1.614198 | 5.45979 | 1.66E-07 | 1.38E-06 | 6.85652 |
| SNHG16 | 0.5399 | 4.681323 | 6.62106 | 4.43E-10 | 8.49E-09 | 12.5724 |
| RPL22 | 0.53991 | 8.662774 | 6.99099 | 5.88E-11 | 1.55E-09 | 14.5318 |
| SLFN11 | 0.54003 | 2.841709 | 4.10492 | 6.25E-05 | 0.000247 | 1.20416 |
| PAWR | 0.54003 | 1.928287 | 4.42535 | 1.71E-05 | 7.87E-05 | 2.42864 |
| ARL2BP | 0.54005 | 5.945596 | 9.04058 | 3.31E-16 | 6.83E-14 | 26.2944 |
| PCDH12 | 0.54008 | 2.055293 | 4.59287 | 8.45E-06 | 4.25E-05 | 3.09778 |
| CCDC167 | 0.54022 | 5.64751 | 5.61835 | 7.69E-08 | 7.07E-07 | 7.594 |
| LSM7 | 0.54025 | 6.823961 | 6.93603 | 7.96E-11 | 1.99E-09 | 14.2369 |
| MYO5B | 0.54028 | 1.524487 | 3.73133 | 0.00026 | 0.000869 | -0.1275 |
| PALMD | 0.54055 | 2.998055 | 5.54301 | 1.11E-07 | 9.73E-07 | 7.24177 |
| MUTYH | 0.54056 | 4.262822 | 7.11036 | 3.02E-11 | 8.79E-10 | 15.1767 |
| SLC39A7 | 0.54086 | 6.096265 | 9.05694 | 2.99E-16 | 6.45E-14 | 26.3932 |
| NEXN | 0.54086 | 1.897596 | 4.50591 | 1.22E-05 | 5.85E-05 | 2.748 |
| ZDHHC15 | 0.54089 | 2.860709 | 6.47209 | 9.84E-10 | 1.68E-08 | 11.8008 |
| DLEC1 | 0.54094 | 1.544176 | 3.98218 | 0.0001 | 0.000376 | 0.75498 |
| TMC8 | 0.54103 | 2.459614 | 5.29815 | 3.57E-07 | 2.67E-06 | 6.11993 |
| UPP1 | 0.54113 | 3.990889 | 3.5866 | 0.00044 | 0.001381 | -0.6146 |
| TTC32 | 0.54128 | 4.262529 | 5.22245 | 5.08E-07 | 3.61E-06 | 5.78039 |
| ECE1 | 0.54147 | 4.640213 | 4.53164 | 1.10E-05 | 5.32E-05 | 2.85094 |
| IKZF1 | 0.54155 | 2.389993 | 4.70409 | 5.24E-06 | 2.80E-05 | 3.55271 |
| WDR12 | 0.5417 | 4.845231 | 7.52687 | 2.85E-12 | 1.13E-10 | 17.4713 |
| RUVBL1 | 0.54187 | 5.108436 | 8.15893 | 7.04E-14 | 4.79E-12 | 21.0724 |
| STON1 | 0.54191 | 3.522099 | 3.47884 | 0.00064 | 0.001919 | -0.9665 |
| SHMT2 | 0.54191 | 6.174058 | 3.97146 | 0.00011 | 0.00039 | 0.7163 |
| ITGB1P1 | 0.54195 | 2.148439 | 3.12102 | 0.00212 | 0.005487 | -2.0675 |
| KIAA0922 | 0.54197 | 2.312216 | 6.10066 | 6.86E-09 | 8.91E-08 | 9.92382 |
| LTBP1 | 0.54234 | 3.650566 | 3.60148 | 0.00041 | 0.001318 | -0.5653 |
| INSM1 | 0.54235 | 2.549084 | 3.17726 | 0.00176 | 0.00467 | -1.9014 |
| ZNF548 | 0.54245 | 3.278997 | 6.5066 | 8.19E-10 | 1.44E-08 | 11.9786 |
| SPN | 0.54252 | 1.799119 | 4.70812 | 5.15E-06 | 2.76E-05 | 3.56935 |
| NODAL | 0.54261 | 1.03747 | 4.3763 | 2.10E-05 | 9.43E-05 | 2.23643 |
| KIAA0020 | 0.54261 | 3.363614 | 6.93092 | 8.19E-11 | 2.03E-09 | 14.2096 |
| ZCCHC9 | 0.5427 | 3.765264 | 8.71955 | 2.39E-15 | 3.06E-13 | 24.3694 |
| C11orf96 | 0.54277 | 4.924818 | 3.33907 | 0.00103 | 0.002921 | -1.409 |
| SHMT1 | 0.543 | 3.553921 | 6.93854 | 7.85E-11 | 1.97E-09 | 14.2504 |
| MCM4 | 0.54323 | 5.098487 | 4.82618 | 3.07E-06 | 1.75E-05 | 4.06167 |
| HNRNPR | 0.54341 | 6.544596 | 9.41298 | 3.26E-17 | 1.10E-14 | 28.5566 |
| C12orf5 | 0.54344 | 3.791732 | 6.27173 | 2.83E-09 | 4.18E-08 | 10.7798 |
| IKBKE | 0.54356 | 1.955043 | 5.72459 | 4.56E-08 | 4.50E-07 | 8.09624 |
| RP11-126K1.6 | 0.54373 | 3.242302 | 5.0993 | 8.98E-07 | 5.93E-06 | 5.23556 |
| P4HB | 0.54381 | 7.561599 | 5.87003 | 2.21E-08 | 2.44E-07 | 8.79396 |
| PLXND1 | 0.5439 | 5.469978 | 4.92335 | 1.99E-06 | 1.19E-05 | 4.47381 |
| ZBTB12 | 0.54391 | 3.449973 | 5.26554 | 4.16E-07 | 3.04E-06 | 5.97325 |
| DCAF12 | 0.54393 | 4.651403 | 6.99663 | 5.69E-11 | 1.51E-09 | 14.5622 |
| LHX9 | 0.54396 | 1.028963 | 4.04121 | 8.03E-05 | 0.000308 | 0.96961 |
| THBD | 0.54432 | 1.508994 | 3.37261 | 0.00092 | 0.002642 | -1.3043 |
| IFI35 | 0.54444 | 4.555908 | 4.60616 | 7.98E-06 | 4.04E-05 | 3.1517 |
| CREB3L2 | 0.54457 | 3.188232 | 6.45068 | 1.10E-09 | 1.85E-08 | 11.6907 |
| XPR1 | 0.54468 | 4.099046 | 6.38008 | 1.60E-09 | 2.54E-08 | 11.3295 |
| SPTSSA | 0.54499 | 4.772269 | 5.8653 | 2.26E-08 | 2.49E-07 | 8.77113 |
| DNAJB11 | 0.54501 | 5.738716 | 7.76647 | 7.12E-13 | 3.48E-11 | 18.8205 |
| PLEKHA2 | 0.54509 | 4.201388 | 7.41692 | 5.35E-12 | 1.97E-10 | 16.8591 |
| PLEKHG4 | 0.54526 | 1.854857 | 3.8209 | 0.00019 | 0.000649 | 0.18204 |
| PCOLCE2 | 0.5454 | 2.197675 | 3.29335 | 0.0012 | 0.00334 | -1.5503 |
| CTC-425F1.4 | 0.54558 | 3.305367 | 3.11136 | 0.00218 | 0.005641 | -2.0958 |
| DDX60 | 0.54559 | 2.993306 | 5.21255 | 5.32E-07 | 3.76E-06 | 5.73623 |
| SDCCAG3 | 0.54567 | 5.316275 | 6.71735 | 2.64E-10 | 5.43E-09 | 13.0766 |
| WIPF1 | 0.54568 | 4.799861 | 6.14901 | 5.35E-09 | 7.20E-08 | 10.1642 |
| SH3BP2 | 0.5457 | 5.164223 | 4.89496 | 2.26E-06 | 1.34E-05 | 4.35276 |
| PRIM1 | 0.54573 | 3.183808 | 6.62107 | 4.43E-10 | 8.49E-09 | 12.5724 |
| RP4-798A10.7 | 0.54579 | 2.171308 | 4.37902 | 2.07E-05 | 9.34E-05 | 2.24706 |
| PSMA5 | 0.54617 | 5.828264 | 8.28853 | 3.24E-14 | 2.62E-12 | 21.8266 |
| RP11-147I3.1 | 0.54639 | 1.64276 | 5.35379 | 2.74E-07 | 2.13E-06 | 6.3717 |
| RELB | 0.54639 | 2.739846 | 4.66777 | 6.13E-06 | 3.21E-05 | 3.40324 |
| RGS10 | 0.5464 | 5.360389 | 3.93442 | 0.00012 | 0.000443 | 0.58326 |
| SPAG1 | 0.54641 | 2.361554 | 4.43242 | 1.66E-05 | 7.68E-05 | 2.45646 |
| SERTAD3 | 0.54643 | 3.245812 | 5.14592 | 7.25E-07 | 4.92E-06 | 5.4407 |
| STK3 | 0.54654 | 3.156715 | 6.36643 | 1.72E-09 | 2.70E-08 | 11.2599 |
| ERCC6L | 0.54661 | 0.64844 | 6.61369 | 4.61E-10 | 8.76E-09 | 12.5339 |
| INTS7 | 0.54666 | 3.277188 | 6.72939 | 2.47E-10 | 5.15E-09 | 13.1399 |
| NFATC2 | 0.54684 | 1.86754 | 5.44855 | 1.75E-07 | 1.45E-06 | 6.80478 |
| FAM72B | 0.54704 | 1.798173 | 7.17138 | 2.15E-11 | 6.59E-10 | 15.5086 |
| CENPJ | 0.54718 | 2.663252 | 5.89884 | 1.91E-08 | 2.16E-07 | 8.93357 |
| ILDR2 | 0.54727 | 3.420739 | 4.7125 | 5.05E-06 | 2.71E-05 | 3.58746 |
| RP5-886K2.3 | 0.54733 | 2.357176 | 6.37188 | 1.67E-09 | 2.64E-08 | 11.2877 |
| ARMCX6 | 0.54746 | 5.351963 | 7.35363 | 7.67E-12 | 2.71E-10 | 16.5087 |
| SERPINB1 | 0.54746 | 3.652361 | 4.06435 | 7.33E-05 | 0.000284 | 1.05444 |
| CTD-2510F5.4 | 0.54748 | 1.272511 | 4.59324 | 8.44E-06 | 4.24E-05 | 3.09928 |
| SPRY4 | 0.54759 | 2.665523 | 2.68483 | 0.00797 | 0.017431 | -3.2645 |
| HILS1 | 0.54764 | 0.786987 | 4.12921 | 5.68E-05 | 0.000227 | 1.29435 |
| AC008738.1 | 0.54787 | 2.98043 | 3.08536 | 0.00237 | 0.006073 | -2.1714 |
| C5orf15 | 0.54789 | 4.997091 | 7.42764 | 5.03E-12 | 1.87E-10 | 16.9186 |
| C1orf85 | 0.54793 | 4.494759 | 5.48948 | 1.44E-07 | 1.22E-06 | 6.99348 |
| HN1 | 0.54812 | 6.836876 | 5.00092 | 1.41E-06 | 8.78E-06 | 4.8072 |
| RPS15A | 0.54823 | 10.05503 | 6.12696 | 5.99E-09 | 7.93E-08 | 10.0544 |
| SNAI2 | 0.54824 | 1.822219 | 3.74592 | 0.00025 | 0.000829 | -0.0775 |
| KLHL6 | 0.54827 | 1.553807 | 5.4413 | 1.81E-07 | 1.50E-06 | 6.77147 |
| ITGA7 | 0.54829 | 5.705174 | 4.1139 | 6.04E-05 | 0.000239 | 1.23746 |
| MAPKAPK2 | 0.54836 | 5.241079 | 6.76201 | 2.07E-10 | 4.45E-09 | 13.3118 |
| ETV1 | 0.54853 | 6.361834 | 2.5046 | 0.0132 | 0.026912 | -3.7109 |
| GPX1 | 0.5487 | 7.803821 | 5.5489 | 1.08E-07 | 9.48E-07 | 7.2692 |
| WARS2-IT1 | 0.54877 | 0.766024 | 6.24335 | 3.28E-09 | 4.75E-08 | 10.6367 |
| PHEX | 0.54891 | 1.119205 | 5.85081 | 2.44E-08 | 2.64E-07 | 8.70111 |
| FREM2 | 0.54895 | 1.21826 | 4.38214 | 2.05E-05 | 9.23E-05 | 2.25922 |
| C21orf62 | 0.54906 | 3.2289 | 2.37616 | 0.0186 | 0.036167 | -4.0115 |
| ZNF93 | 0.54915 | 2.675105 | 5.22895 | 4.93E-07 | 3.52E-06 | 5.8094 |
| SALL3 | 0.54942 | 3.646825 | 3.21231 | 0.00157 | 0.004223 | -1.7966 |
| C4A-AS1 | 0.54946 | 1.342921 | 4.61679 | 7.63E-06 | 3.88E-05 | 3.19492 |
| C4B-AS1 | 0.54946 | 1.342921 | 4.61679 | 7.63E-06 | 3.88E-05 | 3.19492 |
| HP1BP3 | 0.54952 | 7.008361 | 6.45582 | 1.07E-09 | 1.81E-08 | 11.7171 |
| ITGAV | 0.54954 | 5.817047 | 5.51754 | 1.25E-07 | 1.08E-06 | 7.12345 |
| CCL8 | 0.54956 | 1.079273 | 3.21898 | 0.00154 | 0.004147 | -1.7765 |
| BICD1 | 0.54961 | 3.74419 | 5.89889 | 1.91E-08 | 2.16E-07 | 8.93381 |
| CBX8 | 0.5498 | 2.935911 | 5.97502 | 1.30E-08 | 1.55E-07 | 9.30485 |
| TLR6 | 0.5499 | 1.481986 | 6.04792 | 8.98E-09 | 1.13E-07 | 9.663 |
| RP11-155G14.5 | 0.55019 | 1.51807 | 3.2913 | 0.00121 | 0.003359 | -1.5566 |
| CCDC28B | 0.55021 | 4.360208 | 6.69008 | 3.06E-10 | 6.17E-09 | 12.9333 |
| NR4A3 | 0.55037 | 1.719908 | 3.54259 | 0.00051 | 0.001581 | -0.7594 |
| MGST2 | 0.55044 | 4.416396 | 5.19248 | 5.84E-07 | 4.08E-06 | 5.64693 |
| RN7SK | 0.55047 | 8.212233 | 2.23611 | 0.02664 | 0.049362 | -4.3224 |
| C1orf86 | 0.55058 | 5.255961 | 6.42491 | 1.26E-09 | 2.07E-08 | 11.5586 |
| CECR1 | 0.55063 | 3.531708 | 3.91605 | 0.00013 | 0.000472 | 0.51767 |
| SPSB4 | 0.55078 | 2.936597 | 3.49828 | 0.0006 | 0.00181 | -0.9037 |
| MSH6 | 0.55112 | 4.613409 | 6.63526 | 4.11E-10 | 7.98E-09 | 12.6464 |
| TMEM217 | 0.55131 | 1.395336 | 5.32733 | 3.11E-07 | 2.37E-06 | 6.25173 |
| DNAH11 | 0.55141 | 0.747516 | 4.73782 | 4.52E-06 | 2.46E-05 | 3.69231 |
| ADCY7 | 0.55147 | 3.483624 | 5.41306 | 2.07E-07 | 1.68E-06 | 6.64195 |
| ADAM17 | 0.55158 | 4.134028 | 6.84046 | 1.35E-10 | 3.11E-09 | 13.7272 |
| PDCD6IP | 0.55162 | 6.209341 | 7.01731 | 5.08E-11 | 1.37E-09 | 14.6735 |
| MAP3K19 | 0.55163 | 0.97907 | 3.46578 | 0.00067 | 0.001997 | -1.0085 |
| RP11-834C11.5 | 0.55166 | 0.80988 | 3.89274 | 0.00014 | 0.00051 | 0.43483 |
| FHOD1 | 0.55199 | 3.504568 | 4.84312 | 2.85E-06 | 1.64E-05 | 4.1331 |
| HOXA11 | 0.55229 | 0.520845 | 5.1935 | 5.82E-07 | 4.06E-06 | 5.65147 |
| RP11-1035H13.3 | 0.55244 | 1.163312 | 3.56889 | 0.00047 | 0.00146 | -0.6731 |
| KIF20B | 0.55244 | 1.513228 | 9.06804 | 2.80E-16 | 6.18E-14 | 26.4602 |
| EVA1A | 0.55249 | 1.510373 | 4.57378 | 9.17E-06 | 4.55E-05 | 3.02053 |
| RBM38 | 0.55273 | 4.337288 | 7.35832 | 7.47E-12 | 2.64E-10 | 16.5347 |
| PLBD1 | 0.55278 | 1.869532 | 4.49922 | 1.26E-05 | 6.00E-05 | 2.72129 |
| GAR1 | 0.55281 | 4.398299 | 7.44304 | 4.61E-12 | 1.73E-10 | 17.0041 |
| CAP1 | 0.55281 | 7.037948 | 7.14126 | 2.54E-11 | 7.57E-10 | 15.3446 |
| HSPB11 | 0.55285 | 4.338384 | 7.30807 | 9.93E-12 | 3.40E-10 | 16.2575 |
| ETV6 | 0.55291 | 2.631203 | 7.00517 | 5.43E-11 | 1.45E-09 | 14.6081 |
| GPR141 | 0.55295 | 0.733612 | 4.93468 | 1.89E-06 | 1.14E-05 | 4.52227 |
| ADAMDEC1 | 0.55359 | 0.744851 | 4.32347 | 2.60E-05 | 0.000114 | 2.03131 |
| PIF1 | 0.55378 | 1.813866 | 4.67352 | 5.98E-06 | 3.13E-05 | 3.42685 |
| PALD1 | 0.55385 | 4.308934 | 5.37173 | 2.52E-07 | 1.98E-06 | 6.45331 |
| TSPAN31 | 0.55401 | 5.899246 | 4.29941 | 2.87E-05 | 0.000125 | 1.93857 |
| ARL6IP6 | 0.55424 | 4.529196 | 6.44349 | 1.15E-09 | 1.91E-08 | 11.6538 |
| MICB | 0.55434 | 1.162591 | 6.44371 | 1.14E-09 | 1.91E-08 | 11.655 |
| LPCAT2 | 0.5544 | 3.04398 | 4.08298 | 6.82E-05 | 0.000266 | 1.12302 |
| P2RY6 | 0.55452 | 1.753757 | 4.15197 | 5.19E-05 | 0.00021 | 1.37927 |
| HIST1H2BG | 0.5546 | 0.987483 | 5.17611 | 6.31E-07 | 4.36E-06 | 5.57429 |
| C7orf49 | 0.55472 | 4.964456 | 7.69552 | 1.08E-12 | 4.91E-11 | 18.4188 |
| MIIP | 0.55476 | 4.98293 | 5.04018 | 1.18E-06 | 7.53E-06 | 4.97743 |
| DNAJC10 | 0.55527 | 4.984404 | 7.8333 | 4.81E-13 | 2.47E-11 | 19.2004 |
| RP1-43E13.2 | 0.55532 | 2.029738 | 5.25069 | 4.46E-07 | 3.23E-06 | 5.90663 |
| AC018766.5 | 0.55532 | 3.238869 | 5.28943 | 3.72E-07 | 2.76E-06 | 6.08062 |
| ANXA2R | 0.55534 | 1.403407 | 5.989 | 1.21E-08 | 1.46E-07 | 9.37328 |
| HK3 | 0.55547 | 1.707333 | 4.11493 | 6.01E-05 | 0.000238 | 1.24125 |
| RGS18 | 0.55547 | 1.74128 | 5.48633 | 1.46E-07 | 1.23E-06 | 6.97894 |
| WDR90 | 0.55574 | 4.142039 | 5.71806 | 4.71E-08 | 4.63E-07 | 8.0652 |
| ADAM28 | 0.55612 | 3.376769 | 3.95765 | 0.00011 | 0.000409 | 0.66658 |
| TMEM234 | 0.55623 | 3.502953 | 7.6917 | 1.10E-12 | 5.01E-11 | 18.3973 |
| MDM2 | 0.55632 | 4.97312 | 3.92278 | 0.00013 | 0.00046 | 0.54167 |
| HIST1H3H | 0.55683 | 0.806826 | 5.23227 | 4.86E-07 | 3.48E-06 | 5.82423 |
| ERAP2 | 0.55686 | 2.65123 | 3.24177 | 0.00143 | 0.003885 | -1.7077 |
| VASH1 | 0.55693 | 5.137344 | 6.81762 | 1.53E-10 | 3.46E-09 | 13.606 |
| AC011558.5 | 0.55716 | 2.235783 | 2.50861 | 0.01305 | 0.026668 | -3.7013 |
| EFNA4 | 0.55725 | 1.690606 | 6.19432 | 4.23E-09 | 5.90E-08 | 10.3906 |
| MYO1G | 0.5573 | 1.137031 | 4.65076 | 6.59E-06 | 3.42E-05 | 3.33353 |
| PRF1 | 0.55731 | 1.085329 | 5.28297 | 3.83E-07 | 2.83E-06 | 6.05155 |
| DMRTA2 | 0.55737 | 0.853214 | 3.346 | 0.00101 | 0.002861 | -1.3875 |
| SLC25A24 | 0.55743 | 2.115289 | 5.25951 | 4.28E-07 | 3.12E-06 | 5.94615 |
| ZNF473 | 0.55746 | 2.575066 | 7.4503 | 4.42E-12 | 1.66E-10 | 17.0445 |
| TMEM165 | 0.55754 | 5.469595 | 5.49811 | 1.38E-07 | 1.18E-06 | 7.03342 |
| TULP3 | 0.5576 | 3.881988 | 6.47194 | 9.85E-10 | 1.68E-08 | 11.8 |
| MAP2K3 | 0.55783 | 3.377 | 5.67056 | 5.95E-08 | 5.67E-07 | 7.84003 |
| MECOM | 0.55793 | 2.128183 | 5.46687 | 1.60E-07 | 1.34E-06 | 6.88913 |
| TLR5 | 0.558 | 2.072428 | 4.7264 | 4.75E-06 | 2.57E-05 | 3.64499 |
| ZSCAN16 | 0.55802 | 2.635942 | 8.69503 | 2.77E-15 | 3.44E-13 | 24.2234 |
| C9orf117 | 0.55809 | 1.288022 | 3.73746 | 0.00025 | 0.000852 | -0.1066 |
| CD163L1 | 0.55815 | 1.619622 | 4.1521 | 5.19E-05 | 0.00021 | 1.37977 |
| ASCL1 | 0.55816 | 4.914753 | 2.83745 | 0.0051 | 0.011826 | -2.8642 |
| MIA | 0.55829 | 1.291527 | 3.17092 | 0.0018 | 0.004756 | -1.9203 |
| STAC | 0.55829 | 0.951325 | 3.67374 | 0.00032 | 0.001047 | -0.3233 |
| FAM115C | 0.55833 | 1.010876 | 4.78571 | 3.67E-06 | 2.04E-05 | 3.89186 |
| E2F8 | 0.55872 | 0.808284 | 6.90519 | 9.44E-11 | 2.29E-09 | 14.072 |
| TUBB | 0.55873 | 8.30386 | 6.4577 | 1.06E-09 | 1.80E-08 | 11.7268 |
| PGM5 | 0.55891 | 2.581588 | 4.55393 | 9.97E-06 | 4.89E-05 | 2.94049 |
| RPIA | 0.55907 | 3.189612 | 9.72668 | 4.52E-18 | 2.62E-15 | 30.4837 |
| ERP27 | 0.55915 | 0.752529 | 5.28923 | 3.72E-07 | 2.76E-06 | 6.07973 |
| CMTR2 | 0.55937 | 3.336425 | 8.04125 | 1.42E-13 | 8.74E-12 | 20.392 |
| WISP1 | 0.55992 | 1.085053 | 4.0763 | 7.00E-05 | 0.000273 | 1.09843 |
| STT3A | 0.55997 | 5.494604 | 7.45029 | 4.42E-12 | 1.66E-10 | 17.0444 |
| RNASET2 | 0.56009 | 6.342862 | 4.15842 | 5.06E-05 | 0.000205 | 1.40341 |
| SIPA1L2 | 0.56043 | 4.450581 | 5.73933 | 4.24E-08 | 4.24E-07 | 8.16641 |
| MLKL | 0.56051 | 1.880992 | 6.23205 | 3.48E-09 | 4.99E-08 | 10.5799 |
| SCLT1 | 0.56056 | 2.432126 | 9.36307 | 4.45E-17 | 1.44E-14 | 28.2518 |
| ITGA2 | 0.56057 | 1.734172 | 4.51484 | 1.18E-05 | 5.66E-05 | 2.78367 |
| ATF1 | 0.56057 | 3.765871 | 6.94328 | 7.65E-11 | 1.92E-09 | 14.2758 |
| RP11-705C15.2 | 0.56057 | 2.231758 | 6.46686 | 1.01E-09 | 1.72E-08 | 11.7739 |
| PLXDC1 | 0.56058 | 4.077997 | 4.2742 | 3.18E-05 | 0.000136 | 1.8418 |
| SLC35D2 | 0.5607 | 4.048912 | 6.23957 | 3.34E-09 | 4.83E-08 | 10.6177 |
| GALNT2 | 0.56078 | 5.179421 | 6.20303 | 4.04E-09 | 5.66E-08 | 10.4343 |
| FUOM | 0.56084 | 3.00483 | 4.84746 | 2.79E-06 | 1.61E-05 | 4.15139 |
| ORMDL2 | 0.56095 | 4.338826 | 7.24528 | 1.42E-11 | 4.60E-10 | 15.9126 |
| MAP3K8 | 0.56107 | 2.464926 | 4.90785 | 2.14E-06 | 1.27E-05 | 4.40766 |
| CREB1 | 0.56111 | 4.298158 | 6.46247 | 1.04E-09 | 1.76E-08 | 11.7513 |
| ATL3 | 0.56126 | 3.586974 | 5.73631 | 4.31E-08 | 4.29E-07 | 8.15205 |
| BCHE | 0.56167 | 5.975451 | 3.47069 | 0.00066 | 0.001967 | -0.9927 |
| PLB1 | 0.56192 | 2.164067 | 4.67493 | 5.94E-06 | 3.12E-05 | 3.43264 |
| FAM181A-AS1 | 0.56193 | 1.736375 | 3.17275 | 0.00179 | 0.004731 | -1.9148 |
| CEBPA | 0.56198 | 2.161327 | 3.06229 | 0.00255 | 0.006465 | -2.2381 |
| TLR10 | 0.56206 | 1.48792 | 4.93532 | 1.89E-06 | 1.14E-05 | 4.52498 |
| CCDC142 | 0.56236 | 3.054265 | 6.92821 | 8.31E-11 | 2.06E-09 | 14.1951 |
| HSPE1-MOB4 | 0.56249 | 2.93487 | 4.09559 | 6.49E-05 | 0.000255 | 1.16961 |
| MEX3D | 0.56263 | 3.792317 | 6.39075 | 1.51E-09 | 2.42E-08 | 11.3839 |
| NRP2 | 0.56301 | 4.705887 | 4.35811 | 2.26E-05 | 0.000101 | 2.16559 |
| TFDP2 | 0.56313 | 4.278873 | 6.03949 | 9.38E-09 | 1.17E-07 | 9.62145 |
| PLIN2 | 0.56336 | 4.11174 | 3.90278 | 0.00014 | 0.000494 | 0.47045 |
| NR2C2AP | 0.56362 | 3.959623 | 8.59194 | 5.19E-15 | 5.85E-13 | 23.6113 |
| EPS8 | 0.56378 | 4.49424 | 6.37503 | 1.64E-09 | 2.60E-08 | 11.3037 |
| SPATA1 | 0.56384 | 1.012146 | 5.77935 | 3.48E-08 | 3.58E-07 | 8.3576 |
| ADPGK | 0.56388 | 4.559017 | 8.32803 | 2.56E-14 | 2.12E-12 | 22.0575 |
| PRMT6 | 0.56393 | 3.407992 | 8.53425 | 7.37E-15 | 7.72E-13 | 23.27 |
| DAPP1 | 0.56401 | 1.153542 | 5.70337 | 5.07E-08 | 4.93E-07 | 7.99541 |
| DHX40 | 0.56404 | 5.123715 | 7.71147 | 9.80E-13 | 4.57E-11 | 18.509 |
| HMGB1P1 | 0.56406 | 0.975796 | 5.52747 | 1.20E-07 | 1.04E-06 | 7.16953 |
| GUSB | 0.56411 | 4.885398 | 7.00276 | 5.50E-11 | 1.47E-09 | 14.5952 |
| RIPK3 | 0.56416 | 1.861791 | 4.51548 | 1.17E-05 | 5.65E-05 | 2.78622 |
| IFITM3 | 0.56417 | 7.441263 | 3.15134 | 0.00192 | 0.005027 | -1.9783 |
| TRAC | 0.56421 | 1.363019 | 4.01769 | 8.79E-05 | 0.000333 | 0.8838 |
| AC096579.7 | 0.56425 | 1.509835 | 2.5981 | 0.01019 | 0.021538 | -3.4829 |
| PTAFR | 0.5644 | 2.730407 | 4.12504 | 5.78E-05 | 0.00023 | 1.27884 |
| PTPRZ1 | 0.56449 | 8.627772 | 3.89582 | 0.00014 | 0.000505 | 0.44574 |
| ADAMTS1 | 0.5645 | 2.913483 | 3.43748 | 0.00074 | 0.002177 | -1.0991 |
| ZMPSTE24 | 0.56451 | 4.666282 | 8.1576 | 7.09E-14 | 4.81E-12 | 21.0647 |
| WDR1 | 0.56464 | 6.718216 | 6.93149 | 8.16E-11 | 2.03E-09 | 14.2127 |
| POLD3 | 0.56488 | 3.214827 | 9.67986 | 6.08E-18 | 3.21E-15 | 30.1949 |
| PDIA6 | 0.56492 | 6.530837 | 6.73025 | 2.46E-10 | 5.13E-09 | 13.1444 |
| KLRC4-KLRK1 | 0.56508 | 1.55192 | 3.57427 | 0.00046 | 0.001435 | -0.6553 |
| TXNDC17 | 0.5651 | 5.92432 | 8.11887 | 8.93E-14 | 5.89E-12 | 20.8403 |
| SFT2D2 | 0.56511 | 3.164759 | 6.91835 | 8.78E-11 | 2.16E-09 | 14.1423 |
| FZD2 | 0.56533 | 1.896301 | 5.11686 | 8.29E-07 | 5.53E-06 | 5.31268 |
| MAP7D3 | 0.56539 | 2.153155 | 6.42651 | 1.25E-09 | 2.06E-08 | 11.5668 |
| RP1-152L7.5 | 0.56581 | 2.721962 | 5.96799 | 1.35E-08 | 1.60E-07 | 9.27041 |
| HEATR3 | 0.56589 | 3.111281 | 8.36427 | 2.06E-14 | 1.77E-12 | 22.2697 |
| NUP62 | 0.56599 | 4.72851 | 7.35006 | 7.83E-12 | 2.74E-10 | 16.489 |
| GZMH | 0.566 | 1.037114 | 5.2566 | 4.34E-07 | 3.15E-06 | 5.93313 |
| TMSB4XP8 | 0.56601 | 3.556391 | 3.46577 | 0.00067 | 0.001997 | -1.0085 |
| ARRDC3 | 0.56606 | 4.440656 | 5.61601 | 7.78E-08 | 7.14E-07 | 7.58301 |
| TRIT1 | 0.56606 | 3.853868 | 6.48389 | 9.24E-10 | 1.60E-08 | 11.8615 |
| EVC | 0.56614 | 1.617833 | 4.84592 | 2.81E-06 | 1.62E-05 | 4.14491 |
| TNPO1 | 0.56619 | 5.326486 | 6.74151 | 2.31E-10 | 4.88E-09 | 13.2037 |
| MIDN | 0.56621 | 5.870348 | 5.45711 | 1.68E-07 | 1.40E-06 | 6.84418 |
| EFHC1 | 0.5663 | 3.618203 | 6.16977 | 4.80E-09 | 6.57E-08 | 10.2678 |
| RAI14 | 0.56646 | 3.478416 | 5.94797 | 1.49E-08 | 1.75E-07 | 9.17266 |
| CD40 | 0.56647 | 2.406394 | 5.40143 | 2.19E-07 | 1.76E-06 | 6.58875 |
| DOK2 | 0.56654 | 1.25047 | 4.8307 | 3.01E-06 | 1.72E-05 | 4.08071 |
| 10-Sep | 0.56654 | 4.47177 | 5.85976 | 2.33E-08 | 2.55E-07 | 8.74434 |
| C10orf10 | 0.56665 | 4.4566 | 3.3515 | 0.00099 | 0.002814 | -1.3703 |
| HOXA11-AS | 0.56665 | 0.509544 | 5.09412 | 9.20E-07 | 6.06E-06 | 5.21285 |
| C4orf46 | 0.56667 | 2.356929 | 7.77328 | 6.84E-13 | 3.38E-11 | 18.8591 |
| DNMT1 | 0.56674 | 5.057603 | 7.2278 | 1.56E-11 | 4.98E-10 | 15.8168 |
| IRAK4 | 0.56682 | 3.129339 | 7.77347 | 6.83E-13 | 3.38E-11 | 18.8602 |
| H6PD | 0.56706 | 3.283421 | 6.35395 | 1.84E-09 | 2.85E-08 | 11.1964 |
| CHST14 | 0.56714 | 3.760754 | 6.30112 | 2.42E-09 | 3.64E-08 | 10.9283 |
| SRSF4 | 0.56719 | 5.733988 | 7.95254 | 2.39E-13 | 1.38E-11 | 19.8819 |
| HGFAC | 0.56723 | 1.773333 | 5.1373 | 7.54E-07 | 5.10E-06 | 5.40268 |
| SCPEP1 | 0.56734 | 5.18122 | 5.62994 | 7.27E-08 | 6.75E-07 | 7.6485 |
| FAIM3 | 0.56738 | 1.812244 | 4.27625 | 3.15E-05 | 0.000136 | 1.84967 |
| CYP19A1 | 0.56758 | 1.164829 | 4.63866 | 6.95E-06 | 3.58E-05 | 3.28408 |
| UHRF1 | 0.56774 | 3.929126 | 3.40443 | 0.00083 | 0.002404 | -1.2041 |
| BCAT1 | 0.56791 | 2.721286 | 3.31489 | 0.00112 | 0.003135 | -1.484 |
| GEN1 | 0.56803 | 1.735118 | 6.32986 | 2.09E-09 | 3.20E-08 | 11.0739 |
| HIST1H2AG | 0.56813 | 0.70574 | 7.72287 | 9.17E-13 | 4.32E-11 | 18.5734 |
| RHOJ | 0.56814 | 3.179688 | 4.59321 | 8.44E-06 | 4.24E-05 | 3.09918 |
| INMT | 0.56815 | 1.119099 | 3.79408 | 0.00021 | 0.000707 | 0.08869 |
| LSM5 | 0.56816 | 5.272024 | 6.95809 | 7.05E-11 | 1.79E-09 | 14.3552 |
| MED28 | 0.56818 | 4.378305 | 8.20675 | 5.29E-14 | 3.85E-12 | 21.3501 |
| PRCP | 0.56904 | 6.892695 | 5.38679 | 2.35E-07 | 1.86E-06 | 6.52194 |
| PDLIM7 | 0.56909 | 4.812212 | 4.33017 | 2.53E-05 | 0.000112 | 2.0572 |
| DOT1L | 0.56921 | 3.929553 | 5.21704 | 5.21E-07 | 3.69E-06 | 5.75626 |
| HEATR2 | 0.56921 | 3.953188 | 5.93163 | 1.62E-08 | 1.87E-07 | 9.09299 |
| F2RL2 | 0.56922 | 0.791645 | 4.40797 | 1.84E-05 | 8.39E-05 | 2.36032 |
| QTRTD1 | 0.56948 | 3.048624 | 7.90051 | 3.25E-13 | 1.78E-11 | 19.584 |
| HOXA9 | 0.56954 | 0.625446 | 3.71228 | 0.00028 | 0.000925 | -0.1926 |
| RP11-66N24.3 | 0.56958 | 1.728787 | 6.50804 | 8.13E-10 | 1.43E-08 | 11.986 |
| RFC2 | 0.56976 | 4.531418 | 8.11254 | 9.27E-14 | 6.08E-12 | 20.8037 |
| PAX3 | 0.56978 | 0.60763 | 3.6339 | 0.00037 | 0.001189 | -0.4572 |
| PNP | 0.56992 | 4.774144 | 5.49275 | 1.41E-07 | 1.20E-06 | 7.00863 |
| BTBD19 | 0.56994 | 2.078818 | 5.15769 | 6.87E-07 | 4.70E-06 | 5.49271 |
| TMEM39A | 0.56996 | 3.468103 | 7.00637 | 5.40E-11 | 1.45E-09 | 14.6146 |
| GLIPR2 | 0.56998 | 5.536442 | 4.90147 | 2.20E-06 | 1.30E-05 | 4.38046 |
| OSM | 0.57007 | 1.509643 | 2.96533 | 0.00346 | 0.00843 | -2.5134 |
| RP11-211G3.2 | 0.57012 | 1.222762 | 5.33082 | 3.06E-07 | 2.34E-06 | 6.26752 |
| TRAF4 | 0.57032 | 5.40926 | 4.29882 | 2.88E-05 | 0.000125 | 1.93628 |
| MS4A4E | 0.57033 | 0.593994 | 6.82212 | 1.49E-10 | 3.38E-09 | 13.6298 |
| BMP8B | 0.57064 | 1.83091 | 3.88858 | 0.00014 | 0.000516 | 0.42009 |
| DYNLT1 | 0.5707 | 6.923382 | 5.88306 | 2.07E-08 | 2.31E-07 | 8.85704 |
| IGFBP7-AS1 | 0.57075 | 1.664227 | 3.10367 | 0.00224 | 0.005758 | -2.1182 |
| LIMS1 | 0.57084 | 4.805387 | 5.67971 | 5.69E-08 | 5.47E-07 | 7.88329 |
| THBS3 | 0.57088 | 4.200134 | 6.12783 | 5.96E-09 | 7.90E-08 | 10.0588 |
| HSPA1B | 0.57096 | 6.388731 | 2.99325 | 0.00317 | 0.007817 | -2.4349 |
| IL15RA | 0.57097 | 1.960575 | 5.78411 | 3.40E-08 | 3.52E-07 | 8.38039 |
| TNIP2 | 0.57101 | 4.620787 | 5.82472 | 2.78E-08 | 2.95E-07 | 8.57536 |
| DARC | 0.57107 | 2.716032 | 3.47579 | 0.00065 | 0.001937 | -0.9763 |
| ALPK1 | 0.57116 | 2.081098 | 5.17553 | 6.32E-07 | 4.37E-06 | 5.5717 |
| DZIP1L | 0.57127 | 2.107778 | 6.26159 | 2.98E-09 | 4.39E-08 | 10.7286 |
| SLA | 0.57132 | 3.579679 | 3.91947 | 0.00013 | 0.000466 | 0.52987 |
| P4HA1 | 0.57196 | 4.630334 | 5.43673 | 1.85E-07 | 1.53E-06 | 6.75048 |
| GNLY | 0.57197 | 1.607417 | 3.73066 | 0.00026 | 0.000871 | -0.1299 |
| LBH | 0.572 | 3.846491 | 3.72242 | 0.00027 | 0.000895 | -0.158 |
| MMEL1 | 0.57202 | 1.297378 | 3.9943 | 9.63E-05 | 0.000361 | 0.79884 |
| TXNDC12 | 0.57202 | 5.098082 | 7.84935 | 4.38E-13 | 2.27E-11 | 19.2919 |
| CCDC81 | 0.57206 | 1.154557 | 6.41076 | 1.36E-09 | 2.22E-08 | 11.4861 |
| ELF4 | 0.57219 | 1.48708 | 5.55923 | 1.03E-07 | 9.07E-07 | 7.31732 |
| NCF1B | 0.57221 | 1.675064 | 5.06463 | 1.05E-06 | 6.83E-06 | 5.08392 |
| ATAD3A | 0.57245 | 4.045761 | 7.35071 | 7.80E-12 | 2.73E-10 | 16.4926 |
| POLQ | 0.57265 | 0.642497 | 7.76313 | 7.25E-13 | 3.53E-11 | 18.8016 |
| GDAP2 | 0.57277 | 2.367338 | 8.35136 | 2.22E-14 | 1.88E-12 | 22.1941 |
| HAT1 | 0.57293 | 5.078125 | 8.40173 | 1.64E-14 | 1.49E-12 | 22.4895 |
| C19orf48 | 0.57317 | 4.232348 | 6.84417 | 1.32E-10 | 3.05E-09 | 13.7469 |
| IGKV1-5 | 0.57321 | 0.692517 | 2.84186 | 0.00503 | 0.011691 | -2.8523 |
| DPY19L1 | 0.57351 | 4.449761 | 4.83102 | 3.00E-06 | 1.72E-05 | 4.08208 |
| BIN2 | 0.57359 | 3.116532 | 4.54971 | 1.02E-05 | 4.97E-05 | 2.92352 |
| HYAL2 | 0.57367 | 4.867927 | 6.07273 | 7.91E-09 | 1.01E-07 | 9.7855 |
| OLR1 | 0.57383 | 4.204922 | 2.73324 | 0.00693 | 0.015474 | -3.1397 |
| RP11-785H5.1 | 0.57388 | 3.514187 | 3.19741 | 0.00165 | 0.004411 | -1.8413 |
| NPC2 | 0.57389 | 7.047664 | 4.19826 | 4.32E-05 | 0.000178 | 1.55317 |
| PAFAH2 | 0.57404 | 2.945188 | 9.01767 | 3.82E-16 | 7.61E-14 | 26.1562 |
| NSUN7 | 0.57422 | 0.818202 | 5.33057 | 3.06E-07 | 2.34E-06 | 6.2664 |
| GORAB | 0.57433 | 3.680239 | 6.59535 | 5.09E-10 | 9.54E-09 | 12.4384 |
| SIGLEC10 | 0.57457 | 3.769627 | 4.15885 | 5.05E-05 | 0.000205 | 1.40503 |
| SLC38A6 | 0.57482 | 3.159127 | 7.86409 | 4.02E-13 | 2.11E-11 | 19.3759 |
| C1orf109 | 0.57502 | 4.306249 | 7.41816 | 5.31E-12 | 1.97E-10 | 16.8659 |
| TRNAU1AP | 0.57509 | 3.969548 | 8.10335 | 9.79E-14 | 6.37E-12 | 20.7505 |
| TTC23 | 0.57518 | 3.606338 | 7.55532 | 2.42E-12 | 9.81E-11 | 17.6304 |
| OSR2 | 0.57519 | 0.817975 | 3.95676 | 0.00011 | 0.00041 | 0.66339 |
| MAN1C1 | 0.57562 | 4.246678 | 2.98538 | 0.00325 | 0.00798 | -2.4571 |
| CCNJL | 0.57643 | 1.370323 | 6.6959 | 2.96E-10 | 6.02E-09 | 12.9638 |
| CDK11A | 0.57655 | 4.857029 | 7.05267 | 4.17E-11 | 1.15E-09 | 14.8643 |
| PSMC3IP | 0.57656 | 3.173664 | 7.83823 | 4.68E-13 | 2.41E-11 | 19.2285 |
| TMEM140 | 0.57688 | 3.928602 | 6.62438 | 4.36E-10 | 8.40E-09 | 12.5897 |
| GPR160 | 0.57705 | 1.933864 | 5.91994 | 1.72E-08 | 1.97E-07 | 9.03606 |
| CELSR1 | 0.57705 | 1.107515 | 4.33082 | 2.53E-05 | 0.000111 | 2.05973 |
| FKBP10 | 0.57708 | 5.787879 | 3.95577 | 0.00011 | 0.000411 | 0.65981 |
| FCGR1B | 0.57725 | 3.912815 | 3.27967 | 0.00126 | 0.003476 | -1.5923 |
| DONSON | 0.57743 | 3.828134 | 7.25309 | 1.36E-11 | 4.43E-10 | 15.9554 |
| ZDHHC12 | 0.57751 | 3.265451 | 4.96404 | 1.66E-06 | 1.02E-05 | 4.64821 |
| APBB1IP | 0.57767 | 3.918883 | 3.70576 | 0.00028 | 0.000945 | -0.2148 |
| LRRC46 | 0.57781 | 1.499066 | 5.86354 | 2.29E-08 | 2.51E-07 | 8.76261 |
| LYPLA1 | 0.57814 | 4.585774 | 7.87746 | 3.72E-13 | 1.98E-11 | 19.4522 |
| NOL11 | 0.57817 | 4.416431 | 8.71747 | 2.42E-15 | 3.08E-13 | 24.357 |
| LSM14A | 0.57818 | 6.085248 | 7.3011 | 1.03E-11 | 3.50E-10 | 16.2191 |
| SHC1 | 0.57827 | 4.894292 | 5.2343 | 4.81E-07 | 3.45E-06 | 5.83331 |
| ARHGEF19 | 0.5784 | 1.279496 | 5.75869 | 3.85E-08 | 3.90E-07 | 8.2588 |
| FAM132B | 0.57885 | 1.682768 | 4.36759 | 2.17E-05 | 9.74E-05 | 2.20245 |
| LPAR4 | 0.5791 | 1.77469 | 4.81728 | 3.19E-06 | 1.81E-05 | 4.02426 |
| MPZL2 | 0.57915 | 1.176528 | 4.42541 | 1.71E-05 | 7.87E-05 | 2.42888 |
| CMTM7 | 0.57933 | 2.792387 | 4.71833 | 4.92E-06 | 2.65E-05 | 3.61158 |
| TMEM67 | 0.57949 | 3.040626 | 5.82461 | 2.78E-08 | 2.95E-07 | 8.57484 |
| POLE | 0.57974 | 3.477447 | 4.88034 | 2.41E-06 | 1.42E-05 | 4.29063 |
| G0S2 | 0.5798 | 1.380487 | 2.78655 | 0.00593 | 0.013494 | -3 |
| FOSL1 | 0.57982 | 1.446056 | 4.01708 | 8.81E-05 | 0.000334 | 0.88157 |
| SREK1 | 0.57982 | 4.996568 | 7.35065 | 7.80E-12 | 2.73E-10 | 16.4923 |
| NEURL1B | 0.57997 | 3.068928 | 5.13603 | 7.59E-07 | 5.12E-06 | 5.39708 |
| PI4K2B | 0.57998 | 3.032921 | 8.53299 | 7.43E-15 | 7.72E-13 | 23.2626 |
| PTMS | 0.58021 | 8.89086 | 5.23518 | 4.79E-07 | 3.44E-06 | 5.83721 |
| NPHP1 | 0.58051 | 2.683958 | 6.44641 | 1.13E-09 | 1.89E-08 | 11.6688 |
| DHX15 | 0.58065 | 5.767503 | 9.84128 | 2.19E-18 | 1.40E-15 | 31.1921 |
| RP11-452F19.3 | 0.58067 | 3.294074 | 5.89697 | 1.93E-08 | 2.17E-07 | 8.92452 |
| MVP | 0.58069 | 4.975176 | 4.62887 | 7.24E-06 | 3.71E-05 | 3.24413 |
| STK38L | 0.58071 | 4.555522 | 5.77364 | 3.58E-08 | 3.67E-07 | 8.33027 |
| ZCCHC11 | 0.58089 | 4.741064 | 6.74829 | 2.23E-10 | 4.74E-09 | 13.2394 |
| ERI1 | 0.58097 | 2.873087 | 7.06812 | 3.83E-11 | 1.07E-09 | 14.9478 |
| CFH | 0.58101 | 2.734075 | 4.16909 | 4.85E-05 | 0.000198 | 1.44342 |
| RAB27A | 0.58135 | 2.414806 | 6.14874 | 5.35E-09 | 7.20E-08 | 10.1629 |
| EXOSC10 | 0.58151 | 5.134562 | 8.10753 | 9.55E-14 | 6.25E-12 | 20.7747 |
| RP11-524D16__A.3 | 0.58152 | 1.071948 | 3.67346 | 0.00032 | 0.001048 | -0.3243 |
| WSCD1 | 0.58174 | 5.202354 | 4.28372 | 3.06E-05 | 0.000132 | 1.87827 |
| KLHL17 | 0.58189 | 3.347886 | 5.77969 | 3.47E-08 | 3.58E-07 | 8.35923 |
| MRPL42 | 0.58189 | 4.767315 | 8.12842 | 8.44E-14 | 5.61E-12 | 20.8956 |
| FRMD8 | 0.58191 | 3.785957 | 7.57019 | 2.22E-12 | 9.14E-11 | 17.7137 |
| CA9 | 0.58194 | 1.308972 | 2.73189 | 0.00696 | 0.015523 | -3.1432 |
| FRMD6 | 0.582 | 3.245673 | 5.65952 | 6.29E-08 | 5.95E-07 | 7.78791 |
| CMTM1 | 0.58208 | 2.917334 | 5.38684 | 2.35E-07 | 1.86E-06 | 6.52214 |
| BTF3L4 | 0.58216 | 5.820683 | 9.42849 | 2.96E-17 | 1.03E-14 | 28.6515 |
| SMC2 | 0.58235 | 3.498128 | 7.48686 | 3.58E-12 | 1.38E-10 | 17.248 |
| GPR34 | 0.58256 | 4.862456 | 3.50787 | 0.00058 | 0.001757 | -0.8726 |
| FOLR1 | 0.58283 | 1.622901 | 3.81775 | 0.00019 | 0.000655 | 0.17106 |
| UBE2S | 0.58285 | 5.549575 | 5.89082 | 1.99E-08 | 2.23E-07 | 8.89467 |
| TRIM38 | 0.58287 | 1.983387 | 5.80559 | 3.05E-08 | 3.21E-07 | 8.48341 |
| ACPL2 | 0.58301 | 4.821747 | 5.22245 | 5.08E-07 | 3.61E-06 | 5.7804 |
| AC064875.2 | 0.58316 | 0.805366 | 3.96511 | 0.00011 | 0.000399 | 0.69342 |
| IGHA2 | 0.5833 | 0.935553 | 2.8237 | 0.00531 | 0.01225 | -2.9011 |
| NFKB2 | 0.58336 | 3.24078 | 5.83412 | 2.65E-08 | 2.83E-07 | 8.6206 |
| RGS19 | 0.58342 | 3.982168 | 5.82116 | 2.82E-08 | 2.99E-07 | 8.55824 |
| DISP1 | 0.58362 | 2.629005 | 6.12829 | 5.95E-09 | 7.88E-08 | 10.0611 |
| GPR183 | 0.58398 | 2.386167 | 3.84383 | 0.00017 | 0.0006 | 0.26229 |
| CHSY1 | 0.58399 | 3.891767 | 6.61971 | 4.47E-10 | 8.54E-09 | 12.5653 |
| CDC25B | 0.58403 | 5.105845 | 7.12654 | 2.76E-11 | 8.11E-10 | 15.2645 |
| FAM91A1 | 0.58421 | 4.176776 | 7.45159 | 4.39E-12 | 1.66E-10 | 17.0516 |
| CLEC18A | 0.58432 | 1.704336 | 4.6488 | 6.65E-06 | 3.45E-05 | 3.32549 |
| TRIP6 | 0.58436 | 5.033478 | 3.96598 | 0.00011 | 0.000397 | 0.69654 |
| ISL2 | 0.5844 | 0.620047 | 5.42554 | 1.95E-07 | 1.60E-06 | 6.69913 |
| SMAD4 | 0.58445 | 5.253445 | 5.9453 | 1.51E-08 | 1.77E-07 | 9.1596 |
| SPSB1 | 0.58471 | 4.002579 | 5.32412 | 3.16E-07 | 2.40E-06 | 6.23721 |
| AKAP2 | 0.58499 | 3.55485 | 4.98349 | 1.52E-06 | 9.40E-06 | 4.73194 |
| NMNAT3 | 0.5854 | 2.320448 | 3.61583 | 0.00039 | 0.001259 | -0.5176 |
| CHST9 | 0.58567 | 3.401926 | 2.48198 | 0.01403 | 0.02835 | -3.7649 |
| CTNNAL1 | 0.58596 | 3.608572 | 7.90812 | 3.10E-13 | 1.73E-11 | 19.6275 |
| NIP7 | 0.58603 | 3.75443 | 9.49771 | 1.91E-17 | 7.28E-15 | 29.0753 |
| NUDT1 | 0.58605 | 5.127371 | 7.28178 | 1.15E-11 | 3.84E-10 | 16.1129 |
| LIG1 | 0.5861 | 4.045681 | 6.69537 | 2.97E-10 | 6.03E-09 | 12.9611 |
| INPPL1 | 0.58637 | 6.36464 | 5.90239 | 1.88E-08 | 2.12E-07 | 8.95081 |
| CLEC2D | 0.58641 | 2.745499 | 6.03312 | 9.69E-09 | 1.20E-07 | 9.59006 |
| SUSD2 | 0.5865 | 1.506289 | 4.55336 | 1.00E-05 | 4.91E-05 | 2.9382 |
| LDLRAD3 | 0.58653 | 4.887567 | 5.59014 | 8.82E-08 | 7.96E-07 | 7.46174 |
| ZNF468 | 0.5873 | 2.305088 | 6.49712 | 8.61E-10 | 1.50E-08 | 11.9297 |
| TMEM218 | 0.58731 | 4.866712 | 7.15729 | 2.32E-11 | 7.03E-10 | 15.4318 |
| SLC34A2 | 0.58732 | 0.623786 | 5.73154 | 4.41E-08 | 4.38E-07 | 8.12934 |
| CD63 | 0.5879 | 9.146719 | 6.15272 | 5.24E-09 | 7.07E-08 | 10.1827 |
| PTBP3 | 0.58817 | 2.966149 | 7.85539 | 4.23E-13 | 2.20E-11 | 19.3263 |
| GALNT10 | 0.58833 | 4.504424 | 4.66841 | 6.11E-06 | 3.20E-05 | 3.40586 |
| ITK | 0.58851 | 0.809762 | 4.9523 | 1.75E-06 | 1.06E-05 | 4.59776 |
| TUBA1A | 0.58869 | 10.56789 | 6.5494 | 6.51E-10 | 1.18E-08 | 12.1999 |
| GPR82 | 0.58886 | 0.755402 | 6.20378 | 4.03E-09 | 5.65E-08 | 10.438 |
| PAXBP1 | 0.58892 | 4.798282 | 4.98811 | 1.49E-06 | 9.22E-06 | 4.75188 |
| CCDC89 | 0.58907 | 1.331041 | 5.17376 | 6.37E-07 | 4.40E-06 | 5.56383 |
| CD52 | 0.58915 | 2.920507 | 2.97044 | 0.0034 | 0.008316 | -2.4991 |
| MOB1A | 0.58921 | 4.702706 | 6.99852 | 5.64E-11 | 1.50E-09 | 14.5723 |
| ANKRD10-IT1 | 0.58945 | 1.962237 | 4.74853 | 4.32E-06 | 2.36E-05 | 3.73684 |
| AL163636.6 | 0.5896 | 0.958638 | 3.80057 | 0.0002 | 0.000692 | 0.11124 |
| CTGF | 0.58986 | 5.384048 | 3.18667 | 0.00171 | 0.004547 | -1.8734 |
| STIM2 | 0.59006 | 4.604097 | 7.51874 | 2.99E-12 | 1.18E-10 | 17.4258 |
| CCDC14 | 0.59019 | 4.86992 | 6.43002 | 1.23E-09 | 2.03E-08 | 11.5848 |
| CCT6A | 0.59022 | 6.712876 | 7.55878 | 2.37E-12 | 9.68E-11 | 17.6498 |
| CXCL11 | 0.59024 | 1.044834 | 4.5194 | 1.15E-05 | 5.57E-05 | 2.80192 |
| BATF | 0.59036 | 1.072966 | 5.06316 | 1.06E-06 | 6.86E-06 | 5.0775 |
| MEOX2 | 0.59043 | 0.853159 | 3.00651 | 0.00304 | 0.007544 | -2.3974 |
| FBP1 | 0.59051 | 1.929687 | 4.50695 | 1.22E-05 | 5.83E-05 | 2.75215 |
| ZWILCH | 0.59085 | 2.562058 | 7.14893 | 2.44E-11 | 7.31E-10 | 15.3863 |
| PARP10 | 0.59092 | 4.545899 | 5.42429 | 1.96E-07 | 1.61E-06 | 6.6934 |
| HMGN2P5 | 0.59136 | 4.252945 | 4.79539 | 3.52E-06 | 1.97E-05 | 3.9324 |
| TRIB2 | 0.59145 | 5.428379 | 4.33883 | 2.44E-05 | 0.000108 | 2.09074 |
| UACA | 0.59153 | 3.627981 | 5.68269 | 5.61E-08 | 5.40E-07 | 7.8974 |
| SKP2 | 0.59161 | 2.880153 | 8.9615 | 5.40E-16 | 9.73E-14 | 25.8179 |
| TICRR | 0.5917 | 1.380221 | 6.71206 | 2.71E-10 | 5.57E-09 | 13.0487 |
| ZMYM6NB | 0.59177 | 3.741085 | 5.81405 | 2.93E-08 | 3.09E-07 | 8.52406 |
| MYD88 | 0.59218 | 3.597574 | 4.43503 | 1.64E-05 | 7.61E-05 | 2.46676 |
| OS9 | 0.59239 | 7.366322 | 5.20566 | 5.50E-07 | 3.87E-06 | 5.70554 |
| MTBP | 0.59251 | 1.813215 | 8.27224 | 3.58E-14 | 2.81E-12 | 21.7315 |
| ADPRHL2 | 0.59274 | 4.640162 | 7.12553 | 2.78E-11 | 8.15E-10 | 15.2591 |
| IGLV1-51 | 0.59312 | 1.122662 | 3.22846 | 0.00149 | 0.004038 | -1.7479 |
| RPS6KA1 | 0.59327 | 3.53816 | 4.85827 | 2.66E-06 | 1.54E-05 | 4.19711 |
| IL15 | 0.59329 | 1.058772 | 6.20619 | 3.98E-09 | 5.59E-08 | 10.4501 |
| TEKT2 | 0.59361 | 1.976471 | 4.43088 | 1.67E-05 | 7.72E-05 | 2.45042 |
| IPO4 | 0.59407 | 3.602232 | 6.85931 | 1.21E-10 | 2.85E-09 | 13.8275 |
| SYNC | 0.59427 | 2.886758 | 4.13144 | 5.63E-05 | 0.000225 | 1.30265 |
| ARHGAP4 | 0.59458 | 4.14841 | 5.39123 | 2.30E-07 | 1.83E-06 | 6.54216 |
| HLA-E | 0.59482 | 7.647037 | 5.11489 | 8.36E-07 | 5.57E-06 | 5.30401 |
| TEX9 | 0.59484 | 2.836716 | 5.16709 | 6.57E-07 | 4.52E-06 | 5.53432 |
| COL8A2 | 0.5949 | 1.833567 | 4.31976 | 2.64E-05 | 0.000116 | 2.01697 |
| CRB2 | 0.59538 | 2.926403 | 3.12403 | 0.0021 | 0.005441 | -2.0587 |
| HIST2H4B | 0.5955 | 1.987572 | 5.46085 | 1.65E-07 | 1.38E-06 | 6.86137 |
| DYRK3 | 0.59559 | 2.257231 | 5.27103 | 4.05E-07 | 2.97E-06 | 5.99788 |
| SCARNA12 | 0.59585 | 1.62571 | 3.40567 | 0.00082 | 0.002395 | -1.2001 |
| EPHA2 | 0.59656 | 1.880031 | 4.81497 | 3.23E-06 | 1.82E-05 | 4.01454 |
| MFSD1 | 0.59684 | 5.408395 | 6.7995 | 1.69E-10 | 3.73E-09 | 13.51 |
| STK36 | 0.59685 | 5.087533 | 5.32223 | 3.19E-07 | 2.42E-06 | 6.22867 |
| SPATA18 | 0.5969 | 0.975036 | 4.33173 | 2.52E-05 | 0.000111 | 2.06324 |
| E2F3 | 0.59701 | 3.229181 | 7.78621 | 6.34E-13 | 3.17E-11 | 18.9325 |
| MIS18A | 0.5971 | 2.948324 | 8.14758 | 7.53E-14 | 5.06E-12 | 21.0066 |
| DPH5 | 0.59717 | 4.605472 | 8.80159 | 1.44E-15 | 2.05E-13 | 24.859 |
| HIST2H2BE | 0.59762 | 3.879263 | 4.58735 | 8.65E-06 | 4.33E-05 | 3.07544 |
| SMC6 | 0.59812 | 3.303231 | 7.23669 | 1.49E-11 | 4.78E-10 | 15.8655 |
| TSPAN6 | 0.59817 | 5.12742 | 5.42115 | 1.99E-07 | 1.63E-06 | 6.679 |
| TCEB3 | 0.59842 | 4.150537 | 8.66436 | 3.34E-15 | 4.02E-13 | 24.041 |
| CCDC80 | 0.59927 | 5.85979 | 3.35701 | 0.00097 | 0.002768 | -1.3531 |
| TRIM21 | 0.59928 | 3.243742 | 5.56842 | 9.81E-08 | 8.74E-07 | 7.36021 |
| ARHGAP15 | 0.59941 | 2.30512 | 5.25137 | 4.44E-07 | 3.22E-06 | 5.90967 |
| SOX2 | 0.59953 | 7.777359 | 4.94061 | 1.84E-06 | 1.11E-05 | 4.54764 |
| C5orf34 | 0.59969 | 1.430187 | 7.63744 | 1.51E-12 | 6.56E-11 | 18.0914 |
| NES | 0.59975 | 6.559054 | 3.21395 | 0.00157 | 0.004206 | -1.7917 |
| LRIG3 | 0.59984 | 2.419998 | 5.35182 | 2.77E-07 | 2.14E-06 | 6.36276 |
| PARP12 | 0.5999 | 3.324587 | 5.15067 | 7.09E-07 | 4.83E-06 | 5.46169 |
| RDX | 0.60054 | 6.672606 | 6.54093 | 6.82E-10 | 1.23E-08 | 12.156 |
| RP11-512H23.2 | 0.60059 | 0.643002 | 6.76255 | 2.06E-10 | 4.45E-09 | 13.3147 |
| RP11-626G11.3 | 0.60074 | 2.144585 | 4.66399 | 6.23E-06 | 3.25E-05 | 3.38774 |
| PNRC2 | 0.60082 | 5.608773 | 8.28564 | 3.30E-14 | 2.64E-12 | 21.8098 |
| DDX20 | 0.60092 | 2.693814 | 8.89902 | 7.94E-16 | 1.30E-13 | 25.4425 |
| ALOX5AP | 0.60108 | 4.824765 | 2.85016 | 0.00491 | 0.011449 | -2.83 |
| GSDMD | 0.60138 | 3.6623 | 4.73886 | 4.50E-06 | 2.45E-05 | 3.69666 |
| TNFRSF14 | 0.60162 | 3.8074 | 4.98569 | 1.51E-06 | 9.32E-06 | 4.74145 |
| STK32B | 0.60179 | 1.957465 | 4.31706 | 2.67E-05 | 0.000117 | 2.00654 |
| AC010441.1 | 0.60228 | 3.615197 | 4.10438 | 6.27E-05 | 0.000248 | 1.20216 |
| EFNB1 | 0.6023 | 3.166972 | 6.90284 | 9.56E-11 | 2.31E-09 | 14.0595 |
| LMO2 | 0.60248 | 5.428716 | 4.19861 | 4.31E-05 | 0.000178 | 1.55449 |
| H2AFZ | 0.60288 | 7.182272 | 7.44397 | 4.58E-12 | 1.72E-10 | 17.0093 |
| CEP89 | 0.60314 | 3.427291 | 6.98358 | 6.12E-11 | 1.60E-09 | 14.492 |
| DEF6 | 0.60321 | 2.833393 | 4.61817 | 7.58E-06 | 3.86E-05 | 3.20052 |
| RPF1 | 0.60328 | 4.62315 | 9.06809 | 2.79E-16 | 6.18E-14 | 26.4605 |
| RHOH | 0.60346 | 1.341451 | 5.89093 | 1.99E-08 | 2.23E-07 | 8.89522 |
| LINC00960 | 0.60347 | 1.576389 | 4.3225 | 2.61E-05 | 0.000115 | 2.02757 |
| LRRC36 | 0.60358 | 0.97799 | 6.45767 | 1.06E-09 | 1.80E-08 | 11.7266 |
| CARD9 | 0.60392 | 2.319712 | 5.81725 | 2.88E-08 | 3.04E-07 | 8.53941 |
| DUSP6 | 0.60403 | 4.674329 | 3.88214 | 0.00015 | 0.000528 | 0.39726 |
| APOL6 | 0.60444 | 2.620893 | 5.15364 | 7.00E-07 | 4.77E-06 | 5.47483 |
| CCDC135 | 0.60472 | 1.253999 | 4.80852 | 3.32E-06 | 1.87E-05 | 3.98745 |
| TBXAS1 | 0.60486 | 4.088984 | 4.83568 | 2.94E-06 | 1.69E-05 | 4.1017 |
| HIST2H2BF | 0.60491 | 2.2316 | 6.16708 | 4.87E-09 | 6.65E-08 | 10.2544 |
| HNRNPF | 0.60497 | 6.184223 | 9.5373 | 1.49E-17 | 6.05E-15 | 29.3182 |
| KIAA0226L | 0.60498 | 2.35367 | 4.61249 | 7.77E-06 | 3.95E-05 | 3.17741 |
| GSG2 | 0.60525 | 0.818857 | 6.66143 | 3.57E-10 | 7.08E-09 | 12.7832 |
| ZZZ3 | 0.60535 | 4.109694 | 8.29007 | 3.21E-14 | 2.61E-12 | 21.8356 |
| ANKRD10 | 0.60536 | 6.443758 | 7.25374 | 1.35E-11 | 4.42E-10 | 15.9589 |
| SNX20 | 0.60561 | 1.350029 | 5.19653 | 5.74E-07 | 4.01E-06 | 5.66492 |
| AC018730.1 | 0.60562 | 5.56428 | 5.13422 | 7.65E-07 | 5.16E-06 | 5.38911 |
| GBP1P1 | 0.6059 | 0.909782 | 5.23673 | 4.76E-07 | 3.42E-06 | 5.84415 |
| IL8 | 0.60595 | 1.830404 | 2.3985 | 0.01754 | 0.034394 | -3.9603 |
| SPPL2A | 0.606 | 4.206679 | 6.97738 | 6.34E-11 | 1.65E-09 | 14.4587 |
| HMGN4 | 0.60641 | 5.423783 | 8.78993 | 1.55E-15 | 2.18E-13 | 24.7893 |
| RP11-161H23.5 | 0.60663 | 1.606418 | 2.36305 | 0.01925 | 0.037256 | -4.0414 |
| SLC35F5 | 0.6067 | 4.176783 | 8.40951 | 1.57E-14 | 1.43E-12 | 22.5352 |
| PIK3R5 | 0.6068 | 2.615531 | 5.22667 | 4.99E-07 | 3.55E-06 | 5.79922 |
| NEDD4 | 0.60689 | 2.451115 | 5.57791 | 9.36E-08 | 8.39E-07 | 7.40454 |
| CD33 | 0.60695 | 2.782659 | 4.7973 | 3.49E-06 | 1.96E-05 | 3.94038 |
| NUP205 | 0.60697 | 4.442065 | 8.05472 | 1.31E-13 | 8.17E-12 | 20.4696 |
| H3F3A | 0.60712 | 8.629763 | 8.23386 | 4.50E-14 | 3.39E-12 | 21.5079 |
| RP11-262H14.1 | 0.60719 | 2.028118 | 5.24716 | 4.53E-07 | 3.28E-06 | 5.89083 |
| DSEL | 0.60749 | 3.461293 | 5.39146 | 2.30E-07 | 1.83E-06 | 6.54324 |
| RELL1 | 0.60789 | 3.092238 | 5.45359 | 1.71E-07 | 1.42E-06 | 6.82798 |
| B4GALT5 | 0.6079 | 4.925484 | 6.76978 | 1.98E-10 | 4.30E-09 | 13.3529 |
| UNC93B1 | 0.60792 | 3.805432 | 4.46629 | 1.44E-05 | 6.79E-05 | 2.59035 |
| SAT1 | 0.60814 | 7.881195 | 5.23577 | 4.78E-07 | 3.43E-06 | 5.83986 |
| RP3-460G2.2 | 0.60818 | 0.811438 | 3.54927 | 0.0005 | 0.00155 | -0.7376 |
| CTC-360G5.1 | 0.60823 | 1.465196 | 4.23669 | 3.70E-05 | 0.000156 | 1.69873 |
| HIST2H4A | 0.60847 | 2.979159 | 5.09947 | 8.98E-07 | 5.93E-06 | 5.23631 |
| PPP1R3B | 0.60866 | 1.951587 | 6.07601 | 7.78E-09 | 9.95E-08 | 9.80176 |
| PGD | 0.60874 | 6.31083 | 8.13207 | 8.26E-14 | 5.50E-12 | 20.9167 |
| LINC01057 | 0.6088 | 1.741825 | 4.52772 | 1.11E-05 | 5.40E-05 | 2.83525 |
| SSFA2 | 0.60901 | 6.006336 | 7.05611 | 4.09E-11 | 1.14E-09 | 14.8829 |
| KANK2 | 0.60911 | 4.390596 | 5.0073 | 1.37E-06 | 8.56E-06 | 4.83478 |
| ARHGEF39 | 0.60912 | 1.329968 | 6.91946 | 8.72E-11 | 2.15E-09 | 14.1483 |
| RPN2 | 0.60913 | 6.700716 | 9.22407 | 1.06E-16 | 2.97E-14 | 27.4054 |
| NUP107 | 0.60921 | 4.160281 | 8.26586 | 3.72E-14 | 2.89E-12 | 21.6943 |
| ZC3HAV1 | 0.60928 | 3.655368 | 7.19002 | 1.93E-11 | 5.99E-10 | 15.6103 |
| AKNAD1 | 0.60937 | 1.576859 | 5.35449 | 2.74E-07 | 2.12E-06 | 6.37489 |
| TXLNB | 0.60939 | 1.281801 | 4.31488 | 2.70E-05 | 0.000118 | 1.99816 |
| TM4SF18 | 0.60941 | 2.662486 | 5.44326 | 1.79E-07 | 1.48E-06 | 6.78049 |
| PYCARD | 0.60954 | 3.686897 | 4.47824 | 1.37E-05 | 6.49E-05 | 2.6378 |
| SPAG5 | 0.60974 | 3.385191 | 4.96407 | 1.66E-06 | 1.02E-05 | 4.64834 |
| WDR77 | 0.60993 | 4.31573 | 9.1106 | 2.15E-16 | 5.12E-14 | 26.7175 |
| RDH5 | 0.60996 | 2.86607 | 5.46381 | 1.62E-07 | 1.36E-06 | 6.87501 |
| BAX | 0.61009 | 6.387838 | 7.86738 | 3.94E-13 | 2.08E-11 | 19.3947 |
| COLEC12 | 0.61009 | 3.47339 | 4.14977 | 5.24E-05 | 0.000211 | 1.37106 |
| DCAF13 | 0.61086 | 4.739606 | 7.72802 | 8.90E-13 | 4.21E-11 | 18.6026 |
| MGAT2 | 0.61126 | 3.791728 | 7.68627 | 1.13E-12 | 5.14E-11 | 18.3666 |
| CLEC5A | 0.61133 | 1.061476 | 3.4949 | 0.0006 | 0.001828 | -0.9146 |
| MYO9B | 0.61186 | 5.647118 | 7.60387 | 1.83E-12 | 7.75E-11 | 17.9026 |
| CNN2 | 0.61193 | 3.658754 | 3.95292 | 0.00011 | 0.000415 | 0.64957 |
| BCL3 | 0.61193 | 2.69863 | 4.08335 | 6.81E-05 | 0.000266 | 1.1244 |
| HNRNPH1 | 0.61229 | 7.948905 | 7.22871 | 1.56E-11 | 4.97E-10 | 15.8218 |
| TMEM173 | 0.61235 | 3.667062 | 5.21314 | 5.31E-07 | 3.75E-06 | 5.73887 |
| MYL6 | 0.61275 | 9.823688 | 7.6332 | 1.54E-12 | 6.69E-11 | 18.0675 |
| NGFR | 0.61282 | 2.600901 | 3.18409 | 0.00173 | 0.004582 | -1.8811 |
| HILPDA | 0.61293 | 4.018579 | 4.03838 | 8.11E-05 | 0.000311 | 0.95925 |
| ATF5 | 0.61315 | 3.932052 | 5.18495 | 6.05E-07 | 4.21E-06 | 5.61347 |
| TMED7-TICAM2 | 0.61363 | 0.941829 | 5.6186 | 7.68E-08 | 7.07E-07 | 7.59521 |
| GNGT2 | 0.61366 | 1.875332 | 5.77974 | 3.47E-08 | 3.58E-07 | 8.35945 |
| CACNA2D4 | 0.61392 | 1.898514 | 4.97941 | 1.55E-06 | 9.55E-06 | 4.71437 |
| SP110 | 0.6144 | 3.961436 | 5.89631 | 1.94E-08 | 2.18E-07 | 8.92131 |
| SNRNP40 | 0.61446 | 4.61845 | 8.86159 | 9.99E-16 | 1.51E-13 | 25.218 |
| ZNF90 | 0.61457 | 1.55308 | 7.69827 | 1.06E-12 | 4.88E-11 | 18.4344 |
| FCGRT | 0.61465 | 6.295498 | 5.61177 | 7.94E-08 | 7.27E-07 | 7.56311 |
| LAMB2 | 0.61468 | 6.595452 | 5.41369 | 2.06E-07 | 1.68E-06 | 6.64484 |
| CCDC150 | 0.61516 | 1.544294 | 6.37428 | 1.65E-09 | 2.61E-08 | 11.2999 |
| PLOD3 | 0.61522 | 5.148426 | 6.28499 | 2.64E-09 | 3.92E-08 | 10.8467 |
| STC2 | 0.61532 | 2.664147 | 3.31414 | 0.00112 | 0.003142 | -1.4863 |
| GRN | 0.61552 | 6.544349 | 6.249 | 3.18E-09 | 4.64E-08 | 10.6652 |
| AIM1 | 0.61597 | 0.974432 | 5.6955 | 5.27E-08 | 5.10E-07 | 7.9581 |
| EFNA1 | 0.61601 | 4.500748 | 5.55917 | 1.03E-07 | 9.07E-07 | 7.31704 |
| SYK | 0.61611 | 2.906988 | 4.84286 | 2.85E-06 | 1.64E-05 | 4.132 |
| SPHK1 | 0.61613 | 2.737889 | 4.47978 | 1.36E-05 | 6.45E-05 | 2.6439 |
| NOP2 | 0.61616 | 4.706653 | 7.08468 | 3.49E-11 | 9.94E-10 | 15.0375 |
| PLK3 | 0.61631 | 3.351905 | 5.30565 | 3.44E-07 | 2.59E-06 | 6.15373 |
| HMGN1 | 0.6166 | 7.204567 | 7.40097 | 5.86E-12 | 2.14E-10 | 16.7706 |
| EGR1 | 0.61662 | 5.870414 | 2.4375 | 0.01581 | 0.031439 | -3.8698 |
| IFT81 | 0.61672 | 3.859757 | 7.80157 | 5.80E-13 | 2.91E-11 | 19.0198 |
| LCTL | 0.61694 | 1.156202 | 5.8694 | 2.22E-08 | 2.45E-07 | 8.79093 |
| SNRPG | 0.61722 | 6.371591 | 8.99755 | 4.32E-16 | 8.41E-14 | 26.0349 |
| SPC25 | 0.61751 | 2.598893 | 3.7638 | 0.00023 | 0.000782 | -0.016 |
| RAD18 | 0.61766 | 2.241755 | 7.57802 | 2.12E-12 | 8.81E-11 | 17.7576 |
| NID2 | 0.6177 | 3.177957 | 5.28286 | 3.83E-07 | 2.83E-06 | 6.05104 |
| PIK3R3 | 0.61772 | 3.582158 | 6.43629 | 1.19E-09 | 1.98E-08 | 11.6169 |
| RTP4 | 0.61779 | 2.184276 | 6.04401 | 9.17E-09 | 1.15E-07 | 9.64369 |
| PPP4C | 0.61821 | 5.968638 | 9.91827 | 1.34E-18 | 1.05E-15 | 31.6692 |
| OR2I1P | 0.6185 | 0.736678 | 4.49378 | 1.29E-05 | 6.12E-05 | 2.69964 |
| MCM6 | 0.61864 | 3.717492 | 7.8016 | 5.80E-13 | 2.91E-11 | 19.02 |
| NUP37 | 0.61895 | 3.412498 | 8.69765 | 2.73E-15 | 3.40E-13 | 24.239 |
| MYO1B | 0.619 | 3.340329 | 4.45401 | 1.52E-05 | 7.11E-05 | 2.54172 |
| RPAP2 | 0.61912 | 2.932748 | 8.23315 | 4.52E-14 | 3.39E-12 | 21.5038 |
| CISH | 0.61953 | 1.305711 | 5.14799 | 7.18E-07 | 4.88E-06 | 5.44986 |
| ZNF217 | 0.61969 | 2.411667 | 6.16101 | 5.02E-09 | 6.82E-08 | 10.2241 |
| ST8SIA4 | 0.61976 | 1.979849 | 5.96971 | 1.34E-08 | 1.59E-07 | 9.27886 |
| FZD6 | 0.61983 | 1.909375 | 5.3166 | 3.27E-07 | 2.48E-06 | 6.20318 |
| HPGDS | 0.61986 | 2.76289 | 4.49262 | 1.29E-05 | 6.15E-05 | 2.69499 |
| C4orf21 | 0.61989 | 2.047646 | 7.56527 | 2.28E-12 | 9.35E-11 | 17.6861 |
| TRIM14 | 0.61991 | 3.786181 | 5.93505 | 1.59E-08 | 1.84E-07 | 9.10964 |
| PPP4R1L | 0.62023 | 2.163012 | 7.15804 | 2.31E-11 | 7.02E-10 | 15.4359 |
| SIKE1 | 0.62115 | 4.220471 | 9.4297 | 2.93E-17 | 1.03E-14 | 28.6589 |
| RB1 | 0.6212 | 5.033771 | 7.43005 | 4.96E-12 | 1.85E-10 | 16.9319 |
| NMB | 0.62156 | 6.87915 | 2.50434 | 0.01321 | 0.026928 | -3.7116 |
| 15-Sep | 0.6216 | 7.022961 | 6.99852 | 5.64E-11 | 1.50E-09 | 14.5723 |
| ODF2 | 0.62163 | 4.393807 | 8.38354 | 1.83E-14 | 1.62E-12 | 22.3827 |
| APLN | 0.62167 | 4.94398 | 3.50781 | 0.00058 | 0.001757 | -0.8728 |
| IGFLR1 | 0.62169 | 3.391506 | 5.61908 | 7.66E-08 | 7.05E-07 | 7.59744 |
| KCNJ5 | 0.62204 | 1.835516 | 5.0854 | 9.57E-07 | 6.28E-06 | 5.17467 |
| CHPF2 | 0.62232 | 3.944642 | 6.88574 | 1.05E-10 | 2.51E-09 | 13.9682 |
| PSME2 | 0.62257 | 6.259104 | 7.27717 | 1.18E-11 | 3.92E-10 | 16.0876 |
| GPSM3 | 0.6226 | 3.857284 | 4.84261 | 2.85E-06 | 1.64E-05 | 4.13091 |
| UGCG | 0.62275 | 3.55677 | 6.1854 | 4.43E-09 | 6.13E-08 | 10.346 |
| TP53 | 0.62281 | 4.731501 | 4.57004 | 9.31E-06 | 4.61E-05 | 3.00544 |
| SPATA17 | 0.62311 | 1.30389 | 5.0221 | 1.28E-06 | 8.08E-06 | 4.89889 |
| ODC1 | 0.62365 | 6.111956 | 5.98516 | 1.24E-08 | 1.48E-07 | 9.35449 |
| LRRC25 | 0.62365 | 2.22375 | 4.8581 | 2.67E-06 | 1.55E-05 | 4.19638 |
| PHACTR4 | 0.6237 | 3.461321 | 8.51019 | 8.53E-15 | 8.64E-13 | 23.128 |
| FXYD5 | 0.62372 | 4.838751 | 4.40286 | 1.88E-05 | 8.55E-05 | 2.34031 |
| CBX3 | 0.62376 | 6.90727 | 7.49302 | 3.46E-12 | 1.35E-10 | 17.2823 |
| PITX1 | 0.62388 | 0.7358 | 4.80049 | 3.44E-06 | 1.93E-05 | 3.95377 |
| VAV1 | 0.62418 | 2.614312 | 5.13601 | 7.59E-07 | 5.12E-06 | 5.39697 |
| SEPN1 | 0.62424 | 5.808786 | 5.83226 | 2.67E-08 | 2.85E-07 | 8.61168 |
| DR1 | 0.62429 | 4.333998 | 8.04073 | 1.42E-13 | 8.74E-12 | 20.389 |
| ALG6 | 0.62464 | 2.958352 | 8.41983 | 1.47E-14 | 1.35E-12 | 22.5958 |
| CAPZA1 | 0.62472 | 5.114191 | 7.52721 | 2.84E-12 | 1.13E-10 | 17.4732 |
| PRPF40A | 0.62474 | 5.165926 | 7.64296 | 1.46E-12 | 6.41E-11 | 18.1224 |
| DHFR | 0.62479 | 4.893773 | 6.72918 | 2.47E-10 | 5.15E-09 | 13.1388 |
| PPIH | 0.62482 | 4.806197 | 9.15192 | 1.66E-16 | 4.20E-14 | 26.9676 |
| MSMP | 0.62515 | 0.894535 | 3.49258 | 0.00061 | 0.001841 | -0.9222 |
| RPS8 | 0.62539 | 9.749913 | 7.53393 | 2.74E-12 | 1.09E-10 | 17.5107 |
| SERBP1 | 0.62541 | 6.786577 | 8.39661 | 1.69E-14 | 1.53E-12 | 22.4594 |
| MNDA | 0.62618 | 3.341438 | 4.44081 | 1.60E-05 | 7.45E-05 | 2.48956 |
| CARHSP1 | 0.6262 | 5.358336 | 6.86575 | 1.17E-10 | 2.77E-09 | 13.8618 |
| TXNDC5 | 0.62634 | 5.029056 | 8.06257 | 1.25E-13 | 7.86E-12 | 20.5149 |
| TRAM1 | 0.62675 | 5.770394 | 6.95976 | 6.98E-11 | 1.78E-09 | 14.3641 |
| RP11-497H16.7 | 0.62679 | 1.971176 | 4.99822 | 1.42E-06 | 8.86E-06 | 4.79553 |
| CCRL2 | 0.62683 | 1.73947 | 5.90014 | 1.90E-08 | 2.14E-07 | 8.93988 |
| NCF1 | 0.62694 | 2.418339 | 4.56925 | 9.34E-06 | 4.63E-05 | 3.00226 |
| IER5L | 0.62724 | 3.060533 | 4.22052 | 3.95E-05 | 0.000165 | 1.63736 |
| NEK6 | 0.6273 | 5.005855 | 5.03056 | 1.23E-06 | 7.82E-06 | 4.9356 |
| HIST1H3E | 0.62734 | 1.042982 | 5.02904 | 1.24E-06 | 7.86E-06 | 4.929 |
| TWSG1 | 0.6275 | 4.181607 | 6.41005 | 1.37E-09 | 2.22E-08 | 11.4825 |
| BORA | 0.62754 | 1.737736 | 9.45414 | 2.52E-17 | 9.28E-15 | 28.8084 |
| RP11-421L21.3 | 0.62754 | 2.703353 | 6.81643 | 1.54E-10 | 3.48E-09 | 13.5997 |
| C4orf47 | 0.62782 | 2.115346 | 4.38952 | 1.98E-05 | 8.98E-05 | 2.28806 |
| FOSL2 | 0.62786 | 3.823256 | 3.99329 | 9.66E-05 | 0.000362 | 0.79519 |
| CCDC50 | 0.62801 | 4.547486 | 7.73013 | 8.79E-13 | 4.18E-11 | 18.6146 |
| A2M | 0.62812 | 7.607925 | 4.8536 | 2.72E-06 | 1.57E-05 | 4.17736 |
| FAM96A | 0.62821 | 5.349268 | 8.81032 | 1.37E-15 | 1.96E-13 | 24.9111 |
| AOAH | 0.62822 | 2.972295 | 5.59066 | 8.80E-08 | 7.94E-07 | 7.46417 |
| ARL4C | 0.62838 | 4.135567 | 3.5717 | 0.00046 | 0.001447 | -0.6638 |
| NOX4 | 0.6284 | 1.253803 | 4.87963 | 2.42E-06 | 1.42E-05 | 4.28762 |
| WDR78 | 0.62861 | 1.836875 | 4.69767 | 5.39E-06 | 2.87E-05 | 3.52623 |
| EMR2 | 0.62883 | 1.446042 | 5.72644 | 4.52E-08 | 4.47E-07 | 8.10505 |
| SLFN13 | 0.62891 | 1.646376 | 5.31455 | 3.30E-07 | 2.50E-06 | 6.19395 |
| GNS | 0.62895 | 5.07485 | 6.65722 | 3.65E-10 | 7.22E-09 | 12.7612 |
| C4orf33 | 0.62896 | 3.597603 | 8.75237 | 1.95E-15 | 2.60E-13 | 24.565 |
| SPATA6 | 0.62898 | 3.207363 | 4.20737 | 4.16E-05 | 0.000172 | 1.58757 |
| ARHGAP18 | 0.62917 | 2.575032 | 5.19859 | 5.68E-07 | 3.98E-06 | 5.67409 |
| PRIM2 | 0.62926 | 2.314933 | 10.1939 | 2.31E-19 | 4.33E-16 | 33.3847 |
| KLF6 | 0.62949 | 4.737188 | 5.32411 | 3.16E-07 | 2.40E-06 | 6.23714 |
| NAMPTL | 0.62966 | 1.763399 | 3.91507 | 0.00013 | 0.000473 | 0.51418 |
| F2RL1 | 0.62995 | 1.716234 | 4.49654 | 1.27E-05 | 6.06E-05 | 2.71063 |
| AC007620.3 | 0.63013 | 4.285449 | 4.95803 | 1.71E-06 | 1.04E-05 | 4.62236 |
| CD3D | 0.63094 | 1.074536 | 4.93291 | 1.91E-06 | 1.15E-05 | 4.5147 |
| LBR | 0.63267 | 4.357762 | 7.29669 | 1.06E-11 | 3.57E-10 | 16.1949 |
| RFC3 | 0.63311 | 2.838114 | 7.99306 | 1.88E-13 | 1.13E-11 | 20.1146 |
| ZNF300 | 0.63361 | 3.23813 | 4.68007 | 5.81E-06 | 3.06E-05 | 3.45375 |
| NUDT5 | 0.63369 | 5.276897 | 8.56943 | 5.96E-15 | 6.50E-13 | 23.4781 |
| AMICA1 | 0.63388 | 1.497272 | 4.78564 | 3.67E-06 | 2.04E-05 | 3.89156 |
| SMIM4 | 0.6344 | 4.423352 | 6.6207 | 4.44E-10 | 8.50E-09 | 12.5705 |
| TMPO | 0.6344 | 4.820528 | 6.95473 | 7.18E-11 | 1.82E-09 | 14.3371 |
| LAT2 | 0.63442 | 3.928918 | 4.26187 | 3.34E-05 | 0.000143 | 1.79466 |
| DTYMK | 0.63469 | 4.50742 | 8.46993 | 1.09E-14 | 1.07E-12 | 22.8906 |
| C1orf194 | 0.63477 | 3.528158 | 2.94244 | 0.00371 | 0.00896 | -2.5772 |
| LY86 | 0.63494 | 4.077839 | 3.95915 | 0.00011 | 0.000407 | 0.67199 |
| LAG3 | 0.63518 | 1.22589 | 5.84626 | 2.49E-08 | 2.69E-07 | 8.67916 |
| TRPM8 | 0.63537 | 0.918273 | 4.22719 | 3.85E-05 | 0.000161 | 1.66262 |
| RFTN1 | 0.6359 | 4.113098 | 3.98629 | 9.93E-05 | 0.000371 | 0.76985 |
| RP2 | 0.636 | 3.25803 | 7.74154 | 8.23E-13 | 3.94E-11 | 18.6792 |
| SBNO2 | 0.63609 | 3.576173 | 6.49025 | 8.93E-10 | 1.55E-08 | 11.8943 |
| TNFSF8 | 0.63613 | 1.556264 | 5.85336 | 2.40E-08 | 2.62E-07 | 8.71342 |
| RCN3 | 0.63615 | 3.183625 | 5.17588 | 6.31E-07 | 4.36E-06 | 5.57327 |
| IFNGR2 | 0.63652 | 5.265018 | 6.96793 | 6.68E-11 | 1.72E-09 | 14.408 |
| PTAR1 | 0.63666 | 3.882576 | 8.14503 | 7.64E-14 | 5.11E-12 | 20.9918 |
| FOXD1 | 0.63669 | 0.985834 | 5.07443 | 1.01E-06 | 6.56E-06 | 5.12672 |
| IL16 | 0.63688 | 2.610659 | 5.87609 | 2.15E-08 | 2.38E-07 | 8.82333 |
| DSCC1 | 0.63772 | 2.277743 | 8.74669 | 2.02E-15 | 2.67E-13 | 24.5311 |
| LRAT | 0.63788 | 1.369296 | 4.46365 | 1.46E-05 | 6.86E-05 | 2.57989 |
| ZMYM1 | 0.63792 | 2.290377 | 8.94822 | 5.86E-16 | 1.04E-13 | 25.7381 |
| GLA | 0.63828 | 4.02368 | 8.62904 | 4.14E-15 | 4.85E-13 | 23.8313 |
| S100PBP | 0.63845 | 3.694061 | 8.83273 | 1.19E-15 | 1.74E-13 | 25.0452 |
| CNTF | 0.63871 | 1.582036 | 5.39345 | 2.27E-07 | 1.81E-06 | 6.55233 |
| PTGER4 | 0.6388 | 1.689014 | 5.01526 | 1.32E-06 | 8.30E-06 | 4.86925 |
| NKIRAS2 | 0.63891 | 4.820828 | 7.1562 | 2.34E-11 | 7.06E-10 | 15.4259 |
| CYP21A1P | 0.63895 | 1.414559 | 5.19194 | 5.86E-07 | 4.08E-06 | 5.64454 |
| ETS1 | 0.63915 | 4.459001 | 4.66791 | 6.13E-06 | 3.20E-05 | 3.4038 |
| PROM1 | 0.63937 | 2.555367 | 4.5382 | 1.07E-05 | 5.19E-05 | 2.87726 |
| TMEM123 | 0.63941 | 6.302387 | 7.32793 | 8.88E-12 | 3.07E-10 | 16.3669 |
| ADPRH | 0.63944 | 2.043061 | 6.3314 | 2.07E-09 | 3.18E-08 | 11.0818 |
| POLA2 | 0.63949 | 3.414583 | 8.61437 | 4.53E-15 | 5.27E-13 | 23.7442 |
| SRGN | 0.63962 | 5.864 | 4.4658 | 1.45E-05 | 6.80E-05 | 2.58844 |
| DOCK11 | 0.63963 | 2.40358 | 7.26051 | 1.30E-11 | 4.28E-10 | 15.9961 |
| RARRES3 | 0.63984 | 5.750688 | 4.0451 | 7.91E-05 | 0.000304 | 0.98385 |
| EVI2B | 0.64009 | 3.726852 | 4.53834 | 1.07E-05 | 5.18E-05 | 2.87785 |
| MAP3K1 | 0.64014 | 2.673632 | 6.867 | 1.16E-10 | 2.76E-09 | 13.8684 |
| HNRNPAB | 0.64037 | 6.251443 | 8.96333 | 5.34E-16 | 9.69E-14 | 25.8289 |
| TNFRSF10B | 0.64044 | 4.079658 | 5.35864 | 2.68E-07 | 2.08E-06 | 6.39374 |
| NXT1 | 0.64066 | 3.758527 | 8.91408 | 7.24E-16 | 1.23E-13 | 25.5329 |
| SEC61A1 | 0.64076 | 6.654414 | 7.90139 | 3.23E-13 | 1.78E-11 | 19.589 |
| ZNF474 | 0.64115 | 0.81291 | 5.11809 | 8.24E-07 | 5.51E-06 | 5.31808 |
| CTTNBP2NL | 0.6412 | 3.432221 | 8.32502 | 2.61E-14 | 2.15E-12 | 22.0399 |
| KYNU | 0.64122 | 1.376867 | 5.63712 | 7.02E-08 | 6.55E-07 | 7.68228 |
| TES | 0.64135 | 2.004184 | 5.37028 | 2.54E-07 | 1.99E-06 | 6.44669 |
| SHKBP1 | 0.6414 | 4.388555 | 6.88779 | 1.04E-10 | 2.49E-09 | 13.9792 |
| KDELC1 | 0.64158 | 2.32059 | 7.61263 | 1.74E-12 | 7.40E-11 | 17.9518 |
| RBBP4 | 0.64195 | 6.655534 | 8.79807 | 1.48E-15 | 2.09E-13 | 24.8379 |
| GINS4 | 0.64196 | 1.959586 | 7.20551 | 1.77E-11 | 5.56E-10 | 15.6949 |
| ABCC1 | 0.64202 | 2.926077 | 7.55723 | 2.39E-12 | 9.73E-11 | 17.6411 |
| CLECL1 | 0.64202 | 1.123629 | 5.65856 | 6.32E-08 | 5.97E-07 | 7.78337 |
| CHTF18 | 0.64207 | 3.571284 | 6.13908 | 5.63E-09 | 7.52E-08 | 10.1148 |
| TNFAIP8L2 | 0.64213 | 2.858595 | 5.33378 | 3.02E-07 | 2.31E-06 | 6.28093 |
| DOCK2 | 0.64215 | 2.961342 | 5.37644 | 2.46E-07 | 1.94E-06 | 6.47474 |
| PLXNA3 | 0.64219 | 4.39607 | 6.99847 | 5.64E-11 | 1.50E-09 | 14.5721 |
| BMP1 | 0.64245 | 3.932118 | 5.88136 | 2.09E-08 | 2.32E-07 | 8.84881 |
| NCAPD3 | 0.64257 | 3.877304 | 7.1942 | 1.89E-11 | 5.88E-10 | 15.6331 |
| B3GNT5 | 0.64261 | 2.483664 | 4.72138 | 4.86E-06 | 2.62E-05 | 3.62421 |
| RP11-488L18.10 | 0.64265 | 2.55195 | 5.4459 | 1.77E-07 | 1.47E-06 | 6.79261 |
| RPL11 | 0.64278 | 10.12512 | 8.44729 | 1.25E-14 | 1.17E-12 | 22.7573 |
| SLC26A10 | 0.64286 | 3.471924 | 3.96517 | 0.00011 | 0.000399 | 0.69365 |
| ARID5A | 0.64311 | 4.168329 | 5.1544 | 6.97E-07 | 4.76E-06 | 5.47818 |
| AASS | 0.64327 | 4.170879 | 5.82471 | 2.78E-08 | 2.95E-07 | 8.57533 |
| RPA3 | 0.64336 | 4.448986 | 7.19317 | 1.90E-11 | 5.90E-10 | 15.6275 |
| USP1 | 0.64356 | 4.214685 | 8.98599 | 4.64E-16 | 8.96E-14 | 25.9653 |
| NT5DC2 | 0.64372 | 5.476423 | 5.40923 | 2.11E-07 | 1.70E-06 | 6.62442 |
| CTSH | 0.64385 | 6.70764 | 3.08504 | 0.00237 | 0.006078 | -2.1724 |
| TIFA | 0.64411 | 1.914301 | 6.02263 | 1.02E-08 | 1.25E-07 | 9.53842 |
| IL6 | 0.64434 | 1.302963 | 3.55109 | 0.0005 | 0.001542 | -0.7316 |
| HOXA6 | 0.64449 | 0.630373 | 4.57676 | 9.05E-06 | 4.51E-05 | 3.03259 |
| ADAP2 | 0.64454 | 3.641764 | 5.11721 | 8.27E-07 | 5.52E-06 | 5.31423 |
| RUNX3 | 0.64529 | 1.280644 | 7.18459 | 1.99E-11 | 6.16E-10 | 15.5807 |
| XAF1 | 0.64542 | 4.026215 | 4.42988 | 1.68E-05 | 7.75E-05 | 2.44646 |
| GALNT1 | 0.64554 | 5.261688 | 7.98318 | 2.00E-13 | 1.19E-11 | 20.0578 |
| DCLRE1B | 0.64569 | 2.530428 | 9.53129 | 1.55E-17 | 6.18E-15 | 29.2813 |
| HAUS5 | 0.64569 | 3.606391 | 6.69245 | 3.02E-10 | 6.10E-09 | 12.9457 |
| FADD | 0.6457 | 3.796286 | 8.6082 | 4.71E-15 | 5.40E-13 | 23.7077 |
| RPS17 | 0.64596 | 7.462369 | 2.92414 | 0.00392 | 0.009414 | -2.6279 |
| EML4 | 0.64611 | 3.560108 | 10.0019 | 7.88E-19 | 7.84E-16 | 32.1884 |
| IGKV3-11 | 0.64617 | 0.755405 | 3.12777 | 0.00207 | 0.005383 | -2.0477 |
| MMP19 | 0.64643 | 1.467977 | 3.7561 | 0.00024 | 0.000802 | -0.0425 |
| SLC26A2 | 0.64666 | 2.063063 | 6.83134 | 1.42E-10 | 3.24E-09 | 13.6788 |
| RP11-640M9.2 | 0.64691 | 3.459727 | 6.12467 | 6.06E-09 | 8.01E-08 | 10.0431 |
| SERPING1 | 0.64693 | 5.58911 | 3.44144 | 0.00073 | 0.002152 | -1.0865 |
| GYPC | 0.64701 | 4.734701 | 4.58546 | 8.72E-06 | 4.36E-05 | 3.06779 |
| PSRC1 | 0.64722 | 5.237799 | 4.22263 | 3.92E-05 | 0.000163 | 1.64536 |
| HIST1H2AC | 0.6474 | 4.327986 | 4.91665 | 2.05E-06 | 1.22E-05 | 4.44519 |
| VSIG4 | 0.64746 | 5.293889 | 3.2939 | 0.0012 | 0.003335 | -1.5486 |
| STK17A | 0.64775 | 4.067121 | 6.34698 | 1.91E-09 | 2.94E-08 | 11.1609 |
| JUNB | 0.64783 | 6.109001 | 3.1865 | 0.00171 | 0.004549 | -1.8739 |
| VKORC1 | 0.64809 | 5.717233 | 8.1037 | 9.77E-14 | 6.37E-12 | 20.7525 |
| TNFRSF1B | 0.64835 | 3.545092 | 5.24848 | 4.50E-07 | 3.26E-06 | 5.89675 |
| PRDX4 | 0.6491 | 5.833065 | 6.64346 | 3.93E-10 | 7.69E-09 | 12.6892 |
| TAGLN | 0.64939 | 5.900361 | 3.21243 | 0.00157 | 0.004222 | -1.7962 |
| PTRF | 0.64981 | 4.421664 | 3.81516 | 0.00019 | 0.00066 | 0.16203 |
| TCF7 | 0.64997 | 2.147291 | 5.93872 | 1.57E-08 | 1.81E-07 | 9.12751 |
| POLE2 | 0.65011 | 1.496783 | 7.03457 | 4.61E-11 | 1.26E-09 | 14.7666 |
| CASP2 | 0.65102 | 3.329017 | 7.61294 | 1.73E-12 | 7.40E-11 | 17.9536 |
| MMP11 | 0.65129 | 1.569522 | 4.13692 | 5.51E-05 | 0.000221 | 1.32308 |
| PRPF38B | 0.65136 | 4.690338 | 9.05015 | 3.12E-16 | 6.66E-14 | 26.3522 |
| UBALD2 | 0.65136 | 5.641081 | 6.62263 | 4.40E-10 | 8.45E-09 | 12.5805 |
| PALLD | 0.65153 | 5.319375 | 6.30364 | 2.39E-09 | 3.60E-08 | 10.9411 |
| ZYX | 0.6516 | 6.330055 | 4.99574 | 1.44E-06 | 8.94E-06 | 4.78483 |
| HOXA3 | 0.65163 | 0.674577 | 4.1402 | 5.44E-05 | 0.000219 | 1.33531 |
| MSH5 | 0.6522 | 3.463151 | 5.9639 | 1.38E-08 | 1.62E-07 | 9.25043 |
| HOXA-AS2 | 0.65229 | 0.531835 | 4.14139 | 5.42E-05 | 0.000218 | 1.33976 |
| ENG | 0.65257 | 4.7048 | 5.23085 | 4.89E-07 | 3.49E-06 | 5.8179 |
| IGKV1D-39 | 0.65288 | 1.048332 | 2.55939 | 0.01135 | 0.023636 | -3.5783 |
| ATAD2 | 0.65298 | 3.040569 | 7.44911 | 4.45E-12 | 1.67E-10 | 17.0378 |
| KIAA1524 | 0.65368 | 1.990047 | 8.25141 | 4.05E-14 | 3.09E-12 | 21.6101 |
| CXorf21 | 0.65431 | 1.497337 | 5.96916 | 1.34E-08 | 1.59E-07 | 9.27617 |
| MCL1 | 0.65432 | 6.59161 | 6.97914 | 6.27E-11 | 1.63E-09 | 14.4681 |
| LRRK2 | 0.65438 | 2.617609 | 4.52987 | 1.10E-05 | 5.36E-05 | 2.84385 |
| MARVELD1 | 0.65445 | 2.540149 | 4.77922 | 3.77E-06 | 2.09E-05 | 3.86474 |
| SOCS2 | 0.65451 | 3.51951 | 3.49443 | 0.00061 | 0.00183 | -0.9162 |
| PTP4A3 | 0.65461 | 4.764263 | 5.10156 | 8.89E-07 | 5.87E-06 | 5.2455 |
| DUSP10 | 0.65475 | 2.591331 | 5.88925 | 2.01E-08 | 2.25E-07 | 8.88707 |
| WDR34 | 0.65495 | 4.941469 | 8.45006 | 1.23E-14 | 1.16E-12 | 22.7735 |
| SNORD17 | 0.65569 | 2.269411 | 2.69814 | 0.00767 | 0.01688 | -3.2304 |
| ZNF28 | 0.65573 | 2.782298 | 7.33792 | 8.39E-12 | 2.92E-10 | 16.422 |
| RFWD3 | 0.65576 | 3.017653 | 9.27339 | 7.80E-17 | 2.29E-14 | 27.7053 |
| STXBP2 | 0.65608 | 3.632534 | 5.67059 | 5.95E-08 | 5.67E-07 | 7.8402 |
| ARHGAP25 | 0.65681 | 2.973319 | 6.22388 | 3.63E-09 | 5.18E-08 | 10.5389 |
| KDM4A | 0.65717 | 4.17879 | 8.58957 | 5.27E-15 | 5.91E-13 | 23.5973 |
| HS2ST1 | 0.65754 | 4.045435 | 6.99333 | 5.80E-11 | 1.53E-09 | 14.5445 |
| TP73 | 0.65755 | 1.068581 | 5.41824 | 2.02E-07 | 1.64E-06 | 6.66568 |
| CEBPD | 0.65767 | 4.544151 | 3.54983 | 0.0005 | 0.001548 | -0.7357 |
| DDX39A | 0.65798 | 5.708854 | 8.39404 | 1.72E-14 | 1.54E-12 | 22.4443 |
| SRSF10 | 0.65822 | 5.516552 | 10.7476 | 6.56E-21 | 3.19E-17 | 36.8616 |
| HSD3B7 | 0.6586 | 2.511865 | 5.995 | 1.18E-08 | 1.42E-07 | 9.40271 |
| SEC11A | 0.65881 | 6.630492 | 8.91195 | 7.33E-16 | 1.23E-13 | 25.5201 |
| REXO2 | 0.65902 | 5.073833 | 8.23081 | 4.58E-14 | 3.41E-12 | 21.4901 |
| S100A2 | 0.6593 | 2.094469 | 4.58136 | 8.88E-06 | 4.43E-05 | 3.05119 |
| RSPH4A | 0.65951 | 1.179743 | 5.04578 | 1.15E-06 | 7.36E-06 | 5.00176 |
| CLIC2 | 0.65982 | 2.200307 | 6.64845 | 3.83E-10 | 7.52E-09 | 12.7153 |
| LMCD1 | 0.65988 | 3.555863 | 5.53197 | 1.17E-07 | 1.02E-06 | 7.19041 |
| IGKV3-20 | 0.66002 | 1.117183 | 2.62045 | 0.00957 | 0.020428 | -3.4272 |
| CENPI | 0.66017 | 1.137142 | 8.70671 | 2.58E-15 | 3.27E-13 | 24.2929 |
| COMMD2 | 0.66023 | 4.684168 | 8.37496 | 1.93E-14 | 1.68E-12 | 22.3324 |
| CTBS | 0.66028 | 3.189336 | 6.06452 | 8.25E-09 | 1.04E-07 | 9.74493 |
| AFAP1L1 | 0.66034 | 2.288324 | 5.26324 | 4.20E-07 | 3.07E-06 | 5.96292 |
| LYN | 0.66078 | 3.831992 | 5.29192 | 3.67E-07 | 2.73E-06 | 6.09185 |
| VANGL1 | 0.66158 | 1.615481 | 8.34573 | 2.30E-14 | 1.93E-12 | 22.1611 |
| CD84 | 0.66165 | 2.698475 | 4.78424 | 3.69E-06 | 2.05E-05 | 3.88571 |
| NFE2L3 | 0.66166 | 2.091156 | 6.7679 | 2.00E-10 | 4.34E-09 | 13.3429 |
| ITGA1 | 0.66186 | 2.263086 | 4.74094 | 4.46E-06 | 2.43E-05 | 3.70528 |
| TRIM5 | 0.66207 | 3.034703 | 6.23554 | 3.41E-09 | 4.91E-08 | 10.5974 |
| ITPKC | 0.66208 | 3.171491 | 6.31434 | 2.26E-09 | 3.43E-08 | 10.9952 |
| TCF3 | 0.66209 | 5.667357 | 6.68015 | 3.22E-10 | 6.47E-09 | 12.8813 |
| TSPO | 0.66214 | 5.5704 | 5.01352 | 1.33E-06 | 8.35E-06 | 4.86171 |
| SMARCE1 | 0.66222 | 6.330756 | 8.68648 | 2.92E-15 | 3.59E-13 | 24.1725 |
| COLGALT1 | 0.66227 | 4.772132 | 6.89294 | 1.01E-10 | 2.42E-09 | 14.0066 |
| MREG | 0.66235 | 2.985374 | 5.24604 | 4.55E-07 | 3.29E-06 | 5.88582 |
| RIT1 | 0.66294 | 4.260233 | 8.54028 | 7.11E-15 | 7.58E-13 | 23.3057 |
| PTPN6 | 0.66309 | 3.930953 | 4.95896 | 1.70E-06 | 1.04E-05 | 4.62639 |
| HAS2-AS1 | 0.6631 | 1.732139 | 4.98068 | 1.54E-06 | 9.50E-06 | 4.71985 |
| IL1RN | 0.66315 | 1.233933 | 3.87964 | 0.00015 | 0.000532 | 0.38841 |
| IGJ | 0.66318 | 1.161412 | 3.37005 | 0.00093 | 0.002663 | -1.3123 |
| SLC2A10 | 0.66333 | 2.137577 | 5.33057 | 3.06E-07 | 2.34E-06 | 6.2664 |
| EHD4 | 0.66333 | 3.02868 | 6.15807 | 5.10E-09 | 6.89E-08 | 10.2094 |
| HBEGF | 0.6638 | 3.517534 | 4.8769 | 2.45E-06 | 1.44E-05 | 4.27602 |
| KLHL13 | 0.66418 | 3.407017 | 6.66267 | 3.54E-10 | 7.05E-09 | 12.7897 |
| TCEA3 | 0.6645 | 2.225225 | 4.29837 | 2.88E-05 | 0.000125 | 1.93455 |
| VASP | 0.66453 | 4.238628 | 6.03753 | 9.47E-09 | 1.18E-07 | 9.61179 |
| SECTM1 | 0.66533 | 1.964753 | 4.74145 | 4.45E-06 | 2.43E-05 | 3.70739 |
| UBA7 | 0.66533 | 5.174399 | 5.7453 | 4.12E-08 | 4.14E-07 | 8.19488 |
| PARP14 | 0.66538 | 3.607301 | 5.8018 | 3.11E-08 | 3.26E-07 | 8.46521 |
| TRAF3IP3 | 0.66569 | 2.261779 | 6.3743 | 1.65E-09 | 2.61E-08 | 11.3 |
| HPSE | 0.66579 | 1.935543 | 5.86433 | 2.28E-08 | 2.50E-07 | 8.7664 |
| CCDC102A | 0.66637 | 2.219284 | 7.23791 | 1.48E-11 | 4.76E-10 | 15.8722 |
| SRSF11 | 0.66664 | 6.543057 | 7.89381 | 3.38E-13 | 1.83E-11 | 19.5456 |
| IRF8 | 0.6667 | 3.497848 | 4.81336 | 3.25E-06 | 1.83E-05 | 4.00776 |
| ANG | 0.66672 | 1.877923 | 4.95043 | 1.77E-06 | 1.07E-05 | 4.58975 |
| DDX12P | 0.6672 | 1.564679 | 6.12219 | 6.14E-09 | 8.09E-08 | 10.0307 |
| TRDC | 0.66726 | 1.021665 | 4.03717 | 8.15E-05 | 0.000312 | 0.95484 |
| SVIL | 0.66737 | 2.138625 | 5.23889 | 4.71E-07 | 3.39E-06 | 5.85382 |
| NDC1 | 0.66741 | 2.810808 | 9.58857 | 1.08E-17 | 4.74E-15 | 29.6331 |
| AGAP2-AS1 | 0.66744 | 1.827849 | 3.5002 | 0.00059 | 0.001799 | -0.8975 |
| HTRA3 | 0.6679 | 1.091975 | 4.66167 | 6.29E-06 | 3.28E-05 | 3.3782 |
| MILR1 | 0.66798 | 2.325379 | 4.83601 | 2.94E-06 | 1.68E-05 | 4.1031 |
| CFD | 0.66812 | 2.503995 | 4.47453 | 1.39E-05 | 6.59E-05 | 2.62304 |
| PCED1B-AS1 | 0.66835 | 3.951027 | 3.96449 | 0.00011 | 0.000399 | 0.69118 |
| VMP1 | 0.66842 | 5.685894 | 6.50662 | 8.19E-10 | 1.44E-08 | 11.9787 |
| GPRC5A | 0.66853 | 1.141325 | 3.78264 | 0.00021 | 0.000735 | 0.04906 |
| MAGOH | 0.66864 | 5.112671 | 8.57299 | 5.83E-15 | 6.41E-13 | 23.4991 |
| PHTF1 | 0.66879 | 3.189174 | 7.20533 | 1.77E-11 | 5.56E-10 | 15.6939 |
| ITPRIPL2 | 0.66887 | 2.653234 | 6.72743 | 2.50E-10 | 5.19E-09 | 13.1296 |
| TMEM37 | 0.66896 | 1.928635 | 6.32857 | 2.10E-09 | 3.21E-08 | 11.0674 |
| TMEM51 | 0.66911 | 3.143424 | 7.50634 | 3.21E-12 | 1.26E-10 | 17.3566 |
| TRIM34 | 0.66935 | 2.124557 | 6.30374 | 2.39E-09 | 3.60E-08 | 10.9416 |
| C6orf118 | 0.66978 | 1.236338 | 4.61765 | 7.60E-06 | 3.87E-05 | 3.1984 |
| SCIMP | 0.66992 | 2.081061 | 5.2288 | 4.94E-07 | 3.52E-06 | 5.80874 |
| HOXD10 | 0.67002 | 0.777407 | 3.51637 | 0.00056 | 0.001711 | -0.845 |
| CNIH4 | 0.67019 | 4.454488 | 9.59564 | 1.03E-17 | 4.72E-15 | 29.6765 |
| HK2 | 0.67061 | 3.020044 | 4.43404 | 1.65E-05 | 7.63E-05 | 2.46285 |
| RPS19 | 0.67069 | 10.22505 | 8.52525 | 7.79E-15 | 8.02E-13 | 23.2168 |
| HOXD4 | 0.67184 | 0.964041 | 4.68023 | 5.81E-06 | 3.06E-05 | 3.45441 |
| CENPL | 0.67191 | 2.364172 | 9.89401 | 1.57E-18 | 1.12E-15 | 31.5187 |
| MX2 | 0.67219 | 2.300623 | 4.42275 | 1.73E-05 | 7.94E-05 | 2.41842 |
| PCDH18 | 0.67277 | 2.752124 | 5.57775 | 9.37E-08 | 8.39E-07 | 7.4038 |
| FAM81B | 0.67283 | 0.964981 | 4.18364 | 4.58E-05 | 0.000188 | 1.49808 |
| MPEG1 | 0.67308 | 2.939779 | 5.11822 | 8.24E-07 | 5.51E-06 | 5.31865 |
| CROT | 0.67315 | 3.776482 | 7.03248 | 4.67E-11 | 1.27E-09 | 14.7553 |
| RN7SL138P | 0.67339 | 1.885783 | 4.84787 | 2.79E-06 | 1.61E-05 | 4.15312 |
| TTF2 | 0.67341 | 2.437919 | 7.79876 | 5.89E-13 | 2.96E-11 | 19.0038 |
| PIK3AP1 | 0.67341 | 2.893288 | 5.41101 | 2.09E-07 | 1.69E-06 | 6.6326 |
| UGDH | 0.67369 | 3.951616 | 6.44386 | 1.14E-09 | 1.91E-08 | 11.6557 |
| AC093673.5 | 0.67389 | 3.163435 | 5.68414 | 5.57E-08 | 5.36E-07 | 7.90426 |
| SZRD1 | 0.67523 | 5.625992 | 8.23405 | 4.49E-14 | 3.39E-12 | 21.5089 |
| 9-Mar | 0.67576 | 4.41489 | 4.39404 | 1.95E-05 | 8.83E-05 | 2.30574 |
| CYP27B1 | 0.67656 | 1.117915 | 4.51656 | 1.17E-05 | 5.63E-05 | 2.79056 |
| H2AFX | 0.67705 | 5.77387 | 7.74183 | 8.21E-13 | 3.94E-11 | 18.6808 |
| CARD8 | 0.6771 | 4.469876 | 7.91804 | 2.93E-13 | 1.65E-11 | 19.6842 |
| PTGS1 | 0.67713 | 3.02364 | 4.41709 | 1.77E-05 | 8.10E-05 | 2.39613 |
| SF3A3 | 0.67738 | 5.58087 | 8.86181 | 9.98E-16 | 1.51E-13 | 25.2194 |
| RAB13 | 0.67777 | 6.627661 | 5.94552 | 1.51E-08 | 1.76E-07 | 9.16069 |
| TLR7 | 0.67822 | 2.134635 | 5.2314 | 4.88E-07 | 3.49E-06 | 5.82036 |
| MAN2B1 | 0.6783 | 4.997569 | 7.11909 | 2.88E-11 | 8.40E-10 | 15.2241 |
| CHAF1A | 0.67889 | 3.205365 | 7.1766 | 2.09E-11 | 6.41E-10 | 15.5371 |
| KDM1A | 0.67904 | 5.475129 | 7.21007 | 1.73E-11 | 5.43E-10 | 15.7198 |
| CASP3 | 0.67904 | 4.230981 | 7.23121 | 1.53E-11 | 4.92E-10 | 15.8355 |
| NAA38 | 0.67905 | 4.580075 | 8.52356 | 7.87E-15 | 8.04E-13 | 23.2069 |
| LTBR | 0.67944 | 3.479922 | 5.40306 | 2.17E-07 | 1.75E-06 | 6.59621 |
| DRAM2 | 0.67961 | 5.094259 | 8.73529 | 2.17E-15 | 2.83E-13 | 24.4632 |
| PTK7 | 0.67963 | 4.119393 | 6.57862 | 5.57E-10 | 1.03E-08 | 12.3515 |
| CDC25A | 0.68061 | 1.57484 | 6.59165 | 5.19E-10 | 9.70E-09 | 12.4192 |
| STAC3 | 0.68123 | 2.122424 | 7.0254 | 4.85E-11 | 1.32E-09 | 14.7171 |
| RNFT1 | 0.68151 | 3.41477 | 8.89123 | 8.33E-16 | 1.35E-13 | 25.3957 |
| HIST1H2AE | 0.68159 | 1.676307 | 5.3373 | 2.97E-07 | 2.28E-06 | 6.29689 |
| CNTRL | 0.68175 | 2.897313 | 9.97356 | 9.44E-19 | 8.20E-16 | 32.0124 |
| ALOX5 | 0.68188 | 3.480762 | 4.68774 | 5.62E-06 | 2.97E-05 | 3.4853 |
| CDT1 | 0.68215 | 2.373843 | 5.31067 | 3.36E-07 | 2.54E-06 | 6.17642 |
| TP53INP1 | 0.68238 | 3.277325 | 7.99725 | 1.84E-13 | 1.11E-11 | 20.1387 |
| NAT1 | 0.68238 | 1.529131 | 7.35365 | 7.67E-12 | 2.71E-10 | 16.5088 |
| SIGLEC9 | 0.6831 | 2.176232 | 5.16726 | 6.57E-07 | 4.52E-06 | 5.53506 |
| PHC2 | 0.68331 | 6.493209 | 8.07377 | 1.17E-13 | 7.38E-12 | 20.5795 |
| TRIM24 | 0.68374 | 4.473858 | 6.74759 | 2.24E-10 | 4.75E-09 | 13.2358 |
| DDOST | 0.684 | 6.315798 | 9.52194 | 1.64E-17 | 6.45E-15 | 29.2239 |
| GNL2 | 0.68414 | 4.512314 | 8.18731 | 5.94E-14 | 4.20E-12 | 21.2371 |
| GGH | 0.68416 | 3.850737 | 6.85083 | 1.27E-10 | 2.96E-09 | 13.7823 |
| TMED5 | 0.68419 | 5.099016 | 7.2372 | 1.48E-11 | 4.78E-10 | 15.8683 |
| HAPLN3 | 0.68453 | 2.176936 | 5.85867 | 2.34E-08 | 2.56E-07 | 8.73908 |
| KDELC2 | 0.68459 | 2.970651 | 5.83603 | 2.62E-08 | 2.81E-07 | 8.62985 |
| WDR3 | 0.68471 | 3.401846 | 7.1748 | 2.11E-11 | 6.47E-10 | 15.5272 |
| CCNE2 | 0.6848 | 2.643639 | 6.1436 | 5.50E-09 | 7.37E-08 | 10.1373 |
| TRMT13 | 0.68492 | 3.208181 | 8.8652 | 9.77E-16 | 1.50E-13 | 25.2397 |
| JUN | 0.68549 | 6.508865 | 4.52236 | 1.14E-05 | 5.51E-05 | 2.81375 |
| DEK | 0.68562 | 5.656803 | 9.16968 | 1.49E-16 | 3.89E-14 | 27.0753 |
| SLFN12 | 0.68576 | 1.674793 | 6.83515 | 1.39E-10 | 3.18E-09 | 13.699 |
| C1orf112 | 0.68587 | 2.207691 | 9.93729 | 1.19E-18 | 9.64E-16 | 31.7872 |
| BAZ1A | 0.68634 | 3.413813 | 7.41727 | 5.34E-12 | 1.97E-10 | 16.861 |
| BRIP1 | 0.68656 | 0.830166 | 8.38451 | 1.82E-14 | 1.61E-12 | 22.3884 |
| CCDC77 | 0.68657 | 3.102207 | 8.45884 | 1.16E-14 | 1.12E-12 | 22.8253 |
| CPXM1 | 0.68663 | 4.705785 | 3.22123 | 0.00153 | 0.004121 | -1.7697 |
| HELZ2 | 0.68666 | 3.067938 | 6.09683 | 6.99E-09 | 9.07E-08 | 9.90484 |
| KDELR2 | 0.68675 | 5.655354 | 8.51746 | 8.16E-15 | 8.31E-13 | 23.1709 |
| C3AR1 | 0.68694 | 4.142438 | 4.15308 | 5.17E-05 | 0.000209 | 1.38342 |
| IBSP | 0.68697 | 0.936232 | 2.90086 | 0.00421 | 0.010004 | -2.692 |
| AEBP1 | 0.68748 | 5.431486 | 2.74804 | 0.00664 | 0.014893 | -3.1012 |
| EIF4EBP1 | 0.68769 | 4.832405 | 5.21447 | 5.28E-07 | 3.73E-06 | 5.7448 |
| HMGN2 | 0.68769 | 8.181606 | 8.53653 | 7.27E-15 | 7.66E-13 | 23.2835 |
| EVA1B | 0.68798 | 3.602761 | 5.54576 | 1.09E-07 | 9.62E-07 | 7.25456 |
| MPZL1 | 0.68801 | 5.923383 | 6.43232 | 1.22E-09 | 2.01E-08 | 11.5966 |
| RP11-472N13.3 | 0.68828 | 2.110719 | 4.68032 | 5.81E-06 | 3.06E-05 | 3.4548 |
| NFKBIZ | 0.68845 | 2.315847 | 5.01423 | 1.32E-06 | 8.33E-06 | 4.86481 |
| ELK3 | 0.68847 | 3.427154 | 6.11777 | 6.28E-09 | 8.25E-08 | 10.0087 |
| AC002456.2 | 0.68898 | 1.858444 | 4.77203 | 3.90E-06 | 2.15E-05 | 3.8347 |
| SQRDL | 0.68928 | 3.024989 | 5.34904 | 2.81E-07 | 2.17E-06 | 6.35016 |
| DESI2 | 0.68933 | 3.619297 | 9.4932 | 1.97E-17 | 7.37E-15 | 29.0477 |
| C5AR1 | 0.69017 | 2.4749 | 4.10096 | 6.35E-05 | 0.000251 | 1.18949 |
| ACTA2 | 0.69037 | 5.42833 | 3.55921 | 0.00048 | 0.001504 | -0.7049 |
| RACGAP1 | 0.69123 | 3.8874 | 6.39433 | 1.49E-09 | 2.39E-08 | 11.4022 |
| CXCR4 | 0.69139 | 4.457265 | 4.22643 | 3.86E-05 | 0.000162 | 1.65976 |
| RNF135 | 0.69153 | 2.763269 | 5.46977 | 1.58E-07 | 1.33E-06 | 6.90248 |
| SNORD3B-2 | 0.69179 | 0.847815 | 4.44894 | 1.55E-05 | 7.24E-05 | 2.52166 |
| FGFBP2 | 0.69203 | 1.46881 | 4.17593 | 4.72E-05 | 0.000193 | 1.4691 |
| FANCA | 0.69212 | 2.988082 | 6.80975 | 1.59E-10 | 3.57E-09 | 13.5643 |
| TNFAIP2 | 0.69226 | 4.300228 | 4.22865 | 3.82E-05 | 0.00016 | 1.66819 |
| CD151 | 0.69249 | 6.23741 | 6.07931 | 7.65E-09 | 9.81E-08 | 9.81806 |
| STC1 | 0.69252 | 0.943824 | 4.02 | 8.72E-05 | 0.000331 | 0.89219 |
| RP11-495P10.2 | 0.69264 | 2.397128 | 3.25568 | 0.00136 | 0.003733 | -1.6655 |
| GMFG | 0.69265 | 4.535339 | 5.72165 | 4.63E-08 | 4.56E-07 | 8.08225 |
| ANXA5 | 0.69274 | 7.723535 | 6.39311 | 1.50E-09 | 2.40E-08 | 11.396 |
| HMG20B | 0.69294 | 5.728257 | 8.25118 | 4.06E-14 | 3.09E-12 | 21.6088 |
| BACE2 | 0.69305 | 3.028246 | 4.9113 | 2.10E-06 | 1.25E-05 | 4.42236 |
| PPP1R18 | 0.69339 | 5.089812 | 6.44291 | 1.15E-09 | 1.92E-08 | 11.6508 |
| CBX2 | 0.69396 | 2.439265 | 5.63658 | 7.03E-08 | 6.57E-07 | 7.67975 |
| ELN | 0.694 | 5.034733 | 4.38318 | 2.04E-05 | 9.20E-05 | 2.26326 |
| C1orf158 | 0.69433 | 0.846051 | 4.21072 | 4.11E-05 | 0.00017 | 1.60024 |
| RP11-783K16.5 | 0.6944 | 1.361341 | 4.88371 | 2.38E-06 | 1.40E-05 | 4.30494 |
| SNRPB | 0.69446 | 6.118112 | 9.31312 | 6.09E-17 | 1.85E-14 | 27.9472 |
| OASL | 0.69484 | 1.539101 | 4.95019 | 1.77E-06 | 1.07E-05 | 4.58873 |
| IL10RB | 0.69498 | 3.870337 | 8.94506 | 5.98E-16 | 1.05E-13 | 25.7191 |
| ENAH | 0.69505 | 6.160805 | 5.99641 | 1.17E-08 | 1.41E-07 | 9.40964 |
| CYTIP | 0.69506 | 1.818335 | 6.29348 | 2.52E-09 | 3.77E-08 | 10.8897 |
| CYTH4 | 0.69543 | 3.223649 | 5.07071 | 1.02E-06 | 6.66E-06 | 5.11045 |
| LRRC17 | 0.69546 | 3.23608 | 4.31474 | 2.70E-05 | 0.000118 | 1.99762 |
| BRCA1 | 0.6959 | 2.579187 | 8.1584 | 7.06E-14 | 4.80E-12 | 21.0693 |
| ARPC5 | 0.69603 | 6.335345 | 8.23271 | 4.53E-14 | 3.39E-12 | 21.5011 |
| SH3GLB1 | 0.69622 | 5.276078 | 8.06215 | 1.25E-13 | 7.86E-12 | 20.5125 |
| PFN1 | 0.69626 | 8.220854 | 7.85901 | 4.14E-13 | 2.17E-11 | 19.347 |
| CNPY4 | 0.69638 | 4.469988 | 8.04823 | 1.36E-13 | 8.41E-12 | 20.4322 |
| RP4-668G5.1 | 0.69651 | 2.713836 | 3.95939 | 0.00011 | 0.000407 | 0.67283 |
| NADK | 0.697 | 5.081068 | 9.14454 | 1.74E-16 | 4.32E-14 | 26.9229 |
| HMCN2 | 0.69812 | 1.425005 | 3.62494 | 0.00038 | 0.001222 | -0.4872 |
| RECQL4 | 0.69813 | 3.19521 | 6.3144 | 2.26E-09 | 3.43E-08 | 10.9956 |
| RN7SL731P | 0.69849 | 2.539051 | 3.79202 | 0.00021 | 0.000712 | 0.08156 |
| BTN3A3 | 0.69888 | 4.294371 | 6.48972 | 8.96E-10 | 1.55E-08 | 11.8915 |
| CTC-429P9.4 | 0.6991 | 2.548675 | 2.84201 | 0.00503 | 0.011688 | -2.8519 |
| LHFPL2 | 0.6996 | 4.119595 | 5.60806 | 8.09E-08 | 7.39E-07 | 7.54572 |
| RTKN2 | 0.70054 | 1.315996 | 7.16598 | 2.21E-11 | 6.74E-10 | 15.4792 |
| CENPE | 0.70071 | 1.164981 | 6.17933 | 4.57E-09 | 6.29E-08 | 10.3156 |
| FAS | 0.70073 | 2.838233 | 4.58517 | 8.73E-06 | 4.36E-05 | 3.06662 |
| CTD-2521M24.9 | 0.7011 | 1.999856 | 5.38721 | 2.34E-07 | 1.86E-06 | 6.52383 |
| MYO1F | 0.70159 | 3.773027 | 5.38993 | 2.31E-07 | 1.84E-06 | 6.53624 |
| PGAM2 | 0.70163 | 3.435653 | 3.18983 | 0.00169 | 0.004509 | -1.8639 |
| BGN | 0.70183 | 5.34997 | 3.64025 | 0.00036 | 0.001165 | -0.436 |
| ACP5 | 0.70191 | 2.344753 | 4.30729 | 2.78E-05 | 0.000121 | 1.96889 |
| HOXC10 | 0.70223 | 0.929533 | 3.53501 | 0.00052 | 0.001617 | -0.7842 |
| NRAS | 0.70255 | 4.311792 | 10.1607 | 2.86E-19 | 4.97E-16 | 33.1775 |
| IL2RG | 0.70284 | 1.928414 | 5.62522 | 7.44E-08 | 6.89E-07 | 7.6263 |
| HIST1H4H | 0.70301 | 2.058711 | 6.08149 | 7.57E-09 | 9.71E-08 | 9.82885 |
| XRN2 | 0.70323 | 4.85163 | 9.66967 | 6.48E-18 | 3.35E-15 | 30.1322 |
| RSAD2 | 0.70336 | 2.759522 | 4.5543 | 9.96E-06 | 4.89E-05 | 2.94199 |
| TMEM71 | 0.70343 | 1.362547 | 5.32708 | 3.11E-07 | 2.37E-06 | 6.2506 |
| INPP5D | 0.70371 | 4.539146 | 4.58003 | 8.93E-06 | 4.45E-05 | 3.04582 |
| THBS1 | 0.70372 | 2.494998 | 3.40707 | 0.00082 | 0.002385 | -1.1957 |
| FABP5 | 0.70413 | 5.055273 | 2.48382 | 0.01396 | 0.028235 | -3.7605 |
| DSE | 0.70419 | 3.035608 | 7.02302 | 4.92E-11 | 1.33E-09 | 14.7043 |
| LOXL3 | 0.70421 | 3.506222 | 6.19403 | 4.24E-09 | 5.90E-08 | 10.3891 |
| CD3E | 0.70507 | 1.245265 | 5.38964 | 2.32E-07 | 1.84E-06 | 6.53492 |
| IL10RA | 0.70515 | 2.986504 | 5.48111 | 1.50E-07 | 1.26E-06 | 6.95484 |
| RNASE4 | 0.70562 | 2.520903 | 5.13123 | 7.76E-07 | 5.22E-06 | 5.37591 |
| ADAMTS7 | 0.70594 | 1.235213 | 6.52409 | 7.46E-10 | 1.32E-08 | 12.0689 |
| SNX7 | 0.70595 | 4.224346 | 6.94011 | 7.79E-11 | 1.95E-09 | 14.2588 |
| DDR2 | 0.70615 | 3.637546 | 4.23639 | 3.71E-05 | 0.000156 | 1.69759 |
| MCM5 | 0.70643 | 4.402534 | 7.60289 | 1.84E-12 | 7.76E-11 | 17.8971 |
| TNFAIP6 | 0.70649 | 1.521055 | 4.42494 | 1.71E-05 | 7.88E-05 | 2.42704 |
| NEDD1 | 0.70652 | 3.217646 | 7.9153 | 2.98E-13 | 1.66E-11 | 19.6686 |
| SLC4A2 | 0.70653 | 5.570482 | 7.38536 | 6.40E-12 | 2.31E-10 | 16.6842 |
| NEIL3 | 0.70689 | 0.739738 | 8.37121 | 1.97E-14 | 1.71E-12 | 22.3104 |
| SLC30A7 | 0.70689 | 2.167007 | 9.23135 | 1.01E-16 | 2.87E-14 | 27.4496 |
| SCP2 | 0.70729 | 6.779744 | 8.75526 | 1.92E-15 | 2.58E-13 | 24.5823 |
| AR | 0.70793 | 1.771113 | 5.80469 | 3.07E-08 | 3.22E-07 | 8.47905 |
| POC1A | 0.70801 | 1.97013 | 7.38542 | 6.40E-12 | 2.31E-10 | 16.6845 |
| CTDSP2 | 0.7083 | 6.192753 | 6.40824 | 1.38E-09 | 2.24E-08 | 11.4733 |
| PTBP1 | 0.70842 | 6.316339 | 7.54281 | 2.60E-12 | 1.05E-10 | 17.5604 |
| COL6A3 | 0.70847 | 1.518257 | 3.89722 | 0.00014 | 0.000503 | 0.45072 |
| SLC15A3 | 0.70893 | 4.600591 | 5.01924 | 1.29E-06 | 8.17E-06 | 4.8865 |
| NASP | 0.70903 | 6.309215 | 8.96372 | 5.33E-16 | 9.69E-14 | 25.8313 |
| HAUS8 | 0.70923 | 2.787581 | 8.54457 | 6.93E-15 | 7.42E-13 | 23.331 |
| RNASE2 | 0.70924 | 1.853046 | 3.77696 | 0.00022 | 0.000748 | 0.02941 |
| PLA2G4A | 0.70925 | 2.190503 | 6.4526 | 1.09E-09 | 1.83E-08 | 11.7006 |
| IFIH1 | 0.70956 | 2.871022 | 6.4937 | 8.77E-10 | 1.53E-08 | 11.9121 |
| SLC43A3 | 0.70967 | 3.228175 | 4.42876 | 1.69E-05 | 7.78E-05 | 2.44208 |
| TCTEX1D1 | 0.70971 | 1.416372 | 3.31337 | 0.00113 | 0.003149 | -1.4887 |
| FCGR2B | 0.71008 | 1.950013 | 3.29546 | 0.00119 | 0.003321 | -1.5439 |
| MOV10 | 0.71046 | 4.498513 | 6.95897 | 7.01E-11 | 1.79E-09 | 14.3599 |
| RAD54B | 0.71055 | 2.01055 | 7.9538 | 2.37E-13 | 1.38E-11 | 19.8892 |
| PPIC | 0.71056 | 2.813765 | 5.43445 | 1.87E-07 | 1.54E-06 | 6.74002 |
| EMR1 | 0.71109 | 0.929915 | 5.41299 | 2.07E-07 | 1.68E-06 | 6.64162 |
| LXN | 0.71121 | 2.224345 | 5.03936 | 1.18E-06 | 7.55E-06 | 4.97386 |
| SEC24D | 0.71132 | 3.009692 | 6.6701 | 3.40E-10 | 6.79E-09 | 12.8286 |
| IL18BP | 0.71236 | 3.784264 | 6.32139 | 2.18E-09 | 3.33E-08 | 11.031 |
| MTMR11 | 0.7125 | 3.255247 | 6.13699 | 5.69E-09 | 7.59E-08 | 10.1044 |
| ACTL6A | 0.71258 | 4.701625 | 8.70004 | 2.69E-15 | 3.37E-13 | 24.2532 |
| MUC1 | 0.71272 | 3.028684 | 5.19439 | 5.79E-07 | 4.05E-06 | 5.65541 |
| OAS3 | 0.71365 | 3.455839 | 5.23881 | 4.71E-07 | 3.39E-06 | 5.85345 |
| E2F7 | 0.71366 | 0.855393 | 8.17543 | 6.38E-14 | 4.41E-12 | 21.1682 |
| LGALS9 | 0.71393 | 5.61214 | 4.6344 | 7.08E-06 | 3.64E-05 | 3.26667 |
| BTK | 0.71404 | 2.873226 | 5.38973 | 2.31E-07 | 1.84E-06 | 6.53531 |
| PBX3 | 0.71407 | 3.911592 | 6.06227 | 8.35E-09 | 1.05E-07 | 9.73381 |
| FPGT | 0.71417 | 2.8119 | 8.01905 | 1.61E-13 | 9.84E-12 | 20.2641 |
| CDCA4 | 0.71438 | 2.076554 | 8.8193 | 1.30E-15 | 1.86E-13 | 24.9648 |
| IL4I1 | 0.7144 | 1.401977 | 5.35236 | 2.76E-07 | 2.14E-06 | 6.36519 |
| RMI2 | 0.71456 | 2.107125 | 7.2598 | 1.31E-11 | 4.29E-10 | 15.9922 |
| DSN1 | 0.7148 | 2.939204 | 10.6303 | 1.40E-20 | 5.67E-17 | 36.1222 |
| POLD1 | 0.71569 | 3.337255 | 8.2648 | 3.74E-14 | 2.90E-12 | 21.6881 |
| LRRC55 | 0.71577 | 3.240526 | 4.101 | 6.35E-05 | 0.000251 | 1.18962 |
| LGALS3BP | 0.71593 | 6.900739 | 6.25636 | 3.06E-09 | 4.49E-08 | 10.7023 |
| CTSZ | 0.71629 | 6.149945 | 4.96649 | 1.64E-06 | 1.01E-05 | 4.65874 |
| C1orf54 | 0.71637 | 4.532479 | 5.79803 | 3.17E-08 | 3.32E-07 | 8.44713 |
| MIS18BP1 | 0.71709 | 2.736596 | 8.52466 | 7.81E-15 | 8.02E-13 | 23.2134 |
| IGHM | 0.71737 | 1.215157 | 3.3744 | 0.00092 | 0.002628 | -1.2987 |
| WDHD1 | 0.71752 | 1.866431 | 9.30895 | 6.25E-17 | 1.88E-14 | 27.9218 |
| HCK | 0.71756 | 3.53679 | 5.31618 | 3.28E-07 | 2.48E-06 | 6.2013 |
| SLC16A1 | 0.71766 | 5.242406 | 7.14116 | 2.54E-11 | 7.57E-10 | 15.344 |
| ADM | 0.71811 | 2.954205 | 3.44464 | 0.00072 | 0.00213 | -1.0762 |
| OSR1 | 0.71813 | 1.120672 | 4.62734 | 7.29E-06 | 3.73E-05 | 3.23787 |
| COL22A1 | 0.7182 | 2.133883 | 3.22823 | 0.00149 | 0.004039 | -1.7486 |
| BST1 | 0.71821 | 1.895792 | 6.9258 | 8.42E-11 | 2.09E-09 | 14.1822 |
| HAND2 | 0.71907 | 0.906908 | 4.2543 | 3.45E-05 | 0.000146 | 1.76577 |
| TTC26 | 0.71939 | 2.046129 | 7.70058 | 1.04E-12 | 4.83E-11 | 18.4474 |
| TEAD2 | 0.71944 | 2.551079 | 5.31221 | 3.34E-07 | 2.52E-06 | 6.18336 |
| TNFRSF1A | 0.71946 | 5.451821 | 4.92942 | 1.94E-06 | 1.16E-05 | 4.49974 |
| IL32 | 0.72009 | 4.744623 | 4.0253 | 8.54E-05 | 0.000325 | 0.91152 |
| NFIL3 | 0.72013 | 3.959668 | 6.58734 | 5.32E-10 | 9.87E-09 | 12.3968 |
| CITED1 | 0.72017 | 3.648243 | 4.21131 | 4.10E-05 | 0.00017 | 1.60247 |
| HLX | 0.7203 | 2.252427 | 6.12686 | 5.99E-09 | 7.93E-08 | 10.054 |
| CKLF | 0.72038 | 5.413849 | 9.5812 | 1.13E-17 | 4.83E-15 | 29.5877 |
| CD4 | 0.72042 | 4.50888 | 5.24888 | 4.49E-07 | 3.25E-06 | 5.89855 |
| NDE1 | 0.72085 | 4.133844 | 6.62263 | 4.40E-10 | 8.45E-09 | 12.5805 |
| FAM183A | 0.7209 | 0.958822 | 3.54245 | 0.00051 | 0.001581 | -0.7599 |
| TLR3 | 0.72098 | 2.355468 | 6.62107 | 4.43E-10 | 8.49E-09 | 12.5724 |
| RP11-145M9.4 | 0.72129 | 4.809068 | 7.13513 | 2.63E-11 | 7.79E-10 | 15.3113 |
| CKAP2 | 0.72142 | 3.859183 | 7.17706 | 2.08E-11 | 6.41E-10 | 15.5396 |
| ARAP3 | 0.72151 | 2.54844 | 5.08213 | 9.72E-07 | 6.36E-06 | 5.16037 |
| MLTK | 0.72153 | 3.063202 | 5.69747 | 5.22E-08 | 5.06E-07 | 7.96744 |
| KCNE3 | 0.72164 | 1.97486 | 5.7933 | 3.25E-08 | 3.39E-07 | 8.42441 |
| STIL | 0.72171 | 1.434597 | 9.11758 | 2.06E-16 | 5.00E-14 | 26.7597 |
| MNS1 | 0.72194 | 2.685882 | 7.65106 | 1.39E-12 | 6.14E-11 | 18.1681 |
| HOXD-AS2 | 0.722 | 1.044689 | 4.78758 | 3.64E-06 | 2.03E-05 | 3.89968 |
| NECAP2 | 0.7222 | 5.449165 | 7.58159 | 2.08E-12 | 8.64E-11 | 17.7776 |
| APOC2 | 0.72231 | 5.547955 | 3.59282 | 0.00043 | 0.001354 | -0.594 |
| CCDC18 | 0.72253 | 1.64389 | 8.60227 | 4.88E-15 | 5.54E-13 | 23.6726 |
| C1QTNF6 | 0.72324 | 2.456918 | 6.31171 | 2.29E-09 | 3.48E-08 | 10.9819 |
| LST1 | 0.72334 | 4.837922 | 5.373 | 2.51E-07 | 1.97E-06 | 6.45908 |
| SLC39A1 | 0.72352 | 5.685618 | 8.10155 | 9.90E-14 | 6.42E-12 | 20.7401 |
| AGTRAP | 0.72354 | 4.692718 | 6.20352 | 4.03E-09 | 5.65E-08 | 10.4367 |
| CD53 | 0.72388 | 5.225793 | 4.61812 | 7.59E-06 | 3.86E-05 | 3.20032 |
| FAM20C | 0.72466 | 4.872547 | 4.76363 | 4.04E-06 | 2.23E-05 | 3.79967 |
| ITGAL | 0.72487 | 2.346583 | 5.49696 | 1.39E-07 | 1.18E-06 | 7.02809 |
| HELLS | 0.72511 | 1.731338 | 7.25534 | 1.34E-11 | 4.38E-10 | 15.9677 |
| CEP135 | 0.72579 | 1.754561 | 9.74383 | 4.06E-18 | 2.41E-15 | 30.5896 |
| SLC4A7 | 0.72625 | 2.73269 | 8.21105 | 5.16E-14 | 3.80E-12 | 21.3751 |
| LOXL1 | 0.72631 | 2.100873 | 3.80598 | 0.0002 | 0.00068 | 0.13005 |
| FAM181A | 0.72652 | 2.471867 | 3.48848 | 0.00062 | 0.001863 | -0.9354 |
| CD86 | 0.727 | 3.026225 | 5.30283 | 3.49E-07 | 2.62E-06 | 6.141 |
| FSBP | 0.72703 | 1.525181 | 7.37435 | 6.82E-12 | 2.43E-10 | 16.6233 |
| CHRNA9 | 0.7275 | 0.885935 | 4.1326 | 5.61E-05 | 0.000225 | 1.30698 |
| FRMD3 | 0.72784 | 3.510507 | 4.96077 | 1.68E-06 | 1.03E-05 | 4.63417 |
| ZIC1 | 0.72785 | 4.828884 | 4.06016 | 7.45E-05 | 0.000288 | 1.03907 |
| TMEM194A | 0.7284 | 2.861097 | 9.61063 | 9.41E-18 | 4.58E-15 | 29.7687 |
| SNRPGP10 | 0.72859 | 4.549881 | 7.12344 | 2.81E-11 | 8.22E-10 | 15.2477 |
| ARHGAP30 | 0.7287 | 2.940936 | 6.30656 | 2.36E-09 | 3.55E-08 | 10.9559 |
| RPS27L | 0.72901 | 6.27127 | 6.49708 | 8.61E-10 | 1.50E-08 | 11.9294 |
| DOCK7 | 0.72958 | 4.229225 | 6.21665 | 3.77E-09 | 5.34E-08 | 10.5026 |
| PTGFRN | 0.72976 | 3.856632 | 5.13581 | 7.59E-07 | 5.13E-06 | 5.3961 |
| TRAIP | 0.72984 | 2.152771 | 7.19739 | 1.86E-11 | 5.78E-10 | 15.6505 |
| PKN2 | 0.73004 | 3.779383 | 9.59869 | 1.01E-17 | 4.72E-15 | 29.6953 |
| NEAT1 | 0.73029 | 6.050531 | 4.02007 | 8.71E-05 | 0.000331 | 0.89244 |
| COL27A1 | 0.73035 | 2.384049 | 5.22821 | 4.95E-07 | 3.53E-06 | 5.80608 |
| GPC2 | 0.73101 | 3.603353 | 4.71673 | 4.96E-06 | 2.67E-05 | 3.60495 |
| AHR | 0.73147 | 2.956387 | 5.6425 | 6.83E-08 | 6.41E-07 | 7.70761 |
| RECQL | 0.73148 | 4.089409 | 9.71523 | 4.86E-18 | 2.69E-15 | 30.413 |
| GZMA | 0.73166 | 1.16321 | 5.05671 | 1.09E-06 | 7.05E-06 | 5.04937 |
| IRX5 | 0.73179 | 0.81708 | 5.99237 | 1.19E-08 | 1.44E-07 | 9.38982 |
| PPP1R14B | 0.73194 | 6.856641 | 7.11714 | 2.91E-11 | 8.47E-10 | 15.2135 |
| S100A8 | 0.73258 | 3.260148 | 2.78954 | 0.00588 | 0.013392 | -2.9921 |
| RNASE6 | 0.73272 | 3.876074 | 5.88691 | 2.03E-08 | 2.27E-07 | 8.87573 |
| LIF | 0.73277 | 1.36166 | 4.04 | 8.06E-05 | 0.000309 | 0.96519 |
| SNHG3 | 0.73317 | 4.276181 | 3.84284 | 0.00017 | 0.000602 | 0.25884 |
| AIF1 | 0.73378 | 6.181035 | 4.56876 | 9.36E-06 | 4.63E-05 | 3.00029 |
| GLIS3 | 0.73415 | 3.541818 | 4.53122 | 1.10E-05 | 5.33E-05 | 2.84927 |
| ASAP3 | 0.73422 | 4.366824 | 5.73499 | 4.33E-08 | 4.31E-07 | 8.14574 |
| PIM1 | 0.73441 | 3.606291 | 5.91124 | 1.80E-08 | 2.04E-07 | 8.99381 |
| SIGLEC7 | 0.73461 | 1.748785 | 6.20002 | 4.11E-09 | 5.74E-08 | 10.4192 |
| SLC47A2 | 0.73526 | 1.505132 | 3.80594 | 0.0002 | 0.00068 | 0.1299 |
| IL1RAP | 0.73542 | 3.076469 | 4.39665 | 1.93E-05 | 8.75E-05 | 2.31598 |
| TANC1 | 0.73551 | 4.073277 | 8.18142 | 6.15E-14 | 4.30E-12 | 21.2029 |
| ARHGAP11B | 0.73589 | 1.633649 | 7.22568 | 1.58E-11 | 5.03E-10 | 15.8052 |
| YBX3 | 0.73602 | 5.274688 | 5.38259 | 2.39E-07 | 1.89E-06 | 6.50277 |
| SPI1 | 0.73682 | 4.661875 | 4.98023 | 1.54E-06 | 9.52E-06 | 4.71792 |
| BCL2L12 | 0.73692 | 2.477573 | 6.71295 | 2.70E-10 | 5.55E-09 | 13.0534 |
| DDX60L | 0.73813 | 2.698691 | 7.1063 | 3.09E-11 | 8.96E-10 | 15.1547 |
| FPR1 | 0.7382 | 3.641668 | 3.91023 | 0.00013 | 0.000481 | 0.49694 |
| RPS2 | 0.73825 | 10.04481 | 7.96371 | 2.24E-13 | 1.31E-11 | 19.946 |
| SLC16A4 | 0.73853 | 2.960792 | 5.20943 | 5.40E-07 | 3.81E-06 | 5.72235 |
| APOBEC3G | 0.73868 | 2.419589 | 5.79021 | 3.30E-08 | 3.44E-07 | 8.40963 |
| SNORD3B-1 | 0.7387 | 1.221953 | 4.49407 | 1.28E-05 | 6.12E-05 | 2.70077 |
| LIPG | 0.73876 | 2.154126 | 5.25582 | 4.35E-07 | 3.16E-06 | 5.92962 |
| SERINC2 | 0.73892 | 1.666317 | 4.92175 | 2.01E-06 | 1.20E-05 | 4.46697 |
| HAUS1 | 0.73923 | 4.208888 | 10.0543 | 5.64E-19 | 6.24E-16 | 32.5145 |
| TRIM22 | 0.73945 | 5.084688 | 5.14123 | 7.41E-07 | 5.02E-06 | 5.42003 |
| CD180 | 0.74004 | 1.852167 | 5.82299 | 2.80E-08 | 2.97E-07 | 8.56704 |
| HAVCR2 | 0.74007 | 3.718706 | 5.0646 | 1.05E-06 | 6.83E-06 | 5.08376 |
| STK38 | 0.74059 | 3.491708 | 10.2049 | 2.16E-19 | 4.33E-16 | 33.4534 |
| NCF4 | 0.74085 | 2.961169 | 6.30331 | 2.40E-09 | 3.60E-08 | 10.9394 |
| ZNF560 | 0.74103 | 1.175232 | 4.66857 | 6.11E-06 | 3.20E-05 | 3.40652 |
| CCNL2 | 0.74106 | 6.460286 | 7.05617 | 4.09E-11 | 1.14E-09 | 14.8832 |
| DOK3 | 0.74164 | 2.963151 | 6.14209 | 5.54E-09 | 7.41E-08 | 10.1297 |
| ITPRIPL1 | 0.74358 | 1.683942 | 5.82118 | 2.82E-08 | 2.99E-07 | 8.55835 |
| DCBLD2 | 0.74369 | 3.128492 | 7.06204 | 3.96E-11 | 1.10E-09 | 14.915 |
| COL15A1 | 0.74417 | 1.235701 | 5.43073 | 1.90E-07 | 1.56E-06 | 6.72294 |
| HOXD9 | 0.74419 | 0.821004 | 4.62949 | 7.23E-06 | 3.70E-05 | 3.24664 |
| SOAT1 | 0.74456 | 3.73744 | 7.55521 | 2.42E-12 | 9.81E-11 | 17.6298 |
| LMNB2 | 0.74507 | 4.322817 | 7.59251 | 1.95E-12 | 8.19E-11 | 17.8388 |
| RP11-61L23.2 | 0.74509 | 1.665118 | 4.37614 | 2.10E-05 | 9.43E-05 | 2.2358 |
| MEX3A | 0.74517 | 3.451085 | 4.44304 | 1.59E-05 | 7.39E-05 | 2.49839 |
| WLS | 0.74526 | 6.826958 | 6.00153 | 1.14E-08 | 1.38E-07 | 9.43476 |
| LCP1 | 0.74533 | 4.006412 | 5.69824 | 5.20E-08 | 5.04E-07 | 7.97109 |
| CYTL1 | 0.74589 | 3.062294 | 5.53484 | 1.15E-07 | 1.01E-06 | 7.20375 |
| C1RL | 0.74608 | 2.985247 | 4.63293 | 7.12E-06 | 3.66E-05 | 3.26067 |
| CTPS1 | 0.74608 | 3.995908 | 8.27505 | 3.52E-14 | 2.77E-12 | 21.7479 |
| SOX4 | 0.7464 | 5.744473 | 3.57869 | 0.00045 | 0.001415 | -0.6408 |
| TMSB4X | 0.74644 | 11.46548 | 6.21921 | 3.72E-09 | 5.29E-08 | 10.5154 |
| CTD-3049M7.1 | 0.74661 | 1.552345 | 2.82828 | 0.00524 | 0.012112 | -2.8888 |
| IQGAP1 | 0.74693 | 4.933636 | 5.78724 | 3.34E-08 | 3.48E-07 | 8.39538 |
| CCR5 | 0.74739 | 1.339655 | 5.68769 | 5.47E-08 | 5.28E-07 | 7.92107 |
| CASC5 | 0.74748 | 1.209233 | 7.37146 | 6.93E-12 | 2.46E-10 | 16.6073 |
| HOXB7 | 0.74783 | 1.393703 | 4.1373 | 5.50E-05 | 0.000221 | 1.32449 |
| IDH1 | 0.74874 | 5.726471 | 7.57704 | 2.13E-12 | 8.84E-11 | 17.7521 |
| WDR76 | 0.74904 | 2.44769 | 7.22915 | 1.55E-11 | 4.97E-10 | 15.8242 |
| BTN2A3P | 0.74915 | 1.50149 | 8.25191 | 4.04E-14 | 3.09E-12 | 21.613 |
| EPHB2 | 0.74981 | 3.059289 | 5.7085 | 4.94E-08 | 4.82E-07 | 8.01979 |
| KIF14 | 0.75019 | 0.844673 | 9.08947 | 2.45E-16 | 5.67E-14 | 26.5897 |
| FNBP1L | 0.75157 | 3.939601 | 5.31195 | 3.34E-07 | 2.52E-06 | 6.18218 |
| CRYZ | 0.7516 | 4.17554 | 7.04916 | 4.25E-11 | 1.17E-09 | 14.8453 |
| WNT5A | 0.752 | 2.941971 | 5.36678 | 2.58E-07 | 2.02E-06 | 6.43078 |
| BRCA2 | 0.75206 | 1.218056 | 8.17909 | 6.24E-14 | 4.34E-12 | 21.1894 |
| DDX11 | 0.75249 | 3.465813 | 6.08762 | 7.33E-09 | 9.44E-08 | 9.85919 |
| TCIRG1 | 0.75448 | 4.028911 | 6.02342 | 1.02E-08 | 1.25E-07 | 9.54231 |
| CAPS | 0.75508 | 4.374248 | 5.42033 | 2.00E-07 | 1.63E-06 | 6.67527 |
| TPM4 | 0.75562 | 6.885808 | 6.72692 | 2.50E-10 | 5.20E-09 | 13.1269 |
| CXCL9 | 0.75608 | 0.931502 | 5.43657 | 1.85E-07 | 1.53E-06 | 6.74974 |
| FKBP7 | 0.75627 | 2.993936 | 8.14724 | 7.54E-14 | 5.06E-12 | 21.0046 |
| HIST1H2BD | 0.7572 | 3.19886 | 5.61144 | 7.95E-08 | 7.28E-07 | 7.56159 |
| RBBP8 | 0.75747 | 3.697503 | 8.28643 | 3.28E-14 | 2.64E-12 | 21.8144 |
| RP3-428L16.2 | 0.75749 | 3.145138 | 4.81697 | 3.20E-06 | 1.81E-05 | 4.02295 |
| CENPN | 0.75774 | 2.939405 | 6.94775 | 7.46E-11 | 1.89E-09 | 14.2997 |
| KPNA2 | 0.75794 | 5.108523 | 8.38515 | 1.82E-14 | 1.61E-12 | 22.3922 |
| XRCC2 | 0.75802 | 1.263282 | 8.84371 | 1.12E-15 | 1.65E-13 | 25.1109 |
| LAPTM5 | 0.75867 | 6.840655 | 4.68643 | 5.65E-06 | 2.99E-05 | 3.4799 |
| EPB41 | 0.7604 | 3.551928 | 6.38541 | 1.56E-09 | 2.48E-08 | 11.3567 |
| LAP3 | 0.76059 | 6.171507 | 7.65541 | 1.36E-12 | 6.02E-11 | 18.1926 |
| C7orf57 | 0.7607 | 0.873035 | 4.42564 | 1.71E-05 | 7.86E-05 | 2.42977 |
| LIMA1 | 0.7609 | 5.620885 | 5.80302 | 3.09E-08 | 3.24E-07 | 8.47106 |
| CDH11 | 0.76096 | 5.146812 | 5.95484 | 1.44E-08 | 1.69E-07 | 9.20615 |
| RP4-794H19.4 | 0.76105 | 0.662676 | 5.18255 | 6.12E-07 | 4.25E-06 | 5.60284 |
| SLC16A3 | 0.76139 | 3.680673 | 4.77491 | 3.85E-06 | 2.13E-05 | 3.84674 |
| PTPN7 | 0.76207 | 1.440329 | 5.92234 | 1.70E-08 | 1.95E-07 | 9.04774 |
| CASP6 | 0.7622 | 2.911198 | 8.02515 | 1.56E-13 | 9.51E-12 | 20.2992 |
| AP001007.1 | 0.76234 | 2.127339 | 5.99545 | 1.17E-08 | 1.42E-07 | 9.40493 |
| CD2 | 0.76269 | 1.163329 | 5.86138 | 2.31E-08 | 2.53E-07 | 8.75217 |
| TFEC | 0.76287 | 1.827749 | 6.84478 | 1.32E-10 | 3.04E-09 | 13.7502 |
| GRB14 | 0.76362 | 1.45995 | 4.3697 | 2.15E-05 | 9.67E-05 | 2.21068 |
| KANSL1L | 0.76396 | 3.152534 | 7.07642 | 3.65E-11 | 1.03E-09 | 14.9928 |
| SDC1 | 0.76425 | 1.609251 | 5.77235 | 3.60E-08 | 3.69E-07 | 8.32406 |
| REST | 0.76452 | 2.526291 | 8.36215 | 2.08E-14 | 1.79E-12 | 22.2573 |
| LIMD1 | 0.76485 | 2.67532 | 5.82855 | 2.72E-08 | 2.90E-07 | 8.59382 |
| SERPINA5 | 0.76613 | 1.284179 | 3.79234 | 0.00021 | 0.000711 | 0.08264 |
| IGFBP5 | 0.76658 | 6.125731 | 3.97342 | 0.0001 | 0.000387 | 0.72338 |
| SLC1A5 | 0.7677 | 3.073177 | 5.4273 | 1.93E-07 | 1.59E-06 | 6.7072 |
| MYL12A | 0.7678 | 5.944865 | 5.97314 | 1.31E-08 | 1.56E-07 | 9.29563 |
| HN1L | 0.76793 | 3.904409 | 7.88045 | 3.65E-13 | 1.95E-11 | 19.4693 |
| AVIL | 0.76808 | 2.739116 | 5.59211 | 8.74E-08 | 7.91E-07 | 7.47096 |
| IGLV2-14 | 0.76827 | 1.111071 | 3.02565 | 0.00286 | 0.007157 | -2.3431 |
| ELTD1 | 0.7683 | 2.977629 | 6.03358 | 9.67E-09 | 1.20E-07 | 9.59232 |
| BMF | 0.7684 | 2.157509 | 6.85768 | 1.23E-10 | 2.87E-09 | 13.8188 |
| MEST | 0.76877 | 5.497567 | 4.88786 | 2.34E-06 | 1.37E-05 | 4.32257 |
| STK40 | 0.76888 | 4.20289 | 6.93715 | 7.91E-11 | 1.98E-09 | 14.243 |
| ABI3 | 0.76895 | 3.160977 | 5.82981 | 2.71E-08 | 2.88E-07 | 8.59985 |
| COL14A1 | 0.76942 | 1.676492 | 5.03773 | 1.19E-06 | 7.60E-06 | 4.96675 |
| LILRB1 | 0.76953 | 2.581569 | 5.53668 | 1.14E-07 | 9.98E-07 | 7.21234 |
| SPINK8 | 0.76955 | 0.937775 | 5.77676 | 3.52E-08 | 3.63E-07 | 8.34517 |
| FAM46A | 0.7696 | 2.17258 | 6.16071 | 5.03E-09 | 6.82E-08 | 10.2226 |
| SOX11 | 0.76999 | 2.871817 | 4.39144 | 1.97E-05 | 8.92E-05 | 2.29557 |
| LILRB4 | 0.77008 | 4.626071 | 4.53277 | 1.09E-05 | 5.30E-05 | 2.85549 |
| KIF18A | 0.77008 | 1.022442 | 7.71901 | 9.38E-13 | 4.39E-11 | 18.5516 |
| SASH3 | 0.77015 | 3.149979 | 5.39449 | 2.26E-07 | 1.81E-06 | 6.55705 |
| CD36 | 0.77052 | 1.744024 | 4.98048 | 1.54E-06 | 9.51E-06 | 4.71897 |
| SLC44A5 | 0.77065 | 2.525825 | 4.89771 | 2.23E-06 | 1.32E-05 | 4.36445 |
| GIMAP2 | 0.77081 | 3.075852 | 7.37507 | 6.79E-12 | 2.42E-10 | 16.6272 |
| LEPRE1 | 0.77113 | 3.696698 | 8.35932 | 2.12E-14 | 1.80E-12 | 22.2407 |
| AK2 | 0.77151 | 5.99171 | 10.2418 | 1.70E-19 | 4.33E-16 | 33.6838 |
| CLEC12A | 0.77165 | 0.898133 | 6.42945 | 1.23E-09 | 2.03E-08 | 11.5818 |
| TSPAN12 | 0.77223 | 4.172515 | 4.7003 | 5.32E-06 | 2.84E-05 | 3.53709 |
| ARPC1B | 0.7725 | 5.798019 | 5.75872 | 3.85E-08 | 3.90E-07 | 8.25892 |
| TNFAIP3 | 0.77331 | 2.856025 | 6.46761 | 1.01E-09 | 1.72E-08 | 11.7777 |
| PDCD1LG2 | 0.77429 | 1.156295 | 7.20947 | 1.73E-11 | 5.44E-10 | 15.7166 |
| CREB5 | 0.7744 | 3.720792 | 6.31151 | 2.30E-09 | 3.48E-08 | 10.9809 |
| RBM47 | 0.7747 | 2.168815 | 6.37296 | 1.66E-09 | 2.63E-08 | 11.2932 |
| TPST1 | 0.77478 | 5.223035 | 6.84362 | 1.32E-10 | 3.06E-09 | 13.744 |
| HDAC1 | 0.77518 | 5.034835 | 7.89641 | 3.33E-13 | 1.81E-11 | 19.5605 |
| LCP2 | 0.77563 | 4.13471 | 5.81365 | 2.93E-08 | 3.09E-07 | 8.5221 |
| LRR1 | 0.77645 | 2.408896 | 10.201 | 2.21E-19 | 4.33E-16 | 33.4292 |
| SP140L | 0.777 | 2.185487 | 6.84764 | 1.29E-10 | 3.01E-09 | 13.7654 |
| ARHGDIB | 0.77705 | 6.295091 | 5.90364 | 1.87E-08 | 2.11E-07 | 8.95689 |
| HCLS1 | 0.77743 | 4.839229 | 5.55636 | 1.04E-07 | 9.18E-07 | 7.30395 |
| NCKAP1L | 0.77754 | 3.449615 | 5.74409 | 4.14E-08 | 4.15E-07 | 8.18913 |
| SPRY1 | 0.7777 | 3.427493 | 4.45416 | 1.52E-05 | 7.10E-05 | 2.54232 |
| SAMSN1 | 0.77835 | 3.422481 | 5.05299 | 1.11E-06 | 7.15E-06 | 5.03317 |
| EFEMP1 | 0.77903 | 6.500237 | 3.56993 | 0.00046 | 0.001455 | -0.6697 |
| ENPEP | 0.77911 | 1.372064 | 6.09638 | 7.01E-09 | 9.08E-08 | 9.90263 |
| FCGR1C | 0.77946 | 2.687054 | 4.67887 | 5.84E-06 | 3.07E-05 | 3.44882 |
| RAB42 | 0.77982 | 1.414496 | 6.18014 | 4.55E-09 | 6.27E-08 | 10.3197 |
| RFC4 | 0.77995 | 4.231918 | 9.65391 | 7.16E-18 | 3.63E-15 | 30.0351 |
| SKA1 | 0.78023 | 0.996618 | 7.88319 | 3.59E-13 | 1.93E-11 | 19.485 |
| CLCF1 | 0.78031 | 1.157595 | 4.96609 | 1.65E-06 | 1.01E-05 | 4.65703 |
| RP11-347E10.1 | 0.78054 | 0.937369 | 5.62995 | 7.27E-08 | 6.75E-07 | 7.64855 |
| MCM7 | 0.7809 | 6.213522 | 6.60034 | 4.96E-10 | 9.32E-09 | 12.4644 |
| TXLNA | 0.78098 | 4.166785 | 8.95973 | 5.46E-16 | 9.77E-14 | 25.8073 |
| PRRX1 | 0.78229 | 5.412149 | 5.7176 | 4.72E-08 | 4.63E-07 | 8.06301 |
| SMC5 | 0.78255 | 3.595266 | 9.20187 | 1.22E-16 | 3.33E-14 | 27.2706 |
| NKG7 | 0.78259 | 1.727182 | 5.73586 | 4.32E-08 | 4.30E-07 | 8.14991 |
| DAB2 | 0.7827 | 4.288703 | 6.62372 | 4.37E-10 | 8.42E-09 | 12.5862 |
| PLEKHS1 | 0.78279 | 0.609002 | 5.74659 | 4.09E-08 | 4.12E-07 | 8.20102 |
| RAC2 | 0.78286 | 2.793571 | 5.54297 | 1.11E-07 | 9.73E-07 | 7.24158 |
| NETO2 | 0.78322 | 3.693765 | 4.59496 | 8.38E-06 | 4.22E-05 | 3.10626 |
| C11orf82 | 0.78357 | 1.468463 | 8.18207 | 6.13E-14 | 4.30E-12 | 21.2067 |
| PIFO | 0.7842 | 3.212428 | 4.12171 | 5.85E-05 | 0.000233 | 1.26647 |
| PLEKHA8P1 | 0.78436 | 2.077716 | 7.07588 | 3.67E-11 | 1.03E-09 | 14.9898 |
| METTL21B | 0.78453 | 2.724044 | 5.58996 | 8.83E-08 | 7.96E-07 | 7.46092 |
| MCM3 | 0.78485 | 4.633805 | 10.0912 | 4.46E-19 | 6.24E-16 | 32.7445 |
| CD97 | 0.78518 | 3.403825 | 6.2699 | 2.85E-09 | 4.22E-08 | 10.7705 |
| CYP21A2 | 0.7856 | 1.535245 | 6.22326 | 3.64E-09 | 5.19E-08 | 10.5358 |
| NRP1 | 0.78617 | 4.149699 | 5.25583 | 4.35E-07 | 3.16E-06 | 5.92968 |
| SYDE1 | 0.78777 | 3.32908 | 7.31793 | 9.39E-12 | 3.24E-10 | 16.3118 |
| IGF2BP2 | 0.78794 | 1.048186 | 4.75254 | 4.24E-06 | 2.32E-05 | 3.7535 |
| WWTR1 | 0.78813 | 4.534098 | 4.66175 | 6.29E-06 | 3.28E-05 | 3.37855 |
| TMEM106C | 0.78851 | 5.39636 | 8.37769 | 1.90E-14 | 1.66E-12 | 22.3484 |
| FAM114A1 | 0.78866 | 3.171046 | 5.76418 | 3.75E-08 | 3.81E-07 | 8.28504 |
| SGOL2 | 0.78873 | 1.857449 | 9.98405 | 8.83E-19 | 8.20E-16 | 32.0775 |
| LAIR1 | 0.79026 | 4.296208 | 4.8994 | 2.22E-06 | 1.31E-05 | 4.37166 |
| CLEC7A | 0.79062 | 2.736801 | 4.99085 | 1.47E-06 | 9.12E-06 | 4.76369 |
| CSTA | 0.79067 | 1.473407 | 4.93056 | 1.93E-06 | 1.16E-05 | 4.50462 |
| ACTN1 | 0.79153 | 5.158549 | 3.8635 | 0.00016 | 0.000561 | 0.33146 |
| CDC7 | 0.79172 | 2.918265 | 8.87062 | 9.45E-16 | 1.46E-13 | 25.2722 |
| GNB4 | 0.79193 | 4.447448 | 6.54582 | 6.64E-10 | 1.20E-08 | 12.1814 |
| RCAN1 | 0.79219 | 6.109936 | 4.8167 | 3.20E-06 | 1.81E-05 | 4.0218 |
| RP11-79P5.2 | 0.79238 | 1.980471 | 3.82995 | 0.00018 | 0.000629 | 0.21368 |
| LRRC42 | 0.79257 | 3.968097 | 9.63771 | 7.93E-18 | 3.94E-15 | 29.9353 |
| ITGB2 | 0.79271 | 5.24754 | 4.66126 | 6.30E-06 | 3.29E-05 | 3.37653 |
| CAV2 | 0.79309 | 3.174728 | 5.33114 | 3.05E-07 | 2.34E-06 | 6.26899 |
| CMTM6 | 0.7938 | 4.104453 | 9.16677 | 1.51E-16 | 3.92E-14 | 27.0577 |
| WDR62 | 0.79441 | 1.345126 | 7.69532 | 1.08E-12 | 4.91E-11 | 18.4177 |
| HLA-DQA2 | 0.79447 | 1.125245 | 4.62369 | 7.41E-06 | 3.78E-05 | 3.22302 |
| FOS | 0.79454 | 6.977721 | 3.36935 | 0.00093 | 0.002668 | -1.3145 |
| ADAM12 | 0.79464 | 1.391426 | 4.78576 | 3.67E-06 | 2.04E-05 | 3.8921 |
| ORC1 | 0.79474 | 0.992368 | 8.8765 | 9.12E-16 | 1.42E-13 | 25.3074 |
| FILIP1L | 0.7952 | 3.38575 | 5.0034 | 1.39E-06 | 8.69E-06 | 4.81792 |
| PGM2 | 0.79548 | 3.735173 | 9.89964 | 1.51E-18 | 1.12E-15 | 31.5536 |
| ZNF436 | 0.79566 | 3.884556 | 7.69823 | 1.06E-12 | 4.88E-11 | 18.4342 |
| KIRREL | 0.79578 | 2.87079 | 6.64764 | 3.84E-10 | 7.54E-09 | 12.7111 |
| MYCBP | 0.79618 | 2.725701 | 7.82731 | 4.99E-13 | 2.53E-11 | 19.1663 |
| NRM | 0.79622 | 3.377153 | 7.07627 | 3.66E-11 | 1.03E-09 | 14.9919 |
| VCL | 0.79626 | 4.187394 | 6.76861 | 1.99E-10 | 4.32E-09 | 13.3467 |
| FERMT3 | 0.79635 | 3.555059 | 5.93494 | 1.60E-08 | 1.84E-07 | 9.10912 |
| FCGR1A | 0.79637 | 5.223115 | 3.9234 | 0.00013 | 0.00046 | 0.54391 |
| CLEC2B | 0.79651 | 2.143598 | 6.15947 | 5.07E-09 | 6.86E-08 | 10.2164 |
| ISG20 | 0.79741 | 2.374849 | 5.29494 | 3.62E-07 | 2.70E-06 | 6.10546 |
| RPS2P5 | 0.79774 | 5.136816 | 5.5746 | 9.52E-08 | 8.51E-07 | 7.38907 |
| GAL3ST4 | 0.79776 | 4.228741 | 6.81245 | 1.57E-10 | 3.53E-09 | 13.5786 |
| HOXB2 | 0.79792 | 1.167374 | 4.69729 | 5.39E-06 | 2.87E-05 | 3.52467 |
| DPYD | 0.79832 | 2.576165 | 5.30202 | 3.50E-07 | 2.63E-06 | 6.13739 |
| AJUBA | 0.79833 | 1.906967 | 7.87249 | 3.83E-13 | 2.02E-11 | 19.4239 |
| REEP4 | 0.79866 | 3.36644 | 8.83208 | 1.20E-15 | 1.74E-13 | 25.0413 |
| E2F1 | 0.79945 | 2.851759 | 6.92886 | 8.28E-11 | 2.06E-09 | 14.1986 |
| CASP8 | 0.79979 | 2.399818 | 7.45842 | 4.22E-12 | 1.60E-10 | 17.0896 |
| NID1 | 0.79998 | 3.91059 | 4.78795 | 3.63E-06 | 2.03E-05 | 3.90126 |
| GAPT | 0.80046 | 1.582859 | 5.76336 | 3.77E-08 | 3.83E-07 | 8.28111 |
| S100A9 | 0.8006 | 4.187713 | 2.99248 | 0.00318 | 0.007831 | -2.4371 |
| CENPM | 0.80079 | 2.199647 | 6.2164 | 3.77E-09 | 5.34E-08 | 10.5013 |
| SHOX2 | 0.80086 | 0.874902 | 4.44785 | 1.56E-05 | 7.27E-05 | 2.51739 |
| HS3ST1 | 0.80091 | 2.100897 | 5.4207 | 2.00E-07 | 1.63E-06 | 6.67694 |
| TRAM2 | 0.80099 | 2.231639 | 7.82912 | 4.93E-13 | 2.51E-11 | 19.1766 |
| CDC25C | 0.80113 | 1.040391 | 8.28362 | 3.34E-14 | 2.66E-12 | 21.798 |
| NCAPG2 | 0.80117 | 3.315877 | 9.51904 | 1.67E-17 | 6.46E-15 | 29.2061 |
| IRF7 | 0.80123 | 3.603753 | 5.90311 | 1.87E-08 | 2.12E-07 | 8.95432 |
| PVT1 | 0.80137 | 1.939161 | 4.9048 | 2.17E-06 | 1.28E-05 | 4.39464 |
| C2 | 0.80143 | 3.251727 | 5.25713 | 4.32E-07 | 3.15E-06 | 5.93552 |
| CD300LF | 0.80208 | 1.657603 | 6.77533 | 1.92E-10 | 4.19E-09 | 13.3822 |
| PXDN | 0.80223 | 4.422478 | 5.24526 | 4.57E-07 | 3.30E-06 | 5.88234 |
| VAMP8 | 0.80382 | 4.737933 | 5.67637 | 5.79E-08 | 5.55E-07 | 7.86751 |
| HIST1H1C | 0.80462 | 3.828142 | 5.6047 | 8.22E-08 | 7.49E-07 | 7.52995 |
| PLOD1 | 0.80479 | 5.384702 | 6.63811 | 4.05E-10 | 7.87E-09 | 12.6613 |
| FAM20A | 0.80481 | 1.803 | 4.82373 | 3.10E-06 | 1.77E-05 | 4.05139 |
| MXRA5 | 0.80491 | 1.281793 | 5.85182 | 2.42E-08 | 2.63E-07 | 8.70599 |
| TCF19 | 0.80519 | 2.325866 | 8.8597 | 1.01E-15 | 1.52E-13 | 25.2067 |
| RHOC | 0.80587 | 7.584755 | 7.16706 | 2.20E-11 | 6.72E-10 | 15.485 |
| TGFB1I1 | 0.80653 | 3.779152 | 5.73922 | 4.24E-08 | 4.24E-07 | 8.1659 |
| TNFRSF11B | 0.80686 | 1.409929 | 4.75512 | 4.19E-06 | 2.30E-05 | 3.76424 |
| GBE1 | 0.80702 | 3.870017 | 7.14864 | 2.44E-11 | 7.31E-10 | 15.3847 |
| HERC5 | 0.8071 | 2.61765 | 5.81302 | 2.94E-08 | 3.10E-07 | 8.51908 |
| EMILIN2 | 0.80795 | 2.162951 | 5.17957 | 6.21E-07 | 4.30E-06 | 5.58961 |
| ANO6 | 0.80829 | 4.067712 | 6.31136 | 2.30E-09 | 3.48E-08 | 10.9802 |
| TUBA1C | 0.80914 | 4.734267 | 4.3395 | 2.44E-05 | 0.000108 | 2.09332 |
| HSPA6 | 0.80973 | 1.661549 | 4.70445 | 5.23E-06 | 2.80E-05 | 3.55418 |
| TAP1 | 0.8104 | 5.050285 | 6.61663 | 4.54E-10 | 8.66E-09 | 12.5493 |
| FBXO5 | 0.81059 | 2.881645 | 7.92573 | 2.80E-13 | 1.58E-11 | 19.7283 |
| SLC37A2 | 0.81063 | 2.134402 | 6.55671 | 6.26E-10 | 1.14E-08 | 12.2378 |
| IGSF6 | 0.81076 | 3.528459 | 5.98504 | 1.24E-08 | 1.48E-07 | 9.35391 |
| PARPBP | 0.81267 | 1.591146 | 9.3914 | 3.73E-17 | 1.24E-14 | 28.4248 |
| KNTC1 | 0.81305 | 3.004474 | 7.79732 | 5.94E-13 | 2.97E-11 | 18.9956 |
| PLCE1 | 0.81334 | 2.687993 | 7.75257 | 7.72E-13 | 3.73E-11 | 18.7417 |
| PLVAP | 0.81343 | 3.05014 | 4.481 | 1.36E-05 | 6.42E-05 | 2.64875 |
| TNFRSF19 | 0.81373 | 3.449208 | 5.18782 | 5.97E-07 | 4.16E-06 | 5.62624 |
| NTN1 | 0.81386 | 4.56119 | 5.97769 | 1.28E-08 | 1.53E-07 | 9.31788 |
| MFNG | 0.81485 | 3.445224 | 6.75635 | 2.13E-10 | 4.57E-09 | 13.2819 |
| TNFSF13B | 0.81515 | 3.044662 | 4.23872 | 3.67E-05 | 0.000155 | 1.70644 |
| RP13-401N8.3 | 0.81518 | 3.356606 | 4.64537 | 6.75E-06 | 3.49E-05 | 3.3115 |
| FYB | 0.81627 | 3.384576 | 5.4893 | 1.44E-07 | 1.22E-06 | 6.99268 |
| PSMB8 | 0.81633 | 5.430706 | 6.9133 | 9.03E-11 | 2.21E-09 | 14.1153 |
| PKIB | 0.81643 | 2.70817 | 4.20311 | 4.24E-05 | 0.000175 | 1.57147 |
| AC008964.1 | 0.81698 | 2.809346 | 5.44262 | 1.80E-07 | 1.49E-06 | 6.77754 |
| SNORA73B | 0.81811 | 3.220727 | 2.42287 | 0.01644 | 0.03249 | -3.9039 |
| MMP7 | 0.81821 | 0.761974 | 4.45336 | 1.52E-05 | 7.12E-05 | 2.53918 |
| OSTC | 0.8183 | 5.183605 | 8.98225 | 4.75E-16 | 9.03E-14 | 25.9428 |
| TRH | 0.81903 | 1.495834 | 3.47149 | 0.00066 | 0.001962 | -0.9902 |
| OAS1 | 0.81905 | 3.864172 | 5.20793 | 5.44E-07 | 3.83E-06 | 5.71564 |
| CDCA2 | 0.81944 | 1.007062 | 8.27825 | 3.45E-14 | 2.73E-12 | 21.7666 |
| GLIPR1 | 0.81995 | 3.668384 | 5.2996 | 3.54E-07 | 2.65E-06 | 6.12644 |
| ZFP36L2 | 0.82027 | 5.763395 | 7.39205 | 6.16E-12 | 2.23E-10 | 16.7212 |
| SNORD3D | 0.8208 | 0.919759 | 5.04411 | 1.16E-06 | 7.41E-06 | 4.99449 |
| BTN3A1 | 0.82096 | 4.339171 | 8.1875 | 5.94E-14 | 4.20E-12 | 21.2382 |
| GINS1 | 0.82124 | 2.157287 | 8.81943 | 1.29E-15 | 1.86E-13 | 24.9656 |
| NCAPD2 | 0.82151 | 4.266964 | 9.08308 | 2.55E-16 | 5.84E-14 | 26.551 |
| CD69 | 0.82153 | 1.475078 | 5.70141 | 5.12E-08 | 4.97E-07 | 7.98612 |
| MCM8 | 0.82198 | 1.883764 | 10.8414 | 3.58E-21 | 2.17E-17 | 37.4542 |
| BTG3 | 0.82223 | 4.700308 | 8.68395 | 2.97E-15 | 3.63E-13 | 24.1575 |
| HIST1H2BJ | 0.82312 | 1.133392 | 7.43258 | 4.89E-12 | 1.82E-10 | 16.946 |
| TLR2 | 0.82441 | 2.522897 | 5.04509 | 1.15E-06 | 7.38E-06 | 4.99879 |
| TAPSAR1 | 0.82459 | 3.655055 | 6.51794 | 7.71E-10 | 1.36E-08 | 12.0371 |
| FAM60A | 0.82469 | 3.707004 | 6.31563 | 2.25E-09 | 3.41E-08 | 11.0018 |
| GNAI3 | 0.82563 | 4.94417 | 9.72072 | 4.69E-18 | 2.66E-15 | 30.4469 |
| SIGLEC1 | 0.82589 | 1.732256 | 5.59186 | 8.75E-08 | 7.92E-07 | 7.46978 |
| EDNRA | 0.82608 | 3.043336 | 5.36581 | 2.59E-07 | 2.03E-06 | 6.42634 |
| TREM2 | 0.82634 | 5.541768 | 4.24782 | 3.54E-05 | 0.00015 | 1.74108 |
| ODF3B | 0.82653 | 3.015488 | 5.23947 | 4.70E-07 | 3.38E-06 | 5.85641 |
| LRRN4CL | 0.82848 | 1.464111 | 4.92236 | 2.00E-06 | 1.20E-05 | 4.46959 |
| HOXD3 | 0.82851 | 1.249643 | 4.84508 | 2.82E-06 | 1.62E-05 | 4.14134 |
| RNASEH2A | 0.82875 | 4.317264 | 9.2384 | 9.70E-17 | 2.81E-14 | 27.4924 |
| PLOD2 | 0.82918 | 4.255785 | 5.57269 | 9.60E-08 | 8.58E-07 | 7.38016 |
| FAM126A | 0.8293 | 3.145834 | 7.52247 | 2.92E-12 | 1.16E-10 | 17.4467 |
| SLC7A7 | 0.83025 | 3.184346 | 6.74235 | 2.30E-10 | 4.88E-09 | 13.2081 |
| CAPG | 0.83032 | 5.474204 | 4.36893 | 2.16E-05 | 9.69E-05 | 2.20771 |
| EXO1 | 0.83051 | 1.388145 | 7.33189 | 8.68E-12 | 3.01E-10 | 16.3887 |
| C1orf162 | 0.83063 | 3.74505 | 5.58768 | 8.93E-08 | 8.04E-07 | 7.45022 |
| FANCC | 0.83071 | 2.037982 | 10.0609 | 5.41E-19 | 6.24E-16 | 32.5557 |
| GBP5 | 0.83213 | 1.252719 | 6.1826 | 4.49E-09 | 6.20E-08 | 10.3319 |
| DTX3L | 0.83226 | 3.238476 | 7.21959 | 1.64E-11 | 5.16E-10 | 15.7719 |
| MCAM | 0.83256 | 4.916521 | 5.31868 | 3.24E-07 | 2.45E-06 | 6.21261 |
| SERPINB8 | 0.83286 | 2.771789 | 7.46522 | 4.06E-12 | 1.55E-10 | 17.1274 |
| S100A6 | 0.83295 | 8.183906 | 4.73021 | 4.68E-06 | 2.53E-05 | 3.66075 |
| OAS2 | 0.83384 | 2.983363 | 5.70209 | 5.10E-08 | 4.96E-07 | 7.98936 |
| CALD1 | 0.8344 | 5.791407 | 6.52611 | 7.38E-10 | 1.31E-08 | 12.0794 |
| DEPDC1 | 0.83442 | 0.771468 | 8.94411 | 6.01E-16 | 1.05E-13 | 25.7134 |
| IGFBP4 | 0.83455 | 4.76742 | 5.20308 | 5.56E-07 | 3.91E-06 | 5.69408 |
| SOD2 | 0.83456 | 7.499022 | 3.71207 | 0.00028 | 0.000926 | -0.1933 |
| C1S | 0.83614 | 5.263999 | 4.40924 | 1.83E-05 | 8.35E-05 | 2.36531 |
| DENND2D | 0.83617 | 2.014562 | 5.90219 | 1.88E-08 | 2.13E-07 | 8.94986 |
| STAT1 | 0.83738 | 5.666209 | 7.37657 | 6.73E-12 | 2.42E-10 | 16.6355 |
| RP11-698N11.2 | 0.83929 | 1.912201 | 5.15904 | 6.82E-07 | 4.67E-06 | 5.49867 |
| YBX1 | 0.83938 | 8.488766 | 8.68151 | 3.01E-15 | 3.66E-13 | 24.143 |
| ADAM9 | 0.84002 | 5.551352 | 7.85107 | 4.34E-13 | 2.26E-11 | 19.3017 |
| MAD2L2 | 0.84023 | 5.6487 | 8.3756 | 1.92E-14 | 1.68E-12 | 22.3361 |
| HAMP | 0.84065 | 2.766314 | 3.43075 | 0.00075 | 0.002219 | -1.1205 |
| METTL1 | 0.8412 | 4.114492 | 5.34375 | 2.88E-07 | 2.22E-06 | 6.32614 |
| LINC00152 | 0.8413 | 3.010157 | 3.94076 | 0.00012 | 0.000433 | 0.60597 |
| APOL1 | 0.84186 | 2.796108 | 5.78608 | 3.36E-08 | 3.49E-07 | 8.38983 |
| MSTN | 0.84241 | 2.849643 | 3.22963 | 0.00149 | 0.004025 | -1.7444 |
| F13A1 | 0.84309 | 2.925741 | 3.59053 | 0.00043 | 0.001364 | -0.6016 |
| MYOF | 0.84383 | 2.908509 | 5.72595 | 4.53E-08 | 4.48E-07 | 8.10271 |
| SLC11A1 | 0.84415 | 3.463424 | 3.88909 | 0.00014 | 0.000516 | 0.42189 |
| FNDC3B | 0.84426 | 3.263736 | 7.65277 | 1.38E-12 | 6.10E-11 | 18.1777 |
| CMTM3 | 0.84431 | 5.3337 | 7.38553 | 6.40E-12 | 2.31E-10 | 16.6851 |
| MAP3K7CL | 0.84587 | 2.219088 | 4.95679 | 1.72E-06 | 1.04E-05 | 4.61706 |
| TMX1 | 0.84621 | 4.831816 | 9.11153 | 2.13E-16 | 5.12E-14 | 26.7231 |
| C3 | 0.84633 | 7.698882 | 3.95811 | 0.00011 | 0.000408 | 0.66823 |
| MGME1 | 0.84658 | 3.535419 | 17.0005 | 1.40E-38 | 3.42E-34 | 76.4541 |
| HOXA4 | 0.84678 | 0.730779 | 4.73583 | 4.56E-06 | 2.48E-05 | 3.68406 |
| HLA-C | 0.84699 | 6.184606 | 3.53879 | 0.00052 | 0.001599 | -0.7719 |
| RAD54L | 0.8473 | 1.652756 | 8.97492 | 4.97E-16 | 9.27E-14 | 25.8987 |
| CLSPN | 0.84737 | 1.333174 | 7.4811 | 3.70E-12 | 1.43E-10 | 17.2159 |
| TLR1 | 0.85053 | 2.727867 | 6.38774 | 1.54E-09 | 2.46E-08 | 11.3686 |
| PDGFD | 0.85314 | 1.458165 | 5.80075 | 3.13E-08 | 3.28E-07 | 8.46016 |
| ANKRD22 | 0.85648 | 1.839972 | 4.88458 | 2.37E-06 | 1.39E-05 | 4.30863 |
| SLAMF8 | 0.85685 | 1.719794 | 6.04111 | 9.30E-09 | 1.16E-07 | 9.62941 |
| MATN2 | 0.85718 | 5.502933 | 5.14197 | 7.38E-07 | 5.00E-06 | 5.42329 |
| PSORS1C1 | 0.85845 | 1.659565 | 4.83152 | 3.00E-06 | 1.71E-05 | 4.08415 |
| CCR1 | 0.85937 | 2.936433 | 5.76807 | 3.68E-08 | 3.75E-07 | 8.3036 |
| PLK4 | 0.85954 | 2.167221 | 8.08906 | 1.07E-13 | 6.88E-12 | 20.6679 |
| KIAA0040 | 0.86038 | 2.432694 | 5.69372 | 5.31E-08 | 5.14E-07 | 7.94966 |
| TGFB2 | 0.8611 | 3.529529 | 3.81083 | 0.00019 | 0.00067 | 0.14693 |
| PLP2 | 0.86146 | 3.841222 | 4.56267 | 9.61E-06 | 4.74E-05 | 2.97573 |
| CDKN2A | 0.86169 | 3.866151 | 3.64055 | 0.00036 | 0.001164 | -0.435 |
| CALU | 0.86516 | 5.676656 | 6.52085 | 7.59E-10 | 1.34E-08 | 12.0522 |
| ITGB3BP | 0.86539 | 3.48047 | 9.1616 | 1.56E-16 | 4.01E-14 | 27.0263 |
| UBE2T | 0.86578 | 3.341559 | 6.99111 | 5.87E-11 | 1.55E-09 | 14.5325 |
| RP11-834C11.4 | 0.8661 | 2.869874 | 5.59704 | 8.53E-08 | 7.76E-07 | 7.49405 |
| GAS1 | 0.86632 | 3.343724 | 5.33596 | 2.99E-07 | 2.29E-06 | 6.2908 |
| PLAC8 | 0.86648 | 1.340893 | 5.87647 | 2.14E-08 | 2.37E-07 | 8.82514 |
| CTHRC1 | 0.8667 | 2.048322 | 4.67215 | 6.01E-06 | 3.15E-05 | 3.42123 |
| RPE65 | 0.86828 | 2.447763 | 4.10542 | 6.24E-05 | 0.000247 | 1.20602 |
| PLAUR | 0.86871 | 2.897381 | 4.70311 | 5.26E-06 | 2.81E-05 | 3.54867 |
| VEGFA | 0.8688 | 5.546828 | 3.61369 | 0.0004 | 0.001267 | -0.5247 |
| AC099522.1 | 0.86986 | 1.944706 | 5.62349 | 7.50E-08 | 6.93E-07 | 7.6182 |
| ACTG2 | 0.87072 | 1.855334 | 3.95311 | 0.00011 | 0.000415 | 0.65025 |
| FOXJ1 | 0.87093 | 2.39823 | 4.05179 | 7.70E-05 | 0.000297 | 1.00836 |
| POSTN | 0.87108 | 1.225387 | 3.06897 | 0.0025 | 0.006352 | -2.2188 |
| C1orf226 | 0.87148 | 3.278978 | 6.41292 | 1.35E-09 | 2.20E-08 | 11.4972 |
| HLA-DQB1 | 0.87205 | 2.863231 | 2.50554 | 0.01316 | 0.026856 | -3.7087 |
| IGHG3 | 0.87311 | 1.188358 | 3.34217 | 0.00102 | 0.002894 | -1.3994 |
| PTPRC | 0.87316 | 3.353006 | 6.081 | 7.59E-09 | 9.73E-08 | 9.82643 |
| IL18 | 0.87341 | 3.513526 | 5.61374 | 7.86E-08 | 7.21E-07 | 7.57238 |
| FAM129A | 0.87449 | 2.285228 | 5.74104 | 4.21E-08 | 4.21E-07 | 8.17456 |
| TGFBR1 | 0.87487 | 4.162221 | 8.30258 | 2.98E-14 | 2.43E-12 | 21.9087 |
| PLEK | 0.87506 | 3.174755 | 5.89211 | 1.98E-08 | 2.22E-07 | 8.90094 |
| CD300A | 0.8754 | 3.207822 | 7.0764 | 3.65E-11 | 1.03E-09 | 14.9927 |
| ATF3 | 0.87602 | 4.007022 | 4.49431 | 1.28E-05 | 6.11E-05 | 2.70172 |
| TP53I3 | 0.87653 | 4.082098 | 7.63912 | 1.49E-12 | 6.51E-11 | 18.1008 |
| RAB32 | 0.8786 | 3.034162 | 6.24334 | 3.28E-09 | 4.75E-08 | 10.6367 |
| IL13RA2 | 0.87861 | 1.796205 | 3.63196 | 0.00037 | 0.001196 | -0.4637 |
| PCOLCE | 0.87871 | 3.813269 | 5.40244 | 2.18E-07 | 1.75E-06 | 6.59336 |
| SLC40A1 | 0.87921 | 4.486727 | 7.23661 | 1.49E-11 | 4.78E-10 | 15.8651 |
| HMGB2 | 0.8797 | 6.0968 | 8.88607 | 8.60E-16 | 1.38E-13 | 25.3648 |
| RUNX1 | 0.8804 | 2.288644 | 5.49265 | 1.41E-07 | 1.20E-06 | 7.00816 |
| TYROBP | 0.88081 | 6.774016 | 5.69565 | 5.26E-08 | 5.10E-07 | 7.95883 |
| GJC1 | 0.88296 | 2.315505 | 6.35345 | 1.84E-09 | 2.85E-08 | 11.1938 |
| DBF4 | 0.88357 | 2.955623 | 8.7777 | 1.67E-15 | 2.30E-13 | 24.7162 |
| OIP5 | 0.88446 | 1.483068 | 9.10217 | 2.26E-16 | 5.29E-14 | 26.6665 |
| PARVG | 0.88635 | 3.641365 | 6.12306 | 6.11E-09 | 8.06E-08 | 10.0351 |
| ORC6 | 0.88644 | 2.564585 | 8.59202 | 5.19E-15 | 5.85E-13 | 23.6118 |
| LOX | 0.88664 | 1.434911 | 4.33891 | 2.44E-05 | 0.000108 | 2.09106 |
| E2F2 | 0.88701 | 0.962346 | 8.20003 | 5.51E-14 | 3.94E-12 | 21.311 |
| RAD51 | 0.8876 | 1.691091 | 7.93492 | 2.65E-13 | 1.51E-11 | 19.7809 |
| SFRP4 | 0.88761 | 3.033 | 3.97766 | 0.0001 | 0.000382 | 0.73868 |
| EGR2 | 0.88773 | 3.378253 | 3.86287 | 0.00016 | 0.000562 | 0.32923 |
| CD276 | 0.88834 | 5.291652 | 6.70043 | 2.89E-10 | 5.89E-09 | 12.9876 |
| NAMPT | 0.88867 | 5.616427 | 4.46676 | 1.44E-05 | 6.78E-05 | 2.59223 |
| GINS2 | 0.88948 | 2.09265 | 8.93254 | 6.46E-16 | 1.12E-13 | 25.6438 |
| COL8A1 | 0.88965 | 1.267577 | 4.59814 | 8.26E-06 | 4.17E-05 | 3.11917 |
| CCL2 | 0.89106 | 5.219644 | 3.01734 | 0.00294 | 0.007325 | -2.3667 |
| CYBB | 0.89115 | 4.283603 | 5.11398 | 8.40E-07 | 5.59E-06 | 5.30003 |
| OSMR | 0.89137 | 2.762114 | 5.51277 | 1.28E-07 | 1.10E-06 | 7.10132 |
| EPSTI1 | 0.89188 | 2.483793 | 6.41312 | 1.35E-09 | 2.19E-08 | 11.4982 |
| IGFBP7 | 0.89201 | 8.263386 | 6.31983 | 2.20E-09 | 3.35E-08 | 11.0231 |
| RBMS1 | 0.89252 | 3.455785 | 7.64195 | 1.47E-12 | 6.44E-11 | 18.1167 |
| RDH10 | 0.89321 | 3.577179 | 4.6223 | 7.45E-06 | 3.80E-05 | 3.21733 |
| ICAM1 | 0.89387 | 2.996396 | 4.54696 | 1.03E-05 | 5.02E-05 | 2.91246 |
| RCC2 | 0.89399 | 4.989423 | 9.84088 | 2.19E-18 | 1.40E-15 | 31.1896 |
| PLEKHG2 | 0.89471 | 3.856474 | 7.00849 | 5.33E-11 | 1.43E-09 | 14.626 |
| SLN | 0.89493 | 2.301326 | 2.79514 | 0.00578 | 0.013197 | -2.9772 |
| HGF | 0.89549 | 1.97216 | 5.945 | 1.52E-08 | 1.77E-07 | 9.15815 |
| TREM1 | 0.89558 | 1.633343 | 3.99042 | 9.77E-05 | 0.000365 | 0.78479 |
| EMILIN1 | 0.89572 | 3.456164 | 5.68806 | 5.46E-08 | 5.28E-07 | 7.92282 |
| FGL2 | 0.89724 | 3.066789 | 6.55674 | 6.26E-10 | 1.14E-08 | 12.2379 |
| IFI6 | 0.89771 | 7.177602 | 5.05265 | 1.11E-06 | 7.15E-06 | 5.0317 |
| MRC2 | 0.89812 | 4.399468 | 4.74818 | 4.32E-06 | 2.37E-05 | 3.73536 |
| HCP5 | 0.89827 | 2.555082 | 6.61464 | 4.59E-10 | 8.74E-09 | 12.5389 |
| CENPW | 0.89844 | 2.016842 | 7.89528 | 3.35E-13 | 1.82E-11 | 19.5541 |
| CAPN5 | 0.89892 | 4.430068 | 6.42224 | 1.28E-09 | 2.10E-08 | 11.5449 |
| SNHG12 | 0.89941 | 4.766165 | 6.44564 | 1.13E-09 | 1.90E-08 | 11.6649 |
| TRIP13 | 0.89946 | 1.909919 | 9.03521 | 3.43E-16 | 6.94E-14 | 26.262 |
| ESPL1 | 0.89994 | 1.603656 | 5.85324 | 2.41E-08 | 2.62E-07 | 8.71283 |
| ANTXR2 | 0.90117 | 3.373891 | 7.51284 | 3.09E-12 | 1.22E-10 | 17.3929 |
| CHIC2 | 0.90243 | 3.753711 | 7.76235 | 7.29E-13 | 3.54E-11 | 18.7971 |
| SP100 | 0.90314 | 3.93773 | 6.79991 | 1.68E-10 | 3.73E-09 | 13.5122 |
| MCM10 | 0.90378 | 1.364927 | 7.43439 | 4.84E-12 | 1.81E-10 | 16.956 |
| DEPDC1B | 0.90419 | 1.338878 | 8.38672 | 1.80E-14 | 1.60E-12 | 22.4014 |
| LPAR6 | 0.90435 | 4.046207 | 6.48596 | 9.14E-10 | 1.58E-08 | 11.8722 |
| MND1 | 0.90557 | 1.639887 | 7.90632 | 3.14E-13 | 1.74E-11 | 19.6172 |
| IKBIP | 0.90589 | 2.883769 | 8.73363 | 2.19E-15 | 2.85E-13 | 24.4533 |
| DNALI1 | 0.90597 | 4.62946 | 6.805 | 1.64E-10 | 3.65E-09 | 13.5391 |
| SKA3 | 0.90689 | 1.590461 | 7.76559 | 7.15E-13 | 3.49E-11 | 18.8155 |
| PDIA4 | 0.90818 | 5.490358 | 8.07815 | 1.14E-13 | 7.24E-12 | 20.6049 |
| KLHDC8A | 0.90849 | 4.301458 | 4.55123 | 1.01E-05 | 4.95E-05 | 2.92966 |
| FCER1G | 0.90932 | 6.120331 | 5.4108 | 2.09E-07 | 1.69E-06 | 6.63162 |
| FBLIM1 | 0.91019 | 2.374947 | 5.48826 | 1.44E-07 | 1.22E-06 | 6.98788 |
| ST14 | 0.91097 | 1.784464 | 6.27735 | 2.75E-09 | 4.07E-08 | 10.8081 |
| IFI44L | 0.91153 | 4.157584 | 5.18047 | 6.18E-07 | 4.29E-06 | 5.59362 |
| APOBEC3C | 0.91254 | 2.807077 | 6.65838 | 3.63E-10 | 7.18E-09 | 12.7673 |
| ITGB1 | 0.91299 | 6.252144 | 7.54063 | 2.63E-12 | 1.06E-10 | 17.5482 |
| HIST2H2AA4 | 0.9134 | 4.322575 | 5.99107 | 1.20E-08 | 1.44E-07 | 9.38345 |
| HLA-F | 0.91362 | 5.098506 | 6.65111 | 3.77E-10 | 7.43E-09 | 12.7292 |
| CD58 | 0.91589 | 2.955325 | 6.81924 | 1.51E-10 | 3.43E-09 | 13.6146 |
| CDC6 | 0.91619 | 1.747452 | 8.54763 | 6.80E-15 | 7.32E-13 | 23.3491 |
| TNFAIP8 | 0.91631 | 1.822133 | 7.65695 | 1.34E-12 | 5.98E-11 | 18.2012 |
| LAMB1 | 0.91661 | 3.960049 | 4.97319 | 1.59E-06 | 9.80E-06 | 4.68759 |
| OLFML3 | 0.91831 | 4.758784 | 6.91448 | 8.97E-11 | 2.19E-09 | 14.1217 |
| SIX1 | 0.91922 | 1.852524 | 5.53618 | 1.15E-07 | 1.00E-06 | 7.21 |
| HOXD8 | 0.91959 | 1.565447 | 4.01497 | 8.89E-05 | 0.000336 | 0.87388 |
| PYGL | 0.92169 | 3.889086 | 5.72079 | 4.65E-08 | 4.58E-07 | 8.07818 |
| CYBA | 0.92183 | 5.863253 | 5.82402 | 2.78E-08 | 2.96E-07 | 8.57201 |
| NABP1 | 0.92284 | 2.80123 | 7.76621 | 7.13E-13 | 3.48E-11 | 18.819 |
| BARD1 | 0.92422 | 2.401109 | 8.40885 | 1.57E-14 | 1.43E-12 | 22.5313 |
| SAMD9 | 0.92599 | 2.323477 | 8.45778 | 1.17E-14 | 1.13E-12 | 22.819 |
| NMI | 0.92669 | 2.926395 | 7.98111 | 2.02E-13 | 1.20E-11 | 20.0459 |
| HOXA5 | 0.92677 | 0.878511 | 5.10553 | 8.73E-07 | 5.78E-06 | 5.2629 |
| GADD45A | 0.92713 | 5.163129 | 5.24995 | 4.47E-07 | 3.24E-06 | 5.90332 |
| FANCD2 | 0.9291 | 2.569767 | 8.2299 | 4.61E-14 | 3.42E-12 | 21.4848 |
| PCNA | 0.93039 | 5.895946 | 12.3588 | 1.77E-25 | 2.16E-21 | 47.1251 |
| PTX3 | 0.93167 | 1.8316 | 4.15172 | 5.20E-05 | 0.00021 | 1.37835 |
| TMEM255A | 0.93194 | 4.313023 | 6.02442 | 1.01E-08 | 1.24E-07 | 9.54722 |
| ITGA4 | 0.93275 | 1.75956 | 6.9864 | 6.03E-11 | 1.58E-09 | 14.5071 |
| IGHA1 | 0.93402 | 1.712934 | 3.24742 | 0.0014 | 0.003822 | -1.6906 |
| FAM187A | 0.93774 | 2.800594 | 5.40575 | 2.14E-07 | 1.73E-06 | 6.60853 |
| PSMB9 | 0.94023 | 5.125261 | 7.10253 | 3.16E-11 | 9.11E-10 | 15.1342 |
| ECT2 | 0.94135 | 2.797805 | 8.67595 | 3.11E-15 | 3.77E-13 | 24.1099 |
| CD248 | 0.94214 | 2.206192 | 4.69373 | 5.48E-06 | 2.90E-05 | 3.50999 |
| PTGES3P1 | 0.94225 | 2.353829 | 4.75737 | 4.15E-06 | 2.28E-05 | 3.7736 |
| KIF11 | 0.94242 | 2.045272 | 8.00738 | 1.73E-13 | 1.05E-11 | 20.1969 |
| LAMA4 | 0.94459 | 4.017175 | 6.90683 | 9.35E-11 | 2.27E-09 | 14.0808 |
| CPVL | 0.94514 | 4.304619 | 6.68872 | 3.08E-10 | 6.20E-09 | 12.9262 |
| HIST1H2BK | 0.94553 | 4.172816 | 6.85779 | 1.22E-10 | 2.87E-09 | 13.8194 |
| PARP9 | 0.94574 | 4.263996 | 7.90772 | 3.11E-13 | 1.73E-11 | 19.6252 |
| CHEK2 | 0.94633 | 2.080122 | 10.0977 | 4.28E-19 | 6.24E-16 | 32.7846 |
| C1QC | 0.94659 | 7.347106 | 5.77946 | 3.48E-08 | 3.58E-07 | 8.3581 |
| GDF15 | 0.94763 | 1.648848 | 4.8737 | 2.49E-06 | 1.45E-05 | 4.26247 |
| EME1 | 0.94792 | 2.194161 | 9.18173 | 1.38E-16 | 3.69E-14 | 27.1484 |
| DRAXIN | 0.94793 | 1.551566 | 6.37065 | 1.68E-09 | 2.65E-08 | 11.2814 |
| SEC61G | 0.94826 | 7.028991 | 5.56 | 1.02E-07 | 9.05E-07 | 7.32093 |
| CLEC18B | 0.94849 | 2.155138 | 5.29046 | 3.70E-07 | 2.75E-06 | 6.08526 |
| LY96 | 0.94941 | 3.564362 | 5.84951 | 2.45E-08 | 2.66E-07 | 8.69481 |
| CENPH | 0.94972 | 2.969659 | 8.87953 | 8.95E-16 | 1.41E-13 | 25.3256 |
| RP11-124N14.3 | 0.95089 | 1.246036 | 6.88615 | 1.05E-10 | 2.51E-09 | 13.9704 |
| IRF1 | 0.95093 | 3.805533 | 6.39158 | 1.51E-09 | 2.42E-08 | 11.3882 |
| GNG5 | 0.95158 | 6.15655 | 7.68069 | 1.17E-12 | 5.29E-11 | 18.3351 |
| CCND1 | 0.95159 | 5.120446 | 5.94128 | 1.54E-08 | 1.80E-07 | 9.14001 |
| B2M | 0.95346 | 11.70391 | 7.61742 | 1.69E-12 | 7.23E-11 | 17.9788 |
| HAS2 | 0.95501 | 1.913122 | 5.87582 | 2.15E-08 | 2.38E-07 | 8.82201 |
| TUBB6 | 0.95536 | 4.326747 | 5.99666 | 1.17E-08 | 1.41E-07 | 9.41086 |
| ITGA5 | 0.95561 | 3.388858 | 5.42163 | 1.99E-07 | 1.62E-06 | 6.68121 |
| LUM | 0.95583 | 2.692765 | 4.34933 | 2.34E-05 | 0.000104 | 2.13145 |
| BST2 | 0.95761 | 5.559471 | 6.27664 | 2.76E-09 | 4.08E-08 | 10.8045 |
| OLFML2B | 0.95847 | 3.099197 | 5.83568 | 2.63E-08 | 2.81E-07 | 8.62815 |
| CNN3 | 0.95933 | 8.161936 | 7.33169 | 8.69E-12 | 3.01E-10 | 16.3876 |
| CDCA5 | 0.95938 | 2.800487 | 7.49737 | 3.37E-12 | 1.32E-10 | 17.3066 |
| ARHGAP11A | 0.96016 | 2.354968 | 7.90616 | 3.14E-13 | 1.74E-11 | 19.6163 |
| C4A | 0.96068 | 6.879988 | 4.20085 | 4.27E-05 | 0.000176 | 1.56294 |
| BTN3A2 | 0.96069 | 4.682553 | 7.2831 | 1.14E-11 | 3.82E-10 | 16.1201 |
| NLRC5 | 0.96164 | 3.443355 | 7.63543 | 1.52E-12 | 6.62E-11 | 18.0801 |
| ADAMTS15 | 0.96198 | 1.776727 | 5.32981 | 3.07E-07 | 2.35E-06 | 6.26296 |
| ESM1 | 0.96413 | 1.402349 | 4.04139 | 8.02E-05 | 0.000308 | 0.97027 |
| SPON2 | 0.96421 | 2.664025 | 4.56078 | 9.69E-06 | 4.77E-05 | 2.96808 |
| SGOL1 | 0.96709 | 1.287471 | 8.57574 | 5.73E-15 | 6.36E-13 | 23.5154 |
| GPX7 | 0.96725 | 3.378019 | 9.33097 | 5.44E-17 | 1.68E-14 | 28.056 |
| SAMD9L | 0.96808 | 2.868556 | 7.0345 | 4.61E-11 | 1.26E-09 | 14.7662 |
| HMMR | 0.96932 | 1.530698 | 9.20926 | 1.16E-16 | 3.21E-14 | 27.3155 |
| CRISPLD1 | 0.96992 | 4.755015 | 5.59271 | 8.71E-08 | 7.89E-07 | 7.47376 |
| LEPREL1 | 0.97171 | 2.431196 | 6.73751 | 2.36E-10 | 4.97E-09 | 13.1826 |
| DPEP1 | 0.97214 | 1.355057 | 5.56021 | 1.02E-07 | 9.04E-07 | 7.32192 |
| CLIC4 | 0.97252 | 6.753965 | 7.74748 | 7.95E-13 | 3.83E-11 | 18.7128 |
| RP11-565P22.6 | 0.97262 | 2.011027 | 6.81558 | 1.54E-10 | 3.49E-09 | 13.5952 |
| RPS17L | 0.97392 | 9.065956 | 3.1629 | 0.00185 | 0.004864 | -1.9441 |
| STAB1 | 0.97835 | 5.002352 | 5.7985 | 3.16E-08 | 3.31E-07 | 8.44935 |
| SHCBP1 | 0.97997 | 1.845382 | 9.10641 | 2.20E-16 | 5.20E-14 | 26.6921 |
| PROS1 | 0.98048 | 3.936527 | 7.42252 | 5.18E-12 | 1.92E-10 | 16.8902 |
| ZNF367 | 0.98052 | 2.005825 | 9.99844 | 8.06E-19 | 7.84E-16 | 32.167 |
| MCM2 | 0.98071 | 3.41881 | 7.74493 | 8.07E-13 | 3.88E-11 | 18.6984 |
| FAM83D | 0.98087 | 1.468539 | 7.51987 | 2.97E-12 | 1.17E-10 | 17.4322 |
| CCL5 | 0.98099 | 2.146528 | 5.62527 | 7.43E-08 | 6.89E-07 | 7.62652 |
| GTSE1 | 0.98268 | 1.869195 | 6.95879 | 7.02E-11 | 1.79E-09 | 14.3589 |
| PRR11 | 0.98404 | 2.689446 | 8.49222 | 9.51E-15 | 9.56E-13 | 23.0219 |
| RGS16 | 0.98476 | 2.689216 | 5.11348 | 8.42E-07 | 5.60E-06 | 5.29781 |
| ANGPT2 | 0.98595 | 2.369444 | 5.15533 | 6.94E-07 | 4.74E-06 | 5.48229 |
| ESCO2 | 0.98598 | 1.444097 | 8.17502 | 6.39E-14 | 4.41E-12 | 21.1658 |
| CD68 | 0.98605 | 6.266004 | 5.67762 | 5.75E-08 | 5.51E-07 | 7.8734 |
| IQGAP3 | 0.98606 | 1.479577 | 7.72705 | 8.95E-13 | 4.22E-11 | 18.5971 |
| HOXA7 | 0.98715 | 1.068412 | 4.86094 | 2.63E-06 | 1.53E-05 | 4.20838 |
| C4B | 0.98804 | 6.526798 | 4.27269 | 3.20E-05 | 0.000137 | 1.83602 |
| STEAP3 | 0.98887 | 2.552304 | 5.08544 | 9.57E-07 | 6.28E-06 | 5.17487 |
| FAM111A | 0.98906 | 3.602719 | 9.38998 | 3.76E-17 | 1.24E-14 | 28.4161 |
| S100A10 | 0.98936 | 6.773311 | 4.28278 | 3.07E-05 | 0.000132 | 1.87468 |
| MMP2 | 0.98946 | 4.625278 | 6.67508 | 3.31E-10 | 6.63E-09 | 12.8547 |
| MSN | 0.9903 | 5.797573 | 5.91524 | 1.76E-08 | 2.01E-07 | 9.01325 |
| TTK | 0.99206 | 1.380913 | 8.56235 | 6.22E-15 | 6.75E-13 | 23.4361 |
| SPP1 | 0.99315 | 9.104359 | 3.67453 | 0.00032 | 0.001045 | -0.3207 |
| LAMC1 | 0.99373 | 3.873673 | 6.37017 | 1.69E-09 | 2.66E-08 | 11.2789 |
| OR4N2 | 0.99534 | 1.335657 | 4.83383 | 2.97E-06 | 1.70E-05 | 4.0939 |
| COL5A1 | 0.99537 | 1.93404 | 4.90853 | 2.13E-06 | 1.26E-05 | 4.41055 |
| PRC1 | 0.99654 | 3.826196 | 7.32544 | 9.00E-12 | 3.11E-10 | 16.3532 |
| DIRAS3 | 0.99961 | 2.36391 | 5.60326 | 8.28E-08 | 7.53E-07 | 7.52323 |
| RNF122 | 1.00035 | 2.946433 | 8.2033 | 5.40E-14 | 3.91E-12 | 21.33 |
| KIF18B | 1.00176 | 2.03446 | 6.9251 | 8.46E-11 | 2.09E-09 | 14.1784 |
| MMP9 | 1.0019 | 1.330538 | 3.80049 | 0.0002 | 0.000692 | 0.11096 |
| BCL2A1 | 1.00325 | 1.967359 | 5.49743 | 1.38E-07 | 1.18E-06 | 7.03026 |
| HLA-A | 1.00355 | 8.010437 | 5.846 | 2.49E-08 | 2.69E-07 | 8.67791 |
| EN1 | 1.00468 | 0.821862 | 6.16185 | 5.00E-09 | 6.80E-08 | 10.2283 |
| BTN2A2 | 1.00549 | 3.196916 | 10.0636 | 5.32E-19 | 6.24E-16 | 32.5724 |
| KIF15 | 1.00703 | 2.091962 | 8.86458 | 9.81E-16 | 1.50E-13 | 25.236 |
| TIMELESS | 1.01213 | 3.212754 | 9.60151 | 9.96E-18 | 4.72E-15 | 29.7126 |
| NEK2 | 1.01234 | 1.545277 | 7.70613 | 1.01E-12 | 4.70E-11 | 18.4788 |
| ECSCR | 1.01309 | 3.942808 | 5.52708 | 1.20E-07 | 1.04E-06 | 7.16771 |
| MXD3 | 1.01335 | 3.21413 | 8.31704 | 2.73E-14 | 2.25E-12 | 21.9932 |
| IGLC3 | 1.01337 | 1.735662 | 3.11177 | 0.00218 | 0.005634 | -2.0945 |
| LEFTY2 | 1.01482 | 1.270486 | 5.10188 | 8.88E-07 | 5.87E-06 | 5.24687 |
| IGLC2 | 1.01602 | 1.580486 | 3.32347 | 0.00109 | 0.003055 | -1.4575 |
| GPX8 | 1.01628 | 1.429142 | 6.08325 | 7.50E-09 | 9.63E-08 | 9.83757 |
| NPNT | 1.01656 | 2.85467 | 4.90243 | 2.19E-06 | 1.30E-05 | 4.38455 |
| CASP1 | 1.02 | 3.748814 | 7.57134 | 2.21E-12 | 9.09E-11 | 17.7201 |
| HSPA7 | 1.02138 | 1.856909 | 4.96333 | 1.67E-06 | 1.02E-05 | 4.64517 |
| HSPG2 | 1.022 | 3.10715 | 5.54173 | 1.12E-07 | 9.78E-07 | 7.23581 |
| CCDC109B | 1.02322 | 2.652807 | 6.21243 | 3.85E-09 | 5.44E-08 | 10.4814 |
| C1QB | 1.02454 | 7.61579 | 6.10304 | 6.77E-09 | 8.81E-08 | 9.93564 |
| TK1 | 1.025 | 2.303619 | 7.63938 | 1.49E-12 | 6.51E-11 | 18.1023 |
| RGS1 | 1.02816 | 5.046292 | 3.73341 | 0.00026 | 0.000863 | -0.1204 |
| IGLC1 | 1.0283 | 1.410801 | 3.4044 | 0.00083 | 0.002404 | -1.2041 |
| CYR61 | 1.03125 | 4.715581 | 4.32637 | 2.57E-05 | 0.000113 | 2.04253 |
| FAM26F | 1.03213 | 2.499095 | 6.40763 | 1.38E-09 | 2.25E-08 | 11.4702 |
| FLNC | 1.03347 | 2.907285 | 4.56357 | 9.57E-06 | 4.73E-05 | 2.97936 |
| S100A3 | 1.03652 | 1.550208 | 5.54411 | 1.10E-07 | 9.69E-07 | 7.24691 |
| AURKA | 1.0369 | 2.015732 | 9.86464 | 1.89E-18 | 1.31E-15 | 31.3368 |
| CA3 | 1.03747 | 1.47207 | 4.69194 | 5.52E-06 | 2.93E-05 | 3.5026 |
| GAS2L3 | 1.03873 | 1.61266 | 8.48715 | 9.81E-15 | 9.78E-13 | 22.992 |
| HOXA10 | 1.03919 | 0.964724 | 5.35766 | 2.69E-07 | 2.09E-06 | 6.38926 |
| SRPX2 | 1.04015 | 1.668883 | 5.37792 | 2.45E-07 | 1.93E-06 | 6.48147 |
| PLK1 | 1.04061 | 2.689978 | 8.23178 | 4.56E-14 | 3.40E-12 | 21.4957 |
| CDCA7L | 1.04066 | 3.775344 | 7.68443 | 1.15E-12 | 5.18E-11 | 18.3562 |
| IFI44 | 1.04217 | 4.698855 | 7.49229 | 3.47E-12 | 1.35E-10 | 17.2783 |
| CEP55 | 1.04267 | 1.283838 | 8.77838 | 1.67E-15 | 2.30E-13 | 24.7203 |
| HOTAIRM1 | 1.04297 | 1.669742 | 4.83277 | 2.98E-06 | 1.71E-05 | 4.08943 |
| ISG15 | 1.04334 | 5.670755 | 5.68675 | 5.50E-08 | 5.30E-07 | 7.91661 |
| FAM111B | 1.04394 | 1.391274 | 9.34487 | 4.99E-17 | 1.60E-14 | 28.1407 |
| TAGLN2 | 1.044 | 6.120961 | 5.5864 | 8.99E-08 | 8.08E-07 | 7.44422 |
| CHEK1 | 1.04451 | 2.66505 | 10.0032 | 7.81E-19 | 7.84E-16 | 32.1967 |
| TMEM45A | 1.04557 | 3.37967 | 7.62743 | 1.60E-12 | 6.88E-11 | 18.035 |
| IFI16 | 1.04653 | 5.717244 | 8.4382 | 1.32E-14 | 1.22E-12 | 22.7038 |
| AC015936.3 | 1.04691 | 2.907693 | 4.1407 | 5.43E-05 | 0.000218 | 1.33717 |
| MS4A7 | 1.04729 | 4.42522 | 6.23837 | 3.36E-09 | 4.85E-08 | 10.6117 |
| CASP4 | 1.0476 | 3.35074 | 6.94274 | 7.67E-11 | 1.93E-09 | 14.2729 |
| CDKN3 | 1.04812 | 2.673778 | 8.61382 | 4.55E-15 | 5.27E-13 | 23.741 |
| PRSS23 | 1.05038 | 4.372343 | 5.8337 | 2.65E-08 | 2.83E-07 | 8.61862 |
| CSRP2 | 1.05122 | 6.285965 | 5.78542 | 3.38E-08 | 3.50E-07 | 8.38665 |
| EMP3 | 1.05448 | 3.952755 | 3.95001 | 0.00011 | 0.000419 | 0.63914 |
| RCC1 | 1.0548 | 3.547855 | 9.57682 | 1.16E-17 | 4.88E-15 | 29.5608 |
| MIR4435-1HG | 1.05739 | 3.174591 | 4.77164 | 3.90E-06 | 2.16E-05 | 3.83308 |
| FCGR2A | 1.05817 | 4.384453 | 6.04333 | 9.20E-09 | 1.15E-07 | 9.64035 |
| TM4SF1 | 1.05859 | 3.840698 | 6.0247 | 1.01E-08 | 1.24E-07 | 9.5486 |
| CKAP2L | 1.06078 | 1.465128 | 8.9766 | 4.92E-16 | 9.27E-14 | 25.9088 |
| PDIA5 | 1.06094 | 2.448531 | 8.18411 | 6.06E-14 | 4.26E-12 | 21.2185 |
| SERPINA1 | 1.06295 | 3.87548 | 5.2781 | 3.92E-07 | 2.89E-06 | 6.02967 |
| MS4A4A | 1.06348 | 3.571406 | 5.7409 | 4.21E-08 | 4.21E-07 | 8.17389 |
| CDCA3 | 1.06362 | 2.80464 | 9.19976 | 1.23E-16 | 3.33E-14 | 27.2578 |
| H19 | 1.06428 | 1.970663 | 3.66183 | 0.00033 | 0.001088 | -0.3635 |
| GPNMB | 1.06547 | 4.42098 | 4.05537 | 7.60E-05 | 0.000293 | 1.02147 |
| RP1-261G23.7 | 1.06564 | 4.074161 | 2.92618 | 0.0039 | 0.009361 | -2.6223 |
| CCNB1 | 1.06805 | 3.403788 | 8.11689 | 9.04E-14 | 5.94E-12 | 20.8288 |
| MAD2L1 | 1.06833 | 3.240887 | 9.04195 | 3.29E-16 | 6.83E-14 | 26.3027 |
| C1QA | 1.07032 | 6.954405 | 6.47428 | 9.73E-10 | 1.67E-08 | 11.8121 |
| MKI67 | 1.07341 | 2.14158 | 7.28994 | 1.10E-11 | 3.69E-10 | 16.1577 |
| SULF1 | 1.07534 | 3.810962 | 4.7636 | 4.04E-06 | 2.23E-05 | 3.79958 |
| COL11A1 | 1.07536 | 2.679649 | 5.41621 | 2.04E-07 | 1.66E-06 | 6.65637 |
| CDK6 | 1.07644 | 2.950574 | 7.59069 | 1.97E-12 | 8.26E-11 | 17.8287 |
| RP4-792G4.2 | 1.07669 | 1.453586 | 5.92473 | 1.68E-08 | 1.93E-07 | 9.05938 |
| ASPM | 1.0769 | 1.088986 | 9.68144 | 6.02E-18 | 3.21E-15 | 30.2047 |
| CD48 | 1.07846 | 1.899504 | 6.52959 | 7.24E-10 | 1.29E-08 | 12.0973 |
| HMOX1 | 1.07933 | 4.836268 | 5.1711 | 6.45E-07 | 4.45E-06 | 5.55206 |
| S1PR3 | 1.07967 | 3.658468 | 5.77025 | 3.64E-08 | 3.72E-07 | 8.31402 |
| FN1 | 1.08264 | 6.861045 | 5.46336 | 1.63E-07 | 1.36E-06 | 6.87293 |
| CTSS | 1.0835 | 5.11435 | 6.81251 | 1.57E-10 | 3.53E-09 | 13.5789 |
| HLA-B | 1.08555 | 7.81547 | 6.17588 | 4.65E-09 | 6.39E-08 | 10.2984 |
| ABCC3 | 1.08909 | 1.927852 | 4.84777 | 2.79E-06 | 1.61E-05 | 4.15272 |
| UCP2 | 1.09013 | 5.021708 | 6.44608 | 1.13E-09 | 1.89E-08 | 11.6671 |
| DTL | 1.09068 | 2.161639 | 7.17664 | 2.09E-11 | 6.41E-10 | 15.5373 |
| BUB1B | 1.09149 | 1.980067 | 7.73504 | 8.54E-13 | 4.08E-11 | 18.6423 |
| S100A11 | 1.09319 | 6.494501 | 5.8511 | 2.43E-08 | 2.64E-07 | 8.70251 |
| CKS2 | 1.09427 | 4.510552 | 8.3509 | 2.23E-14 | 1.88E-12 | 22.1914 |
| GPR65 | 1.0949 | 1.694969 | 7.06376 | 3.92E-11 | 1.10E-09 | 14.9243 |
| PLAT | 1.09531 | 3.39052 | 5.54233 | 1.11E-07 | 9.75E-07 | 7.23861 |
| PLSCR1 | 1.09602 | 4.693449 | 7.64928 | 1.41E-12 | 6.20E-11 | 18.158 |
| ID3 | 1.09757 | 7.347878 | 5.65888 | 6.31E-08 | 5.97E-07 | 7.78488 |
| CARD16 | 1.10053 | 3.098061 | 6.83723 | 1.37E-10 | 3.15E-09 | 13.7101 |
| TGIF1 | 1.10165 | 4.108725 | 8.08685 | 1.08E-13 | 6.94E-12 | 20.6551 |
| CAV1 | 1.10322 | 4.036287 | 5.63969 | 6.93E-08 | 6.48E-07 | 7.69437 |
| NCAPH | 1.1034 | 2.036158 | 8.73623 | 2.16E-15 | 2.83E-13 | 24.4688 |
| IGHG2 | 1.10494 | 1.626941 | 3.45153 | 0.0007 | 0.002086 | -1.0542 |
| S100A4 | 1.10738 | 3.598466 | 4.89226 | 2.29E-06 | 1.35E-05 | 4.34129 |
| RAD51AP1 | 1.10941 | 2.402346 | 9.84205 | 2.18E-18 | 1.40E-15 | 31.1968 |
| CDC45 | 1.10963 | 1.898529 | 8.12458 | 8.63E-14 | 5.72E-12 | 20.8734 |
| FLNA | 1.11048 | 6.705289 | 6.73074 | 2.45E-10 | 5.12E-09 | 13.147 |
| CDK4 | 1.11257 | 7.126828 | 6.913 | 9.04E-11 | 2.21E-09 | 14.1138 |
| SERPINE1 | 1.12143 | 3.172314 | 4.06511 | 7.31E-05 | 0.000283 | 1.05723 |
| CIITA | 1.12322 | 2.432616 | 6.84587 | 1.31E-10 | 3.03E-09 | 13.756 |
| FANCI | 1.1236 | 2.838625 | 8.70363 | 2.63E-15 | 3.31E-13 | 24.2746 |
| F2R | 1.12382 | 4.555331 | 7.15456 | 2.36E-11 | 7.11E-10 | 15.417 |
| KIF23 | 1.12413 | 1.814443 | 8.45466 | 1.19E-14 | 1.13E-12 | 22.8006 |
| SCIN | 1.12474 | 3.16458 | 5.11101 | 8.51E-07 | 5.65E-06 | 5.28699 |
| ZWINT | 1.12541 | 3.358475 | 8.45514 | 1.19E-14 | 1.13E-12 | 22.8035 |
| SMC4 | 1.12628 | 3.626267 | 8.9848 | 4.68E-16 | 8.96E-14 | 25.9582 |
| UBD | 1.13075 | 2.204294 | 5.2977 | 3.58E-07 | 2.67E-06 | 6.11791 |
| ECM2 | 1.13081 | 3.210491 | 6.80307 | 1.65E-10 | 3.67E-09 | 13.5289 |
| HJURP | 1.13257 | 1.872924 | 8.53348 | 7.41E-15 | 7.72E-13 | 23.2655 |
| C1R | 1.13328 | 6.157131 | 5.08138 | 9.75E-07 | 6.38E-06 | 5.1571 |
| MFAP2 | 1.13654 | 1.540682 | 6.38362 | 1.57E-09 | 2.50E-08 | 11.3475 |
| CDCA8 | 1.13686 | 1.66064 | 9.04649 | 3.19E-16 | 6.76E-14 | 26.3301 |
| CD14 | 1.14374 | 5.435732 | 5.26189 | 4.23E-07 | 3.08E-06 | 5.95686 |
| CDKN2C | 1.14459 | 4.332685 | 6.9346 | 8.03E-11 | 2.00E-09 | 14.2293 |
| DLGAP5 | 1.14474 | 1.21058 | 9.3347 | 5.32E-17 | 1.66E-14 | 28.0787 |
| GBP3 | 1.14677 | 3.69046 | 6.51851 | 7.68E-10 | 1.36E-08 | 12.0401 |
| CDCA7 | 1.14806 | 2.713208 | 7.92718 | 2.78E-13 | 1.57E-11 | 19.7366 |
| CLIC1 | 1.14859 | 5.587265 | 6.74081 | 2.32E-10 | 4.90E-09 | 13.2 |
| KIF4A | 1.14947 | 1.585638 | 9.179 | 1.40E-16 | 3.71E-14 | 27.1318 |
| PDLIM1 | 1.14971 | 2.995162 | 5.85515 | 2.38E-08 | 2.60E-07 | 8.72208 |
| GEM | 1.15305 | 3.636533 | 6.51043 | 8.02E-10 | 1.41E-08 | 11.9983 |
| TEAD4 | 1.15348 | 1.879162 | 7.06752 | 3.84E-11 | 1.08E-09 | 14.9446 |
| CTSC | 1.15713 | 4.849493 | 7.37592 | 6.76E-12 | 2.42E-10 | 16.6319 |
| NNMT | 1.1572 | 2.972084 | 3.87814 | 0.00015 | 0.000535 | 0.38314 |
| KIF20A | 1.15728 | 1.535565 | 8.66328 | 3.36E-15 | 4.03E-13 | 24.0346 |
| COL6A2 | 1.16184 | 3.967817 | 5.06265 | 1.06E-06 | 6.88E-06 | 5.0753 |
| TMSB15A | 1.1624 | 3.045928 | 5.14773 | 7.19E-07 | 4.88E-06 | 5.44872 |
| SPC24 | 1.16582 | 1.981125 | 7.97235 | 2.13E-13 | 1.26E-11 | 19.9956 |
| FCGR2C | 1.1677 | 2.165822 | 5.23996 | 4.69E-07 | 3.38E-06 | 5.85859 |
| LMNB1 | 1.16814 | 3.961558 | 7.88459 | 3.56E-13 | 1.91E-11 | 19.493 |
| COL5A2 | 1.1701 | 2.832701 | 5.87102 | 2.20E-08 | 2.43E-07 | 8.79878 |
| SOCS3 | 1.177 | 2.981677 | 4.441 | 1.60E-05 | 7.45E-05 | 2.49033 |
| FPR3 | 1.18019 | 2.133437 | 7.4699 | 3.95E-12 | 1.51E-10 | 17.1535 |
| FCGBP | 1.18156 | 2.750295 | 4.6869 | 5.64E-06 | 2.98E-05 | 3.48185 |
| IGF2BP3 | 1.18912 | 1.341942 | 6.39991 | 1.44E-09 | 2.33E-08 | 11.4307 |
| CENPA | 1.18923 | 1.540672 | 9.1505 | 1.68E-16 | 4.20E-14 | 26.9591 |
| HLA-DMA | 1.2005 | 5.219035 | 6.76522 | 2.03E-10 | 4.39E-09 | 13.3287 |
| CCNA2 | 1.20089 | 2.069102 | 10.0644 | 5.29E-19 | 6.24E-16 | 32.5774 |
| HLA-DMB | 1.20478 | 4.900311 | 6.75147 | 2.19E-10 | 4.67E-09 | 13.2562 |
| BUB1 | 1.20847 | 1.881746 | 9.59352 | 1.05E-17 | 4.72E-15 | 29.6635 |
| FSTL1 | 1.21065 | 4.991492 | 7.29752 | 1.05E-11 | 3.56E-10 | 16.1994 |
| CD93 | 1.21566 | 2.667392 | 6.62625 | 4.31E-10 | 8.33E-09 | 12.5994 |
| MMP14 | 1.21731 | 4.577119 | 6.29778 | 2.47E-09 | 3.70E-08 | 10.9114 |
| CD74 | 1.21788 | 9.438264 | 5.84086 | 2.56E-08 | 2.75E-07 | 8.6531 |
| LTF | 1.21841 | 2.256784 | 3.29398 | 0.0012 | 0.003334 | -1.5484 |
| NAPSB | 1.22321 | 3.280889 | 5.4208 | 2.00E-07 | 1.63E-06 | 6.67742 |
| EZH2 | 1.22479 | 2.994425 | 8.90844 | 7.49E-16 | 1.24E-13 | 25.4991 |
| LYZ | 1.22849 | 3.480157 | 5.11652 | 8.30E-07 | 5.54E-06 | 5.31117 |
| HLA-DOA | 1.23083 | 2.9718 | 6.83796 | 1.37E-10 | 3.14E-09 | 13.714 |
| PLEKHA4 | 1.23644 | 3.763422 | 6.64808 | 3.83E-10 | 7.53E-09 | 12.7134 |
| TROAP | 1.2409 | 2.086473 | 7.83197 | 4.85E-13 | 2.48E-11 | 19.1928 |
| NCAPG | 1.24254 | 1.680513 | 9.96387 | 1.00E-18 | 8.42E-16 | 31.9522 |
| CP | 1.24539 | 3.38675 | 4.65238 | 6.55E-06 | 3.40E-05 | 3.34016 |
| CD44 | 1.25553 | 6.097367 | 5.21677 | 5.22E-07 | 3.70E-06 | 5.75507 |
| CXCL10 | 1.25733 | 1.828334 | 5.32832 | 3.10E-07 | 2.36E-06 | 6.2562 |
| HLA-DRB1 | 1.2625 | 5.279701 | 4.89492 | 2.26E-06 | 1.34E-05 | 4.35257 |
| TYMS | 1.26775 | 4.352697 | 8.28327 | 3.35E-14 | 2.66E-12 | 21.7959 |
| IQGAP2 | 1.26944 | 2.863809 | 7.77114 | 6.92E-13 | 3.40E-11 | 18.847 |
| TACC3 | 1.27012 | 3.244663 | 9.43109 | 2.91E-17 | 1.03E-14 | 28.6674 |
| ASF1B | 1.27025 | 2.113359 | 9.55177 | 1.36E-17 | 5.62E-15 | 29.407 |
| CDK2 | 1.27488 | 3.781396 | 10.4877 | 3.51E-20 | 1.22E-16 | 35.2254 |
| TGFBI | 1.27693 | 4.714059 | 5.68095 | 5.66E-08 | 5.44E-07 | 7.88917 |
| GNG12 | 1.27736 | 3.943041 | 6.94704 | 7.49E-11 | 1.89E-09 | 14.2959 |
| COL1A2 | 1.27784 | 4.255514 | 5.29564 | 3.61E-07 | 2.70E-06 | 6.1086 |
| COL1A1 | 1.2782 | 3.28128 | 4.49366 | 1.29E-05 | 6.13E-05 | 2.69913 |
| LOXL2 | 1.28248 | 2.833214 | 6.97744 | 6.33E-11 | 1.65E-09 | 14.459 |
| NUF2 | 1.28412 | 2.186247 | 10.2049 | 2.16E-19 | 4.33E-16 | 33.4536 |
| WEE1 | 1.29425 | 3.29982 | 8.66182 | 3.39E-15 | 4.05E-13 | 24.0259 |
| TNC | 1.30388 | 5.434789 | 5.965 | 1.37E-08 | 1.62E-07 | 9.25584 |
| KIF2C | 1.30533 | 1.998765 | 9.06319 | 2.88E-16 | 6.29E-14 | 26.4309 |
| CD163 | 1.30786 | 3.85104 | 5.11764 | 8.26E-07 | 5.51E-06 | 5.3161 |
| CENPK | 1.30818 | 1.992222 | 11.1375 | 5.23E-22 | 4.24E-18 | 39.3299 |
| IGKC | 1.30921 | 3.103965 | 3.40195 | 0.00083 | 0.00242 | -1.2119 |
| EMP1 | 1.31046 | 5.016288 | 6.21467 | 3.81E-09 | 5.38E-08 | 10.4926 |
| KIAA0101 | 1.31503 | 2.523579 | 8.46435 | 1.13E-14 | 1.10E-12 | 22.8577 |
| FOXM1 | 1.31681 | 3.060054 | 7.94826 | 2.45E-13 | 1.41E-11 | 19.8574 |
| SERPINH1 | 1.31928 | 4.631601 | 7.11792 | 2.90E-11 | 8.44E-10 | 15.2178 |
| HLA-DQA1 | 1.32924 | 3.230809 | 3.96287 | 0.00011 | 0.000402 | 0.68536 |
| TIMP1 | 1.33703 | 5.293564 | 4.23253 | 3.76E-05 | 0.000158 | 1.68293 |
| METTL7B | 1.33851 | 3.019085 | 5.59637 | 8.56E-08 | 7.77E-07 | 7.49094 |
| MELK | 1.33863 | 1.716496 | 9.97619 | 9.28E-19 | 8.20E-16 | 32.0287 |
| NDC80 | 1.34178 | 1.764727 | 10.3927 | 6.46E-20 | 1.97E-16 | 34.629 |
| IGHG1 | 1.36456 | 2.473354 | 3.5905 | 0.00043 | 0.001364 | -0.6017 |
| APOL4 | 1.36657 | 2.238833 | 6.87046 | 1.14E-10 | 2.71E-09 | 13.8868 |
| AURKB | 1.36716 | 2.412418 | 8.92282 | 6.86E-16 | 1.17E-13 | 25.5854 |
| TPX2 | 1.36853 | 2.843904 | 8.89614 | 8.08E-16 | 1.32E-13 | 25.4252 |
| MLF1IP | 1.3832 | 2.521636 | 10.0621 | 5.37E-19 | 6.24E-16 | 32.563 |
| TYMP | 1.38947 | 3.813007 | 6.70082 | 2.88E-10 | 5.89E-09 | 12.9897 |
| KIFC1 | 1.39741 | 2.390402 | 8.90826 | 7.50E-16 | 1.24E-13 | 25.498 |
| CHI3L1 | 1.39938 | 5.307812 | 3.03591 | 0.00277 | 0.006958 | -2.3138 |
| MSR1 | 1.40006 | 3.591883 | 7.04833 | 4.27E-11 | 1.18E-09 | 14.8409 |
| C8orf4 | 1.40221 | 3.553357 | 6.82646 | 1.45E-10 | 3.31E-09 | 13.6529 |
| FCGR3A | 1.40492 | 5.45506 | 6.44192 | 1.15E-09 | 1.92E-08 | 11.6458 |
| MGP | 1.40766 | 4.974617 | 5.01282 | 1.33E-06 | 8.38E-06 | 4.85868 |
| ANXA2 | 1.4096 | 5.968817 | 6.07324 | 7.89E-09 | 1.01E-07 | 9.78802 |
| HLA-DPB1 | 1.42405 | 5.485305 | 6.47732 | 9.57E-10 | 1.65E-08 | 11.8277 |
| CDC20 | 1.43537 | 2.518984 | 9.13492 | 1.85E-16 | 4.54E-14 | 26.8647 |
| PTTG1 | 1.45122 | 3.909567 | 9.08106 | 2.58E-16 | 5.86E-14 | 26.5389 |
| IGFBP2 | 1.45301 | 5.09478 | 5.02739 | 1.25E-06 | 7.91E-06 | 4.92185 |
| MYBL2 | 1.45677 | 2.140075 | 7.87582 | 3.75E-13 | 1.99E-11 | 19.4429 |
| PBK | 1.45909 | 2.456885 | 8.44616 | 1.26E-14 | 1.18E-12 | 22.7506 |
| PLAU | 1.46116 | 2.631763 | 6.6801 | 3.22E-10 | 6.47E-09 | 12.881 |
| GBP1 | 1.46329 | 3.91951 | 7.10078 | 3.19E-11 | 9.18E-10 | 15.1247 |
| VCAM1 | 1.46407 | 4.04329 | 6.18054 | 4.54E-09 | 6.26E-08 | 10.3217 |
| PDPN | 1.46833 | 3.680038 | 5.39022 | 2.31E-07 | 1.84E-06 | 6.53756 |
| CENPF | 1.46923 | 2.670919 | 9.0289 | 3.56E-16 | 7.16E-14 | 26.224 |
| RRM2 | 1.47139 | 2.786204 | 8.38093 | 1.86E-14 | 1.63E-12 | 22.3674 |
| GBP2 | 1.4848 | 4.1352 | 6.20107 | 4.08E-09 | 5.72E-08 | 10.4244 |
| CDK1 | 1.48993 | 2.86748 | 9.58705 | 1.09E-17 | 4.74E-15 | 29.6237 |
| BIRC5 | 1.51376 | 2.400216 | 9.06222 | 2.90E-16 | 6.29E-14 | 26.425 |
| FAM64A | 1.51586 | 2.592021 | 8.88686 | 8.55E-16 | 1.38E-13 | 25.3695 |
| CFI | 1.52893 | 3.567609 | 7.13696 | 2.61E-11 | 7.72E-10 | 15.3212 |
| CCNB2 | 1.53045 | 2.231905 | 9.42956 | 2.94E-17 | 1.03E-14 | 28.6581 |
| SERPINA3 | 1.56202 | 6.559445 | 4.28057 | 3.10E-05 | 0.000133 | 1.86623 |
| SPOCD1 | 1.56485 | 2.712142 | 5.1154 | 8.34E-07 | 5.56E-06 | 5.30628 |
| NUSAP1 | 1.58461 | 3.513829 | 8.88198 | 8.82E-16 | 1.40E-13 | 25.3403 |
| IFI30 | 1.58766 | 4.990131 | 7.24106 | 1.45E-11 | 4.70E-10 | 15.8895 |
| TFPI | 1.5897 | 2.868079 | 7.68004 | 1.18E-12 | 5.29E-11 | 18.3315 |
| HLA-DPA1 | 1.59584 | 6.894804 | 7.16719 | 2.20E-11 | 6.72E-10 | 15.4858 |
| COL4A2 | 1.61044 | 4.250267 | 6.49305 | 8.80E-10 | 1.53E-08 | 11.9087 |
| TNFRSF12A | 1.61817 | 3.614044 | 6.90141 | 9.64E-11 | 2.33E-09 | 14.0519 |
| VIM | 1.62327 | 9.142917 | 6.81493 | 1.55E-10 | 3.49E-09 | 13.5918 |
| HLA-DRA | 1.64503 | 7.349821 | 7.43617 | 4.79E-12 | 1.79E-10 | 16.9659 |
| MS4A6A | 1.68063 | 5.127183 | 6.91316 | 9.03E-11 | 2.21E-09 | 14.1146 |
| CRNDE | 1.68367 | 2.400982 | 7.40251 | 5.81E-12 | 2.12E-10 | 16.7792 |
| COL3A1 | 1.68929 | 3.101433 | 5.76314 | 3.77E-08 | 3.83E-07 | 8.28007 |
| TOP2A | 1.70583 | 3.175507 | 9.0162 | 3.85E-16 | 7.62E-14 | 26.1474 |
| UBE2C | 1.76486 | 3.332977 | 9.07826 | 2.62E-16 | 5.91E-14 | 26.522 |
| CHI3L2 | 1.77822 | 4.561093 | 4.85354 | 2.72E-06 | 1.57E-05 | 4.1771 |
| ANXA1 | 1.87874 | 5.210158 | 6.79368 | 1.74E-10 | 3.84E-09 | 13.4792 |
| COL4A1 | 1.88844 | 3.979236 | 6.99851 | 5.64E-11 | 1.50E-09 | 14.5723 |
