# Supplementary material for: MGME1 associates with poor prognosis and is vital for cell proliferation in lower-grade glioma
Source: Aging (Albany NY). 2023 May 8;15(9):3690–714. doi: 10.18632/aging.204705 (PMC10449294; doi:10.18632/aging.204705)
Supplement: Supplementary Tables 5-7 [file aging-15-204705-s006.pdf]

SUPPLEMENTARY TABLES

Supplementary Table 5. Clinical features of LGG patients from TCGA.

Clinical features		Total (477)	%
Age	Age ≤45	287	60.17%
	Age >45	190	39.83 %
Gender	Female	216	45.28%
	Male	261	54.72%
Grade	WHO II	231	48.43%
	WHO III	246	51.57%
1p/19q	Non-codel	321	67.30%
	Codel	156	32.70%
IDH	Mutant	389	81.55%
	Wildtype	85	17.82%
	Unknow	3	0.63%
MGMT	Unmethylated	82	17.19%
	Methylated	395	82.81%

Supplementary Table 6. Clinical features of LGG patients from CGGA.

Clinical features		Total (170)	%
Age	Age ≤45	129	75.88%
	Age >45	41	24.12%
Gender	Female	65	38.24%
	Male	105	61.76%
Grade	WHO II	97	57.06%
	WHO III	73	42.94%
1p/19q	Non-codel	113	66.47%
	Codel	55	32.35%
	Unknow	2	1.18%
IDH	Mutant	125	73.53%
	Wildtype	44	25.88%
	Unknow	1	0.59%
MGMT	Unmethylated	70	41.18%
	Methylated	84	49.41%
	Unknow	16	9.41%

Supplementary Table 7. Clinical features of LGG patients from GSE16011.

Clinical features		Total (102)	%
Age	Age ≤45	57	55.88%
	Age >45	45	44.12%
Gender	Female	35	34.31%
	Male	67	65.69%
Grade	WHO II	22	21.57%
	WHO III	80	78.43%
1p/19q	Non-codel	35	34.31%
	Codel	40	39.22%
	Unknow	27	26.47
IDH	Mutant	44	43.14%
	Wildtype	37	36.27%
	Unknow	21	20.59%
